# Global Analysis of Differentially Expressed Genes and Proteins in the Wheat Callus Infected by *Agrobacterium tumefacien*s

**DOI:** 10.1371/journal.pone.0079390

**Published:** 2013-11-20

**Authors:** Xiaohong Zhou, Ke Wang, Dongwen Lv, Chengjun Wu, Jiarui Li, Pei Zhao, Zhishan Lin, Lipu Du, Yueming Yan, Xingguo Ye

**Affiliations:** 1 National Key Facility of Crop Gene Resources and Genetic Improvement/Institute of Crop Sciences, Chinese Academy of Agricultural Sciences, Beijing, China; 2 Key Laboratory of Genetics and Biotechnology, College of Life Science, Capital Normal University, Beijing, China; 3 Department of Medical Biochemistry and Microbiology, Uppsala University, Uppsala, Sweden; 4 Department of Plant Pathology, Kansas State University, Manhattan, Kansas, United States of America; University of Wisconsin-Milwaukee, United States of America

## Abstract

*Agrobacterium*-mediated plant transformation is an extremely complex and evolved process involving genetic determinants of both the bacteria and the host plant cells. However, the mechanism of the determinants remains obscure, especially in some cereal crops such as wheat, which is recalcitrant for *Agrobacterium*-mediated transformation. In this study, differentially expressed genes (DEGs) and differentially expressed proteins (DEPs) were analyzed in wheat callus cells co-cultured with *Agrobacterium* by using RNA sequencing (RNA-seq) and two-dimensional electrophoresis (2-DE) in conjunction with mass spectrometry (MS). A set of 4,889 DEGs and 90 DEPs were identified, respectively. Most of them are related to metabolism, chromatin assembly or disassembly and immune defense. After comparative analysis, 24 of the 90 DEPs were detected in RNA-seq and proteomics datasets simultaneously. In addition, real-time RT-PCR experiments were performed to check the differential expression of the 24 genes, and the results were consistent with the RNA-seq data. According to gene ontology (GO) analysis, we found that a big part of these differentially expressed genes were related to the process of stress or immunity response. Several putative determinants and candidate effectors responsive to *Agrobacterium* mediated transformation of wheat cells were discussed. We speculate that some of these genes are possibly related to *Agrobacterium* infection. Our results will help to understand the interaction between *Agrobacterium* and host cells, and may facilitate developing efficient transformation strategies in cereal crops.

## Introduction

Genetic transformation, as a reverse genetics tool, has been widely used in modification of some economically important plant species. Great successes have been achieved in enhancing the production of major crops such as soybean, maize and cotton, which have contributed a lot to the global agricultural economy and helped to meet the food demand for human and animal worldwide [1]. However, almost no promising progress has occurred on genetically modified wheat [2]. Presently, the most economic strategy of plant transformation is still *Agrobacterium*- mediated method, which is progressed slowly in wheat even though it was initiated in 1980s when it was successfully applied to obtain transgenic tobacco plants [3].

The mechanism of *Agrobaterium*-mediated transformation has been explored in both pathogens and plants, and some pathogen or host proteins/genes have been identified to participate in the *Agrobacterium* infection and T-DNA delivery process [4]–[11]. A few of these genes were proved to result in improved transformation efficiency in some dicot plants such as *Arabidopsis* and tobacco, and also in several cereal plants such as rice and maize [12], [13]. Taking rice as an example, even though its transformation process is not difficult, *Agrobacterium*-mediated transformation efficiency for *indica* rice variety is much lower than that for *japonica* cultivars. Tie *et al.* identified the differentially expressed genes by microarray, and the results were very useful to identify genes involved in the process of *Agrobacterium*-mediated transformation [14].

*Agrobacterium* infection of plant cells consists of a series of events, including attachment of *Agrobacterium* on plant tissues, recognition between *Agrobacterium* and host, production of transferred substrates, transferring of the components into host cell, movement of the substrates into host nucleus, integration of T-DNA into host genome, and expression of the integrated T-DNA, among which the most vital step is the integration of T-DNA into plant genome. During the whole process, several *vir* genes and *chv* genes were proved to contribute to the cellular transportation or transformation of the target DNA fragments [15]. However, only a few literatures reported the response of host response to the infection of *Agrobacterium* by cDNA-AFLP [16] and genome microarray [17]. Tzfra *et al.* screened an *Arabidopsis* cDNA library by the yeast two-hybrid method with the *Agrobacterium* VirE2 protein as a bait and found that the identified plant protein, designated VIP1, was specifically bound with VirE2, and allowed its nuclear import to participate in the early stages of T-DNA expression [18]. Subsequent research indicated that VIP1 is imported into the nucleus of plants via the karyopherin-α dependent pathway, and its over-expression significantly rendered plants more susceptible to genetic transformation mediated by *Agrobacterium* [18], [19]. Moreover, the ability of VIP1 interacting with VirE2 protein and localizing in nucleus helped the transportation of the foreign DNA transiently into plant cells and nucleus, and its interaction with a host histone protein of H2A is required for the upcoming stable genetic transformation of the alien DNA strands [20]. VIP2 is another *Arabidopsis* protein which interacts with VIP1, and also plays an important role in the *Agrobacterium*-mediated transformation in plants [21]. Because of the complexity of the whole transformation process, a lot of host genes are postulated to participate in the delivery process. Identifying more host genes involved in the response to infection and transformation will help us to further understand the process, and improve the efficiency of *Agrobacterium*-mediated wheat transformation eventually.

However, *Agrobacterium*-mediated wheat genetic transformation has remained very low efficiency and strong genotype-dependent [22]. Therefore, particle bombardment method is still the major approach for wheat transformation [22]. Up to now, some improved transformation protocols mediated by *Agrobacterium* have been reported in wheat since 1997 [23], [24]. For example, Hu *et al.* reported that they obtained more than 3,000 independent transgenic events with average transformation efficiency of 4.4% [25]. However, these results were limited mainly to few wheat varieties, and the methods they used have been proved difficult to follow up [24]–[27] even if the advances and progress on wheat *Agrobacterium*-mediated transformation approach were described in freshly published papers [23], [28]. Indeed, no wheat variety has been proved to be competent for the transformation mediated by *Agrobacterium*. Therefore, more work needs to be conducted to find key host genes involved in the T-DNA delivery process after the wheat cells are infected by *Agrobacterium*.

In the past few years, development of next-generation sequencing (NGS) technologies has provided a new paradigm for genome and transcriptome characterization [29], [30]. RNA sequencing (RNA-seq) has exhibited some obvious advantages over existing approaches. This technique has been proved to be highly repeatable, and is expected to revolutionize the manner of analyzing eukaryotic transcriptomes [31]. On the other hand, some technologies such as mass spectrometry (MS) and two-dimensional electrophoresis (2-DE) have been widely used in proteomics. Evidences showed that proteomics and transcriptome can mutually promote the detection of expressed genes with complementary advantages at low cost [32]. In this study, the expression activities of associated genes with transformation process were analyzed in the infected wheat callus by *Agrobacterium* using RNA-seq and 2-DE in conjunction with MS strategy. We identified differentially expressed genes that might be involved in the process of *Agrobacterium* infection and T-DNA delivery. A set of 4,889 differentially expressed genes (DEGs) and 90 differentially expressed proteins (DEPs) were identified, respectively. Most of them are related to chromatin assembly or disassembly and to immune. After comparative analysis, 24 aligned DEPs were identified to be potentially closely related to *Agrobacterium* infection response and transformation, and involved in 23 pathways.

## Materials and Methods

### Plant materials and *Agrobacterium* strain

A semi-winter wheat (*Triticum aestivum* L.) variety used throughout this study, Yangmai12, which is a largely commercial wheat variety in southeast China with good agronomic characteristics and high regeneration ability of immature embryos, was kindly provided by Prof. Shunhe Chen at Yangzhou Agricultural Institute, Jiangsu Academy of Agricultural Sciences, China. Wheat immature caryopses were collected from Yangmai12 plants 12–14 days post anthesis. The immature embryos were dissected aseptically and cultured on MSD2 medium (MS inorganic salts, 2 mgl^–1^ dicamba, 3.0% sucrose, 2.4 gl^–1^ gelrite, pH 5.8) for 4 days at 25°C under dark conditions before infection by *Agrobacterium tumefaciens*. The *Agrobacterium* strain used in this study is C58C1, which harbored a binary vector pZP211 carrying a T-DNA without target gene, and was kindly provided by Dr. Tom Clemente at University of Nebraska-Lincoln, USA.

### Infection of pre-cultured immature embryos by *Agrobacterium*

*Agrobacterium tumefaciens* strain C58C1 with binary vector pZP211 was incubated overnight in 5 ml YEP medium (10 gl^–1^ tryptone, 10 gl^–1^ yeast extract, 5 gl^–1^ N_a_Cl, pH 7.0) with 50 mgl^–1^ rifampicin, 50 mgl^–1^ streptomycin, and 50 mgl^–1^ spectinomycin inside a shaker with 220 rpm at 28°C. The overnight *Agrobacterium* culture was put into 45 ml fresh YEP medium, and incubated inside a shake for 6 hours at the same conditions as above mentioned. The *Agrobacterium* cells was pelleted by centrifugation at 4500 rpm for 10 min at room temperature, and re-suspended by adding 25 ml of liquid inoculation medium WCC (1/10 MS basic medium, 4.0 mM 2-(N-morpholine)-ethane sulphonic acid (MES), 0.75 gl^–1^ MgCl_2_, 200 µM acetosyringone, 1.0% glucose, 4.0% maltose, 2.0 mgl^–1^ dicamba, 2.2 mgl^–1^ picloram, 100 mgl^–1^ casein hydrolysate (CH), pH 5.4). The cell density was adjusted to an optical density of 0.5 (OD_650_) for inoculation [24].

About 50 pre-cultured immature embryos (PCIEs) of wheat were transferred into the prepared *Agrobacterium* suspension in a petri dish (35 mm×15 mm) containing 3 ml of *Agrobacterium* culture. In total, 100 PCIEs were infected by *Agrobacterium* in two plates. Another 100 PCIEs were transferred into 6 ml 1/10 WCC as a control [24]. The inoculation was performed at room temperature for 30 min, then the cell clusters were blotted on sterile filter paper and transferred to larger plates (90 mm×20 mm) containing a piece of sterile filter paper for co-cultivation at 23–24°C in the dark for 36 hours [33]. The infection experiment was designed by three repeats, and RNA isolation was performed from every repeat.

### RNA isolation, cDNA library preparation and sequencing

Total RNA was isolated with TRIZOL (Invitrogen, Carlsbad, CA, USA) from the *Agrobacterium* infected and non-infected PCIEs, which were treated in a solution containing 200 mgl^–1^ carbenicillin disodium salt (Amresco, USA) for 10 min and then washed with sterile water for 3 times, according to the manufacturer's instructions. Then the RNA-seq were performed in BGI (Beijing Genomics Institute).

Three RNA samples from each treatment were mixed, respectively, and treated with RNase-free DNase I for 30 min at 37°C to remove residual DNA. Beads with oligo (dT) were used to isolate poly (A) mRNA. Next, the mRNA was broken into short fragments (about 200 bp) after adding fragmentation buffer. First strand cDNA was synthesized using random hexamer-primer and reverse transcriptase (Invitrogen, Carlsbad, CA, USA). The second strand cDNA was synthesized using RNase H (Invitrogen, Carlsbad, CA, USA) and DNA polymerase I (Invitrogen, Carlsbad, CA, USA) [34]. The double strand cDNA was purified with QiaQuick PCR extraction kit (Invitrogen, Carlsbad, CA, USA), and washed with EB buffer. A single adenosine was added to the cDNA using Klenowexo–fragment with dATP. Sequencing adaptors were ligated onto the repaired ends of the fragments. The required fragments were purified by agarose gel electrophoresis and enriched by PCR amplification. Finally, the library products were sequenced via Illumina HiSeq™ 2000 (Illumina, San Diego, CA, USA). All the reads sequences have been submitted to the *Sequence Read Archive, NCBI*. Accession numbers of experiment-SRX273368 run-SRR837407 for treatment group dataset, and experiment-SRX276082 run-SRR847734 for control group dataset have been given.

### Raw reads filtering and clean reads aligning with reference sequences

The original image data were transferred into sequence data by base calling, which is defined as raw data or raw reads. Before data analysis, it was prerequisite to remove the dirty raw reads. The filtering steps included (1) removing the reads with adaptors, (2) removing the reads in which unknown bases were more than 10%, and (3) removing low-quality reads (the percentage of the low-quality bases with which value≤5 was more than 50% in a read). Next, the clean reads were aligned to reference sequences using SOAPaligner/soap2 [35], and mismatches less than 2 bases were allowed in the alignment. The reference unigene or EST (Expressed sequence tags) database and annotation data were downloaded from the websites of http://compbio.dfci.harvard.edu/cgi-bin/tgi/tc_ann.pl?gudb=wheat and http://www.ncbi.nlm.nih.gov/nucest/. The ratio we used to assess the percentage of the gene coverage by reads was the quotient of the base numbers in a target gene covered by unique mapping reads divided by the total base numbers of this target gene.

### Screening and analysis of differentially expressed genes (DEGs)

The gene expression level was calculated by counting the number of reads which mapped to the reference genes. Gene expression levels were measured as reads per kilo base per million reads (RPKM) method using the formula previously described by Mortazavi *et al.* [36]. RPKM were calculated from the following formula:
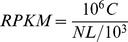



To find genes that have different expression levels between the two samples, we developed a strict algorithm according to the method reported previously [37]. If every gene's expression occupies only a small part of the whole library, *p(x)* will closely follow the Poisson distribution, in which the amount of unambiguous clean reads from gene A is denoted as x, and the probability of gene A expression is presented by *p(x)*.
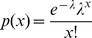



If the amount of clean reads for sample 1 and sample 2 is N_1_ and N_2_, respectively, gene A holds x reads in sample 1 and y tags in sample 2. The probability of expression quantity of gene A in sample 1 as much as in sample 2 can be calculated by the following formula:
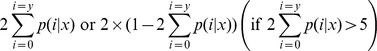


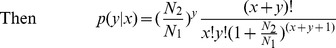



P-value corresponds to the test of differential gene expression. We threw in FDR (False discovery rate) to determine the threshold of P-value in multiple tests, and preset the FDR to a number no bigger than 0.01 [38]. The standard (FDR≤0.001 and the absolute value of |log2|ratio≥1) was used as the threshold to judge the significance of gene expression difference. More stringent criteria with smaller FDR and greater fold-change value are used to identify DEGs.

In order to remove the disturbances of the genes from *Agrobacterium*, we checked out the whole dataset and finally deleted the bacterium genes.

### Gene ontology analysis and pathway enrichment analysis of DEGs

DEGs were categorized according to the genome gene ontology (GO) annotations. GO enrichment analysis provides all GO terms which are significantly enriched in DEGs compared with the genome background and filter the DEGs that correspond to biological functions. Using this method all DEGs can be primarily mapped to GO terms in the database (http://www.geneontology.org/), calculating gene numbers for every term, then hyper geometric test was used to find significantly enriched GO terms in DEGs compared with the genome background. This analysis is able to recognize the main biological functions that DEGs play. The calculating formula is as follows:
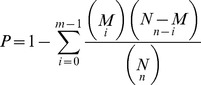



In this formula, N stands for the number of all genes with GO annotation, n for the number of DEGs in N, M for the number of all genes that are annotated to the certain GO terms, and m for the number of DEGs in M. The calculated p-value goes through Bonferroni Correction, taking corrected p-value≤0.05 as a threshold. GO terms fulfilling this condition are defined as significantly enriched GO terms in DEGs.

We also analyzed the gene functions employing pathway database, and extracted the metabolic annotation data from KEGG [39]. Significantly enriched metabolic pathways or signal transduction pathways in DEGs can be achieved using the method of enrichment analysis compared with the whole genome background. The calculating formula is the same as that in GO analysis, but here N means the number of all genes with KEGG annotation, n for the number of DEGs in N, M for the number of all genes annotated to specific pathways and m for the number of DEGs in M.

### 2-DE analysis of total protein from wheat callus infected and non-infected by *Agrobacterium*

Total protein extraction from the 3 replicated samples, respectively, was carried out following the standard protocol of TRIZOL reagent (Invitrogen, Carlsbad, CA, USA) after extraction of total RNA. Roughly 600 µg total protein from each sample was first separated by isoelectric focusing (IEF) over a pH range of 3–10 using precast first-dimension dry strip (GE Healthcare, Waukesha, WI, USA). The first-dimension strips were equilibrated in equilibration buffer (50 mM Tris-HCl (pH 8.8), 6 M urea, 30% [v/v] glycerol, 2% [w/v] SDS and trace of bromophenol blue) plus 1% DTT for 15 min, and then equilibrated in equilibration buffer plus 4% of iodoacetamide instead of 1% DTT. The equilibrated first-dimension strip was loaded on a 12% SDS-PAGE. The prepared gels were stained with colloidal Coomassie Brilliant blue G-250 (Sigma, St. Louis, MO, USA), and then different stains compared to the control were selected for MALDI-TOF/TOF analysis.

### MALDI-TOF/TOF analysis

The MALDI-TOF/TOF analysis was performed in Shanghai Applied Protein Technology Co.Ltd. Quantitative image analysis was performed with ImageMaster 2D Platinum Software Version 5.0 (Amersham Biosciences). and then the interested spots (vol.%≥2 fold and p-value≤0.05) were excised from the Coomassie Blue-stained gels for MALDI-TOF/TOF analyses, which was carried out on an ABI 4800 proteomic analyzer MALDI-TOF/TOF MS (Applied Biosystems/MDS Sciex, USA). The MS together with MS/MS spectra were searched against the NCBI non-redundant green plant database using GPS explorer software (Applied Biosystems, Grand Island, NY, USA) and MASCOT (Matrix Science, Boston, MA, USA) through the following parameters: maximum missed cleavage was 2, peptide mass tolerance was set to ±0.2 Dalton (Da), and fragment tolerance set to ±0.3 Da. The proteins with both protein score confidence interval (CI) and total ion score CI above 95% were identified as credible results for the MS/MS.

### Quantitative reverse transcription PCR (qRT-PCR) analysis

qRT-PCR was performed on ABI 7300 (ABI, Foster City, CA, USA) according to the manufacturer's instructions (TaKaRa, Dalian, China) to assess the transcription levels determined by RNA-seq and protein 2-D gel, in which *TaActin* was used as an internal standard and amplified with its genome-specific primers at the same time. The cDNA derived from the total RNA used in the process of RNA-seq was used as template. The cycle threshold values (CT) were determined through using ADP ribosylation factor (ADP) as the endogenous reference genes [40]. Next, the relative different expression ratios were calculated by the 2^−ΔΔCT^ mathematical model [41]. Each experiment was repeated by three times. Two experiments for the two independent cDNA samples were performed to confirm the reproducibility of the results.

## Results

### Summary of RNA-seq results

A total of 11,589,085 reads (567,865,165 base pairs) were obtained from the RNA of wheat callus co-cultured with *Agrobacterium tumefaciens* C58C1 (accession number of *Sequence Read Archive, NCBI*: experiment-SRX273368 run-SRR837407) and 11,601,434 reads (568,470,266 base pairs) were obtained from control callus which was not infected with C58C1 (accession number of NCBI: experiment-SRX276082 run-SRR847734). Over 95% of the reads from both samples were clean reads (File S1), and over 80% of these reads were mapped to the reference unigenes (File S2). The randomness and sequencing saturation analysis showed that the reads location on the gene was standardized to a relative position, and the number of detected genes reached saturation (File S3). The results of the gene coverage statistics are shown in File S4. In both infected and non-infected samples, more than 11% unigenes demonstrated very high levels of gene coverage (coverage>80%).

### Transcription profiles reveal DEGs between infected and non-infected samples

We used the RPKM method to identify the gene expression levels. The gene expression is calculated by the number of reads mapped to the reference sequence; the ratio of RPKM (infected)/RPKM (control) was used to determine the different expression level of each gene. According to the datasets of RPKM of 93,508 unigenes (or ESTs), compared to non-infected samples, the infected samples had 4,889 unigenes (or ESTs) showing different levels of transcription (|log2|ratio (infected/control)≥1 and FDR (false discovery rate) ≤0.001) (File S5). Among them, 2,503 unigenes were up-regulated, and 2,386 were down-regulated. The DEGs that had the mean of |log2|ratio (infected/control)≥5 are listed in Table 1 and Table 2.

**Table 1:** The differentially expressed genes (|log2|ratio (infected/control)≥5).

Gene ID	Control -RPKM	Infected -RPKM	|Log2|ratio	P-value	FDR	Description
CA594889	0.001	27.68816	14.75698	1.72E−06	0.000046	Ta cDNA clone: WT012_D04
EB512672	0.001	24.2069	14.56313	1.06E−07	3.75E−06	Blue copper-binding protein homolog
TC374940	0.001	21.75113	14.4088	0	0	Pathogenesis-related 1a
GH722147	0.001	14.4799	13.82176	1.97E−10	1.19E−08	Xylanase inhibitor precursor
TC420579	0.001	14.32455	13.8062	6.01E−12	4.65E−10	Response regulator
CA683879	0.001	14.02145	13.77535	7.96E−10	4.33E−08	Oxalate oxidase 2 precursor
TC440260	0.001	13.67654	13.73942	6.47E−09	2.94E−07	Putative disease resistance protein RGA3-like
CF133353	0.001	12.02828	13.55414	0.000014	0.000287	
CJ654139	0.001	10.98716	13.42353	2.12E−07	7.04E−06	Os07g0137900 protein
CA661174	0.001	10.48387	13.35588	1.72E−06	4.61E−05	Prion-like-(Q/n-rich)-domain-bearing protein protein 75
TC397228	0.001	10.1623	13.31094	6.47E−09	2.94E−07	
TC416495	0.001	10.04916	13.29479	3.47E−06	8.54E−05	Mannose phosphate isomerase (MPI)
TC451603	0.001	9.740984	13.24985	1.6E−09	8.17E−08	LOC496215 protein
CA693299	0.001	9.60027	13.22886	2.82E−05	0.000523	
BE414946	0.001	9.059047	13.14514	1.72E−06	0.000046	4-coumarate-CoA ligase 4CL2
BJ236765	0.001	8.983681	13.13309	1.72E−06	4.61E−05	
TC455795	0.001	8.654863	13.0793	3.47E−06	8.53E−05	
TC458503	0.001	8.165747	12.99537	2.61E−08	1.05E−06	Early nodulin protein
CJ875280	0.001	7.524698	12.87742	6.97E−06	0.000156	
CA663181	0.001	7.492188	12.87117	5.66E−05	0.00095	Os04g0630300 protein
TC442697	0.001	7.417109	12.85664	0.000014	0.000287	
CA638794	0.001	7.413078	12.85586	2.82E−05	0.000523	Protein kinase domain containing protein
TC426144	0.001	7.374602	12.84835	2.82E−05	0.000523	Vitis vinifera Chromosome chr8 scaffold_106
CA499029	0.001	7.253538	12.82447	6.97E−06	0.000156	Nitrate-induced NOI protein
BM135604	0.001	6.897282	12.75181	2.82E−05	0.000523	
CJ696409	0.001	6.893563	12.75103	3.47E−06	8.54E−05	Transcription initiation factor
TC418073	0.001	5.809657	12.50424	1.06E−07	3.75E−06	Phenylalanine ammonia-lyase
TC415483	0.001	5.710553	12.47941	4.27E−07	1.32E−05	Glucosyltransferase
TC392329	0.001	5.470306	12.41741	1.72E−06	4.61E−05	Reponse regulator 6
TC381923	0.001	4.514375	12.14031	1.72E−06	4.61E−05	Vitis vinifera Chromosome chr2 scaffold_105
TC458205	0.001	3.898996	11.92889	6.97E−06	0.000156	High molecular weight glutenin subunit
CK211359	0.001	3.700793	11.85362	5.66E−05	0.000951	NADH dehydrogenase subunit I
TC379055	0.001	2.43027	11.2469	5.66E−05	0.000951	UDP-glucosyltransferase
TC414250	5.548758	867.0767	7.287851	1.7E−06	4.56E−05	Wali6 protein
TC415670	7.672419	1009.571	7.039846	8.1E−08	2.95E−06	Wali3 protein
TC416295	3.794324	390.9363	6.686947	9.61E−06	0.000207	Wali6 protein
TC426400	3.150807	126.3979	5.326108	9.61E−06	0.000207	ATP-dependent Clp protease proteolytic subunit
TC447088	17.75787	0.001	−14.1162	2.11E−06	5.52E−05	
CA501626	13.80722	0.001	−13.7531	1.66E−05	0.000333	
CA678188	13.02965	0.001	−13.6695	2.68E−07	8.68E−06	Os10g0329400 protein
TC396751	12.65317	0.001	−13.6272	1.09E−09	5.73E−08	Histone H2B.4
TC374009	12.05666	0.001	−13.5575	2.82E−13	2.89E−11	Agrostis stolonifera Crs-1
CA606062	11.30046	0.001	−13.4641	2.11E−06	5.52E−05	Vacuolar-processing enzyme gamma- isozyme precursor
CA654969	10.32932	0.001	−13.3345	8.35E−06	0.000183	
CD931119	9.342799	0.001	−13.1896	4.2E−06	0.000101	Kinesin heavy chain
CA642440	9.315299	0.001	−13.1854	3.31E−05	0.000598	TonB-like protein
CJ536742	8.682779	0.001	−13.0839	3.31E−05	0.000598	
TC414261	6.403385	0.001	−12.6446	3.31E−05	0.000598	ERN2
TC390944	6.107886	0.001	−12.5765	8.35E−06	0.000183	Pathogenesis related protein-1
TC409167	5.647619	0.001	−12.4634	1.66E−05	0.000334	
TC441032	5.39911	0.001	−12.3985	2.11E−06	5.52E−05	Mitochondrial ATP synthase
CK205455	5.16714	0.001	−12.3352	4.2E−06	0.000101	
TC416474	5.10914	0.001	−12.3189	1.66E−05	0.000334	TBP-binding protein-like
TC432960	5.050132	0.001	−12.3021	8.35E−06	0.000183	
TC392126	4.7211	0.001	−12.2049	8.35E−06	0.000183	Germin-like protein 6a
TC434510	28.3137	0.366668	−6.27088	1.98E−22	5.33E−20	Peroxidase PXC2 precursor

**Table 2:** GO analysis for DEGs (|log2|ratio (infected/control)≥5).

Gene ID	Cellular component	Molecular function	Biological process
TC374940	0016023		
TC420579	0005575 0005739	0030528 0000156	0006355 0009736 0000160 0019827 0009735
TC416495	0009536		
TC458503	0009536	0003674	
TC418073	0005634 0000786	0003677	0006334 0007283 0007076
TC415483	0005739 0016023		
TC392329	0005575 0005634 0005739	0003677 0030528 0000156	0006355 0006950 0009736 0019827 0000160 0009735
TC381923	0005575 0016023	0016706	0019748
TC458205	0009536	0003677	0006310
TC396751	0008021 0042589 0030141 0031201 0030672 0005576 0016021 0000786 0005575 0005634	0000149 0003677 0003674 0005516 0017075 0005543 0017022	0017157 0008285 0006334 0008150 0050829 0017156 0006944 0009792 0016079 0050830 0051276 0006955 0040007 0002119
TC390944	0005576 0016023	0003674	0008150
TC409167	0016023		
TC392126	0016023		0008150

**Figure 1:**
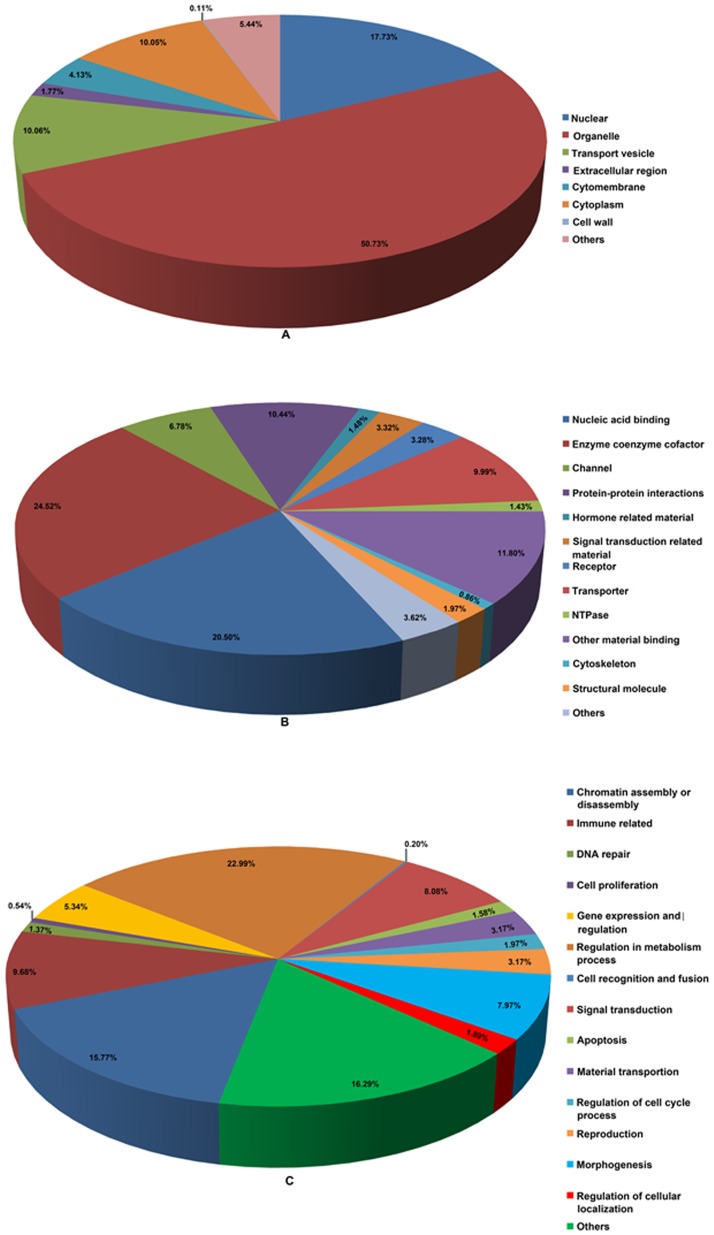
Categorization of the GO terms based on the differentially expressed genes. Categorization of GO terms with a p-value greater than or equal to 1: cellular component terms (A), molecular function terms (B) and biological process terms (C). All of the differentially expressed genes were classified based on GO analysis. By this method all DEGs are firstly mapped to GO terms in the database (http://www.geneontology.org/), calculating gene numbers for every term, then using hypergeometric test to find significantly enriched GO terms in DEGs comparing to the genome background. Each category is labeled with different colors, and the numbers refer to ratio of these categories to the all dataset.

Furthermore, we classified the differentially expressed unigenes (or ESTs) by transcribed genes into three GO categories: cellular component, molecular function, and biological process. All of the differentially expressed unigenes shared 2,020 GO terms, including 289 cellular component terms (File S6), 382 molecular function terms (File S7), and 1,349 biological processes (File S8). To demonstrate further relationships of the DEGs and the biochemical processes occurring in the infection, the DEGs were categorized into 8, 13, and 15 groups according to cellular component, molecular function, and biological process independently (Figure 1). Based on the results of gene ontology analysis, most of the DEGs were related to various organelles (50.73%). For examples, lots of them were related to mitochondria, and about half of the DEGs had function of enzyme, coenzyme, or cofactor (24.52%) and the rests were related to the function of the unclear binding (20.5%). For the biological process of GO term, about a quarter of DEGs were involved in the metabolism process (22.9%), 15.77% of DEGs were involved in the chromatin assembly or disassembly process, and another 9.68% of DEGs were related to the process of immunity.

According the analysis of pathway enrichment, 2295 DEGs were involved in 111 pathways (Table 3). Distribution of all DEGs in the pathways was shown in File S9. 507 DEGs were related to metabolic pathways, and account for the largest portion (16.72%). However, these DEGs were not mapped in KEGG database and the metabolic pathways they were involved were quite broad, and almost incorporated all aspects of the metabolic processes, such as starch and sucrose metabolism and fatty acid metabolism. Most of the DEGs that were classed into metabolic pathways were found also to be presented in other pathways. For example, phenylalanine ammonia-lyase (TC418073) was involved in metabolic pathways, but it also participated in phenylalanine metabolism, phenylpropanoid biosynthesis and nitrogen metabolism when the function of this enzyme was concretely implemented. Besides metabolic pathways, the most important bioprocess in *Agrobacterium* response is biosynthesis of secondary metabolites (9.74%). Furthermore, the rate of the pathways on plant-pathogen interaction, phenylpropanoid biosynthesis and spliceosome were more than 3%. The most weakly tested pathways were about biotin metabolism, arachidonic acid metabolism, photosynthesis and photosynthesis-antenna proteins (0.03%).

**Table 3:** Pathway analysis of DEGs based on KEGG database.

Pathway	Annotation (number)	Annotation rate (%)	P-value	Q-value	Pathway ID
Metabolic pathways	507	16.72	1	1.00E+00	ko01100
Biosynthesis of secondary metabolites	287	9.47	0.999988	1.00E+00	ko01110
Plant-pathogen interaction	111	3.66	0.940559	1.00E+00	ko04626
Phenylpropanoid biosynthesis	102	3.36	0.078706	3.49E−01	ko00940
Spliceosome	95	3.13	0.008077	5.27E−02	ko03040
Ribosome	85	2.80	1	1.00E+00	ko03010
Starch and sucrose metabolism	79	2.61	0.082119	3.51E−01	ko00500
Purine metabolism	73	2.41	2.22E−06	4.93E−05	ko00230
Phenylalanine metabolism	72	2.37	0.007202	5.00E−02	ko00360
Protein processing in endoplasmic reticulum	67	2.21	0.998354	1.00E+00	ko04141
Glutathione metabolism	65	2.14	0.002017	2.24E−02	ko00480
Ubiquitin mediated proteolysis	51	1.68	0.149354	5.42E−01	ko04120
Endocytosis	50	1.65	0.006456	4.78E−02	ko04144
Amino sugar and nucleotide sugar metabolism	49	1.62	0.024959	1.39E−01	ko00520
Pyrimidine metabolism	48	1.58	0.004626	3.67E−02	ko00240
DNA replication	41	1.35	1.32E−11	1.47E−09	ko03030
Alpha-Linolenic acid metabolism	39	1.29	0.000108	1.50E−03	ko00592
RNA degradation	38	1.25	0.003272	2.79E−02	ko03018
Nucleotide excision repair	36	1.19	3.35E−06	6.20E−05	ko03420
Peroxisome	35	1.15	0.774472	1.00E+00	ko04146
Flavonoid biosynthesis	34	1.12	0.539443	1.00E+00	ko00941
Cysteine and methionine metabolism	33	1.09	0.971505	1.00E+00	ko00270
Glycolysis/Gluconeogenesis	33	1.09	1	1.00E+00	ko00010
Nitrogen metabolism	32	1.06	0.220608	7.65E−01	ko00910
Galactose metabolism	31	1.02	0.021792	1.27E−01	ko00052
Phagosome	31	1.02	0.999595	1.00E+00	ko04145
ABC transporters	30	0.99	3.41E−10	1.89E−08	ko02010
Base excision repair	29	0.96	6.56E−07	2.19E−05	ko03410
Cyanoamino acid metabolism	29	0.96	0.110194	4.37E−01	ko00460
Stilbenoid, diarylheptanoid and gingerol biosynthesis	29	0.96	0.693333	1.00E+00	ko00945
Tryptophan metabolism	26	0.86	0.4863	1.00E+00	ko00380
Oxidative phosphorylation	26	0.86	1	1.00E+00	ko00190
Fructose and mannose metabolism	25	0.82	0.903134	1.00E+00	ko00051
Zeatin biosynthesis	23	0.76	0.002555	2.58E−02	ko00908
Carbon fixation in photosynthetic organisms	23	0.76	1	1.00E+00	ko00710
Mismatch repair	22	0.73	8.79E−06	1.39E−04	ko03430
Phosphatidylinositol signaling system	22	0.73	0.133323	5.10E−01	ko04070
Limonene and pinene degradation	21	0.69	0.899491	1.00E+00	ko00903
RNA polymerase	20	0.66	0.001317	1.62E−02	ko03020
Selenoamino acid metabolism	20	0.66	0.353576	1.00E+00	ko00450
Pyruvate metabolism	20	0.66	0.99903	1.00E+00	ko00620
Fatty acid metabolism	19	0.63	0.784428	1.00E+00	ko00071
Sulfur metabolism	17	0.56	0.003233	2.79E−02	ko00920
Inositol phosphate metabolism	17	0.56	0.362768	1.00E+00	ko00562
Aminoacyl-tRNA biosynthesis	17	0.56	0.420217	1.00E+00	ko00970
Alanine, aspartate and glutamate metabolism	17	0.56	0.941328	1.00E+00	ko00250
Citrate cycle (TCA cycle)	17	0.56	0.989953	1.00E+00	ko00020
Circadian rhythm - plant	16	0.53	0.68574	1.00E+00	ko04712
Arginine and proline metabolism	15	0.49	0.998069	1.00E+00	ko00330
Linoleic acid metabolism	14	0.46	0.468498	1.00E+00	ko00591
Proteasome	14	0.46	0.999554	1.00E+00	ko03050
Homologous recombination	13	0.43	0.054938	2.54E−01	ko03440
Glycerolipid metabolism	13	0.43	0.888763	1.00E+00	ko00561
Caffeine metabolism	12	0.40	7.91E−07	2.19E−05	ko00232
Valine, leucine and isoleucine biosynthesis	12	0.40	0.430602	1.00E+00	ko00290
Biosynthesis of unsaturated fatty acids	12	0.40	0.82953	1.00E+00	ko01040
Benzoxazinoid biosynthesis	12	0.40	0.921449	1.00E+00	ko00402
Tyrosine metabolism	12	0.40	0.927114	1.00E+00	ko00350
Pentose phosphate pathway	12	0.40	0.999553	1.00E+00	ko00030
Glyoxylate and dicarboxylate metabolism	12	0.40	1	1.00E+00	ko00630
Sphingolipid metabolism	11	0.36	0.050595	2.50E−01	ko00600
N-Glycan biosynthesis	10	0.33	0.38074	1.00E+00	ko00510
Glycerophospholipid metabolism	10	0.33	0.985818	1.00E+00	ko00564
Ascorbate and aldarate metabolism	10	0.33	0.997898	1.00E+00	ko00053
Other glycan degradation	9	0.30	0.01401	8.64E−02	ko00511
SNARE interactions in vesicular transport	9	0.30	0.604893	1.00E+00	ko04130
Fatty acid biosynthesis	9	0.30	0.756422	1.00E+00	ko00061
Terpenoid backbone biosynthesis	9	0.30	0.791906	1.00E+00	ko00900
Lysine degradation	9	0.30	0.87531	1.00E+00	ko00310
Butanoate metabolism	9	0.30	0.974329	1.00E+00	ko00650
Glycine, serine and threonine metabolism	9	0.30	0.976647	1.00E+00	ko00260
Pantothenate and CoA biosynthesis	8	0.26	0.381927	1.00E+00	ko00770
Valine, leucine and isoleucine degradation	8	0.26	0.996475	1.00E+00	ko00280
Ether lipid metabolism	7	0.23	0.386485	1.00E+00	ko00565
Tropane, piperidine and pyridine alkaloid biosynthesis	7	0.23	0.474718	1.00E+00	ko00960
Diterpenoid biosynthesis	7	0.23	0.515453	1.00E+00	ko00904
Regulation of autophagy	7	0.23	0.696261	1.00E+00	ko04140
Natural killer cell mediated cytotoxicity	7	0.23	0.788938	1.00E+00	ko04650
Propanoate metabolism	7	0.23	0.99625	1.00E+00	ko00640
Indole alkaloid biosynthesis	6	0.20	0.099823	4.10E−01	ko00901
Basal transcription factors	6	0.20	0.892823	1.00E+00	ko03022
Pentose and glucuronate interconversions	6	0.20	0.975639	1.00E+00	ko00040
Phenylalanine, tyrosine and tryptophan biosynthesis	6	0.20	0.980414	1.00E+00	ko00400
Flavone and flavonol biosynthesis	6	0.20	0.982781	1.00E+00	ko00944
Protein export	6	0.20	0.993342	1.00E+00	ko03060
Isoquinoline alkaloid biosynthesis	5	0.16	0.70927	1.00E+00	ko00950
Glucosinolate biosynthesis	5	0.16	0.811041	1.00E+00	ko00966
Porphyrin and chlorophyll metabolism	5	0.16	0.999916	1.00E+00	ko00860
Non-homologous end-joining	4	0.13	0.03947	2.09E−01	ko03450
Ubiquinone and other terpenoid- quinone biosynthesis	4	0.13	0.966216	1.00E+00	ko00130
Histidine metabolism	4	0.13	0.982783	1.00E+00	ko00340
Steroid biosynthesis	4	0.13	0.987955	1.00E+00	ko00100
beta-Alanine metabolism	4	0.13	0.998679	1.00E+00	ko00410
Glycosphingolipid biosynthesis - ganglio series	3	0.10	0.151237	5.42E−01	ko00604
Glycosaminoglycan degradation	3	0.10	0.340238	1.00E+00	ko00531
Lysine biosynthesis	3	0.10	0.625386	1.00E+00	ko00300
One carbon pool by folate	3	0.10	0.899855	1.00E+00	ko00670
Carotenoid biosynthesis	3	0.10	0.960924	1.00E+00	ko00906
Anthocyanin biosynthesis	2	0.07	0.051858	2.50E−01	ko00942
Vitamin B6 metabolism	2	0.07	0.431126	1.00E+00	ko00750
C5-Branched dibasic acid metabolism	2	0.07	0.497579	1.00E+00	ko00660
Glycosphingolipid biosynthesis - globo series	2	0.07	0.622102	1.00E+00	ko00603
Folate biosynthesis	2	0.07	0.684606	1.00E+00	ko00790
Synthesis and degradation of ketone bodies	2	0.07	0.684606	1.00E+00	ko00072
Thiamine metabolism	2	0.07	0.697287	1.00E+00	ko00730
Nicotinate and nicotinamide metabolism	2	0.07	0.793251	1.00E+00	ko00760
Polyketide sugar unit biosynthesis	2	0.07	0.95041	1.00E+00	ko00523
Biotin metabolism	1	0.03	0.658762	1.00E+00	ko00780
Arachidonic acid metabolism	1	0.03	0.982434	1.00E+00	ko00590
Photosynthesis	1	0.03	1	1.00E+00	ko00195
Photosynthesis - antenna proteins	1	0.03	1	1.00E+00	ko00196

### Identification and classification of differentially expressed proteins

**Figure 2:**
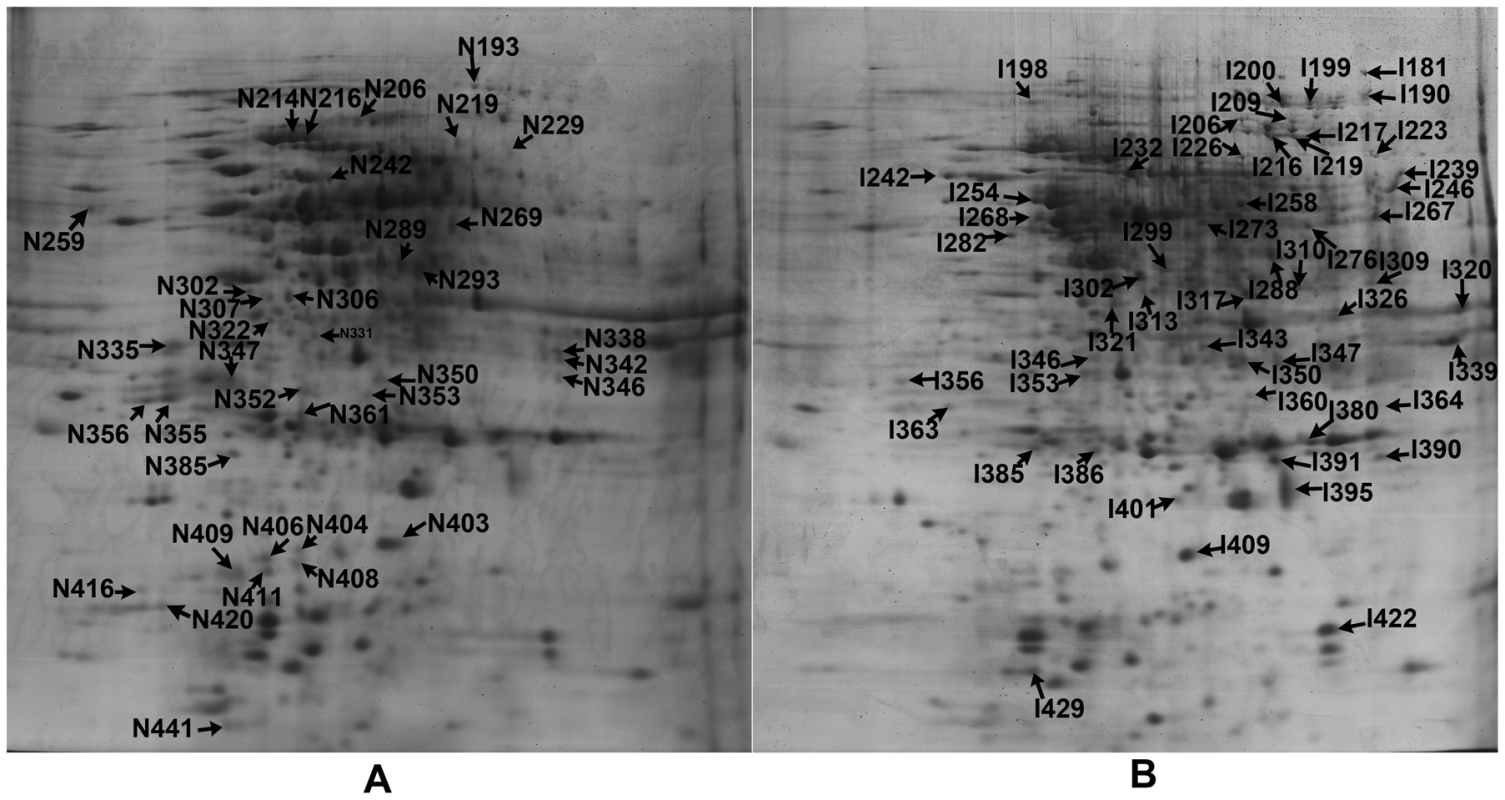
2-DE patterns of proteins extracted from control callus (A) and infected callus (B). 2-DE patterns of proteins extracted from CK (uninfected) PCIEs (A) and infected PCIEs (B). A protein sample of 1200 µg was loaded on each IPG strip (pH 3–10) and protein spots were visualized using coomassie brilliant blue staining. The experiment was repeated three times, 132 differentially expressed protein spots showing significant volume change under *Agrobacterium* infection. 90 protein spots which were changed by more than two folds were identified by MALDI-TOF/TOF analysis, and labeled on the figure.

Soluble proteins were extracted from infected and non-infected samples, and proteomic dynamics was investigated by high-resolution 2-DE. Protein spots displaying reproducible patterns were identified, and their expression patterns were analyzed. Among the *Agrobacterium*-infected and the control samples, a total of 867 reproducible protein spots were detected. The expression abundances (vol.%) of 132 protein spots changed by more than two folds, and thus were treated as DEPs. Due to the limited number of protein entries in the database, only 90 proteins spots were identified eventually through MALDI-TOF/TOF (Table 4). The maps are shown in Figure 2. Among these proteins, nine potential isoforms were targeted, and each of them had two or three spots located at different positions in the same gel. For example, in the infected tissues spots I216, I217 and I219 were identified as methionine synthase 1, and spots I320 and I343 were fructose-bisphosphate aldolase. In non-infected tissues, the isoforms include fructose-bisphosphate aldolase GTPase-activating protein-binding protein 1-like (spots N214 and N216), phosphoglucomutase (spots N219 and N229), predicted: pyruvate dehydrogenase E1 component subunit beta (spots N307 and N306), elongation factor 1-beta (spots N356 and N355), and glutathione transferase (spots I385 and I386). Furthermore, there were two groups of unnamed protein (spots N322 and N331/N338 and N342). These isoforms might represent post-translational modification forms of the same protein (Table 4).

**Table 4:** Differentially expressed proteins under control and infected conditions.

Group ID	I/C (rate of % vol.)	Accession No.	Protein score	Protein score C. I. %	Total ion score C. I. %	Protein name
I198	0.111434/0	gi|42391858	200	100	100	cold shock domain protein 3
I206	0.094232/0	gi|222834414	172	100	100	predicted protein
I223	0.045147/0	gi|158513193	468	100	100	pyruvate decarboxylase isozyme 2
I216(I217 I219)	0.210277/0	gi|68655495	159	100	100	methionine synthase 1 enzyme
I209	0.135266/0	gi|313661595	503	100	100	lipoxygenase-1
I181	0.079847/0	gi|326510251	394	100	100	predicted protein
I190	0.191266/0	gi|326514130	324	100	99.998	predicted protein
I226	0.118491/0	gi|357113565	483	100	100	predicted: succinate dehydrogenase, ubiquinone, flavoprotein subunit, mitochondrial
I267	0.481638/0	gi|14018051	466	100	100	putative alanine aminotransferase
I239	0.166667/0	gi|119388723	486	100	100	alcohol dehydrogenase ADH1A
I276	0.23262/0	gi|6561606	149	100	100	ATPase alpha subunit
I288	0.937654/0	gi|57635161	235	100	100	peroxidase 8
I299	0.189651/0	gi|129916	138	100	85.61	phosphoglycerate kinase
I302	0.466078/0	gi|326527793	893	100	100	predicted protein
I313	0.129406/0	gi|4158232	322	100	100	glycosylated polypeptide
I317	0.488077/0	gi|229358240	731	100	100	cytosolic malate dehydrogenase
I326	0.217391/0	gi|226316439	420	100	100	fructose-bisphosphate aldolase
I309	0.473017/0	gi|326492375	150	100	99.962	predicted protein
I320 (I343)	0.812175/0	gi|300681519	317	100	100	fructose-bisphosphate aldolase, chloroplast precursor
I339	0.634438/0	gi|326497973	746	100	100	predicted protein
I347	0.301374/0	gi|159895412	329	100	100	NADPH-dependent thioredoxin reductase isoform 2
I346	0.236009/0	gi|242058197	104	99.996	100	hypothetical protein
I242	0.288839/0	gi|585032	399	100	100	cysteine synthase
I364	0.409977/0	gi|326492319	278	100	100	predicted protein
I360	0.122988/0	gi|357133190	137	100	99.907	predicted protein
I390	0.47657/0	gi|20067415	384	100	100	glutathione transferase
I429	0.479179/0	gi|728594	218	100	100	glycine rich protein, RNA binding protein
I310	6.23836	gi|146216737	367	100	100	SGT1
I254	5.987146	gi|222872490	136	100	100	predicted protein
I350	5.771895	gi|326505660	177	100	100	predicted protein
I395	3.330519	gi|27544804	405	100	100	phospholipid hydroperoxide glutathione peroxidase
I232	3.201333	gi|133872360	551	100	100	Bp2A protein, partial
I409	3.036805	gi|9230743	64	58.026	100	sucrose synthase-2
I391	2.829641	gi|259017810	274	100	100	dehydroascorbate reductase
I199	2.5657	gi|108862362	146	100	100	oxidoreductase, zinc-binding dehydrogenase family
I200	2.513553	gi|49425361	382	100	100	spermidine synthase
I246	2.456281	gi|11124572	399	100	100	triosephosphat-isomerase
I380	2.320409	gi|3688398	483	100	100	ascorbate peroxidase
I353	2.202462	gi|357158835	501	100	100	predicted: glucose-6-phosphate isomerase-like
I422	2.117428	gi|326494858	187	100	100	predicted protein
N193	0/0.080562	gi|326495130	503	100	100	predicted protein
N214 (N216)	0/0.835538	gi|357167359	261	100	100	predicted: ras GTPase-activating protein-binding
N206	0/0.384636	gi|326533372	365	100	100	predicted protein
N219(N229)	0/0.303445	gi|18076790	612	100	100	phosphoglucomutase
N259	0/0.195904	gi|164565159	418	100	100	ribulose-1,5-bisphosphate carboxylase/oxygenase
N242	0/0.121703	gi|212275097	224	100	100	uncharacterized protein
N269	0/0.190315	gi|357110692	510	100	100	6-phosphogluconate dehydrogenase,decarboxylating
N289	0/0.278852	gi|28172907	510	100	100	cytosolic 3-phosphoglycerate kinase
N293	0/0.557188	gi|326500176	428	100	100	predicted protein
N302	0/0.055462	gi|326528557	521	100	100	predicted protein
N307 (N306)	0/0.242551	gi|357148637	458	100	100	pyruvate dehydrogenase E1 component subunit beta, mitochondrial
N335	0/0.869609	gi|326512374	585	100	100	predicted protein
N347	0/0.470007	gi|326506676	275	100	100	predicted protein
N322 (N331)	0/0.090235	gi|326499686	327	100	100	predicted protein
N338 (N342)	0/0.120011	gi|326518738	542	100	100	predicted protein
N356 (N355)	0/0.645651	gi|232033	219	100	100	elongation factor 1-beta
N352	0/0.393733	gi|326507956	314	100	100	predicted protein
N361	0/0.381971	gi|15808779	273	100	100	ascorbate peroxidase
N350	0/0.479167	gi|18146827	527	100	100	chitinase 2
N353	0/0.119597	gi|326489985	227	100	100	predicted protein
N346	0/0.221933	gi|357130336	190	100	100	26S proteasome non-ATPase regulatory subunit 14
N385	0/0.214757	gi|2499932	741	100	100	adenine phosphoribosyl transferase 1
N416	0/0.113163	gi|125548641	115	100	96.242	hypothetical protein OsI_16233
N420	0/0.261293	gi|326534206	224	100	100	predicted protein
N409	0/0.977368	gi|326497111	567	100	100	predicted protein
N406	0/0.44138	gi|22535646	81	99.143	99.653	hypothetical protein
N411	0/0.264197	gi|48475065	80	99.016	75.904	contains ubiquitin carboxyl-terminal hydrolase
N404	0/0.154189	gi|326496833	161	100	100	predicted protein
N408	0/0.278555	gi|326517577	81	99.143	81.951	predicted protein
N403	0/1.02402	gi|40363759	561	100	100	putative glycine-rich protein
N441	0/0.226741	gi|8980491	116	100	100	thioredoxin h
I321	0.492256	gi|40317418	158	100	99.997	glutamine synthetase isoform GSr2
I356	0.398343	gi|300807845	210	100	100	profilin
I282	0.340188	gi|326499079	236	100	100	predicted protein
I385 (I386)	0.329162	gi|20067423	293	100	100	glutathione transferase
I268	0.296689	gi|525291	1,030	100	100	ATP synthase beta subunit
I258	0.282746	gi|9408184	147	100	100	F0-F1 ATPase alpha subunit
I401	0.181083	gi|112821176	217	100	100	hypothetical protein
I273	0.169152	gi|164422240	545	100	100	ATP1
I363	0.099712	gi|40781605	530	100	100	14-3-3 protein

I/C: infected/control. C.I.: chemical ionization; I: infected; N: non-infected.

### Comparative analysis between the results of RNA-seq and proteomics

**Figure 3:**
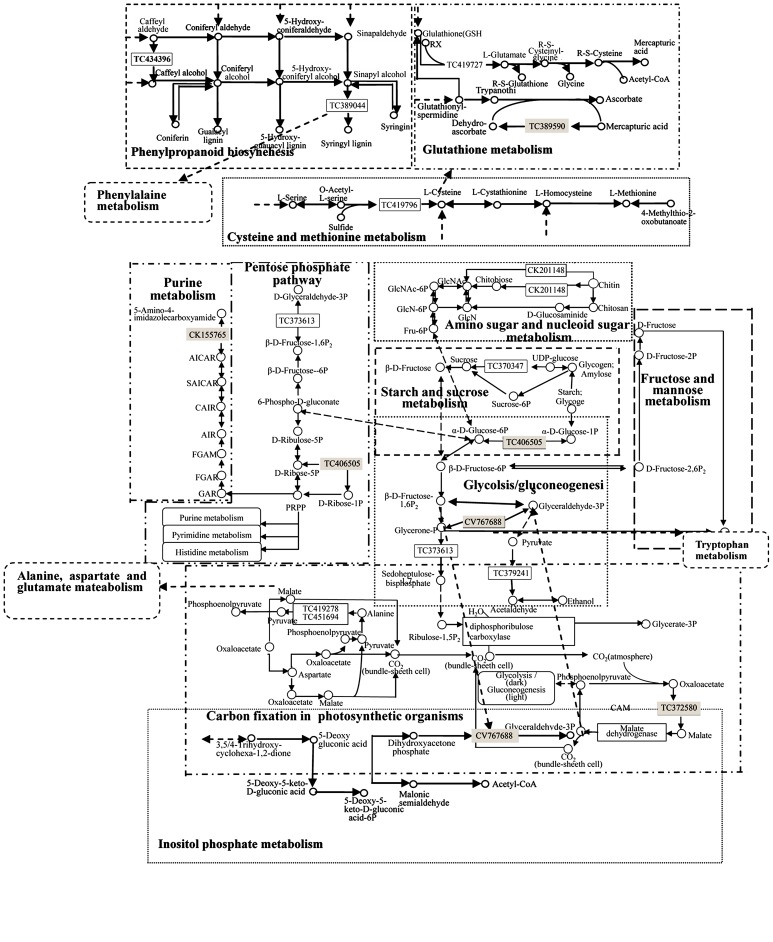
Pathway network consist by the 18 DEPs out of the aligned ones. Pathway network constructed based on the pathways of the 18 from the 24 aligned DEPs in both RNA-seq and proteomics datasets. Up-regulated genes are colored as black box, down-regulated genes as gray. Dashed arrow means that some steps are omitted, and solid arrows means the direct processes.

To describe the differently expressed genes in the process of transcription more accurately, we compared the results of RNA-seq with proteomics. 24 DEPs (26 spots) from the proteomics dataset were in consistent with the RNA-seq dataset (Table 5). On the basis of the pathway analysis of DEGs, the aligned 24 DEPs were involved in 23 pathways (Table 5), which are shown in Figure 3.

**Table 5:** The compared proteins between proteinomics and RNA-seq datasets.

Protein spot ID (rate of % vol.)	Gene ID (|log2|ratio)	Pathways
gi|42391858 (0.111434/0)	BE431040 (2.13714081)	
gi|158513193 (0.045147/0)	TC379241 (1.108033409), TC385701 (1.068969307), TC419215 (1.014521523)	Tryptophan metabolism, Glycolysis/Gluconeogenesis, Metabolic pathways (no map in kegg database)
gi|313661595 (0.135266/0)	CA733413 (2.196724855), TC388136 (2.156815406)	Alpha-Linolenic acid metabolism, Linoleic acid metabolism, Metabolic pathways (no map in kegg database)
gi|357113565 (0.118491/0)	TC384162 (−1.03191405)	
gi|14018051 (0.481638/0)	TC419278 (1.587471506), TC451694 (1.49691429)	Alanine, aspartate and glutamate metabolism, Metabolic pathways (no map in kegg database), Carbon fixation in photosynthetic organisms
gi|119388723 (0.166667/0)	TC434396 (1.831048091), TC383270 (1.529094696)	Phenylpropanoid biosynthesis, Biosynthesis of secondary metabolites (no map in kegg database), Metabolic pathways (no map in kegg database)
gi|9408184 (0.282746)	TC460547 (3.29707538)	
gi|57635161 (0.937654/0)	TC389044 (1.315388003)	Phenylalanine metabolism, Phenylpropanoid biosynthesis, Metabolic pathways (no map in kegg database), Biosynthesis of secondary metabolites (no map in kegg database)
gi|4158232 (0.129406/0)	TC369736 (−1.380338094)	
gi|229358240 (0.488077/0)	TC457520 (3.014521523), TC372580 (−1.122982)	Citrate cycle (TCA cycle), Pyruvate metabolism, Biosynthesis of secondary metabolites (no map in kegg database), Glyoxylate and dicarboxylate metabolism, Metabolic pathways (no map in kegg database), Carbon fixation in photosynthetic organisms
gi|585032 (0.288839/0)	TC419796 (2.599484024), TC373702 (1.751487117), TC376351 (1.501967518)	Sulfur metabolism, Cyanoamino acid metabolism, Selenoamino acid metabolism, Cysteine and methionine metabolism, Metabolic pathways (no map in kegg database)
gi|357133190 (0.122988/0)	TC420420 (−1.340011237) TC397562 (−1.160565183)	Proteasome
gi|40317418 (0.492256)	TC419727 (3.101984365), TC369687 (2.059845514)	Glutathione metabolism
gi|728594 (0.479179/0)	TC400906 (1.599484024)	
gi|18076790 (0/0.303445)	TC406505 (−1.090831477)	Purine metabolism, Galactose metabolism, Amino sugar and nucleotide sugar metabolism, Starch and sucrose metabolism, Pentose phosphate pathway, Biosynthesis of secondary metabolites (no map in kegg database), Glycolysis/Gluconeogenesis, Metabolic pathways (no map in kegg database)
gi|232033 (0/0.645651)	TC376420 (−1.000125253)	
gi|15808779 (0/0.381971)	TC389590 (−1.052592673)	Glutathione metabolism, Ascorbate and aldarate metabolism
gi|18146827 (0/0.479167)	CK201148 (1.325723212)	Amino sugar and nucleotide sugar metabolism
gi|2499932 (0/0.214757)	CK155765 (−1.3639901)	Purine metabolism, Metabolic pathways (no map in kegg database)
gi|9230743 (3.036805)	TC370347 (1.175549678)	Starch and sucrose metabolism, Metabolic pathways (no map in kegg database)
gi|8980491 (0/0.226741)	TC396636 (−1.064190453)	
gi|525291 (0.296689)	TC411471 (−1.32428039)	Oxidative phosphorylation, Metabolic pathways (no map in kegg database)
gi|11124572 (2.456281)	CV767688 (−1.491369406)	Inositol phosphate metabolism, Fructose and mannose metabolism, Biosynthesis of secondary metabolites (no map in kegg database), Glycolysis/Gluconeogenesis, Metabolic pathways (no map in kegg database), Carbon fixation in photosynthetic organisms
gi|27544804 (3.330519)	CD908771 (1.284973401), CA729147 (2.336449618)	

**Figure 4:**
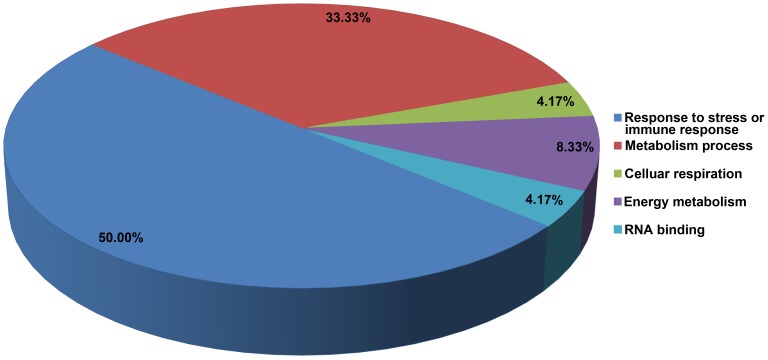
Categorization of the 24 aligned DEPs in proteomics dataset with the RNA-seq dataset. Categorizing of the 24 aligned DEPs based on their functions. Each category is labeled with the different colors, and the numbers means the percentage of each DEPs to total aligned DEPs.

According to their functions, the aligned proteins were categorized into 5 groups (Figure 4). Half of these proteins (12 proteins) were involved in stress or immune responses, including cold shock domain protein 3, pyruvate decarboxylase isozyme 2, alanine aminotransferase 2, alcohol dehydrogenase ADH1A, peroxidase 8, cysteine synthase, glutamine synthetase isoform GSr2, elongation factor 1-beta, ascorbate peroxidase, thioredoxin h, hospholipid hydroperoxide glutathione peroxidase, and chitinase 2. Eight proteins (33.3%) are related to the process of metabolism, including lipoxygenase-1, cytosolic malate dehydrogenase, proteasome subunit alpha type-3-like, phosphoglucomutase, adenine phosphoribosyltransferase 1, reversibly glycosylated polypeptide and sucrose synthase-2, and triosephosphat-isomerase. In addition, succinate dehydrogenase is a key enzyme of the respiratory chain [42]. F0-F1 ATPase alpha subunit and ATP synthase beta subunit are involved in the energy metabolism, and glycine-rich protein or RNA binding protein is a kind of nucleic acid binding protein.

For these 24 proteins, their variations on up/down-regulation in proteomics and RNA-seq datasets are not completely consistent (Table 5). Succinate dehydrogenase and triosephosphat-isomerase displayed up-regulation in the proteomics dataset but down-regulation in the RNA-seq dataset. On the contrary, glutamine synthetase isoform GSr2, F0-F1 ATPase alpha subunit and chitinase 2 showed down-regulation in the proteomics dataset but up-regulation in the RNA-seq dataset. This inconsistent phenomenon might be caused by post-translational modification of the target gene and different metabolism process of the corresponding protein.

### Expression analysis using quantitative reverse transcription PCR (qRT-PCR)

**Figure 5:**
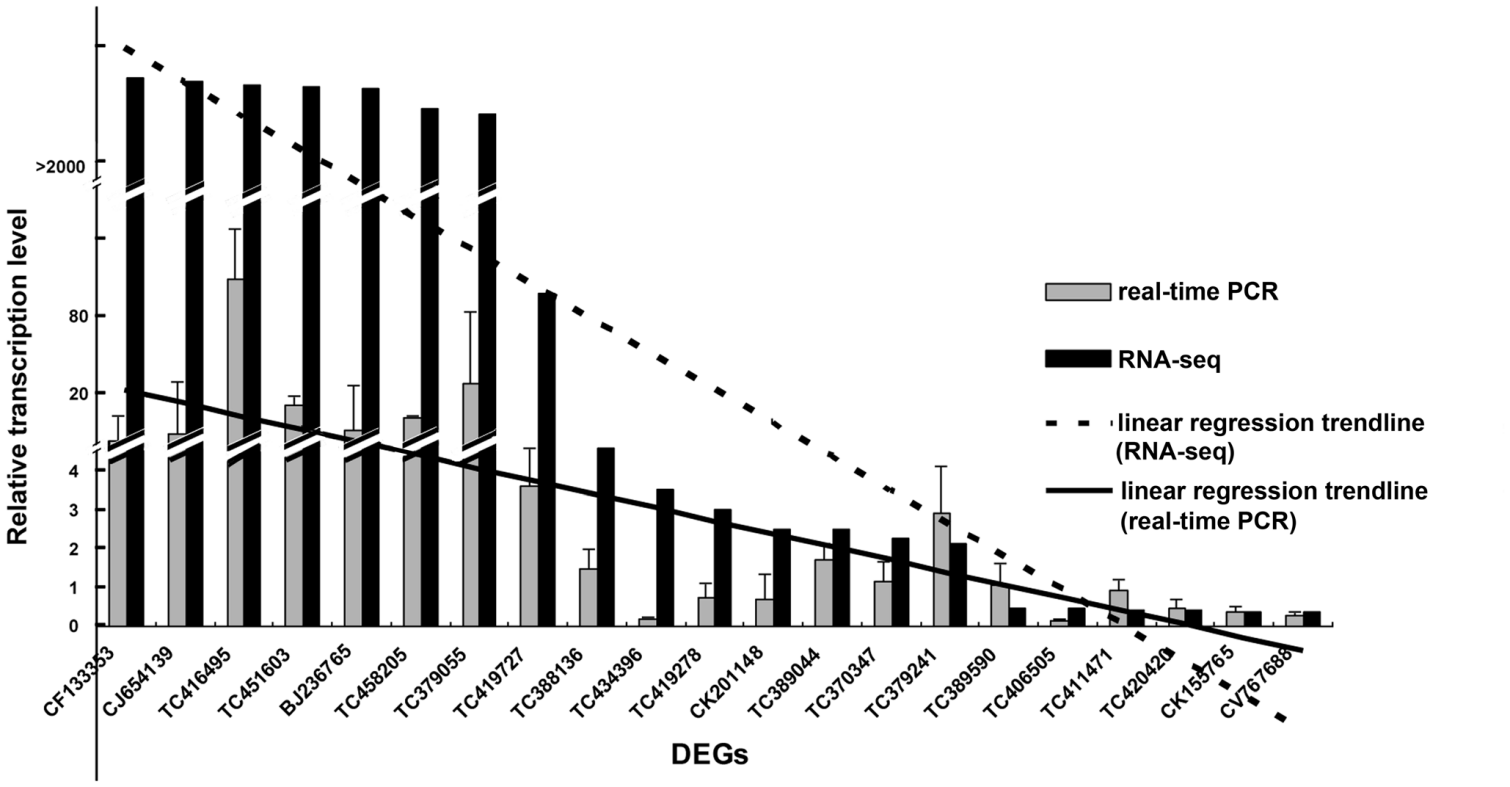
Expression level and trend line of certain DEGs according to RNA-seq and qRT-PCR. Assessment of the expression level of 21 DEGs, and contrasting of the results with RNA-seq. Ranging the DEGs in size of |log2|ratio plotted horizontal axis (from largest to smallest). The black bars mean the |log2|ratio, and the gray bars mean the 2^−ΔΔCT^ of the DEGs. And the gene annotations are as bellow. CF133353, expressed protein; CJ654139, Os07g0137900 protein; TC416495, MPI; TC451603, LOC496215 protein; BJ236765, unknown; TC458205, high molecular weight glutenin subunit; TC379055, unknown; TC419727, glutathione transferase; TC388136, lipoxygenase 1; TC434396, cinnamyl alcohol dehydrogenase; TC419278, alanine aminotransferase 2, CK201148, chitinase 2; TC389044, peroxidase 8; TC370347, sucrose synthase type I; TC379241, pyruvate decarboxylase isozyme 2; TC389590, thylakoid-bound ascorbate peroxidase; TC406505, phosphoglucomutase; TC411471, ATP synthase subunit a; TC420420, proteasome subunit alpha type-5; CK155765, adenine phosphoribosyltransferase 1; CV767688, triosephosphate isomerase.

To verify the DEG and DEP data, we used qRT-PCR to analyze the expression levels of 21 genes including 14 found in both DEGs and DEPs, and 7 other DEGs (|log2|ratio (infected/control)>10) (File S10). The results showed that 11 genes were up-regulated, and 3 genes were down-regulated in the infected callus compared with the non-infected callus, while 7 genes had no changes in expression levels (Figure 5). Although the qRT-PCR data did not match the RNA-seq data perfectly, some genes did show consistent expression patterns in both datasets. For examples, CF133353 (expressed protein), CJ654139 (Os07g0137900 protein), TC416495 (MPI), TC451603 (LOC496215 protein), BJ236765 (unknown), TC458205 (high molecular weight glutenin subunit) and TC379055 (unknown) got vastly different expression both from qRT-PCR and RNA-seq. And the difference of expression from the two analysis both significantly reduced in TC419727 (glutathione transferase), TC388136 (lipoxygenase 1), TC434396 (cinnamyl alcohol dehydrogenase), TC419278 (alanine aminotransferase 2) and CK201148 (chitinase 2). At last, TC406505 (phosphoglucomutase). TC411471 (ATP synthase subunit a), TC420420 (proteasome subunit alpha type-5), CK155765 (adenine phosphoribosyltransferase 1) and CV767688 (triosephosphate isomerase) were down regulated.

## Discussion

### Transferring process of T-DNA from *Agrobacterium* cells into wheat genome

*Agrobacterium tumefacien*s is a kind of pathogenic bacteria that causes crown gall disease (the formation of tumours) by the insertion of a T-DNA from a plasmid into plant cells in over 140 species of dicots under natural conditions. Unlike some tumor-inducing viruses, *Agrobacterium* T-DNA insertion into a host genome is a semi-random process [43] that causes an antibacterial response in the host. Up to date, even though many strains of *Agrobacterium* can be used in plant genetic transformation for T-DNA delivery, each strain has its suitable species to infect on. In this study, C58C1 strain was chosen because it was successfully used in many reports on wheat transformation [24]. According to some published papers [24], [26], [27], wheat transformation process mediated by *Agrobacterium* was finished within 48 hours. Especially, the growth peak of *Agrobacterium* on the surface of the host cells was observed when the co-culture period of *Agrobacterium* and wheat cells was proceeded for 36 h (File S11), and the expression of T-DNA was very intense after co-culture for 36 h [44]. Therefore, we expect that all of the transformation steps (attraction of *Agrobacteriu*m, T-DNA transportation and interaction) were lancing within this time since the infection. Therefore, in this investigation we chose the wheat immature embryos infected with *Agrobacterium* for 36 hours as materials for RNA-seq and proteomics analysis.

Investigating the host response to *Agrobacterium* infection process will contribute to understanding the interaction process and find some valuable clues on developing or optimizing of *Agrobacterium*- mediated transformation process. In our present study, 4,889 DEGs and 90 DEPs were identified to be closely related to *Agrobacterium* infection and all the DEGs involved in 111 pathways. Actually, RNA-seq is much more sensitive than 2D proteomics analysis, but the number of DEPs is much fewer than the DEGs. This kind of inconsistence is partly due to translational/post-translational regulation, but the most significant reason is that a DEP does not correspond to only one DEG (Table 5). In the response process of wheat cells to *Agrobacterium*, the genes related to secondary metabolites metabolic played the most important roles according to the results from pathway analysis and gene ontology analysis for biological process (Table 3, File S8). On the contrary, minimum of genes relate to photosynthesis pathway were detected. It indicated that the photosynthesis related genes avoided participating in the process of the transformation.

### Potential roles of related metabolism process proteins or secondary metabolites in *Agrobacterium* mediated approach

A large portion of the DEGs and DEPs in our datasets were found to be involved in the metabolism process. Some of them may play important roles in the interaction between wheat cells and *Agrobacterium*. Among them, sucrose synthase is an attractive functional protein. This carbohydrate was proved to participate both in sucrose synthesis and cleavage in plants, and catalyzes the chemical reaction of UDP-glucose+D-fructose←→UDP+sucrose [45].In this study, we found that sucrose synthase-2 was up-regulated according to RNA-seq and qRT-PCR, but was down-regulated according to proteomics dataset. The reason might be that the protein is degraded dramatically or transformed into other homologous type very soon although the transcription is activated. The up-regulation of this synthase at the level of transcription was also found in *Arabidopsis thaliana* under the same situation [46]. As sucrose synthase is beneficial to root nodule organogenesis in legumes [47], this corresponding gene might be related to the process of *Agrobacterium*-mediated genetic transformation. Furthermore, UDP-glucose is the substrate of UDP-glycosyltransferase, which was confirmed to participate in the response to pathogens [48]. In addition, UDP-glycosyltransferase has also been found to detoxify deoxynivalenol in *Fusarium* [49]. Recently, an *Arabidopsis* hat mutant over-expressing a UDP- glucosyltransferase gene was found to be resistant to *Agrobacterium*-mediated transformation, in which many defense genes were down-regulated [10]. And in our results, we also found that both sucrose synthase and UDP-glucosyltransferase were up-regulated at the level of transcription after infection by *Agrobacterium*. It is implied that saccharo metabolism might affect the infection process.

Some DEPs were involved in proteasome, such as proteasome subunit alpha type-3-like. proteasomes, played a straightforward and critical role in the process of plant immune system [50]. 26S proteasome and ubiquitin emerge as a key regulatory mechanism in selective protein degradation [51]. This pathway was involved in a wide variety of cellular processes in plants, such as hormone signaling, photomorphogenesis, flower development, embryo development, and defense response [52]. Meanwhile, ubiquitin-protein ligase (CA714086) was identified in our RNA-seq datasets and remarkably up-regulated (|log2|ratio = 4.080610714). The activation of ubiquitin is a typical reaction during the process of pathogen infection [53]. In addition, E3 ubiquitin ligase is required for cell death and defense response in plants [54]. Therefore, ubiquitin-protein ligase might be also related to the *Agrobacterium* mediated DNA delivery. On the other hand, 26S proteasome subunit was proved to be involved in innate immunity in *Arabidopsis* [55], In plant, selective removal of short-lived regulatory proteins is a very important controlling strategy for physiology, growth, and development [56]. However, in our study, proteasome maturation factor (TC379459) is down-regulated according to RNA-seq (|log2|ratio = −1.44004434). In wheat cells, some regulatory proteins might produce a favorable environment for *Agrobacterium* infection. Thus, to defend the resistance from plant cells, *Agrobacterium* might suppress the proteasomes from plant. Function of above candidate genes screened from this study in wheat *Agrobacterium*-mediated transformation needs to be further investigated.

### Relationship of plant phenylpropanoid biosynthesis and *Agrobacterium* infection

To our knowledge, UDP-glycosyltransferase mediates the transfer of glycosyl residues from nucleotide sugars to acceptor molecules (aglycones), such as plant secondary metabolites [57]. Most plant secondary metabolites might play important roles during *Agrobacterium* infection process. For examples, plant phenolics such as acetosyringone is an essential inducer for *Agrobacterium* infection. Acetosyringone is widely used in the protocol of *Agrobacterium*-mediated plant transformation. Other phenolics such as protocatechuic acid, β-resorcylic acid and protocatechuate also launch into the *Agrobacterium*-mediated transformation [58]. In our database, phenylalanine ammonia-lyase (PAL) was found up-regulated dramatically (|log2|ratio = 12.50423728). PAL catalyzes the first step in the biosynthesis of phenylpropanoids, which are further modified into a wide variety of phenolic compounds [59]. Another important secondary metabolites is flavonoid which involved in several biological process for plant development and defense [60]. In our research, two unknown proteins (TC413199, TC430821) were found to be involved in flavonoid biosynthetic process according to the GO analysis. Flavonoid is also a class of plant antibiotics. Sakuranetin, as a member of flavonoid, is recently demonstrated to have anti-inflammatory, anti-mutagenic, and anti-pathogenic activities.

### Expression of stress response related proteins during the interaction between plant and *Agrobacterium*

Based on the gene ontology analysis of the RNA-seq dataset, we found 9.68% DEGs were involved in the immunity process. According to the pathway analysis, 111 DEGs were found to be related to the plant-pathogen interaction pathway. In consideration of *Agrobacterium* being a kind of plant-pathogen in nature, the stress and pathogen response genes should be the focuses of the transformation process.

Most of the DEGs are involved in responses to reactive oxygen species (ROS) stresses. As we know, oxidative burst is the first defense of plants against pathogen attacks [61]. The ROS, stimulated by stress from pathogen attack and generated from both plant and pathogen [62], plays a key role in the crosstalk between biotic and abiotic stress signaling [63]. Plants generated ROS by activating various oxidases and peroxidases [64]. In the meanwhile, it was found in wheat that *Agrobacterium* infection induces plant cell to produce hydrogen peroxide (H_2_O_2_) rapidly and leads wheat cell death severally [65]. In plant, a series of peroxidases can eliminate ROS, such as catalase, which activity was confirmed to be closely related to efficient regeneration potential of wheat immature embryos during the somatic embryogenesis [66]. There are 3 kinds of peroxidases (peroxidase 8 (TC389044), phospholipid hydroperoxide glutathione peroxidase (CD908771 and CA729147) and ascorbate peroxidase (TC389590)) found in our datasets. In the datasets of RNA-seq and proteomics, peroxidase 8 was up-regulated (|log2|ratio = 1.31539) during the infection process while ascorbate peroxidase was down-regulated (|log2|ratio = −1.05259). Furthermore, according to the results of qRT-PCR, the expression level of peroxidase 8 was up-regulated 1.7 times but the expression level of ascorbate peroxidase almost had no change (File S10). By coincidence, peroxidase was also identified in *Ageratum conyzoides* and *Arabidopsis thaliana* responding to *Agrobacterium tumefaciens* infection [16], [67],. Peroxidase has the function of interrupting the cascades of uncontrolled oxidation [68]. Peroxidase 8 is a kind of peroxidase belonging to Class-III in *Triticum monococcum*, which was a component of defense system responding to powdery mildew attack [46]. Ascorbate peroxidase scavenges hydrogen peroxide in plants, and is essential to protect cell constituents from lesion by hydrogen peroxide and other hydroxyl radicals produced from the interaction process of plant cells and pathogens [67], [69], Hydrogen peroxide is a kind of inhibitor for the invading pathogens, but it also contributes some virulence to pathogens [61]. Thereby, hydrogen peroxide might be an advantageous compound for both host cells and *Agrobacterium*, and it might be necessary to repress the accumulation of hydrogen peroxide-scavenging enzyme such as ascorbate peroxidase in the infection process.

Phospholipid hydroperoxide glutathione peroxidase was determined to be related to the metabolism of glutathione, which is an effective antioxidant preventing damage of important cellular components caused by ROS [70]. Moreover, phospholipid hydroperoxide glutathione peroxidase is a monomer, and the donor substrate of this peroxidase is not only restricted to glutathione (GSH) but also binds to specific mitochondrial proteins. In present research, phospholipid hydroperoxide glutathione peroxidase was detected to be up-regulated according to RNA-seq but down-regulated according to proteomics in the infection process. It is possible that phospholipid hydroperoxide glutathione peroxidase binds on mitochondrial proteins dramatically when the activity of mitochondrial is elevated. It makes protein of phospholipid hydroperoxide glutathione peroxidase decrease although the gene's expression was activated.

The rest of DEPs identified are related to be biotic stress response, such as chitinase (CK201148) and thioredoxin h (TC396636). As chitin is an important component of the cell wall of fungi and chitinases are generally found in organisms that dissolve and digest the chitin of fungi [71] plant chitinases are thought to be related to pathogen resistance [72]. Chitinase was up-regulated in our RNA-seq datasets and there was almost no diversity according to qRT-PCR result (2^−ΔΔCT^ = 0.982055), but was down-regulated in proteomics datasets. Moreover, by reducing the defense of host cells, chitinases enable symbiotic interaction with nitrogen-fixing bacteria or mycorrhizal fungi [73]. To remove the barriers of infection, *Agrobacterium* suppressed the accumulation of chitinase from plant cells although the gene's expression was already activated. hioredoxin h also has potential capability against pathogens attaching, and evidence showed *thioedoxin h* gene was strongly induced within 4 hours in *Arabidopsis* cell suspensions treated with fungal elicitors, which contained wide range stress inducing agent [74]. In our study, we found that thioredoxin h was down-regulated both in RNA-seq and proteomics datasets which means the immune response of the host was impaired during the infection process.

### Relationship of T-DNA integration and host proteins related to nucleic acid binding and nucleotide excision repair

The ultimate aim of *Agrobacterium* transformation is to import the T-DNA into plant genome. So, several nucleic acid binding proteins should take part in the two steps: T-DNA nuclear import and integration. And the typically nucleic acid binding proteins are thought to be the T-complex ones from *Agrobacterium* [75]. Now some host proteins have shown to be important in the last two steps for T-DNA delivery [7]. In this study the nucleic acid binding proteins take a very large part of the DEGs based on the GO analysis. The pathway analysis indicated that the spliceosome proteins should be paid more attention. T-DNA integration into plant chromosome actually belongs to the way of non-homologous (illegitimate) recombination (NHR), even when the T-DNA shares high homology with the host genome. As for the pattern of the T-DNA integration, the double-strand-break repair (DSBR) model and single-strand-gap repair (SSGR) model were originally proposed [76]. Above findings suggest that nucleotide excision repair proteins are the key players in the process of T-DNA integration.

Particularly, histone was demonstrated to play an important role in the transformation process mediated by *Agrobacterium* [7]. Especially, histone H2A, histone H4, and histone H3-11 in *Arabidopsis* can increase transformation susceptibility. Other plant proteins related to the transformation process include BTIs (VirB2-interacting proteins), AtRAB8, and DIG3. Hwang *et al.* used the C-terminal-processed portion of VirB2 as the bait to search the interaction protein by yeast two-hybrid in *Arabidopsis*, and found that BTI1, BTI2, BTI3, and a membrane-associated GTPase, AtRAB8 interact with VirB2. Their further study showed the positive meaning of these proteins in the infection process of *Agrobacterium* [8]. DIG3, found in tomato, encodes an enzymatically active type 2C serine/threonine protein phosphatase, which interacts with VirD2. Over-expression of DIG3 in tobacco protoplasts inhibited nuclear import of VirD2 nuclear localization [9]. In our study, Histone and GTPase related protein genes were also identified, such as TC396751 encoding Histone H2B. In the process of *Agrobacterium* infection, H2B was down-regulated dramatically (|log2|ratio = −13.62721073). In the previous research [13], H2B did not lead to increased transformation susceptibility. However, according to our results, we assumed that H2B might have a negative effect during the transformation process. Therefore, the expression level of H2B might be depressed by *Agrobacterium*. Besides, we also obtained several serine/threonine-protein kinases such as TC440175 (Serine/threonine-protein kinase Nek5, (|log2|ratio = 2.297455487)). Some of them probably are similar to DIG3, and interact with VirD2. Beyond that, a big part of the DEGs have the function of nucleic acid binding and protein-protein interaction based on the categorization of the GO terms (Figure 1B). It is suggested that some genes among the DEGs should play roles in the nucleic importing and integration into genome of T-DNA.

## Conclusions

In this study, we identified a set of 4988 DEGs and 90 DEPs in *Agrobacterium*-infected wheat tissues. After comparative analysis, 24 of the 90 DEPs were detected in RNA-seq and proteomics datasets simultaneously. The expressions of the most DEGs were found to be uniformly up/down-regulated between RNA-seq and qRT-PCR datasets, which proved the authenticity of the results from RNA-seq. According to GO analysis, we found that a big part of these differentially expressed genes were related to the process of stress or immunity response, and other major part of DEGs involved in the process of molecular modification. We believe that some of these genes are closely related to the transformation process mediated by *Agrobacterium*. The findings achieved in this study will help to further exploit the interaction between *Agrobacterium* and host cells, and may facilitate the development of efficient plant transformation strategies.

## Supporting Information

File S1 **Categorization of row reads of the control material A and the infected material B.**
(DOC)

File S2 **Alignment statistics of total reads.**
(DOC)

File S3 **Randomness assessment A and sequencing saturation analysis B.**
(DOC)

File S4 **Gene coverage statistics.**
(DOC)

File S5 **The differentially expressed genes (|log2|ratio (infected/control)≥1).**
(XLS)

File S6 **Gene Ontology analysis information for cellular component.**
(DOC)

File S7 **Gene Ontology analysis information for molecular function.**
(DOC)

File S8 **Gene Ontology analysis information for biological process.**
(DOC)

File S9 **Distribution of all GEGs on the pathways.**
(DOC)

File S10 **Comparison of expression level between the data of real-time PCR and RNA-seq.**
(DOC)

File S11 **Detection of the** ***Agrobacterium*** **attachment on wheat callus by scanning electron microscope (SEM).** The adsorption of *Agrobacterium* to wheat callus after co-culture for 30 minutes, 12 hours, 24 hours, 36 hours, and 48 hours.
(TIF)
