# Supplementary material for: Global Analysis of Differentially Expressed Genes and Proteins in the Wheat Callus Infected by Agrobacterium tumefaciens
Source: PLoS One. 2013 Nov 20;8(11):e79390. doi: 10.1371/journal.pone.0079390 (PMC3835833; doi:10.1371/journal.pone.0079390)
Supplement: File S1 — Categorization of row reads of the control material A and the infected material B. (DOC) [file pone.0079390.s001.doc]

**File S1**  **Categorization of row reads of the control material A and the infected material B**


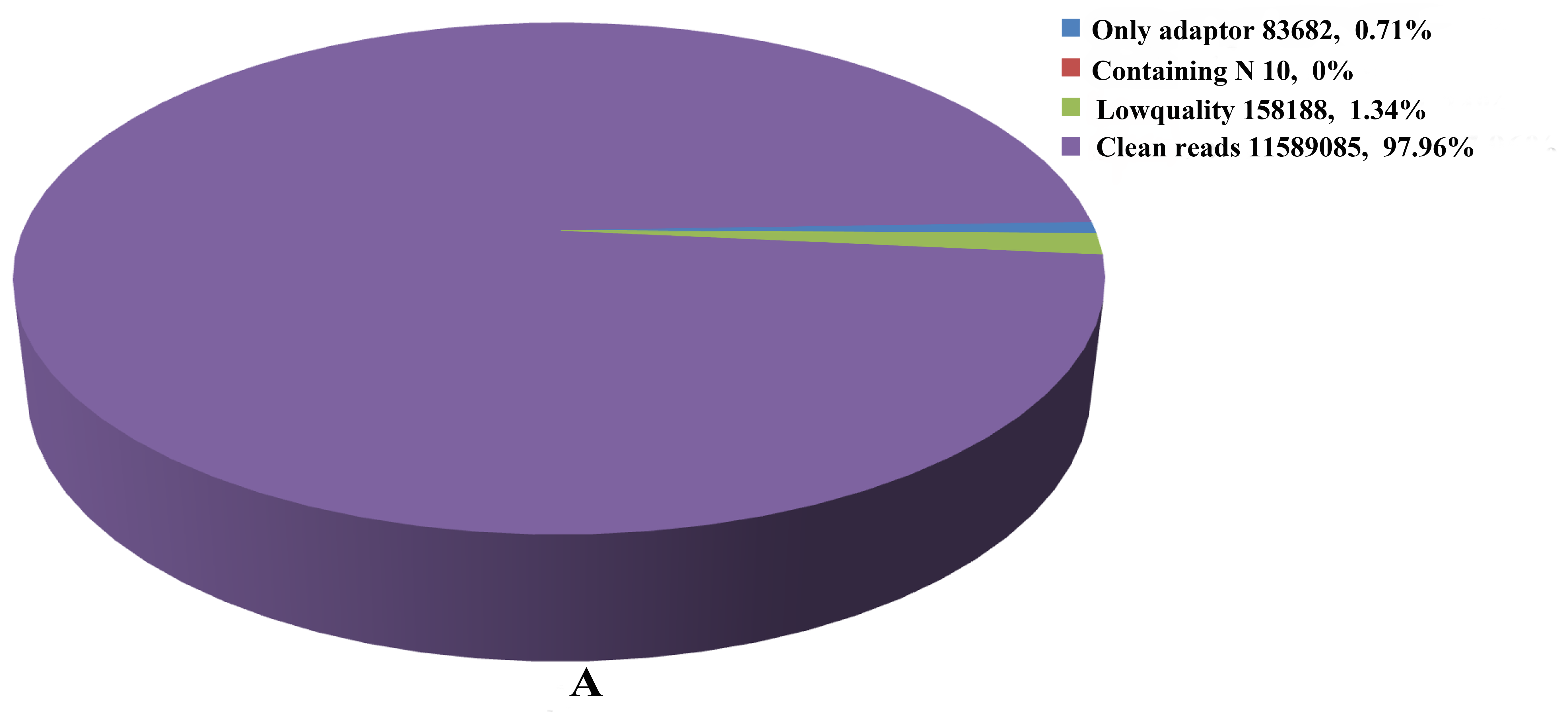


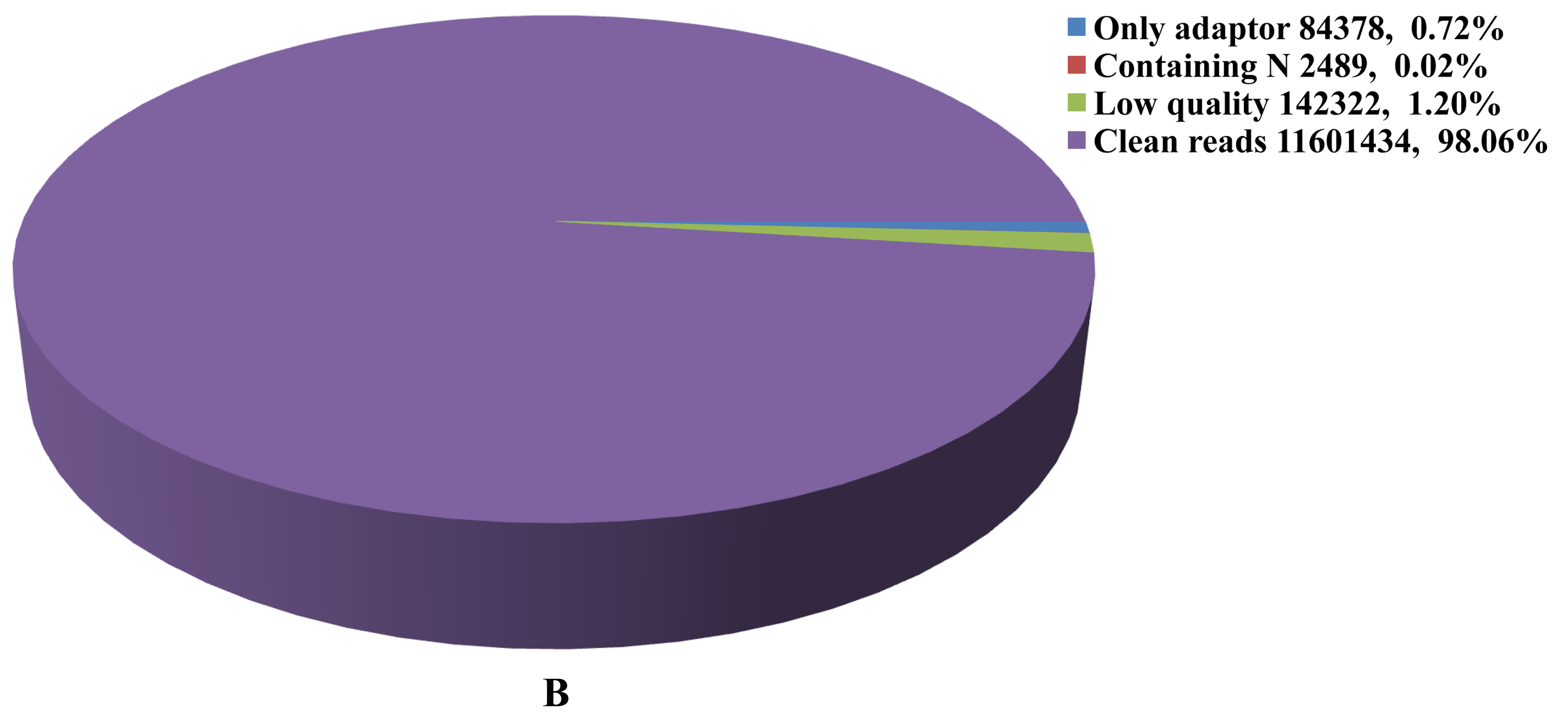


“Only adaptor” means the reads with adaptor only, “Containing N” the reads with unknown nucleic acid, “Low quality” the the reads of low quality, and “Clean reads” the reads without mistake. Each category is labeled with the percentage of total RNA-seq reads.
