# Supplementary material for: Global Analysis of Differentially Expressed Genes and Proteins in the Wheat Callus Infected by Agrobacterium tumefaciens
Source: PLoS One. 2013 Nov 20;8(11):e79390. doi: 10.1371/journal.pone.0079390 (PMC3835833; doi:10.1371/journal.pone.0079390)
Supplement: File S2 — Alignment statistics of total reads. (DOC) [file pone.0079390.s002.doc]

**File S2 Alignment statistics** of total reads

| **Items** | **Control** | | **Infected** | |
| --- | --- | --- | --- | --- |
| **Map to gene** | **Reads number** | **Percentage (%)** | **Reads number** | **Percentage (%)** |
| Total reads | 11601434 | 100.00 | 11589085 | 100.00 |
| Total base pairs | 568470266 | 100.00 | 567865165 | 100.00 |
| Total mapped reads | 9902472 | 85.36 | 9476561 | 81.77 |
| Perfect match | 8092153 | 69.75 | 7636666 | 65.90 |
| ≤2bp mismatch | 1810319 | 15.60 | 1839895 | 15.88 |
| Unique match | 3554645 | 30.64 | 3519045 | 30.37 |
| Multi-position match | 6347827 | 54.72 | 595716 | 51.41 |
| Total unmapped reads | 1698962 | 14.64 | 2112524 | 18.23 |
