# Supplementary material for: Global Analysis of Differentially Expressed Genes and Proteins in the Wheat Callus Infected by Agrobacterium tumefaciens
Source: PLoS One. 2013 Nov 20;8(11):e79390. doi: 10.1371/journal.pone.0079390 (PMC3835833; doi:10.1371/journal.pone.0079390)
Supplement: File S3 — Randomness assessment A and sequencing saturation analysis B. (DOC) [file pone.0079390.s003.doc]

**File S3** **Randomness assessment A and sequencing saturation analysis B**


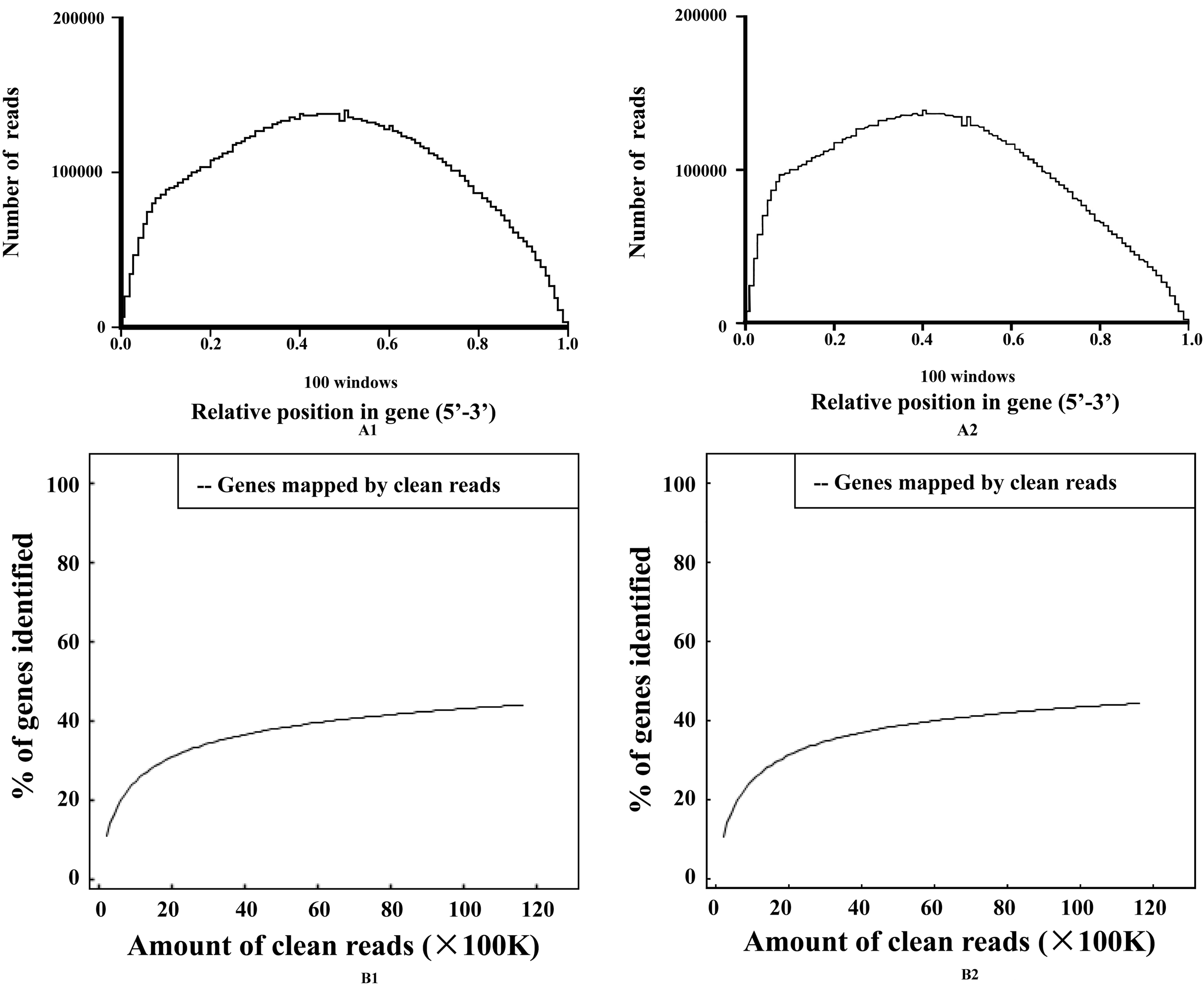


Randomness assessment is the distribution of reads locating on the genes. If the randomness is good, the reads in every position would be evenly distributed. Sequence saturation analysis is used to measure the sequencing data of a sample. With the number of reads increasing, the number of detected genes is increasing. When the number of reads reaches certain value, the growth rate of detected genes flattens. A1 and B1 are from no-infected sample, A2 and B2 are from infected sample.
