# Supplementary material for: Global Analysis of Differentially Expressed Genes and Proteins in the Wheat Callus Infected by Agrobacterium tumefaciens
Source: PLoS One. 2013 Nov 20;8(11):e79390. doi: 10.1371/journal.pone.0079390 (PMC3835833; doi:10.1371/journal.pone.0079390)
Supplement: File S4 — Gene coverage statistics. (DOC) [file pone.0079390.s004.doc]

**File S4 Gene coverage statistics**


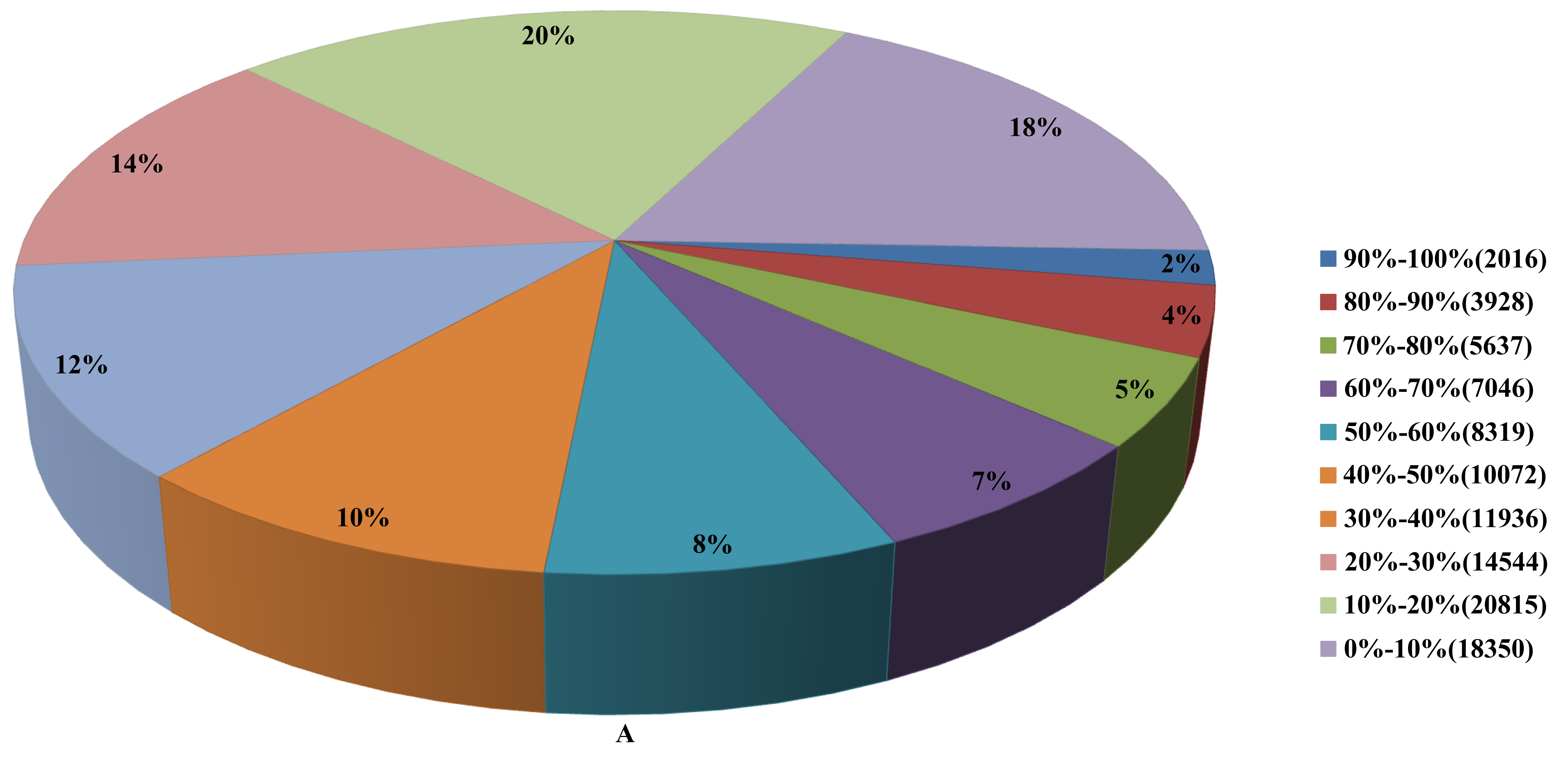


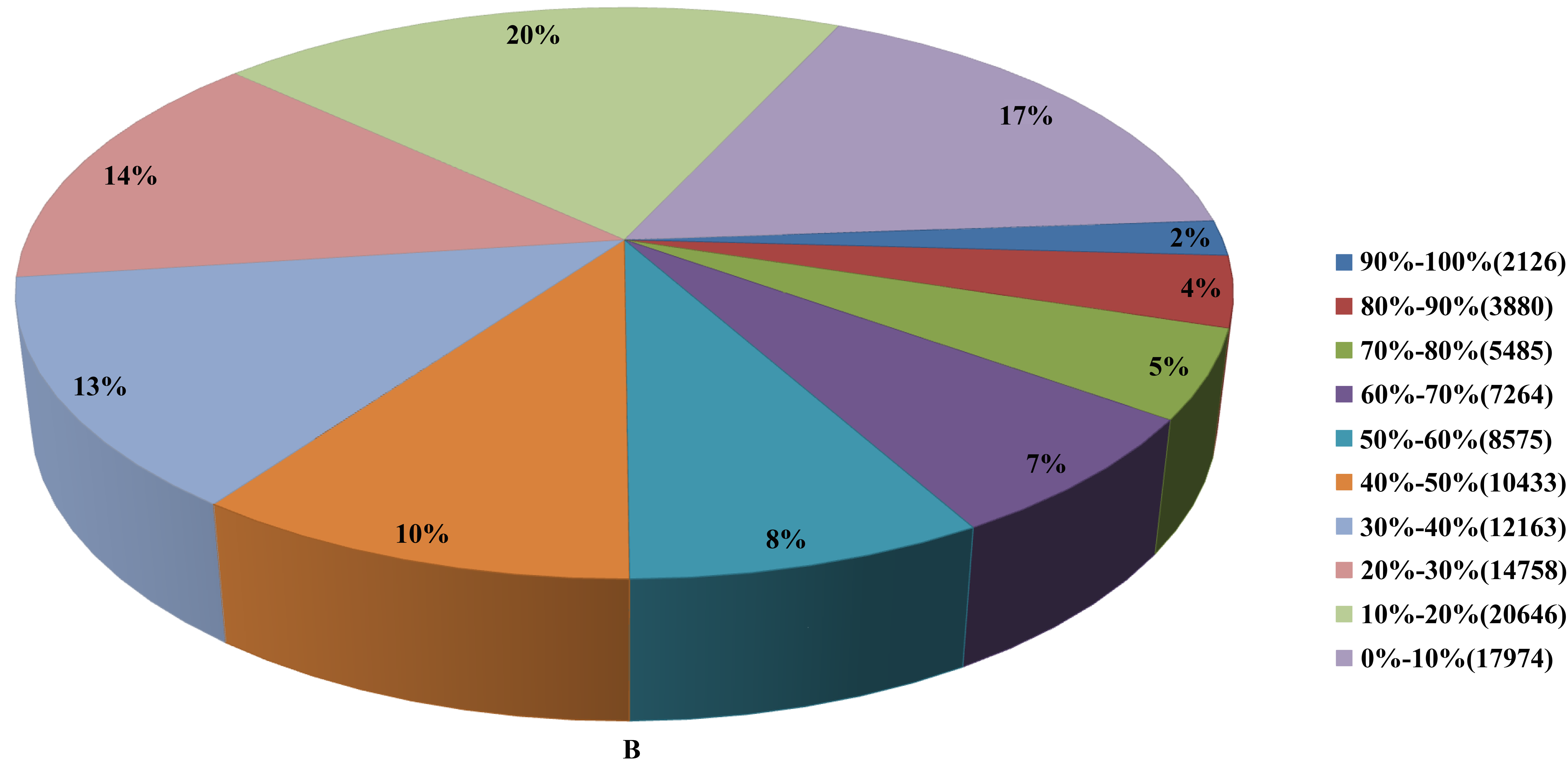


This coverage is equal to the ratio of the base pair number in a gene covered by unique mapping reads to the total base pairs number of the gene. The genes with different coverage are labeled with different colors. Each coverage category is labeled with the percentage of total tested genes.
