# Supplementary material for: Global Analysis of Differentially Expressed Genes and Proteins in the Wheat Callus Infected by Agrobacterium tumefaciens
Source: PLoS One. 2013 Nov 20;8(11):e79390. doi: 10.1371/journal.pone.0079390 (PMC3835833; doi:10.1371/journal.pone.0079390)
Supplement: File S6 — Gene Ontology analysis information for cellular component. (DOC) [file pone.0079390.s006.doc]

| **Gene ontology term**  **File S6 Gene Ontology analysis information for cellular component** | **Genes annotated to the term** |
| --- | --- |
| [Nuclear chromatin](http://amigo.geneontology.org/cgi-bin/amigo/go.cgi?action=query&view=query&query=GO:0000790&search_constraint=terms) | TC392303, TC410352, TC390393, TC416529, TC397258, TC396230, TC435533, TC416069, BQ838511, TC374240, CA710880, TC424204, TC387579, TC416154, TC423252, TC397660, TC393814, TC389363, TC388914, TC416493, TC458562, TC446235, TC398538, TC410063, TC403885, TC420735, TC404371, TC395453, TC386961, TC384219, TC410194, TC390569, TC416685, TC419057, TC403872, TC389090, TC412483, TC389375, TC382655, CK200433, TC398714, BQ294582, TC406038, CJ550278, TC395351, TC401915, TC418032, TC397444, CK210754, TC417012, CA632212, DR739994, TC384454, TC411287, TC394916, TC393904, TC390994, TC406236, TC378274, TC391130, CK217367, TC446038, TC379853, CK214702, TC400755, TC403986, TC395723, TC405440, CK213497, TC385365, TC401210, DR737360, TC379357, TC377225, TC386279, TC411684, TC381943, TC412520, TC380662, TC413392, BQ607161, TC389162, DR739471, TC402534, TC434820, TC443387, TC379711, TC415083, TC379903, TC423265, TC392272, TC401244, TC406584, TC386313, CV759879, TC408907, TC402440, TC391438, TC417992, TC394307, TC375146, TC386963, TC389589, TC412732, TC452762, TC405695, TC392778, TC397312, TC388403, TC386422, TC416695, TC388688, TC406469, TC388822, TC410690, TC377496, TC406708, TC429771, TC423576, TC402186, TC416658, TC405356, TC393561, TC393692, TC391671, TC393554, TC407614, TC402603, TC441241, TC379436, TC413043, CA729339, CK214224, TC408299, TC376263, TC379635, TC416169, TC433589, TC377410, TC381218, TC389816, TC392323, TC413066, CA613620, CK211469, CB307332, TC404842, TC397729, TC403803, TC399342, TC396895, TC434831, TC426326, TC382342, TC381462, TC393106, TC401758, TC392263, TC396460, TC389661, TC428944 |
| [Nuclear nucleosome](http://amigo.geneontology.org/cgi-bin/amigo/go.cgi?action=query&view=query&query=GO:0000788&search_constraint=terms) | TC392303, TC410352, TC390393, TC416529, TC397258, TC396230, TC435533, TC416069, BQ838511, CA710880, TC424204, TC387579, TC416154, TC423252, TC397660, TC393814, TC389363, TC388914, TC416493, TC458562, TC446235, TC398538, TC410063, TC403885, TC420735, TC404371, TC395453, TC386961, TC384219, TC410194, TC390569, TC416685, TC419057, TC403872, TC389090, TC412483, TC389375, TC382655, CK200433, TC398714, BQ294582, TC406038, TC395351, TC401915, TC397444, TC418032, CK210754, TC417012, CA632212, DR739994, TC384454, TC411287, TC394916, TC393904, TC390994, TC406236, TC378274, TC391130, CK217367, TC446038, TC379853, CK214702, TC400755, TC403986, TC405440, CK213497, TC385365, TC401210, DR737360, TC379357, TC377225, TC386279, TC411684, TC381943, TC412520, TC380662, TC413392, BQ607161, DR739471, TC402534, TC434820, TC443387, TC379711, TC415083, TC379903, TC423265, TC392272, TC401244, TC406584, TC386313, CV759879, TC408907, TC402440, TC391438, TC417992, TC394307, TC375146, TC386963, TC389589, TC412732, TC452762, TC405695, TC392778, TC397312, TC388403, TC386422, TC416695, TC388688, TC406469, TC388822, TC410690, TC377496, TC406708, TC429771, TC423576, TC402186, TC416658, TC405356, TC393561, TC393692, TC391671, TC393554, TC407614, TC402603, TC441241, TC379436, TC413043, CA729339, CK214224, TC408299, TC376263, TC379635, TC416169, TC433589, TC377410, TC381218, TC389816, TC392323, TC413066, CA613620, CK211469, CB307332, TC404842, TC397729, TC403803, TC399342, TC396895, TC434831, TC426326, TC382342, TC381462, TC393106, TC401758, TC392263, TC396460, TC389661, TC428944 |
| [Nuclear chromosome](http://amigo.geneontology.org/cgi-bin/amigo/go.cgi?action=query&view=query&query=GO:0000228&search_constraint=terms) | TC392303, TC410352, TC390393, TC416529, TC397258, TC396230, TC435533, TC416069, BQ838511, TC374240, CA710880, TC424204, TC387579, TC416154, TC423252, TC397660, TC393814, TC389363, TC388914, TC416493, TC458562, TC446235, TC398538, TC410063, TC403885, TC420735, TC404371, TC395453, TC386961, TC384219, TC410194, TC390569, TC416685, TC419057, TC403872, TC389090, TC412483, TC389375, TC382655, CK200433, TC398714, BQ294582, TC406038, CJ550278, TC395351, TC401915, TC418032, TC397444, CK210754, TC417012, CA632212, DR739994, TC384454, TC411287, TC394916, TC393904, TC390994, TC406236, TC378274, TC391130, CK217367, TC446038, TC379853, CK214702, TC400755, TC403986, TC395723, TC405440, CK213497, TC385365, TC401210, DR737360, TC379357, TC377225, TC386279, TC411684, TC381943, TC412520, TC380662, TC413392, BQ607161, TC389162, DR739471, TC402534, TC434820, TC443387, TC379711, TC415083, TC379903, TC423265, TC392272, TC401244, TC406584, TC386313, CV759879, TC408907, TC402440, TC391438, TC417992, TC394307, TC375146, TC386963, TC389589, TC412732, TC452762, TC405695, TC392778, TC397312, TC388403, TC386422, TC416695, TC388688, TC406469, TC388822, TC410690, TC377496, TC406708, TC429771, TC423576, TC402186, TC416658, TC405356, TC393561, TC393692, TC391671, TC393554, TC407614, TC402603, TC441241, TC379436, TC413043, CA729339, CK214224, TC408299, TC394118, TC376263, TC379635, TC416169, TC433589, TC377410, TC381218, TC389816, TC392323, TC413066, CA613620, CK211469, CB307332, TC404842, TC397729, TC403803, TC399342, TC396895, TC434831, TC426326, TC382342, TC381462, TC393106, TC401758, TC392263, TC396460, TC389661, TC428944 |
| [Nuclear chromosome part](http://amigo.geneontology.org/cgi-bin/amigo/go.cgi?action=query&view=query&query=GO:0044454&search_constraint=terms) | TC392303, TC410352, TC390393, TC416529, TC397258, TC396230, TC435533, TC416069, BQ838511, TC374240, CA710880, TC424204, TC387579, TC416154, TC423252, TC397660, TC393814, TC389363, TC388914, TC416493, TC458562, TC446235, TC398538, TC410063, TC403885, TC420735, TC404371, TC395453, TC386961, TC384219, TC410194, TC390569, TC416685, TC419057, TC403872, TC389090, TC412483, TC389375, TC382655, CK200433, TC398714, BQ294582, TC406038, CJ550278, TC395351, TC401915, TC418032, TC397444, CK210754, TC417012, CA632212, DR739994, TC384454, TC411287, TC394916, TC393904, TC390994, TC406236, TC378274, TC391130, CK217367, TC446038, TC379853, CK214702, TC400755, TC403986, TC395723, TC405440, CK213497, TC385365, TC401210, DR737360, TC379357, TC377225, TC386279, TC411684, TC381943, TC412520, TC380662, TC413392, BQ607161, TC389162, DR739471, TC402534, TC434820, TC443387, TC379711, TC415083, TC379903, TC423265, TC392272, TC401244, TC406584, TC386313, CV759879, TC408907, TC402440, TC391438, TC417992, TC394307, TC375146, TC386963, TC389589, TC412732, TC452762, TC405695, TC392778, TC397312, TC388403, TC386422, TC416695, TC388688, TC406469, TC388822, TC410690, TC377496, TC406708, TC429771, TC423576, TC402186, TC416658, TC405356, TC393561, TC393692, TC391671, TC393554, TC407614, TC402603, TC441241, TC379436, TC413043, CA729339, CK214224, TC408299, TC376263, TC379635, TC416169, TC433589, TC377410, TC381218, TC389816, TC392323, TC413066, CA613620, CK211469, CB307332, TC404842, TC397729, TC403803, TC399342, TC396895, TC434831, TC426326, TC382342, TC381462, TC393106, TC401758, TC392263, TC396460, TC389661, TC428944 |
| [Nucleosome](http://amigo.geneontology.org/cgi-bin/amigo/go.cgi?action=query&view=query&query=GO:0000786&search_constraint=terms) | TC461921, TC410352, TC386040, TC390393, TC416529, TC397258, CV775873, TC396230, TC460760, TC427210, TC416069, BQ838511, TC398052, CA710880, TC400388, TC433162, TC394459, TC416154, TC384738, TC423252, TC397660, TC389363, TC388410, CK203550, TC416493, TC458562, TC406264, TC380063, TC398538, TC410063, TC378054, TC448471, TC395453, TC410194, TC392875, TC435546, TC390569, TC405540, TC419057, TC403872, TC425847, CJ727624, TC398714, BQ294582, CJ550278, TC413427, TC411128, TC401915, TC397444, TC398304, TC425878, TC393100, TC433957, TC417012, CA632212, DR739994, TC459193, TC394916, TC394206, TC394820, TC406236, TC418073, TC378274, TC391130, TC417308, CK217367, TC445767, TC446038, TC413460, TC379853, BQ609416, CK214702, TC449504, TC395723, TC405440, CK213497, CJ792862, TC389718, TC404052, TC377225, TC381943, TC412520, TC432001, TC413392, BQ607161, TC402308, TC434820, TC379711, TC400330, CA595837, TC379903, TC423265, TC392272, TC401244, CA730421, TC446465, TC386313, CV759879, TC408907, CA606693, TC391438, CK211707, TC386963, TC412732, TC405695, TC392778, TC396751, TC397312, TC405030, TC388403, TC376527, CA615187, TC416695, TC385780, TC406469, TC405784, TC399919, TC407572, TC372175, TC410690, TC406708, TC419747, TC410074, TC402186, CV763657, TC405356, TC402603, TC441241, TC413043, CA729339, CK214224, TC408299, BQ239045, CA709177, TC376263, TC416169, TC381218, TC408229, TC422425, CA613620, TC398805, CV781430, TC388520, TC411191, TC399342, TC434831, TC426326, TC381462, CD882425, TC373914, TC392263, TC392303, TC435533, TC425841, CF554444, TC424204, TC387579, TC406870, TC388914, TC393814, TC387621, TC398633, TC394716, TC446235, TC380943, TC393830, TC420735, TC403885, TC404371, TC384219, TC386961, TC416685, TC387344, TC412483, TC389090, TC389375, TC382655, CK200433, TC406038, TC395351, TC418032, CK210754, TC410147, TC384454, TC411287, TC386237, TC393904, TC390994, TC391143, TC381463, TC403157, TC386414, TC395303, TC400755, TC440636, TC403986, TC458987, TC404843, TC431879, CD876572, TC385365, TC401210, DR737360, TC379357, TC390135, TC397885, TC386279, TC411480, TC411684, TC380662, DR739471, TC402534, TC443387, TC415083, TC386535, GH729256, TC406584, TC386519, TC402440, TC417992, TC391411, TC394307, TC375146, TC389589, TC452762, TC386344, TC381963, TC383176, CD878039, TC386422, TC388688, TC388547, TC414606, TC388822, TC377496, TC388976, TC396365, TC429771, TC423576, TC386639, TC416658, TC393561, TC393692, TC391671, TC393554, TC407614, TC382928, TC379436, TC417363, TC403573, TC403929, TC413700, TC449043, TC379635, TC440321, TC433589, TC377410, TC389816, TC413066, TC392323, CB307332, CK211469, TC404842, TC397729, TC403803, TC396895, TC382342, TC393106, TC401758, TC397676, TC396460, TC432504, TC417067, TC389661, TC428944 |
| [Protein-DNA complex](http://amigo.geneontology.org/cgi-bin/amigo/go.cgi?action=query&view=query&query=GO:0032993&search_constraint=terms) | TC461921, TC410352, TC386040, TC390393, TC416529, TC397258, CV775873, TC396230, TC460760, TC427210, TC416069, BQ838511, TC398052, CA710880, TC400388, TC433162, TC394459, TC416154, TC384738, TC423252, TC397660, TC389363, TC388410, CK203550, TC416493, TC458562, TC406264, TC380063, TC398538, TC410063, TC378054, TC448471, TC395453, TC410194, TC392875, TC435546, TC390569, TC405540, TC419057, TC403872, TC425847, CJ727624, TC398714, BQ294582, CJ550278, TC413427, TC411128, TC401915, TC397444, TC398304, TC425878, TC393100, TC433957, TC417012, CA632212, DR739994, TC459193, TC394916, TC394206, TC394820, TC406236, TC418073, TC378274, TC391130, TC417308, CK217367, TC445767, TC446038, TC413460, TC379853, BQ609416, CK214702, TC449504, TC395723, TC405440, CK213497, CJ792862, TC389718, TC404052, TC377225, TC381943, TC412520, TC432001, TC413392, BQ607161, TC402308, TC434820, TC379711, TC400330, CA595837, TC379903, TC423265, TC392272, TC401244, CA730421, TC446465, TC386313, CV759879, TC408907, CA606693, TC391438, CK211707, TC386963, TC412732, TC405695, TC392778, TC396751, TC397312, TC405030, TC388403, TC376527, CA615187, TC416695, TC385780, TC406469, TC405784, TC399919, TC407572, TC372175, TC410690, TC406708, TC419747, TC410074, TC402186, CV763657, TC405356, TC402603, TC441241, TC413043, CA729339, CK214224, TC408299, BQ239045, CA709177, TC376263, TC416169, TC381218, TC408229, TC422425, CA613620, TC398805, CV781430, TC388520, TC411191, TC399342, TC434831, TC426326, TC381462, CD882425, TC373914, TC392263, TC392303, TC435533, TC425841, CF554444, TC424204, TC387579, TC406870, TC388914, TC393814, TC387621, TC398633, TC394716, TC446235, TC380943, TC393830, TC420735, TC403885, TC404371, TC384219, TC386961, TC416685, TC387344, TC412483, TC389090, TC389375, TC382655, CK200433, TC406038, TC395351, TC418032, CK210754, TC410147, TC384454, TC411287, TC386237, TC393904, TC390994, TC391143, TC381463, TC403157, TC386414, TC395303, TC400755, TC440636, TC403986, TC458987, TC404843, TC431879, CD876572, TC385365, TC401210, DR737360, TC379357, TC390135, TC397885, TC386279, TC411480, TC411684, TC380662, DR739471, TC402534, TC443387, TC415083, TC386535, GH729256, TC406584, TC386519, TC402440, TC417992, TC391411, TC394307, TC375146, TC389589, TC452762, TC386344, TC381963, TC383176, CD878039, TC386422, TC388688, TC388547, TC414606, TC388822, TC377496, TC388976, TC396365, TC429771, TC423576, TC386639, TC416658, TC393561, TC393692, TC391671, TC393554, TC407614, TC382928, TC379436, TC417363, TC403573, TC403929, TC413700, TC449043, TC379635, TC440321, TC433589, TC377410, TC389816, TC413066, TC392323, CB307332, CK211469, TC404842, TC397729, TC403803, TC396895, TC382342, TC393106, TC401758, TC397676, TC396460, TC432504, TC417067, TC389661, TC428944 |
| [Chromosomal part](http://amigo.geneontology.org/cgi-bin/amigo/go.cgi?action=query&view=query&query=GO:0044427&search_constraint=terms) | TC461921, TC410352, TC386040, TC390393, TC416529, TC397258, CV775873, TC396230, TC460760, TC427210, TC416069, BQ838511, TC398052, CA710880, TC400388, TC433162, TC394459, TC416154, TC384738, TC423252, TC397660, TC389363, TC388410, CK203550, TC416493, TC458562, TC406264, TC380063, TC398538, TC410063, TC378054, TC448471, TC395453, TC410194, TC392875, TC435546, TC384735, TC390569, TC405540, TC419057, TC403872, TC425847, CJ727624, TC398714, BQ294582, CJ550278, TC413427, TC411128, TC401915, TC397444, TC398304, TC425878, TC393100, TC433957, TC417012, CA632212, DR739994, TC459193, TC394916, TC394206, TC394820, TC406236, TC418073, TC378274, TC391130, TC417308, CK217367, TC445767, TC446038, TC413460, TC379853, TC370315, BQ609416, CK214702, TC449504, TC395723, TC405440, CK213497, CJ792862, TC389718, TC404052, TC377225, TC381943, TC412520, TC432001, TC413392, BQ607161, TC402308, TC434820, TC441343, TC379711, TC400330, CA595837, TC379903, TC423265, TC392272, TC377061, TC401244, CA730421, TC446465, TC386313, CV759879, TC408907, CA606693, TC391438, CK211707, TC386963, TC412732, TC405695, TC392778, TC396751, TC397312, TC405030, CK211589, TC388403, TC376527, CA615187, TC416695, TC385780, TC406469, TC405784, TC399919, TC407572, TC372175, TC410690, TC406708, TC419747, TC410074, TC402186, CV763657, TC405356, TC402603, TC441241, TC413571, TC413043, CA729339, CK214224, TC408299, BQ239045, CA709177, TC376263, TC416169, TC381218, TC408229, TC422425, CA613620, TC398805, TC369092, CV781430, TC388520, TC411191, TC399342, TC434831, TC426326, TC381462, CD882425, TC373914, TC392263, TC392303, TC394796, TC435533, TC374240, TC425841, CF554444, TC424204, TC387579, TC406870, TC388914, TC393814, TC387621, TC398633, TC394716, TC446235, TC380943, TC393830, TC403885, TC420735, TC404371, TC384219, TC386961, TC416685, TC387344, TC412483, TC389090, TC389375, TC382655, CK200433, TC406038, TC395351, TC418032, CK210754, TC410147, TC384454, TC411287, TC386237, TC393904, TC390994, TC391143, TC381463, TC403157, TC386414, TC395303, TC400755, TC440636, TC403986, TC458987, TC404843, TC431879, CD876572, TC385365, TC401210, DR737360, TC379357, TC390135, TC397885, TC386279, TC411480, TC411684, TC380662, TC393960, TC389162, DR739471, TC402534, TC443387, TC415083, TC386535, GH729256, TC406584, TC386519, TC402440, TC417992, TC391411, TC394307, TC375146, TC389589, TC452762, TC386344, TC381963, TC383176, CD878039, TC374879, TC386422, TC374164, TC388688, TC388547, TC414606, TC388822, TC377496, TC388976, TC396365, TC429771, TC423576, TC386639, TC416658, TC393561, TC393692, TC391671, TC393554, TC407614, TC382928, TC379436, TC417363, TC403573, TC403929, TC413700, TC449043, TC379635, TC440321, TC433589, TC377410, TC389816, TC392323, TC413066, CB307332, CK211469, TC404842, TC381817, TC397729, TC403803, TC396895, TC382342, TC393106, TC401758, TC397676, TC396460, TC432504, TC417067, TC389661, TC428944 |
| [Chromosome](http://amigo.geneontology.org/cgi-bin/amigo/go.cgi?action=query&view=query&query=GO:0005694&search_constraint=terms) | TC461921, TC410352, TC386040, TC390393, TC416529, TC397258, CV775873, TC396230, TC460760, TC427210, TC416069, BQ838511, TC398052, CA710880, TC400388, TC433162, TC394459, TC416154, TC384738, TC423252, TC397660, TC389363, TC388410, CK203550, TC416493, TC458562, TC406264, TC380063, TC398538, TC410063, TC378054, TC448471, TC395453, TC410194, TC392875, TC435546, TC384735, TC390569, TC405540, TC419057, TC403872, TC425847, CJ727624, TC398714, BQ294582, CJ550278, TC413427, TC411128, TC401915, TC397444, TC398304, TC425878, TC393100, TC433957, TC417012, CA632212, DR739994, TC459193, TC394916, TC394206, TC394820, TC406236, TC418073, TC378274, TC391130, TC417308, CK217367, TC445767, TC446038, TC413460, TC379853, TC370315, BQ609416, CK214702, TC449504, TC395723, TC405440, CK213497, CJ792862, TC389718, TC404052, TC377225, TC381943, TC412520, TC432001, TC413392, BQ607161, TC402308, TC434820, TC441343, TC379711, TC400330, CA595837, TC379903, TC423265, TC392272, TC377061, TC401244, CA730421, TC446465, TC386313, CV759879, TC408907, CA606693, TC391438, CK211707, TC386963, TC412732, TC405695, TC392778, TC396751, TC397312, TC405030, CK211589, TC388403, TC376527, CA615187, TC416695, TC385780, TC406469, TC405784, TC399919, TC407572, TC372175, TC410690, TC406708, TC419747, TC410074, TC402186, TC405356, CV763657, TC402603, TC441241, TC413571, TC413043, CA729339, CK214224, TC408299, BQ239045, CA709177, TC376263, TC416169, TC381218, TC408229, TC422425, CA613620, TC398805, TC369092, CV781430, TC388520, TC411191, TC399342, TC434831, TC426326, TC381462, CD882425, TC373914, TC392263, TC392303, TC394796, TC435533, TC374240, TC425841, CF554444, TC424204, TC387579, TC406870, TC388914, TC393814, TC387621, TC398633, TC394716, TC446235, TC380943, TC393830, TC403885, TC420735, TC404371, TC384219, TC386961, TC416685, TC387344, TC412483, TC389090, TC389375, TC382655, CK200433, TC406038, TC395351, TC418032, CK210754, TC410147, TC384454, TC411287, TC386237, TC393904, TC390994, TC391143, TC381463, TC403157, TC386414, TC395303, TC400755, TC440636, TC403986, TC458987, TC404843, TC431879, CD876572, TC385365, TC401210, DR737360, TC379357, TC390135, TC397885, TC386279, TC411480, TC411684, TC380662, TC393960, TC389162, DR739471, TC402534, TC443387, TC415083, TC386535, GH729256, TC406584, TC386519, TC402440, TC417992, TC391411, TC394307, TC375146, TC389589, TC452762, TC386344, TC381963, TC383176, CD878039, TC374879, TC386422, TC374164, TC388688, TC388547, TC414606, TC388822, TC377496, TC388976, TC396365, TC429771, TC423576, TC386639, TC416658, TC393561, TC393692, TC391671, TC393554, TC407614, TC382928, TC379436, TC417363, TC403573, TC403929, TC413700, TC394118, TC449043, TC379635, TC440321, TC433589, TC377410, TC389816, TC392323, TC413066, CB307332, CK211469, TC404842, TC381817, TC397729, TC403803, TC396895, TC382342, TC393106, TC401758, TC397676, TC396460, TC432504, TC417067, TC389661, TC428944 |
| [Chromatin](http://amigo.geneontology.org/cgi-bin/amigo/go.cgi?action=query&view=query&query=GO:0000785&search_constraint=terms) | TC461921, TC410352, TC386040, TC390393, TC416529, TC397258, CV775873, TC396230, TC460760, TC427210, TC416069, BQ838511, TC398052, CA710880, TC400388, TC433162, TC394459, TC416154, TC384738, TC423252, TC397660, TC389363, TC388410, CK203550, TC416493, TC458562, TC406264, TC380063, TC398538, TC410063, TC378054, TC448471, TC395453, TC410194, TC392875, TC435546, TC384735, TC390569, TC405540, TC419057, TC403872, TC425847, CJ727624, TC398714, BQ294582, CJ550278, TC413427, TC411128, TC401915, TC397444, TC398304, TC425878, TC393100, TC433957, TC417012, CA632212, DR739994, TC459193, TC394916, TC394206, TC394820, TC406236, TC418073, TC378274, TC391130, TC417308, CK217367, TC445767, TC446038, TC413460, TC379853, BQ609416, CK214702, TC449504, TC395723, TC405440, CK213497, CJ792862, TC389718, TC404052, TC377225, TC381943, TC412520, TC432001, TC413392, BQ607161, TC402308, TC434820, TC441343, TC379711, TC400330, CA595837, TC379903, TC423265, TC392272, TC401244, CA730421, TC446465, TC386313, CV759879, TC408907, CA606693, TC391438, CK211707, TC386963, TC412732, TC405695, TC392778, TC396751, TC397312, TC405030, CK211589, TC388403, TC376527, CA615187, TC416695, TC385780, TC406469, TC405784, TC399919, TC407572, TC372175, TC410690, TC406708, TC419747, TC410074, TC402186, CV763657, TC405356, TC402603, TC441241, TC413571, TC413043, CA729339, CK214224, TC408299, BQ239045, CA709177, TC376263, TC416169, TC381218, TC408229, TC422425, CA613620, TC398805, CV781430, TC388520, TC411191, TC399342, TC434831, TC426326, TC381462, CD882425, TC373914, TC392263, TC392303, TC435533, TC374240, TC425841, CF554444, TC424204, TC387579, TC406870, TC388914, TC393814, TC387621, TC398633, TC394716, TC446235, TC380943, TC393830, TC420735, TC403885, TC404371, TC384219, TC386961, TC416685, TC387344, TC412483, TC389090, TC389375, TC382655, CK200433, TC406038, TC395351, TC418032, CK210754, TC410147, TC384454, TC411287, TC386237, TC393904, TC390994, TC391143, TC381463, TC403157, TC386414, TC395303, TC400755, TC440636, TC403986, TC458987, TC404843, TC431879, CD876572, TC385365, TC401210, DR737360, TC379357, TC390135, TC397885, TC386279, TC411480, TC411684, TC380662, TC393960, TC389162, DR739471, TC402534, TC443387, TC415083, TC386535, GH729256, TC406584, TC386519, TC402440, TC417992, TC391411, TC394307, TC375146, TC389589, TC452762, TC386344, TC381963, TC383176, CD878039, TC374879, TC386422, TC388688, TC388547, TC414606, TC388822, TC377496, TC388976, TC396365, TC429771, TC423576, TC386639, TC416658, TC393561, TC393692, TC391671, TC393554, TC407614, TC382928, TC379436, TC417363, TC403573, TC403929, TC413700, TC449043, TC379635, TC440321, TC433589, TC377410, TC389816, TC413066, TC392323, CB307332, CK211469, TC404842, TC381817, TC397729, TC403803, TC396895, TC382342, TC393106, TC401758, TC397676, TC396460, TC432504, TC417067, TC389661, TC428944 |
| [Synapse part](http://amigo.geneontology.org/cgi-bin/amigo/go.cgi?action=query&view=query&query=GO:0044456&search_constraint=terms) | TC398304, TC393100, TC433957, CK210754, TC382928, CV775873, TC441241, TC413571, TC417363, TC386237, TC394916, TC386313, TC393904, TC390994, TC398052, TC425841, TC378274, TC403929, TC387579, CA606693, TC394459, TC384738, CK211707, TC386963, TC388410, TC377410, TC381218, TC395303, TC422425, TC458562, TC400755, TC458987, TC396751, TC397312, CK211589, CA615187, TC401210, TC386961, TC398514, TC390135, TC397729, TC377225, TC411191, TC388547, TC387344, TC405784, TC399919, TC407572, TC372175, TC409650, TC381462, TC396365, TC419747, TC380662, TC401758, TC398714, TC392263, TC389661 |
| [Nuclear part](http://amigo.geneontology.org/cgi-bin/amigo/go.cgi?action=query&view=query&query=GO:0044428&search_constraint=terms) | TC392303, TC410352, TC407978, TC390393, TC416529, TC397258, TC396230, TC435533, TC416069, BQ838511, TC374240, CA710880, TC424204, TC387579, TC371242, TC416154, TC423252, TC397660, TC393814, TC389363, TC388914, TC379171, TC416493, TC376220, TC458562, TC446235, TC387410, TC398538, TC397176, TC410063, TC380433, TC403885, TC420735, TC404371, TC395453, TC386961, TC384219, TC410194, TC398606, TC384735, TC390569, TC405540, TC416685, TC372664, TC419057, TC403872, TC389090, TC412483, TC389375, TC404413, TC382655, CK200433, TC456230, TC398714, TC373994, BQ294582, TC370114, TC406038, CJ550278, TC395351, TC401915, TC418032, TC397444, CK210754, TC417012, CA632212, DR739994, TC384454, TC411287, TC394916, EB512907, TC393904, TC390994, TC406236, TC378274, TC391130, CK217367, TC391128, TC409599, TC446038, TC423804, TC379853, TC370315, CK214702, TC400755, TC403986, TC395723, TC405440, CK213497, TC377190, TC391613, TC379536, TC385233, TC385365, TC401210, DR737360, TC379357, TC387135, TC377225, TC386279, TC411684, TC381943, TC412520, TC380662, TC413392, BQ607161, TC393960, TC382045, TC404606, TC369628, TC389162, DR739471, TC402534, TC434820, TC443387, TC441343, TC379711, TC388566, TC415083, TC379903, TC423265, TC390630, TC392272, TC377061, TC401244, TC417077, TC406584, TC386313, CV759879, TC408907, TC402440, TC391438, TC417992, TC417341, TC394307, TC375146, TC386963, TC389589, TC412732, TC452762, TC405475, TC405695, TC392778, TC397312, TC373637, CK211589, TC388403, TC383909, TC449463, TC386422, TC416695, TC388688, TC383701, TC406469, TC388822, TC410690, TC377496, TC406708, TC429771, TC423576, TC409459, TC402186, TC416658, TC405356, TC374230, TC393561, TC435799, TC393948, TC393692, TC391671, TC393554, TC407614, TC402603, TC438243, TC441241, TC449724, TC413571, TC379436, TC413043, CA729339, TC460615, CK214224, TC408299, TC387710, TC394118, TC376263, TC379635, TC416169, TC373259, TC433589, TC377410, TC381218, TC389816, TC399352, TC392323, TC413066, CA613620, TC393820, CK211469, CB307332, TC400362, TC404842, TC435808, TC381817, TC397729, TC403803, TC399342, TC434831, TC396895, TC426326, TC381462, TC418850, TC382342, TC393106, TC401758, TC396460, TC392263, TC428944, TC389661 |
| [Synapse](http://amigo.geneontology.org/cgi-bin/amigo/go.cgi?action=query&view=query&query=GO:0045202&search_constraint=terms) | TC398304, TC393100, TC433957, CK210754, TC382928, CV775873, TC441241, TC413571, TC417363, TC386237, TC394916, TC386313, TC393904, TC390994, TC398052, TC425841, TC378274, TC403929, TC387579, CA606693, TC394459, TC384738, CK211707, TC386963, TC388410, TC377410, TC381218, TC395303, TC422425, TC458562, TC400755, TC458987, TC396751, TC397312, CK211589, CA615187, TC401210, TC386961, TC398514, TC390135, TC397729, TC377225, TC411191, TC388547, TC387344, TC405784, TC399919, TC407572, TC372175, TC409650, TC381462, TC396365, TC419747, TC380662, TC401758, TC398714, TC392263, TC389661, TC382045 |
| [Replication fork](http://amigo.geneontology.org/cgi-bin/amigo/go.cgi?action=query&view=query&query=GO:0005657&search_constraint=terms) | TC389162, TC386040, TC402308, TC393692, TC397258, TC402603, TC377061, TC379436, TC411287, TC435533, TC386519, TC374240, CV759879, CK214224, TC408907, TC391143, TC433162, TC391438, TC433589, TC386414, TC398633, TC394716, TC440636, TC395723, TC380063, TC380943, TC393830, TC386344, CK211469, TC405030, TC378054, TC381963, TC388403, TC383176, TC404371, TC369092, TC389718, TC374164, TC384219, TC379357, TC386279, TC385780, TC381943, TC388976, TC382342, TC373914, TC432504, TC386639, TC397676, TC402186 |
| [Secretory granule membrane](http://amigo.geneontology.org/cgi-bin/amigo/go.cgi?action=query&view=query&query=GO:0030667&search_constraint=terms) | TC398304, TC393100, TC433957, CK210754, TC382928, CV775873, TC441241, TC417363, TC386237, TC394916, TC386313, TC390994, TC398052, TC425841, TC378274, TC403929, TC387579, CA606693, TC394459, TC384738, CK211707, TC386963, TC388410, TC395303, TC422425, TC458562, TC400755, TC458987, TC396751, CA615187, TC386961, TC398514, TC390135, TC377225, TC411191, TC388547, TC387344, TC405784, TC399919, TC407572, TC372175, TC396365, TC419747, TC380662, TC401758, TC398714, TC389661 |
| [Synaptic vesicle membrane](http://amigo.geneontology.org/cgi-bin/amigo/go.cgi?action=query&view=query&query=GO:0030672&search_constraint=terms) | TC398304, TC393100, TC433957, CK210754, TC382928, CV775873, TC441241, TC417363, TC386237, TC394916, TC386313, TC390994, TC398052, TC425841, TC378274, TC403929, TC387579, CA606693, TC394459, TC384738, CK211707, TC386963, TC388410, TC395303, TC422425, TC458562, TC400755, TC458987, TC396751, CA615187, TC386961, TC398514, TC390135, TC377225, TC411191, TC388547, TC387344, TC405784, TC399919, TC407572, TC372175, TC396365, TC419747, TC380662, TC401758, TC398714, TC389661 |
| [Zymogen granule membrane](http://amigo.geneontology.org/cgi-bin/amigo/go.cgi?action=query&view=query&query=GO:0042589&search_constraint=terms) | TC398304, TC393100, TC433957, CK210754, TC382928, CV775873, TC441241, TC417363, TC386237, TC394916, TC386313, TC390994, TC398052, TC425841, TC378274, TC403929, TC387579, CA606693, TC394459, TC384738, CK211707, TC386963, TC388410, TC395303, TC422425, TC458562, TC400755, TC458987, TC396751, CA615187, TC386961, TC398514, TC390135, TC377225, TC411191, TC388547, TC387344, TC405784, TC399919, TC407572, TC372175, TC396365, TC419747, TC380662, TC401758, TC398714, TC389661 |
| [Zymogen granule](http://amigo.geneontology.org/cgi-bin/amigo/go.cgi?action=query&view=query&query=GO:0042588&search_constraint=terms) | TC398304, TC393100, TC433957, CK210754, TC382928, CV775873, TC441241, TC417363, TC386237, TC394916, TC386313, TC390994, TC398052, TC425841, TC378274, TC403929, TC387579, CA606693, TC394459, TC384738, CK211707, TC386963, TC388410, TC395303, TC422425, TC458562, TC400755, TC458987, TC396751, CA615187, TC386961, TC398514, TC390135, TC377225, TC411191, TC388547, TC387344, TC405784, TC399919, TC407572, TC372175, TC396365, TC419747, TC380662, TC401758, TC398714, TC389661 |
| [SNARE complex](http://amigo.geneontology.org/cgi-bin/amigo/go.cgi?action=query&view=query&query=GO:0031201&search_constraint=terms) | TC398304, TC393100, TC433957, CK210754, TC382928, CV775873, TC441241, TC417363, TC386237, TC394916, TC386313, TC390994, TC398052, TC425841, TC378274, TC403929, TC387579, CA606693, TC394459, TC384738, CK211707, TC386963, TC388410, TC395303, TC422425, TC458562, TC400755, TC458987, TC396751, CA615187, TC386961, TC398514, TC390135, TC377225, TC411191, TC388547, TC387344, TC405784, TC399919, TC407572, TC372175, TC396365, TC419747, TC380662, TC401758, TC398714, TC389661 |
| [Synaptic vesicle](http://amigo.geneontology.org/cgi-bin/amigo/go.cgi?action=query&view=query&query=GO:0008021&search_constraint=terms) | TC398304, TC393100, TC433957, CK210754, TC382928, CV775873, TC441241, TC417363, TC386237, TC394916, TC386313, TC390994, TC398052, TC425841, TC378274, TC403929, TC387579, CA606693, TC394459, TC384738, CK211707, TC386963, TC388410, TC395303, TC422425, TC458562, TC400755, TC458987, TC396751, CA615187, TC386961, TC398514, TC390135, TC377225, TC411191, TC388547, TC387344, TC405784, TC399919, TC407572, TC372175, TC409650, TC396365, TC419747, TC380662, TC401758, TC398714, TC389661 |
| [Extracellular region](http://amigo.geneontology.org/cgi-bin/amigo/go.cgi?action=query&view=query&query=GO:0005576&search_constraint=terms) | TC392303, TC410352, TC390944, TC394796, CD902983, CV775873, TC397033, TC398052, CA710880, TC425841, TC387579, TC394459, TC371242, TC384738, TC423252, TC397660, TC389363, TC388914, TC388410, TC458562, TC446235, TC420735, TC395453, TC386961, TC384735, TC390569, TC416685, TC387344, TC372664, TC407076, TC403872, TC389090, TC389375, TC382655, CK200433, TC398714, TC370114, BQ294582, TC406038, TC395351, TC401915, TC398304, TC393100, TC433957, CK210754, DR739994, TC384454, TC386237, TC394916, TC393904, TC390994, TC378274, CK217367, TC391128, TC379853, TC395303, TC400755, TC405440, TC458987, TC398730, TC401210, TC390135, DR737360, TC377225, TC388049, CK201148, TC380662, BQ607161, TC406807, DR739471, TC434820, TC441343, TC443387, TC379711, TC377308, TC392272, TC417077, TC386313, TC402440, CA606693, TC394307, CK211707, TC375146, TC386963, TC389589, TC394661, TC392778, TC396751, CK211589, TC370044, CA615187, TC386422, TC388688, TC388547, TC405784, TC399919, TC372175, TC407572, TC369199, TC388822, TC377496, TC406708, TC396365, TC419747, TC429771, TC409459, TC423576, TC416658, TC393561, TC391671, TC393554, TC407614, TC459656, TC382928, TC441241, TC438243, TC413571, CA729339, TC417363, TC403929, TC380882, TC384010, TC425690, TC377410, TC381218, TC389816, TC422425, TC384194, CA613620, TC461622, TC403588, TC388520, TC397729, TC411191, DR740372, TC396895, TC434831, TC426326, TC381462, TC393106, TC401758, TC392263, TC396460, TC389661, TC428944 |
| [Stored secretory granule](http://amigo.geneontology.org/cgi-bin/amigo/go.cgi?action=query&view=query&query=GO:0030141&search_constraint=terms) | TC398304, TC393100, TC433957, CK210754, TC382928, CV775873, TC441241, TC417363, TC386237, TC394916, TC386313, TC390994, TC398052, TC425841, TC378274, TC403929, TC387579, CA606693, TC394459, TC384738, CK211707, TC386963, TC388410, TC395303, TC422425, TC458562, TC400755, TC458987, TC396751, CA615187, TC386961, TC398514, TC390135, TC377225, TC411191, TC388547, TC387344, TC405784, TC399919, TC407572, TC372175, TC396365, TC419747, TC380662, TC401758, TC398714, TC389661 |
| [Integral to plasma membrane](http://amigo.geneontology.org/cgi-bin/amigo/go.cgi?action=query&view=query&query=GO:0005887&search_constraint=terms) | TC410352, DR739471, TC404158, TC434820, TC443387, TC379711, TC392272, CV775873, TC417077, TC374240, TC386313, TC398052, CA710880, TC390402, TC425841, TC402440, TC387579, TC394459, TC371242, TC384738, TC423252, TC394307, TC397660, CK211707, TC375146, TC386963, TC389363, TC388914, TC388410, TC389589, TC421914, TC446235, TC378271, TC392778, TC397312, TC420735, TC395453, TC386422, TC386961, TC388688, TC390569, TC416685, TC403872, TC412150, TC399919, TC407572, TC389090, TC372175, TC389375, TC388822, TC396365, TC406708, TC377496, TC419747, TC382655, TC429771, CK200433, TC423576, BQ294582, TC370114, TC406038, CJ550278, TC416658, TC395351, TC386646, TC401915, TC393561, TC393100, TC435799, TC391671, TC393554, TC407614, TC382928, TC459656, DR739994, TC438243, TC384454, CA729339, TC417363, TC386237, TC393904, CK217367, TC428066, TC425690, TC379853, TC377410, TC381218, TC395303, TC389816, TC400755, TC395723, TC405440, TC376420, TC458987, CA613620, TC401210, DR737360, TC390135, TC397729, TC377225, TC434831, TC396895, TC426326, TC381462, TC393106, TC401758, BQ607161, TC406807, TC396460, TC392263, TC428944, TC389661 |
| [Intrinsic to plasma membrane](http://amigo.geneontology.org/cgi-bin/amigo/go.cgi?action=query&view=query&query=GO:0031226&search_constraint=terms) | TC410352, DR739471, TC404158, TC434820, TC443387, TC379711, TC392272, CV775873, TC417077, TC374240, TC386313, TC398052, CA710880, TC390402, TC425841, TC402440, TC387579, TC394459, TC371242, TC384738, TC423252, TC394307, TC397660, CK211707, TC375146, TC386963, TC389363, TC388914, TC388410, TC389589, TC421914, TC446235, TC378271, TC392778, TC397312, TC420735, TC395453, TC386422, TC386961, TC388688, TC390569, TC416685, TC403872, TC412150, TC399919, TC407572, TC389090, TC372175, TC389375, TC388822, TC396365, TC406708, TC377496, TC419747, TC382655, TC429771, CK200433, TC423576, BQ294582, TC370114, TC406038, CJ550278, TC416658, TC395351, TC386646, TC401915, TC393561, TC393100, TC435799, TC391671, TC393554, TC407614, TC382928, TC459656, DR739994, TC438243, TC384454, CA729339, TC417363, TC386237, TC393904, CK217367, TC428066, TC425690, TC379853, TC377410, TC381218, TC395303, TC389816, TC400755, TC395723, TC405440, TC376420, TC458987, CA613620, TC401210, DR737360, TC390135, TC397729, TC377225, TC434831, TC396895, TC426326, TC381462, TC393106, TC401758, BQ607161, TC406807, TC396460, TC392263, TC428944, TC389661 |
| [Nucleus](http://amigo.geneontology.org/cgi-bin/amigo/go.cgi?action=query&view=query&query=GO:0005634&search_constraint=terms) | TC461921, TC410352, TC386040, TC373613, TC407978, TC390393, TC444546, TC416529, TC397258, TC389190, CV775873, TC396230, TC375864, TC420420, TC460760, TC408524, TC427210, TC416069, BQ838511, TC398052, TC375313, CA710880, TC400388, TC457112, TC421871, TC398862, TC433162, TC394459, TC371242, TC416154, TC384738, TC423252, TC397660, TC389363, TC379171, CK203550, TC416493, TC458562, TC416442, TC406264, TC387410, TC380063, TC398538, TC397176, TC418414, TC410063, TC378054, TC448471, TC395453, TC410194, TC392875, TC392329, TC409843, TC435546, TC384735, TC390569, TC405540, TC407076, TC419057, TC403872, TC425847, CJ727624, TC398714, BQ294582, CJ550278, TC398970, TC413427, TC411128, TC401915, GH732878, TC397444, TC398304, TC425878, TC393100, TC433957, TC417012, TC423239, CA632212, DR739994, TC459193, TC394916, TC394206, TC394820, TC398731, TC406236, TC418073, TC378274, TC391130, TC417308, CK217367, TC391128, TC409599, TC445767, TC446038, TC423804, TC413460, TC379853, TC370315, BQ609416, CK214702, TC449504, TC395723, TC405440, CK213497, CJ792862, TC379536, TC385233, TC398730, TC389718, TC404052, TC377225, TC381943, TC412520, TC432001, TC413392, TC418928, BQ607161, TC403977, TC406807, TC382045, TC404606, TC369628, TC434820, TC416906, TC402308, TC441343, TC379711, TC400330, TC378790, CA595837, TC379903, TC423265, TC392272, TC377061, TC401244, CA730421, TC417077, TC417388, TC446465, TC386313, CV759879, CK208222, TC375539, TC408907, CA606693, TC391438, CK211707, TC370912, TC386963, TC412732, TC431201, TC405695, TC392778, TC396751, TC397312, TC405030, CK211589, TC388403, TC383909, TC376527, CA615187, TC416695, TC419584, TC383701, TC406469, TC385780, TC374392, TC405784, TC399919, TC407572, TC372175, TC410690, TC406708, TC419747, TC409459, TC410074, TC402186, TC386646, TC405356, CV763657, TC423354, TC402603, TC403264, TC441241, TC449724, TC413571, TC413043, CA729339, CK214224, TC408299, BQ239045, CA709177, TC376263, TC416169, TC425690, TC381218, TC399352, TC408229, TC422425, CA613620, TC398805, TC400362, CV781430, TC388520, TC395872, TC411191, TC399342, TC434831, TC432185, TC426326, TC381462, CD882425, TC373914, TC392263, TC450285, TC392303, TC394796, TC372677, TC435533, TC374240, TC425841, CF554444, TC424204, TC387579, TC406870, TC388914, TC393814, TC376220, TC387621, TC398633, TC394716, TC446235, TC404926, TC402545, TC380943, TC393830, TC380433, TC403885, TC420735, TC404371, TC427405, TC386961, TC373615, TC384219, TC398606, TC397562, TC416685, TC387344, TC372664, TC389090, TC412483, TC389375, TC404413, TC382655, CK200433, TC456230, TC370114, TC373994, TC406038, TC393970, TC395351, TC459245, TC418032, CK210754, TC417769, TC410147, TC384454, TC411287, TC380329, TC386237, EB512907, TC393904, TC390994, TC391143, TC381463, TC403157, TC386414, TC400755, TC403986, TC440636, TC405511, TC406193, TC405615, TC376420, TC377190, TC404843, TC423110, TC431879, TC422348, TC391613, CD876572, TC385365, TC401210, DR737360, TC379357, TC390135, TC387135, TC386279, TC397885, TC411684, TC411480, TC380662, TC393960, TC426358, TC389162, DR739471, TC373251, TC402534, TC443387, TC388566, TC415083, TC390630, TC386535, GH729256, TC407340, TC406584, TC386519, TC402440, TC391411, TC417992, TC417341, TC394307, TC375146, TC388665, TC454407, TC389589, TC452762, TC405475, TC386344, TC394661, TC373637, TC381963, TC383176, CD878039, TC371455, TC449463, TC386422, TC388688, TC400181, TC414606, TC388547, TC388822, TC396365, TC377496, TC388976, TC429771, TC423576, TC386639, TC375530, TC416658, TC374230, TC393561, TC393948, TC435799, TC393692, TC391671, TC393554, TC407614, TC459656, TC382928, TC438243, TC391007, TC379436, TC417363, TC460615, TC403573, TC411941, TC387710, TC413700, TC403929, TC394118, TC449043, TC380882, TC423880, TC440321, TC379635, TC373259, TC433589, TC377410, TC389816, TC372530, TC392323, TC413066, TC393820, CK211469, CB307332, TC404842, TC435808, TC381817, TC403588, TC397729, TC403803, TC396895, TC418850, TC440526, TC382342, TC393106, TC401758, TC432504, TC397676, TC396460, TC428944, TC389661, TC417067 |
| [Clathrin coated vesicle membrane](http://amigo.geneontology.org/cgi-bin/amigo/go.cgi?action=query&view=query&query=GO:0030665&search_constraint=terms) | TC398304, TC393100, TC433957, CK210754, TC382928, CV775873, TC441241, TC417363, TC386237, TC394916, TC386313, TC390994, TC398052, TC425841, TC378274, TC403929, TC387579, CA606693, TC394459, TC384738, CK211707, TC386963, TC388410, TC395303, TC422425, TC458562, TC400755, TC458987, TC396751, CA615187, TC386961, TC398514, TC390135, TC377225, TC411191, TC388547, TC387344, TC405784, TC399919, TC407572, TC372175, TC409650, TC396365, TC419747, TC380662, TC401758, TC398714, TC389661 |
| [Cytoplasmic vesicle membrane](http://amigo.geneontology.org/cgi-bin/amigo/go.cgi?action=query&view=query&query=GO:0030659&search_constraint=terms) | TC398304, TC393100, TC433957, CK210754, TC382928, CV775873, TC441241, TC417363, TC386237, TC394916, TC386313, TC390994, TC398052, TC425841, TC378274, TC403929, TC385515, TC387579, CA606693, TC394459, TC384738, CK211707, TC386963, TC388410, TC395303, TC422425, TC458562, TC400755, TC458987, TC396751, CA615187, TC386961, TC398514, TC390135, TC377225, TC411191, TC388547, TC387344, TC405784, TC399919, TC407572, TC372175, TC409650, TC396365, TC419747, TC380662, TC401758, TC398714, TC389661 |
| [Coated vesicle membrane](http://amigo.geneontology.org/cgi-bin/amigo/go.cgi?action=query&view=query&query=GO:0030662&search_constraint=terms) | TC398304, TC393100, TC433957, CK210754, TC382928, CV775873, TC441241, TC417363, TC386237, TC394916, TC386313, TC390994, TC398052, TC425841, TC378274, TC403929, TC385515, TC387579, CA606693, TC394459, TC384738, CK211707, TC386963, TC388410, TC395303, TC422425, TC458562, TC400755, TC458987, TC396751, CA615187, TC386961, TC398514, TC390135, TC377225, TC411191, TC388547, TC387344, TC405784, TC399919, TC407572, TC372175, TC409650, TC396365, TC419747, TC380662, TC401758, TC398714, TC389661 |
| [Cytoplasmic vesicle part](http://amigo.geneontology.org/cgi-bin/amigo/go.cgi?action=query&view=query&query=GO:0044433&search_constraint=terms) | TC398304, TC393100, TC433957, CK210754, TC382928, CV775873, TC441241, TC417363, TC386237, TC394916, TC386313, TC390994, TC398052, TC425841, TC378274, TC403929, TC385515, TC387579, CA606693, TC394459, TC384738, CK211707, TC386963, TC388410, TC395303, TC422425, TC458562, TC400755, TC458987, TC396751, CA615187, TC386961, TC398514, TC390135, TC377225, TC411191, TC388547, TC387344, TC405784, TC399919, TC407572, TC372175, TC409650, TC396365, TC419747, TC380662, TC401758, TC398714, TC389661 |
| [Yesicle membrane](http://amigo.geneontology.org/cgi-bin/amigo/go.cgi?action=query&view=query&query=GO:0012506&search_constraint=terms) | TC398304, TC393100, TC433957, CK210754, TC382928, CV775873, TC441241, TC417363, TC386237, TC394916, TC386313, TC390994, TC398052, TC425841, TC378274, TC403929, TC385515, TC387579, CA606693, TC394459, TC384738, CK211707, TC386963, TC388410, TC395303, TC422425, TC458562, TC400755, TC458987, TC396751, CA615187, TC386961, TC398514, TC390135, TC377225, TC411191, TC388547, TC387344, TC405784, TC399919, TC407572, TC372175, TC409650, TC396365, TC419747, TC380662, TC401758, TC398714, TC389661 |
| [Postsynaptic membrane](http://amigo.geneontology.org/cgi-bin/amigo/go.cgi?action=query&view=query&query=GO:0045211&search_constraint=terms) | TC393100, TC382928, CV775873, TC417363, TC386237, TC386313, TC393904, TC398052, TC425841, TC387579, TC394459, TC384738, CK211707, TC386963, TC388410, TC377410, TC381218, TC395303, TC400755, TC458987, TC397312, TC401210, TC386961, TC390135, TC397729, TC377225, TC372175, TC407572, TC399919, TC396365, TC381462, TC419747, TC401758, TC392263, TC389661 |
| [Integral to membrane](http://amigo.geneontology.org/cgi-bin/amigo/go.cgi?action=query&view=query&query=GO:0016021&search_constraint=terms) | TC410352, TC404158, TC373958, TC394796, CV775873, TC374240, TC398052, CA710880, TC425841, TC398862, TC387579, TC394459, TC371242, TC384738, TC423252, TC397660, TC389363, TC388914, TC388410, TC458562, TC421914, TC446235, TC378271, TC418414, TC420735, TC395453, TC386961, TC390569, TC416685, TC387344, TC407076, TC403872, TC418365, TC389090, TC389375, TC382655, CK200433, TC398714, TC370114, BQ294582, TC406038, CJ550278, TC395351, TC401915, TC456619, TC398304, TC393100, TC433957, CK210754, TC412569, DR739994, TC384454, TC386237, TC394916, TC409343, TC393904, TC390994, TC378274, CK217367, TC428066, TC379853, TC370315, TC395303, TC400755, TC395723, TC405440, TC376420, TC458987, TC398730, TC401210, TC390135, DR737360, TC398514, TC387135, TC377225, TC380662, BQ607161, TC406807, DR739471, TC416906, TC434820, TC443387, TC379711, TC390630, TC392272, TC377061, TC417077, TC417388, TC386313, TC390402, TC402440, CA606693, TC394307, CK211707, TC375146, TC386963, TC389589, TC392778, TC397312, TC396751, CK211589, TC383909, TC449463, CA615187, TC386422, TC388688, TC388547, TC405784, TC412150, TC399919, TC407572, TC372175, TC388822, TC377496, TC406708, TC396365, TC419747, TC429771, TC409459, TC423576, TC416658, TC386646, TC393561, TC393948, TC435799, TC386322, TC391671, TC393554, TC407614, TC459656, TC382928, TC441241, TC438243, TC413571, CA729339, TC417363, TC403929, TC394118, TC425690, TC377410, TC381218, TC389816, TC422425, TC384194, TC446092, CA613620, TC397729, TC411191, TC432185, TC396895, TC434831, TC426326, TC381462, TC393106, TC401758, TC392263, TC396460, TC389661, TC428944 |
| [Presynaptic membrane](http://amigo.geneontology.org/cgi-bin/amigo/go.cgi?action=query&view=query&query=GO:0042734&search_constraint=terms) | TC393100, TC382928, CV775873, TC417363, TC386237, TC386313, TC393904, TC398052, TC425841, TC387579, TC394459, TC384738, CK211707, TC386963, TC388410, TC377410, TC381218, TC395303, TC400755, TC458987, TC397312, TC401210, TC386961, TC390135, TC397729, TC377225, TC372175, TC407572, TC399919, TC396365, TC381462, TC419747, TC401758, TC392263, TC389661 |
| [Clathrin-coated vesicle](http://amigo.geneontology.org/cgi-bin/amigo/go.cgi?action=query&view=query&query=GO:0030136&search_constraint=terms) | TC398304, TC393100, TC433957, CK210754, TC382928, CV775873, TC441241, TC417363, TC386237, TC394916, TC386313, TC390994, TC398052, TC425841, TC378274, TC403929, TC387579, CA606693, TC394459, TC384738, CK211707, TC386963, TC388410, TC395303, TC422425, TC458562, TC400755, TC458987, TC396751, CA615187, TC386961, TC398514, TC390135, TC377225, TC411191, TC388547, TC387344, TC405784, TC399919, TC407572, TC372175, TC409650, TC396365, TC419747, TC380662, TC401758, TC398714, TC389661 |
| [Coated vesicle](http://amigo.geneontology.org/cgi-bin/amigo/go.cgi?action=query&view=query&query=GO:0030135&search_constraint=terms) | TC398304, TC393100, TC433957, CK210754, TC382928, CV775873, TC441241, TC417363, TC386237, TC394916, TC386313, TC390994, TC398052, TC425841, TC378274, TC403929, TC385515, TC387579, CA606693, TC394459, TC384738, CK211707, TC386963, TC388410, TC395303, TC422425, TC458562, TC400755, TC458987, TC396751, CA615187, TC386961, TC398514, TC390135, TC377225, TC411191, TC388547, TC387344, TC405784, TC399919, TC407572, TC372175, TC409650, TC396365, TC419747, TC380662, TC401758, TC398714, TC389661 |
| [Plasma membrane part](http://amigo.geneontology.org/cgi-bin/amigo/go.cgi?action=query&view=query&query=GO:0044459&search_constraint=terms) | TC410352, TC404158, CV775873, TC374240, TC398052, CA710880, TC425841, TC387579, TC394459, TC371242, TC384738, TC423252, TC397660, TC389363, TC388914, TC388410, TC421914, TC446235, TC387410, TC378271, TC380433, TC420735, TC395453, TC386961, TC390569, TC416685, TC407076, TC403872, TC389090, TC389375, TC382655, CK200433, TC370114, BQ294582, TC406038, CJ550278, TC395351, TC401915, TC393100, DR739994, TC384454, TC386237, TC393904, CK217367, TC428066, TC379853, TC370315, TC395303, TC400755, TC395723, TC405440, TC376420, TC458987, TC423110, TC379536, TC398730, TC401210, DR737360, TC390135, TC387135, TC377225, TC409650, BQ607161, TC406807, TC382045, DR739471, TC434820, TC443387, TC379711, TC378790, TC392272, TC417077, TC386313, TC390402, TC402440, TC394307, TC375146, CK211707, TC386963, TC389589, TC392778, TC397312, TC386422, TC388688, TC412150, TC372175, TC407572, TC399919, TC377496, TC406708, TC396365, TC388822, TC429771, TC419747, TC423576, TC416658, TC386646, TC393561, TC435799, TC391671, TC407614, TC393554, TC459656, TC382928, TC438243, CA729339, TC417363, TC394118, TC380882, TC425690, TC377410, TC381218, TC389816, TC371387, CA613620, TC461622, TC403588, TC397729, TC396895, TC434831, TC381462, TC426326, TC393106, TC401758, TC392263, TC396460, TC389661, TC428944 |
| [Intrinsic to membrane](http://amigo.geneontology.org/cgi-bin/amigo/go.cgi?action=query&view=query&query=GO:0031224&search_constraint=terms) | TC410352, TC404158, TC373958, TC394796, CV775873, TC374240, TC398052, CA710880, TC425841, TC398862, TC387579, TC394459, TC371242, TC384738, TC423252, TC397660, TC389363, TC388914, TC388410, TC458562, TC421914, TC446235, TC378271, TC418414, TC420735, TC395453, TC386961, TC390569, TC416685, TC387344, TC407076, TC403872, TC418365, TC389090, TC389375, TC382655, CK200433, TC398714, TC370114, BQ294582, TC406038, CJ550278, TC395351, TC403679, TC401915, TC456619, TC398304, TC393100, TC433957, CK210754, TC412569, DR739994, TC384454, TC386237, TC394916, TC409343, TC393904, TC390994, TC378274, CK217367, TC428066, TC379853, TC370315, TC395303, TC400755, TC395723, TC405440, TC376420, TC458987, TC398730, TC401210, TC390135, DR737360, TC398514, TC387135, TC377225, TC380662, BQ607161, TC406807, DR739471, TC416906, TC434820, TC443387, TC379711, TC390630, TC392272, TC377061, TC417077, TC417388, TC386313, TC390402, TC402440, CA606693, TC394307, CK211707, TC375146, TC386963, TC389589, TC392778, TC397312, TC396751, CK211589, TC383909, TC449463, CA615187, TC386422, TC388688, TC388547, TC405784, TC412150, TC399919, TC407572, TC372175, TC388822, TC377496, TC406708, TC396365, TC419747, TC429771, TC409459, TC423576, TC416658, TC386646, TC393561, TC393948, TC435799, TC386322, TC391671, TC393554, TC407614, TC459656, TC382928, TC441241, TC438243, TC413571, CA729339, TC417363, TC403929, TC394118, TC425690, TC377410, TC381218, TC389816, TC422425, TC384194, TC446092, CA613620, TC397729, TC411191, TC432185, TC396895, TC434831, TC426326, TC381462, TC393106, TC401758, TC392263, TC396460, TC389661, TC428944 |
| [Non-membrane-bounded organelle](http://amigo.geneontology.org/cgi-bin/amigo/go.cgi?action=query&view=query&query=GO:0043228&search_constraint=terms) | TC461921, TC410352, TC386040, TC379942, TC407978, TC390393, TC444546, TC416529, TC397258, CV775873, TC396230, TC460760, TC451511, TC427210, TC416069, BQ838511, TC398052, TC387861, CA710880, TC400388, TC398862, TC433162, TC394459, TC371242, TC416154, TC384738, TC423252, TC397660, TC389363, TC379171, TC388410, CK203550, TC416493, TC458562, TC416442, TC421914, TC406264, TC387410, TC380063, TC398538, TC418414, CJ944525, TC410063, TC378054, TC448471, TC395453, TC410194, TC392875, TC435546, TC384735, TC390569, TC405540, TC407076, TC419057, TC403872, TC425847, TC391948, CJ727624, TC398714, BQ294582, CJ550278, TC381619, TC398970, TC457126, TC403580, TC413427, TC411128, TC401915, TC397444, TC398304, TC425878, TC393100, TC433957, TC417012, CA632212, DR739994, TC459193, TC413027, TC394916, TC394206, TC394820, TC406236, TC418073, TC378274, TC391130, TC417308, CK217367, TC409599, TC445767, TC446038, TC423804, TC413460, TC379853, TC370315, BQ609416, CK214702, TC449504, TC395723, TC405440, CK213497, CJ792862, TC389718, TC404052, TC377225, TC381943, TC412520, TC432001, TC413392, BQ607161, TC406807, TC382045, TC404606, TC369628, TC416906, TC402308, TC434820, TC441343, TC379711, TC400330, CA595837, TC379903, TC423265, TC392272, TC377061, TC401244, CA730421, TC417077, TC417388, TC446465, TC440066, TC386313, CV759879, TC408907, CA606693, TC391438, CK211707, TC386963, TC412732, TC431201, TC405695, TC392778, TC396751, TC397312, TC405030, CK211589, TC388403, TC383909, TC376527, CA615187, TC416695, TC388718, TC406469, TC385780, TC405784, TC399919, TC407572, TC372175, TC410690, TC406708, TC419747, TC409459, TC410074, TC402186, TC405356, CV763657, TC402603, TC403264, TC441241, TC449724, TC413571, TC413043, CA729339, CK214224, TC408299, BQ239045, TC368603, CA709177, TC376263, TC416169, TC425690, TC423182, TC381218, TC408229, TC422425, TC408312, CA613620, TC398805, TC369092, CV781430, TC388520, TC411191, TC400108, TC399342, TC434831, TC426326, TC381462, TC375431, CD882425, TC415501, TC396451, TC373914, TC392263, TC450285, TC392303, TC394796, TC445166, TC435533, TC374240, TC425841, TC389168, CF554444, TC424204, TC387579, TC406870, TC415685, TC388914, TC393814, TC387621, TC398633, TC394716, TC446235, TC410126, TC402545, TC378271, TC380943, TC393830, TC380433, TC403885, TC420735, TC435281, TC404371, TC386961, TC384219, TC398606, TC416685, TC387344, TC372664, TC389090, TC412483, TC389375, TC382655, CK200433, TC456230, TC370114, TC373994, TC406038, TC393970, TC376874, TC395351, TC418032, TC456619, CK210754, TC417769, TC410147, TC384454, TC411287, CA598430, TC386237, TC393904, TC390994, TC391143, TC399471, TC381463, TC403157, TC410066, TC395303, TC386414, TC400755, TC403986, TC440636, TC458987, TC377190, TC443814, TC404843, TC423110, TC431879, TC391613, CD876572, TC385365, TC382737, TC401210, DR737360, TC379357, TC390135, TC386279, TC397885, TC411684, TC411480, TC391785, TC380662, TC393960, TC395298, TC389162, DR739471, TC402534, TC443387, TC415083, TC390630, TC386535, GH729256, TC406584, TC386519, TC402440, TC417992, TC391411, TC417341, TC394307, TC409208, TC375146, CK199175, TC389589, TC381988, TC452762, TC405475, TC386344, TC373637, TC381963, TC383176, TC370044, CD878039, TC449463, TC374879, TC386422, TC374164, TC403328, TC388688, TC418845, TC388547, TC414606, TC388822, TC396365, TC377496, TC388976, TC429771, TC410078, TC423576, TC386639, TC416658, TC393561, TC393948, TC435799, TC393692, TC371970, TC391671, TC393554, TC407614, TC459656, TC382928, TC379436, TC417363, TC403573, TC387710, TC413700, TC403929, TC394118, TC449043, TC379635, TC440321, TC373259, TC433589, TC377410, TC418091, TC389816, TC392323, TC413066, TC391641, TC393820, CK211469, CB307332, TC404842, TC381817, TC397729, TC403803, TC396895, TC418850, TC382342, TC393106, TC390489, TC401758, TC432504, TC397676, TC396460, TC428944, TC389661, TC417067 |
| [Intracellular non-membrane-bounded organelle](http://amigo.geneontology.org/cgi-bin/amigo/go.cgi?action=query&view=query&query=GO:0043232&search_constraint=terms) | TC461921, TC410352, TC386040, TC379942, TC407978, TC390393, TC444546, TC416529, TC397258, CV775873, TC396230, TC460760, TC451511, TC427210, TC416069, BQ838511, TC398052, TC387861, CA710880, TC400388, TC398862, TC433162, TC394459, TC371242, TC416154, TC384738, TC423252, TC397660, TC389363, TC379171, TC388410, CK203550, TC416493, TC458562, TC416442, TC421914, TC406264, TC387410, TC380063, TC398538, TC418414, CJ944525, TC410063, TC378054, TC448471, TC395453, TC410194, TC392875, TC435546, TC384735, TC390569, TC405540, TC407076, TC419057, TC403872, TC425847, TC391948, CJ727624, TC398714, BQ294582, CJ550278, TC381619, TC398970, TC457126, TC403580, TC413427, TC411128, TC401915, TC397444, TC398304, TC425878, TC393100, TC433957, TC417012, CA632212, DR739994, TC459193, TC413027, TC394916, TC394206, TC394820, TC406236, TC418073, TC378274, TC391130, TC417308, CK217367, TC409599, TC445767, TC446038, TC423804, TC413460, TC379853, TC370315, BQ609416, CK214702, TC449504, TC395723, TC405440, CK213497, CJ792862, TC389718, TC404052, TC377225, TC381943, TC412520, TC432001, TC413392, BQ607161, TC406807, TC382045, TC404606, TC369628, TC416906, TC402308, TC434820, TC441343, TC379711, TC400330, CA595837, TC379903, TC423265, TC392272, TC377061, TC401244, CA730421, TC417077, TC417388, TC446465, TC440066, TC386313, CV759879, TC408907, CA606693, TC391438, CK211707, TC386963, TC412732, TC431201, TC405695, TC392778, TC396751, TC397312, TC405030, CK211589, TC388403, TC383909, TC376527, CA615187, TC416695, TC388718, TC406469, TC385780, TC405784, TC399919, TC407572, TC372175, TC410690, TC406708, TC419747, TC409459, TC410074, TC402186, TC405356, CV763657, TC402603, TC403264, TC441241, TC449724, TC413571, TC413043, CA729339, CK214224, TC408299, BQ239045, TC368603, CA709177, TC376263, TC416169, TC425690, TC423182, TC381218, TC408229, TC422425, TC408312, CA613620, TC398805, TC369092, CV781430, TC388520, TC411191, TC400108, TC399342, TC434831, TC426326, TC381462, TC375431, CD882425, TC415501, TC396451, TC373914, TC392263, TC450285, TC392303, TC394796, TC445166, TC435533, TC374240, TC425841, TC389168, CF554444, TC424204, TC387579, TC406870, TC415685, TC388914, TC393814, TC387621, TC398633, TC394716, TC446235, TC410126, TC402545, TC378271, TC380943, TC393830, TC380433, TC403885, TC420735, TC435281, TC404371, TC386961, TC384219, TC398606, TC416685, TC387344, TC372664, TC389090, TC412483, TC389375, TC382655, CK200433, TC456230, TC370114, TC373994, TC406038, TC393970, TC376874, TC395351, TC418032, TC456619, CK210754, TC417769, TC410147, TC384454, TC411287, CA598430, TC386237, TC393904, TC390994, TC391143, TC399471, TC381463, TC403157, TC410066, TC395303, TC386414, TC400755, TC403986, TC440636, TC458987, TC377190, TC443814, TC404843, TC423110, TC431879, TC391613, CD876572, TC385365, TC382737, TC401210, DR737360, TC379357, TC390135, TC386279, TC397885, TC411684, TC411480, TC391785, TC380662, TC393960, TC395298, TC389162, DR739471, TC402534, TC443387, TC415083, TC390630, TC386535, GH729256, TC406584, TC386519, TC402440, TC417992, TC391411, TC417341, TC394307, TC409208, TC375146, CK199175, TC389589, TC381988, TC452762, TC405475, TC386344, TC373637, TC381963, TC383176, TC370044, CD878039, TC449463, TC374879, TC386422, TC374164, TC403328, TC388688, TC418845, TC388547, TC414606, TC388822, TC396365, TC377496, TC388976, TC429771, TC410078, TC423576, TC386639, TC416658, TC393561, TC393948, TC435799, TC393692, TC371970, TC391671, TC393554, TC407614, TC459656, TC382928, TC379436, TC417363, TC403573, TC387710, TC413700, TC403929, TC394118, TC449043, TC379635, TC440321, TC373259, TC433589, TC377410, TC418091, TC389816, TC392323, TC413066, TC391641, TC393820, CK211469, CB307332, TC404842, TC381817, TC397729, TC403803, TC396895, TC418850, TC382342, TC393106, TC390489, TC401758, TC432504, TC397676, TC396460, TC428944, TC389661, TC417067 |
| [Neuron projection](http://amigo.geneontology.org/cgi-bin/amigo/go.cgi?action=query&view=query&query=GO:0043005&search_constraint=terms) | TC393100, TC382928, CV775873, TC413571, TC417363, TC386237, TC386313, TC374240, TC393904, TC398052, TC425841, TC387579, TC380882, TC394459, TC384738, CK211707, TC386963, TC388410, TC377410, TC381218, TC395303, TC400755, TC421914, TC395723, TC458987, TC378271, TC461622, TC380433, TC397312, CK211589, TC403588, TC401210, TC386961, TC390135, TC397729, TC377225, TC399919, TC407572, TC372175, TC396365, TC381462, TC419747, TC401758, TC392263, CJ550278, TC389661, TC382045 |
| [Plasma membrane](http://amigo.geneontology.org/cgi-bin/amigo/go.cgi?action=query&view=query&query=GO:0005886&search_constraint=terms) | TC410352, TC404158, TC407978, CV775873, TC374240, TC398052, CA710880, TC425841, TC397994, TC387579, TC394459, TC371242, TC384738, TC423252, TC397660, TC404978, TC389363, TC388914, TC388410, TC421914, TC446235, TC387410, TC378271, TC380433, TC420735, TC395453, TC386961, TC390569, TC416685, TC407076, TC403872, TC389090, TC389375, TC382655, CK200433, TC370114, BQ294582, TC406038, CJ550278, TC395351, TC401915, TC393100, DR739994, TC384454, TC386237, TC393904, CK217367, TC428066, TC379853, TC370315, TC395303, TC400755, TC395723, TC405440, TC376420, TC458987, TC423110, TC379536, TC398730, TC401210, DR737360, TC390135, TC387135, TC377225, TC409650, BQ607161, TC406807, TC382045, DR739471, TC434820, TC443387, TC379711, TC378790, TC392272, TC417077, TC386313, TC390402, TC369844, TC402440, TC394307, CK211707, TC375146, TC370912, TC386963, TC373583, TC389589, TC370178, TC392778, TC397312, CK211589, TC373678, TC386422, TC388688, TC412150, TC399919, TC372175, TC407572, TC388822, TC377496, TC406708, TC396365, TC419747, TC429771, TC423576, TC416658, TC384678, TC386646, TC393561, TC435799, TC391671, TC393554, TC407614, TC459656, TC382928, TC438243, TC413571, CA729339, TC417363, TC394118, TC380882, TC373848, TC425690, TC377410, TC381218, TC389816, TC371387, CA613620, TC461622, TC437163, TC403588, TC376933, TC385659, TC397729, TC387683, TC396895, TC434831, TC426326, TC381462, TC377749, TC393106, TC401758, TC392263, TC396460, TC389661, TC428944 |
| [Neuronal cell body](http://amigo.geneontology.org/cgi-bin/amigo/go.cgi?action=query&view=query&query=GO:0043025&search_constraint=terms) | TC393100, TC382928, CV775873, TC413571, TC417363, TC386237, TC386313, TC393904, TC398052, TC425841, TC387579, TC371242, TC394459, TC384738, CK211707, TC386963, TC388410, TC377410, TC381218, TC395303, TC400755, TC376420, TC458987, TC380433, TC397312, CK211589, TC401210, TC386961, TC390135, TC397729, TC377225, TC399919, TC407572, TC372175, TC396365, TC381462, TC419747, TC401758, TC392263, TC389661, TC382045 |
| [Cell body](http://amigo.geneontology.org/cgi-bin/amigo/go.cgi?action=query&view=query&query=GO:0044297&search_constraint=terms) | TC393100, TC382928, CV775873, TC413571, TC417363, TC386237, TC386313, TC393904, TC398052, TC425841, TC387579, TC371242, TC394459, TC384738, CK211707, TC386963, TC388410, TC377410, TC381218, TC395303, TC400755, TC376420, TC458987, TC380433, TC397312, CK211589, TC401210, TC386961, TC390135, TC397729, TC377225, TC399919, TC407572, TC372175, TC396365, TC381462, TC419747, TC401758, TC392263, TC389661, TC382045 |
| [Cell periphery](http://amigo.geneontology.org/cgi-bin/amigo/go.cgi?action=query&view=query&query=GO:0071944&search_constraint=terms) | TC410352, TC404158, TC407978, CV775873, TC374240, TC398052, CA710880, TC425841, TC397994, CK215979, TC387579, TC394459, TC371242, TC384738, TC423252, TC397660, TC404978, TC389363, TC388914, TC388410, TC421914, TC446235, TC387410, TC378271, TC380433, TC420735, TC395453, TC386961, TC390569, TC416685, TC407076, TC403872, TC389090, TC389375, TC382655, CK200433, TC370114, BQ294582, TC406038, CJ550278, TC395351, TC401915, TC393100, DR739994, TC384454, TC386237, TC393904, CK217367, TC428066, TC379853, TC370315, TC395303, TC400755, TC405511, TC395723, TC405440, TC376420, TC458987, TC423110, TC379536, TC398730, TC401210, DR737360, TC390135, TC387135, TC377225, TC409650, BQ607161, TC406807, TC382045, TC382830, DR739471, TC434820, TC443387, CK212850, TC379711, TC378790, TC392272, TC417077, TC386313, TC390402, TC402440, TC369844, TC394307, CK211707, TC370912, TC375146, TC386963, TC373583, TC389589, TC370178, TC392778, TC373637, TC397312, CK211589, TC373678, TC386422, TC388688, TC412150, TC399919, TC407572, TC372175, TC388822, TC377496, TC406708, TC396365, TC419747, TC429771, TC423576, TC416658, TC384678, TC386646, TC393561, TC435799, TC391671, TC393554, TC407614, TC459656, TC382928, TC423354, TC438243, TC413571, CA729339, TC417363, TC394118, TC380882, TC373848, TC423880, TC425690, TC377410, TC381218, TC389816, TC371387, TC435224, CA613620, TC461622, TC437163, TC403588, TC376933, TC385659, TC397729, TC387683, TC396895, TC434831, TC426326, TC381462, TC377749, TC393106, TC401758, TC392263, TC396460, TC389661, TC428944 |
| [Mitochondrion](http://amigo.geneontology.org/cgi-bin/amigo/go.cgi?action=query&view=query&query=GO:0005739&search_constraint=terms) | TC399730, TC410352, TC368580, TC386040, TC373613, TC390393, TC416529, TC390516, TC391946, TC375864, TC420420, TC415588, BQ838511, TC387861, TC391749, CA710880, TC432124, TC452945, TC400388, TC376351, TC371242, TC416154, TC423252, TC383028, CV766916, TC397660, TC407404, BJ282766, TC416493, TC374996, TC388136, TC393781, TC393436, TC435210, TC380063, TC398538, CK214103, TC418414, TC410063, TC421143, TC378054, TC386396, TC373702, TC419273, TC395453, TC461607, TC392329, TC437445, TC407076, TC403872, CA614761, CJ550278, TC395746, TC397444, TC439210, TC415483, TC417012, CA632212, TC435852, TC413027, TC372549, TC378274, TC391130, CK217367, TC378153, TC444410, CK214702, TC426907, TC395723, TC369415, TC387981, TC385233, TC391950, TC389718, TC386707, BE217043, TC402069, TC381943, TC420358, TC398379, TC422142, CV770684, TC413392, TC398209, TC376758, BQ607161, TC372845, TC382045, TC402308, TC441343, TC378432, TC388317, TC389843, CV759879, TC376625, TC391438, TC385714, TC439708, TC412212, TC373563, TC375124, BJ258087, TC405695, TC370178, TC392778, TC397312, TC405030, TC388403, TC416695, TC444549, TC406469, TC410690, TC406708, TC418691, TC374461, TC409459, TC420337, TC443289, TC386646, TC416471, TC419919, TC372761, TC399841, TC437137, CN010359, TC369687, TC402603, TC433557, TC449724, TC395914, TC372867, TC397909, CA729339, TC408299, TC415803, TC454526, TC389993, TC398830, TC399352, TC408312, CA613620, TC371129, TC400362, TC420816, TC401293, TC369325, TC429747, TC384162, TC430501, TC400108, TC383739, TC399342, TC432185, TC455736, TC396451, TC427986, TC429217, TC372677, TC421162, TC374240, TC376306, TC425841, TC389168, TC424204, TC422288, TC393814, TC373402, TC376220, TC446235, TC428008, TC402545, TC427006, TC393830, TC380943, TC403885, TC394451, TC398606, TC395312, CV772029, TC396134, TC418365, TC389090, TC370347, TC389139, TC412483, TC375554, TC373002, TC382655, TC389144, CK200433, TC374731, TC395351, TC392709, TC372563, TC418032, TC388228, TC397291, TC411908, CA598430, TC377373, TC459008, TC380329, TC376930, TC391516, TC389696, TC391143, TC426916, TC381463, TC410066, TC382742, TC395303, TC405511, TC442140, TC386410, TC393890, TC423110, TC390762, TC385365, TC442727, TC430544, DR737360, TC379357, TC387135, TC381007, TC411684, TC388049, TC404850, TC433940, TC393960, DR734732, TC409077, TC387686, TC413124, TC443387, TC388566, TC415083, TC399245, TC390630, TC418716, TC379023, TC386519, TC374726, TC417992, TC394307, TC406371, TC389589, TC398592, TC392363, TC378911, TC452762, TC386401, TC407490, TC386344, TC452356, TC370044, TC421872, TC386422, TC400379, TC377993, TC426078, TC412150, TC393037, TC388822, TC429771, TC383570, TC386639, TC375069, TC416658, TC424962, TC379965, TC456370, TC374230, TC393948, TC403624, TC371970, TC375726, TC393554, TC407614, TC379436, TC460615, TC403573, TC385445, TC403929, TC379635, TC373848, TC426870, TC433589, TC389816, TC372530, TC392323, TC370907, TC384194, TC413066, TC404426, TC371172, TC439472, TC372167, TC461622, TC400056, TC379470, TC419090, TC403588, TC377944, TC381817, TC385659, TC389451, TC394728, TC403803, TC440526, TC428944, CA726837, TC381279, TC380333, TC379942, TC397258, TC396230, TC374404, TC394965, TC384373, TC368599, TC416069, TC427210, TC375313, TC398862, TC433162, TC400317, TC389363, TC433116, TC413263, TC411471, TC416442, TC378072, TC406516, TC388950, TC419222, TC410194, TC448840, TC408228, TC409005, TC371145, TC384735, TC405540, TC390569, TC419057, TC391948, TC451950, TC380695, BQ294582, TC381619, TC424179, TC398970, TC457126, TC403679, TC456854, TC418747, TC386305, TC401915, TC372667, TC433957, TC392432, TC423239, DR739994, TC370962, TC395506, TC395672, TC450972, TC406236, TC391128, TC445767, TC387501, TC446038, TC379853, TC403687, TC418685, TC405440, CK213497, TC460795, TC414899, TC422789, TC440819, TC398514, TC416492, TC391900, TC426980, TC392033, TC412520, TC382858, TC429865, TC416906, TC434820, TC379711, TC377308, TC379903, TC406749, TC423265, TC392272, TC401244, TC417388, TC440066, TC413339, TC389108, TC408907, TC375539, TC385013, TC420579, TC380083, TC412732, TC381191, TC370392, TC370350, TC411116, TC388718, CN010697, TC383701, TC452793, TC385780, CV761628, TC378568, TC395566, TC404420, TC383235, TC369199, TC407304, TC402186, TC385927, TC405356, TC377441, BE415178, TC429062, TC403264, TC423354, TC409344, TC413043, BJ243383, TC400484, CK214224, TC368603, TC445281, TC376263, TC374964, TC375234, TC409187, TC416169, TC425690, TC375253, TC423182, TC409043, TC377290, TC409190, CJ563078, TC403537, TC381255, TC392297, TC388520, TC418399, TC431382, TC434831, TC426326, TC375431, TC424252, TC391995, TC373914, TC450285, TC382024, TC445649, TC369899, TC404998, TC382777, TC435533, TC403936, TC414243, TC403234, TC387050, TC430988, TC388914, TC415056, TC413771, TC387621, TC398633, TC394716, TC460689, TC397027, TC380125, TC369064, TC377292, TC432154, TC420735, TC435281, TC404371, TC402939, TC384219, TC383949, TC378409, TC416685, TC389375, TC404413, TC370114, TC406038, TC390319, TC408192, TC376874, CJ626653, TC369928, TC370727, TC419438, TC449235, TC376490, TC384454, TC411287, TC407395, TC435909, TC447801, TC399471, TC430096, TC427981, TC434396, TC398536, TC421880, CA500690, TC386414, TC389590, CJ854725, TC403717, TC440636, TC372701, TC403986, TC376420, TC382737, TC370633, TC386279, TC411480, TC432320, TC391785, TC439225, TC419727, TC388158, TC395298, TC405773, TC394253, TC389162, DR739471, TC402534, TC399432, TC389678, TC434949, TC382129, TC390285, CA727263, TC387766, TC407340, TC398026, TC406584, TC405295, TC392465, TC402440, TC455676, TC396636, TC375146, TC421704, TC394661, TC389044, TC381963, TC383176, GH728664, TC385701, TC388688, TC403328, TC375268, TC452503, TC418759, TC398978, TC368549, TC377496, TC388976, TC401158, TC387133, TC385526, TC423576, TC402668, TC393561, TC393692, TC391671, TC397415, TC392887, TC370210, TC370507, TC375918, TC417363, TC458699, TC385515, TC380882, TC423880, TC451285, BG263159, TC372580, TC444588, TC458503, TC400721, TC387194, TC434689, TC393820, CK211469, CB307332, TC404842, TC399408, TC417970, TC411817, TC396895, TC436290, TC382342, TC393106, TC390489, TC428932, TC432504, TC397676, TC396460, TC383143, TC379968 |
| [Extracellular space](http://amigo.geneontology.org/cgi-bin/amigo/go.cgi?action=query&view=query&query=GO:0005615&search_constraint=terms) | TC410352, DR739471, TC434820, TC443387, TC441343, TC379711, TC394796, CD902983, TC392272, TC417077, TC397033, CA710880, TC402440, TC371242, TC423252, TC394307, TC397660, TC375146, TC389363, TC388914, TC389589, TC446235, TC392778, TC394661, CK211589, TC420735, TC370044, TC395453, TC386422, TC388688, TC384735, TC390569, TC416685, TC372664, TC407076, TC403872, TC389090, TC389375, TC388822, TC406708, TC377496, TC382655, TC429771, CK200433, TC423576, BQ294582, TC370114, TC409459, TC406038, TC416658, TC395351, TC401915, TC393561, TC391671, TC393554, TC407614, TC459656, DR739994, TC438243, TC384454, TC413571, CA729339, TC391128, CK217367, TC380882, TC379853, TC389816, TC384194, TC405440, CA613620, TC461622, TC398730, TC403588, DR737360, TC388049, TC396895, TC434831, TC426326, TC393106, BQ607161, TC406807, TC396460, TC428944 |
| [Endomembrane system](http://amigo.geneontology.org/cgi-bin/amigo/go.cgi?action=query&view=query&query=GO:0012505&search_constraint=terms) | TC407978, TC378790, TC390630, CV775873, TC386313, TC398052, TC390402, TC425841, TC387579, CA606693, TC394459, TC384738, CK211707, TC386963, TC388410, TC458562, TC396751, TC383909, TC449463, CA615187, TC386961, TC405041, TC388547, TC387344, TC405784, TC399919, TC407572, TC372175, TC396365, TC419747, TC370114, TC409459, TC398714, TC398304, TC433957, TC393100, TC393948, CK210754, TC412569, TC382928, TC441241, TC438243, TC386237, TC417363, TC409343, TC394916, TC387191, TC390994, TC378274, TC403929, TC385515, TC395303, TC422425, TC400755, TC458987, TC435808, TC390135, TC398514, TC387135, TC377225, TC411191, TC409650, TC380662, TC401758, TC389661 |
| [Extracellular region part](http://amigo.geneontology.org/cgi-bin/amigo/go.cgi?action=query&view=query&query=GO:0044421&search_constraint=terms) | TC410352, DR739471, TC434820, TC443387, TC441343, TC379711, TC394796, CD902983, TC392272, TC417077, TC397033, CA710880, TC402440, TC371242, TC423252, TC394307, TC397660, TC375146, TC389363, TC388914, TC389589, TC446235, TC392778, TC394661, CK211589, TC420735, TC370044, TC395453, TC386422, TC388688, TC384735, TC390569, TC416685, TC372664, TC407076, TC403872, TC389090, TC389375, TC388822, TC406708, TC377496, TC382655, TC429771, CK200433, TC423576, BQ294582, TC370114, TC409459, TC406038, TC416658, TC395351, TC401915, TC393561, TC391671, TC393554, TC407614, TC459656, DR739994, TC438243, TC384454, TC413571, CA729339, TC391128, CK217367, TC380882, TC379853, TC389816, TC384194, TC405440, CA613620, TC461622, TC398730, TC403588, DR737360, TC388049, TC396895, TC434831, TC426326, TC393106, BQ607161, TC406807, TC396460, TC428944 |
| [Membrane part](http://amigo.geneontology.org/cgi-bin/amigo/go.cgi?action=query&view=query&query=GO:0044425&search_constraint=terms) | TC410352, TC404158, TC373958, TC394796, CV775873, TC374240, TC398052, CA710880, TC425841, TC398862, TC387579, TC394459, TC371242, TC384738, TC423252, TC397660, TC389363, TC388914, TC388410, BJ282766, TC387621, TC458562, TC411471, TC421914, TC446235, TC387410, TC378271, TC418414, TC380433, TC420735, TC395453, TC386961, TC390569, TC416685, TC387344, TC407076, TC403872, TC418365, TC389090, TC389375, TC382655, CK200433, TC398714, TC370114, BQ294582, TC406038, CJ550278, TC395351, TC403679, TC401915, TC456619, TC398304, TC393100, TC433957, CK210754, TC412569, DR739994, TC397291, TC384454, TC386237, TC394916, TC409343, TC393904, TC390994, TC387191, TC378274, CK217367, TC428066, TC379853, TC370315, TC395303, TC400755, TC395723, TC405440, TC376420, TC458987, TC377190, TC423110, TC379536, TC398730, TC401210, TC390135, TC398514, DR737360, TC387135, TC377225, TC409650, TC380662, TC398379, BQ607161, TC406807, TC382045, DR739471, TC416906, TC434820, TC443387, TC379711, TC400260, TC378790, TC390630, TC392272, TC377061, TC417077, TC417388, TC386313, TC390402, TC402440, CA606693, TC394307, CK211707, TC375146, TC386963, TC389589, TC392778, TC396751, TC397312, CK211589, TC383909, TC449463, CA615187, TC386422, TC388688, TC405041, TC388547, TC405784, TC412150, TC399919, TC407572, TC372175, TC388822, TC396365, TC377496, TC406708, TC419747, TC429771, TC409459, TC423576, TC416658, TC386646, TC393561, TC393948, TC435799, TC386322, TC391671, TC393554, TC407614, TC459656, TC382928, TC441241, TC438243, TC413571, CA729339, TC417363, TC403929, TC385515, TC394118, TC380882, TC425690, TC377410, TC381218, TC389816, TC371387, TC422425, TC384194, TC439472, TC446092, CA613620, TC461622, TC403588, TC397729, TC411191, TC434831, TC432185, TC396895, TC426326, TC381462, TC393106, TC401758, TC392263, TC396460, TC431306, TC428944, TC389661 |
| [Cellular_component](http://amigo.geneontology.org/cgi-bin/amigo/go.cgi?action=query&view=query&query=GO:0005575&search_constraint=terms) | TC461921, BJ284275, TC410352, TC390944, TC370791, TC389190, TC453487, TC375864, TC432212, TC415588, TC389842, BQ838511, TC398052, TC387861, TC391749, TC374098, TC400388, TC457112, TC431932, CK215979, CK206714, TC376351, TC430471, TC439423, TC383028, TC379171, TC388410, BJ282766, CK203550, TC416493, TC388136, TC458983, TC458562, TC393436, TC417260, TC422842, CK214103, TC433844, CK209294, TC410063, TC421143, TC386396, TC378615, TC373702, TC448471, TC456784, TC395453, TC392329, TC410182, TC369255, TC445939, TC425847, CJ727624, CA614761, CJ550278, BE591166, TC411128, GH732878, TC397444, TC412569, TC415483, CA632212, TC393523, TC435852, TC413027, TC372549, TC394820, TC398731, TC418073, TC411784, TC417308, TC387817, TC395069, TC413460, TC411008, BQ609416, CK214702, TC426907, TC395723, TC369415, TC387981, TC385233, TC391950, TC398730, TC394087, TC386707, TC376248, BE217043, TC374409, TC402069, TC381943, TC432001, TC420358, CV770684, TC418928, TC398209, BQ607161, TC372845, TC382045, TC431175, TC402308, TC378790, TC417077, TC385009, CV759879, TC376625, TC390016, TC380474, CA606693, TC370912, TC432369, TC385714, TC412212, TC375124, TC392778, TC397312, TC405030, CK161232, CA615187, TC405041, TC444549, TC397019, BJ317882, TC406469, TC399919, TC407572, TC413118, TC406708, TC373898, TC418691, TC409459, TC420337, TC391360, TC416471, TC419919, CV763657, TC372761, TC406809, TC410569, TC437137, CN010359, TC373001, TC402603, TC449724, TC395914, TC372867, TC377559, TC397909, CA729339, TC383019, TC423348, BQ239045, TC430163, TC389993, TC426864, TC408312, CV766349, TC391156, TC401941, CA613620, TC400362, TC398154, TC401293, TC369325, TC429747, TC375612, TC430501, CJ867263, TC400108, TC406106, TC381462, CD882425, TC377749, TC396451, TC431198, TC424344, TC404158, CK201269, TC416381, TC429217, TC372677, CJ868963, TC374240, TC376306, TC425841, TC405982, TC387579, TC420460, TC397779, TC428008, TC427006, TC380943, TC376202, TC403885, TC405946, TC460907, TC394451, TC395312, CV772029, TC370603, TC384688, TC387344, TC389090, TC389139, TC412483, TC382655, TC389144, TC456230, TC373994, TC395351, TC392709, TC372563, TC418032, CK162413, TC397291, TC461977, TC430555, TC411908, CA598430, TC459008, TC380329, TC376930, TC391516, TC409343, TC389696, TC403852, TC428066, TC419733, TC392074, TC382742, TC377190, TC443814, TC431879, TC423110, TC422348, TC391613, TC385365, TC430544, TC382339, TC379357, TC371003, TC387135, TC390566, TC378437, TC438587, TC375760, TC381923, TC426016, BJ309186, DR734732, TC387686, TC373251, TC413124, TC388566, TC378225, TC399245, TC373429, GH729256, TC418716, TC392447, TC421467, TC379023, TC386519, TC394219, TC383047, TC402072, TC401260, TC408381, TC417992, TC385090, TC407964, TC394307, TC388665, TC373583, TC406371, TC382139, TC392363, TC381988, TC370106, TC435034, TC388819, TC405475, TC386401, TC422022, TC386344, TC452356, TC373637, TC370044, TC421872, TC386422, TC442623, CJ776780, TC422583, TC384071, TC400379, TC414606, TC393037, CJ730226, TC384592, TC421733, TC383570, TC396228, TC379965, TC396650, TC384678, TC456370, TC404053, TC400562, TC371970, TC376523, TC375726, TC391461, TC459656, DR734904, TC378783, TC379436, TC377765, TC440632, TC403929, TC457991, TC394118, TC378601, TC373848, TC426870, TC437918, TC387007, TC375935, TC418091, TC389816, TC371387, TC372530, TC392323, TC370907, TC384194, TC413066, TC371172, TC390211, TC446092, TC372167, TC391641, TC461622, TC393426, TC400056, TC405033, TC379470, TC419090, TC385659, TC376933, TC382910, TC432678, TC389451, TC458892, DR740372, TC394693, TC416495, TC401758, TC428944, TC417067, TC449093, TC379942, TC434821, TC374404, BQ295499, TC396230, TC377766, TC398383, TC394965, TC368599, TC391648, TC376937, TC433162, TC400317, TC404978, TC389363, TC371248, TC379338, TC433116, TC409167, TC416442, TC374679, TC387410, TC378072, TC397176, TC406516, TC406986, TC371145, TC435546, TC419057, TC451950, TC380695, TC398714, TC381619, TC424179, TC394028, TC398970, TC457126, TC456854, TC403679, TC418747, TC440803, TC403580, TC413427, TC386305, TC424044, TC423374, TC372667, TC433957, TC386440, TC392432, TC423239, TC425821, TC370962, TC403055, TC394206, TC406236, TC382080, TC391962, TC391128, TC445767, TC387501, TC446038, TC374852, TC369830, TC423804, TC413199, TC418685, CK213497, TC414899, TC436309, TC398514, TC391900, TC392033, TC423091, TC447694, TC380825, TC392269, TC412520, TC406807, TC395090, TC404606, TC441239, TC416906, TC416438, CA595837, TC423265, TC372744, TC417388, TC440066, TC386313, CK208222, TC404179, TC435055, TC389108, TC369844, TC420579, CK211707, TC392126, TC402340, TC431201, CK217689, TC370392, CV761547, TC396751, CK211589, TC411116, TC388718, TC383701, TC452793, TC385780, TC405784, TC395566, TC372175, TC382734, TC405896, TC385927, TC369072, TC385513, TC439501, TC405356, TC444586, TC449448, TC377441, TC381596, TC403264, TC369348, TC461971, TC435825, TC422176, TC388232, TC445281, TC376263, TC415521, TC384010, TC416169, TC409043, TC408229, TC377290, TC409190, TC383677, TC403537, TC381255, CV781430, TC379924, TC418399, TC431382, TC387683, TC409855, TC434831, TC406194, TC427161, TC373914, TC450285, TC392303, TC382024, TC404998, TC369899, TC378846, TC372330, TC448051, TC397286, TC381068, CD930656, TC403936, CF554444, TC451276, TC444375, TC387050, TC415685, TC388914, TC415056, TC433488, TC377178, TC377438, TC394716, TC397027, TC456160, TC394670, TC378271, TC377292, TC380433, TC451621, TC435281, TC384219, TC397562, TC428853, TC404413, TC408192, CJ626653, TC372046, CK210754, TC407191, TC370727, TC417769, TC449235, TC410147, TC442100, TC414564, TC405910, TC431603, TC393904, TC387191, TC407395, TC447801, TC380416, TC427981, CA500690, TC398536, TC400755, TC403717, TC372701, TC405615, TC376420, TC458987, TC422851, TC404843, TC382737, TC370633, TC390135, TC376774, TC411480, TC417755, TC420394, TC432320, TC387170, TC439225, TC380662, TC388649, TC419727, TC413462, TC394263, TC436772, TC395298, TC394253, TC412094, CV782550, TC389162, TC376630, CK212850, TC389678, TC399432, TC434006, TC434949, TC369726, CA727263, TC379241, TC405295, TC417480, TC392465, TC455676, TC373962, TC391411, TC409208, TC375813, TC379343, TC374741, TC429809, TC386591, TC421704, TC420201, TC381963, TC430821, TC456247, TC371455, TC374879, GH728664, TC385701, TC379459, TC400181, TC418759, TC431825, TC377496, TC368549, TC401158, TC387133, TC410078, TC415942, TC393561, TC393692, TC386322, TC391671, TC373787, BQ160837, TC382928, TC397415, TC370507, TC369637, TC396487, TC436832, TC458699, TC380882, TC440321, TC372580, TC444588, TC377410, TC400721, TC451657, TC445214, TC451519, TC393820, TC435808, TC449739, TC416981, BE586004, TC397729, TC399408, TC417970, TC426743, TC396895, TC382342, TC393106, TC428932, TC407183, TC431306, TC396460, TC397676, TC379968, TC399730, TC368580, TC407456, TC441536, TC373613, TC386040, TC407978, TC390393, TC373958, TC444546, TC411284, TC392283, TC390516, TC416529, TC391946, TC460760, TC420420, TC383139, TC408524, TC417106, TC419614, TC432124, CA710880, TC452945, TC375593, TC421871, TC371242, TC394459, TC423252, TC384738, TC416154, TC397660, CV766916, TC407404, TC390379, TC374996, TC393781, TC421914, TC435210, TC380063, TC398538, TC418414, CJ944525, TC396772, TC378054, TC453207, TC410954, TC419273, TC461607, TC409843, TC406594, TC437445, TC403872, TC407076, TC400595, TC371357, TC406207, TC389586, TC395746, TC439210, TC398304, TC393100, TC425878, CV771134, BE431108, TC417012, TC391447, TC455515, TC394916, TC397793, TC404914, TC391130, TC378274, CK217367, TC378153, TC379990, TC410144, TC378333, TC444410, TC435863, BQ788843, TC452050, CJ845228, TC379536, TC391621, TC404052, TC389718, TC429713, CK201148, TC386088, TC389534, TC413392, TC422142, TC398379, TC385783, TC376758, CJ815867, TC369628, TC382950, TC441343, TC400260, TC400330, TC372796, TC399057, TC408191, TC378432, TC377061, TC389843, TC388317, CD937281, TC446465, TC416801, AL827131, BF474051, TC379312, TC391438, TC381268, TC412317, TC371325, TC439708, TC428113, TC373563, TC370619, BJ258087, TC421345, TC422007, TC370178, TC405695, TC388403, TC373678, TC416695, TC402121, TC410690, TC374461, TC410074, TC443289, TC386646, TC399841, TC369687, TC458205, TC441241, TC433557, TC413571, TC415803, TC408299, TC410108, TC454526, TC398830, TC399352, TC422425, TC371129, TC398805, TC372654, TC428992, TC369092, TC420816, TC384162, TC395872, TC383739, TC399342, TC432185, TC381583, TC455736, TC392263, TC432130, TC375665, TC385205, TC373145, TC384625, TC427986, TC422645, CD902983, TC425957, TC445166, BG904091, TC421162, TC429341, TC397033, TC398121, TC389168, TC424204, TC422288, TC439324, TC413854, TC406870, TC424317, TC393814, BJ279521, TC376220, TC373402, TC408309, TC404926, TC446235, TC402545, TC393830, TC415808, TC444899, TC386961, TC398606, TC387319, TC396134, TC369182, TC418365, TC373204, TC370347, TC373002, TC375554, CK200433, TC393970, TC374731, TC452494, TC456619, TC388228, TC444138, TC377373, TC415365, EB512907, TC390994, TC412205, TC393437, TC447260, TC426916, TC391143, TC381463, TC403157, TC410066, TC414090, TC395303, TC442140, TC405511, TC386410, TC392203, TC393890, TC442727, TC390762, TC401210, DR737360, TC381007, TC397885, TC411684, TC382728, TC388049, TC404850, TC370033, TC433940, TC374485, TC390436, TC393960, TC426358, TC385326, TC449064, TC387707, TC409077, TC443387, TC415083, TC370470, TC386535, TC390630, TC390402, TC374726, TC417341, TC415954, TC376790, TC398592, TC389589, TC378911, TC452762, TC407490, CD878039, TC374164, TC418845, TC377993, TC438657, DR735108, TC412150, TC426078, TC388822, TC429771, TC386639, TC375069, TC416658, TC424962, TC379944, TC374230, TC391913, TC375834, TC393948, TC403624, TC400638, TC393554, TC407614, TC400477, TC384049, TC384357, TC391007, TC460615, TC403573, TC411941, TC385445, TC395841, TC449043, TC379635, TC392247, TC387116, TC373259, TC433589, TC382338, TC375914, TC398343, TC377253, TC404426, TC439472, TC377944, TC381817, TC403588, TC375798, TC394728, TC403803, TC391122, TC418850, TC440526, TC436944, TC371341, TC389661, TC381279, CA726837, TC387386, TC383820, TC396657, TC380333, TC377064, TC397258, TC426838, CV775873, TC451511, TC431330, TC384373, TC416069, TC427210, TC422606, TC399271, TC375313, TC398862, DR739303, TC372450, TC381613, TC434442, TC413263, TC380590, TC411471, TC431660, TC434570, TC378878, TC406264, TC390792, TC394020, TC388950, TC374669, TC419222, TC410194, TC369371, TC448840, TC392875, TC409005, TC408228, TC384735, TC439904, TC418728, TC405540, TC390569, TC391948, BQ294582, TC401915, CA724903, TC421954, TC405652, TC385710, CV779166, DR739994, TC459193, TC395506, TC395672, TC377021, TC450972, TC392358, TC409599, TC419165, TC404636, TC389092, TC376800, TC379853, TC420248, CK199846, TC370315, TC449504, TC403687, TC405440, TC403968, TC460795, CJ792862, TC422789, TC370885, TC407232, TC381561, TC391742, TC440819, TC416492, TC393015, TC426980, TC377225, TC434000, TC404620, TC382858, TC403977, CA646741, TC429865, TC434820, TC379711, TC393198, TC379903, TC377308, TC406749, TC392272, TC401244, CA730421, DR739350, TC400114, TC413339, TC408907, TC375539, TC412551, TC385013, TC380083, TC386963, TC412732, TC381191, TC369010, TC402080, TC373518, TC370350, TC383909, TC376527, TC374940, TC419584, CN010697, TC374392, TC378568, TC369983, CV761628, TC404420, TC383235, TC390150, TC373489, TC369199, TC419747, TC407304, CA676115, TC402186, BE415178, TC429062, TC423354, TC409344, TC413043, BJ243383, TC400484, CK214224, TC368603, TC384939, TC398040, TC374964, TC375234, CA709177, TC385815, TC409187, TC429374, TC425690, TC375253, TC423182, TC381218, TC401124, TC375873, TC444302, TC435224, CJ563078, TC394850, TC384344, TC439154, TC387511, TC455532, TC392297, TC427600, TC388520, TC443712, TC379422, TC378956, TC411191, TC426326, TC375431, TC424252, TC391995, TC415501, TC422841, TC387064, TC445649, CA498496, TC394796, TC382777, TC440499, TC435533, TC407349, TC397994, TC414243, TC403234, TC429437, TC430988, TC371738, TC413771, TC387621, TC398633, TC368548, TC460689, TC369633, TC380125, TC410126, TC369064, TC432154, TC420735, TC422922, TC404371, TC402939, TC427405, TC449256, TC373615, TC383949, TC378409, TC416685, TC451342, TC372664, TC399986, TC389375, TC433812, TC370114, TC406038, TC390319, TC376874, TC424154, TC458807, TC459245, TC429871, TC369928, TC419438, TC376490, TC384454, TC411287, TC386237, TC388751, TC431722, AL820497, TC435909, TC399471, TC408126, TC430096, TC399972, TC434396, TC421880, TC386414, TC389590, CJ854725, TC440636, TC403986, TC406193, TC383763, TC384553, CD876572, TC369736, TC386279, CK197833, TC391785, TC377427, TC382786, TC409650, TC388158, TC430561, TC418340, TC405773, TC382830, TC377653, CD905784, DR739471, TC402534, TC371600, TC382129, TC391840, TC390285, TC412989, TC387766, TC407340, TC398026, TC406584, CV065343, TC402440, TC396636, TC432205, TC375146, TC454407, CK199175, TC384122, TC370037, TC394661, TC389044, TC383176, TC449463, TC371037, TC388688, TC403328, TC408095, TC439939, TC375268, TC452503, TC388547, TC420877, TC429048, TC398978, TC388976, TC396365, TC379069, TC385526, TC423576, TC375530, TC377635, TC397500, TC423010, TC402668, TC435799, TC388691, TC438243, TC392887, TC370210, TC375918, TC417363, TC400592, TC387710, TC413700, TC385515, TC423880, TC451285, BG263159, TC458503, TC387194, TC391865, TC434689, TC441995, CK211469, TC437163, CB307332, TC404842, TC381296, TC444402, TC411817, TC436290, TC390489, TC421341, TC432504, TC383143 |
| [Fungal-type vacuole membrane](http://amigo.geneontology.org/cgi-bin/amigo/go.cgi?action=query&view=query&query=GO:0000329&search_constraint=terms) | TC405511, TC423354, TC403588, TC380882, TC423880 |
| [Macromolecular complex](http://amigo.geneontology.org/cgi-bin/amigo/go.cgi?action=query&view=query&query=GO:0032991&search_constraint=terms) | TC461921, TC410352, TC386040, TC379942, TC407978, TC390393, TC444546, TC416529, TC397258, CV775873, TC396230, TC420420, TC460760, TC451511, TC427210, TC416069, BQ838511, TC398052, TC387861, CA710880, TC400388, TC398862, TC433162, TC394459, TC371242, TC416154, TC384738, TC423252, TC397660, TC389363, TC379171, TC388410, BJ282766, CK203550, TC416493, TC458562, TC416442, TC411471, TC421914, TC406264, TC387410, TC380063, TC398538, TC397176, TC418414, CJ944525, TC410063, TC378054, TC448471, TC395453, TC410194, TC392875, TC435546, TC384735, TC390569, TC405540, TC407076, TC419057, TC403872, TC425847, TC391948, CJ727624, TC398714, BQ294582, CJ550278, TC381619, TC398970, TC457126, TC403580, TC413427, TC411128, TC401915, TC397444, TC398304, TC425878, TC393100, TC433957, TC412569, TC417012, CA632212, DR739994, TC459193, TC413027, TC394916, TC394206, TC394820, TC406236, TC418073, TC378274, TC391130, TC417308, CK217367, TC391128, TC409599, TC445767, TC446038, TC423804, TC413460, TC379853, TC370315, BQ609416, CK214702, TC449504, TC395723, TC405440, CK213497, CJ792862, TC379536, TC385233, TC398730, TC389718, TC404052, TC398514, TC377225, TC381943, TC412520, TC432001, TC398379, TC413392, BQ607161, TC406807, TC372845, TC382045, TC404606, CA646741, TC369628, TC434820, TC416906, TC402308, TC441343, TC379711, TC400330, TC400260, TC416438, CA595837, TC379903, TC423265, TC392272, TC377061, TC401244, CA730421, TC417077, TC417388, TC446465, TC440066, TC386313, CV759879, TC408907, CA606693, TC391438, CK211707, TC386963, TC412732, TC431201, TC405695, TC392778, TC396751, TC397312, TC405030, CK211589, TC388403, TC383909, TC376527, CA615187, TC416695, TC388718, TC383701, TC406469, TC385780, TC374392, TC405784, TC399919, TC407572, TC372175, TC410690, TC406708, TC419747, TC409459, TC410074, TC402186, TC405356, CV763657, TC423354, TC402603, TC403264, TC441241, TC449724, TC413571, TC413043, CA729339, CK214224, TC408299, BQ239045, TC368603, CA709177, TC376263, TC416169, TC425690, TC423182, TC381218, TC399352, TC408229, TC422425, TC408312, CA613620, TC398805, TC400362, TC369092, CV781430, TC388520, TC379422, TC411191, TC400108, TC399342, TC434831, TC426326, TC381462, TC375431, CD882425, TC415501, TC396451, TC373914, TC392263, TC450285, TC392303, TC394796, TC425957, TC445166, TC435533, TC374240, TC425841, TC389168, TC398121, CF554444, TC424204, TC387579, TC406870, TC415685, TC388914, TC393814, TC376220, TC387621, TC398633, TC394716, TC446235, TC410126, TC402545, TC378271, TC380943, TC393830, TC380433, TC403885, TC420735, TC435281, TC404371, TC386961, TC384219, TC398606, TC397562, TC416685, TC387344, TC372664, TC389090, TC412483, TC389375, TC404413, TC382655, CK200433, TC456230, TC370114, TC373994, TC406038, TC376874, TC395351, TC418032, TC456619, CK210754, TC369928, TC417769, TC410147, TC384454, TC397291, TC411287, CA598430, TC386237, EB512907, TC409343, TC393904, TC388751, TC390994, TC391143, TC399471, TC381463, TC403157, TC410066, TC395303, TC386414, TC400755, TC403986, TC440636, TC405511, TC376420, TC458987, TC377190, TC443814, TC431879, TC404843, TC423110, CD876572, TC385365, TC382737, TC401210, DR737360, TC379357, TC390135, TC387135, TC386279, TC397885, TC411684, TC411480, TC391785, TC409650, TC380662, TC388649, TC393960, TC395298, TC389162, DR739471, TC402534, TC443387, TC388566, TC415083, TC373429, TC390630, TC386535, GH729256, TC369726, TC407340, TC406584, TC386519, TC390402, TC402440, TC391411, TC417992, TC417341, TC394307, TC409208, TC375146, CK199175, TC389589, TC381988, TC452762, TC386344, TC373637, TC381963, TC383176, TC370044, CD878039, TC449463, TC371037, TC386422, TC374164, TC403328, TC388688, TC418845, TC414606, TC388547, TC388822, TC396365, TC377496, TC388976, TC429771, TC410078, TC423576, TC386639, TC416658, TC374230, TC393561, TC375834, TC435799, TC393948, TC393692, TC371970, TC391671, TC393554, TC407614, TC459656, TC382928, TC438243, TC379436, TC417363, TC460615, TC403573, TC387710, TC413700, TC403929, TC385515, TC449043, TC380882, TC423880, TC440321, TC379635, TC373259, TC433589, TC377410, TC418091, TC389816, TC371387, TC392323, TC413066, TC439472, TC391641, TC393820, CK211469, CB307332, TC404842, TC381817, TC403588, TC397729, TC403803, TC396895, TC418850, TC382342, TC393106, TC390489, TC401758, TC432504, TC397676, TC396460, TC431306, TC428944, TC389661, TC417067 |
| [Cell projection](http://amigo.geneontology.org/cgi-bin/amigo/go.cgi?action=query&view=query&query=GO:0042995&search_constraint=terms) | TC407456, TC393948, TC393100, TC390630, TC382928, CV775873, TC413571, TC417363, TC386237, TC374240, TC386313, TC393904, TC398052, TC435055, TC425841, TC387579, TC380882, TC371242, TC394459, TC384738, CK211707, TC386963, TC388410, TC377410, TC381218, TC395303, TC400755, TC421914, TC395723, TC387410, TC378271, TC458987, TC461622, TC397312, TC380433, CK211589, TC403588, TC401210, TC386961, TC390135, TC397729, TC377225, TC399919, TC407572, TC372175, TC381462, TC396365, TC419747, TC401758, TC392263, CJ550278, TC393970, TC389661, TC382045 |
| [DNA replication factor C complex](http://amigo.geneontology.org/cgi-bin/amigo/go.cgi?action=query&view=query&query=GO:0005663&search_constraint=terms) | TC389162, TC377061, TC369092, TC374164 |
| [Lytic vacuole](http://amigo.geneontology.org/cgi-bin/amigo/go.cgi?action=query&view=query&query=GO:0000323&search_constraint=terms) | TC372530, TC405511, TC373613, TC432185, TC380882, TC423880, TC423354, TC403588 |
| [Fungal-type vacuole](http://amigo.geneontology.org/cgi-bin/amigo/go.cgi?action=query&view=query&query=GO:0000324&search_constraint=terms) | TC405511, TC423354, TC403588, TC380882, TC423880 |
| [Cyclin-dependent protein kinase holoenzyme complex](http://amigo.geneontology.org/cgi-bin/amigo/go.cgi?action=query&view=query&query=GO:0000307&search_constraint=terms) | TC374230, TC377061, TC407340, TC382045 |
| [Intracellular organelle part](http://amigo.geneontology.org/cgi-bin/amigo/go.cgi?action=query&view=query&query=GO:0044446&search_constraint=terms) | TC461921, TC410352, TC386040, TC373613, TC407978, TC390393, TC444546, TC416529, TC397258, CV775873, TC396230, TC460760, TC451511, TC427210, TC416069, BQ838511, TC398052, TC387861, CA710880, TC400388, TC398862, TC433162, TC394459, TC371242, TC416154, TC384738, TC423252, TC397660, TC389363, TC379171, TC388410, BJ282766, CK203550, TC416493, TC458562, TC416442, TC411471, TC421914, TC406264, TC387410, TC380063, TC398538, TC397176, TC418414, TC406516, CJ944525, TC410063, TC378054, TC386396, TC448471, TC395453, TC410194, TC392875, TC435546, TC384735, TC390569, TC405540, TC407076, TC419057, TC403872, TC425847, CJ727624, TC398714, BQ294582, CJ550278, TC398970, TC457126, TC403580, TC413427, TC411128, TC401915, TC397444, TC398304, TC425878, TC393100, TC433957, TC412569, TC417012, CA632212, DR739994, TC459193, TC394916, TC394206, TC394820, TC406236, TC418073, TC378274, TC391130, TC411784, TC417308, CK217367, TC391128, TC409599, TC445767, TC446038, TC423804, TC413460, TC379853, TC370315, BQ609416, CK214702, TC449504, TC395723, TC405440, CK213497, CJ792862, TC379536, TC385233, TC389718, TC404052, TC398514, TC377225, TC381943, TC412520, TC432001, TC398379, TC413392, BQ607161, TC406807, TC382045, TC404606, TC369628, TC434820, TC416906, TC402308, TC441343, TC379711, TC400330, TC400260, TC378790, CA595837, TC379903, TC423265, TC392272, TC377061, TC401244, CA730421, TC417077, TC417388, TC446465, TC440066, TC386313, CV759879, TC408907, CA606693, TC391438, CK211707, TC386963, TC412732, TC431201, TC405695, TC392778, TC396751, TC397312, TC405030, CK211589, TC388403, TC383909, TC376527, CA615187, TC416695, TC388718, TC405041, TC383701, TC406469, TC385780, TC405784, TC399919, TC407572, TC372175, TC410690, TC406708, TC419747, TC409459, TC410074, TC402186, TC405356, CV763657, TC373001, TC423354, TC402603, TC403264, TC441241, TC449724, TC413571, TC413043, CA729339, CK214224, TC408299, BQ239045, TC368603, CA709177, TC376263, TC416169, TC425690, TC381218, TC399352, TC408229, TC422425, CA613620, TC398805, TC400362, TC369092, CV781430, TC388520, TC411191, TC399342, TC434831, TC432185, TC426326, TC381462, TC375431, CD882425, TC415501, TC396451, TC373914, TC392263, TC450285, TC392303, TC394796, TC445166, TC372677, TC435533, TC374240, TC425841, TC389168, CF554444, TC424204, TC387579, TC406870, TC388914, TC393814, TC376220, TC387621, TC398633, TC394716, TC446235, TC410126, TC402545, TC378271, TC380943, TC393830, TC380433, TC432154, TC403885, TC420735, TC404371, TC386961, TC384219, TC398606, TC416685, TC387344, TC372664, TC389090, TC412483, TC389375, TC404413, TC373002, TC382655, CK200433, TC456230, TC370114, TC373994, TC406038, TC393970, TC376874, TC395351, TC418032, TC456619, CK210754, TC417769, TC410147, TC376490, TC384454, TC397291, TC411287, CA598430, TC386237, EB512907, TC409343, TC393904, TC390994, TC387191, TC391143, TC381463, TC403157, TC395303, TC386414, TC400755, TC403986, TC440636, TC405511, TC458987, TC377190, TC431879, TC404843, TC423110, TC391613, CD876572, TC385365, TC401210, TC369736, DR737360, TC379357, TC390135, TC387135, TC386279, TC397885, TC411684, TC411480, TC388049, TC391785, TC409650, TC380662, TC393960, TC389162, DR739471, TC402534, TC443387, TC388566, TC415083, TC390630, TC386535, GH729256, TC418716, TC407340, TC406584, TC386519, TC390402, TC402440, TC391411, TC417992, TC417341, TC394307, TC409208, TC375146, CK199175, TC389589, TC381988, TC452762, TC405475, TC386344, TC373637, TC381963, TC383176, TC370044, CD878039, TC449463, TC374879, TC386422, TC374164, TC403328, TC388688, TC418845, TC452503, TC414606, TC388547, TC388822, TC396365, TC377496, TC388976, TC429771, TC410078, TC423576, TC386639, TC416658, TC374230, TC393561, TC393948, TC435799, TC393692, TC371970, TC391671, TC393554, TC407614, TC459656, TC382928, TC438243, TC379436, TC375918, TC417363, TC460615, TC403573, TC387710, TC413700, TC403929, TC385515, TC394118, TC449043, TC380882, TC423880, TC440321, TC379635, TC387007, TC373259, TC433589, TC377410, TC398343, TC418091, TC389816, TC392323, TC413066, TC439472, TC391641, TC393820, CK211469, CB307332, TC404842, TC435808, TC381817, TC403588, TC397729, TC403803, TC396895, TC418850, TC382342, TC393106, TC401758, TC432504, TC397676, TC396460, TC431306, TC428944, TC389661, TC417067 |
| [Chaperonin-containing T-complex](http://amigo.geneontology.org/cgi-bin/amigo/go.cgi?action=query&view=query&query=GO:0005832&search_constraint=terms) | TC405511, TC423354, TC403588, TC380882, TC423880 |
| [Organelle part](http://amigo.geneontology.org/cgi-bin/amigo/go.cgi?action=query&view=query&query=GO:0044422&search_constraint=terms) | TC461921, TC410352, TC386040, TC373613, TC407978, TC390393, TC444546, TC416529, TC397258, CV775873, TC396230, TC460760, TC451511, TC427210, TC416069, BQ838511, TC398052, TC387861, CA710880, TC400388, TC398862, TC433162, TC394459, TC371242, TC416154, TC384738, TC423252, TC397660, TC389363, TC379171, TC388410, BJ282766, CK203550, TC416493, TC458562, TC416442, TC411471, TC421914, TC406264, TC387410, TC380063, TC398538, TC397176, TC418414, TC406516, CJ944525, TC410063, TC378054, TC386396, TC448471, TC395453, TC410194, TC392875, TC435546, TC384735, TC390569, TC405540, TC407076, TC419057, TC403872, TC425847, CJ727624, TC398714, BQ294582, CJ550278, TC398970, TC457126, TC403580, TC413427, TC411128, TC401915, TC397444, TC398304, TC425878, TC393100, TC433957, TC412569, TC417012, CA632212, DR739994, TC459193, TC394916, TC394206, TC394820, TC406236, TC418073, TC378274, TC391130, TC411784, TC417308, CK217367, TC391128, TC409599, TC445767, TC446038, TC423804, TC413460, TC379853, TC370315, BQ609416, CK214702, TC449504, TC395723, TC405440, CK213497, CJ792862, TC379536, TC385233, TC389718, TC404052, TC398514, TC377225, TC381943, TC412520, TC432001, TC398379, TC413392, BQ607161, TC406807, TC382045, TC404606, TC369628, TC434820, TC416906, TC402308, TC441343, TC379711, TC400330, TC400260, TC378790, CA595837, TC379903, TC423265, TC392272, TC377061, TC401244, CA730421, TC417077, TC417388, TC446465, TC440066, TC386313, CV759879, TC408907, CA606693, TC391438, CK211707, TC386963, TC412732, TC431201, TC405695, TC392778, TC396751, TC397312, TC405030, CK211589, TC388403, TC383909, TC376527, CA615187, TC416695, TC388718, TC405041, TC383701, TC406469, TC385780, TC405784, TC399919, TC407572, TC372175, TC410690, TC406708, TC419747, TC409459, TC410074, TC402186, TC405356, CV763657, TC373001, TC423354, TC402603, TC403264, TC441241, TC449724, TC413571, TC413043, CA729339, CK214224, TC408299, BQ239045, TC368603, CA709177, TC376263, TC416169, TC425690, TC381218, TC399352, TC408229, TC422425, CA613620, TC398805, TC400362, TC369092, CV781430, TC388520, TC411191, TC399342, TC434831, TC432185, TC426326, TC381462, TC375431, CD882425, TC415501, TC396451, TC373914, TC392263, TC450285, TC392303, TC394796, TC445166, TC372677, TC435533, TC374240, TC425841, TC389168, CF554444, TC424204, TC387579, TC406870, TC388914, TC393814, TC376220, TC387621, TC398633, TC394716, TC446235, TC410126, TC402545, TC378271, TC380943, TC393830, TC380433, TC432154, TC403885, TC420735, TC404371, TC386961, TC384219, TC398606, TC416685, TC387344, TC372664, TC389090, TC412483, TC389375, TC404413, TC373002, TC382655, CK200433, TC456230, TC370114, TC373994, TC406038, TC393970, TC376874, TC395351, TC418032, TC456619, CK210754, TC417769, TC410147, TC376490, TC384454, TC397291, TC411287, CA598430, TC386237, EB512907, TC409343, TC393904, TC390994, TC387191, TC391143, TC381463, TC403157, TC395303, TC386414, TC400755, TC403986, TC440636, TC405511, TC458987, TC377190, TC431879, TC404843, TC423110, TC391613, CD876572, TC385365, TC401210, TC369736, DR737360, TC379357, TC390135, TC387135, TC386279, TC397885, TC411684, TC411480, TC388049, TC391785, TC409650, TC380662, TC393960, TC389162, DR739471, TC402534, TC443387, TC388566, TC415083, TC390630, TC386535, GH729256, TC418716, TC407340, TC406584, TC386519, TC390402, TC402440, TC391411, TC417992, TC417341, TC394307, TC409208, TC375146, CK199175, TC389589, TC381988, TC452762, TC405475, TC386344, TC373637, TC381963, TC383176, TC370044, CD878039, TC449463, TC374879, TC386422, TC374164, TC403328, TC388688, TC418845, TC452503, TC414606, TC388547, TC388822, TC396365, TC377496, TC388976, TC429771, TC410078, TC423576, TC386639, TC416658, TC374230, TC393561, TC393948, TC435799, TC393692, TC371970, TC391671, TC393554, TC407614, TC459656, TC382928, TC438243, TC379436, TC375918, TC417363, TC460615, TC403573, TC387710, TC413700, TC403929, TC385515, TC394118, TC449043, TC380882, TC423880, TC440321, TC379635, TC387007, TC373259, TC433589, TC377410, TC398343, TC418091, TC389816, TC392323, TC413066, TC439472, TC391641, TC393820, CK211469, CB307332, TC404842, TC435808, TC381817, TC403588, TC397729, TC403803, TC396895, TC418850, TC382342, TC393106, TC401758, TC432504, TC397676, TC396460, TC431306, TC428944, TC389661, TC417067 |
| [Muscle myosin complex](http://amigo.geneontology.org/cgi-bin/amigo/go.cgi?action=query&view=query&query=GO:0005859&search_constraint=terms) | TC459656, TC406807, TC410126, CJ944525 |
| [Striated muscle myosin Thick filament](http://amigo.geneontology.org/cgi-bin/amigo/go.cgi?action=query&view=query&query=GO:0005863&search_constraint=terms) | TC459656, TC406807, TC410126, CJ944525 |
| [Myosin filament](http://amigo.geneontology.org/cgi-bin/amigo/go.cgi?action=query&view=query&query=GO:0032982&search_constraint=terms) | TC459656, TC406807, TC410126, CJ944525 |
| [Fatty acid synthase complex](http://amigo.geneontology.org/cgi-bin/amigo/go.cgi?action=query&view=query&query=GO:0005835&search_constraint=terms) | TC371037, TC398121 |
| [Myosin II complex](http://amigo.geneontology.org/cgi-bin/amigo/go.cgi?action=query&view=query&query=GO:0016460&search_constraint=terms) | TC459656, TC406807, TC410126, CJ944525 |
| [Membrane-bounded organelle](http://amigo.geneontology.org/cgi-bin/amigo/go.cgi?action=query&view=query&query=GO:0043227&search_constraint=terms) | TC461921, BJ284275, TC410352, TC390944, TC370791, TC389190, TC453487, TC375864, TC432212, TC415588, TC389842, BQ838511, TC398052, TC387861, TC391749, TC374098, TC400388, TC457112, TC431932, CK215979, TC376351, TC430471, TC439423, TC383028, TC379171, TC388410, BJ282766, CK203550, TC416493, TC388136, TC458562, TC393436, TC417260, CK214103, TC433844, CK209294, TC410063, TC421143, TC386396, TC378615, TC373702, TC448471, TC456784, TC395453, TC392329, TC410182, TC445939, TC425847, CJ727624, CA614761, CJ550278, BE591166, TC411128, GH732878, TC397444, TC412569, TC415483, CA632212, TC393523, TC435852, TC413027, TC372549, TC394820, TC398731, TC418073, TC411784, TC417308, TC387817, TC395069, TC413460, TC411008, BQ609416, CK214702, TC426907, TC395723, TC369415, TC387981, TC385233, TC391950, TC398730, TC386707, TC376248, BE217043, TC374409, TC402069, TC381943, TC432001, TC420358, CV770684, TC418928, TC398209, BQ607161, TC372845, TC382045, TC431175, TC402308, TC378790, TC417077, TC385009, CV759879, TC376625, TC390016, TC380474, CA606693, TC370912, TC432369, TC385714, TC412212, TC375124, TC392778, TC397312, TC405030, CA615187, TC405041, TC444549, BJ317882, TC406469, TC399919, TC407572, TC413118, TC406708, TC373898, TC418691, TC409459, TC420337, TC391360, TC416471, TC419919, CV763657, TC372761, TC406809, TC410569, TC437137, CN010359, TC373001, TC402603, TC449724, TC395914, TC372867, TC397909, CA729339, BQ239045, TC430163, TC389993, TC426864, TC408312, TC391156, CA613620, TC400362, TC398154, TC401293, TC369325, TC429747, TC375612, TC430501, CJ867263, TC400108, TC406106, TC381462, CD882425, TC377749, TC396451, TC431198, TC424344, TC429217, TC372677, CJ868963, TC374240, TC376306, TC425841, TC405982, TC387579, TC420460, TC428008, TC427006, TC380943, TC376202, TC403885, TC405946, TC460907, TC394451, TC395312, CV772029, TC387344, TC389090, TC389139, TC412483, TC382655, TC389144, TC456230, TC373994, TC395351, TC392709, TC372563, TC418032, CK162413, TC397291, TC461977, TC411908, CA598430, TC459008, TC380329, TC376930, TC391516, TC409343, TC389696, TC403852, TC382742, TC377190, TC443814, TC431879, TC423110, TC422348, TC391613, TC385365, TC430544, TC382339, TC379357, TC371003, TC387135, TC378437, TC438587, TC375760, TC381923, TC426016, DR734732, TC387686, TC373251, TC413124, TC388566, TC399245, TC373429, GH729256, TC418716, TC392447, TC421467, TC379023, TC386519, TC394219, TC383047, TC417992, TC385090, TC407964, TC394307, TC388665, TC373583, TC406371, TC382139, TC392363, TC370106, TC388819, TC405475, TC386401, TC422022, TC386344, TC452356, TC373637, TC370044, TC421872, TC386422, TC442623, CJ776780, TC422583, TC400379, TC414606, TC393037, CJ730226, TC421733, TC383570, TC396228, TC379965, TC384678, TC456370, TC404053, TC400562, TC371970, TC375726, TC459656, TC378783, TC379436, TC377765, TC403929, TC457991, TC394118, TC373848, TC426870, TC437918, TC387007, TC375935, TC389816, TC372530, TC392323, TC370907, TC384194, TC413066, TC371172, TC390211, TC372167, TC461622, TC393426, TC400056, TC405033, TC379470, TC419090, TC385659, TC382910, TC432678, TC389451, TC458892, DR740372, TC394693, TC416495, TC401758, TC428944, TC417067, TC449093, TC379942, TC434821, TC374404, TC396230, BQ295499, TC377766, TC394965, TC368599, TC391648, TC376937, TC433162, TC400317, TC404978, TC389363, TC371248, TC379338, TC433116, TC409167, TC416442, TC374679, TC387410, TC378072, TC397176, TC406516, TC406986, TC371145, TC435546, TC419057, TC451950, TC380695, TC398714, TC381619, TC424179, TC394028, TC398970, TC457126, TC456854, TC403679, TC418747, TC440803, TC413427, TC386305, TC424044, TC372667, TC433957, TC392432, TC386440, TC423239, TC425821, TC370962, TC403055, TC394206, TC406236, TC382080, TC391962, TC391128, TC445767, TC387501, TC446038, TC374852, TC369830, TC423804, TC418685, CK213497, TC414899, TC436309, TC398514, TC391900, TC392033, TC423091, TC447694, TC380825, TC412520, TC406807, TC395090, TC404606, TC441239, TC416906, TC416438, CA595837, TC423265, TC417388, TC440066, TC386313, CK208222, TC404179, TC389108, TC369844, TC420579, CK211707, TC392126, TC402340, TC431201, CK217689, TC370392, CV761547, TC396751, CK211589, TC411116, TC388718, TC383701, TC452793, TC385780, TC405784, TC395566, TC372175, TC382734, TC405896, TC385927, TC369072, TC385513, TC439501, TC405356, TC444586, TC449448, TC377441, TC381596, TC403264, TC369348, TC461971, TC435825, TC422176, TC388232, TC445281, TC376263, TC415521, TC384010, TC416169, TC409043, TC408229, TC377290, TC409190, TC403537, TC381255, CV781430, TC379924, TC418399, TC431382, TC387683, TC409855, TC434831, TC406194, TC427161, TC373914, TC450285, TC392303, TC382024, TC404998, TC369899, TC372330, TC448051, TC381068, CD930656, TC403936, CF554444, TC451276, TC444375, TC387050, TC388914, TC415056, TC433488, TC377178, TC377438, TC394716, TC397027, TC456160, TC394670, TC377292, TC380433, TC451621, TC435281, TC384219, TC397562, TC428853, TC404413, TC408192, CJ626653, TC372046, CK210754, TC407191, TC370727, TC417769, TC449235, TC410147, TC414564, TC405910, TC431603, TC393904, TC387191, TC407395, TC447801, TC380416, TC427981, CA500690, TC398536, TC400755, TC403717, TC372701, TC405615, TC376420, TC458987, TC404843, TC382737, TC370633, TC390135, TC411480, TC417755, TC420394, TC432320, TC387170, TC439225, TC380662, TC419727, TC394263, TC436772, TC395298, TC394253, TC412094, CV782550, TC389162, TC376630, CK212850, TC389678, TC399432, TC434006, TC434949, CA727263, TC379241, TC405295, TC392465, TC455676, TC373962, TC391411, TC375813, TC379343, TC374741, TC429809, TC386591, TC421704, TC420201, TC381963, TC456247, TC371455, TC374879, GH728664, TC385701, TC400181, TC418759, TC431825, TC377496, TC368549, TC401158, TC387133, TC415942, TC393561, TC393692, TC391671, TC373787, BQ160837, TC382928, TC397415, TC370507, TC369637, TC396487, TC436832, TC458699, TC380882, TC440321, TC372580, TC444588, TC377410, TC400721, TC451657, TC445214, TC451519, TC393820, TC435808, TC449739, TC416981, BE586004, TC397729, TC399408, TC417970, TC426743, TC396895, TC382342, TC393106, TC428932, TC431306, TC396460, TC397676, TC379968, TC399730, TC368580, TC441536, TC373613, TC386040, TC407978, TC390393, TC444546, TC392283, TC390516, TC416529, TC391946, TC460760, TC420420, TC408524, TC417106, TC432124, CA710880, TC452945, TC375593, TC421871, TC371242, TC394459, TC423252, TC384738, TC416154, TC397660, CV766916, TC390379, TC407404, TC374996, TC393781, TC435210, TC398538, TC380063, TC418414, TC396772, TC378054, TC453207, TC419273, TC461607, TC409843, TC437445, TC403872, TC407076, TC371357, TC406207, TC389586, TC395746, TC439210, TC398304, TC393100, TC425878, CV771134, BE431108, TC417012, TC391447, TC394916, TC404914, TC391130, TC378274, CK217367, TC378153, TC379990, TC378333, TC444410, TC435863, BQ788843, TC452050, CJ845228, TC379536, TC391621, TC404052, TC389718, TC429713, CK201148, TC386088, TC389534, TC413392, TC422142, TC398379, TC376758, CJ815867, TC369628, TC441343, TC400260, TC400330, TC399057, TC408191, TC378432, TC377061, TC389843, TC388317, CD937281, TC416801, AL827131, TC446465, TC379312, TC391438, TC381268, TC412317, TC371325, TC439708, TC428113, TC373563, TC370619, BJ258087, TC421345, TC422007, TC370178, TC405695, TC388403, TC373678, TC416695, TC410690, TC374461, TC410074, TC443289, TC386646, TC399841, TC369687, TC458205, TC441241, TC433557, TC413571, TC415803, TC408299, TC410108, TC454526, TC398830, TC399352, TC422425, TC371129, TC398805, TC372654, TC428992, TC420816, TC384162, TC395872, TC383739, TC399342, TC432185, TC381583, TC455736, TC392263, TC432130, TC375665, TC385205, TC427986, TC422645, BG904091, TC421162, TC398121, TC389168, TC424204, TC422288, TC406870, TC424317, TC393814, TC376220, TC373402, TC404926, TC446235, TC402545, TC393830, TC444899, TC386961, TC398606, TC387319, TC396134, TC369182, TC418365, TC370347, TC373002, TC375554, CK200433, TC393970, TC374731, TC452494, TC388228, TC444138, TC377373, EB512907, TC390994, TC393437, TC412205, TC426916, TC391143, TC381463, TC403157, TC410066, TC414090, TC395303, TC442140, TC405511, TC386410, TC392203, TC393890, TC442727, TC390762, TC401210, DR737360, TC381007, TC397885, TC411684, TC382728, TC388049, TC404850, TC370033, TC433940, TC374485, TC393960, TC390436, TC426358, TC385326, TC387707, TC409077, TC443387, TC415083, TC370470, TC386535, TC390630, TC390402, TC374726, TC417341, TC415954, TC376790, TC398592, TC389589, TC378911, TC452762, TC407490, CD878039, TC374164, TC377993, DR735108, TC438657, TC412150, TC426078, TC388822, TC429771, TC375069, TC386639, TC416658, TC424962, TC379944, TC374230, TC391913, TC375834, TC393948, TC403624, TC400638, TC393554, TC407614, TC384049, TC391007, TC384357, TC460615, TC403573, TC411941, TC385445, TC395841, TC449043, TC379635, TC392247, TC373259, TC433589, TC382338, TC375914, TC398343, TC377253, TC404426, TC439472, TC377944, TC381817, TC403588, TC394728, TC403803, TC418850, TC440526, TC436944, TC371341, TC381279, TC389661, CA726837, TC387386, TC383820, TC396657, TC380333, TC377064, TC397258, TC426838, CV775873, TC431330, TC384373, TC416069, TC427210, TC422606, TC399271, TC375313, TC398862, DR739303, TC434442, TC413263, TC411471, TC431660, TC434570, TC378878, TC406264, TC390792, TC394020, TC388950, TC374669, TC419222, TC410194, TC369371, TC448840, TC392875, TC409005, TC408228, TC384735, TC418728, TC405540, TC390569, TC391948, BQ294582, TC401915, CA724903, CV779166, DR739994, TC459193, TC395506, TC395672, TC450972, TC392358, TC409599, TC419165, TC389092, TC376800, TC379853, TC420248, TC370315, TC449504, TC403687, TC405440, TC460795, CJ792862, TC422789, TC370885, TC407232, TC440819, TC416492, TC393015, TC426980, TC377225, TC404620, TC382858, TC403977, TC429865, TC434820, TC379711, TC379903, TC377308, TC406749, TC392272, TC401244, CA730421, TC400114, TC413339, TC408907, TC375539, TC385013, TC380083, TC386963, TC412732, TC381191, TC369010, TC402080, TC373518, TC370350, TC383909, TC376527, TC374940, TC419584, CN010697, TC374392, TC378568, TC369983, CV761628, TC404420, TC383235, TC373489, TC369199, TC419747, TC407304, CA676115, TC402186, BE415178, TC429062, TC423354, TC409344, TC413043, BJ243383, TC400484, CK214224, TC368603, TC398040, TC374964, TC375234, CA709177, TC385815, TC409187, TC429374, TC425690, TC375253, TC423182, TC381218, TC401124, TC375873, TC444302, TC435224, CJ563078, TC394850, TC384344, TC392297, TC388520, TC443712, TC379422, TC378956, TC411191, TC426326, TC375431, TC424252, TC391995, TC422841, TC387064, TC445649, CA498496, TC394796, TC382777, TC435533, TC407349, TC397994, TC414243, TC403234, TC429437, TC430988, TC371738, TC413771, TC387621, TC398633, TC460689, TC380125, TC369064, TC432154, TC420735, TC404371, TC402939, TC427405, TC449256, TC373615, TC383949, TC378409, TC416685, TC451342, TC372664, TC399986, TC389375, TC433812, TC370114, TC406038, TC390319, TC376874, TC424154, TC458807, TC459245, TC429871, TC369928, TC419438, TC376490, TC384454, TC411287, TC386237, TC388751, TC431722, AL820497, TC435909, TC399471, TC408126, TC430096, TC399972, TC434396, TC421880, TC386414, TC389590, CJ854725, TC440636, TC403986, TC406193, TC383763, CD876572, TC369736, TC386279, CK197833, TC391785, TC377427, TC382786, TC409650, TC388158, TC418340, TC405773, TC382830, TC377653, DR739471, TC402534, TC382129, TC391840, TC390285, TC412989, TC387766, TC407340, TC398026, TC406584, CV065343, TC402440, TC396636, TC375146, TC454407, TC394661, TC389044, TC383176, TC449463, TC371037, TC388688, TC403328, TC408095, TC439939, TC375268, TC452503, TC388547, TC420877, TC429048, TC398978, TC388976, TC396365, TC379069, TC385526, TC423576, TC375530, TC377635, TC402668, TC435799, TC438243, TC392887, TC370210, TC375918, TC417363, TC400592, TC413700, TC387710, TC385515, TC423880, TC451285, BG263159, TC458503, TC387194, TC391865, TC434689, TC441995, CK211469, CB307332, TC404842, TC381296, TC411817, TC436290, TC390489, TC421341, TC432504, TC383143 |
| [Intracellular membrane-bounded organelle](http://amigo.geneontology.org/cgi-bin/amigo/go.cgi?action=query&view=query&query=GO:0043231&search_constraint=terms) | TC461921, BJ284275, TC410352, TC390944, TC370791, TC389190, TC453487, TC375864, TC432212, TC415588, TC389842, BQ838511, TC398052, TC387861, TC391749, TC374098, TC400388, TC457112, TC431932, CK215979, TC376351, TC430471, TC439423, TC383028, TC379171, TC388410, BJ282766, CK203550, TC416493, TC388136, TC458562, TC393436, TC417260, CK214103, TC433844, CK209294, TC410063, TC421143, TC386396, TC378615, TC373702, TC448471, TC456784, TC395453, TC392329, TC410182, TC445939, TC425847, CJ727624, CA614761, CJ550278, BE591166, TC411128, GH732878, TC397444, TC412569, TC415483, CA632212, TC393523, TC435852, TC413027, TC372549, TC394820, TC398731, TC418073, TC411784, TC417308, TC387817, TC395069, TC413460, TC411008, BQ609416, CK214702, TC426907, TC395723, TC369415, TC387981, TC385233, TC391950, TC398730, TC386707, TC376248, BE217043, TC374409, TC402069, TC381943, TC432001, TC420358, CV770684, TC418928, TC398209, BQ607161, TC372845, TC382045, TC431175, TC402308, TC378790, TC417077, TC385009, CV759879, TC376625, TC390016, TC380474, CA606693, TC370912, TC432369, TC385714, TC412212, TC375124, TC392778, TC397312, TC405030, CA615187, TC405041, TC444549, BJ317882, TC406469, TC399919, TC407572, TC413118, TC406708, TC373898, TC418691, TC409459, TC420337, TC391360, TC416471, TC419919, CV763657, TC372761, TC406809, TC410569, TC437137, CN010359, TC373001, TC402603, TC449724, TC395914, TC372867, TC397909, CA729339, BQ239045, TC430163, TC389993, TC426864, TC408312, TC391156, CA613620, TC400362, TC398154, TC401293, TC369325, TC429747, TC375612, TC430501, CJ867263, TC400108, TC406106, TC381462, CD882425, TC377749, TC396451, TC431198, TC424344, TC429217, TC372677, CJ868963, TC374240, TC376306, TC425841, TC405982, TC387579, TC420460, TC428008, TC427006, TC380943, TC376202, TC403885, TC405946, TC460907, TC394451, TC395312, CV772029, TC387344, TC389090, TC389139, TC412483, TC382655, TC389144, TC456230, TC373994, TC395351, TC392709, TC372563, TC418032, CK162413, TC397291, TC461977, TC411908, CA598430, TC459008, TC380329, TC376930, TC391516, TC409343, TC389696, TC403852, TC382742, TC377190, TC443814, TC431879, TC423110, TC422348, TC391613, TC385365, TC430544, TC382339, TC379357, TC371003, TC387135, TC378437, TC438587, TC375760, TC381923, TC426016, DR734732, TC387686, TC373251, TC413124, TC388566, TC399245, TC373429, GH729256, TC418716, TC392447, TC421467, TC379023, TC386519, TC394219, TC383047, TC417992, TC385090, TC407964, TC394307, TC388665, TC373583, TC406371, TC382139, TC392363, TC370106, TC388819, TC405475, TC386401, TC422022, TC386344, TC452356, TC373637, TC370044, TC421872, TC386422, TC442623, CJ776780, TC422583, TC400379, TC414606, TC393037, CJ730226, TC421733, TC383570, TC396228, TC379965, TC384678, TC456370, TC404053, TC400562, TC371970, TC375726, TC459656, TC378783, TC379436, TC377765, TC403929, TC457991, TC394118, TC373848, TC426870, TC437918, TC387007, TC375935, TC389816, TC372530, TC392323, TC370907, TC384194, TC413066, TC371172, TC390211, TC372167, TC461622, TC393426, TC400056, TC405033, TC379470, TC419090, TC385659, TC382910, TC432678, TC389451, TC458892, DR740372, TC394693, TC416495, TC401758, TC428944, TC417067, TC449093, TC379942, TC434821, TC374404, TC396230, BQ295499, TC377766, TC394965, TC368599, TC391648, TC376937, TC433162, TC400317, TC404978, TC389363, TC371248, TC379338, TC433116, TC409167, TC416442, TC374679, TC387410, TC378072, TC397176, TC406516, TC406986, TC371145, TC435546, TC419057, TC451950, TC380695, TC398714, TC381619, TC424179, TC394028, TC398970, TC457126, TC456854, TC403679, TC418747, TC440803, TC413427, TC386305, TC424044, TC372667, TC433957, TC392432, TC386440, TC423239, TC425821, TC370962, TC403055, TC394206, TC406236, TC382080, TC391962, TC391128, TC445767, TC387501, TC446038, TC374852, TC369830, TC423804, TC418685, CK213497, TC414899, TC436309, TC398514, TC391900, TC392033, TC423091, TC447694, TC380825, TC412520, TC406807, TC395090, TC404606, TC441239, TC416906, TC416438, CA595837, TC423265, TC417388, TC440066, TC386313, CK208222, TC404179, TC389108, TC369844, TC420579, CK211707, TC392126, TC402340, TC431201, CK217689, TC370392, CV761547, TC396751, CK211589, TC411116, TC388718, TC383701, TC452793, TC385780, TC405784, TC395566, TC372175, TC382734, TC405896, TC385927, TC369072, TC385513, TC439501, TC405356, TC444586, TC449448, TC377441, TC381596, TC403264, TC369348, TC461971, TC435825, TC422176, TC388232, TC445281, TC376263, TC415521, TC384010, TC416169, TC409043, TC408229, TC377290, TC409190, TC403537, TC381255, CV781430, TC379924, TC418399, TC431382, TC387683, TC409855, TC434831, TC406194, TC427161, TC373914, TC450285, TC392303, TC382024, TC404998, TC369899, TC372330, TC448051, TC381068, CD930656, TC403936, CF554444, TC451276, TC444375, TC387050, TC388914, TC415056, TC433488, TC377178, TC377438, TC394716, TC397027, TC456160, TC394670, TC377292, TC380433, TC451621, TC435281, TC384219, TC397562, TC428853, TC404413, TC408192, CJ626653, TC372046, CK210754, TC407191, TC370727, TC417769, TC449235, TC410147, TC414564, TC405910, TC431603, TC393904, TC387191, TC407395, TC447801, TC380416, TC427981, CA500690, TC398536, TC400755, TC403717, TC372701, TC405615, TC376420, TC458987, TC404843, TC382737, TC370633, TC390135, TC411480, TC417755, TC420394, TC432320, TC387170, TC439225, TC380662, TC419727, TC394263, TC436772, TC395298, TC394253, TC412094, CV782550, TC389162, TC376630, CK212850, TC389678, TC399432, TC434006, TC434949, CA727263, TC379241, TC405295, TC392465, TC455676, TC373962, TC391411, TC375813, TC379343, TC374741, TC429809, TC386591, TC421704, TC420201, TC381963, TC456247, TC371455, TC374879, GH728664, TC385701, TC400181, TC418759, TC431825, TC377496, TC368549, TC401158, TC387133, TC415942, TC393561, TC393692, TC391671, TC373787, BQ160837, TC382928, TC397415, TC370507, TC369637, TC396487, TC436832, TC458699, TC380882, TC440321, TC372580, TC444588, TC377410, TC400721, TC451657, TC445214, TC451519, TC393820, TC435808, TC449739, TC416981, BE586004, TC397729, TC399408, TC417970, TC426743, TC396895, TC382342, TC393106, TC428932, TC431306, TC396460, TC397676, TC379968, TC399730, TC368580, TC441536, TC373613, TC386040, TC407978, TC390393, TC444546, TC392283, TC390516, TC416529, TC391946, TC460760, TC420420, TC408524, TC417106, TC432124, CA710880, TC452945, TC375593, TC421871, TC371242, TC394459, TC423252, TC384738, TC416154, TC397660, CV766916, TC390379, TC407404, TC374996, TC393781, TC435210, TC398538, TC380063, TC418414, TC396772, TC378054, TC453207, TC419273, TC461607, TC409843, TC437445, TC403872, TC407076, TC371357, TC406207, TC389586, TC395746, TC439210, TC398304, TC393100, TC425878, CV771134, BE431108, TC417012, TC391447, TC394916, TC404914, TC391130, TC378274, CK217367, TC378153, TC379990, TC378333, TC444410, TC435863, BQ788843, TC452050, CJ845228, TC379536, TC391621, TC404052, TC389718, TC429713, CK201148, TC386088, TC389534, TC413392, TC422142, TC398379, TC376758, CJ815867, TC369628, TC441343, TC400260, TC400330, TC399057, TC408191, TC378432, TC377061, TC389843, TC388317, CD937281, TC416801, AL827131, TC446465, TC379312, TC391438, TC381268, TC412317, TC371325, TC439708, TC428113, TC373563, TC370619, BJ258087, TC421345, TC422007, TC370178, TC405695, TC388403, TC373678, TC416695, TC410690, TC374461, TC410074, TC443289, TC386646, TC399841, TC369687, TC458205, TC441241, TC433557, TC413571, TC415803, TC408299, TC410108, TC454526, TC398830, TC399352, TC422425, TC371129, TC398805, TC372654, TC428992, TC420816, TC384162, TC395872, TC383739, TC399342, TC432185, TC381583, TC455736, TC392263, TC432130, TC375665, TC385205, TC427986, TC422645, BG904091, TC421162, TC398121, TC389168, TC424204, TC422288, TC406870, TC424317, TC393814, TC376220, TC373402, TC404926, TC446235, TC402545, TC393830, TC444899, TC386961, TC398606, TC387319, TC396134, TC369182, TC418365, TC370347, TC373002, TC375554, CK200433, TC393970, TC374731, TC452494, TC388228, TC444138, TC377373, EB512907, TC390994, TC393437, TC412205, TC426916, TC391143, TC381463, TC403157, TC410066, TC414090, TC395303, TC442140, TC405511, TC386410, TC392203, TC393890, TC442727, TC390762, TC401210, DR737360, TC381007, TC397885, TC411684, TC382728, TC388049, TC404850, TC370033, TC433940, TC374485, TC393960, TC390436, TC426358, TC385326, TC387707, TC409077, TC443387, TC415083, TC370470, TC386535, TC390630, TC390402, TC374726, TC417341, TC415954, TC376790, TC398592, TC389589, TC378911, TC452762, TC407490, CD878039, TC374164, TC377993, DR735108, TC438657, TC412150, TC426078, TC388822, TC429771, TC375069, TC386639, TC416658, TC424962, TC379944, TC374230, TC391913, TC375834, TC393948, TC403624, TC400638, TC393554, TC407614, TC384049, TC391007, TC384357, TC460615, TC403573, TC411941, TC385445, TC395841, TC449043, TC379635, TC392247, TC373259, TC433589, TC382338, TC375914, TC398343, TC377253, TC404426, TC439472, TC377944, TC381817, TC403588, TC394728, TC403803, TC418850, TC440526, TC436944, TC371341, TC381279, TC389661, CA726837, TC387386, TC383820, TC396657, TC380333, TC377064, TC397258, TC426838, CV775873, TC431330, TC384373, TC416069, TC427210, TC422606, TC399271, TC375313, TC398862, DR739303, TC434442, TC413263, TC411471, TC431660, TC434570, TC378878, TC406264, TC390792, TC394020, TC388950, TC374669, TC419222, TC410194, TC369371, TC448840, TC392875, TC409005, TC408228, TC384735, TC418728, TC405540, TC390569, TC391948, BQ294582, TC401915, CA724903, CV779166, DR739994, TC459193, TC395506, TC395672, TC450972, TC392358, TC409599, TC419165, TC389092, TC376800, TC379853, TC420248, TC370315, TC449504, TC403687, TC405440, TC460795, CJ792862, TC422789, TC370885, TC407232, TC440819, TC416492, TC393015, TC426980, TC377225, TC404620, TC382858, TC403977, TC429865, TC434820, TC379711, TC379903, TC377308, TC406749, TC392272, TC401244, CA730421, TC400114, TC413339, TC408907, TC375539, TC385013, TC380083, TC386963, TC412732, TC381191, TC369010, TC402080, TC373518, TC370350, TC383909, TC376527, TC374940, TC419584, CN010697, TC374392, TC378568, TC369983, CV761628, TC404420, TC383235, TC373489, TC369199, TC419747, TC407304, CA676115, TC402186, BE415178, TC429062, TC423354, TC409344, TC413043, BJ243383, TC400484, CK214224, TC368603, TC398040, TC374964, TC375234, CA709177, TC385815, TC409187, TC429374, TC425690, TC375253, TC423182, TC381218, TC401124, TC375873, TC444302, TC435224, CJ563078, TC394850, TC384344, TC392297, TC388520, TC443712, TC379422, TC378956, TC411191, TC426326, TC375431, TC424252, TC391995, TC422841, TC387064, TC445649, CA498496, TC394796, TC382777, TC435533, TC407349, TC397994, TC414243, TC403234, TC429437, TC430988, TC371738, TC413771, TC387621, TC398633, TC460689, TC380125, TC369064, TC432154, TC420735, TC404371, TC402939, TC427405, TC449256, TC373615, TC383949, TC378409, TC416685, TC451342, TC372664, TC399986, TC389375, TC433812, TC370114, TC406038, TC390319, TC376874, TC424154, TC458807, TC459245, TC429871, TC369928, TC419438, TC376490, TC384454, TC411287, TC386237, TC388751, TC431722, AL820497, TC435909, TC399471, TC408126, TC430096, TC399972, TC434396, TC421880, TC386414, TC389590, CJ854725, TC440636, TC403986, TC406193, TC383763, CD876572, TC369736, TC386279, CK197833, TC391785, TC377427, TC382786, TC409650, TC388158, TC418340, TC405773, TC382830, TC377653, DR739471, TC402534, TC382129, TC391840, TC390285, TC412989, TC387766, TC407340, TC398026, TC406584, CV065343, TC402440, TC396636, TC375146, TC454407, TC394661, TC389044, TC383176, TC449463, TC371037, TC388688, TC403328, TC408095, TC439939, TC375268, TC452503, TC388547, TC420877, TC429048, TC398978, TC388976, TC396365, TC379069, TC385526, TC423576, TC375530, TC377635, TC402668, TC435799, TC438243, TC392887, TC370210, TC375918, TC417363, TC400592, TC413700, TC387710, TC385515, TC423880, TC451285, BG263159, TC458503, TC387194, TC391865, TC434689, TC441995, CK211469, CB307332, TC404842, TC381296, TC411817, TC436290, TC390489, TC421341, TC432504, TC383143 |
| [Calcium- and calmodulin-dependent protein kinase complex](http://amigo.geneontology.org/cgi-bin/amigo/go.cgi?action=query&view=query&query=GO:0005954&search_constraint=terms) | CA646741, TC374240, TC395723, CJ550278 |
| [Rad17 RFC-like complex](http://amigo.geneontology.org/cgi-bin/amigo/go.cgi?action=query&view=query&query=GO:0031389&search_constraint=terms) | TC389162, TC374164 |
| [Elg1 RFC-like complex](http://amigo.geneontology.org/cgi-bin/amigo/go.cgi?action=query&view=query&query=GO:0031391&search_constraint=terms) | TC389162, TC374164 |
| [Mitochondrial small ribosomal subunit](http://amigo.geneontology.org/cgi-bin/amigo/go.cgi?action=query&view=query&query=GO:0005763&search_constraint=terms) | TC396451, TC368603, TC371970 |
| [6-phosphofructokinase complex](http://amigo.geneontology.org/cgi-bin/amigo/go.cgi?action=query&view=query&query=GO:0005945&search_constraint=terms) | TC388751, TC379422 |
| [Storage vacuole](http://amigo.geneontology.org/cgi-bin/amigo/go.cgi?action=query&view=query&query=GO:0000322&search_constraint=terms) | TC405511, TC423354, TC403588, TC380882, TC423880 |
| [Gap junction](http://amigo.geneontology.org/cgi-bin/amigo/go.cgi?action=query&view=query&query=GO:0005921&search_constraint=terms) | TC387135, TC459656, TC398730, TC406807 |
| [Connexon complex](http://amigo.geneontology.org/cgi-bin/amigo/go.cgi?action=query&view=query&query=GO:0005922&search_constraint=terms) | TC387135, TC459656, TC398730, TC406807 |
| [Cytoplasmic membrane-bounded vesicle](http://amigo.geneontology.org/cgi-bin/amigo/go.cgi?action=query&view=query&query=GO:0016023&search_constraint=terms) | BJ284275, TC368580, TC390944, TC379942, TC370791, TC434821, TC392283, CV775873, TC377766, TC432212, TC389842, TC427210, TC398052, TC432124, TC391648, TC400388, TC398862, CK215979, TC376351, TC394459, TC384738, TC383028, DR739303, TC404978, TC388410, TC379338, TC458562, TC409167, TC374679, TC434570, TC387410, TC378878, CK214103, TC433844, TC418414, TC390792, TC394020, TC406986, TC396772, TC373702, TC374669, TC448840, TC369371, TC410182, TC409843, TC405540, TC445939, TC371357, TC398714, TC424179, TC406207, TC394028, TC456854, TC403679, TC440803, TC411128, TC398304, TC393100, TC433957, TC415483, CV771134, TC391447, TC393523, TC403055, TC394916, TC372549, TC404914, TC378274, TC382080, TC391128, TC419165, TC378153, TC387817, TC369830, TC374852, TC395069, TC413460, TC411008, TC378333, TC452050, TC403687, CJ845228, TC418685, TC414899, TC391621, TC398514, TC377225, TC376248, TC423091, TC429713, TC374409, CK201148, TC386088, TC404620, TC398209, TC376758, TC395090, TC382045, TC431175, TC416906, TC378790, TC377308, TC406749, TC389843, TC417388, CD937281, TC400114, TC379312, TC386313, TC385009, TC404179, TC390016, TC369844, TC381268, CA606693, TC380083, CK211707, TC386963, TC392126, TC402340, TC385714, TC428113, TC375124, TC421345, TC422007, CK217689, TC370392, TC373518, TC396751, TC373678, CA615187, TC374940, BJ317882, TC404420, TC405784, TC395566, TC399919, TC407572, TC372175, TC382734, TC369199, TC419747, TC373898, TC443289, TC385927, TC386646, TC369072, TC372761, TC449448, TC381596, CN010359, TC441241, TC433557, TC449724, TC395914, TC409344, TC372867, TC461971, TC435825, TC422176, TC410108, TC415521, TC385815, TC429374, TC384010, TC375253, TC422425, TC375873, TC435224, TC372654, TC403537, TC401293, TC369325, TC443712, TC379924, TC378956, TC375612, TC411191, TC431382, TC406194, TC391995, TC424252, TC375431, TC377749, TC381583, TC387064, TC422841, TC424344, TC432130, TC385205, TC382777, TC381068, TC421162, CJ868963, TC425841, TC407349, TC397994, TC387579, TC451276, TC424317, TC377178, TC433488, TC394670, TC380125, TC380433, TC405946, TC435281, TC460907, TC386961, TC449256, CV772029, TC398606, TC378409, TC387344, TC428853, TC370347, TC424154, TC376874, CJ626653, TC458807, TC392709, CK210754, TC429871, TC407191, TC370727, TC419438, CK162413, TC410147, TC461977, TC377373, CA598430, TC386237, TC405910, TC431603, TC390994, TC407395, TC426916, TC380416, TC408126, TC430096, TC427981, TC395303, TC400755, TC405511, TC458987, TC383763, TC382339, TC390135, TC381007, TC417755, TC382728, TC420394, CK197833, TC438587, TC375760, TC381923, TC377427, TC409650, TC439225, TC426016, TC370033, TC380662, TC374485, TC418340, TC385326, TC412094, TC405773, CV782550, TC387707, TC376630, TC382830, TC377653, CK212850, TC388566, TC434006, TC370470, TC390630, TC412989, TC398026, TC418716, TC421467, TC379023, TC394219, TC374726, TC455676, TC373962, TC415954, TC376790, TC373583, TC406371, TC382139, TC429809, TC386591, TC420201, TC389044, TC421872, TC456247, TC439939, TC408095, TC422583, CJ776780, TC442623, TC452503, TC438657, DR735108, TC388547, TC431825, TC412150, TC420877, CJ730226, TC396365, TC398978, TC384678, TC374230, TC391913, TC393948, TC373787, TC384049, TC397415, TC382928, TC378783, TC384357, TC370210, TC377765, TC417363, TC369637, TC396487, TC436832, TC458699, TC457991, TC403929, TC385515, TC395841, TC372580, TC437918, TC375935, TC382338, TC375914, TC400721, TC451657, TC372530, TC377253, TC391865, TC381296, BE586004, TC432678, DR740372, TC440526, TC421341, TC401758, TC389661, TC387386, TC383820, TC396657 |
| [Membrane-bounded vesicle](http://amigo.geneontology.org/cgi-bin/amigo/go.cgi?action=query&view=query&query=GO:0031988&search_constraint=terms) | BJ284275, TC368580, TC390944, TC379942, TC370791, TC434821, TC392283, CV775873, TC377766, TC432212, TC389842, TC427210, TC398052, TC432124, TC391648, TC400388, TC398862, CK215979, TC376351, TC394459, TC384738, TC383028, DR739303, TC404978, TC388410, TC379338, TC458562, TC409167, TC374679, TC434570, TC387410, TC378878, CK214103, TC433844, TC418414, TC390792, TC394020, TC406986, TC396772, TC373702, TC374669, TC448840, TC369371, TC410182, TC409843, TC405540, TC445939, TC371357, TC398714, TC424179, TC406207, TC394028, TC456854, TC403679, TC440803, TC411128, TC398304, TC393100, TC433957, TC415483, CV771134, TC391447, TC393523, TC403055, TC394916, TC372549, TC404914, TC378274, TC382080, TC391128, TC419165, TC378153, TC387817, TC369830, TC374852, TC395069, TC413460, TC411008, TC378333, TC452050, TC403687, CJ845228, TC418685, TC414899, TC391621, TC398514, TC377225, TC376248, TC423091, TC429713, TC374409, CK201148, TC386088, TC404620, TC398209, TC376758, TC395090, TC382045, TC431175, TC416906, TC378790, TC377308, TC406749, TC389843, TC417388, CD937281, TC400114, TC379312, TC386313, TC385009, TC404179, TC390016, TC369844, TC381268, CA606693, TC380083, CK211707, TC386963, TC392126, TC402340, TC385714, TC428113, TC375124, TC421345, TC422007, CK217689, TC370392, TC373518, TC396751, TC373678, CA615187, TC374940, BJ317882, TC404420, TC405784, TC395566, TC399919, TC407572, TC372175, TC382734, TC369199, TC419747, TC373898, TC443289, TC385927, TC386646, TC369072, TC372761, TC449448, TC381596, CN010359, TC441241, TC433557, TC449724, TC395914, TC409344, TC372867, TC461971, TC435825, TC422176, TC410108, TC415521, TC385815, TC429374, TC384010, TC375253, TC422425, TC375873, TC435224, TC372654, TC403537, TC401293, TC369325, TC443712, TC379924, TC378956, TC375612, TC411191, TC431382, TC406194, TC391995, TC424252, TC375431, TC377749, TC381583, TC387064, TC422841, TC424344, TC432130, TC385205, TC382777, TC381068, TC421162, CJ868963, TC425841, TC407349, TC397994, TC387579, TC451276, TC424317, TC377178, TC433488, TC394670, TC380125, TC380433, TC405946, TC435281, TC460907, TC386961, TC449256, CV772029, TC398606, TC378409, TC387344, TC428853, TC370347, TC424154, TC376874, CJ626653, TC458807, TC392709, CK210754, TC429871, TC407191, TC370727, TC419438, CK162413, TC410147, TC461977, TC377373, CA598430, TC386237, TC405910, TC431603, TC390994, TC407395, TC426916, TC380416, TC408126, TC430096, TC427981, TC395303, TC400755, TC405511, TC458987, TC383763, TC382339, TC390135, TC381007, TC417755, TC382728, TC420394, CK197833, TC438587, TC375760, TC381923, TC377427, TC409650, TC439225, TC426016, TC370033, TC380662, TC374485, TC418340, TC385326, TC412094, TC405773, CV782550, TC387707, TC376630, TC382830, TC377653, CK212850, TC388566, TC434006, TC370470, TC390630, TC412989, TC398026, TC418716, TC421467, TC379023, TC394219, TC374726, TC455676, TC373962, TC415954, TC376790, TC373583, TC406371, TC382139, TC429809, TC386591, TC420201, TC389044, TC421872, TC456247, TC439939, TC408095, TC422583, CJ776780, TC442623, TC452503, TC438657, DR735108, TC388547, TC431825, TC412150, TC420877, CJ730226, TC396365, TC398978, TC384678, TC374230, TC391913, TC393948, TC373787, TC384049, TC397415, TC382928, TC378783, TC384357, TC370210, TC377765, TC417363, TC369637, TC396487, TC436832, TC458699, TC457991, TC403929, TC385515, TC395841, TC372580, TC437918, TC375935, TC382338, TC375914, TC400721, TC451657, TC372530, TC377253, TC391865, TC381296, BE586004, TC432678, DR740372, TC440526, TC421341, TC401758, TC389661, TC387386, TC383820, TC396657 |
| [Cytoplasmic vesicle](http://amigo.geneontology.org/cgi-bin/amigo/go.cgi?action=query&view=query&query=GO:0031410&search_constraint=terms) | BJ284275, TC368580, TC390944, TC379942, TC370791, TC434821, TC392283, CV775873, TC377766, TC432212, TC389842, TC427210, TC398052, TC432124, TC391648, TC400388, TC398862, CK215979, TC376351, TC394459, TC384738, TC383028, DR739303, TC404978, TC388410, TC379338, TC458562, TC409167, TC374679, TC434570, TC387410, TC378878, CK214103, TC433844, TC418414, TC390792, TC394020, TC406986, TC396772, TC373702, TC374669, TC448840, TC369371, TC410182, TC409843, TC405540, TC445939, TC371357, TC398714, TC424179, TC406207, TC394028, TC456854, TC403679, TC440803, TC411128, TC398304, TC393100, TC433957, TC415483, CV771134, TC391447, TC393523, TC403055, TC394916, TC372549, TC404914, TC378274, TC382080, TC391128, TC419165, TC378153, TC387817, TC369830, TC374852, TC395069, TC413460, TC411008, TC378333, TC452050, TC403687, CJ845228, TC418685, TC414899, TC391621, TC398514, TC377225, TC376248, TC423091, TC429713, TC374409, CK201148, TC386088, TC404620, TC398209, TC376758, TC395090, TC382045, TC431175, TC416906, TC378790, TC377308, TC406749, TC389843, TC417388, CD937281, TC400114, TC379312, TC386313, TC385009, TC404179, TC390016, TC369844, TC381268, CA606693, TC380083, CK211707, TC386963, TC392126, TC402340, TC385714, TC428113, TC375124, TC421345, TC422007, CK217689, TC370392, TC373518, TC396751, TC373678, CA615187, TC374940, BJ317882, TC404420, TC405784, TC395566, TC399919, TC407572, TC372175, TC382734, TC369199, TC419747, TC373898, TC443289, TC385927, TC386646, TC369072, TC372761, TC449448, TC381596, CN010359, TC441241, TC433557, TC449724, TC395914, TC409344, TC372867, TC461971, TC435825, TC422176, TC410108, TC415521, TC385815, TC429374, TC384010, TC375253, TC422425, TC375873, TC435224, TC372654, TC403537, TC401293, TC369325, TC443712, TC379924, TC378956, TC375612, TC411191, TC431382, TC406194, TC391995, TC424252, TC375431, TC377749, TC381583, TC387064, TC422841, TC424344, TC432130, TC385205, TC382777, TC381068, TC421162, CJ868963, TC425841, TC407349, TC397994, TC387579, TC451276, TC424317, TC377178, TC433488, TC394670, TC380125, TC380433, TC405946, TC435281, TC460907, TC386961, TC449256, CV772029, TC398606, TC378409, TC387344, TC428853, TC370347, TC424154, TC376874, CJ626653, TC458807, TC392709, CK210754, TC429871, TC407191, TC370727, TC419438, CK162413, TC410147, TC461977, TC377373, CA598430, TC386237, TC405910, TC431603, TC390994, TC407395, TC426916, TC380416, TC408126, TC430096, TC427981, TC395303, TC400755, TC405511, TC458987, TC383763, TC382339, TC390135, TC381007, TC417755, TC382728, TC420394, CK197833, TC438587, TC375760, TC381923, TC377427, TC409650, TC439225, TC426016, TC370033, TC380662, TC374485, TC418340, TC385326, TC412094, TC405773, CV782550, TC387707, TC376630, TC382830, TC377653, CK212850, TC388566, TC434006, TC370470, TC390630, TC412989, TC398026, TC418716, TC421467, TC379023, TC394219, TC374726, TC455676, TC373962, TC415954, TC376790, TC373583, TC406371, TC382139, TC429809, TC386591, TC420201, TC389044, TC421872, TC456247, TC439939, TC408095, TC422583, CJ776780, TC442623, TC452503, TC438657, DR735108, TC388547, TC431825, TC412150, TC420877, CJ730226, TC396365, TC398978, TC384678, TC374230, TC391913, TC393948, TC373787, TC384049, TC397415, TC382928, TC378783, TC384357, TC370210, TC377765, TC417363, TC369637, TC396487, TC436832, TC458699, TC457991, TC403929, TC385515, TC395841, TC372580, TC437918, TC375935, TC382338, TC375914, TC400721, TC451657, TC372530, TC377253, TC391865, TC381296, BE586004, TC432678, DR740372, TC440526, TC421341, TC401758, TC389661, TC387386, TC383820, TC396657 |
| [Organelle membrane](http://amigo.geneontology.org/cgi-bin/amigo/go.cgi?action=query&view=query&query=GO:0031090&search_constraint=terms) | TC378790, CV775873, TC372677, TC386313, TC398052, TC390402, TC425841, TC387579, CA606693, TC394459, TC384738, CK211707, TC386963, TC388410, BJ282766, TC387621, TC458562, TC411471, TC396751, TC432154, TC386396, CA615187, TC386961, TC405041, TC388547, TC387344, TC405784, TC399919, TC407572, TC372175, TC404413, TC396365, TC373002, TC419747, TC398714, TC398304, TC433957, TC393100, CK210754, TC412569, TC382928, TC423354, TC441241, TC449724, TC397291, TC386237, TC417363, TC394916, TC409343, TC387191, TC390994, TC378274, TC403929, TC385515, TC380882, TC423880, TC395303, TC422425, TC400755, TC405511, TC458987, TC393820, TC400362, TC403588, TC390135, TC398514, TC377225, TC411191, TC388049, TC409650, TC380662, TC401758, TC431306, TC393960, TC389661 |
| [Chromatin assembly complex](http://amigo.geneontology.org/cgi-bin/amigo/go.cgi?action=query&view=query&query=GO:0005678&search_constraint=terms) | TC374240, TC395723, CJ550278 |
| [U1 snRNP](http://amigo.geneontology.org/cgi-bin/amigo/go.cgi?action=query&view=query&query=GO:0005685&search_constraint=terms) | TC369628, TC387135, TC385233, TC405540, TC388566 |
| [Yesicle](http://amigo.geneontology.org/cgi-bin/amigo/go.cgi?action=query&view=query&query=GO:0031982&search_constraint=terms) | BJ284275, TC368580, TC390944, TC379942, TC370791, TC434821, TC392283, CV775873, TC377766, TC432212, TC389842, TC427210, TC398052, TC432124, TC391648, TC400388, TC398862, CK215979, TC376351, TC394459, TC384738, TC383028, DR739303, TC404978, TC388410, TC379338, TC458562, TC409167, TC374679, TC434570, TC387410, TC378878, CK214103, TC433844, TC418414, TC390792, TC394020, TC406986, TC396772, TC373702, TC374669, TC448840, TC369371, TC410182, TC409843, TC405540, TC445939, TC371357, TC398714, TC424179, TC406207, TC394028, TC456854, TC403679, TC440803, TC411128, TC398304, TC393100, TC433957, TC415483, CV771134, TC391447, TC393523, TC403055, TC394916, TC372549, TC404914, TC378274, TC382080, TC391128, TC419165, TC378153, TC387817, TC369830, TC374852, TC395069, TC413460, TC411008, TC378333, TC452050, TC403687, CJ845228, TC418685, TC414899, TC391621, TC398514, TC377225, TC376248, TC423091, TC429713, TC374409, CK201148, TC386088, TC404620, TC398209, TC376758, TC395090, TC382045, TC431175, TC416906, TC378790, TC377308, TC406749, TC389843, TC417388, CD937281, TC400114, TC379312, TC386313, TC385009, TC404179, TC390016, TC369844, TC381268, CA606693, TC380083, CK211707, TC386963, TC392126, TC402340, TC385714, TC428113, TC375124, TC421345, TC422007, CK217689, TC370392, TC373518, TC396751, TC373678, CA615187, TC374940, BJ317882, TC404420, TC405784, TC395566, TC399919, TC407572, TC372175, TC382734, TC369199, TC419747, TC373898, TC443289, TC385927, TC386646, TC369072, TC372761, TC449448, TC381596, CN010359, TC441241, TC433557, TC449724, TC395914, TC409344, TC372867, TC461971, TC435825, TC422176, TC410108, TC415521, TC385815, TC429374, TC384010, TC375253, TC422425, TC375873, TC435224, TC372654, TC403537, TC401293, TC369325, TC443712, TC379924, TC378956, TC375612, TC411191, TC431382, TC406194, TC391995, TC424252, TC375431, TC377749, TC381583, TC387064, TC422841, TC424344, TC432130, TC385205, TC382777, TC381068, TC421162, CJ868963, TC425841, TC407349, TC397994, TC387579, TC451276, TC424317, TC377178, TC433488, TC394670, TC380125, TC380433, TC405946, TC435281, TC460907, TC386961, TC449256, CV772029, TC398606, TC378409, TC387344, TC428853, TC370347, TC424154, TC376874, CJ626653, TC458807, TC392709, CK210754, TC429871, TC407191, TC370727, TC419438, CK162413, TC410147, TC461977, TC377373, CA598430, TC386237, TC405910, TC431603, TC390994, TC407395, TC426916, TC380416, TC408126, TC430096, TC427981, TC395303, TC400755, TC405511, TC458987, TC383763, TC382339, TC390135, TC381007, TC417755, TC382728, TC420394, CK197833, TC438587, TC375760, TC381923, TC377427, TC409650, TC439225, TC426016, TC370033, TC380662, TC374485, TC418340, TC385326, TC412094, TC405773, CV782550, TC387707, TC376630, TC382830, TC377653, CK212850, TC388566, TC434006, TC370470, TC390630, TC412989, TC398026, TC418716, TC421467, TC379023, TC394219, TC374726, TC455676, TC373962, TC415954, TC376790, TC373583, TC406371, TC382139, TC429809, TC386591, TC420201, TC389044, TC421872, TC456247, TC439939, TC408095, TC422583, CJ776780, TC442623, TC452503, TC438657, DR735108, TC388547, TC431825, TC412150, TC420877, CJ730226, TC396365, TC398978, TC384678, TC374230, TC391913, TC393948, TC373787, TC384049, TC397415, TC382928, TC378783, TC384357, TC370210, TC377765, TC417363, TC369637, TC396487, TC436832, TC458699, TC457991, TC403929, TC385515, TC395841, TC372580, TC437918, TC375935, TC382338, TC375914, TC400721, TC451657, TC372530, TC377253, TC391865, TC381296, BE586004, TC432678, DR740372, TC440526, TC421341, TC401758, TC389661, TC387386, TC383820, TC396657 |
| [Cell junction](http://amigo.geneontology.org/cgi-bin/amigo/go.cgi?action=query&view=query&query=GO:0030054&search_constraint=terms) | TC387135, TC459656, TC398730, TC406807, TC370315, TC382045, TC378790 |
| [Mitochondrial ribosome](http://amigo.geneontology.org/cgi-bin/amigo/go.cgi?action=query&view=query&query=GO:0005761&search_constraint=terms) | TC396451, TC368603, TC371970 |
| [Organellar small ribosomal subunit](http://amigo.geneontology.org/cgi-bin/amigo/go.cgi?action=query&view=query&query=GO:0000314&search_constraint=terms) | TC396451, TC368603, TC371970 |
| [Small nuclear ribonucleoprotein complex](http://amigo.geneontology.org/cgi-bin/amigo/go.cgi?action=query&view=query&query=GO:0030532&search_constraint=terms) | TC369628, TC387135, TC387410, TC405540, TC387710, TC388566, TC385233, TC456230, TC376220, TC404606 |
| [Cell-cell junction](http://amigo.geneontology.org/cgi-bin/amigo/go.cgi?action=query&view=query&query=GO:0005911&search_constraint=terms) | TC387135, TC459656, TC398730, TC406807, TC370315, TC378790 |
| [U4/U6 x U5 tri-snRNP complex](http://amigo.geneontology.org/cgi-bin/amigo/go.cgi?action=query&view=query&query=GO:0046540&search_constraint=terms) | TC387135, TC385233, TC387410, TC405540, TC387710, TC404606, TC388566 |
| [Golgi-associated vesicle membrane](http://amigo.geneontology.org/cgi-bin/amigo/go.cgi?action=query&view=query&query=GO:0030660&search_constraint=terms) | TC385515, TC409650 |
| [Rough endoplasmic reticulum membrane](http://amigo.geneontology.org/cgi-bin/amigo/go.cgi?action=query&view=query&query=GO:0030867&search_constraint=terms) | TC409343, TC390402, TC412569 |
| [Axolemma](http://amigo.geneontology.org/cgi-bin/amigo/go.cgi?action=query&view=query&query=GO:0030673&search_constraint=terms) | TC403588, TC461622, TC380882 |
| [Leading edge membrane](http://amigo.geneontology.org/cgi-bin/amigo/go.cgi?action=query&view=query&query=GO:0031256&search_constraint=terms) | TC403588, TC461622, TC380882 |
| [Neuron projection membrane](http://amigo.geneontology.org/cgi-bin/amigo/go.cgi?action=query&view=query&query=GO:0032589&search_constraint=terms) | TC403588, TC461622, TC380882 |
| [Main axon](http://amigo.geneontology.org/cgi-bin/amigo/go.cgi?action=query&view=query&query=GO:0044304&search_constraint=terms) | TC403588, TC461622, TC380882 |
| [Condensed chromosome kinetochore](http://amigo.geneontology.org/cgi-bin/amigo/go.cgi?action=query&view=query&query=GO:0000777&search_constraint=terms) | TC394796, TC370315 |
| [Sarcomere](http://amigo.geneontology.org/cgi-bin/amigo/go.cgi?action=query&view=query&query=GO:0030017&search_constraint=terms) | TC459656, TC406807, TC410126, CJ944525 |
| [Nuclear lamina](http://amigo.geneontology.org/cgi-bin/amigo/go.cgi?action=query&view=query&query=GO:0005652&search_constraint=terms) | TC423804, TC377061 |
| [Plastid](http://amigo.geneontology.org/cgi-bin/amigo/go.cgi?action=query&view=query&query=GO:0009536&search_constraint=terms) | TC410352, TC441536, TC386040, TC370791, TC407978, TC390393, TC390516, TC453487, TC391946, TC417106, TC374098, CA710880, TC452945, TC457112, TC421871, TC375593, TC431932, TC376351, TC430471, TC439423, TC423252, TC397660, TC379171, TC390379, TC393781, TC417260, TC380063, TC433844, TC418414, CK209294, TC378054, TC453207, TC378615, TC373702, TC395453, TC409843, TC407076, TC445939, TC403872, CJ550278, BE591166, TC389586, GH732878, BE431108, CA632212, TC398731, CK217367, TC378153, TC387817, TC379990, TC378333, TC435863, BQ788843, TC426907, TC395723, TC369415, TC379536, TC389718, TC386707, TC381943, TC389534, TC420358, TC398379, BQ607161, CJ815867, TC402308, TC400260, TC378790, TC399057, TC408191, TC388317, TC389843, AL827131, TC416801, TC385009, CV759879, TC380474, TC391438, TC371325, TC432369, TC370619, TC422007, TC392778, TC405030, TC388403, TC373678, TC413118, TC406708, TC374461, TC391360, TC416471, TC410569, CN010359, TC458205, TC369687, TC373001, TC402603, TC433557, TC397909, CA729339, TC415803, TC430163, TC410108, TC389993, TC426864, TC391156, CA613620, TC400362, TC428992, TC398154, TC420816, TC369325, TC429747, TC384162, CJ867263, TC432185, TC377749, TC455736, TC375665, TC385205, TC427986, TC422645, BG904091, TC372677, TC421162, TC374240, TC405982, TC398121, TC393814, TC420460, TC373402, TC446235, TC427006, TC380943, TC393830, TC376202, TC444899, CV772029, TC398606, TC387319, TC396134, TC369182, TC418365, TC389090, TC389139, TC370347, TC373002, TC382655, CK200433, TC373994, TC393970, TC395351, TC452494, TC444138, CK162413, TC411908, CA598430, TC377373, TC376930, TC403852, TC412205, TC393437, TC391143, TC414090, TC392203, TC377190, TC443814, TC423110, TC391613, TC430544, DR737360, TC379357, TC371003, TC378437, TC404850, TC426016, TC390436, TC393960, TC385326, TC413124, TC443387, TC399245, TC373429, TC390630, TC386535, TC418716, TC392447, TC386519, TC383047, TC374726, TC385090, TC407964, TC394307, TC373583, TC382139, TC389589, TC370106, TC452762, TC405475, TC386344, TC422022, TC373637, TC386422, TC374164, TC400379, TC393037, TC426078, TC388822, TC429771, TC421733, TC386639, TC396228, TC416658, TC384678, TC379965, TC379944, TC404053, TC375834, TC400562, TC393948, TC375726, TC400638, TC393554, TC407614, TC384049, TC459656, TC384357, TC379436, TC411941, TC394118, TC449043, TC392247, TC426870, TC387007, TC375935, TC433589, TC389816, TC371172, TC390211, TC439472, TC372167, TC393426, TC400056, TC405033, TC382910, TC458892, DR740372, TC394693, TC416495, TC440526, TC436944, TC371341, TC428944, TC381279, TC396657, TC449093, TC379942, TC380333, TC377064, TC397258, TC426838, BQ295499, TC431330, TC394965, TC399271, TC422606, TC376937, TC398862, TC433162, TC389363, TC434442, TC371248, TC413263, TC431660, TC378878, TC378072, TC388950, TC406516, TC419222, TC392875, TC409005, TC371145, TC418728, TC390569, TC419057, CA724903, TC424044, TC401915, TC392432, TC386440, TC423239, TC425821, CV779166, DR739994, TC392358, TC391962, TC387501, TC374852, TC389092, TC376800, TC379853, TC420248, TC405440, TC414899, TC370885, TC407232, TC436309, TC440819, TC398514, TC393015, TC426980, TC392033, TC380825, TC447694, TC404620, TC406807, TC441239, TC416906, TC434820, TC379711, TC379903, TC416438, TC392272, TC417388, TC413339, TC404179, TC408907, TC375539, TC381191, TC369010, TC402080, TC370392, TC373518, CV761547, TC370350, TC411116, TC383909, TC374392, TC385780, TC369983, TC395566, TC369199, TC373489, CA676115, TC405896, TC402186, TC385927, TC385513, TC439501, TC444586, TC429062, TC403264, TC369348, TC400484, TC435825, CK214224, TC388232, TC398040, TC445281, TC409187, TC429374, TC384010, TC375253, TC401124, TC444302, TC409190, TC394850, TC384344, TC379422, TC418399, TC387683, TC409855, TC434831, TC426326, TC427161, TC373914, TC445649, TC404998, TC369899, CA498496, TC372330, TC381068, CD930656, TC435533, TC444375, TC429437, TC388914, TC371738, TC387621, TC433488, TC377438, TC398633, TC394716, TC456160, TC397027, TC369064, TC377292, TC380433, TC432154, TC420735, TC451621, TC435281, TC404371, TC427405, TC384219, TC449256, TC373615, TC383949, TC397562, TC416685, TC451342, TC372664, TC428853, TC389375, TC399986, TC404413, TC433812, TC390319, TC406038, TC376874, TC424154, TC372046, TC376490, TC384454, TC414564, TC411287, TC388751, TC407395, AL820497, TC431722, TC380416, TC399972, TC434396, TC398536, TC386414, CJ854725, TC440636, TC370633, TC386279, TC387170, TC382786, TC439225, TC419727, TC394263, TC436772, TC418340, TC405773, TC394253, TC389162, DR739471, TC399432, TC389678, TC391840, TC390285, CA727263, TC379241, CV065343, TC402440, TC373962, TC375146, TC375813, TC454407, TC379343, TC374741, TC389044, TC381963, TC383176, TC371037, TC449463, TC374879, TC385701, TC388688, TC408095, TC452503, TC429048, TC368549, TC377496, TC388976, TC379069, TC401158, TC423576, TC377635, TC415942, TC402668, TC393561, TC435799, TC393692, TC391671, TC373787, TC397415, BQ160837, TC438243, TC370507, TC396487, TC458699, TC400592, TC451285, TC458503, TC400721, TC391865, TC441995, TC445214, TC451519, CK211469, TC416981, TC449739, BE586004, TC399408, TC426743, TC396895, TC436290, TC382342, TC393106, TC397676, TC396460, TC431306, TC432504, TC379968 |
| [Kinetochore](http://amigo.geneontology.org/cgi-bin/amigo/go.cgi?action=query&view=query&query=GO:0000776&search_constraint=terms) | TC394796, TC370315 |
| [Lysosome](http://amigo.geneontology.org/cgi-bin/amigo/go.cgi?action=query&view=query&query=GO:0005764&search_constraint=terms) | TC372530, TC373613, TC432185 |
| [Rough endoplasmic reticulum](http://amigo.geneontology.org/cgi-bin/amigo/go.cgi?action=query&view=query&query=GO:0005791&search_constraint=terms) | TC409343, TC390402, TC412569 |
| [Myosin complex](http://amigo.geneontology.org/cgi-bin/amigo/go.cgi?action=query&view=query&query=GO:0016459&search_constraint=terms) | TC459656, TC406807, TC410126, CK199175, CJ944525 |
| [Organelle](http://amigo.geneontology.org/cgi-bin/amigo/go.cgi?action=query&view=query&query=GO:0043226&search_constraint=terms) | TC461921, BJ284275, TC410352, TC390944, TC370791, TC389190, TC453487, TC375864, TC432212, TC415588, TC389842, BQ838511, TC398052, TC387861, TC391749, TC374098, TC400388, TC457112, TC431932, CK215979, TC376351, TC430471, TC439423, TC383028, TC379171, TC388410, BJ282766, CK203550, TC416493, TC388136, TC458562, TC393436, TC417260, CK214103, TC433844, CK209294, TC410063, TC421143, TC386396, TC378615, TC373702, TC448471, TC456784, TC395453, TC392329, TC410182, TC445939, TC425847, CJ727624, CA614761, CJ550278, BE591166, TC411128, GH732878, TC397444, TC412569, TC415483, CA632212, TC393523, TC435852, TC413027, TC372549, TC394820, TC398731, TC418073, TC411784, TC417308, TC387817, TC395069, TC413460, TC411008, BQ609416, CK214702, TC426907, TC395723, TC369415, TC387981, TC385233, TC391950, TC398730, TC386707, TC376248, BE217043, TC374409, TC402069, TC381943, TC432001, TC420358, CV770684, TC418928, TC398209, BQ607161, TC372845, TC382045, TC431175, TC402308, TC378790, TC417077, TC385009, CV759879, TC376625, TC390016, TC380474, CA606693, TC370912, TC432369, TC385714, TC412212, TC375124, TC392778, TC397312, TC405030, CA615187, TC405041, TC444549, BJ317882, TC406469, TC399919, TC407572, TC413118, TC406708, TC373898, TC418691, TC409459, TC420337, TC391360, TC416471, TC419919, CV763657, TC372761, TC406809, TC410569, TC437137, CN010359, TC373001, TC402603, TC449724, TC395914, TC372867, TC397909, CA729339, BQ239045, TC430163, TC389993, TC426864, TC408312, TC391156, CA613620, TC400362, TC398154, TC401293, TC369325, TC429747, TC375612, TC430501, CJ867263, TC400108, TC406106, TC381462, CD882425, TC377749, TC396451, TC431198, TC424344, TC429217, TC372677, CJ868963, TC374240, TC376306, TC425841, TC405982, TC387579, TC420460, TC428008, TC427006, TC380943, TC376202, TC403885, TC405946, TC460907, TC394451, TC395312, CV772029, TC387344, TC389090, TC389139, TC412483, TC382655, TC389144, TC456230, TC373994, TC395351, TC392709, TC372563, TC418032, CK162413, TC397291, TC461977, TC411908, CA598430, TC459008, TC380329, TC376930, TC391516, TC409343, TC389696, TC403852, TC382742, TC377190, TC443814, TC431879, TC423110, TC422348, TC391613, TC385365, TC430544, TC382339, TC379357, TC371003, TC387135, TC378437, TC438587, TC375760, TC381923, TC426016, DR734732, TC387686, TC373251, TC413124, TC388566, TC399245, TC373429, GH729256, TC418716, TC392447, TC421467, TC379023, TC386519, TC394219, TC383047, TC417992, TC385090, TC407964, TC394307, TC388665, TC373583, TC406371, TC382139, TC392363, TC381988, TC370106, TC388819, TC405475, TC386401, TC422022, TC386344, TC452356, TC373637, TC370044, TC421872, TC386422, TC442623, CJ776780, TC422583, TC400379, TC414606, TC393037, CJ730226, TC421733, TC383570, TC396228, TC379965, TC384678, TC456370, TC404053, TC400562, TC371970, TC375726, TC459656, TC378783, TC379436, TC377765, TC403929, TC457991, TC394118, TC373848, TC426870, TC437918, TC387007, TC375935, TC418091, TC389816, TC372530, TC392323, TC370907, TC384194, TC413066, TC371172, TC390211, TC372167, TC391641, TC461622, TC393426, TC400056, TC405033, TC379470, TC419090, TC385659, TC382910, TC432678, TC389451, TC458892, DR740372, TC394693, TC416495, TC401758, TC428944, TC417067, TC449093, TC379942, TC434821, TC374404, TC396230, BQ295499, TC377766, TC394965, TC368599, TC391648, TC376937, TC433162, TC400317, TC404978, TC389363, TC371248, TC379338, TC433116, TC409167, TC416442, TC374679, TC387410, TC378072, TC397176, TC406516, TC406986, TC371145, TC435546, TC419057, TC451950, TC380695, TC398714, TC381619, TC424179, TC394028, TC398970, TC457126, TC456854, TC403679, TC418747, TC440803, TC403580, TC413427, TC386305, TC424044, TC372667, TC433957, TC392432, TC386440, TC423239, TC425821, TC370962, TC403055, TC394206, TC406236, TC382080, TC391962, TC391128, TC445767, TC387501, TC446038, TC374852, TC369830, TC423804, TC418685, CK213497, TC414899, TC436309, TC398514, TC391900, TC392033, TC423091, TC447694, TC380825, TC412520, TC406807, TC395090, TC404606, TC441239, TC416906, TC416438, CA595837, TC423265, TC417388, TC440066, TC386313, CK208222, TC404179, TC389108, TC369844, TC420579, CK211707, TC392126, TC402340, TC431201, CK217689, TC370392, CV761547, TC396751, CK211589, TC411116, TC388718, TC383701, TC452793, TC385780, TC405784, TC395566, TC372175, TC382734, TC405896, TC385927, TC369072, TC385513, TC439501, TC405356, TC444586, TC449448, TC377441, TC381596, TC403264, TC369348, TC461971, TC435825, TC422176, TC388232, TC445281, TC376263, TC415521, TC384010, TC416169, TC409043, TC408229, TC377290, TC409190, TC403537, TC381255, CV781430, TC379924, TC418399, TC431382, TC387683, TC409855, TC434831, TC406194, TC427161, TC373914, TC450285, TC392303, TC382024, TC404998, TC369899, TC372330, TC448051, TC381068, CD930656, TC403936, CF554444, TC451276, TC444375, TC387050, TC415685, TC388914, TC415056, TC433488, TC377178, TC377438, TC394716, TC397027, TC456160, TC394670, TC378271, TC377292, TC380433, TC451621, TC435281, TC384219, TC397562, TC428853, TC404413, TC408192, CJ626653, TC372046, CK210754, TC407191, TC370727, TC417769, TC449235, TC410147, TC414564, TC405910, TC431603, TC393904, TC387191, TC407395, TC447801, TC380416, TC427981, CA500690, TC398536, TC400755, TC403717, TC372701, TC405615, TC376420, TC458987, TC404843, TC382737, TC370633, TC390135, TC411480, TC417755, TC420394, TC432320, TC387170, TC439225, TC380662, TC419727, TC394263, TC436772, TC395298, TC394253, TC412094, CV782550, TC389162, TC376630, CK212850, TC389678, TC399432, TC434006, TC434949, CA727263, TC379241, TC405295, TC392465, TC455676, TC373962, TC391411, TC409208, TC375813, TC379343, TC374741, TC429809, TC386591, TC421704, TC420201, TC381963, TC456247, TC371455, TC374879, GH728664, TC385701, TC400181, TC418759, TC431825, TC377496, TC368549, TC401158, TC387133, TC410078, TC415942, TC393561, TC393692, TC391671, TC373787, BQ160837, TC382928, TC397415, TC370507, TC369637, TC396487, TC436832, TC458699, TC380882, TC440321, TC372580, TC444588, TC377410, TC400721, TC451657, TC445214, TC451519, TC393820, TC435808, TC449739, TC416981, BE586004, TC397729, TC399408, TC417970, TC426743, TC396895, TC382342, TC393106, TC428932, TC431306, TC396460, TC397676, TC379968, TC399730, TC368580, TC441536, TC373613, TC386040, TC407978, TC390393, TC444546, TC392283, TC390516, TC416529, TC391946, TC460760, TC420420, TC408524, TC417106, TC432124, CA710880, TC452945, TC375593, TC421871, TC371242, TC394459, TC423252, TC384738, TC416154, TC397660, CV766916, TC390379, TC407404, TC374996, TC393781, TC421914, TC435210, TC398538, TC380063, TC418414, CJ944525, TC396772, TC378054, TC453207, TC419273, TC461607, TC409843, TC437445, TC403872, TC407076, TC371357, TC406207, TC389586, TC395746, TC439210, TC398304, TC393100, TC425878, CV771134, BE431108, TC417012, TC391447, TC394916, TC404914, TC391130, TC378274, CK217367, TC378153, TC379990, TC378333, TC444410, TC435863, BQ788843, TC452050, CJ845228, TC379536, TC391621, TC404052, TC389718, TC429713, CK201148, TC386088, TC389534, TC413392, TC422142, TC398379, TC376758, CJ815867, TC369628, TC441343, TC400260, TC400330, TC399057, TC408191, TC378432, TC377061, TC389843, TC388317, CD937281, TC416801, AL827131, TC446465, TC379312, TC391438, TC381268, TC412317, TC371325, TC439708, TC428113, TC373563, TC370619, BJ258087, TC421345, TC422007, TC370178, TC405695, TC388403, TC373678, TC416695, TC410690, TC374461, TC410074, TC443289, TC386646, TC399841, TC369687, TC458205, TC441241, TC433557, TC413571, TC415803, TC408299, TC410108, TC454526, TC398830, TC399352, TC422425, TC371129, TC398805, TC372654, TC428992, TC369092, TC420816, TC384162, TC395872, TC383739, TC399342, TC432185, TC381583, TC455736, TC392263, TC432130, TC375665, TC385205, TC427986, TC422645, TC445166, BG904091, TC421162, TC398121, TC389168, TC424204, TC422288, TC406870, TC424317, TC393814, TC376220, TC373402, TC404926, TC446235, TC402545, TC393830, TC444899, TC386961, TC398606, TC387319, TC396134, TC369182, TC418365, TC370347, TC373002, TC375554, CK200433, TC393970, TC374731, TC452494, TC456619, TC388228, TC444138, TC377373, EB512907, TC390994, TC393437, TC412205, TC426916, TC391143, TC381463, TC403157, TC410066, TC414090, TC395303, TC442140, TC405511, TC386410, TC392203, TC393890, TC442727, TC390762, TC401210, DR737360, TC381007, TC397885, TC411684, TC382728, TC388049, TC404850, TC370033, TC433940, TC374485, TC393960, TC390436, TC426358, TC385326, TC387707, TC409077, TC443387, TC415083, TC370470, TC386535, TC390630, TC390402, TC374726, TC417341, TC415954, TC376790, TC398592, TC389589, TC378911, TC452762, TC407490, CD878039, TC374164, TC418845, TC377993, TC438657, DR735108, TC412150, TC426078, TC388822, TC429771, TC386639, TC375069, TC416658, TC424962, TC379944, TC374230, TC391913, TC375834, TC393948, TC403624, TC400638, TC393554, TC407614, TC384049, TC384357, TC391007, TC460615, TC403573, TC411941, TC385445, TC395841, TC449043, TC379635, TC392247, TC373259, TC433589, TC382338, TC375914, TC398343, TC377253, TC404426, TC439472, TC377944, TC381817, TC403588, TC394728, TC403803, TC418850, TC440526, TC436944, TC371341, TC381279, TC389661, CA726837, TC387386, TC383820, TC396657, TC380333, TC377064, TC397258, TC426838, CV775873, TC451511, TC431330, TC384373, TC416069, TC427210, TC422606, TC399271, TC375313, TC398862, DR739303, TC434442, TC413263, TC411471, TC431660, TC434570, TC378878, TC406264, TC390792, TC394020, TC388950, TC374669, TC419222, TC410194, TC369371, TC448840, TC392875, TC409005, TC408228, TC384735, TC418728, TC405540, TC390569, TC391948, BQ294582, TC401915, CA724903, CV779166, DR739994, TC459193, TC395506, TC395672, TC450972, TC392358, TC409599, TC419165, TC389092, TC376800, TC379853, TC420248, TC370315, TC449504, TC403687, TC405440, TC460795, CJ792862, TC422789, TC370885, TC407232, TC440819, TC416492, TC393015, TC426980, TC377225, TC404620, TC382858, TC403977, TC429865, TC434820, TC379711, TC379903, TC377308, TC406749, TC392272, TC401244, CA730421, TC400114, TC413339, TC408907, TC375539, TC385013, TC380083, TC386963, TC412732, TC381191, TC369010, TC402080, TC373518, TC370350, TC383909, TC376527, TC374940, TC419584, CN010697, TC374392, TC378568, TC369983, CV761628, TC404420, TC383235, TC373489, TC369199, TC419747, TC407304, CA676115, TC402186, BE415178, TC429062, TC423354, TC409344, TC413043, BJ243383, TC400484, CK214224, TC368603, TC398040, TC374964, TC375234, CA709177, TC385815, TC409187, TC429374, TC425690, TC375253, TC423182, TC381218, TC401124, TC375873, TC444302, TC435224, CJ563078, TC394850, TC384344, TC392297, TC388520, TC443712, TC379422, TC378956, TC411191, TC426326, TC375431, TC424252, TC391995, TC415501, TC422841, TC387064, TC445649, CA498496, TC394796, TC382777, TC435533, TC407349, TC397994, TC414243, TC403234, TC429437, TC430988, TC371738, TC413771, TC387621, TC398633, TC460689, TC380125, TC410126, TC369064, TC432154, TC420735, TC404371, TC402939, TC427405, TC449256, TC373615, TC383949, TC378409, TC416685, TC451342, TC372664, TC399986, TC389375, TC433812, TC370114, TC406038, TC390319, TC376874, TC424154, TC458807, TC459245, TC429871, TC369928, TC419438, TC376490, TC384454, TC411287, TC386237, TC388751, TC431722, AL820497, TC435909, TC399471, TC408126, TC430096, TC399972, TC434396, TC421880, TC386414, TC389590, CJ854725, TC440636, TC403986, TC406193, TC383763, CD876572, TC369736, TC386279, CK197833, TC391785, TC377427, TC382786, TC409650, TC388158, TC418340, TC405773, TC382830, TC377653, DR739471, TC402534, TC382129, TC391840, TC390285, TC412989, TC387766, TC407340, TC398026, TC406584, CV065343, TC402440, TC396636, TC375146, TC454407, CK199175, TC394661, TC389044, TC383176, TC449463, TC371037, TC388688, TC403328, TC408095, TC439939, TC375268, TC452503, TC388547, TC420877, TC429048, TC398978, TC388976, TC396365, TC379069, TC385526, TC423576, TC375530, TC377635, TC402668, TC435799, TC438243, TC392887, TC370210, TC375918, TC417363, TC400592, TC413700, TC387710, TC385515, TC423880, TC451285, BG263159, TC458503, TC387194, TC391865, TC434689, TC441995, CK211469, CB307332, TC404842, TC381296, TC411817, TC436290, TC390489, TC421341, TC432504, TC383143 |
| [Intracellular organelle](http://amigo.geneontology.org/cgi-bin/amigo/go.cgi?action=query&view=query&query=GO:0043229&search_constraint=terms) | TC461921, BJ284275, TC410352, TC390944, TC370791, TC389190, TC453487, TC375864, TC432212, TC415588, TC389842, BQ838511, TC398052, TC387861, TC391749, TC374098, TC400388, TC457112, TC431932, CK215979, TC376351, TC430471, TC439423, TC383028, TC379171, TC388410, BJ282766, CK203550, TC416493, TC388136, TC458562, TC393436, TC417260, CK214103, TC433844, CK209294, TC410063, TC421143, TC386396, TC378615, TC373702, TC448471, TC456784, TC395453, TC392329, TC410182, TC445939, TC425847, CJ727624, CA614761, CJ550278, BE591166, TC411128, GH732878, TC397444, TC412569, TC415483, CA632212, TC393523, TC435852, TC413027, TC372549, TC394820, TC398731, TC418073, TC411784, TC417308, TC387817, TC395069, TC413460, TC411008, BQ609416, CK214702, TC426907, TC395723, TC369415, TC387981, TC385233, TC391950, TC398730, TC386707, TC376248, BE217043, TC374409, TC402069, TC381943, TC432001, TC420358, CV770684, TC418928, TC398209, BQ607161, TC372845, TC382045, TC431175, TC402308, TC378790, TC417077, TC385009, CV759879, TC376625, TC390016, TC380474, CA606693, TC370912, TC432369, TC385714, TC412212, TC375124, TC392778, TC397312, TC405030, CA615187, TC405041, TC444549, BJ317882, TC406469, TC399919, TC407572, TC413118, TC406708, TC373898, TC418691, TC409459, TC420337, TC391360, TC416471, TC419919, CV763657, TC372761, TC406809, TC410569, TC437137, CN010359, TC373001, TC402603, TC449724, TC395914, TC372867, TC397909, CA729339, BQ239045, TC430163, TC389993, TC426864, TC408312, TC391156, CA613620, TC400362, TC398154, TC401293, TC369325, TC429747, TC375612, TC430501, CJ867263, TC400108, TC406106, TC381462, CD882425, TC377749, TC396451, TC431198, TC424344, TC429217, TC372677, CJ868963, TC374240, TC376306, TC425841, TC405982, TC387579, TC420460, TC428008, TC427006, TC380943, TC376202, TC403885, TC405946, TC460907, TC394451, TC395312, CV772029, TC387344, TC389090, TC389139, TC412483, TC382655, TC389144, TC456230, TC373994, TC395351, TC392709, TC372563, TC418032, CK162413, TC397291, TC461977, TC411908, CA598430, TC459008, TC380329, TC376930, TC391516, TC409343, TC389696, TC403852, TC382742, TC377190, TC443814, TC431879, TC423110, TC422348, TC391613, TC385365, TC430544, TC382339, TC379357, TC371003, TC387135, TC378437, TC438587, TC375760, TC381923, TC426016, DR734732, TC387686, TC373251, TC413124, TC388566, TC399245, TC373429, GH729256, TC418716, TC392447, TC421467, TC379023, TC386519, TC394219, TC383047, TC417992, TC385090, TC407964, TC394307, TC388665, TC373583, TC406371, TC382139, TC392363, TC381988, TC370106, TC388819, TC405475, TC386401, TC422022, TC386344, TC452356, TC373637, TC370044, TC421872, TC386422, TC442623, CJ776780, TC422583, TC400379, TC414606, TC393037, CJ730226, TC421733, TC383570, TC396228, TC379965, TC384678, TC456370, TC404053, TC400562, TC371970, TC375726, TC459656, TC378783, TC379436, TC377765, TC403929, TC457991, TC394118, TC373848, TC426870, TC437918, TC387007, TC375935, TC418091, TC389816, TC372530, TC392323, TC370907, TC384194, TC413066, TC371172, TC390211, TC372167, TC391641, TC461622, TC393426, TC400056, TC405033, TC379470, TC419090, TC385659, TC382910, TC432678, TC389451, TC458892, DR740372, TC394693, TC416495, TC401758, TC428944, TC417067, TC449093, TC379942, TC434821, TC374404, TC396230, BQ295499, TC377766, TC394965, TC368599, TC391648, TC376937, TC433162, TC400317, TC404978, TC389363, TC371248, TC379338, TC433116, TC409167, TC416442, TC374679, TC387410, TC378072, TC397176, TC406516, TC406986, TC371145, TC435546, TC419057, TC451950, TC380695, TC398714, TC381619, TC424179, TC394028, TC398970, TC457126, TC456854, TC403679, TC418747, TC440803, TC403580, TC413427, TC386305, TC424044, TC372667, TC433957, TC392432, TC386440, TC423239, TC425821, TC370962, TC403055, TC394206, TC406236, TC382080, TC391962, TC391128, TC445767, TC387501, TC446038, TC374852, TC369830, TC423804, TC418685, CK213497, TC414899, TC436309, TC398514, TC391900, TC392033, TC423091, TC447694, TC380825, TC412520, TC406807, TC395090, TC404606, TC441239, TC416906, TC416438, CA595837, TC423265, TC417388, TC440066, TC386313, CK208222, TC404179, TC389108, TC369844, TC420579, CK211707, TC392126, TC402340, TC431201, CK217689, TC370392, CV761547, TC396751, CK211589, TC411116, TC388718, TC383701, TC452793, TC385780, TC405784, TC395566, TC372175, TC382734, TC405896, TC385927, TC369072, TC385513, TC439501, TC405356, TC444586, TC449448, TC377441, TC381596, TC403264, TC369348, TC461971, TC435825, TC422176, TC388232, TC445281, TC376263, TC415521, TC384010, TC416169, TC409043, TC408229, TC377290, TC409190, TC403537, TC381255, CV781430, TC379924, TC418399, TC431382, TC387683, TC409855, TC434831, TC406194, TC427161, TC373914, TC450285, TC392303, TC382024, TC404998, TC369899, TC372330, TC448051, TC381068, CD930656, TC403936, CF554444, TC451276, TC444375, TC387050, TC415685, TC388914, TC415056, TC433488, TC377178, TC377438, TC394716, TC397027, TC456160, TC394670, TC378271, TC377292, TC380433, TC451621, TC435281, TC384219, TC397562, TC428853, TC404413, TC408192, CJ626653, TC372046, CK210754, TC407191, TC370727, TC417769, TC449235, TC410147, TC414564, TC405910, TC431603, TC393904, TC387191, TC407395, TC447801, TC380416, TC427981, CA500690, TC398536, TC400755, TC403717, TC372701, TC405615, TC376420, TC458987, TC404843, TC382737, TC370633, TC390135, TC411480, TC417755, TC420394, TC432320, TC387170, TC439225, TC380662, TC419727, TC394263, TC436772, TC395298, TC394253, TC412094, CV782550, TC389162, TC376630, CK212850, TC389678, TC399432, TC434006, TC434949, CA727263, TC379241, TC405295, TC392465, TC455676, TC373962, TC391411, TC409208, TC375813, TC379343, TC374741, TC429809, TC386591, TC421704, TC420201, TC381963, TC456247, TC371455, TC374879, GH728664, TC385701, TC400181, TC418759, TC431825, TC377496, TC368549, TC401158, TC387133, TC410078, TC415942, TC393561, TC393692, TC391671, TC373787, BQ160837, TC382928, TC397415, TC370507, TC369637, TC396487, TC436832, TC458699, TC380882, TC440321, TC372580, TC444588, TC377410, TC400721, TC451657, TC445214, TC451519, TC393820, TC435808, TC449739, TC416981, BE586004, TC397729, TC399408, TC417970, TC426743, TC396895, TC382342, TC393106, TC428932, TC431306, TC396460, TC397676, TC379968, TC399730, TC368580, TC441536, TC373613, TC386040, TC407978, TC390393, TC444546, TC392283, TC390516, TC416529, TC391946, TC460760, TC420420, TC408524, TC417106, TC432124, CA710880, TC452945, TC375593, TC421871, TC371242, TC394459, TC423252, TC384738, TC416154, TC397660, CV766916, TC390379, TC407404, TC374996, TC393781, TC421914, TC435210, TC398538, TC380063, TC418414, CJ944525, TC396772, TC378054, TC453207, TC419273, TC461607, TC409843, TC437445, TC403872, TC407076, TC371357, TC406207, TC389586, TC395746, TC439210, TC398304, TC393100, TC425878, CV771134, BE431108, TC417012, TC391447, TC394916, TC404914, TC391130, TC378274, CK217367, TC378153, TC379990, TC378333, TC444410, TC435863, BQ788843, TC452050, CJ845228, TC379536, TC391621, TC404052, TC389718, TC429713, CK201148, TC386088, TC389534, TC413392, TC422142, TC398379, TC376758, CJ815867, TC369628, TC441343, TC400260, TC400330, TC399057, TC408191, TC378432, TC377061, TC389843, TC388317, CD937281, TC416801, AL827131, TC446465, TC379312, TC391438, TC381268, TC412317, TC371325, TC439708, TC428113, TC373563, TC370619, BJ258087, TC421345, TC422007, TC370178, TC405695, TC388403, TC373678, TC416695, TC410690, TC374461, TC410074, TC443289, TC386646, TC399841, TC369687, TC458205, TC441241, TC433557, TC413571, TC415803, TC408299, TC410108, TC454526, TC398830, TC399352, TC422425, TC371129, TC398805, TC372654, TC428992, TC369092, TC420816, TC384162, TC395872, TC383739, TC399342, TC432185, TC381583, TC455736, TC392263, TC432130, TC375665, TC385205, TC427986, TC422645, TC445166, BG904091, TC421162, TC398121, TC389168, TC424204, TC422288, TC406870, TC424317, TC393814, TC376220, TC373402, TC404926, TC446235, TC402545, TC393830, TC444899, TC386961, TC398606, TC387319, TC396134, TC369182, TC418365, TC370347, TC373002, TC375554, CK200433, TC393970, TC374731, TC452494, TC456619, TC388228, TC444138, TC377373, EB512907, TC390994, TC393437, TC412205, TC426916, TC391143, TC381463, TC403157, TC410066, TC414090, TC395303, TC442140, TC405511, TC386410, TC392203, TC393890, TC442727, TC390762, TC401210, DR737360, TC381007, TC397885, TC411684, TC382728, TC388049, TC404850, TC370033, TC433940, TC374485, TC393960, TC390436, TC426358, TC385326, TC387707, TC409077, TC443387, TC415083, TC370470, TC386535, TC390630, TC390402, TC374726, TC417341, TC415954, TC376790, TC398592, TC389589, TC378911, TC452762, TC407490, CD878039, TC374164, TC418845, TC377993, TC438657, DR735108, TC412150, TC426078, TC388822, TC429771, TC386639, TC375069, TC416658, TC424962, TC379944, TC374230, TC391913, TC375834, TC393948, TC403624, TC400638, TC393554, TC407614, TC384049, TC384357, TC391007, TC460615, TC403573, TC411941, TC385445, TC395841, TC449043, TC379635, TC392247, TC373259, TC433589, TC382338, TC375914, TC398343, TC377253, TC404426, TC439472, TC377944, TC381817, TC403588, TC394728, TC403803, TC418850, TC440526, TC436944, TC371341, TC381279, TC389661, CA726837, TC387386, TC383820, TC396657, TC380333, TC377064, TC397258, TC426838, CV775873, TC451511, TC431330, TC384373, TC416069, TC427210, TC422606, TC399271, TC375313, TC398862, DR739303, TC434442, TC413263, TC411471, TC431660, TC434570, TC378878, TC406264, TC390792, TC394020, TC388950, TC374669, TC419222, TC410194, TC369371, TC448840, TC392875, TC409005, TC408228, TC384735, TC418728, TC405540, TC390569, TC391948, BQ294582, TC401915, CA724903, CV779166, DR739994, TC459193, TC395506, TC395672, TC450972, TC392358, TC409599, TC419165, TC389092, TC376800, TC379853, TC420248, TC370315, TC449504, TC403687, TC405440, TC460795, CJ792862, TC422789, TC370885, TC407232, TC440819, TC416492, TC393015, TC426980, TC377225, TC404620, TC382858, TC403977, TC429865, TC434820, TC379711, TC379903, TC377308, TC406749, TC392272, TC401244, CA730421, TC400114, TC413339, TC408907, TC375539, TC385013, TC380083, TC386963, TC412732, TC381191, TC369010, TC402080, TC373518, TC370350, TC383909, TC376527, TC374940, TC419584, CN010697, TC374392, TC378568, TC369983, CV761628, TC404420, TC383235, TC373489, TC369199, TC419747, TC407304, CA676115, TC402186, BE415178, TC429062, TC423354, TC409344, TC413043, BJ243383, TC400484, CK214224, TC368603, TC398040, TC374964, TC375234, CA709177, TC385815, TC409187, TC429374, TC425690, TC375253, TC423182, TC381218, TC401124, TC375873, TC444302, TC435224, CJ563078, TC394850, TC384344, TC392297, TC388520, TC443712, TC379422, TC378956, TC411191, TC426326, TC375431, TC424252, TC391995, TC415501, TC422841, TC387064, TC445649, CA498496, TC394796, TC382777, TC435533, TC407349, TC397994, TC414243, TC403234, TC429437, TC430988, TC371738, TC413771, TC387621, TC398633, TC460689, TC380125, TC410126, TC369064, TC432154, TC420735, TC404371, TC402939, TC427405, TC449256, TC373615, TC383949, TC378409, TC416685, TC451342, TC372664, TC399986, TC389375, TC433812, TC370114, TC406038, TC390319, TC376874, TC424154, TC458807, TC459245, TC429871, TC369928, TC419438, TC376490, TC384454, TC411287, TC386237, TC388751, TC431722, AL820497, TC435909, TC399471, TC408126, TC430096, TC399972, TC434396, TC421880, TC386414, TC389590, CJ854725, TC440636, TC403986, TC406193, TC383763, CD876572, TC369736, TC386279, CK197833, TC391785, TC377427, TC382786, TC409650, TC388158, TC418340, TC405773, TC382830, TC377653, DR739471, TC402534, TC382129, TC391840, TC390285, TC412989, TC387766, TC407340, TC398026, TC406584, CV065343, TC402440, TC396636, TC375146, TC454407, CK199175, TC394661, TC389044, TC383176, TC449463, TC371037, TC388688, TC403328, TC408095, TC439939, TC375268, TC452503, TC388547, TC420877, TC429048, TC398978, TC388976, TC396365, TC379069, TC385526, TC423576, TC375530, TC377635, TC402668, TC435799, TC438243, TC392887, TC370210, TC375918, TC417363, TC400592, TC413700, TC387710, TC385515, TC423880, TC451285, BG263159, TC458503, TC387194, TC391865, TC434689, TC441995, CK211469, CB307332, TC404842, TC381296, TC411817, TC436290, TC390489, TC421341, TC432504, TC383143 |
| [Cell projection membrane](http://amigo.geneontology.org/cgi-bin/amigo/go.cgi?action=query&view=query&query=GO:0031253&search_constraint=terms) | TC403588, TC461622, TC380882 |
| [Golgi stack](http://amigo.geneontology.org/cgi-bin/amigo/go.cgi?action=query&view=query&query=GO:0005795&search_constraint=terms) | TC376490, TC411784, TC398343, TC369736 |
| [Cell](http://amigo.geneontology.org/cgi-bin/amigo/go.cgi?action=query&view=query&query=GO:0005623&search_constraint=terms) | TC461921, BJ284275, TC410352, TC390944, TC370791, TC389190, TC453487, TC375864, TC432212, TC415588, TC389842, BQ838511, TC398052, TC387861, TC391749, TC374098, TC400388, TC457112, TC431932, CK215979, TC376351, TC430471, TC439423, TC383028, TC379171, TC388410, BJ282766, CK203550, TC416493, TC388136, TC458983, TC458562, TC393436, TC417260, TC422842, CK214103, TC433844, CK209294, TC410063, TC421143, TC386396, TC378615, TC373702, TC448471, TC456784, TC395453, TC392329, TC410182, TC445939, TC425847, CJ727624, CA614761, CJ550278, BE591166, TC411128, GH732878, TC397444, TC412569, TC415483, CA632212, TC393523, TC435852, TC413027, TC372549, TC394820, TC398731, TC418073, TC411784, TC417308, TC387817, TC395069, TC413460, TC411008, BQ609416, CK214702, TC426907, TC395723, TC369415, TC387981, TC385233, TC391950, TC398730, TC386707, TC376248, BE217043, TC374409, TC402069, TC381943, TC432001, TC420358, CV770684, TC418928, TC398209, BQ607161, TC372845, TC382045, TC431175, TC402308, TC378790, TC417077, TC385009, CV759879, TC376625, TC390016, TC380474, CA606693, TC370912, TC432369, TC385714, TC412212, TC375124, TC392778, TC397312, TC405030, CA615187, TC405041, TC444549, TC397019, BJ317882, TC406469, TC399919, TC407572, TC413118, TC406708, TC373898, TC418691, TC409459, TC420337, TC391360, TC416471, TC419919, CV763657, TC372761, TC406809, TC410569, TC437137, CN010359, TC373001, TC402603, TC449724, TC395914, TC372867, TC397909, CA729339, TC423348, BQ239045, TC430163, TC389993, TC426864, TC408312, CV766349, TC391156, CA613620, TC400362, TC398154, TC401293, TC369325, TC429747, TC375612, TC430501, CJ867263, TC400108, TC406106, TC381462, CD882425, TC377749, TC396451, TC431198, TC424344, TC404158, CK201269, TC416381, TC429217, TC372677, CJ868963, TC374240, TC376306, TC425841, TC405982, TC387579, TC420460, TC428008, TC427006, TC380943, TC376202, TC403885, TC405946, TC460907, TC394451, TC395312, CV772029, TC387344, TC389090, TC389139, TC412483, TC382655, TC389144, TC456230, TC373994, TC395351, TC392709, TC372563, TC418032, CK162413, TC397291, TC461977, TC430555, TC411908, CA598430, TC459008, TC380329, TC376930, TC391516, TC409343, TC389696, TC403852, TC428066, TC382742, TC377190, TC443814, TC431879, TC423110, TC422348, TC391613, TC385365, TC430544, TC382339, TC379357, TC371003, TC387135, TC390566, TC378437, TC438587, TC375760, TC381923, TC426016, DR734732, TC387686, TC373251, TC413124, TC388566, TC378225, TC399245, TC373429, GH729256, TC418716, TC392447, TC421467, TC379023, TC386519, TC394219, TC383047, TC408381, TC417992, TC385090, TC407964, TC394307, TC388665, TC373583, TC406371, TC382139, TC392363, TC381988, TC370106, TC388819, TC405475, TC386401, TC422022, TC386344, TC452356, TC373637, TC370044, TC421872, TC386422, TC442623, CJ776780, TC422583, TC384071, TC400379, TC414606, TC393037, CJ730226, TC421733, TC383570, TC396228, TC379965, TC396650, TC384678, TC456370, TC404053, TC400562, TC371970, TC375726, TC391461, TC459656, DR734904, TC378783, TC379436, TC377765, TC440632, TC403929, TC457991, TC394118, TC378601, TC373848, TC426870, TC437918, TC387007, TC375935, TC418091, TC389816, TC371387, TC372530, TC392323, TC370907, TC384194, TC413066, TC371172, TC390211, TC446092, TC372167, TC391641, TC461622, TC393426, TC400056, TC405033, TC379470, TC419090, TC385659, TC376933, TC382910, TC432678, TC389451, TC458892, DR740372, TC394693, TC416495, TC401758, TC428944, TC417067, TC449093, TC379942, TC434821, TC374404, BQ295499, TC396230, TC377766, TC398383, TC394965, TC368599, TC391648, TC376937, TC433162, TC400317, TC404978, TC389363, TC371248, TC379338, TC433116, TC409167, TC416442, TC374679, TC387410, TC378072, TC397176, TC406516, TC406986, TC371145, TC435546, TC419057, TC451950, TC380695, TC398714, TC381619, TC424179, TC394028, TC398970, TC457126, TC456854, TC403679, TC418747, TC440803, TC403580, TC413427, TC386305, TC424044, TC372667, TC433957, TC386440, TC392432, TC423239, TC425821, TC370962, TC403055, TC394206, TC406236, TC382080, TC391962, TC391128, TC445767, TC387501, TC446038, TC374852, TC369830, TC423804, TC418685, CK213497, TC414899, TC436309, TC398514, TC391900, TC392033, TC423091, TC447694, TC380825, TC392269, TC412520, TC406807, TC395090, TC404606, TC441239, TC416906, TC416438, CA595837, TC423265, TC417388, TC440066, TC386313, CK208222, TC404179, TC435055, TC389108, TC369844, TC420579, CK211707, TC392126, TC402340, TC431201, CK217689, TC370392, CV761547, TC396751, CK211589, TC411116, TC388718, TC383701, TC452793, TC385780, TC405784, TC395566, TC372175, TC382734, TC405896, TC385927, TC369072, TC385513, TC439501, TC405356, TC444586, TC449448, TC377441, TC381596, TC403264, TC369348, TC461971, TC435825, TC422176, TC388232, TC445281, TC376263, TC415521, TC384010, TC416169, TC409043, TC408229, TC377290, TC409190, TC403537, TC381255, CV781430, TC379924, TC418399, TC431382, TC387683, TC409855, TC434831, TC406194, TC427161, TC373914, TC450285, TC392303, TC382024, TC404998, TC369899, TC378846, TC372330, TC448051, TC381068, CD930656, TC403936, CF554444, TC451276, TC444375, TC387050, TC415685, TC388914, TC415056, TC433488, TC377178, TC377438, TC394716, TC397027, TC456160, TC394670, TC378271, TC377292, TC380433, TC451621, TC435281, TC384219, TC397562, TC428853, TC404413, TC408192, CJ626653, TC372046, CK210754, TC407191, TC370727, TC417769, TC449235, TC410147, TC414564, TC405910, TC431603, TC393904, TC387191, TC407395, TC447801, TC380416, TC427981, CA500690, TC398536, TC400755, TC403717, TC372701, TC405615, TC376420, TC458987, TC404843, TC382737, TC370633, TC390135, TC411480, TC417755, TC420394, TC432320, TC387170, TC439225, TC380662, TC388649, TC419727, TC413462, TC394263, TC436772, TC395298, TC394253, TC412094, CV782550, TC389162, TC376630, CK212850, TC389678, TC399432, TC434006, TC434949, TC369726, CA727263, TC379241, TC405295, TC417480, TC392465, TC455676, TC373962, TC391411, TC409208, TC375813, TC379343, TC374741, TC429809, TC386591, TC421704, TC420201, TC381963, TC456247, TC371455, TC374879, GH728664, TC385701, TC400181, TC418759, TC431825, TC377496, TC368549, TC401158, TC387133, TC410078, TC415942, TC393561, TC393692, TC386322, TC391671, TC373787, BQ160837, TC382928, TC397415, TC370507, TC369637, TC396487, TC436832, TC458699, TC380882, TC440321, TC372580, TC444588, TC377410, TC400721, TC451657, TC445214, TC451519, TC393820, TC435808, TC449739, TC416981, BE586004, TC397729, TC399408, TC417970, TC426743, TC396895, TC382342, TC393106, TC428932, TC431306, TC396460, TC397676, TC379968, TC399730, TC368580, TC407456, TC441536, TC373613, TC386040, TC407978, TC390393, TC373958, TC444546, TC411284, TC392283, TC390516, TC416529, TC391946, TC460760, TC420420, TC383139, TC408524, TC417106, TC419614, TC432124, CA710880, TC452945, TC375593, TC421871, TC371242, TC394459, TC423252, TC384738, TC416154, TC397660, CV766916, TC407404, TC390379, TC374996, TC393781, TC421914, TC435210, TC380063, TC398538, TC418414, CJ944525, TC396772, TC378054, TC453207, TC419273, TC461607, TC409843, TC406594, TC437445, TC403872, TC407076, TC400595, TC371357, TC406207, TC389586, TC395746, TC439210, TC398304, TC393100, TC425878, CV771134, BE431108, TC417012, TC391447, TC455515, TC394916, TC397793, TC404914, TC391130, TC378274, CK217367, TC378153, TC379990, TC410144, TC378333, TC444410, TC435863, BQ788843, TC452050, CJ845228, TC379536, TC391621, TC404052, TC389718, TC429713, CK201148, TC386088, TC389534, TC413392, TC422142, TC398379, TC385783, TC376758, CJ815867, TC369628, TC441343, TC400260, TC400330, TC372796, TC399057, TC408191, TC378432, TC377061, TC389843, TC388317, CD937281, TC446465, TC416801, AL827131, TC379312, TC391438, TC381268, TC412317, TC371325, TC439708, TC428113, TC373563, TC370619, BJ258087, TC421345, TC422007, TC370178, TC405695, TC388403, TC373678, TC416695, TC410690, TC374461, TC410074, TC443289, TC386646, TC399841, TC369687, TC458205, TC441241, TC433557, TC413571, TC415803, TC408299, TC410108, TC454526, TC398830, TC399352, TC422425, TC371129, TC398805, TC372654, TC428992, TC369092, TC420816, TC384162, TC395872, TC383739, TC399342, TC432185, TC381583, TC455736, TC392263, TC432130, TC375665, TC385205, TC384625, TC427986, TC422645, TC425957, TC445166, BG904091, TC421162, TC429341, TC398121, TC389168, TC424204, TC439324, TC422288, TC406870, TC424317, TC393814, TC376220, TC373402, TC408309, TC404926, TC446235, TC402545, TC393830, TC415808, TC444899, TC386961, TC398606, TC387319, TC396134, TC369182, TC418365, TC373204, TC370347, TC373002, TC375554, CK200433, TC393970, TC374731, TC452494, TC456619, TC388228, TC444138, TC377373, TC415365, EB512907, TC390994, TC412205, TC393437, TC447260, TC426916, TC391143, TC381463, TC403157, TC410066, TC414090, TC395303, TC442140, TC405511, TC386410, TC392203, TC393890, TC442727, TC390762, TC401210, DR737360, TC381007, TC397885, TC411684, TC382728, TC388049, TC404850, TC370033, TC433940, TC374485, TC390436, TC393960, TC426358, TC385326, TC449064, TC387707, TC409077, TC443387, TC415083, TC370470, TC386535, TC390630, TC390402, TC374726, TC417341, TC415954, TC376790, TC398592, TC389589, TC378911, TC452762, TC407490, CD878039, TC374164, TC418845, TC377993, TC438657, DR735108, TC412150, TC426078, TC388822, TC429771, TC386639, TC375069, TC416658, TC424962, TC379944, TC374230, TC391913, TC375834, TC393948, TC403624, TC400638, TC393554, TC407614, TC400477, TC384049, TC384357, TC391007, TC460615, TC403573, TC411941, TC385445, TC395841, TC449043, TC379635, TC392247, TC373259, TC433589, TC382338, TC375914, TC398343, TC377253, TC404426, TC439472, TC377944, TC381817, TC403588, TC394728, TC403803, TC418850, TC440526, TC436944, TC371341, TC389661, TC381279, CA726837, TC387386, TC383820, TC396657, TC380333, TC377064, TC397258, TC426838, CV775873, TC451511, TC431330, TC384373, TC416069, TC427210, TC422606, TC399271, TC375313, TC398862, DR739303, TC381613, TC434442, TC413263, TC411471, TC431660, TC434570, TC378878, TC406264, TC390792, TC394020, TC388950, TC374669, TC419222, TC410194, TC369371, TC448840, TC392875, TC409005, TC408228, TC384735, TC418728, TC405540, TC390569, TC391948, BQ294582, TC401915, CA724903, TC421954, TC405652, CV779166, DR739994, TC459193, TC395506, TC395672, TC450972, TC392358, TC409599, TC419165, TC389092, TC376800, TC379853, TC420248, CK199846, TC370315, TC449504, TC403687, TC405440, TC403968, TC460795, CJ792862, TC422789, TC370885, TC407232, TC440819, TC416492, TC393015, TC426980, TC377225, TC434000, TC404620, TC382858, TC403977, CA646741, TC429865, TC434820, TC379711, TC379903, TC377308, TC406749, TC392272, TC401244, CA730421, TC400114, TC413339, TC408907, TC375539, TC385013, TC380083, TC386963, TC412732, TC381191, TC369010, TC402080, TC373518, TC370350, TC383909, TC376527, TC374940, TC419584, CN010697, TC374392, TC378568, TC369983, CV761628, TC404420, TC383235, TC390150, TC373489, TC369199, TC419747, TC407304, CA676115, TC402186, BE415178, TC429062, TC423354, TC409344, TC413043, BJ243383, TC400484, CK214224, TC368603, TC398040, TC374964, TC375234, CA709177, TC385815, TC409187, TC429374, TC425690, TC375253, TC423182, TC381218, TC401124, TC375873, TC444302, TC435224, CJ563078, TC394850, TC384344, TC439154, TC387511, TC455532, TC392297, TC427600, TC388520, TC443712, TC379422, TC378956, TC411191, TC426326, TC375431, TC424252, TC391995, TC415501, TC422841, TC387064, TC445649, CA498496, TC394796, TC382777, TC440499, TC435533, TC407349, TC397994, TC414243, TC403234, TC429437, TC430988, TC371738, TC413771, TC387621, TC398633, TC460689, TC380125, TC410126, TC369064, TC432154, TC420735, TC404371, TC402939, TC427405, TC449256, TC373615, TC383949, TC378409, TC416685, TC451342, TC372664, TC399986, TC389375, TC433812, TC370114, TC406038, TC390319, TC376874, TC424154, TC458807, TC459245, TC429871, TC369928, TC419438, TC376490, TC384454, TC411287, TC386237, TC388751, TC431722, AL820497, TC435909, TC399471, TC408126, TC430096, TC399972, TC434396, TC421880, TC386414, TC389590, CJ854725, TC440636, TC403986, TC406193, TC383763, CD876572, TC369736, TC386279, CK197833, TC391785, TC377427, TC382786, TC409650, TC388158, TC418340, TC405773, TC382830, TC377653, DR739471, TC402534, TC371600, TC382129, TC391840, TC390285, TC412989, TC387766, TC407340, TC398026, TC406584, CV065343, TC402440, TC396636, TC375146, TC454407, CK199175, TC394661, TC389044, TC383176, TC449463, TC371037, TC388688, TC403328, TC408095, TC439939, TC375268, TC452503, TC388547, TC420877, TC429048, TC398978, TC388976, TC396365, TC379069, TC385526, TC423576, TC375530, TC377635, TC423010, TC402668, TC435799, TC438243, TC392887, TC370210, TC375918, TC417363, TC400592, TC387710, TC413700, TC385515, TC423880, TC451285, BG263159, TC458503, TC387194, TC391865, TC434689, TC441995, CK211469, TC437163, CB307332, TC404842, TC381296, TC444402, TC411817, TC436290, TC390489, TC421341, TC432504, TC383143 |
| [Cell part](http://amigo.geneontology.org/cgi-bin/amigo/go.cgi?action=query&view=query&query=GO:0044464&search_constraint=terms) | TC461921, BJ284275, TC410352, TC390944, TC370791, TC389190, TC453487, TC375864, TC432212, TC415588, TC389842, BQ838511, TC398052, TC387861, TC391749, TC374098, TC400388, TC457112, TC431932, CK215979, TC376351, TC430471, TC439423, TC383028, TC379171, TC388410, BJ282766, CK203550, TC416493, TC388136, TC458983, TC458562, TC393436, TC417260, TC422842, CK214103, TC433844, CK209294, TC410063, TC421143, TC386396, TC378615, TC373702, TC448471, TC456784, TC395453, TC392329, TC410182, TC445939, TC425847, CJ727624, CA614761, CJ550278, BE591166, TC411128, GH732878, TC397444, TC412569, TC415483, CA632212, TC393523, TC435852, TC413027, TC372549, TC394820, TC398731, TC418073, TC411784, TC417308, TC387817, TC395069, TC413460, TC411008, BQ609416, CK214702, TC426907, TC395723, TC369415, TC387981, TC385233, TC391950, TC398730, TC386707, TC376248, BE217043, TC374409, TC402069, TC381943, TC432001, TC420358, CV770684, TC418928, TC398209, BQ607161, TC372845, TC382045, TC431175, TC402308, TC378790, TC417077, TC385009, CV759879, TC376625, TC390016, TC380474, CA606693, TC370912, TC432369, TC385714, TC412212, TC375124, TC392778, TC397312, TC405030, CA615187, TC405041, TC444549, TC397019, BJ317882, TC406469, TC399919, TC407572, TC413118, TC406708, TC373898, TC418691, TC409459, TC420337, TC391360, TC416471, TC419919, CV763657, TC372761, TC406809, TC410569, TC437137, CN010359, TC373001, TC402603, TC449724, TC395914, TC372867, TC397909, CA729339, TC423348, BQ239045, TC430163, TC389993, TC426864, TC408312, CV766349, TC391156, CA613620, TC400362, TC398154, TC401293, TC369325, TC429747, TC375612, TC430501, CJ867263, TC400108, TC406106, TC381462, CD882425, TC377749, TC396451, TC431198, TC424344, TC404158, CK201269, TC416381, TC429217, TC372677, CJ868963, TC374240, TC376306, TC425841, TC405982, TC387579, TC420460, TC428008, TC427006, TC380943, TC376202, TC403885, TC405946, TC460907, TC394451, TC395312, CV772029, TC387344, TC389090, TC389139, TC412483, TC382655, TC389144, TC456230, TC373994, TC395351, TC392709, TC372563, TC418032, CK162413, TC397291, TC461977, TC430555, TC411908, CA598430, TC459008, TC380329, TC376930, TC391516, TC409343, TC389696, TC403852, TC428066, TC382742, TC377190, TC443814, TC431879, TC423110, TC422348, TC391613, TC385365, TC430544, TC382339, TC379357, TC371003, TC387135, TC390566, TC378437, TC438587, TC375760, TC381923, TC426016, DR734732, TC387686, TC373251, TC413124, TC388566, TC378225, TC399245, TC373429, GH729256, TC418716, TC392447, TC421467, TC379023, TC386519, TC394219, TC383047, TC408381, TC417992, TC385090, TC407964, TC394307, TC388665, TC373583, TC406371, TC382139, TC392363, TC381988, TC370106, TC388819, TC405475, TC386401, TC422022, TC386344, TC452356, TC373637, TC370044, TC421872, TC386422, TC442623, CJ776780, TC422583, TC384071, TC400379, TC414606, TC393037, CJ730226, TC421733, TC383570, TC396228, TC379965, TC396650, TC384678, TC456370, TC404053, TC400562, TC371970, TC375726, TC391461, TC459656, DR734904, TC378783, TC379436, TC377765, TC440632, TC403929, TC457991, TC394118, TC378601, TC373848, TC426870, TC437918, TC387007, TC375935, TC418091, TC389816, TC371387, TC372530, TC392323, TC370907, TC384194, TC413066, TC371172, TC390211, TC446092, TC372167, TC391641, TC461622, TC393426, TC400056, TC405033, TC379470, TC419090, TC385659, TC376933, TC382910, TC432678, TC389451, TC458892, DR740372, TC394693, TC416495, TC401758, TC428944, TC417067, TC449093, TC379942, TC434821, TC374404, BQ295499, TC396230, TC377766, TC398383, TC394965, TC368599, TC391648, TC376937, TC433162, TC400317, TC404978, TC389363, TC371248, TC379338, TC433116, TC409167, TC416442, TC374679, TC387410, TC378072, TC397176, TC406516, TC406986, TC371145, TC435546, TC419057, TC451950, TC380695, TC398714, TC381619, TC424179, TC394028, TC398970, TC457126, TC456854, TC403679, TC418747, TC440803, TC403580, TC413427, TC386305, TC424044, TC372667, TC433957, TC386440, TC392432, TC423239, TC425821, TC370962, TC403055, TC394206, TC406236, TC382080, TC391962, TC391128, TC445767, TC387501, TC446038, TC374852, TC369830, TC423804, TC418685, CK213497, TC414899, TC436309, TC398514, TC391900, TC392033, TC423091, TC447694, TC380825, TC392269, TC412520, TC406807, TC395090, TC404606, TC441239, TC416906, TC416438, CA595837, TC423265, TC417388, TC440066, TC386313, CK208222, TC404179, TC435055, TC389108, TC369844, TC420579, CK211707, TC392126, TC402340, TC431201, CK217689, TC370392, CV761547, TC396751, CK211589, TC411116, TC388718, TC383701, TC452793, TC385780, TC405784, TC395566, TC372175, TC382734, TC405896, TC385927, TC369072, TC385513, TC439501, TC405356, TC444586, TC449448, TC377441, TC381596, TC403264, TC369348, TC461971, TC435825, TC422176, TC388232, TC445281, TC376263, TC415521, TC384010, TC416169, TC409043, TC408229, TC377290, TC409190, TC403537, TC381255, CV781430, TC379924, TC418399, TC431382, TC387683, TC409855, TC434831, TC406194, TC427161, TC373914, TC450285, TC392303, TC382024, TC404998, TC369899, TC378846, TC372330, TC448051, TC381068, CD930656, TC403936, CF554444, TC451276, TC444375, TC387050, TC415685, TC388914, TC415056, TC433488, TC377178, TC377438, TC394716, TC397027, TC456160, TC394670, TC378271, TC377292, TC380433, TC451621, TC435281, TC384219, TC397562, TC428853, TC404413, TC408192, CJ626653, TC372046, CK210754, TC407191, TC370727, TC417769, TC449235, TC410147, TC414564, TC405910, TC431603, TC393904, TC387191, TC407395, TC447801, TC380416, TC427981, CA500690, TC398536, TC400755, TC403717, TC372701, TC405615, TC376420, TC458987, TC404843, TC382737, TC370633, TC390135, TC411480, TC417755, TC420394, TC432320, TC387170, TC439225, TC380662, TC388649, TC419727, TC413462, TC394263, TC436772, TC395298, TC394253, TC412094, CV782550, TC389162, TC376630, CK212850, TC389678, TC399432, TC434006, TC434949, TC369726, CA727263, TC379241, TC405295, TC417480, TC392465, TC455676, TC373962, TC391411, TC409208, TC375813, TC379343, TC374741, TC429809, TC386591, TC421704, TC420201, TC381963, TC456247, TC371455, TC374879, GH728664, TC385701, TC400181, TC418759, TC431825, TC377496, TC368549, TC401158, TC387133, TC410078, TC415942, TC393561, TC393692, TC386322, TC391671, TC373787, BQ160837, TC382928, TC397415, TC370507, TC369637, TC396487, TC436832, TC458699, TC380882, TC440321, TC372580, TC444588, TC377410, TC400721, TC451657, TC445214, TC451519, TC393820, TC435808, TC449739, TC416981, BE586004, TC397729, TC399408, TC417970, TC426743, TC396895, TC382342, TC393106, TC428932, TC431306, TC396460, TC397676, TC379968, TC399730, TC368580, TC407456, TC441536, TC373613, TC386040, TC407978, TC390393, TC373958, TC444546, TC411284, TC392283, TC390516, TC416529, TC391946, TC460760, TC420420, TC383139, TC408524, TC417106, TC419614, TC432124, CA710880, TC452945, TC375593, TC421871, TC371242, TC394459, TC423252, TC384738, TC416154, TC397660, CV766916, TC407404, TC390379, TC374996, TC393781, TC421914, TC435210, TC380063, TC398538, TC418414, CJ944525, TC396772, TC378054, TC453207, TC419273, TC461607, TC409843, TC406594, TC437445, TC403872, TC407076, TC400595, TC371357, TC406207, TC389586, TC395746, TC439210, TC398304, TC393100, TC425878, CV771134, BE431108, TC417012, TC391447, TC455515, TC394916, TC397793, TC404914, TC391130, TC378274, CK217367, TC378153, TC379990, TC410144, TC378333, TC444410, TC435863, BQ788843, TC452050, CJ845228, TC379536, TC391621, TC404052, TC389718, TC429713, CK201148, TC386088, TC389534, TC413392, TC422142, TC398379, TC385783, TC376758, CJ815867, TC369628, TC441343, TC400260, TC400330, TC372796, TC399057, TC408191, TC378432, TC377061, TC389843, TC388317, CD937281, TC446465, TC416801, AL827131, TC379312, TC391438, TC381268, TC412317, TC371325, TC439708, TC428113, TC373563, TC370619, BJ258087, TC421345, TC422007, TC370178, TC405695, TC388403, TC373678, TC416695, TC410690, TC374461, TC410074, TC443289, TC386646, TC399841, TC369687, TC458205, TC441241, TC433557, TC413571, TC415803, TC408299, TC410108, TC454526, TC398830, TC399352, TC422425, TC371129, TC398805, TC372654, TC428992, TC369092, TC420816, TC384162, TC395872, TC383739, TC399342, TC432185, TC381583, TC455736, TC392263, TC432130, TC375665, TC385205, TC384625, TC427986, TC422645, TC425957, TC445166, BG904091, TC421162, TC429341, TC398121, TC389168, TC424204, TC439324, TC422288, TC406870, TC424317, TC393814, TC376220, TC373402, TC408309, TC404926, TC446235, TC402545, TC393830, TC415808, TC444899, TC386961, TC398606, TC387319, TC396134, TC369182, TC418365, TC373204, TC370347, TC373002, TC375554, CK200433, TC393970, TC374731, TC452494, TC456619, TC388228, TC444138, TC377373, TC415365, EB512907, TC390994, TC412205, TC393437, TC447260, TC426916, TC391143, TC381463, TC403157, TC410066, TC414090, TC395303, TC442140, TC405511, TC386410, TC392203, TC393890, TC442727, TC390762, TC401210, DR737360, TC381007, TC397885, TC411684, TC382728, TC388049, TC404850, TC370033, TC433940, TC374485, TC390436, TC393960, TC426358, TC385326, TC449064, TC387707, TC409077, TC443387, TC415083, TC370470, TC386535, TC390630, TC390402, TC374726, TC417341, TC415954, TC376790, TC398592, TC389589, TC378911, TC452762, TC407490, CD878039, TC374164, TC418845, TC377993, TC438657, DR735108, TC412150, TC426078, TC388822, TC429771, TC386639, TC375069, TC416658, TC424962, TC379944, TC374230, TC391913, TC375834, TC393948, TC403624, TC400638, TC393554, TC407614, TC400477, TC384049, TC384357, TC391007, TC460615, TC403573, TC411941, TC385445, TC395841, TC449043, TC379635, TC392247, TC373259, TC433589, TC382338, TC375914, TC398343, TC377253, TC404426, TC439472, TC377944, TC381817, TC403588, TC394728, TC403803, TC418850, TC440526, TC436944, TC371341, TC389661, TC381279, CA726837, TC387386, TC383820, TC396657, TC380333, TC377064, TC397258, TC426838, CV775873, TC451511, TC431330, TC384373, TC416069, TC427210, TC422606, TC399271, TC375313, TC398862, DR739303, TC381613, TC434442, TC413263, TC411471, TC431660, TC434570, TC378878, TC406264, TC390792, TC394020, TC388950, TC374669, TC419222, TC410194, TC369371, TC448840, TC392875, TC409005, TC408228, TC384735, TC418728, TC405540, TC390569, TC391948, BQ294582, TC401915, CA724903, TC421954, TC405652, CV779166, DR739994, TC459193, TC395506, TC395672, TC450972, TC392358, TC409599, TC419165, TC389092, TC376800, TC379853, TC420248, CK199846, TC370315, TC449504, TC403687, TC405440, TC403968, TC460795, CJ792862, TC422789, TC370885, TC407232, TC440819, TC416492, TC393015, TC426980, TC377225, TC434000, TC404620, TC382858, TC403977, CA646741, TC429865, TC434820, TC379711, TC379903, TC377308, TC406749, TC392272, TC401244, CA730421, TC400114, TC413339, TC408907, TC375539, TC385013, TC380083, TC386963, TC412732, TC381191, TC369010, TC402080, TC373518, TC370350, TC383909, TC376527, TC374940, TC419584, CN010697, TC374392, TC378568, TC369983, CV761628, TC404420, TC383235, TC390150, TC373489, TC369199, TC419747, TC407304, CA676115, TC402186, BE415178, TC429062, TC423354, TC409344, TC413043, BJ243383, TC400484, CK214224, TC368603, TC398040, TC374964, TC375234, CA709177, TC385815, TC409187, TC429374, TC425690, TC375253, TC423182, TC381218, TC401124, TC375873, TC444302, TC435224, CJ563078, TC394850, TC384344, TC439154, TC387511, TC455532, TC392297, TC427600, TC388520, TC443712, TC379422, TC378956, TC411191, TC426326, TC375431, TC424252, TC391995, TC415501, TC422841, TC387064, TC445649, CA498496, TC394796, TC382777, TC440499, TC435533, TC407349, TC397994, TC414243, TC403234, TC429437, TC430988, TC371738, TC413771, TC387621, TC398633, TC460689, TC380125, TC410126, TC369064, TC432154, TC420735, TC404371, TC402939, TC427405, TC449256, TC373615, TC383949, TC378409, TC416685, TC451342, TC372664, TC399986, TC389375, TC433812, TC370114, TC406038, TC390319, TC376874, TC424154, TC458807, TC459245, TC429871, TC369928, TC419438, TC376490, TC384454, TC411287, TC386237, TC388751, TC431722, AL820497, TC435909, TC399471, TC408126, TC430096, TC399972, TC434396, TC421880, TC386414, TC389590, CJ854725, TC440636, TC403986, TC406193, TC383763, CD876572, TC369736, TC386279, CK197833, TC391785, TC377427, TC382786, TC409650, TC388158, TC418340, TC405773, TC382830, TC377653, DR739471, TC402534, TC371600, TC382129, TC391840, TC390285, TC412989, TC387766, TC407340, TC398026, TC406584, CV065343, TC402440, TC396636, TC375146, TC454407, CK199175, TC394661, TC389044, TC383176, TC449463, TC371037, TC388688, TC403328, TC408095, TC439939, TC375268, TC452503, TC388547, TC420877, TC429048, TC398978, TC388976, TC396365, TC379069, TC385526, TC423576, TC375530, TC377635, TC423010, TC402668, TC435799, TC438243, TC392887, TC370210, TC375918, TC417363, TC400592, TC387710, TC413700, TC385515, TC423880, TC451285, BG263159, TC458503, TC387194, TC391865, TC434689, TC441995, CK211469, TC437163, CB307332, TC404842, TC381296, TC444402, TC411817, TC436290, TC390489, TC421341, TC432504, TC383143 |
| [Intracellular part](http://amigo.geneontology.org/cgi-bin/amigo/go.cgi?action=query&view=query&query=GO:0044424&search_constraint=terms) | TC461921, BJ284275, TC410352, TC390944, TC370791, TC389190, TC453487, TC375864, TC432212, TC415588, TC389842, BQ838511, TC398052, TC387861, TC391749, TC374098, TC400388, TC457112, TC431932, CK215979, TC376351, TC430471, TC439423, TC383028, TC379171, TC388410, BJ282766, CK203550, TC416493, TC388136, TC458562, TC393436, TC417260, CK214103, TC433844, CK209294, TC410063, TC421143, TC386396, TC378615, TC373702, TC448471, TC456784, TC395453, TC392329, TC410182, TC445939, TC425847, CJ727624, CA614761, CJ550278, BE591166, TC411128, GH732878, TC397444, TC412569, TC415483, CA632212, TC393523, TC435852, TC413027, TC372549, TC394820, TC398731, TC418073, TC411784, TC417308, TC387817, TC395069, TC413460, TC411008, BQ609416, CK214702, TC426907, TC395723, TC369415, TC387981, TC385233, TC391950, TC398730, TC386707, TC376248, BE217043, TC374409, TC402069, TC381943, TC432001, TC420358, CV770684, TC418928, TC398209, BQ607161, TC372845, TC382045, TC431175, TC402308, TC378790, TC417077, TC385009, CV759879, TC376625, TC390016, TC380474, CA606693, TC370912, TC432369, TC385714, TC412212, TC375124, TC392778, TC397312, TC405030, CA615187, TC405041, TC444549, TC397019, BJ317882, TC406469, TC399919, TC407572, TC413118, TC406708, TC373898, TC418691, TC409459, TC420337, TC391360, TC416471, TC419919, CV763657, TC372761, TC406809, TC410569, TC437137, CN010359, TC373001, TC402603, TC449724, TC395914, TC372867, TC397909, CA729339, BQ239045, TC430163, TC389993, TC426864, TC408312, TC391156, CA613620, TC400362, TC398154, TC401293, TC369325, TC429747, TC375612, TC430501, CJ867263, TC400108, TC406106, TC381462, CD882425, TC377749, TC396451, TC431198, TC424344, TC429217, TC372677, CJ868963, TC374240, TC376306, TC425841, TC405982, TC387579, TC420460, TC428008, TC427006, TC380943, TC376202, TC403885, TC405946, TC460907, TC394451, TC395312, CV772029, TC387344, TC389090, TC389139, TC412483, TC382655, TC389144, TC456230, TC373994, TC395351, TC392709, TC372563, TC418032, CK162413, TC397291, TC461977, TC411908, CA598430, TC459008, TC380329, TC376930, TC391516, TC409343, TC389696, TC403852, TC382742, TC377190, TC443814, TC431879, TC423110, TC422348, TC391613, TC385365, TC430544, TC382339, TC379357, TC371003, TC387135, TC378437, TC438587, TC375760, TC381923, TC426016, DR734732, TC387686, TC373251, TC413124, TC388566, TC399245, TC373429, GH729256, TC418716, TC392447, TC421467, TC379023, TC386519, TC394219, TC383047, TC417992, TC385090, TC407964, TC394307, TC388665, TC373583, TC406371, TC382139, TC392363, TC381988, TC370106, TC388819, TC405475, TC386401, TC422022, TC386344, TC452356, TC373637, TC370044, TC421872, TC386422, TC442623, CJ776780, TC422583, TC400379, TC414606, TC393037, CJ730226, TC421733, TC383570, TC396228, TC379965, TC396650, TC384678, TC456370, TC404053, TC400562, TC371970, TC375726, TC459656, DR734904, TC378783, TC379436, TC377765, TC403929, TC457991, TC394118, TC378601, TC373848, TC426870, TC437918, TC387007, TC375935, TC418091, TC389816, TC372530, TC392323, TC370907, TC384194, TC413066, TC371172, TC390211, TC372167, TC391641, TC461622, TC393426, TC400056, TC405033, TC379470, TC419090, TC385659, TC382910, TC432678, TC389451, TC458892, DR740372, TC394693, TC416495, TC401758, TC428944, TC417067, TC449093, TC379942, TC434821, TC374404, BQ295499, TC396230, TC377766, TC394965, TC368599, TC391648, TC376937, TC433162, TC400317, TC404978, TC389363, TC371248, TC379338, TC433116, TC409167, TC416442, TC374679, TC387410, TC378072, TC397176, TC406516, TC406986, TC371145, TC435546, TC419057, TC451950, TC380695, TC398714, TC381619, TC424179, TC394028, TC398970, TC457126, TC456854, TC403679, TC418747, TC440803, TC403580, TC413427, TC386305, TC424044, TC372667, TC433957, TC386440, TC392432, TC423239, TC425821, TC370962, TC403055, TC394206, TC406236, TC382080, TC391962, TC391128, TC445767, TC387501, TC446038, TC374852, TC369830, TC423804, TC418685, CK213497, TC414899, TC436309, TC398514, TC391900, TC392033, TC423091, TC447694, TC380825, TC412520, TC406807, TC395090, TC404606, TC441239, TC416906, TC416438, CA595837, TC423265, TC417388, TC440066, TC386313, CK208222, TC404179, TC389108, TC369844, TC420579, CK211707, TC392126, TC402340, TC431201, CK217689, TC370392, CV761547, TC396751, CK211589, TC411116, TC388718, TC383701, TC452793, TC385780, TC405784, TC395566, TC372175, TC382734, TC405896, TC385927, TC369072, TC385513, TC439501, TC405356, TC444586, TC449448, TC377441, TC381596, TC403264, TC369348, TC461971, TC435825, TC422176, TC388232, TC445281, TC376263, TC415521, TC384010, TC416169, TC409043, TC408229, TC377290, TC409190, TC403537, TC381255, CV781430, TC379924, TC418399, TC431382, TC387683, TC409855, TC434831, TC406194, TC427161, TC373914, TC450285, TC392303, TC382024, TC404998, TC369899, TC378846, TC372330, TC448051, TC381068, CD930656, TC403936, CF554444, TC451276, TC444375, TC387050, TC415685, TC388914, TC415056, TC433488, TC377178, TC377438, TC394716, TC397027, TC456160, TC394670, TC378271, TC377292, TC380433, TC451621, TC435281, TC384219, TC397562, TC428853, TC404413, TC408192, CJ626653, TC372046, CK210754, TC407191, TC370727, TC417769, TC449235, TC410147, TC414564, TC405910, TC431603, TC393904, TC387191, TC407395, TC447801, TC380416, TC427981, CA500690, TC398536, TC400755, TC403717, TC372701, TC405615, TC376420, TC458987, TC404843, TC382737, TC370633, TC390135, TC411480, TC417755, TC420394, TC432320, TC387170, TC439225, TC380662, TC388649, TC419727, TC394263, TC436772, TC395298, TC394253, TC412094, CV782550, TC389162, TC376630, CK212850, TC389678, TC399432, TC434006, TC434949, TC369726, CA727263, TC379241, TC405295, TC417480, TC392465, TC455676, TC373962, TC391411, TC409208, TC375813, TC379343, TC374741, TC429809, TC386591, TC421704, TC420201, TC381963, TC456247, TC371455, TC374879, GH728664, TC385701, TC400181, TC418759, TC431825, TC377496, TC368549, TC401158, TC387133, TC410078, TC415942, TC393561, TC393692, TC391671, TC373787, BQ160837, TC382928, TC397415, TC370507, TC369637, TC396487, TC436832, TC458699, TC380882, TC440321, TC372580, TC444588, TC377410, TC400721, TC451657, TC445214, TC451519, TC393820, TC435808, TC449739, TC416981, BE586004, TC397729, TC399408, TC417970, TC426743, TC396895, TC382342, TC393106, TC428932, TC431306, TC396460, TC397676, TC379968, TC399730, TC368580, TC441536, TC373613, TC386040, TC407978, TC390393, TC444546, TC392283, TC390516, TC416529, TC391946, TC460760, TC420420, TC408524, TC417106, TC432124, CA710880, TC452945, TC375593, TC421871, TC371242, TC394459, TC423252, TC384738, TC416154, TC397660, CV766916, TC390379, TC407404, TC374996, TC393781, TC421914, TC435210, TC398538, TC380063, TC418414, CJ944525, TC396772, TC378054, TC453207, TC419273, TC461607, TC409843, TC437445, TC403872, TC407076, TC371357, TC406207, TC389586, TC395746, TC439210, TC398304, TC393100, TC425878, CV771134, BE431108, TC417012, TC391447, TC455515, TC394916, TC397793, TC404914, TC391130, TC378274, CK217367, TC378153, TC379990, TC378333, TC444410, TC435863, BQ788843, TC452050, CJ845228, TC379536, TC391621, TC404052, TC389718, TC429713, CK201148, TC386088, TC389534, TC413392, TC422142, TC398379, TC376758, CJ815867, TC369628, TC441343, TC400260, TC400330, TC399057, TC408191, TC378432, TC377061, TC389843, TC388317, CD937281, TC416801, AL827131, TC446465, TC379312, TC391438, TC381268, TC412317, TC371325, TC439708, TC428113, TC373563, TC370619, BJ258087, TC421345, TC422007, TC370178, TC405695, TC388403, TC373678, TC416695, TC410690, TC374461, TC410074, TC443289, TC386646, TC399841, TC369687, TC458205, TC441241, TC433557, TC413571, TC415803, TC408299, TC410108, TC454526, TC398830, TC399352, TC422425, TC371129, TC398805, TC372654, TC428992, TC369092, TC420816, TC384162, TC395872, TC383739, TC399342, TC432185, TC381583, TC455736, TC392263, TC432130, TC375665, TC385205, TC427986, TC422645, TC425957, TC445166, BG904091, TC421162, TC398121, TC389168, TC424204, TC439324, TC422288, TC406870, TC424317, TC393814, TC376220, TC373402, TC404926, TC446235, TC402545, TC393830, TC444899, TC386961, TC398606, TC387319, TC396134, TC369182, TC418365, TC373204, TC370347, TC373002, TC375554, CK200433, TC393970, TC374731, TC452494, TC456619, TC388228, TC444138, TC377373, EB512907, TC390994, TC412205, TC393437, TC426916, TC391143, TC381463, TC403157, TC410066, TC414090, TC395303, TC442140, TC405511, TC386410, TC392203, TC393890, TC442727, TC390762, TC401210, DR737360, TC381007, TC397885, TC411684, TC382728, TC388049, TC404850, TC370033, TC433940, TC374485, TC390436, TC393960, TC426358, TC385326, TC387707, TC409077, TC443387, TC415083, TC370470, TC386535, TC390630, TC390402, TC374726, TC417341, TC415954, TC376790, TC398592, TC389589, TC378911, TC452762, TC407490, CD878039, TC374164, TC418845, TC377993, TC438657, DR735108, TC412150, TC426078, TC388822, TC429771, TC386639, TC375069, TC416658, TC424962, TC379944, TC374230, TC391913, TC375834, TC393948, TC403624, TC400638, TC393554, TC407614, TC400477, TC384049, TC384357, TC391007, TC460615, TC403573, TC411941, TC385445, TC395841, TC449043, TC379635, TC392247, TC373259, TC433589, TC382338, TC375914, TC398343, TC377253, TC404426, TC439472, TC377944, TC381817, TC403588, TC394728, TC403803, TC418850, TC440526, TC436944, TC371341, TC381279, TC389661, CA726837, TC387386, TC383820, TC396657, TC380333, TC377064, TC397258, TC426838, CV775873, TC451511, TC431330, TC384373, TC416069, TC427210, TC422606, TC399271, TC375313, TC398862, DR739303, TC434442, TC413263, TC411471, TC431660, TC434570, TC378878, TC406264, TC390792, TC394020, TC388950, TC374669, TC419222, TC410194, TC369371, TC448840, TC392875, TC409005, TC408228, TC384735, TC418728, TC405540, TC390569, TC391948, BQ294582, TC401915, CA724903, TC405652, CV779166, DR739994, TC459193, TC395506, TC395672, TC450972, TC392358, TC409599, TC419165, TC389092, TC376800, TC379853, TC420248, CK199846, TC370315, TC449504, TC403687, TC405440, TC403968, TC460795, CJ792862, TC422789, TC370885, TC407232, TC440819, TC416492, TC393015, TC426980, TC377225, TC404620, TC382858, TC403977, CA646741, TC429865, TC434820, TC379711, TC379903, TC377308, TC406749, TC392272, TC401244, CA730421, TC400114, TC413339, TC408907, TC375539, TC385013, TC380083, TC386963, TC412732, TC381191, TC369010, TC402080, TC373518, TC370350, TC383909, TC376527, TC374940, TC419584, CN010697, TC374392, TC378568, TC369983, CV761628, TC404420, TC383235, TC373489, TC369199, TC419747, TC407304, CA676115, TC402186, BE415178, TC429062, TC423354, TC409344, TC413043, BJ243383, TC400484, CK214224, TC368603, TC398040, TC374964, TC375234, CA709177, TC385815, TC409187, TC429374, TC425690, TC375253, TC423182, TC381218, TC401124, TC375873, TC444302, TC435224, CJ563078, TC394850, TC384344, TC392297, TC388520, TC443712, TC379422, TC378956, TC411191, TC426326, TC375431, TC424252, TC391995, TC415501, TC422841, TC387064, TC445649, CA498496, TC394796, TC382777, TC440499, TC435533, TC407349, TC397994, TC414243, TC403234, TC429437, TC430988, TC371738, TC413771, TC387621, TC398633, TC460689, TC380125, TC410126, TC369064, TC432154, TC420735, TC404371, TC402939, TC427405, TC449256, TC373615, TC383949, TC378409, TC416685, TC451342, TC372664, TC399986, TC389375, TC433812, TC370114, TC406038, TC390319, TC376874, TC424154, TC458807, TC459245, TC429871, TC369928, TC419438, TC376490, TC384454, TC411287, TC386237, TC388751, TC431722, AL820497, TC435909, TC399471, TC408126, TC430096, TC399972, TC434396, TC421880, TC386414, TC389590, CJ854725, TC440636, TC403986, TC406193, TC383763, CD876572, TC369736, TC386279, CK197833, TC391785, TC377427, TC382786, TC409650, TC388158, TC418340, TC405773, TC382830, TC377653, DR739471, TC402534, TC371600, TC382129, TC391840, TC390285, TC412989, TC387766, TC407340, TC398026, TC406584, CV065343, TC402440, TC396636, TC375146, TC454407, CK199175, TC394661, TC389044, TC383176, TC449463, TC371037, TC388688, TC403328, TC408095, TC439939, TC375268, TC452503, TC388547, TC420877, TC429048, TC398978, TC388976, TC396365, TC379069, TC385526, TC423576, TC375530, TC377635, TC402668, TC435799, TC438243, TC392887, TC370210, TC375918, TC417363, TC400592, TC387710, TC413700, TC385515, TC423880, TC451285, BG263159, TC458503, TC387194, TC391865, TC434689, TC441995, CK211469, CB307332, TC404842, TC381296, TC411817, TC436290, TC390489, TC421341, TC432504, TC383143 |
| [Soluble fraction](http://amigo.geneontology.org/cgi-bin/amigo/go.cgi?action=query&view=query&query=GO:0005625&search_constraint=terms) | TC381988, TC405511, TC407076, TC435799, TC370885, TC461622, TC404843, TC400056, TC380882, TC423880, TC410078, TC425957, TC423354, TC403588, TC445166, TC398514 |
| [Small nucleolar ribonucleoprotein complex](http://amigo.geneontology.org/cgi-bin/amigo/go.cgi?action=query&view=query&query=GO:0005732&search_constraint=terms) | TC387135, TC387410, TC405540, TC387710, TC388566, TC417341, TC385233, TC398730, TC404606 |
| [Condensed chromosome, centromeric region](http://amigo.geneontology.org/cgi-bin/amigo/go.cgi?action=query&view=query&query=GO:0000779&search_constraint=terms) | TC394796, TC370315 |
| [Sec61 translocon complex](http://amigo.geneontology.org/cgi-bin/amigo/go.cgi?action=query&view=query&query=GO:0005784&search_constraint=terms) | TC409343, TC412569 |
| [Translocon complex](http://amigo.geneontology.org/cgi-bin/amigo/go.cgi?action=query&view=query&query=GO:0071256&search_constraint=terms) | TC409343, TC412569 |
| [Postsynaptic density](http://amigo.geneontology.org/cgi-bin/amigo/go.cgi?action=query&view=query&query=GO:0014069&search_constraint=terms) | CK211589, TC413571 |
| [Intracellular](http://amigo.geneontology.org/cgi-bin/amigo/go.cgi?action=query&view=query&query=GO:0005622&search_constraint=terms) | TC461921, BJ284275, TC410352, TC390944, TC370791, TC389190, TC453487, TC375864, TC432212, TC415588, TC389842, BQ838511, TC398052, TC387861, TC391749, TC374098, TC400388, TC457112, TC431932, CK215979, TC376351, TC430471, TC439423, TC383028, TC379171, TC388410, BJ282766, CK203550, TC416493, TC388136, TC458562, TC393436, TC417260, CK214103, TC433844, CK209294, TC410063, TC421143, TC386396, TC378615, TC373702, TC448471, TC456784, TC395453, TC392329, TC410182, TC445939, TC425847, CJ727624, CA614761, CJ550278, BE591166, TC411128, GH732878, TC397444, TC412569, TC415483, CA632212, TC393523, TC435852, TC413027, TC372549, TC394820, TC398731, TC418073, TC411784, TC417308, TC387817, TC395069, TC413460, TC411008, BQ609416, CK214702, TC426907, TC395723, TC369415, TC387981, TC385233, TC391950, TC398730, TC386707, TC376248, BE217043, TC374409, TC402069, TC381943, TC432001, TC420358, CV770684, TC418928, TC398209, BQ607161, TC372845, TC382045, TC431175, TC402308, TC378790, TC417077, TC385009, CV759879, TC376625, TC390016, TC380474, CA606693, TC370912, TC432369, TC385714, TC412212, TC375124, TC392778, TC397312, TC405030, CA615187, TC405041, TC444549, TC397019, BJ317882, TC406469, TC399919, TC407572, TC413118, TC406708, TC373898, TC418691, TC409459, TC420337, TC391360, TC416471, TC419919, CV763657, TC372761, TC406809, TC410569, TC437137, CN010359, TC373001, TC402603, TC449724, TC395914, TC372867, TC397909, CA729339, BQ239045, TC430163, TC389993, TC426864, TC408312, TC391156, CA613620, TC400362, TC398154, TC401293, TC369325, TC429747, TC375612, TC430501, CJ867263, TC400108, TC406106, TC381462, CD882425, TC377749, TC396451, TC431198, TC424344, TC429217, TC372677, CJ868963, TC374240, TC376306, TC425841, TC405982, TC387579, TC420460, TC428008, TC427006, TC380943, TC376202, TC403885, TC405946, TC460907, TC394451, TC395312, CV772029, TC387344, TC389090, TC389139, TC412483, TC382655, TC389144, TC456230, TC373994, TC395351, TC392709, TC372563, TC418032, CK162413, TC397291, TC461977, TC411908, CA598430, TC459008, TC380329, TC376930, TC391516, TC409343, TC389696, TC403852, TC382742, TC377190, TC443814, TC431879, TC423110, TC422348, TC391613, TC385365, TC430544, TC382339, TC379357, TC371003, TC387135, TC378437, TC438587, TC375760, TC381923, TC426016, DR734732, TC387686, TC373251, TC413124, TC388566, TC399245, TC373429, GH729256, TC418716, TC392447, TC421467, TC379023, TC386519, TC394219, TC383047, TC417992, TC385090, TC407964, TC394307, TC388665, TC373583, TC406371, TC382139, TC392363, TC381988, TC370106, TC388819, TC405475, TC386401, TC422022, TC386344, TC452356, TC373637, TC370044, TC421872, TC386422, TC442623, CJ776780, TC422583, TC400379, TC414606, TC393037, CJ730226, TC421733, TC383570, TC396228, TC379965, TC396650, TC384678, TC456370, TC404053, TC400562, TC371970, TC375726, TC459656, DR734904, TC378783, TC379436, TC377765, TC403929, TC457991, TC394118, TC378601, TC373848, TC426870, TC437918, TC387007, TC375935, TC418091, TC389816, TC372530, TC392323, TC370907, TC384194, TC413066, TC371172, TC390211, TC372167, TC391641, TC461622, TC393426, TC400056, TC405033, TC379470, TC419090, TC385659, TC382910, TC432678, TC389451, TC458892, DR740372, TC394693, TC416495, TC401758, TC428944, TC417067, TC449093, TC379942, TC434821, TC374404, BQ295499, TC396230, TC377766, TC394965, TC368599, TC391648, TC376937, TC433162, TC400317, TC404978, TC389363, TC371248, TC379338, TC433116, TC409167, TC416442, TC374679, TC387410, TC378072, TC397176, TC406516, TC406986, TC371145, TC435546, TC419057, TC451950, TC380695, TC398714, TC381619, TC424179, TC394028, TC398970, TC457126, TC456854, TC403679, TC418747, TC440803, TC403580, TC413427, TC386305, TC424044, TC372667, TC433957, TC386440, TC392432, TC423239, TC425821, TC370962, TC403055, TC394206, TC406236, TC382080, TC391962, TC391128, TC445767, TC387501, TC446038, TC374852, TC369830, TC423804, TC418685, CK213497, TC414899, TC436309, TC398514, TC391900, TC392033, TC423091, TC447694, TC380825, TC412520, TC406807, TC395090, TC404606, TC441239, TC416906, TC416438, CA595837, TC423265, TC417388, TC440066, TC386313, CK208222, TC404179, TC389108, TC369844, TC420579, CK211707, TC392126, TC402340, TC431201, CK217689, TC370392, CV761547, TC396751, CK211589, TC411116, TC388718, TC383701, TC452793, TC385780, TC405784, TC395566, TC372175, TC382734, TC405896, TC385927, TC369072, TC385513, TC439501, TC405356, TC444586, TC449448, TC377441, TC381596, TC403264, TC369348, TC461971, TC435825, TC422176, TC388232, TC445281, TC376263, TC415521, TC384010, TC416169, TC409043, TC408229, TC377290, TC409190, TC403537, TC381255, CV781430, TC379924, TC418399, TC431382, TC387683, TC409855, TC434831, TC406194, TC427161, TC373914, TC450285, TC392303, TC382024, TC404998, TC369899, TC378846, TC372330, TC448051, TC381068, CD930656, TC403936, CF554444, TC451276, TC444375, TC387050, TC415685, TC388914, TC415056, TC433488, TC377178, TC377438, TC394716, TC397027, TC456160, TC394670, TC378271, TC377292, TC380433, TC451621, TC435281, TC384219, TC397562, TC428853, TC404413, TC408192, CJ626653, TC372046, CK210754, TC407191, TC370727, TC417769, TC449235, TC410147, TC414564, TC405910, TC431603, TC393904, TC387191, TC407395, TC447801, TC380416, TC427981, CA500690, TC398536, TC400755, TC403717, TC372701, TC405615, TC376420, TC458987, TC404843, TC382737, TC370633, TC390135, TC411480, TC417755, TC420394, TC432320, TC387170, TC439225, TC380662, TC388649, TC419727, TC394263, TC436772, TC395298, TC394253, TC412094, CV782550, TC389162, TC376630, CK212850, TC389678, TC399432, TC434006, TC434949, TC369726, CA727263, TC379241, TC405295, TC417480, TC392465, TC455676, TC373962, TC391411, TC409208, TC375813, TC379343, TC374741, TC429809, TC386591, TC421704, TC420201, TC381963, TC456247, TC371455, TC374879, GH728664, TC385701, TC400181, TC418759, TC431825, TC377496, TC368549, TC401158, TC387133, TC410078, TC415942, TC393561, TC393692, TC391671, TC373787, BQ160837, TC382928, TC397415, TC370507, TC369637, TC396487, TC436832, TC458699, TC380882, TC440321, TC372580, TC444588, TC377410, TC400721, TC451657, TC445214, TC451519, TC393820, TC435808, TC449739, TC416981, BE586004, TC397729, TC399408, TC417970, TC426743, TC396895, TC382342, TC393106, TC428932, TC431306, TC396460, TC397676, TC379968, TC399730, TC368580, TC441536, TC373613, TC386040, TC407978, TC390393, TC444546, TC392283, TC390516, TC416529, TC391946, TC460760, TC420420, TC408524, TC417106, TC432124, CA710880, TC452945, TC375593, TC421871, TC371242, TC394459, TC423252, TC384738, TC416154, TC397660, CV766916, TC390379, TC407404, TC374996, TC393781, TC421914, TC435210, TC398538, TC380063, TC418414, CJ944525, TC396772, TC378054, TC453207, TC419273, TC461607, TC409843, TC437445, TC403872, TC407076, TC371357, TC406207, TC389586, TC395746, TC439210, TC398304, TC393100, TC425878, CV771134, BE431108, TC417012, TC391447, TC455515, TC394916, TC397793, TC404914, TC391130, TC378274, CK217367, TC378153, TC379990, TC378333, TC444410, TC435863, BQ788843, TC452050, CJ845228, TC379536, TC391621, TC404052, TC389718, TC429713, CK201148, TC386088, TC389534, TC413392, TC422142, TC398379, TC376758, CJ815867, TC369628, TC441343, TC400260, TC400330, TC399057, TC408191, TC378432, TC377061, TC389843, TC388317, CD937281, TC416801, AL827131, TC446465, TC379312, TC391438, TC381268, TC412317, TC371325, TC439708, TC428113, TC373563, TC370619, BJ258087, TC421345, TC422007, TC370178, TC405695, TC388403, TC373678, TC416695, TC410690, TC374461, TC410074, TC443289, TC386646, TC399841, TC369687, TC458205, TC441241, TC433557, TC413571, TC415803, TC408299, TC410108, TC454526, TC398830, TC399352, TC422425, TC371129, TC398805, TC372654, TC428992, TC369092, TC420816, TC384162, TC395872, TC383739, TC399342, TC432185, TC381583, TC455736, TC392263, TC432130, TC375665, TC385205, TC427986, TC422645, TC425957, TC445166, BG904091, TC421162, TC398121, TC389168, TC424204, TC439324, TC422288, TC406870, TC424317, TC393814, TC376220, TC373402, TC404926, TC446235, TC402545, TC393830, TC444899, TC386961, TC398606, TC387319, TC396134, TC369182, TC418365, TC373204, TC370347, TC373002, TC375554, CK200433, TC393970, TC374731, TC452494, TC456619, TC388228, TC444138, TC377373, EB512907, TC390994, TC412205, TC393437, TC426916, TC391143, TC381463, TC403157, TC410066, TC414090, TC395303, TC442140, TC405511, TC386410, TC392203, TC393890, TC442727, TC390762, TC401210, DR737360, TC381007, TC397885, TC411684, TC382728, TC388049, TC404850, TC370033, TC433940, TC374485, TC390436, TC393960, TC426358, TC385326, TC387707, TC409077, TC443387, TC415083, TC370470, TC386535, TC390630, TC390402, TC374726, TC417341, TC415954, TC376790, TC398592, TC389589, TC378911, TC452762, TC407490, CD878039, TC374164, TC418845, TC377993, TC438657, DR735108, TC412150, TC426078, TC388822, TC429771, TC386639, TC375069, TC416658, TC424962, TC379944, TC374230, TC391913, TC375834, TC393948, TC403624, TC400638, TC393554, TC407614, TC400477, TC384049, TC384357, TC391007, TC460615, TC403573, TC411941, TC385445, TC395841, TC449043, TC379635, TC392247, TC373259, TC433589, TC382338, TC375914, TC398343, TC377253, TC404426, TC439472, TC377944, TC381817, TC403588, TC394728, TC403803, TC418850, TC440526, TC436944, TC371341, TC381279, TC389661, CA726837, TC387386, TC383820, TC396657, TC380333, TC377064, TC397258, TC426838, CV775873, TC451511, TC431330, TC384373, TC416069, TC427210, TC422606, TC399271, TC375313, TC398862, DR739303, TC434442, TC413263, TC411471, TC431660, TC434570, TC378878, TC406264, TC390792, TC394020, TC388950, TC374669, TC419222, TC410194, TC369371, TC448840, TC392875, TC409005, TC408228, TC384735, TC418728, TC405540, TC390569, TC391948, BQ294582, TC401915, CA724903, TC405652, CV779166, DR739994, TC459193, TC395506, TC395672, TC450972, TC392358, TC409599, TC419165, TC389092, TC376800, TC379853, TC420248, CK199846, TC370315, TC449504, TC403687, TC405440, TC403968, TC460795, CJ792862, TC422789, TC370885, TC407232, TC440819, TC416492, TC393015, TC426980, TC377225, TC404620, TC382858, TC403977, CA646741, TC429865, TC434820, TC379711, TC379903, TC377308, TC406749, TC392272, TC401244, CA730421, TC400114, TC413339, TC408907, TC375539, TC385013, TC380083, TC386963, TC412732, TC381191, TC369010, TC402080, TC373518, TC370350, TC383909, TC376527, TC374940, TC419584, CN010697, TC374392, TC378568, TC369983, CV761628, TC404420, TC383235, TC373489, TC369199, TC419747, TC407304, CA676115, TC402186, BE415178, TC429062, TC423354, TC409344, TC413043, BJ243383, TC400484, CK214224, TC368603, TC398040, TC374964, TC375234, CA709177, TC385815, TC409187, TC429374, TC425690, TC375253, TC423182, TC381218, TC401124, TC375873, TC444302, TC435224, CJ563078, TC394850, TC384344, TC392297, TC388520, TC443712, TC379422, TC378956, TC411191, TC426326, TC375431, TC424252, TC391995, TC415501, TC422841, TC387064, TC445649, CA498496, TC394796, TC382777, TC440499, TC435533, TC407349, TC397994, TC414243, TC403234, TC429437, TC430988, TC371738, TC413771, TC387621, TC398633, TC460689, TC380125, TC410126, TC369064, TC432154, TC420735, TC404371, TC402939, TC427405, TC449256, TC373615, TC383949, TC378409, TC416685, TC451342, TC372664, TC399986, TC389375, TC433812, TC370114, TC406038, TC390319, TC376874, TC424154, TC458807, TC459245, TC429871, TC369928, TC419438, TC376490, TC384454, TC411287, TC386237, TC388751, TC431722, AL820497, TC435909, TC399471, TC408126, TC430096, TC399972, TC434396, TC421880, TC386414, TC389590, CJ854725, TC440636, TC403986, TC406193, TC383763, CD876572, TC369736, TC386279, CK197833, TC391785, TC377427, TC382786, TC409650, TC388158, TC418340, TC405773, TC382830, TC377653, DR739471, TC402534, TC371600, TC382129, TC391840, TC390285, TC412989, TC387766, TC407340, TC398026, TC406584, CV065343, TC402440, TC396636, TC375146, TC454407, CK199175, TC394661, TC389044, TC383176, TC449463, TC371037, TC388688, TC403328, TC408095, TC439939, TC375268, TC452503, TC388547, TC420877, TC429048, TC398978, TC388976, TC396365, TC379069, TC385526, TC423576, TC375530, TC377635, TC402668, TC435799, TC438243, TC392887, TC370210, TC375918, TC417363, TC400592, TC387710, TC413700, TC385515, TC423880, TC451285, BG263159, TC458503, TC387194, TC391865, TC434689, TC441995, CK211469, CB307332, TC404842, TC381296, TC411817, TC436290, TC390489, TC421341, TC432504, TC383143 |
| [Extrinsic to plasma membrane](http://amigo.geneontology.org/cgi-bin/amigo/go.cgi?action=query&view=query&query=GO:0019897&search_constraint=terms) | TC387410, TC423110, TC378790, TC371387, TC379536 |
| [Chromosome, centromeric region](http://amigo.geneontology.org/cgi-bin/amigo/go.cgi?action=query&view=query&query=GO:0000775&search_constraint=terms) | TC394796, TC370315 |
| [Integral to endoplasmic reticulum membrane](http://amigo.geneontology.org/cgi-bin/amigo/go.cgi?action=query&view=query&query=GO:0030176&search_constraint=terms) | TC409343, TC412569 |
| [Intrinsic to endoplasmic reticulum membrane](http://amigo.geneontology.org/cgi-bin/amigo/go.cgi?action=query&view=query&query=GO:0031227&search_constraint=terms) | TC409343, TC412569 |
| [Eukaryotic translation initiation factor 3 complex](http://amigo.geneontology.org/cgi-bin/amigo/go.cgi?action=query&view=query&query=GO:0005852&search_constraint=terms) | TC369726, TC374392 |
| [Asymmetric synapse](http://amigo.geneontology.org/cgi-bin/amigo/go.cgi?action=query&view=query&query=GO:0032279&search_constraint=terms) | CK211589, TC413571 |
| [Fungal-type cell wall](http://amigo.geneontology.org/cgi-bin/amigo/go.cgi?action=query&view=query&query=GO:0009277&search_constraint=terms) | TC405511, TC407076, TC407978, TC380882, TC423880, TC423354, TC403588, TC438243 |
| [Condensed chromosome](http://amigo.geneontology.org/cgi-bin/amigo/go.cgi?action=query&view=query&query=GO:0000793&search_constraint=terms) | TC394796, TC370315 |
| [U6 snRNP](http://amigo.geneontology.org/cgi-bin/amigo/go.cgi?action=query&view=query&query=GO:0005688&search_constraint=terms) | TC387710, TC404606 |
| [Dendritic spine](http://amigo.geneontology.org/cgi-bin/amigo/go.cgi?action=query&view=query&query=GO:0043197&search_constraint=terms) | CK211589, TC413571 |
| [Neuron spine](http://amigo.geneontology.org/cgi-bin/amigo/go.cgi?action=query&view=query&query=GO:0044309&search_constraint=terms) | CK211589, TC413571 |
| [Organellar ribosome](http://amigo.geneontology.org/cgi-bin/amigo/go.cgi?action=query&view=query&query=GO:0000313&search_constraint=terms) | TC396451, TC368603, TC371970 |
| [Barrier septum](http://amigo.geneontology.org/cgi-bin/amigo/go.cgi?action=query&view=query&query=GO:0000935&search_constraint=terms) | TC438243, TC407978, TC370315 |
| [Heterotrimeric G-protein complex](http://amigo.geneontology.org/cgi-bin/amigo/go.cgi?action=query&view=query&query=GO:0005834&search_constraint=terms) | TC423110, TC371387, TC379536 |
| [Cell surface](http://amigo.geneontology.org/cgi-bin/amigo/go.cgi?action=query&view=query&query=GO:0009986&search_constraint=terms) | TC421914, TC405511, TC395723, TC378271, TC407978, TC461622, TC423354, TC438243, TC403588, TC374240, TC407076, TC394118, TC423880, TC380882, TC425690, CJ550278 |
| [Cytoplasmic microtubule](http://amigo.geneontology.org/cgi-bin/amigo/go.cgi?action=query&view=query&query=GO:0005881&search_constraint=terms) | TC373637, TC379171, TC382045, TC371242 |
| [Chromatin remodeling complex](http://amigo.geneontology.org/cgi-bin/amigo/go.cgi?action=query&view=query&query=GO:0016585&search_constraint=terms) | TC383701, TC395723, TC384735, TC441343, TC391128, TC380433, CK211589, TC381817, CJ550278, TC413571 |
| [Axon](http://amigo.geneontology.org/cgi-bin/amigo/go.cgi?action=query&view=query&query=GO:0030424&search_constraint=terms) | TC421914, TC374240, TC395723, TC378271, TC461622, TC380882, TC380433, CJ550278, TC403588, TC382045 |
| [Peroxisome](http://amigo.geneontology.org/cgi-bin/amigo/go.cgi?action=query&view=query&query=GO:0005777&search_constraint=terms) | TC380416, TC384194, TC372580, TC431198, TC456784, TC374996 |
| [Microbody](http://amigo.geneontology.org/cgi-bin/amigo/go.cgi?action=query&view=query&query=GO:0042579&search_constraint=terms) | TC380416, TC384194, TC372580, TC431198, TC456784, TC374996 |
| [Cilium](http://amigo.geneontology.org/cgi-bin/amigo/go.cgi?action=query&view=query&query=GO:0005929&search_constraint=terms) | TC407456, TC435055 |
| [Nuclear periphery](http://amigo.geneontology.org/cgi-bin/amigo/go.cgi?action=query&view=query&query=GO:0034399&search_constraint=terms) | TC423804, TC377061 |
| [Perinuclear region of cytoplasm](http://amigo.geneontology.org/cgi-bin/amigo/go.cgi?action=query&view=query&query=GO:0048471&search_constraint=terms) | TC438243, TC373613, TC432185 |
| [Dendritic shaft](http://amigo.geneontology.org/cgi-bin/amigo/go.cgi?action=query&view=query&query=GO:0043198&search_constraint=terms) | CK211589, TC413571 |
| [Pseudopodium](http://amigo.geneontology.org/cgi-bin/amigo/go.cgi?action=query&view=query&query=GO:0031143&search_constraint=terms) | TC390630, TC393948 |
| [Cell septum](http://amigo.geneontology.org/cgi-bin/amigo/go.cgi?action=query&view=query&query=GO:0030428&search_constraint=terms) | TC438243, TC407978, TC370315 |
| [Membrane](http://amigo.geneontology.org/cgi-bin/amigo/go.cgi?action=query&view=query&query=GO:0016020&search_constraint=terms) | TC410352, TC407978, TC373958, TC411284, CV775873, TC377766, TC383139, TC398383, TC368599, TC389842, TC398052, TC419614, TC391749, CA710880, TC421871, TC398862, TC394459, TC371242, TC384738, TC423252, TC397660, TC404978, TC381613, TC389363, TC388410, BJ282766, TC458983, TC458562, TC411471, TC421914, TC434570, TC387410, TC422842, TC418414, TC390792, TC386396, TC395453, TC410182, TC406594, TC390569, TC418728, TC407076, TC403872, TC400595, TC398714, BQ294582, CJ550278, BE591166, TC403679, TC386305, TC401915, GH732878, TC398304, TC425878, TC393100, TC433957, TC421954, TC412569, DR739994, TC395672, TC394916, TC378274, CK217367, TC378153, TC410144, TC379853, TC411008, TC370315, TC395723, TC405440, TC379536, TC391621, TC398730, TC398514, TC377225, TC392269, TC434000, TC398379, TC385783, TC376758, BQ607161, TC406807, TC382045, TC416906, TC434820, TC379711, TC400260, TC372796, TC378790, TC392272, TC377061, TC417077, TC417388, TC386313, TC375539, TC369844, CA606693, CK211707, TC370912, TC386963, BJ258087, TC370178, TC373518, TC392778, TC396751, TC397312, CK211589, TC383909, TC373678, CA615187, TC405041, TC405784, TC399919, TC407572, TC372175, TC390150, TC406708, TC419747, TC418691, TC409459, TC443289, TC386646, TC369072, TC416471, CN010359, TC423354, TC441241, TC449724, TC413571, CA729339, TC461971, TC423348, TC410108, TC429374, TC425690, TC381218, TC422425, CV766349, TC384344, TC387511, TC439154, CA613620, TC400362, TC455532, TC427600, TC401293, TC375612, TC430501, TC411191, TC387683, TC434831, TC432185, TC426326, TC381462, TC377749, TC392263, TC432130, TC384625, TC404158, TC394796, CK201269, TC416381, TC372677, TC429341, TC374240, TC425841, TC397994, TC387579, TC422288, TC388914, TC387621, TC408309, TC446235, TC378271, TC380433, TC432154, TC420735, TC415808, TC386961, TC395312, TC397562, TC387319, TC416685, TC369182, TC418365, TC387344, TC389090, TC389375, TC373002, TC404413, TC382655, TC433812, CK200433, TC370114, TC373994, TC406038, TC374731, TC395351, TC392709, TC456619, CK210754, TC407191, TC370727, TC384454, TC397291, TC430555, TC386237, TC415365, TC409343, TC393904, TC390994, TC387191, TC403852, TC447260, TC428066, TC399972, TC395303, TC400755, TC405511, TC376420, TC458987, TC377190, TC423110, TC401210, TC369736, DR737360, TC390135, TC390566, TC387135, TC388049, TC409650, TC380662, TC374485, TC413462, TC393960, TC418340, TC449064, DR734732, DR739471, TC443387, TC378225, TC390630, TC412989, TC418716, TC390402, TC402440, TC408381, TC394307, TC375146, TC373583, TC406371, TC389589, TC421872, TC449463, TC386422, TC388688, TC384071, TC375268, TC452503, TC388547, DR735108, TC412150, TC388822, TC377496, TC396365, TC429771, TC423576, TC423010, TC416658, TC384678, TC393561, TC393948, TC435799, TC386322, TC391671, TC393554, TC407614, TC391461, TC459656, TC382928, TC438243, TC417363, TC396487, TC458699, TC440632, TC403929, TC385515, TC394118, TC380882, TC373848, TC423880, TC377410, TC389816, TC371387, TC384194, TC439472, TC446092, TC393820, TC461622, TC437163, TC379470, TC403588, TC376933, TC385659, TC397729, TC382910, TC444402, TC394728, TC417970, TC394693, TC411817, TC396895, TC393106, TC401758, TC396460, TC431306, TC389661, TC428944 |
| [Cell tip](http://amigo.geneontology.org/cgi-bin/amigo/go.cgi?action=query&view=query&query=GO:0051286&search_constraint=terms) | TC438243, TC407978 |
| [Membrane coat](http://amigo.geneontology.org/cgi-bin/amigo/go.cgi?action=query&view=query&query=GO:0030117&search_constraint=terms) | TC385515, TC409650 |
| [Yesicle coat](http://amigo.geneontology.org/cgi-bin/amigo/go.cgi?action=query&view=query&query=GO:0030120&search_constraint=terms) | TC385515, TC409650 |
| [Integral to organelle membrane](http://amigo.geneontology.org/cgi-bin/amigo/go.cgi?action=query&view=query&query=GO:0031301&search_constraint=terms) | TC409343, TC412569 |
| [Coated membrane](http://amigo.geneontology.org/cgi-bin/amigo/go.cgi?action=query&view=query&query=GO:0048475&search_constraint=terms) | TC385515, TC409650 |
| [Zeta DNA polymerase complex](http://amigo.geneontology.org/cgi-bin/amigo/go.cgi?action=query&view=query&query=GO:0016035&search_constraint=terms) | TC413571, TC371242 |
| [Proteasome core complex, alpha-subunit complex](http://amigo.geneontology.org/cgi-bin/amigo/go.cgi?action=query&view=query&query=GO:0019773&search_constraint=terms) | TC397562, TC420420 |
| [Intrinsic to organelle Membrane](http://amigo.geneontology.org/cgi-bin/amigo/go.cgi?action=query&view=query&query=GO:0031300&search_constraint=terms) | TC409343, TC412569 |
| [DNA polymerase complex](http://amigo.geneontology.org/cgi-bin/amigo/go.cgi?action=query&view=query&query=GO:0042575&search_constraint=terms) | TC413571, TC371242 |
| [External side of plasma membrane](http://amigo.geneontology.org/cgi-bin/amigo/go.cgi?action=query&view=query&query=GO:0009897&search_constraint=terms) | TC421914, TC374240, TC395723, TC425690, CJ550278, TC378271, TC394118 |
| [Spindle microtubule](http://amigo.geneontology.org/cgi-bin/amigo/go.cgi?action=query&view=query&query=GO:0005876&search_constraint=terms) | TC379171, TC382045, TC371242 |
| [Preribosome](http://amigo.geneontology.org/cgi-bin/amigo/go.cgi?action=query&view=query&query=GO:0030684&search_constraint=terms) | TC387410, TC459656, TC406807, TC456619, TC372664 |
| [Mitochondrial matrix](http://amigo.geneontology.org/cgi-bin/amigo/go.cgi?action=query&view=query&query=GO:0005759&search_constraint=terms) | TC374230, TC396451, TC388049, TC368603, TC407340, TC371970, TC382045, TC391128, TC375918 |
| [Mitochondrial lumen](http://amigo.geneontology.org/cgi-bin/amigo/go.cgi?action=query&view=query&query=GO:0031980&search_constraint=terms) | TC374230, TC396451, TC388049, TC368603, TC407340, TC371970, TC382045, TC391128, TC375918 |
| [Axon part](http://amigo.geneontology.org/cgi-bin/amigo/go.cgi?action=query&view=query&query=GO:0033267&search_constraint=terms) | TC380433, TC403588, TC461622, TC380882 |
| [NuRD complex](http://amigo.geneontology.org/cgi-bin/amigo/go.cgi?action=query&view=query&query=GO:0016581&search_constraint=terms) | CK211589, TC384735, TC381817, TC441343, TC413571 |
| [Small-subunit processome](http://amigo.geneontology.org/cgi-bin/amigo/go.cgi?action=query&view=query&query=GO:0032040&search_constraint=terms) | TC387410, TC459656, TC406807, TC372664 |
| [Myofibril](http://amigo.geneontology.org/cgi-bin/amigo/go.cgi?action=query&view=query&query=GO:0030016&search_constraint=terms) | TC459656, TC406807, TC410126, CJ944525 |
| [Contractile fiber](http://amigo.geneontology.org/cgi-bin/amigo/go.cgi?action=query&view=query&query=GO:0043292&search_constraint=terms) | TC459656, TC406807, TC410126, CJ944525 |
| [Contractile fiber part](http://amigo.geneontology.org/cgi-bin/amigo/go.cgi?action=query&view=query&query=GO:0044449&search_constraint=terms) | TC459656, TC406807, TC410126, CJ944525 |
| [Extrinsic to membrane](http://amigo.geneontology.org/cgi-bin/amigo/go.cgi?action=query&view=query&query=GO:0019898&search_constraint=terms) | TC387410, TC376420, TC423110, TC378790, TC371387, TC379536 |
| [Spliceosomal complex](http://amigo.geneontology.org/cgi-bin/amigo/go.cgi?action=query&view=query&query=GO:0005681&search_constraint=terms) | TC387135, TC385233, TC405540, TC399352, TC388566 |
| [Dendrite](http://amigo.geneontology.org/cgi-bin/amigo/go.cgi?action=query&view=query&query=GO:0030425&search_constraint=terms) | CK211589, TC413571, TC382045 |
| [SCF ubiquitin ligase complex](http://amigo.geneontology.org/cgi-bin/amigo/go.cgi?action=query&view=query&query=GO:0019005&search_constraint=terms) | TC388649, TC394820 |
| [Cullin-RING ubiquitin ligase complex](http://amigo.geneontology.org/cgi-bin/amigo/go.cgi?action=query&view=query&query=GO:0031461&search_constraint=terms) | TC388649, TC394820 |
| [Cytoplasm](http://amigo.geneontology.org/cgi-bin/amigo/go.cgi?action=query&view=query&query=GO:0005737&search_constraint=terms) | BJ284275, TC410352, TC390944, TC370791, TC453487, TC375864, TC432212, TC415588, TC389842, BQ838511, TC398052, TC387861, TC391749, TC374098, TC400388, TC457112, TC431932, CK215979, TC376351, TC430471, TC439423, TC383028, TC379171, TC388410, BJ282766, TC416493, TC388136, TC458562, TC393436, TC417260, CK214103, TC433844, CK209294, TC410063, TC421143, TC386396, TC378615, TC373702, TC456784, TC395453, TC392329, TC410182, TC445939, CA614761, CJ550278, BE591166, TC411128, GH732878, TC397444, TC412569, TC415483, CA632212, TC393523, TC435852, TC413027, TC372549, TC394820, TC398731, TC411784, TC387817, TC395069, TC413460, TC411008, CK214702, TC426907, TC395723, TC369415, TC387981, TC385233, TC391950, TC386707, TC376248, BE217043, TC374409, TC402069, TC381943, TC420358, CV770684, TC398209, BQ607161, TC372845, TC382045, TC431175, TC402308, TC378790, TC417077, TC385009, CV759879, TC376625, TC390016, TC380474, CA606693, TC370912, TC432369, TC385714, TC412212, TC375124, TC392778, TC397312, TC405030, CA615187, TC405041, TC444549, TC397019, BJ317882, TC406469, TC399919, TC407572, TC413118, TC406708, TC373898, TC418691, TC409459, TC420337, TC391360, TC416471, TC419919, TC372761, TC406809, TC410569, TC437137, CN010359, TC373001, TC402603, TC449724, TC395914, TC372867, TC397909, CA729339, TC430163, TC389993, TC426864, TC408312, TC391156, CA613620, TC400362, TC398154, TC401293, TC369325, TC429747, TC375612, TC430501, CJ867263, TC400108, TC406106, TC377749, TC396451, TC431198, TC424344, TC429217, TC372677, CJ868963, TC374240, TC376306, TC425841, TC405982, TC387579, TC420460, TC428008, TC427006, TC380943, TC376202, TC403885, TC405946, TC460907, TC394451, TC395312, CV772029, TC387344, TC389090, TC389139, TC412483, TC382655, TC389144, TC373994, TC395351, TC392709, TC372563, TC418032, CK162413, TC397291, TC461977, TC411908, CA598430, TC459008, TC380329, TC376930, TC391516, TC409343, TC389696, TC403852, TC382742, TC377190, TC443814, TC423110, TC391613, TC385365, TC430544, TC382339, TC379357, TC371003, TC387135, TC378437, TC438587, TC375760, TC381923, TC426016, DR734732, TC387686, TC373251, TC413124, TC388566, TC399245, TC373429, TC418716, TC392447, TC421467, TC379023, TC386519, TC394219, TC383047, TC417992, TC385090, TC407964, TC394307, TC373583, TC406371, TC382139, TC392363, TC381988, TC370106, TC388819, TC405475, TC386401, TC422022, TC386344, TC452356, TC373637, TC370044, TC421872, TC386422, TC442623, CJ776780, TC422583, TC400379, TC393037, CJ730226, TC421733, TC383570, TC396228, TC379965, TC396650, TC384678, TC456370, TC404053, TC400562, TC371970, TC375726, TC459656, DR734904, TC378783, TC379436, TC377765, TC403929, TC457991, TC394118, TC378601, TC373848, TC426870, TC437918, TC387007, TC375935, TC418091, TC389816, TC372530, TC392323, TC370907, TC384194, TC413066, TC371172, TC390211, TC372167, TC391641, TC461622, TC393426, TC400056, TC405033, TC379470, TC419090, TC385659, TC382910, TC432678, TC389451, TC458892, DR740372, TC394693, TC416495, TC401758, TC428944, TC449093, TC379942, TC434821, TC374404, TC396230, BQ295499, TC377766, TC394965, TC368599, TC391648, TC376937, TC433162, TC400317, TC404978, TC389363, TC371248, TC379338, TC433116, TC409167, TC416442, TC374679, TC387410, TC378072, TC406516, TC406986, TC371145, TC419057, TC451950, TC380695, TC398714, TC381619, TC424179, TC394028, TC398970, TC457126, TC456854, TC403679, TC418747, TC440803, TC403580, TC386305, TC424044, TC372667, TC433957, TC392432, TC386440, TC423239, TC425821, TC370962, TC403055, TC406236, TC382080, TC391962, TC391128, TC445767, TC387501, TC446038, TC374852, TC369830, TC423804, TC418685, CK213497, TC414899, TC436309, TC398514, TC391900, TC392033, TC423091, TC447694, TC380825, TC412520, TC406807, TC395090, TC441239, TC416906, TC416438, TC423265, TC417388, TC440066, TC386313, TC404179, TC389108, TC369844, TC420579, CK211707, TC392126, TC402340, TC431201, CK217689, TC370392, CV761547, TC396751, CK211589, TC411116, TC388718, TC383701, TC452793, TC385780, TC405784, TC395566, TC372175, TC382734, TC405896, TC385927, TC369072, TC385513, TC439501, TC405356, TC444586, TC449448, TC381596, TC377441, TC403264, TC369348, TC461971, TC435825, TC422176, TC388232, TC445281, TC376263, TC415521, TC384010, TC416169, TC409043, TC377290, TC409190, TC403537, TC381255, TC379924, TC418399, TC431382, TC387683, TC409855, TC434831, TC406194, TC427161, TC373914, TC450285, TC382024, TC369899, TC404998, TC378846, TC372330, TC448051, TC381068, CD930656, TC403936, TC451276, TC444375, TC387050, TC415685, TC388914, TC415056, TC433488, TC377178, TC377438, TC394716, TC397027, TC456160, TC394670, TC378271, TC377292, TC380433, TC451621, TC435281, TC384219, TC397562, TC428853, TC404413, TC408192, CJ626653, TC372046, CK210754, TC407191, TC370727, TC417769, TC449235, TC410147, TC414564, TC405910, TC431603, TC387191, TC407395, TC447801, TC380416, TC427981, CA500690, TC398536, TC400755, TC403717, TC372701, TC376420, TC458987, TC404843, TC382737, TC370633, TC390135, TC411480, TC417755, TC420394, TC432320, TC387170, TC439225, TC380662, TC419727, TC394263, TC436772, TC395298, TC394253, TC412094, CV782550, TC389162, TC376630, CK212850, TC389678, TC399432, TC434006, TC434949, TC369726, CA727263, TC379241, TC405295, TC392465, TC417480, TC455676, TC373962, TC409208, TC375813, TC379343, TC374741, TC429809, TC386591, TC421704, TC420201, TC381963, TC456247, TC374879, GH728664, TC385701, TC418759, TC431825, TC377496, TC368549, TC401158, TC387133, TC410078, TC415942, TC393561, TC393692, TC391671, TC373787, BQ160837, TC382928, TC397415, TC370507, TC369637, TC396487, TC436832, TC458699, TC380882, TC372580, TC444588, TC400721, TC451657, TC445214, TC451519, TC393820, TC435808, TC449739, TC416981, BE586004, TC399408, TC417970, TC426743, TC396895, TC382342, TC393106, TC428932, TC431306, TC396460, TC397676, TC379968, TC399730, TC368580, TC441536, TC373613, TC386040, TC407978, TC390393, TC444546, TC392283, TC390516, TC416529, TC391946, TC420420, TC417106, TC432124, CA710880, TC452945, TC375593, TC421871, TC371242, TC394459, TC423252, TC384738, TC416154, TC397660, CV766916, TC390379, TC407404, TC374996, TC393781, TC421914, TC435210, TC398538, TC380063, TC418414, CJ944525, TC396772, TC378054, TC453207, TC419273, TC461607, TC409843, TC437445, TC403872, TC407076, TC371357, TC406207, TC389586, TC395746, TC439210, TC398304, TC393100, CV771134, BE431108, TC417012, TC391447, TC455515, TC394916, TC397793, TC404914, TC391130, TC378274, CK217367, TC378153, TC379990, TC378333, TC444410, TC435863, BQ788843, TC452050, CJ845228, TC379536, TC391621, TC389718, TC429713, CK201148, TC386088, TC389534, TC413392, TC422142, TC398379, TC376758, CJ815867, TC369628, TC441343, TC400260, TC399057, TC408191, TC378432, TC377061, TC389843, TC388317, CD937281, TC416801, AL827131, TC379312, TC391438, TC381268, TC412317, TC371325, TC439708, TC428113, TC373563, TC370619, BJ258087, TC421345, TC422007, TC370178, TC405695, TC388403, TC373678, TC416695, TC410690, TC374461, TC443289, TC386646, TC399841, TC369687, TC458205, TC441241, TC433557, TC413571, TC415803, TC408299, TC410108, TC454526, TC398830, TC399352, TC422425, TC371129, TC372654, TC428992, TC420816, TC384162, TC383739, TC399342, TC432185, TC381583, TC455736, TC432130, TC375665, TC385205, TC427986, TC422645, TC425957, TC445166, BG904091, TC421162, TC398121, TC389168, TC424204, TC439324, TC422288, TC424317, TC393814, TC376220, TC373402, TC446235, TC402545, TC393830, TC444899, TC386961, TC398606, TC387319, TC396134, TC369182, TC418365, TC373204, TC370347, TC373002, TC375554, CK200433, TC393970, TC374731, TC452494, TC456619, TC388228, TC444138, TC377373, TC390994, TC393437, TC412205, TC426916, TC391143, TC381463, TC410066, TC414090, TC395303, TC442140, TC405511, TC386410, TC392203, TC393890, TC442727, TC390762, DR737360, TC381007, TC411684, TC382728, TC388049, TC404850, TC370033, TC433940, TC374485, TC393960, TC390436, TC385326, TC387707, TC409077, TC443387, TC415083, TC370470, TC386535, TC390630, TC390402, TC374726, TC415954, TC376790, TC398592, TC389589, TC378911, TC452762, TC407490, TC374164, TC377993, DR735108, TC438657, TC412150, TC426078, TC388822, TC429771, TC375069, TC386639, TC416658, TC424962, TC379944, TC374230, TC391913, TC375834, TC393948, TC403624, TC400638, TC393554, TC400477, TC407614, TC384049, TC384357, TC460615, TC403573, TC411941, TC385445, TC395841, TC449043, TC379635, TC392247, TC373259, TC433589, TC382338, TC375914, TC398343, TC377253, TC404426, TC439472, TC377944, TC381817, TC403588, TC394728, TC403803, TC418850, TC440526, TC436944, TC371341, TC381279, TC389661, CA726837, TC387386, TC383820, TC396657, TC380333, TC377064, TC397258, TC426838, CV775873, TC451511, TC431330, TC384373, TC416069, TC427210, TC422606, TC375313, TC399271, TC398862, DR739303, TC434442, TC413263, TC411471, TC431660, TC434570, TC378878, TC390792, TC394020, TC388950, TC374669, TC419222, TC410194, TC369371, TC448840, TC392875, TC409005, TC408228, TC384735, TC418728, TC405540, TC390569, TC391948, BQ294582, CA724903, TC401915, TC405652, CV779166, DR739994, TC395506, TC395672, TC392358, TC450972, TC409599, TC419165, TC389092, TC376800, TC379853, TC420248, CK199846, TC370315, TC403687, TC405440, TC403968, TC460795, TC422789, TC370885, TC407232, TC440819, TC416492, TC393015, TC426980, TC377225, TC382858, TC404620, TC429865, TC434820, TC379711, TC379903, TC377308, TC406749, TC392272, TC401244, TC400114, TC413339, TC408907, TC375539, TC385013, TC380083, TC386963, TC412732, TC381191, TC369010, TC402080, TC373518, TC370350, TC383909, TC374940, CN010697, TC374392, TC378568, TC369983, CV761628, TC404420, TC383235, TC373489, TC369199, TC419747, TC407304, CA676115, TC402186, BE415178, TC429062, TC423354, TC409344, TC413043, BJ243383, TC400484, CK214224, TC368603, TC398040, TC374964, TC375234, TC385815, TC409187, TC429374, TC425690, TC375253, TC423182, TC401124, TC375873, TC444302, TC435224, CJ563078, TC394850, TC384344, TC392297, TC388520, TC443712, TC379422, TC378956, TC411191, TC426326, TC375431, TC424252, TC391995, TC415501, TC422841, TC387064, TC445649, CA498496, TC382777, TC440499, TC435533, TC407349, TC397994, TC414243, TC403234, TC429437, TC430988, TC371738, TC413771, TC387621, TC398633, TC460689, TC380125, TC410126, TC369064, TC432154, TC420735, TC404371, TC402939, TC427405, TC449256, TC373615, TC383949, TC378409, TC416685, TC451342, TC372664, TC399986, TC389375, TC433812, TC370114, TC390319, TC406038, TC376874, TC424154, TC458807, TC369928, TC429871, TC419438, TC376490, TC384454, TC411287, TC386237, TC388751, AL820497, TC435909, TC431722, TC399471, TC408126, TC430096, TC399972, TC434396, TC421880, TC386414, TC389590, CJ854725, TC440636, TC403986, TC383763, TC369736, TC386279, CK197833, TC391785, TC377427, TC382786, TC409650, TC388158, TC418340, TC405773, TC382830, TC377653, DR739471, TC402534, TC371600, TC382129, TC391840, TC390285, TC412989, TC387766, TC407340, TC398026, TC406584, CV065343, TC402440, TC396636, TC375146, TC454407, TC394661, TC389044, TC383176, TC371037, TC449463, TC388688, TC403328, TC408095, TC439939, TC375268, TC452503, TC388547, TC420877, TC429048, TC398978, TC388976, TC396365, TC379069, TC385526, TC423576, TC377635, TC402668, TC435799, TC438243, TC392887, TC370210, TC375918, TC417363, TC400592, TC385515, TC423880, TC451285, BG263159, TC458503, TC387194, TC391865, TC434689, TC441995, CB307332, CK211469, TC404842, TC381296, TC411817, TC436290, TC390489, TC421341, TC432504, TC383143 |
| [Cell leading edge](http://amigo.geneontology.org/cgi-bin/amigo/go.cgi?action=query&view=query&query=GO:0031252&search_constraint=terms) | TC390630, TC387410, TC403588, TC393948, TC461622, TC382045, TC380882 |
| [Transcription factor complex](http://amigo.geneontology.org/cgi-bin/amigo/go.cgi?action=query&view=query&query=GO:0005667&search_constraint=terms) | TC460615, TC374230, TC435799, TC404413, TC400362, TC370114, TC409459, TC417077, TC393960, TC382045 |
| [Kinetochore microtubule](http://amigo.geneontology.org/cgi-bin/amigo/go.cgi?action=query&view=query&query=GO:0005828&search_constraint=terms) | TC379171, TC371242 |
| [Nucleolar part](http://amigo.geneontology.org/cgi-bin/amigo/go.cgi?action=query&view=query&query=GO:0044452&search_constraint=terms) | TC390630, TC387410, TC394118 |
| [Actin filament](http://amigo.geneontology.org/cgi-bin/amigo/go.cgi?action=query&view=query&query=GO:0005884&search_constraint=terms) | TC390630, TC393948 |
| [Cell wall](http://amigo.geneontology.org/cgi-bin/amigo/go.cgi?action=query&view=query&query=GO:0005618&search_constraint=terms) | TC405511, TC382830, TC435224, TC407076, TC407978, CK212850, CK215979, TC380882, TC423880, TC373637, TC423354, TC438243, TC403588 |
| [External encapsulating structure](http://amigo.geneontology.org/cgi-bin/amigo/go.cgi?action=query&view=query&query=GO:0030312&search_constraint=terms) | TC405511, TC382830, TC435224, TC407076, TC407978, CK212850, CK215979, TC380882, TC423880, TC373637, TC423354, TC438243, TC403588 |
| [Transcriptional repressor complex](http://amigo.geneontology.org/cgi-bin/amigo/go.cgi?action=query&view=query&query=GO:0017053&search_constraint=terms) | CK211589, TC384735, TC381817, TC441343, TC413571, TC418850 |
| [Ubiquitin ligase complex](http://amigo.geneontology.org/cgi-bin/amigo/go.cgi?action=query&view=query&query=GO:0000151&search_constraint=terms) | TC388649, TC394820, TC393960, TC379536 |
| [Nuclear pore](http://amigo.geneontology.org/cgi-bin/amigo/go.cgi?action=query&view=query&query=GO:0005643&search_constraint=terms) | TC387135, TC390630, TC409459, TC370114, TC383909, TC449463, TC393948 |
| [Pore complex](http://amigo.geneontology.org/cgi-bin/amigo/go.cgi?action=query&view=query&query=GO:0046930&search_constraint=terms) | TC387135, TC390630, TC409459, TC370114, TC383909, TC449463, TC393948 |
| [Polar microtubule](http://amigo.geneontology.org/cgi-bin/amigo/go.cgi?action=query&view=query&query=GO:0005827&search_constraint=terms) | TC379171, TC371242 |
| [Endoplasmic reticulum membrane](http://amigo.geneontology.org/cgi-bin/amigo/go.cgi?action=query&view=query&query=GO:0005789&search_constraint=terms) | TC409343, TC387191, TC390402, TC378790, TC405041, TC412569 |
| [Nuclear membrane-endoplasmic reticulum network](http://amigo.geneontology.org/cgi-bin/amigo/go.cgi?action=query&view=query&query=GO:0042175&search_constraint=terms) | TC409343, TC387191, TC390402, TC378790, TC405041, TC412569 |
| [Cytoplasmic part](http://amigo.geneontology.org/cgi-bin/amigo/go.cgi?action=query&view=query&query=GO:0044444&search_constraint=terms) | BJ284275, TC410352, TC390944, TC370791, TC453487, TC375864, TC432212, TC415588, TC389842, BQ838511, TC398052, TC387861, TC391749, TC374098, TC400388, TC457112, TC431932, CK215979, TC376351, TC430471, TC439423, TC383028, TC379171, TC388410, BJ282766, TC416493, TC388136, TC458562, TC393436, TC417260, CK214103, TC433844, CK209294, TC410063, TC421143, TC386396, TC378615, TC373702, TC456784, TC395453, TC392329, TC410182, TC445939, CA614761, CJ550278, BE591166, TC411128, GH732878, TC397444, TC412569, TC415483, CA632212, TC393523, TC435852, TC413027, TC372549, TC394820, TC398731, TC411784, TC387817, TC395069, TC413460, TC411008, CK214702, TC426907, TC395723, TC369415, TC387981, TC385233, TC391950, TC386707, TC376248, BE217043, TC374409, TC402069, TC381943, TC420358, CV770684, TC398209, BQ607161, TC372845, TC382045, TC431175, TC402308, TC378790, TC417077, TC385009, CV759879, TC376625, TC390016, TC380474, CA606693, TC432369, TC385714, TC412212, TC375124, TC392778, TC397312, TC405030, CA615187, TC405041, TC444549, BJ317882, TC406469, TC399919, TC407572, TC413118, TC406708, TC373898, TC418691, TC409459, TC420337, TC391360, TC416471, TC419919, TC372761, TC406809, TC410569, TC437137, CN010359, TC373001, TC402603, TC449724, TC395914, TC372867, TC397909, CA729339, TC430163, TC389993, TC426864, TC408312, TC391156, CA613620, TC400362, TC398154, TC401293, TC369325, TC429747, TC375612, TC430501, CJ867263, TC400108, TC406106, TC377749, TC396451, TC431198, TC424344, TC429217, TC372677, CJ868963, TC374240, TC376306, TC425841, TC405982, TC387579, TC420460, TC428008, TC427006, TC380943, TC376202, TC403885, TC405946, TC460907, TC394451, TC395312, CV772029, TC387344, TC389090, TC389139, TC412483, TC382655, TC389144, TC373994, TC395351, TC392709, TC372563, TC418032, CK162413, TC397291, TC461977, TC411908, CA598430, TC459008, TC380329, TC376930, TC391516, TC409343, TC389696, TC403852, TC382742, TC377190, TC443814, TC423110, TC391613, TC385365, TC430544, TC382339, TC379357, TC371003, TC387135, TC378437, TC438587, TC375760, TC381923, TC426016, DR734732, TC387686, TC413124, TC388566, TC399245, TC373429, TC418716, TC392447, TC421467, TC379023, TC386519, TC394219, TC383047, TC417992, TC385090, TC407964, TC394307, TC373583, TC406371, TC382139, TC392363, TC381988, TC370106, TC388819, TC405475, TC386401, TC422022, TC386344, TC452356, TC373637, TC370044, TC421872, TC386422, TC442623, CJ776780, TC422583, TC400379, TC393037, CJ730226, TC421733, TC383570, TC396228, TC379965, TC384678, TC456370, TC404053, TC400562, TC371970, TC375726, TC459656, TC378783, TC379436, TC377765, TC403929, TC457991, TC394118, TC373848, TC426870, TC437918, TC387007, TC375935, TC418091, TC389816, TC372530, TC392323, TC370907, TC384194, TC413066, TC371172, TC390211, TC372167, TC391641, TC461622, TC393426, TC400056, TC405033, TC379470, TC419090, TC385659, TC382910, TC432678, TC389451, TC458892, DR740372, TC394693, TC416495, TC401758, TC428944, TC449093, TC379942, TC434821, TC374404, TC396230, BQ295499, TC377766, TC394965, TC368599, TC391648, TC376937, TC433162, TC400317, TC404978, TC389363, TC371248, TC379338, TC433116, TC409167, TC416442, TC374679, TC387410, TC378072, TC406516, TC406986, TC371145, TC419057, TC451950, TC380695, TC398714, TC381619, TC424179, TC394028, TC398970, TC457126, TC456854, TC403679, TC418747, TC440803, TC403580, TC386305, TC424044, TC372667, TC433957, TC392432, TC386440, TC423239, TC425821, TC370962, TC403055, TC406236, TC382080, TC391962, TC391128, TC445767, TC387501, TC446038, TC374852, TC369830, TC423804, TC418685, CK213497, TC414899, TC436309, TC398514, TC391900, TC392033, TC423091, TC447694, TC380825, TC412520, TC406807, TC395090, TC441239, TC416906, TC416438, TC423265, TC417388, TC440066, TC386313, TC404179, TC389108, TC369844, TC420579, CK211707, TC392126, TC402340, TC431201, CK217689, TC370392, CV761547, TC396751, CK211589, TC411116, TC388718, TC383701, TC452793, TC385780, TC405784, TC395566, TC372175, TC382734, TC405896, TC385927, TC369072, TC385513, TC439501, TC405356, TC444586, TC449448, TC381596, TC377441, TC403264, TC369348, TC461971, TC435825, TC422176, TC388232, TC445281, TC376263, TC415521, TC384010, TC416169, TC409043, TC377290, TC409190, TC403537, TC381255, TC379924, TC418399, TC431382, TC387683, TC409855, TC434831, TC406194, TC427161, TC373914, TC450285, TC382024, TC369899, TC404998, TC372330, TC448051, TC381068, CD930656, TC403936, TC451276, TC444375, TC387050, TC415685, TC388914, TC415056, TC433488, TC377178, TC377438, TC394716, TC397027, TC456160, TC394670, TC378271, TC377292, TC380433, TC451621, TC435281, TC384219, TC397562, TC428853, TC404413, TC408192, CJ626653, TC372046, CK210754, TC407191, TC370727, TC417769, TC449235, TC410147, TC414564, TC405910, TC431603, TC387191, TC407395, TC447801, TC380416, TC427981, CA500690, TC398536, TC400755, TC403717, TC372701, TC376420, TC458987, TC404843, TC382737, TC370633, TC390135, TC411480, TC417755, TC420394, TC432320, TC387170, TC439225, TC380662, TC419727, TC394263, TC436772, TC395298, TC394253, TC412094, CV782550, TC389162, TC376630, CK212850, TC389678, TC399432, TC434006, TC434949, TC369726, CA727263, TC379241, TC405295, TC392465, TC455676, TC373962, TC409208, TC375813, TC379343, TC374741, TC429809, TC386591, TC421704, TC420201, TC381963, TC456247, TC374879, GH728664, TC385701, TC418759, TC431825, TC377496, TC368549, TC401158, TC387133, TC410078, TC415942, TC393561, TC393692, TC391671, TC373787, BQ160837, TC382928, TC397415, TC370507, TC369637, TC396487, TC436832, TC458699, TC380882, TC372580, TC444588, TC400721, TC451657, TC445214, TC451519, TC393820, TC449739, TC416981, BE586004, TC399408, TC417970, TC426743, TC396895, TC382342, TC393106, TC428932, TC431306, TC396460, TC397676, TC379968, TC399730, TC368580, TC441536, TC373613, TC386040, TC407978, TC390393, TC444546, TC392283, TC390516, TC416529, TC391946, TC420420, TC417106, TC432124, CA710880, TC452945, TC375593, TC421871, TC371242, TC394459, TC423252, TC384738, TC416154, TC397660, CV766916, TC390379, TC407404, TC374996, TC393781, TC421914, TC435210, TC398538, TC380063, TC418414, CJ944525, TC396772, TC378054, TC453207, TC419273, TC461607, TC409843, TC437445, TC403872, TC407076, TC371357, TC406207, TC389586, TC395746, TC439210, TC398304, TC393100, CV771134, BE431108, TC417012, TC391447, TC455515, TC394916, TC404914, TC391130, TC378274, CK217367, TC378153, TC379990, TC378333, TC444410, TC435863, BQ788843, TC452050, CJ845228, TC379536, TC391621, TC389718, TC429713, CK201148, TC386088, TC389534, TC413392, TC422142, TC398379, TC376758, CJ815867, TC441343, TC400260, TC399057, TC408191, TC378432, TC377061, TC389843, TC388317, CD937281, TC416801, AL827131, TC379312, TC391438, TC381268, TC412317, TC371325, TC439708, TC428113, TC373563, TC370619, BJ258087, TC421345, TC422007, TC370178, TC405695, TC388403, TC373678, TC416695, TC410690, TC374461, TC443289, TC386646, TC399841, TC369687, TC458205, TC441241, TC433557, TC413571, TC415803, TC408299, TC410108, TC454526, TC398830, TC399352, TC422425, TC371129, TC372654, TC428992, TC420816, TC384162, TC383739, TC399342, TC432185, TC381583, TC455736, TC432130, TC375665, TC385205, TC427986, TC422645, TC425957, TC445166, BG904091, TC421162, TC398121, TC389168, TC424204, TC422288, TC424317, TC393814, TC376220, TC373402, TC446235, TC402545, TC393830, TC444899, TC386961, TC398606, TC387319, TC396134, TC369182, TC418365, TC370347, TC373002, TC375554, CK200433, TC393970, TC374731, TC452494, TC456619, TC388228, TC444138, TC377373, TC390994, TC393437, TC412205, TC426916, TC391143, TC381463, TC410066, TC414090, TC395303, TC442140, TC405511, TC386410, TC392203, TC393890, TC442727, TC390762, DR737360, TC381007, TC411684, TC382728, TC388049, TC404850, TC370033, TC433940, TC374485, TC393960, TC390436, TC385326, TC387707, TC409077, TC443387, TC415083, TC370470, TC386535, TC390630, TC390402, TC374726, TC415954, TC376790, TC398592, TC389589, TC378911, TC452762, TC407490, TC374164, TC377993, DR735108, TC438657, TC412150, TC426078, TC388822, TC429771, TC375069, TC386639, TC416658, TC424962, TC379944, TC374230, TC391913, TC375834, TC393948, TC403624, TC400638, TC393554, TC407614, TC384049, TC384357, TC460615, TC403573, TC411941, TC385445, TC395841, TC449043, TC379635, TC392247, TC373259, TC433589, TC382338, TC375914, TC398343, TC377253, TC404426, TC439472, TC377944, TC381817, TC403588, TC394728, TC403803, TC418850, TC440526, TC436944, TC371341, TC381279, TC389661, CA726837, TC387386, TC383820, TC396657, TC380333, TC377064, TC397258, TC426838, CV775873, TC451511, TC431330, TC384373, TC416069, TC427210, TC422606, TC375313, TC399271, TC398862, DR739303, TC434442, TC413263, TC411471, TC431660, TC434570, TC378878, TC390792, TC394020, TC388950, TC374669, TC419222, TC410194, TC369371, TC448840, TC392875, TC409005, TC408228, TC384735, TC418728, TC405540, TC390569, TC391948, BQ294582, CA724903, TC401915, CV779166, DR739994, TC395506, TC395672, TC392358, TC450972, TC409599, TC419165, TC389092, TC376800, TC379853, TC420248, TC370315, TC403687, TC405440, TC460795, TC422789, TC370885, TC407232, TC440819, TC416492, TC393015, TC426980, TC377225, TC382858, TC404620, TC429865, TC434820, TC379711, TC379903, TC377308, TC406749, TC392272, TC401244, TC400114, TC413339, TC408907, TC375539, TC385013, TC380083, TC386963, TC412732, TC381191, TC369010, TC402080, TC373518, TC370350, TC383909, TC374940, CN010697, TC374392, TC378568, TC369983, CV761628, TC404420, TC383235, TC373489, TC369199, TC419747, TC407304, CA676115, TC402186, BE415178, TC429062, TC423354, TC409344, TC413043, BJ243383, TC400484, CK214224, TC368603, TC398040, TC374964, TC375234, TC385815, TC409187, TC429374, TC425690, TC375253, TC423182, TC401124, TC375873, TC444302, TC435224, CJ563078, TC394850, TC384344, TC392297, TC388520, TC443712, TC379422, TC378956, TC411191, TC426326, TC375431, TC424252, TC391995, TC415501, TC422841, TC387064, TC445649, CA498496, TC382777, TC435533, TC407349, TC397994, TC414243, TC403234, TC429437, TC430988, TC371738, TC413771, TC387621, TC398633, TC460689, TC380125, TC410126, TC369064, TC432154, TC420735, TC404371, TC402939, TC427405, TC449256, TC373615, TC383949, TC378409, TC416685, TC451342, TC372664, TC399986, TC389375, TC433812, TC370114, TC390319, TC406038, TC376874, TC424154, TC458807, TC369928, TC429871, TC419438, TC376490, TC384454, TC411287, TC386237, TC388751, AL820497, TC435909, TC431722, TC399471, TC408126, TC430096, TC399972, TC434396, TC421880, TC386414, TC389590, CJ854725, TC440636, TC403986, TC383763, TC369736, TC386279, CK197833, TC391785, TC377427, TC382786, TC409650, TC388158, TC418340, TC405773, TC382830, TC377653, DR739471, TC402534, TC382129, TC391840, TC390285, TC412989, TC387766, TC407340, TC398026, TC406584, CV065343, TC402440, TC396636, TC375146, TC454407, TC394661, TC389044, TC383176, TC371037, TC449463, TC388688, TC403328, TC408095, TC439939, TC375268, TC452503, TC388547, TC420877, TC429048, TC398978, TC388976, TC396365, TC379069, TC385526, TC423576, TC377635, TC402668, TC435799, TC438243, TC392887, TC370210, TC375918, TC417363, TC400592, TC385515, TC423880, TC451285, BG263159, TC458503, TC387194, TC391865, TC434689, TC441995, CB307332, CK211469, TC404842, TC381296, TC411817, TC436290, TC390489, TC421341, TC432504, TC383143 |
| [Microtubule](http://amigo.geneontology.org/cgi-bin/amigo/go.cgi?action=query&view=query&query=GO:0005874&search_constraint=terms) | TC415501, TC373637, TC379171, TC382045, TC371242 |
| [DNA-directed RNA polymerase complex](http://amigo.geneontology.org/cgi-bin/amigo/go.cgi?action=query&view=query&query=GO:0000428&search_constraint=terms) | TC390630, EB512907, TC397176 |
| [RNA polymerase complex](http://amigo.geneontology.org/cgi-bin/amigo/go.cgi?action=query&view=query&query=GO:0030880&search_constraint=terms) | TC390630, EB512907, TC397176 |
| [Nuclear DNA-directed RNA polymerase complex](http://amigo.geneontology.org/cgi-bin/amigo/go.cgi?action=query&view=query&query=GO:0055029&search_constraint=terms) | TC390630, EB512907, TC397176 |
| [Actin cytoskeleton](http://amigo.geneontology.org/cgi-bin/amigo/go.cgi?action=query&view=query&query=GO:0015629&search_constraint=terms) | TC381988, TC410126, TC393948, TC404843, CJ944525, TC410078, TC390630, TC459656, TC406807, CK199175, TC445166 |
| [Histone deacetylase complex](http://amigo.geneontology.org/cgi-bin/amigo/go.cgi?action=query&view=query&query=GO:0000118&search_constraint=terms) | CK211589, TC384735, TC381817, TC441343, TC413571, TC391128 |
| [Phragmoplast](http://amigo.geneontology.org/cgi-bin/amigo/go.cgi?action=query&view=query&query=GO:0009524&search_constraint=terms) | TC415501, TC373637, TC421871 |
| [Lamellipodium](http://amigo.geneontology.org/cgi-bin/amigo/go.cgi?action=query&view=query&query=GO:0030027&search_constraint=terms) | TC387410, TC382045 |
| [Heterochromatin](http://amigo.geneontology.org/cgi-bin/amigo/go.cgi?action=query&view=query&query=GO:0000792&search_constraint=terms) | CK211589, TC384735, TC381817, TC441343, TC413571, TC393960 |
| [Endoplasmic reticulum part](http://amigo.geneontology.org/cgi-bin/amigo/go.cgi?action=query&view=query&query=GO:0044432&search_constraint=terms) | TC409343, TC387191, TC390402, TC378790, TC405041, TC412569 |
| [Microtubule associated complex](http://amigo.geneontology.org/cgi-bin/amigo/go.cgi?action=query&view=query&query=GO:0005875&search_constraint=terms) | TC418845, TC407076, TC394796 |
| [Centriole](http://amigo.geneontology.org/cgi-bin/amigo/go.cgi?action=query&view=query&query=GO:0005814&search_constraint=terms) | TC425690, TC423110 |
| [Nuclear body](http://amigo.geneontology.org/cgi-bin/amigo/go.cgi?action=query&view=query&query=GO:0016604&search_constraint=terms) | TC369628, TC377190 |
| [Tubulin complex](http://amigo.geneontology.org/cgi-bin/amigo/go.cgi?action=query&view=query&query=GO:0045298&search_constraint=terms) | TC379171, TC371242 |
| [Vacuolar membrane](http://amigo.geneontology.org/cgi-bin/amigo/go.cgi?action=query&view=query&query=GO:0005774&search_constraint=terms) | TC405511, TC423354, TC403588, TC380882, TC423880 |
| [Nuclear envelope](http://amigo.geneontology.org/cgi-bin/amigo/go.cgi?action=query&view=query&query=GO:0005635&search_constraint=terms) | TC387135, TC393948, TC407978, TC390630, TC370114, TC409459, TC435808, TC438243, TC383909, TC449463 |
| [Nucleoplasm](http://amigo.geneontology.org/cgi-bin/amigo/go.cgi?action=query&view=query&query=GO:0005654&search_constraint=terms) | TC369628, TC374230, TC435799, TC441343, TC377190, TC397176, TC400362, CK211589, TC381817, TC417077, TC413571, TC460615, EB512907, TC384735, TC391128, TC418850, TC404413, TC409459, TC370114, TC393960, TC382045 |
| [Nucleoplasm part](http://amigo.geneontology.org/cgi-bin/amigo/go.cgi?action=query&view=query&query=GO:0044451&search_constraint=terms) | TC369628, TC374230, TC435799, TC441343, TC377190, TC397176, TC400362, CK211589, TC381817, TC417077, TC413571, TC460615, EB512907, TC384735, TC391128, TC418850, TC404413, TC409459, TC370114, TC393960, TC382045 |
| [Membrane raft](http://amigo.geneontology.org/cgi-bin/amigo/go.cgi?action=query&view=query&query=GO:0045121&search_constraint=terms) | TC425690, TC394118 |
| [Endocytic vesicle](http://amigo.geneontology.org/cgi-bin/amigo/go.cgi?action=query&view=query&query=GO:0030139&search_constraint=terms) | TC390630, TC393948, TC449724, TC433844, TC409650 |
| [Plant-type cell wall](http://amigo.geneontology.org/cgi-bin/amigo/go.cgi?action=query&view=query&query=GO:0009505&search_constraint=terms) | TC382830, TC435224, CK212850, CK215979 |
| [Phagocytic vesicle](http://amigo.geneontology.org/cgi-bin/amigo/go.cgi?action=query&view=query&query=GO:0045335&search_constraint=terms) | TC390630, TC393948, TC449724, TC433844 |
| [Vacuole](http://amigo.geneontology.org/cgi-bin/amigo/go.cgi?action=query&view=query&query=GO:0005773&search_constraint=terms) | TC372530, TC442623, TC405511, TC388819, TC373613, TC432185, TC380882, TC423880, TC423354, TC403588 |
| [Cell projection part](http://amigo.geneontology.org/cgi-bin/amigo/go.cgi?action=query&view=query&query=GO:0044463&search_constraint=terms) | TC461622, TC380882, TC371242, TC380433, CK211589, TC403588, TC393970, TC413571, TC382045 |
| [Nuclear microtubule](http://amigo.geneontology.org/cgi-bin/amigo/go.cgi?action=query&view=query&query=GO:0005880&search_constraint=terms) | TC379171, TC371242 |
| [Cellular bud neck](http://amigo.geneontology.org/cgi-bin/amigo/go.cgi?action=query&view=query&query=GO:0005935&search_constraint=terms) | TC393970, TC382045 |
| [NADH dehydrogenase complex](http://amigo.geneontology.org/cgi-bin/amigo/go.cgi?action=query&view=query&query=GO:0030964&search_constraint=terms) | BJ282766, TC397291, TC377190 |
| [Golgi apparatus part](http://amigo.geneontology.org/cgi-bin/amigo/go.cgi?action=query&view=query&query=GO:0044431&search_constraint=terms) | TC376490, TC411784, TC398343, TC369736, TC385515, TC409650, TC378790 |
| [Cell cortex](http://amigo.geneontology.org/cgi-bin/amigo/go.cgi?action=query&view=query&query=GO:0005938&search_constraint=terms) | TC373637, TC412150 |
| [Spindle](http://amigo.geneontology.org/cgi-bin/amigo/go.cgi?action=query&view=query&query=GO:0005819&search_constraint=terms) | TC377190, TC394796, TC423110, TC371242, TC373637, TC425690, TC379171, TC393970, TC382045 |
| [Cellular bud](http://amigo.geneontology.org/cgi-bin/amigo/go.cgi?action=query&view=query&query=GO:0005933&search_constraint=terms) | TC393970, TC382045 |
| [Yeast-form cell wall](http://amigo.geneontology.org/cgi-bin/amigo/go.cgi?action=query&view=query&query=GO:0030445&search_constraint=terms) | TC438243, TC407076, TC407978 |
| [Cell fraction](http://amigo.geneontology.org/cgi-bin/amigo/go.cgi?action=query&view=query&query=GO:0000267&search_constraint=terms) | TC381988, TC405511, TC387410, TC435799, TC370885, TC461622, TC393820, TC404843, TC400056, TC423354, TC425957, TC403588, TC449724, TC445166, TC388718, TC398514, TC407076, TC387710, TC380882, TC423880, TC410078, TC425690, TC457126, TC404606 |
| [Vacuolar part](http://amigo.geneontology.org/cgi-bin/amigo/go.cgi?action=query&view=query&query=GO:0044437&search_constraint=terms) | TC405511, TC423354, TC403588, TC380882, TC423880 |
| [Plant-type vacuole](http://amigo.geneontology.org/cgi-bin/amigo/go.cgi?action=query&view=query&query=GO:0000325&search_constraint=terms) | TC442623, TC388819 |
| [Endosome](http://amigo.geneontology.org/cgi-bin/amigo/go.cgi?action=query&view=query&query=GO:0005768&search_constraint=terms) | TC372530, TC431306, TC385659, TC370178, TC373848 |
| [Golgi membrane](http://amigo.geneontology.org/cgi-bin/amigo/go.cgi?action=query&view=query&query=GO:0000139&search_constraint=terms) | TC385515, TC409650, TC378790 |
| [Extracellular matrix part](http://amigo.geneontology.org/cgi-bin/amigo/go.cgi?action=query&view=query&query=GO:0044420&search_constraint=terms) | TC407076, TC371242 |
| [Receptor complex](http://amigo.geneontology.org/cgi-bin/amigo/go.cgi?action=query&view=query&query=GO:0043235&search_constraint=terms) | TC438243, TC390402, TC376420, TC407076 |
| [Spindle pole body](http://amigo.geneontology.org/cgi-bin/amigo/go.cgi?action=query&view=query&query=GO:0005816&search_constraint=terms) | TC425690, TC379171, TC393970, TC382045, TC371242 |
| [Microtubule organizing center part](http://amigo.geneontology.org/cgi-bin/amigo/go.cgi?action=query&view=query&query=GO:0044450&search_constraint=terms) | TC425690, TC393970, TC423110 |
| [Site of polarized growth](http://amigo.geneontology.org/cgi-bin/amigo/go.cgi?action=query&view=query&query=GO:0030427&search_constraint=terms) | TC438243, TC393970, TC407978, TC382045 |
| [Spindle pole](http://amigo.geneontology.org/cgi-bin/amigo/go.cgi?action=query&view=query&query=GO:0000922&search_constraint=terms) | TC425690, TC379171, TC393970, TC382045, TC371242 |
| [Cytoskeleton](http://amigo.geneontology.org/cgi-bin/amigo/go.cgi?action=query&view=query&query=GO:0005856&search_constraint=terms) | TC381988, TC410126, TC393948, TC377190, TC394796, TC423110, TC404843, CJ944525, TC373637, CK211589, TC390630, TC370044, TC459656, TC445166, TC413571, TC418845, TC407076, TC371242, TC415501, TC410078, TC425690, TC423804, TC406807, TC379171, TC393970, CK199175, TC382045 |
| [Proteasome core complex](http://amigo.geneontology.org/cgi-bin/amigo/go.cgi?action=query&view=query&query=GO:0005839&search_constraint=terms) | TC397562, TC420420 |
| [Cytoskeletal part](http://amigo.geneontology.org/cgi-bin/amigo/go.cgi?action=query&view=query&query=GO:0044430&search_constraint=terms) | TC410126, TC393948, TC377190, TC394796, TC423110, CJ944525, TC373637, CK211589, TC390630, TC459656, TC413571, TC418845, TC407076, TC371242, TC415501, TC425690, TC423804, TC406807, TC379171, CK199175, TC393970, TC382045 |
| [Respiratory chain complex I](http://amigo.geneontology.org/cgi-bin/amigo/go.cgi?action=query&view=query&query=GO:0045271&search_constraint=terms) | BJ282766, TC397291 |
| [Chloroplast thylakoid lumen](http://amigo.geneontology.org/cgi-bin/amigo/go.cgi?action=query&view=query&query=GO:0009543&search_constraint=terms) | TC387007, TC406516 |
| [Thylakoid lumen](http://amigo.geneontology.org/cgi-bin/amigo/go.cgi?action=query&view=query&query=GO:0031977&search_constraint=terms) | TC387007, TC406516 |
| [Plastid thylakoid lumen](http://amigo.geneontology.org/cgi-bin/amigo/go.cgi?action=query&view=query&query=GO:0031978&search_constraint=terms) | TC387007, TC406516 |
| [Golgi-associated vesicle](http://amigo.geneontology.org/cgi-bin/amigo/go.cgi?action=query&view=query&query=GO:0005798&search_constraint=terms) | TC385515, TC409650 |
| [Proteinaceous extracellular matrix](http://amigo.geneontology.org/cgi-bin/amigo/go.cgi?action=query&view=query&query=GO:0005578&search_constraint=terms) | TC407076, TC371242 |
| [Microtubule organizing center](http://amigo.geneontology.org/cgi-bin/amigo/go.cgi?action=query&view=query&query=GO:0005815&search_constraint=terms) | TC373637, TC425690, TC379171, TC393970, TC423110, TC382045, TC371242 |
| [Immunoglobulin complex](http://amigo.geneontology.org/cgi-bin/amigo/go.cgi?action=query&view=query&query=GO:0019814&search_constraint=terms) | TC376420, TC407076 |
| [B cell receptor complex](http://amigo.geneontology.org/cgi-bin/amigo/go.cgi?action=query&view=query&query=GO:0019815&search_constraint=terms) | TC376420, TC407076 |
| [T cell receptor complex](http://amigo.geneontology.org/cgi-bin/amigo/go.cgi?action=query&view=query&query=GO:0042101&search_constraint=terms) | TC376420, TC407076 |
| [Small ribosomal subunit](http://amigo.geneontology.org/cgi-bin/amigo/go.cgi?action=query&view=query&query=GO:0015935&search_constraint=terms) | TC381988, TC403580, TC421914, TC456619, TC378271, TC407978, TC441343, TC371970, TC404843, TC459656, TC383909, TC449463, TC451511, TC445166, TC403328, CA598430, TC384735, TC368603, TC372664, TC409599, TC375431, TC410078, TC409208, TC396451, TC406807, TC376874, TC418091 |
| [Microtubule cytoskeleton](http://amigo.geneontology.org/cgi-bin/amigo/go.cgi?action=query&view=query&query=GO:0015630&search_constraint=terms) | TC418845, TC407076, TC377190, TC394796, TC423110, TC371242, TC415501, TC373637, CK211589, TC370044, TC425690, TC379171, TC393970, TC413571, TC382045 |
| [Lipid particle](http://amigo.geneontology.org/cgi-bin/amigo/go.cgi?action=query&view=query&query=GO:0005811&search_constraint=terms) | TC381988, TC372530, TC403580, TC405511, TC376420, TC407076, TC407978, TC404843, TC380882, TC423880, TC378790, TC410078, TC423354, TC403588, TC445166 |
| [Mitochondrial inner membrane](http://amigo.geneontology.org/cgi-bin/amigo/go.cgi?action=query&view=query&query=GO:0005743&search_constraint=terms) | TC411471, TC388049, TC393820, TC404413, TC400362, TC386396, TC432154, TC449724, TC393960, TC372677, TC387621, TC398514 |
| [Nuclear lumen](http://amigo.geneontology.org/cgi-bin/amigo/go.cgi?action=query&view=query&query=GO:0031981&search_constraint=terms) | TC369628, TC374230, TC435799, TC441343, TC390630, TC377061, TC449724, TC417077, TC413571, TC460615, EB512907, TC387710, TC394118, TC409599, TC391128, TC417341, TC423804, TC373259, TC370315, TC387410, TC405475, TC377190, TC397176, TC393820, TC391613, TC373637, TC380433, TC400362, CK211589, TC381817, TC398606, TC384735, TC372664, TC404413, TC418850, TC456230, TC409459, TC373994, TC370114, TC393960, TC404606, TC382045 |
| [Endoplasmic reticulum](http://amigo.geneontology.org/cgi-bin/amigo/go.cgi?action=query&view=query&query=GO:0005783&search_constraint=terms) | TC406809, TC378790, TC412569, TC372654, TC369348, TC405041, TC389842, TC409343, TC385009, TC375268, TC376248, TC387191, TC396487, TC390402, TC406106, TC427981, TC374485, TC412317, TC381619, TC457126, TC382045, TC395298 |
| [Mitochondrial part](http://amigo.geneontology.org/cgi-bin/amigo/go.cgi?action=query&view=query&query=GO:0044429&search_constraint=terms) | TC411471, TC374230, TC373613, TC371970, TC393820, TC400362, TC432154, TC386396, TC397291, TC449724, TC407340, TC372677, TC375918, TC398514, TC388049, TC407076, TC368603, TC432185, TC391128, TC404413, TC373002, TC398379, TC396451, BJ282766, TC393960, TC387621, TC382045 |
| [Intracellular organelle lumen](http://amigo.geneontology.org/cgi-bin/amigo/go.cgi?action=query&view=query&query=GO:0070013&search_constraint=terms) | TC369628, TC374230, TC435799, TC441343, TC371970, TC390630, TC377061, TC449724, TC417077, TC413571, TC407340, TC375918, TC460615, EB512907, TC368603, TC387710, TC391128, TC394118, TC409599, TC417341, TC423804, TC373259, TC370315, TC387410, TC405475, TC377190, TC397176, TC393820, TC391613, TC373637, TC380433, TC400362, CK211589, TC381817, TC398606, TC384735, TC388049, TC372664, TC404413, TC418850, TC456230, TC409459, TC373994, TC370114, TC396451, TC393960, TC404606, TC382045 |
| [Organelle lumen](http://amigo.geneontology.org/cgi-bin/amigo/go.cgi?action=query&view=query&query=GO:0043233&search_constraint=terms) | TC369628, TC374230, TC435799, TC441343, TC371970, TC390630, TC377061, TC449724, TC417077, TC413571, TC407340, TC375918, TC460615, EB512907, TC368603, TC387710, TC391128, TC394118, TC409599, TC417341, TC423804, TC373259, TC370315, TC387410, TC405475, TC377190, TC397176, TC393820, TC391613, TC373637, TC380433, TC400362, CK211589, TC381817, TC398606, TC384735, TC388049, TC372664, TC404413, TC418850, TC456230, TC409459, TC373994, TC370114, TC396451, TC393960, TC404606, TC382045 |
| [Golgi apparatus](http://amigo.geneontology.org/cgi-bin/amigo/go.cgi?action=query&view=query&query=GO:0005794&search_constraint=terms) | TC372530, TC411784, TC385515, TC448051, TC409650, TC378790, TC376490, TC398343, TC369736 |
| [Nucleolus](http://amigo.geneontology.org/cgi-bin/amigo/go.cgi?action=query&view=query&query=GO:0005730&search_constraint=terms) | TC369628, TC387410, TC441343, TC405475, TC393820, TC391613, TC380433, TC373637, TC390630, TC449724, TC398606, TC384735, TC372664, TC387710, TC394118, TC409599, TC417341, TC456230, TC423804, TC373994, TC373259, TC370315, TC404606, TC382045 |
| [Cytosolic large ribosomal subunit](http://amigo.geneontology.org/cgi-bin/amigo/go.cgi?action=query&view=query&query=GO:0022625&search_constraint=terms) | TC416442, TC416906, TC402545, TC431201, TC435799, TC418414, TC393820, TC444546, TC380433, CK211589, TC370044, TC403264, TC417769, TC449724, TC417077, TC413571, TC417388, TC388718, TC440066, TC387861, TC398862, TC370114, TC373994, TC425690, TC409459, TC450285, TC398970, TC457126 |
| [Organelle inner membrane](http://amigo.geneontology.org/cgi-bin/amigo/go.cgi?action=query&view=query&query=GO:0019866&search_constraint=terms) | TC411471, TC388049, TC393820, TC404413, TC400362, TC386396, TC432154, TC449724, TC393960, TC372677, TC387621, TC398514 |
| [Centrosome](http://amigo.geneontology.org/cgi-bin/amigo/go.cgi?action=query&view=query&query=GO:0005813&search_constraint=terms) | TC425690, TC423110 |
| [Mitochondrial membrane](http://amigo.geneontology.org/cgi-bin/amigo/go.cgi?action=query&view=query&query=GO:0031966&search_constraint=terms) | TC411471, TC388049, TC393820, TC404413, TC373002, TC400362, TC432154, TC386396, BJ282766, TC449724, TC397291, TC393960, TC372677, TC387621, TC398514 |
| [Cytosolic part](http://amigo.geneontology.org/cgi-bin/amigo/go.cgi?action=query&view=query&query=GO:0044445&search_constraint=terms) | TC403580, TC456619, TC416906, TC435799, TC407978, TC441343, TC444546, TC423354, TC459656, TC403264, TC417769, TC449724, TC417077, TC451511, TC445166, TC413571, TC417388, TC440066, CA598430, TC388751, TC387861, TC398121, TC398862, TC409599, TC380882, TC423880, TC409208, TC425690, TC418091, TC416442, TC381988, TC421914, TC405511, TC402545, TC378271, TC431201, TC418414, TC404843, TC393820, TC380433, CK211589, TC370044, TC403588, TC383909, TC449463, TC371037, TC388718, TC379422, TC384735, TC372664, TC375431, TC410078, TC370114, TC373994, TC409459, TC406807, TC450285, TC398970, TC457126, TC376874, TC372845 |
| [Extracellular matrix](http://amigo.geneontology.org/cgi-bin/amigo/go.cgi?action=query&view=query&query=GO:0031012&search_constraint=terms) | TC407076, TC371242 |
| [Cytosolic small Ribosomal subunit](http://amigo.geneontology.org/cgi-bin/amigo/go.cgi?action=query&view=query&query=GO:0022627&search_constraint=terms) | TC381988, TC403580, TC421914, TC456619, TC378271, TC407978, TC441343, TC404843, TC459656, TC383909, TC449463, TC451511, TC445166, CA598430, TC384735, TC372664, TC409599, TC375431, TC410078, TC409208, TC406807, TC376874, TC418091 |
| [Membrane-enclosed lumen](http://amigo.geneontology.org/cgi-bin/amigo/go.cgi?action=query&view=query&query=GO:0031974&search_constraint=terms) | TC369628, TC374230, TC435799, TC441343, TC371970, TC390630, TC377061, TC449724, TC417077, TC413571, TC407340, TC375918, TC460615, EB512907, TC368603, TC387710, TC391128, TC394118, TC409599, TC417341, TC423804, TC373259, TC370315, TC387410, TC405475, TC377190, TC397176, TC393820, TC391613, TC373637, TC380433, TC400362, CK211589, TC381817, TC398606, TC384735, TC388049, TC372664, TC404413, TC418850, TC456230, TC409459, TC373994, TC370114, TC396451, TC393960, TC404606, TC382045 |
| [Mitochondrial envelope](http://amigo.geneontology.org/cgi-bin/amigo/go.cgi?action=query&view=query&query=GO:0005740&search_constraint=terms) | TC411471, TC373613, TC393820, TC400362, TC432154, TC386396, TC397291, TC449724, TC372677, TC398514, TC388049, TC407076, TC432185, TC404413, TC373002, TC398379, BJ282766, TC393960, TC387621 |
| [Large ribosomal subunit](http://amigo.geneontology.org/cgi-bin/amigo/go.cgi?action=query&view=query&query=GO:0015934&search_constraint=terms) | TC416442, TC416906, TC402545, TC431201, TC435799, TC391641, TC418414, TC393820, TC444546, TC380433, CK211589, TC370044, TC403264, TC417769, TC449724, TC417077, TC413571, TC417388, TC388718, TC440066, TC387861, TC389168, TC391785, TC398862, TC370114, TC373994, TC425690, TC409459, TC450285, TC398970, TC457126 |
| [Organelle envelope](http://amigo.geneontology.org/cgi-bin/amigo/go.cgi?action=query&view=query&query=GO:0031967&search_constraint=terms) | TC411471, TC373613, TC393948, TC407978, TC393820, TC400362, TC432154, TC386396, TC390630, TC435808, TC438243, TC383909, TC449463, TC397291, TC449724, TC372677, TC398514, TC387135, TC388049, TC407076, TC432185, TC404413, TC373002, TC398379, TC409459, TC370114, BJ282766, TC393960, TC387621 |
| [Envelope](http://amigo.geneontology.org/cgi-bin/amigo/go.cgi?action=query&view=query&query=GO:0031975&search_constraint=terms) | TC411471, TC373613, TC393948, TC407978, TC393820, TC400362, TC432154, TC386396, TC390630, TC435808, TC438243, TC383909, TC449463, TC397291, TC449724, TC372677, TC398514, TC387135, TC388049, TC407076, TC432185, TC404413, TC373002, TC398379, TC409459, TC370114, BJ282766, TC393960, TC387621 |
| [Respiratory chain](http://amigo.geneontology.org/cgi-bin/amigo/go.cgi?action=query&view=query&query=GO:0070469&search_constraint=terms) | BJ282766, TC397291 |
| [Protein complex](http://amigo.geneontology.org/cgi-bin/amigo/go.cgi?action=query&view=query&query=GO:0043234&search_constraint=terms) | TC394796, TC425957, CV775873, TC420420, TC374240, TC398052, TC425841, TC398121, TC387579, TC394459, TC371242, TC384738, TC379171, TC388410, BJ282766, TC387621, TC458562, TC411471, TC410126, TC397176, CJ944525, TC380433, TC386961, TC398606, TC397562, TC384735, TC387344, TC407076, TC404413, TC398714, TC370114, CJ550278, TC398304, TC433957, TC393100, CK210754, TC369928, TC412569, TC397291, TC386237, TC394916, EB512907, TC409343, TC388751, TC390994, TC394820, TC378274, TC391128, TC423804, TC370315, TC395303, TC400755, TC405511, TC395723, TC376420, TC458987, TC377190, TC423110, TC379536, TC398730, TC398514, TC390135, TC387135, TC377225, TC409650, TC380662, TC398379, TC388649, TC406807, TC393960, TC372845, TC382045, CA646741, TC389162, TC441343, TC400260, TC416438, TC390630, TC369726, TC377061, TC417077, TC407340, TC386313, TC390402, CA606693, CK211707, TC386963, CK199175, TC373637, TC396751, CK211589, TC383909, TC371037, CA615187, TC449463, TC374164, TC383701, TC418845, TC374392, TC388547, TC405784, TC372175, TC407572, TC399919, TC396365, TC419747, TC409459, TC374230, TC375834, TC393948, TC435799, TC459656, TC382928, TC423354, TC441241, TC438243, TC413571, TC460615, TC417363, TC403929, TC385515, TC380882, TC423880, TC422425, TC371387, TC439472, TC400362, TC403588, TC369092, TC381817, TC379422, TC411191, TC418850, TC415501, TC401758, TC431306, TC389661 |
| [Mitochondrial membrane part](http://amigo.geneontology.org/cgi-bin/amigo/go.cgi?action=query&view=query&query=GO:0044455&search_constraint=terms) | TC411471, TC387621 |
| [Membrane fraction](http://amigo.geneontology.org/cgi-bin/amigo/go.cgi?action=query&view=query&query=GO:0005624&search_constraint=terms) | TC387410, TC435799, TC387710, TC393820, TC425690, TC449724, TC457126, TC388718, TC404606 |
| [Insoluble fraction](http://amigo.geneontology.org/cgi-bin/amigo/go.cgi?action=query&view=query&query=GO:0005626&search_constraint=terms) | TC387410, TC435799, TC387710, TC393820, TC425690, TC449724, TC457126, TC388718, TC404606 |
| [Ribosomal subunit](http://amigo.geneontology.org/cgi-bin/amigo/go.cgi?action=query&view=query&query=GO:0033279&search_constraint=terms) | TC403580, TC456619, TC416906, TC435799, TC407978, TC441343, TC371970, TC444546, TC459656, TC403264, TC417769, TC449724, TC417077, TC451511, TC445166, TC413571, TC417388, TC440066, CA598430, TC387861, TC389168, TC368603, TC398862, TC409599, TC409208, TC425690, TC418091, TC416442, TC381988, TC421914, TC402545, TC378271, TC431201, TC391641, TC418414, TC404843, TC393820, TC380433, CK211589, TC370044, TC383909, TC449463, TC388718, TC403328, TC384735, TC372664, TC391785, TC375431, TC410078, TC370114, TC373994, TC409459, TC396451, TC406807, TC450285, TC398970, TC457126, TC376874 |
| [Photosystem](http://amigo.geneontology.org/cgi-bin/amigo/go.cgi?action=query&view=query&query=GO:0009521&search_constraint=terms) | TC439472, TC400260 |
| [Ribonucleoprotein complex](http://amigo.geneontology.org/cgi-bin/amigo/go.cgi?action=query&view=query&query=GO:0030529&search_constraint=terms) | TC369628, TC416906, TC379942, TC407978, TC441343, TC388566, TC444546, TC373429, TC417077, TC451511, TC445166, TC417388, TC440066, TC387861, TC389168, TC398862, TC417341, TC415685, TC409208, TC376220, TC416442, TC381988, TC421914, TC387410, TC402545, TC378271, TC431201, TC418414, TC380433, CK211589, TC370044, TC435281, TC383909, TC449463, TC388718, TC403328, TC384735, TC405540, TC372664, TC391948, TC410078, TC456230, TC409459, TC370114, TC373994, TC381619, TC398970, TC457126, TC376874, TC403580, TC456619, TC435799, TC371970, TC403264, TC459656, TC417769, TC449724, TC413571, CA598430, TC413027, TC368603, TC387710, TC409599, TC399471, TC425690, TC373259, TC423182, TC410066, TC418091, TC399352, TC408312, TC391641, TC443814, TC393820, TC404843, TC385233, TC398730, TC382737, TC387135, TC400108, TC391785, TC418850, TC375431, TC390489, TC396451, TC406807, TC450285, TC404606, TC382045, TC395298 |
| [Cytosolic ribosome](http://amigo.geneontology.org/cgi-bin/amigo/go.cgi?action=query&view=query&query=GO:0022626&search_constraint=terms) | TC403580, TC456619, TC416906, TC435799, TC407978, TC441343, TC444546, TC459656, TC403264, TC417769, TC449724, TC417077, TC451511, TC445166, TC413571, TC417388, TC440066, CA598430, TC387861, TC398862, TC409599, TC409208, TC425690, TC423182, TC418091, TC416442, TC381988, TC421914, TC402545, TC378271, TC431201, TC418414, TC404843, TC393820, TC380433, CK211589, TC370044, TC383909, TC449463, TC388718, TC384735, TC372664, TC375431, TC410078, TC373994, TC370114, TC409459, TC406807, TC381619, TC450285, TC398970, TC457126, TC376874, TC395298 |
| [Proteasome complex](http://amigo.geneontology.org/cgi-bin/amigo/go.cgi?action=query&view=query&query=GO:0000502&search_constraint=terms) | TC397562, TC420420 |
| [Ribosome](http://amigo.geneontology.org/cgi-bin/amigo/go.cgi?action=query&view=query&query=GO:0005840&search_constraint=terms) | TC416906, TC379942, TC407978, TC441343, TC444546, TC417077, TC451511, TC445166, TC417388, TC440066, TC387861, TC389168, TC398862, TC415685, TC409208, TC416442, TC381988, TC421914, TC402545, TC378271, TC431201, TC418414, TC380433, CK211589, TC370044, TC435281, TC383909, TC449463, TC388718, TC403328, TC384735, TC372664, TC391948, TC410078, TC370114, TC373994, TC409459, TC381619, TC398970, TC457126, TC376874, TC403580, TC456619, TC435799, TC371970, TC403264, TC459656, TC417769, TC449724, TC413571, CA598430, TC413027, TC368603, TC409599, TC399471, TC425690, TC373259, TC423182, TC410066, TC418091, TC408312, TC391641, TC443814, TC393820, TC404843, TC382737, TC400108, TC391785, TC418850, TC375431, TC390489, TC396451, TC406807, TC450285, TC382045, TC395298 |
| [Plastoglobule](http://amigo.geneontology.org/cgi-bin/amigo/go.cgi?action=query&view=query&query=GO:0010287&search_constraint=terms) | TC373001, TC439472, TC432185 |
| [Cytosol](http://amigo.geneontology.org/cgi-bin/amigo/go.cgi?action=query&view=query&query=GO:0005829&search_constraint=terms) | TC389162, TC373613, TC416906, TC407978, TC441343, TC388566, TC378790, TC444546, TC425957, TC377061, TC417077, TC451511, TC445166, TC417388, TC440066, TC417106, TC387861, TC457112, TC398121, TC398862, TC376351, TC409208, TC454407, TC416442, TC381988, TC421914, TC402545, TC378271, TC431201, TC418414, TC380433, CK211589, TC373702, TC370044, TC383909, TC449463, TC371037, TC427405, TC388718, TC397562, TC384735, TC405540, TC372664, TC407076, TC410078, TC409459, TC370114, TC373994, TC393970, TC398970, TC457126, TC376874, TC386646, TC403580, GH732878, TC456619, TC435799, TC369928, TC403264, TC459656, TC423354, TC417769, TC438243, TC455515, TC376490, TC449724, TC413571, CA598430, TC388751, TC394820, TC409599, TC391128, TC380882, TC423880, TC425690, TC423804, TC418091, TC370315, TC372530, TC405511, TC376420, TC393820, TC404843, TC461622, TC403588, TC398514, TC379422, TC387135, TC432185, TC375431, TC440526, TC406807, TC450285, TC393960, TC382045, TC372845 |
| [Chloroplast](http://amigo.geneontology.org/cgi-bin/amigo/go.cgi?action=query&view=query&query=GO:0009507&search_constraint=terms) | TC439472, TC369899, TC407978, TC400260, TC406516, TC399245, TC373001, TC438243, TC418716, TC417106, TC452503, TC432185, TC387007, TC375813, TC402668, TC377438 |
| [Plastid stroma](http://amigo.geneontology.org/cgi-bin/amigo/go.cgi?action=query&view=query&query=GO:0009532&search_constraint=terms) | TC373001, TC439472, TC438243, TC407978, TC432185 |
| [Chloroplast thylakoid](http://amigo.geneontology.org/cgi-bin/amigo/go.cgi?action=query&view=query&query=GO:0009534&search_constraint=terms) | TC439472, TC387007, TC452503, TC418716, TC400260, TC406516 |
| [Chloroplast thylakoid membrane](http://amigo.geneontology.org/cgi-bin/amigo/go.cgi?action=query&view=query&query=GO:0009535&search_constraint=terms) | TC439472, TC452503, TC418716, TC400260 |
| [Chloroplast stroma](http://amigo.geneontology.org/cgi-bin/amigo/go.cgi?action=query&view=query&query=GO:0009570&search_constraint=terms) | TC373001, TC439472, TC438243, TC407978, TC432185 |
| [Thylakoid](http://amigo.geneontology.org/cgi-bin/amigo/go.cgi?action=query&view=query&query=GO:0009579&search_constraint=terms) | TC439472, TC387007, TC452503, TC418716, TC400260, TC406516 |
| [Plastid thylakoid](http://amigo.geneontology.org/cgi-bin/amigo/go.cgi?action=query&view=query&query=GO:0031976&search_constraint=terms) | TC439472, TC387007, TC452503, TC418716, TC400260, TC406516 |
| [Organelle subcompartment](http://amigo.geneontology.org/cgi-bin/amigo/go.cgi?action=query&view=query&query=GO:0031984&search_constraint=terms) | TC439472, TC387007, TC452503, TC418716, TC400260, TC369736, TC406516 |
| [Photosynthetic membrane](http://amigo.geneontology.org/cgi-bin/amigo/go.cgi?action=query&view=query&query=GO:0034357&search_constraint=terms) | TC439472, TC452503, TC418716, TC400260 |
| [Thylakoid membrane](http://amigo.geneontology.org/cgi-bin/amigo/go.cgi?action=query&view=query&query=GO:0042651&search_constraint=terms) | TC439472, TC452503, TC418716, TC400260 |
| [Chloroplast part](http://amigo.geneontology.org/cgi-bin/amigo/go.cgi?action=query&view=query&query=GO:0044434&search_constraint=terms) | TC439472, TC452503, TC407978, TC400260, TC432185, TC406516, TC373001, TC387007, TC438243, TC418716 |
| [Plastid part](http://amigo.geneontology.org/cgi-bin/amigo/go.cgi?action=query&view=query&query=GO:0044435&search_constraint=terms) | TC439472, TC452503, TC407978, TC400260, TC432185, TC406516, TC373001, TC387007, TC438243, TC418716 |
| [Thylakoid part](http://amigo.geneontology.org/cgi-bin/amigo/go.cgi?action=query&view=query&query=GO:0044436&search_constraint=terms) | TC439472, TC387007, TC452503, TC418716, TC400260, TC406516 |
| [Plastid thylakoid membrane](http://amigo.geneontology.org/cgi-bin/amigo/go.cgi?action=query&view=query&query=GO:0055035&search_constraint=terms) | TC439472, TC452503, TC418716, TC400260 |
