# Supplementary material for: Global Analysis of Differentially Expressed Genes and Proteins in the Wheat Callus Infected by Agrobacterium tumefaciens
Source: PLoS One. 2013 Nov 20;8(11):e79390. doi: 10.1371/journal.pone.0079390 (PMC3835833; doi:10.1371/journal.pone.0079390)
Supplement: File S7 — Gene Ontology analysis information for molecular function. (DOC) [file pone.0079390.s007.doc]

| **Gene ontology term**  **File S7 Gene ontology analysis information for molecular function** | **Genes annotated to the term** |
| --- | --- |
| [DNA binding](http://amigo.geneontology.org/cgi-bin/amigo/go.cgi?action=query&view=query&query=GO:0003677&search_constraint=terms) | TC461921, TC410352, TC386040, TC390393, TC416529, TC397258, TC389190, CV775873, TC396230, TC460760, TC427210, TC416069, BQ838511, TC398052, CA710880, TC400388, TC433162, TC394459, TC416154, TC384738, TC423252, TC397660, TC389363, TC388410, CK203550, TC416493, TC458562, TC406264, TC380063, TC398538, TC397176, TC410063, TC378054, TC448471, TC395453, TC410194, TC392875, TC392329, TC409843, TC435546, TC390569, TC405540, CA682223, TC419057, TC403872, TC425847, CJ727624, TC398714, BQ294582, CA614761, CJ550278, TC413427, TC411128, TC401915, TC397444, TC398304, TC425878, TC393100, TC433957, TC417012, TC423239, CA632212, DR739994, TC459193, TC394916, TC394206, TC394820, TC398731, TC406236, TC418073, TC378274, TC391130, TC417308, CK217367, TC445767, TC389092, TC446038, TC413460, TC379853, BQ609416, CK214702, TC449504, TC395723, TC405440, TC403968, CK213497, CJ792862, TC389718, TC404052, TC377225, TC381943, TC412520, TC432001, TC413392, BQ607161, TC402308, TC434820, TC379711, TC400330, CA595837, TC379903, TC423265, TC392272, TC377061, TC401244, CA730421, TC417077, TC446465, TC386313, CV759879, CK208222, TC408907, CA606693, TC391438, CK211707, TC386963, TC412732, TC405695, CA700201, TC392778, TC396751, TC397312, TC405030, CK211589, TC388403, TC376527, CA615187, TC416695, TC419584, TC385780, TC406469, TC405784, TC399919, TC407572, TC372175, TC410690, TC406708, TC419747, TC409459, TC410074, TC402186, TC405356, CV763657, TC458205, TC402603, TC441241, TC413571, TC413043, CA729339, CK214224, TC408299, BQ239045, CA709177, TC376263, TC416169, TC425690, TC381218, TC408229, TC422425, CA613620, TC398805, TC369092, CV781430, TC388520, TC411191, TC399342, TC434831, TC426326, TC381462, CD882425, TC373914, TC392263, TC392303, CA611770, TC435533, TC374240, CA720842, TC425841, CF554444, TC424204, TC387579, TC406870, TC388914, TC393814, TC387621, TC398633, TC394716, TC446235, TC402545, TC380943, TC393830, TC380433, TC403885, TC420735, TC404371, TC373615, TC384219, TC386961, TC416685, TC387344, TC412483, TC389090, TC389375, TC382655, TC370114, CK200433, TC406038, TC395351, TC418032, CK210754, TC410147, TC384454, TC380329, TC411287, TC386237, TC393904, TC390994, TC391143, TC381463, TC403157, TC386414, TC395303, TC400755, TC440636, TC403986, TC406193, TC405615, TC458987, TC377190, TC443814, TC404843, TC431879, TC422348, CD876572, TC385365, TC401210, DR737360, TC379357, TC390135, TC397885, TC386279, TC411684, TC411480, TC380662, TC426358, TC389162, DR739471, TC402534, TC443387, TC415083, TC386535, GH729256, TC406584, TC386519, CA605200, TC402440, TC417992, TC391411, TC394307, TC388665, TC375146, TC389589, TC452762, TC386344, TC381963, TC383176, CD878039, TC374879, TC386422, TC374164, TC388688, TC388547, TC414606, TC388822, TC377496, TC368549, TC388976, TC396365, TC429771, TC423576, TC386639, TC397500, TC416658, TC393561, TC393692, TC391671, TC393554, TC407614, TC382928, TC379436, TC417363, TC403573, TC403929, TC413700, TC449043, TC379635, TC440321, TC433589, TC377410, TC389816, TC392323, TC413066, CK211469, CB307332, TC404842, TC397729, TC403803, TC396895, TC418850, TC382342, TC393106, TC401758, TC432504, TC397676, TC396460, TC417067, TC389661, TC428944 |
| [Transferase activity](http://amigo.geneontology.org/cgi-bin/amigo/go.cgi?action=query&view=query&query=GO:0016740&search_constraint=terms) | TC410352, TC427986, TC369899, TC378846, TC394796, TC440499, TC391946, TC394965, TC384373, TC374240, TC417106, TC375313, CA710880, TC395966, TC398121, TC439324, TC403234, TC439423, TC413854, TC423252, TC397660, TC389363, TC388914, TC434442, BJ279521, TC413263, TC380590, TC368548, TC446235, TC378072, TC397176, TC376202, TC420735, TC456784, TC395453, TC409843, TC390569, TC416685, TC384688, TC407076, TC403872, TC389090, TC370347, TC389375, TC382655, CK200433, TC370114, TC406038, TC390319, CJ550278, TC395351, TC372563, TC401915, TC369928, TC405652, DR739994, TC384454, TC411908, EB512907, TC397793, TC388751, CK217367, TC379853, TC392074, TC398536, CK199846, TC370315, CJ854725, TC395723, TC405440, TC376420, TC443814, TC436347, TC384553, TC430544, TC394087, TC370633, TC369736, TC398514, DR737360, TC392033, CK197833, TC376758, BQ607161, TC406807, CD892838, TC419727, TC395090, TC372845, TC382045, CA646741, TC409077, TC382830, DR739471, TC434820, TC443387, CK212850, TC379711, TC371600, TC416438, TC399245, TC390630, TC392272, DR739350, TC407340, TC413339, TC369655, TC402072, TC402440, TC394307, TC370912, TC375146, TC432369, TC398592, TC389589, TC388819, TC394661, TC392778, TC370350, CK211589, TC435595, TC371037, TC386422, TC374164, TC388688, TC405041, TC439939, TC397019, TC388822, TC377496, TC368549, TC406708, TC401158, TC429771, TC385526, TC374461, TC397500, TC416658, TC405896, TC379965, TC381957, TC374230, TC393561, TC393948, TC375726, TC391671, TC393554, TC407614, TC400477, TC369687, TC388691, TC459656, DR734904, TC449724, TC413571, TC397909, CA729339, TC396487, TC411941, TC457991, TC378601, TC409187, TC389993, TC369664, TC389816, TC371172, TC383677, TC372167, TC393820, TC437163, TC379470, TC429747, TC379422, TC430501, TC387683, TC434831, TC396895, TC426326, TC393106, TC396460, TC428944 |
| [Transferase activity, transferring acyl groups](http://amigo.geneontology.org/cgi-bin/amigo/go.cgi?action=query&view=query&query=GO:0016746&search_constraint=terms) | TC410352, DR739471, TC434820, TC443387, TC379711, TC392272, TC407340, CA710880, TC398121, TC402440, TC423252, TC394307, TC397660, TC375146, TC389363, TC388914, TC434442, TC389589, TC446235, TC378072, TC392778, TC420735, TC456784, TC371037, TC386422, TC395453, TC388688, TC409843, TC390569, TC416685, TC403872, TC389090, TC389375, TC388822, TC377496, TC406708, TC382655, TC429771, CK200433, TC406038, TC416658, TC395351, TC374230, TC393561, TC401915, TC369928, TC391671, TC393554, TC407614, DR739994, TC384454, CA729339, CK217367, TC379853, TC389816, TC405440, DR737360, TC398514, TC396895, TC434831, TC426326, TC393106, BQ607161, TC396460, TC428944, TC382045, TC372845 |
| [Nucleic acid binding](http://amigo.geneontology.org/cgi-bin/amigo/go.cgi?action=query&view=query&query=GO:0003676&search_constraint=terms) | TC461921, TC410352, TC386040, TC407978, TC390393, TC416529, TC397258, TC389190, CV775873, TC396230, TC460760, TC451511, TC408524, TC427210, TC416069, BQ838511, TC398052, TC387861, CA710880, TC400388, TC398862, TC433162, TC394459, TC416154, TC384738, TC423252, TC397660, TC389363, TC388410, CK203550, TC416493, TC458562, TC406264, TC387410, TC378878, TC380063, TC398538, TC397176, TC418414, TC410063, TC378054, TC448471, TC395453, TC410194, TC392875, TC392329, TC409843, TC435546, TC384735, TC390569, TC405540, CA682223, TC407076, TC419057, TC403872, TC425847, CJ727624, TC398714, BQ294582, CA614761, CJ550278, TC457126, TC403580, TC413427, TC411128, TC401915, TC397444, TC398304, TC425878, TC393100, TC433957, TC417012, TC423239, CA632212, DR739994, TC459193, TC394916, TC394206, TC394820, TC398731, TC406236, TC418073, TC378274, TC391130, TC417308, CK217367, TC445767, TC389092, TC446038, TC423804, TC413460, TC379853, BQ609416, CK214702, TC449504, TC395723, TC405440, TC403968, CK213497, CJ792862, TC385233, TC440819, TC389718, TC404052, TC377225, TC381943, TC412520, TC432001, TC413392, BQ607161, TC406807, TC404606, TC369628, TC416906, TC402308, TC434820, TC441343, TC379711, TC400330, CA595837, TC379903, TC423265, TC392272, TC377061, TC401244, CA730421, TC417077, TC417388, TC446465, TC386313, CV759879, CK208222, TC408907, CA606693, TC391438, CK211707, TC386963, CK163367, TC412732, TC405695, CA700201, TC392778, TC396751, TC397312, TC405030, CK211589, TC388403, TC383909, TC376527, CA615187, TC416695, TC388718, TC419584, TC406469, TC385780, TC374392, TC405784, TC399919, TC407572, TC372175, TC402121, TC410690, TC406708, TC419747, TC409459, TC410074, TC402186, TC405356, CV763657, TC458205, TC402603, TC441241, TC449724, TC413571, TC413043, CA729339, CK214224, TC408299, BQ239045, CA709177, TC376263, TC416169, TC425690, TC381218, TC408229, TC422425, TC408312, CA613620, TC398805, TC369092, CV781430, TC388520, TC411191, TC399342, TC434831, TC426326, TC381462, TC375431, CD882425, TC373914, TC392263, TC392303, TC373145, TC445166, CA611770, TC435533, TC374240, CA720842, TC425841, CF554444, TC424204, TC387579, TC415685, TC406870, TC388914, TC393814, TC387621, TC398633, TC394716, TC460689, TC446235, TC402545, TC380943, TC393830, TC380433, TC403885, TC420735, TC404371, TC373615, TC384219, TC386961, TC416685, TC387344, TC372664, TC389090, TC412483, TC389375, TC382655, CK200433, TC370114, TC406038, TC395351, TC459245, TC418032, TC456619, CK210754, TC410147, TC384454, TC411287, TC380329, TC386237, TC393904, TC390994, TC391143, TC381463, TC403157, TC386414, TC395303, TC400755, TC403986, TC440636, TC406193, TC405615, TC376420, TC458987, TC377190, TC443814, TC404843, TC431879, TC422348, CD876572, TC385365, TC401210, DR737360, TC379357, TC390135, TC386279, TC397885, TC411684, TC411480, TC388049, TC380662, TC426358, TC389162, DR739471, TC402534, TC443387, TC415083, TC373429, TC386535, GH729256, TC369726, TC406584, TC386519, CA605200, TC402440, TC417992, TC391411, TC417341, TC394307, TC375146, TC388665, TC389589, TC381988, TC452762, TC386344, TC381963, TC383176, CD878039, TC371455, TC449463, TC374879, TC386422, TC374164, TC388688, TC388547, TC414606, TC388822, TC396365, TC377496, TC368549, TC388976, TC429771, TC410078, TC423576, TC386639, TC397500, TC416658, TC393561, TC393692, TC391671, TC393554, TC407614, TC459656, TC382928, TC379436, TC417363, TC403573, TC387710, TC413700, TC403929, TC394118, TC449043, TC379635, TC440321, TC433589, TC377410, TC389816, TC392323, TC413066, TC393820, CK211469, CB307332, TC404842, TC397729, TC403803, TC396895, TC418850, TC382342, TC393106, TC401758, TC432504, TC397676, TC396460, TC428944, TC389661, TC417067 |
| [Ligand-gated sodium channel activity](http://amigo.geneontology.org/cgi-bin/amigo/go.cgi?action=query&view=query&query=GO:0015280&search_constraint=terms) | TC410352, TC401915, DR739471, TC393561, TC434820, TC443387, TC379711, TC391671, TC393554, TC407614, TC392272, TC459656, DR739994, TC384454, CA729339, TC402440, CK217367, TC423252, TC394307, TC397660, TC375146, TC389363, TC379853, TC388914, TC389589, TC389816, TC446235, TC405440, TC392778, TC420735, TC386422, TC395453, DR737360, TC388688, TC416685, TC390569, TC403872, TC389090, TC434831, TC396895, TC389375, TC426326, TC388822, TC377496, TC406708, TC382655, TC393106, TC429771, CK200433, BQ607161, TC423576, TC406807, TC406038, TC396460, TC416658, TC428944, TC395351, TC386646 |
| [Acyltransferase activity](http://amigo.geneontology.org/cgi-bin/amigo/go.cgi?action=query&view=query&query=GO:0008415&search_constraint=terms) | TC410352, TC401915, DR739471, TC393561, TC434820, TC443387, TC379711, TC391671, TC393554, TC407614, TC392272, DR739994, TC384454, CA729339, CA710880, TC398121, TC402440, CK217367, TC423252, TC394307, TC397660, TC375146, TC389363, TC388914, TC379853, TC434442, TC389589, TC389816, TC446235, TC405440, TC378072, TC392778, TC420735, TC456784, TC371037, TC386422, TC395453, TC398514, DR737360, TC388688, TC409843, TC390569, TC416685, TC403872, TC389090, TC434831, TC396895, TC389375, TC426326, TC388822, TC377496, TC406708, TC382655, TC393106, TC429771, CK200433, BQ607161, TC406038, TC396460, TC416658, TC428944, TC395351, TC372845 |
| [Ligand-gated ion channel activity](http://amigo.geneontology.org/cgi-bin/amigo/go.cgi?action=query&view=query&query=GO:0015276&search_constraint=terms) | TC410352, TC401915, DR739471, TC393561, TC434820, TC443387, TC379711, TC391671, TC393554, TC407614, TC392272, TC459656, DR739994, TC384454, CA729339, TC402440, CK217367, TC423252, TC394307, TC397660, TC375146, TC389363, TC379853, TC388914, TC389589, TC389816, TC446235, TC405440, TC392778, TC420735, TC386422, TC395453, DR737360, TC388688, TC416685, TC390569, TC403872, TC389090, TC434831, TC396895, TC389375, TC426326, TC388822, TC377496, TC406708, TC382655, TC393106, TC429771, CK200433, BQ607161, TC423576, TC406807, TC406038, TC396460, TC416658, TC428944, TC395351, TC386646 |
| [Ligand-gated channel activity](http://amigo.geneontology.org/cgi-bin/amigo/go.cgi?action=query&view=query&query=GO:0022834&search_constraint=terms) | TC410352, TC401915, DR739471, TC393561, TC434820, TC443387, TC379711, TC391671, TC393554, TC407614, TC392272, TC459656, DR739994, TC384454, CA729339, TC402440, CK217367, TC423252, TC394307, TC397660, TC375146, TC389363, TC379853, TC388914, TC389589, TC389816, TC446235, TC405440, TC392778, TC420735, TC386422, TC395453, DR737360, TC388688, TC416685, TC390569, TC403872, TC389090, TC434831, TC396895, TC389375, TC426326, TC388822, TC377496, TC406708, TC382655, TC393106, TC429771, CK200433, BQ607161, TC423576, TC406807, TC406038, TC396460, TC416658, TC428944, TC395351, TC386646 |
| [Transferase activity, transferring acyl groups other than amino-acyl groups](http://amigo.geneontology.org/cgi-bin/amigo/go.cgi?action=query&view=query&query=GO:0016747&search_constraint=terms) | TC410352, TC401915, DR739471, TC393561, TC434820, TC443387, TC379711, TC391671, TC393554, TC407614, TC392272, DR739994, TC384454, CA729339, CA710880, TC398121, TC402440, CK217367, TC423252, TC394307, TC397660, TC375146, TC389363, TC388914, TC379853, TC434442, TC389589, TC389816, TC446235, TC405440, TC378072, TC392778, TC420735, TC456784, TC371037, TC386422, TC395453, TC398514, DR737360, TC388688, TC409843, TC390569, TC416685, TC403872, TC389090, TC434831, TC396895, TC389375, TC426326, TC388822, TC377496, TC406708, TC382655, TC393106, TC429771, CK200433, BQ607161, TC406038, TC396460, TC416658, TC428944, TC395351, TC372845 |
| [Sodium channel activity](http://amigo.geneontology.org/cgi-bin/amigo/go.cgi?action=query&view=query&query=GO:0005272&search_constraint=terms) | TC410352, TC401915, DR739471, TC393561, TC434820, TC443387, TC379711, TC391671, TC393554, TC407614, TC392272, TC459656, DR739994, TC384454, CA729339, TC402440, CK217367, TC423252, TC394307, TC397660, TC375146, TC389363, TC379853, TC388914, TC389589, TC389816, TC446235, TC405440, TC392778, TC420735, TC386422, TC395453, DR737360, TC388688, TC416685, TC390569, TC403872, TC389090, TC434831, TC396895, TC389375, TC426326, TC388822, TC377496, TC406708, TC382655, TC393106, TC429771, CK200433, BQ607161, TC423576, TC406807, TC406038, TC396460, TC416658, TC428944, TC395351, TC386646 |
| [Gated channel activity](http://amigo.geneontology.org/cgi-bin/amigo/go.cgi?action=query&view=query&query=GO:0022836&search_constraint=terms) | TC410352, TC401915, DR739471, TC393561, TC434820, TC456619, TC443387, TC379711, TC391671, TC393554, TC407614, TC392272, TC459656, DR739994, TC384454, CA729339, TC402440, CK217367, TC423252, TC394307, TC397660, TC375146, TC389363, TC388914, TC379853, TC370315, TC389589, TC389816, TC446235, TC405440, TC392778, TC420735, TC386422, TC395453, DR737360, TC388688, TC416685, TC390569, TC403872, TC389090, TC434831, TC396895, TC389375, TC426326, TC388822, TC373002, TC377496, TC406708, TC382655, TC393106, TC429771, CK200433, TC423576, BQ607161, TC406807, TC406038, TC396460, TC416658, TC428944, TC395351, TC386646 |
| [Damaged DNA binding](http://amigo.geneontology.org/cgi-bin/amigo/go.cgi?action=query&view=query&query=GO:0003684&search_constraint=terms) | TC386040, TC402308, TC393692, TC397258, TC402603, TC379436, TC411287, TC435533, TC386519, TC374240, CV759879, CK214224, TC408907, TC391143, TC433162, TC391438, TC433589, TC386414, TC398633, TC394716, TC440636, TC395723, TC380063, TC380943, TC393830, TC386344, CK211469, TC405030, TC378054, TC381963, TC388403, TC383176, TC404371, TC389718, TC384219, TC379357, TC386279, TC385780, TC381943, TC388976, TC382342, TC373914, TC432504, TC386639, TC397676, TC402186 |
| [Syntaxin-1 binding](http://amigo.geneontology.org/cgi-bin/amigo/go.cgi?action=query&view=query&query=GO:0017075&search_constraint=terms) | TC398304, TC393100, TC433957, CK210754, TC382928, CV775873, TC441241, TC417363, TC386237, TC394916, TC386313, TC390994, TC398052, TC425841, TC378274, TC403929, TC387579, CA606693, TC394459, TC384738, CK211707, TC386963, TC388410, TC395303, TC422425, TC458562, TC400755, TC458987, TC396751, CA615187, TC386961, TC398514, TC390135, TC377225, TC411191, TC388547, TC387344, TC405784, TC399919, TC407572, TC372175, TC396365, TC419747, TC380662, TC401758, TC398714, TC389661 |
| [Cation channel activity](http://amigo.geneontology.org/cgi-bin/amigo/go.cgi?action=query&view=query&query=GO:0005261&search_constraint=terms) | TC410352, TC401915, DR739471, TC393561, TC434820, TC456619, TC443387, TC379711, TC391671, TC393554, TC407614, TC392272, TC459656, DR739994, TC384454, TC413571, CA729339, TC402440, CK217367, TC423252, TC394307, TC397660, TC375146, TC389363, TC379853, TC388914, TC370315, TC389589, TC389816, TC446235, TC405440, TC392778, CK211589, TC420735, TC386422, TC395453, DR737360, TC388688, TC416685, TC390569, TC403872, TC389090, TC434831, TC396895, TC389375, TC426326, TC388822, TC377496, TC406708, TC382655, TC393106, TC429771, CK200433, BQ607161, TC423576, TC406807, TC406038, TC396460, TC416658, TC428944, TC395351, TC386646 |
| [Syntaxin binding](http://amigo.geneontology.org/cgi-bin/amigo/go.cgi?action=query&view=query&query=GO:0019905&search_constraint=terms) | TC398304, TC393100, TC433957, CK210754, TC382928, CV775873, TC441241, TC417363, TC386237, TC394916, TC386313, TC390994, TC398052, TC425841, TC378274, TC403929, TC387579, CA606693, TC394459, TC384738, CK211707, TC386963, TC388410, TC395303, TC422425, TC458562, TC400755, TC458987, TC396751, CA615187, TC386961, TC398514, TC390135, TC377225, TC411191, TC388547, TC387344, TC405784, TC399919, TC407572, TC372175, TC396365, TC419747, TC380662, TC401758, TC398714, TC389661 |
| [Ion channel activity](http://amigo.geneontology.org/cgi-bin/amigo/go.cgi?action=query&view=query&query=GO:0005216&search_constraint=terms) | TC410352, TC401915, DR739471, TC393561, TC434820, TC456619, TC443387, TC379711, TC391671, TC393554, TC407614, TC392272, TC459656, DR739994, TC384454, TC413571, CA729339, TC402440, CK217367, TC423252, TC394307, TC397660, TC375146, TC389363, TC388914, TC379853, TC370315, TC389589, TC389816, TC446235, TC405440, TC392778, CK211589, TC420735, TC386422, TC395453, DR737360, TC388688, TC416685, TC390569, TC403872, TC389090, TC434831, TC396895, TC389375, TC426326, TC388822, TC373002, TC377496, TC406708, TC382655, TC393106, TC429771, CK200433, TC423576, BQ607161, TC406807, TC406038, TC396460, TC416658, TC428944, TC395351, TC386646 |
| [Thyroid-stimulating hormone receptor activity](http://amigo.geneontology.org/cgi-bin/amigo/go.cgi?action=query&view=query&query=GO:0004996&search_constraint=terms) | TC410352, TC401915, DR739471, TC393561, TC434820, TC443387, TC379711, TC391671, TC393554, TC407614, TC392272, DR739994, TC384454, CA729339, CA710880, TC402440, CK217367, TC423252, TC394307, TC397660, TC375146, TC389363, TC379853, TC388914, TC389589, TC389816, TC446235, TC405440, CA613620, TC392778, TC420735, TC386422, TC395453, DR737360, TC388688, TC416685, TC390569, TC403872, TC389090, TC434831, TC396895, TC389375, TC426326, TC388822, TC377496, TC406708, TC382655, TC393106, TC429771, CK200433, TC423576, BQ294582, BQ607161, TC406038, TC396460, TC416658, TC428944, TC395351 |
| [Protein-hormone receptor activity](http://amigo.geneontology.org/cgi-bin/amigo/go.cgi?action=query&view=query&query=GO:0016500&search_constraint=terms) | TC410352, TC401915, DR739471, TC393561, TC434820, TC443387, TC379711, TC391671, TC393554, TC407614, TC392272, DR739994, TC384454, CA729339, CA710880, TC402440, CK217367, TC423252, TC394307, TC397660, TC375146, TC389363, TC379853, TC388914, TC389589, TC389816, TC446235, TC405440, CA613620, TC392778, TC420735, TC386422, TC395453, DR737360, TC388688, TC416685, TC390569, TC403872, TC389090, TC434831, TC396895, TC389375, TC426326, TC388822, TC377496, TC406708, TC382655, TC393106, TC429771, CK200433, TC423576, BQ294582, BQ607161, TC406038, TC396460, TC416658, TC428944, TC395351 |
| [SNARE binding](http://amigo.geneontology.org/cgi-bin/amigo/go.cgi?action=query&view=query&query=GO:0000149&search_constraint=terms) | TC398304, TC393100, TC433957, CK210754, TC382928, CV775873, TC441241, TC417363, TC386237, TC394916, TC386313, TC390994, TC398052, TC425841, TC378274, TC403929, TC387579, CA606693, TC394459, TC384738, CK211707, TC386963, TC388410, TC395303, TC422425, TC458562, TC400755, TC458987, TC396751, CA615187, TC386961, TC398514, TC390135, TC377225, TC411191, TC388547, TC387344, TC405784, TC399919, TC407572, TC372175, TC396365, TC419747, TC380662, TC401758, TC398714, TC389661 |
| [G-protein coupled receptor activity](http://amigo.geneontology.org/cgi-bin/amigo/go.cgi?action=query&view=query&query=GO:0004930&search_constraint=terms) | TC410352, DR739471, TC434820, TC416906, TC443387, TC379711, TC392272, CV775873, TC377061, TC417388, TC386313, TC398052, CA710880, TC425841, TC402440, TC398862, TC387579, TC394459, TC371242, TC384738, TC423252, TC394307, TC397660, CK211707, TC375146, TC386963, TC389363, TC388914, TC388410, TC389589, TC446235, TC418414, TC392778, TC397312, TC420735, TC383909, TC449463, TC395453, TC386422, TC386961, TC388688, TC390569, TC416685, TC407076, TC403872, TC399919, TC407572, TC389090, TC372175, TC389375, TC388822, TC396365, TC406708, TC377496, TC419747, TC382655, TC429771, CK200433, TC423576, BQ294582, TC406038, TC416658, TC395351, TC401915, TC393561, TC393100, TC391671, TC393554, TC407614, TC382928, DR739994, TC438243, TC449724, TC384454, CA729339, TC417363, TC386237, TC393904, TC387710, CK217367, TC425690, TC379853, TC377410, TC381218, TC395303, TC389816, TC400755, TC405440, TC458987, CA613620, TC393820, TC401210, DR737360, TC390135, TC397729, TC377225, TC434831, TC432185, TC396895, TC426326, TC381462, TC393106, TC401758, BQ607161, TC396460, TC392263, TC428944, TC389661, TC404606 |
| [Myosin binding](http://amigo.geneontology.org/cgi-bin/amigo/go.cgi?action=query&view=query&query=GO:0017022&search_constraint=terms) | TC398304, TC393948, TC393100, TC433957, CK210754, TC390630, TC382928, CV775873, TC441241, TC417363, TC386237, TC394916, TC386313, TC390994, TC398052, TC425841, TC378274, TC403929, TC387579, CA606693, TC394459, TC384738, CK211707, TC386963, TC388410, TC395303, TC422425, TC458562, TC400755, TC458987, TC396751, CA615187, TC386961, TC398514, TC390135, TC377225, TC411191, TC388547, TC387344, TC405784, TC399919, TC407572, TC372175, TC396365, TC419747, TC380662, TC401758, TC398714, TC389661 |
| [Transmembrane receptor activity](http://amigo.geneontology.org/cgi-bin/amigo/go.cgi?action=query&view=query&query=GO:0004888&search_constraint=terms) | TC410352, DR739471, TC434820, TC416906, TC443387, TC379711, TC392272, CV775873, TC377061, TC417388, TC386313, TC398052, CA710880, TC425841, TC402440, TC398862, TC387579, TC394459, TC371242, TC384738, TC423252, TC394307, TC397660, CK211707, TC375146, TC386963, TC389363, TC388914, TC388410, TC389589, TC446235, TC418414, TC392778, TC397312, CK211589, TC420735, TC383909, TC449463, TC395453, TC386422, TC386961, TC388688, TC390569, TC416685, TC407076, TC403872, TC399919, TC407572, TC389090, TC372175, TC389375, TC388822, TC396365, TC406708, TC377496, TC419747, TC382655, TC429771, CK200433, TC423576, BQ294582, TC406038, TC416658, TC395351, TC401915, TC393561, TC393100, TC391671, TC393554, TC407614, TC382928, TC459656, DR739994, TC438243, TC449724, TC384454, TC413571, CA729339, TC417363, TC386237, TC393904, TC387710, CK217367, TC394118, TC380882, TC425690, TC379853, TC377410, TC381218, TC395303, TC389816, TC400755, TC405440, TC376420, TC458987, TC461622, CA613620, TC393820, TC403588, TC401210, DR737360, TC390135, TC397729, TC377225, TC434831, TC396895, TC432185, TC426326, TC381462, TC393106, TC401758, BQ607161, TC406807, TC396460, TC392263, TC428944, TC389661, TC404606 |
| [Signal transducer activity](http://amigo.geneontology.org/cgi-bin/amigo/go.cgi?action=query&view=query&query=GO:0004871&search_constraint=terms) | TC410352, CV775873, TC398052, CA710880, TC425841, TC398862, TC387579, TC394459, TC371242, TC384738, TC423252, TC397660, TC389363, TC388914, TC388410, TC446235, TC418414, TC380433, TC420735, TC395453, TC386961, TC392329, TC390569, TC416685, TC407076, TC403872, TC389090, TC389375, TC382655, CK200433, BQ294582, TC406038, TC395351, TC390904, TC401915, TC393100, DR739994, TC384454, TC380329, TC386237, TC393904, CK217367, TC379853, TC395303, TC400755, TC405440, TC403968, TC376420, TC458987, TC423110, TC379536, TC401210, DR737360, TC390135, TC377225, BQ607161, TC406807, TC404606, DR739471, TC416906, TC434820, TC443387, TC379711, TC392272, TC377061, TC417388, TC386313, TC413339, TC402440, TC420579, TC394307, TC375146, CK211707, TC386963, TC398592, TC389589, TC392778, TC397312, CK211589, TC449463, TC383909, TC386422, TC388688, TC405041, TC372175, TC407572, TC399919, TC377496, TC406708, TC396365, TC388822, TC429771, TC419747, TC423576, TC416658, TC393561, TC391671, TC407614, TC393554, TC459656, TC382928, TC438243, TC449724, TC413571, CA729339, TC417363, TC387710, TC394118, TC380882, TC425690, TC377410, TC381218, TC389816, CA613620, TC393820, TC461622, TC403588, TC397729, TC432185, TC396895, TC434831, TC426326, TC381462, TC393106, TC401758, TC392263, TC396460, TC389661, TC428944 |
| [Molecular transducer activity](http://amigo.geneontology.org/cgi-bin/amigo/go.cgi?action=query&view=query&query=GO:0060089&search_constraint=terms) | TC410352, CV775873, TC398052, CA710880, TC425841, TC398862, TC387579, TC394459, TC371242, TC384738, TC423252, TC397660, TC389363, TC388914, TC388410, TC446235, TC418414, TC380433, TC420735, TC395453, TC386961, TC392329, TC390569, TC416685, TC407076, TC403872, TC389090, TC389375, TC382655, CK200433, BQ294582, TC406038, TC395351, TC390904, TC401915, TC393100, DR739994, TC384454, TC380329, TC386237, TC393904, CK217367, TC379853, TC395303, TC400755, TC405440, TC403968, TC376420, TC458987, TC423110, TC379536, TC401210, DR737360, TC390135, TC377225, BQ607161, TC406807, TC404606, DR739471, TC416906, TC434820, TC443387, TC379711, TC392272, TC377061, TC417388, TC386313, TC413339, TC402440, TC420579, TC394307, TC375146, CK211707, TC386963, TC398592, TC389589, TC392778, TC397312, CK211589, TC449463, TC383909, TC386422, TC388688, TC405041, TC372175, TC407572, TC399919, TC377496, TC406708, TC396365, TC388822, TC429771, TC419747, TC423576, TC416658, TC393561, TC391671, TC407614, TC393554, TC459656, TC382928, TC438243, TC449724, TC413571, CA729339, TC417363, TC387710, TC394118, TC380882, TC425690, TC377410, TC381218, TC389816, CA613620, TC393820, TC461622, TC403588, TC397729, TC432185, TC396895, TC434831, TC426326, TC381462, TC393106, TC401758, TC392263, TC396460, TC389661, TC428944 |
| [Phospholipid binding](http://amigo.geneontology.org/cgi-bin/amigo/go.cgi?action=query&view=query&query=GO:0005543&search_constraint=terms) | TC398304, TC393100, TC433957, CK210754, TC459656, TC382928, CV775873, TC441241, TC417363, TC386237, TC394916, TC386313, TC390994, TC398052, TC425841, TC378274, TC403929, TC387579, CA606693, TC394459, TC384738, CK211707, TC386963, TC388410, TC395303, TC422425, TC458562, TC400755, TC458987, TC396751, CA615187, TC386961, TC398514, TC390135, TC377225, TC411191, TC388547, TC387344, TC405784, TC399919, TC407572, TC372175, TC396365, TC419747, TC380662, TC401758, TC398714, TC406807, TC389661 |
| [Receptor activity](http://amigo.geneontology.org/cgi-bin/amigo/go.cgi?action=query&view=query&query=GO:0004872&search_constraint=terms) | TC410352, DR739471, TC434820, TC416906, TC443387, TC379711, TC392272, CV775873, TC377061, TC417388, TC386313, TC398052, CA710880, TC425841, TC402440, TC398862, TC387579, TC394459, TC371242, TC384738, TC423252, TC394307, TC397660, CK211707, TC375146, TC386963, TC389363, TC388914, TC388410, TC389589, TC446235, TC418414, TC392778, TC397312, TC380433, CK211589, TC420735, TC383909, TC449463, TC395453, TC386422, TC386961, TC405041, TC388688, TC390569, TC416685, TC407076, TC403872, TC399919, TC407572, TC389090, TC372175, TC389375, TC388822, TC396365, TC406708, TC377496, TC419747, TC382655, TC429771, CK200433, TC423576, BQ294582, TC406038, TC416658, TC395351, TC401915, TC393561, TC393100, TC391671, TC393554, TC407614, TC382928, TC459656, TC438243, DR739994, TC449724, TC384454, TC413571, CA729339, TC417363, TC386237, TC393904, TC387710, CK217367, TC394118, TC380882, TC425690, TC379853, TC377410, TC381218, TC395303, TC389816, TC400755, TC405440, TC376420, TC458987, TC461622, CA613620, TC393820, TC403588, TC401210, DR737360, TC390135, TC397729, TC377225, TC434831, TC396895, TC432185, TC426326, TC381462, TC393106, TC401758, BQ607161, TC406807, TC396460, TC392263, TC428944, TC389661, TC404606 |
| [Adenylate cyclase inhibiting metabotropic glutamate receptor activity](http://amigo.geneontology.org/cgi-bin/amigo/go.cgi?action=query&view=query&query=GO:0001640&search_constraint=terms) | TC393100, TC382928, CV775873, TC417363, TC386237, TC386313, TC393904, TC398052, TC425841, TC387579, TC394459, TC384738, CK211707, TC386963, TC388410, TC377410, TC381218, TC395303, TC400755, TC458987, TC397312, TC401210, TC386961, TC390135, TC397729, TC377225, TC372175, TC407572, TC399919, TC396365, TC381462, TC419747, TC401758, TC392263, TC389661 |
| [Group III metabotropic glutamate receptor activity](http://amigo.geneontology.org/cgi-bin/amigo/go.cgi?action=query&view=query&query=GO:0001642&search_constraint=terms) | TC393100, TC382928, CV775873, TC417363, TC386237, TC386313, TC393904, TC398052, TC425841, TC387579, TC394459, TC384738, CK211707, TC386963, TC388410, TC377410, TC381218, TC395303, TC400755, TC458987, TC397312, TC401210, TC386961, TC390135, TC397729, TC377225, TC372175, TC407572, TC399919, TC396365, TC381462, TC419747, TC401758, TC392263, TC389661 |
| [RNA polymerase II transcription factor activity](http://amigo.geneontology.org/cgi-bin/amigo/go.cgi?action=query&view=query&query=GO:0003702&search_constraint=terms) | TC386040, TC402308, TC393692, TC390393, TC379903, TC397258, TC402603, TC435533, TC386519, CV759879, TC391143, TC433162, TC391438, TC393814, TC433589, TC386414, TC398633, TC394716, TC440636, TC395723, TC452762, TC380063, TC380943, TC393830, TC386344, CK211469, TC405030, TC378054, TC381963, TC388403, TC383176, TC389718, TC384219, TC387135, TC385780, TC419057, TC381943, TC388976, TC382342, TC373914, TC432504, TC386639, TC397676, CJ550278 |
| [Substrate-specific channel activity](http://amigo.geneontology.org/cgi-bin/amigo/go.cgi?action=query&view=query&query=GO:0022838&search_constraint=terms) | TC410352, DR739471, TC434820, TC443387, TC379711, TC392272, TC402440, TC423252, TC394307, TC397660, TC375146, TC389363, TC388914, TC389589, TC446235, TC392778, CK211589, TC420735, TC386422, TC395453, TC388688, TC390569, TC416685, TC403872, TC389090, TC389375, TC388822, TC373002, TC377496, TC406708, TC382655, TC429771, CK200433, TC423576, TC406038, TC416658, TC395351, TC386646, TC393561, TC401915, TC456619, TC391671, TC407614, TC393554, TC459656, DR739994, TC384454, TC413571, CA729339, CK217367, TC379853, TC389816, TC370315, TC405440, TC376933, DR737360, TC396895, TC434831, TC426326, TC393106, BQ607161, TC396460, TC406807, TC428944 |
| [Channel activity](http://amigo.geneontology.org/cgi-bin/amigo/go.cgi?action=query&view=query&query=GO:0015267&search_constraint=terms) | TC410352, DR739471, TC434820, TC443387, TC379711, TC392272, TC402440, TC423252, TC394307, TC397660, TC375146, TC389363, TC388914, TC389589, TC446235, TC392778, CK211589, TC420735, TC386422, TC395453, TC388688, TC390569, TC416685, TC403872, TC389090, TC389375, TC388822, TC373002, TC377496, TC406708, TC382655, TC429771, CK200433, TC423576, TC406038, TC416658, TC395351, TC386646, TC393561, TC401915, TC456619, TC391671, TC407614, TC393554, TC459656, DR739994, TC384454, TC413571, CA729339, CK217367, TC379853, TC389816, TC370315, TC405440, TC376933, DR737360, TC396895, TC434831, TC426326, TC393106, BQ607161, TC396460, TC406807, TC428944 |
| [Passive transmembrane transporter activity](http://amigo.geneontology.org/cgi-bin/amigo/go.cgi?action=query&view=query&query=GO:0022803&search_constraint=terms) | TC410352, DR739471, TC434820, TC443387, TC379711, TC392272, TC402440, TC423252, TC394307, TC397660, TC375146, TC389363, TC388914, TC389589, TC446235, TC392778, CK211589, TC420735, TC386422, TC395453, TC388688, TC390569, TC416685, TC403872, TC389090, TC389375, TC388822, TC373002, TC377496, TC406708, TC382655, TC429771, CK200433, TC423576, TC406038, TC416658, TC395351, TC386646, TC393561, TC401915, TC456619, TC391671, TC407614, TC393554, TC459656, DR739994, TC384454, TC413571, CA729339, CK217367, TC379853, TC389816, TC370315, TC405440, TC376933, DR737360, TC396895, TC434831, TC426326, TC393106, BQ607161, TC396460, TC406807, TC428944 |
| [Calmodulin binding](http://amigo.geneontology.org/cgi-bin/amigo/go.cgi?action=query&view=query&query=GO:0005516&search_constraint=terms) | CA646741, TC398304, TC393100, TC433957, CK210754, TC459656, TC382928, CV775873, TC441241, TC417363, TC386237, TC394916, TC374240, TC386313, TC390994, TC398052, TC425841, TC378274, TC403929, TC387579, CA606693, TC394459, TC384738, CK211707, TC386963, TC388410, TC395303, TC422425, TC458562, TC400755, TC395723, TC376420, TC458987, TC400056, CJ944525, TC396751, CA615187, TC386961, TC398514, TC390135, TC377225, TC411191, TC388547, TC387344, TC405784, TC399919, TC407572, TC372175, TC396365, TC419747, TC380662, TC401758, TC398714, TC406807, CJ550278, TC389661 |
| [Glutamate receptor activity](http://amigo.geneontology.org/cgi-bin/amigo/go.cgi?action=query&view=query&query=GO:0008066&search_constraint=terms) | TC393100, TC382928, CV775873, TC413571, TC417363, TC386237, TC386313, TC393904, TC398052, TC425841, TC387579, TC394459, TC384738, CK211707, TC386963, TC388410, TC377410, TC381218, TC395303, TC400755, TC458987, TC397312, CK211589, TC401210, TC386961, TC390135, TC397729, TC377225, TC372175, TC407572, TC399919, TC396365, TC381462, TC419747, TC401758, TC392263, TC389661 |
| [Nucleic acid binding transcription factor activity](http://amigo.geneontology.org/cgi-bin/amigo/go.cgi?action=query&view=query&query=GO:0001071&search_constraint=terms) | TC410352, TC386040, TC390393, TC397258, TC435533, TC374240, CA710880, TC398862, TC433162, TC423252, TC397660, TC393814, TC389363, TC388914, TC394716, TC398633, TC446235, TC380063, TC402545, TC393830, TC380943, TC418414, TC378054, TC420735, TC404371, TC395453, TC384219, TC384735, TC390569, TC416685, TC403872, TC389090, TC389375, TC404413, TC382655, CK200433, TC370114, TC406038, TC408192, TC395351, TC401915, DR739994, TC384454, TC411287, TC391143, CK217367, TC379853, TC386414, TC440636, TC395723, TC405440, TC406193, TC385233, TC389718, TC379357, DR737360, TC386279, TC381943, BQ607161, TC393960, DR739471, TC416906, TC402308, TC434820, TC441343, TC443387, TC379711, TC379903, TC392272, TC417077, TC417388, TC386519, CV759879, TC408907, TC402440, TC391438, TC394307, TC375146, TC389589, TC452762, TC386344, TC392778, TC405030, TC388403, CK211589, TC381963, TC383176, TC386422, TC374879, TC388688, TC385780, TC377496, TC388976, TC406708, TC388822, TC429771, TC409459, TC423576, TC386639, TC416658, TC402186, TC393561, TC393692, TC391671, TC407614, TC393554, TC402603, TC379436, TC413571, CA729339, TC460615, CK214224, TC433589, TC389816, CA613620, CK211469, TC400362, TC381817, TC395872, TC396895, TC434831, TC418850, TC382342, TC426326, TC393106, TC397676, TC396460, TC432504, TC373914, TC428944 |
| [Sequence-specific DNA binding transcription factor activity](http://amigo.geneontology.org/cgi-bin/amigo/go.cgi?action=query&view=query&query=GO:0003700&search_constraint=terms) | TC410352, TC386040, TC390393, TC397258, TC435533, TC374240, CA710880, TC398862, TC433162, TC423252, TC397660, TC393814, TC389363, TC388914, TC394716, TC398633, TC446235, TC380063, TC402545, TC393830, TC380943, TC418414, TC378054, TC420735, TC404371, TC395453, TC384219, TC384735, TC390569, TC416685, TC403872, TC389090, TC389375, TC404413, TC382655, CK200433, TC370114, TC406038, TC408192, TC395351, TC401915, DR739994, TC384454, TC411287, TC391143, CK217367, TC379853, TC386414, TC440636, TC395723, TC405440, TC406193, TC385233, TC389718, TC379357, DR737360, TC386279, TC381943, BQ607161, TC393960, DR739471, TC416906, TC402308, TC434820, TC441343, TC443387, TC379711, TC379903, TC392272, TC417077, TC417388, TC386519, CV759879, TC408907, TC402440, TC391438, TC394307, TC375146, TC389589, TC452762, TC386344, TC392778, TC405030, TC388403, CK211589, TC381963, TC383176, TC386422, TC374879, TC388688, TC385780, TC377496, TC388976, TC406708, TC388822, TC429771, TC409459, TC423576, TC386639, TC416658, TC402186, TC393561, TC393692, TC391671, TC407614, TC393554, TC402603, TC379436, TC413571, CA729339, TC460615, CK214224, TC433589, TC389816, CA613620, CK211469, TC400362, TC381817, TC395872, TC396895, TC434831, TC418850, TC382342, TC426326, TC393106, TC397676, TC396460, TC432504, TC373914, TC428944 |
| [Glutathione transferase activity](http://amigo.geneontology.org/cgi-bin/amigo/go.cgi?action=query&view=query&query=GO:0004364&search_constraint=terms) | TC427986, TC378846, TC371600, TC405652, TC376202, TC369687, TC400477, TC388691, TC440499, DR734904, TC397019, TC397793, TC396487, CK197833, TC439324, TC439423, TC374461, TC392074, TC390319, TC419727, CK199846 |
| [Transcription regulator activity](http://amigo.geneontology.org/cgi-bin/amigo/go.cgi?action=query&view=query&query=GO:0030528&search_constraint=terms) | TC386040, TC402308, TC393692, TC390393, TC379903, TC397258, TC402603, TC459656, TC372677, TC380329, TC460615, TC435533, TC386519, CV759879, TC391143, TC433162, TC394118, TC420579, TC391438, TC393814, TC433589, TC386414, TC394716, TC398633, TC404926, TC440636, TC395723, TC452762, TC403968, TC380063, TC393830, TC380943, TC386344, CK211469, TC400362, TC380433, TC378054, TC405030, TC381963, TC388403, TC383176, TC389718, TC384219, TC387135, TC392329, TC373129, TC385780, TC419057, TC381943, TC404413, TC382342, TC388976, TC418850, TC370114, TC409459, TC403977, TC373914, TC406807, TC432504, TC386639, CJ550278, TC397676, TC393960 |
| [Oxidoreductase activity, acting on the CH-CH group of donors, NAD or NADP as acceptor](http://amigo.geneontology.org/cgi-bin/amigo/go.cgi?action=query&view=query&query=GO:0016628&search_constraint=terms) | TC392033, TC398121, TC375726, TC440526, TC409187, TC389993, TC430544, TC391946, TC370633, TC371037, TC427405, TC421880, TC398536, TC397909, TC379965, TC411908 |
| [Cytoskeletal protein binding](http://amigo.geneontology.org/cgi-bin/amigo/go.cgi?action=query&view=query&query=GO:0008092&search_constraint=terms) | TC390630, CV775873, TC445166, TC374240, TC386313, TC398052, TC425841, TC387579, CA606693, TC394459, TC384738, CK211707, TC386963, TC388410, TC458562, TC381988, TC421914, TC378271, CJ944525, TC396751, TC373637, CA615187, TC386961, TC388547, TC387344, TC405784, TC399919, TC407572, TC372175, TC396365, TC419747, TC410078, TC370114, TC409459, TC398714, CJ550278, TC398304, TC433957, TC393100, TC393948, CK210754, TC382928, TC459656, TC441241, TC386237, TC417363, TC394916, TC390994, TC378274, TC403929, TC395303, TC422425, TC400755, TC395723, TC458987, TC404843, TC390135, TC398514, TC377225, TC411191, TC380662, TC401758, TC406807, TC389661 |
| [12-oxophytodienoate Reductase activity](http://amigo.geneontology.org/cgi-bin/amigo/go.cgi?action=query&view=query&query=GO:0016629&search_constraint=terms) | TC392033, TC375726, TC409187, TC389993, TC430544, TC391946, TC370633, TC398536, TC397909, TC379965, TC411908 |
| [Carbohydrate binding](http://amigo.geneontology.org/cgi-bin/amigo/go.cgi?action=query&view=query&query=GO:0030246&search_constraint=terms) | TC405079, CJ854725, TC372563, TC375726, TC377308, TC430544, TC369807, TC391946, TC370633, TC397909, TC411908, TC392033, DR740372, CK201148, CK201126, TC369199, TC409187, TC384010, TC389993, TC379965, TC398536 |
| [Oxidoreductase activity, acting on the CH-CH group of donors](http://amigo.geneontology.org/cgi-bin/amigo/go.cgi?action=query&view=query&query=GO:0016627&search_constraint=terms) | TC392033, TC398121, TC375726, TC440526, TC409187, TC389993, TC430544, TC391946, TC431198, TC370633, TC371037, TC427405, TC421880, TC398536, TC397909, TC379965, TC411908 |
| [Protein binding](http://amigo.geneontology.org/cgi-bin/amigo/go.cgi?action=query&view=query&query=GO:0005515&search_constraint=terms) | TC410352, TC386040, TC373613, TC407978, TC390393, TC416529, TC397258, CV775873, TC396230, TC451511, TC416069, BQ838511, TC398052, TC375313, CA710880, TC398862, TC433162, TC376351, TC394459, TC371242, TC416154, TC384738, TC423252, TC397660, TC389363, TC379171, TC388410, TC416493, TC458562, TC421914, TC387410, TC380063, TC398538, TC418414, CJ944525, TC410063, TC378054, TC373702, TC395453, TC410194, TC409843, TC384735, TC390569, TC405540, TC407076, TC419057, TC403872, TC398714, BQ294582, CJ550278, TC403580, TC401915, TC397444, TC398304, TC423374, TC393100, TC433957, TC417012, CA632212, DR739994, TC394916, TC394820, TC406236, TC378274, TC391130, CK217367, TC409599, TC446038, TC423804, TC379853, TC370315, CK214702, TC395723, TC405440, CK213497, TC385233, TC389718, TC398514, TC377225, TC381943, TC412520, TC413392, BQ607161, TC406807, TC382045, CA646741, TC416906, TC402308, TC434820, TC441343, TC379711, TC378790, TC379903, TC423265, TC392272, TC377061, TC401244, TC417077, TC417388, TC386313, CV759879, TC408907, CA606693, TC391438, CK211707, TC386963, TC412732, TC421345, TC405695, TC392778, TC396751, TC405030, CK211589, TC388403, TC383909, CA615187, TC416695, TC383701, BJ317882, TC385780, TC406469, TC405784, TC399919, TC407572, TC372175, TC410690, TC406708, TC419747, TC409459, TC402186, TC386646, TC405356, TC423354, TC402603, TC441241, TC449724, TC413571, TC413043, CA729339, CK214224, TC408299, TC376263, TC416169, TC425690, TC422425, CA613620, TC400362, TC411191, TC399342, TC434831, TC432185, TC426326, TC373914, TC396529, TC394796, TC445166, TC435533, TC374240, TC425841, TC424204, TC387579, TC388914, TC393814, TC398633, TC394716, TC368548, TC446235, TC369633, TC378271, TC402545, TC380943, TC393830, TC380433, TC403885, TC420735, TC404371, TC384219, TC386961, TC397562, TC416685, TC372664, TC387344, TC412483, TC389090, TC389375, TC404413, TC382655, TC370114, TC373994, CK200433, TC406038, TC393970, TC395351, TC418032, TC456619, CK210754, TC376490, TC384454, TC411287, TC386237, TC390994, TC391143, TC428066, TC386414, TC395303, TC400755, TC440636, TC405511, TC403986, TC376420, TC458987, TC377190, TC404843, TC423110, TC385365, DR737360, TC379357, TC390135, TC387135, TC386279, TC411684, TC373129, TC420394, TC409650, TC380662, TC393960, TC389162, DR739471, TC402534, TC443387, TC388566, TC415083, TC390630, TC407340, TC406584, TC386519, TC390402, TC402440, TC417992, TC417341, TC394307, TC375146, TC375813, TC389589, TC381988, TC452762, TC386344, TC373637, TC381963, TC383176, TC449463, TC386422, TC388688, TC388547, TC388822, TC377496, TC388976, TC396365, TC429771, TC410078, TC423576, TC386639, TC375530, TC416658, TC374230, TC393561, TC393948, TC435799, TC393692, TC391671, TC393554, TC407614, TC459656, TC382928, TC438243, TC379436, TC460615, TC417363, TC411941, TC403929, TC394118, TC380882, TC379635, TC423880, TC373259, TC433589, TC389816, TC392323, TC413066, TC400056, TC393820, TC461622, CK211469, CB307332, TC404842, TC435808, TC381817, TC403588, TC375798, TC403803, TC396895, TC391122, TC418850, TC382342, TC393106, TC401758, TC432504, TC397676, TC396460, TC389661, TC428944 |
| [Ion transmembrane transporter activity](http://amigo.geneontology.org/cgi-bin/amigo/go.cgi?action=query&view=query&query=GO:0015075&search_constraint=terms) | TC410352, DR739471, TC434820, TC443387, TC379711, TC373958, TC392272, TC429341, TC397994, TC402440, TC371242, TC423252, TC394307, TC397660, TC375146, TC389363, TC388914, TC373583, TC389589, TC446235, TC392778, TC432154, CK211589, TC420735, TC373678, TC386422, TC395453, TC388688, TC387319, TC390569, TC369182, TC416685, TC403872, TC389090, TC389375, TC388822, TC406708, TC373002, TC377496, TC382655, TC429771, CK200433, TC423576, TC406038, TC416658, TC395351, TC386646, TC392709, TC393561, TC401915, TC456619, TC391671, TC393554, TC407614, TC459656, DR739994, TC384454, TC413571, CA729339, CK217367, TC378153, TC429374, TC379853, TC389816, TC370315, TC405440, TC384344, DR737360, TC444402, TC396895, TC434831, TC426326, TC393106, BQ607161, TC406807, TC396460, TC428944 |
| [Cation transmembrane transporter activity](http://amigo.geneontology.org/cgi-bin/amigo/go.cgi?action=query&view=query&query=GO:0008324&search_constraint=terms) | TC410352, DR739471, TC434820, TC443387, TC379711, TC373958, TC392272, TC429341, TC397994, TC402440, TC371242, TC423252, TC394307, TC397660, TC375146, TC389363, TC388914, TC373583, TC389589, TC446235, TC392778, CK211589, TC420735, TC373678, TC386422, TC395453, TC388688, TC390569, TC369182, TC416685, TC403872, TC389090, TC389375, TC388822, TC406708, TC377496, TC382655, TC429771, CK200433, TC423576, TC406038, TC416658, TC395351, TC386646, TC392709, TC393561, TC401915, TC456619, TC391671, TC393554, TC407614, TC459656, DR739994, TC384454, TC413571, CA729339, CK217367, TC378153, TC429374, TC379853, TC389816, TC370315, TC405440, TC384344, DR737360, TC444402, TC396895, TC434831, TC426326, TC393106, BQ607161, TC406807, TC396460, TC428944 |
| [Transmembrane transporter activity](http://amigo.geneontology.org/cgi-bin/amigo/go.cgi?action=query&view=query&query=GO:0022857&search_constraint=terms) | TC410352, DR739471, TC404158, TC434820, TC443387, TC379711, TC373958, TC378225, TC392272, TC429341, TC397994, TC402440, TC371242, TC423252, TC394307, TC397660, TC375146, TC404978, TC389363, TC375813, TC388914, TC379171, TC373583, TC387621, TC389589, TC446235, TC392778, TC432154, CK211589, TC420735, TC373678, TC395453, TC386422, TC388688, TC387319, TC406594, TC384071, TC390569, TC416685, TC369182, TC403872, TC389090, TC390150, TC389375, TC388822, TC406708, TC377496, TC373002, TC382655, TC429771, CK200433, TC423576, TC406038, TC416658, TC384678, TC395351, TC386646, TC392709, TC401915, TC393561, TC456619, TC435799, TC391671, TC393554, TC407614, TC459656, DR739994, TC384454, TC413571, CA729339, CK217367, TC378153, TC429374, TC379853, TC370315, TC389816, TC405440, TC384344, TC391621, TC385659, TC376933, DR737360, TC382910, TC444402, TC434831, TC396895, TC426326, TC393106, TC377749, TC398379, BQ607161, TC406807, TC396460, TC428944, TC418340 |
| [Molecular_function](http://amigo.geneontology.org/cgi-bin/amigo/go.cgi?action=query&view=query&query=GO:0003674&search_constraint=terms) | TC461921, TC410352, TC386040, TC373613, TC390944, TC407978, TC373958, TC390393, TC444546, TC416529, TC389190, TC453487, TC391946, TC375864, TC420420, TC460760, TC383139, TC415588, TC408524, BQ838511, TC417106, TC398052, TC387861, TC374098, CA710880, TC452945, TC400388, TC457112, TC421871, CK215979, CK206714, TC376351, TC394459, TC371242, TC439423, TC416154, TC384738, TC423252, TC397660, TC379171, TC388410, BJ282766, CK203550, TC416493, TC388136, TC458562, TC421914, TC417260, TC380063, TC398538, TC433844, TC418414, CJ944525, TC410063, TC378054, TC386396, TC373702, TC410954, TC448471, TC456784, TC395453, TC392329, TC409843, TC406594, CA682223, TC407076, TC445939, TC403872, TC425847, CJ727624, CA614761, CJ550278, TC411128, GH732878, TC397444, TC398304, TC379558, TC425878, TC393100, TC412569, CV771134, TC417012, CA632212, TC393523, TC455515, TC413027, TC394916, TC397793, TC394820, TC398731, TC418073, TC378274, TC411784, TC391130, TC417308, CK217367, TC378153, TC387817, TC395069, TC413460, BQ609416, TC452050, CK214702, TC396857, TC395723, TC387981, TC379536, TC385233, TC398730, TC391621, TC394087, TC389718, TC404052, TC386707, TC376248, TC429713, TC374409, TC391947, CK201148, TC381943, TC432001, TC420358, TC398379, TC422142, TC413392, TC418928, TC376758, BQ607161, TC372953, TC372845, TC382045, TC369628, TC402308, TC441343, TC400330, TC400260, TC378790, TC378432, TC377061, TC417077, TC446465, TC416801, BF474051, CV759879, TC391438, CA606693, TC370912, TC412317, TC432369, TC412212, TC373563, TC375124, TC425291, TC421345, TC405695, TC370178, TC392778, TC397312, TC405030, TC388403, CK161232, TC373678, CA615187, TC416695, TC405041, TC397019, BJ317882, TC406469, TC399919, TC407572, TC402121, TC410690, TC406708, TC374461, TC409459, TC410074, TC386646, TC416471, CV763657, TC406809, TC383270, CN010359, TC458205, TC369687, TC402603, TC441241, TC433557, TC449724, TC413571, TC372867, TC377559, TC397909, CA729339, TC383019, TC408299, BQ239045, TC389993, TC399352, TC422425, TC408312, CA613620, TC372654, TC398805, TC400362, TC369092, TC429747, TC375612, TC430501, TC395872, TC371641, TC400108, TC406106, TC399342, TC432185, TC381462, CD882425, TC377749, TC455736, TC396451, TC392263, TC431198, TC424344, TC432130, TC396529, TC373145, TC404158, TC427986, CK201269, TC425957, TC445166, TC372677, TC421162, TC429341, TC397033, TC374240, CA720842, TC376306, TC425841, TC389168, TC398121, TC424204, TC387579, TC439324, TC406870, TC413854, TC393814, BJ279521, TC376220, TC408309, TC446235, TC404926, TC402545, TC427006, TC393830, TC380943, TC376202, TC403885, TC386961, TC398606, TC387319, TC370603, TC384688, TC369182, TC387344, TC418365, TC389090, TC370347, TC389139, TC412483, TC375554, TC373002, TC382655, CK200433, TC373994, TC393970, TC374731, TC395351, TC392709, TC372563, TC418032, TC456619, CK162413, TC397291, TC411908, CA598430, TC377373, TC380329, TC415365, EB512907, TC409343, TC390994, TC382674, TC391143, TC428066, TC381463, TC419733, TC392074, TC403157, TC410066, TC382742, TC395303, TC405511, TC377190, TC443814, TC431879, TC423110, TC376606, TC422348, TC391613, TC385365, TC430544, TC401210, DR737360, TC379357, TC387135, TC397885, TC411684, TC388049, TC375760, TC381923, BJ309186, TC393960, TC426358, TC385326, TC409077, TC373251, TC443387, TC388566, TC415083, TC378225, TC399245, TC373429, TC390630, TC386535, GH729256, TC418716, CA605200, TC386519, TC369655, TC383047, TC402072, TC390402, TC370158, TC401260, TC417992, TC417341, TC394307, TC388665, TC373583, TC406371, TC389589, TC398592, TC392363, TC370106, TC381988, TC452762, TC388819, TC405475, TC386344, TC373637, TC370044, CD878039, TC435595, TC374164, TC386422, TC442623, TC418845, TC384071, TC414606, TC412150, TC388822, TC384592, TC429771, TC386639, TC416658, TC381957, TC384678, TC396650, TC379965, TC374230, TC391913, TC375834, TC393948, TC392109, TC371970, TC375726, TC400638, TC393554, TC407614, TC400477, TC459656, DR734904, TC384357, TC391007, TC379436, TC460615, TC403573, TC411941, TC440632, TC403929, CK201126, TC457991, TC378601, TC394118, TC449043, TC392247, TC379635, TC373848, TC387116, TC373259, TC387007, TC433589, TC398343, TC418091, TC369664, TC389816, TC371387, TC372530, TC392323, TC384194, TC413066, TC371172, TC446092, TC439472, TC433574, TC372167, TC391641, TC461622, TC400056, TC379470, TC403588, TC381817, TC385659, TC376933, TC375798, TC382910, TC389451, TC394728, DR740372, TC403803, TC391122, TC440526, TC418850, TC401758, TC428944, TC417067, TC389661, TC381279, TC396657, TC379942, TC397258, TC426838, CV775873, TC374404, TC396230, TC377766, TC451511, TC394965, TC384373, TC427210, TC416069, TC375313, TC398862, TC433162, DR739303, TC404978, TC389363, TC434442, TC413263, TC380590, TC416442, TC378878, TC387410, TC406264, TC378072, TC397176, TC390792, TC406516, TC388950, TC419222, TC448840, TC410194, TC392875, TC435546, TC371145, TC384735, TC390569, TC405540, TC439904, TC419057, TC391948, BQ294582, TC398714, TC373791, TC381619, TC440693, TC394028, TC398970, TC457126, TC390904, TC403679, TC403580, TC413427, TC401915, TC424044, TC433957, TC423374, TC421954, TC405652, TC385710, TC423239, TC425821, DR739994, TC459193, TC377021, TC394206, TC406236, TC382080, TC409599, TC391128, TC391962, TC445767, TC389092, TC446038, TC423804, TC379853, TC413199, CK199846, TC370315, TC449504, TC418685, TC405440, TC403968, CK213497, TC460795, CJ792862, TC414899, TC370885, TC422789, TC436347, TC381561, TC391742, TC440819, TC422691, TC398514, TC416492, TC377225, TC392033, TC412520, TC403977, TC406807, CD892838, TC395090, TC404606, CA646741, TC416906, TC434820, TC379711, TC393198, TC416438, TC377308, TC379903, CA595837, TC406749, TC423265, TC392272, TC372744, TC401244, DR739350, CA730421, TC417388, TC440066, TC386313, TC413339, CK208222, TC375539, TC408907, TC369844, TC420579, CK211707, TC386963, CK163367, TC412732, TC431201, CA700201, TC396751, TC370350, CK211589, TC411116, TC383909, TC376527, TC388718, TC419584, TC383701, TC390373, TC378568, TC385780, TC374392, TC405784, TC372175, TC382734, TC390150, TC369199, TC419747, TC411350, TC402186, TC405896, TC405356, TC377441, TC403264, TC423354, TC369348, TC413043, CK214224, TC368603, TC398040, CA709177, TC376263, BE585841, TC429374, TC409187, TC384010, TC416169, TC425690, TC423182, TC381218, TC409043, TC408229, TC409190, TC383677, TC435224, TC384344, TC392297, CV781430, TC388520, TC379422, TC411191, TC387683, TC434831, TC426326, TC375431, TC391995, TC422841, TC387064, TC373914, TC450285, TC392303, TC369899, TC378846, TC394796, TC372330, TC420057, TC440499, TC397286, TC381068, CA611770, TC435533, TC395966, TC397994, CF554444, TC414243, TC451276, TC403234, TC415685, CJ692089, TC371738, TC388914, TC387621, TC377438, TC368548, TC398633, TC394716, TC369633, TC460689, TC410126, TC380125, TC378271, TC369064, TC432154, TC380433, TC420735, TC435281, TC422922, TC404371, TC427405, TC373615, TC449256, TC384219, TC397562, TC416685, TC372664, TC389375, TC404413, TC370114, TC406038, TC390319, TC408192, TC376874, TC424154, TC458807, TC459245, CK210754, TC369928, TC370727, TC417769, TC410147, TC442100, TC376490, TC384454, TC411287, TC386237, TC393904, TC388751, TC387191, TC447801, TC399471, TC380416, TC427981, TC434396, CV780698, TC398536, TC421880, TC386414, TC400755, CJ854725, TC403986, TC372701, TC440636, TC406193, TC405615, TC376420, TC458987, TC383763, TC404843, TC384553, CD876572, TC434140, TC382737, TC370633, TC369736, TC390135, TC386279, TC424376, TC376774, TC411480, TC373129, TC420394, CK197833, TC432320, TC391785, TC409650, TC382786, TC380662, TC388649, TC419727, TC388158, TC430561, TC418340, TC395298, TC405773, TC389162, TC382830, DR739471, CD905784, TC402534, CK212850, TC371600, TC399432, TC390285, TC369726, TC398026, TC407340, TC387766, TC406584, TC405295, TC402440, TC455676, TC391411, TC409208, TC375146, TC432205, TC375813, TC454407, TC384122, TC394661, TC381963, TC383176, TC430821, TC371455, TC449463, TC371037, TC374879, TC385701, TC403328, TC388688, TC439939, TC379459, TC375268, TC400181, TC452503, TC388547, CK207939, TC396365, TC377496, TC368549, TC388976, TC379069, TC401158, TC387133, TC410078, TC423576, TC385526, TC397500, TC375530, TC368591, TC402668, TC405079, TC393561, TC435799, TC393692, TC386322, TC391671, TC373787, TC388691, TC382928, TC397415, TC369807, TC438243, TC375918, TC417363, TC396487, TC387710, TC413700, TC385515, TC380882, TC423880, TC440321, TC451285, TC372580, TC458503, TC377410, TC396083, TC371073, TC393820, TC451519, CK211469, CB307332, TC437163, CJ930688, TC404842, TC435808, BE586004, TC397729, TC399408, TC444402, TC411817, TC426743, TC436290, TC396895, TC382342, TC393106, TC390489, TC407183, TC432504, TC431306, TC396460, TC397676, TC399124 |
| [Transferase activity, transferring alkyl or aryl (other than methyl) groups](http://amigo.geneontology.org/cgi-bin/amigo/go.cgi?action=query&view=query&query=GO:0016765&search_constraint=terms) | TC427986, TC378846, TC371600, TC405652, TC376202, TC369687, TC400477, CK211589, TC388691, TC440499, DR734904, TC413571, TC397019, TC397793, TC396487, CK197833, TC439324, TC439423, TC374461, TC392074, TC390319, TC419727, CK199846 |
| [Substrate-specific transmembrane transporter activity](http://amigo.geneontology.org/cgi-bin/amigo/go.cgi?action=query&view=query&query=GO:0022891&search_constraint=terms) | TC410352, DR739471, TC404158, TC434820, TC443387, TC379711, TC373958, TC392272, TC429341, TC397994, TC402440, TC371242, TC423252, TC394307, TC397660, TC375146, TC404978, TC389363, TC388914, TC379171, TC373583, TC387621, TC389589, TC446235, TC392778, TC432154, CK211589, TC420735, TC373678, TC395453, TC386422, TC388688, TC387319, TC390569, TC369182, TC416685, TC403872, TC389090, TC390150, TC389375, TC388822, TC406708, TC373002, TC377496, TC382655, TC429771, CK200433, TC423576, TC406038, TC416658, TC384678, TC395351, TC386646, TC392709, TC401915, TC393561, TC456619, TC435799, TC391671, TC393554, TC407614, TC459656, DR739994, TC384454, TC413571, CA729339, CK217367, TC378153, TC429374, TC379853, TC370315, TC389816, TC405440, TC384344, TC391621, TC376933, DR737360, TC382910, TC444402, TC396895, TC434831, TC426326, TC393106, BQ607161, TC406807, TC396460, TC428944 |
| [Substrate-specific transporter activity](http://amigo.geneontology.org/cgi-bin/amigo/go.cgi?action=query&view=query&query=GO:0022892&search_constraint=terms) | TC410352, DR739471, TC404158, TC434820, TC443387, TC379711, TC373958, TC378790, TC392272, TC429341, TC383047, TC397994, TC402440, TC371242, TC423252, TC394307, TC397660, TC375146, TC404978, TC389363, TC388914, TC379171, TC373583, TC387621, TC389589, TC446235, TC392778, TC432154, CK211589, TC420735, TC373678, TC395453, TC386422, TC388688, TC387319, TC390569, TC369182, TC416685, TC403872, TC389090, TC390150, TC389375, TC388822, TC406708, TC377496, TC373002, TC382655, TC429771, CK200433, TC423576, TC406038, TC416658, TC384678, TC395351, TC386646, TC392709, TC401915, TC393561, TC456619, TC435799, TC386322, TC391671, TC412569, TC393554, TC407614, TC459656, DR739994, TC438243, TC384454, TC413571, CA729339, TC375918, TC409343, TC411784, CK217367, TC378153, TC429374, TC425690, TC379853, TC398343, TC370315, TC389816, TC405440, TC446092, TC384344, TC435808, TC391621, TC391742, TC376933, DR737360, TC382910, TC444402, TC434831, TC396895, TC426326, TC393106, TC418928, TC455736, BQ607161, TC406807, TC396460, TC428944 |
| [Lipid binding](http://amigo.geneontology.org/cgi-bin/amigo/go.cgi?action=query&view=query&query=GO:0008289&search_constraint=terms) | TC398304, TC393100, TC433957, CK210754, TC459656, TC382928, CV775873, TC441241, TC418716, TC417363, TC386237, TC394916, TC386313, TC390994, TC398052, TC425841, TC378274, TC403929, TC387579, CA606693, TC394459, TC371242, TC384738, CK211707, TC386963, TC388410, TC395303, TC422425, TC458562, TC400755, TC418685, TC458987, TC396751, CA615187, TC386961, TC398514, TC390135, TC377225, TC411191, TC452503, TC388547, TC387344, TC407076, TC405784, TC399919, TC407572, TC372175, TC396365, TC419747, TC380662, TC401758, TC398714, TC406807, TC389661 |
| [Transferase activity, transferring phosphorus-containing groups](http://amigo.geneontology.org/cgi-bin/amigo/go.cgi?action=query&view=query&query=GO:0016772&search_constraint=terms) | CA646741, TC409077, TC369899, TC394796, TC416438, TC399245, TC391946, DR739350, TC407340, TC394965, TC374240, TC413339, TC375313, TC369655, TC395966, TC370912, BJ279521, TC398592, TC397176, TC394661, TC435595, TC374164, TC405041, TC384688, TC407076, TC368549, TC370114, TC397500, CJ550278, TC405896, TC381957, TC379965, TC372563, TC374230, TC375726, TC459656, TC449724, TC397909, TC411908, EB512907, TC411941, TC388751, TC457991, TC409187, TC389993, TC398536, TC370315, CJ854725, TC395723, TC371172, TC383677, TC376420, TC372167, TC443814, TC393820, TC437163, TC384553, TC430544, TC394087, TC370633, TC429747, TC379422, TC430501, TC392033, TC387683, TC406807, CD892838, TC395090, TC382045 |
| [Aldo-keto reductase activity](http://amigo.geneontology.org/cgi-bin/amigo/go.cgi?action=query&view=query&query=GO:0004033&search_constraint=terms) | TC403573, TC422142, TC433574, TC376306, TC387981, TC388158, TC375554 |
| [Binding](http://amigo.geneontology.org/cgi-bin/amigo/go.cgi?action=query&view=query&query=GO:0005488&search_constraint=terms) | TC461921, TC410352, TC386040, TC373613, TC407978, TC390393, TC416529, TC397258, TC389190, CV775873, TC391946, TC396230, TC460760, TC451511, TC394965, TC415588, TC408524, TC427210, TC416069, BQ838511, TC398052, TC375313, TC387861, CA710880, TC400388, TC398862, TC433162, TC376351, TC394459, TC371242, TC416154, TC384738, TC423252, TC397660, TC389363, TC379171, TC388410, CK203550, TC416493, TC388136, TC458562, TC421914, TC406264, TC387410, TC378878, TC380063, TC398538, TC397176, TC418414, CJ944525, TC410063, TC378054, TC386396, TC373702, TC448471, TC395453, TC410194, TC392875, TC392329, TC409843, TC435546, TC384735, TC390569, TC405540, CA682223, TC407076, TC419057, TC403872, TC425847, CJ727624, TC398714, BQ294582, CA614761, CJ550278, TC394028, TC457126, TC413427, TC403580, TC411128, TC401915, TC397444, TC398304, TC425878, TC423374, TC393100, TC433957, CV771134, TC417012, TC425821, TC423239, CA632212, DR739994, TC459193, TC455515, TC394916, TC394206, TC394820, TC398731, TC406236, TC418073, TC378274, TC391130, TC417308, CK217367, TC391128, TC409599, TC445767, TC389092, TC446038, TC423804, TC413460, TC379853, TC370315, BQ609416, CK214702, TC449504, TC418685, TC395723, TC405440, TC403968, CK213497, CJ792862, TC379536, TC385233, TC391742, TC440819, TC389718, TC404052, TC398514, TC377225, TC392033, CK201148, TC391947, TC381943, TC412520, TC432001, TC398379, TC413392, BQ607161, TC406807, CD892838, TC372953, TC395090, TC382045, TC404606, CA646741, TC369628, TC434820, TC416906, TC402308, TC441343, TC379711, TC400330, TC378790, CA595837, TC379903, TC377308, TC423265, TC392272, TC377061, TC401244, CA730421, TC417077, TC417388, TC446465, TC386313, CV759879, TC413339, CK208222, TC408907, CA606693, TC391438, CK211707, TC386963, CK163367, TC412732, TC421345, TC405695, CA700201, TC392778, TC396751, TC397312, TC405030, CK211589, TC388403, TC383909, TC376527, CA615187, TC416695, TC388718, TC405041, TC419584, TC383701, BJ317882, TC390373, TC406469, TC385780, TC374392, TC405784, TC399919, TC407572, TC372175, TC402121, TC369199, TC410690, TC406708, TC419747, TC409459, TC410074, TC402186, TC386646, TC416471, TC405356, CV763657, TC383270, TC377441, TC458205, TC423354, TC402603, TC441241, TC449724, TC413571, TC397909, TC413043, CA729339, CK214224, TC408299, BQ239045, CA709177, BE585841, TC376263, TC409187, TC384010, TC389993, TC416169, TC425690, TC381218, TC399352, TC409043, TC408229, TC422425, TC408312, CA613620, TC398805, TC400362, TC369092, CV781430, TC388520, TC429747, TC411191, TC371641, TC387683, TC399342, TC434831, TC432185, TC426326, TC381462, TC375431, CD882425, TC387064, TC373914, TC392263, TC396529, TC373145, TC392303, TC394796, TC420057, TC425957, TC445166, TC372677, CA611770, TC435533, TC374240, CA720842, TC425841, TC397994, CF554444, TC424204, TC387579, TC406870, TC415685, TC393814, TC388914, TC376220, TC387621, TC368548, TC394716, TC398633, TC369633, TC446235, TC460689, TC402545, TC378271, TC380943, TC393830, TC432154, TC380433, TC403885, TC420735, TC404371, TC386961, TC373615, TC384219, TC397562, TC416685, TC384688, TC387344, TC372664, TC389090, TC412483, TC389375, TC404413, TC382655, CK200433, TC370114, TC373994, TC406038, TC393970, TC395351, TC372563, TC459245, TC418032, TC456619, CK210754, TC410147, TC376490, TC384454, TC411908, TC411287, TC380329, TC386237, TC393904, TC390994, TC391143, TC428066, TC381463, TC403157, TC382742, TC398536, TC395303, TC386414, TC400755, CJ854725, TC403986, TC405511, TC440636, TC406193, TC405615, TC376420, TC458987, TC377190, TC443814, TC431879, TC423110, TC404843, TC422348, TC384553, CD876572, TC385365, TC430544, TC370633, TC401210, DR737360, TC379357, TC390135, TC387135, TC386279, TC397885, TC411684, TC411480, TC388049, TC373129, TC420394, TC409650, TC380662, TC393960, TC426358, TC389162, DR739471, TC402534, TC443387, TC388566, TC415083, TC373429, TC390630, TC386535, GH729256, TC369726, TC418716, TC407340, TC406584, TC386519, CA605200, TC369655, TC390402, TC402440, TC391411, TC417992, TC417341, TC394307, TC375146, TC388665, TC432205, TC375813, TC389589, TC381988, TC452762, TC386344, TC394661, TC373637, TC381963, TC383176, TC370044, CD878039, TC371455, TC449463, TC435595, TC374879, TC386422, TC374164, TC388688, TC452503, TC414606, TC388547, TC388822, TC396365, TC377496, TC368549, TC388976, TC429771, TC410078, TC423576, TC386639, TC397500, TC375530, TC416658, TC368591, TC396650, TC379965, TC381957, TC405079, TC374230, TC393561, TC375834, TC435799, TC393948, TC393692, TC375726, TC391671, TC393554, TC407614, TC382928, TC459656, TC369807, TC438243, TC379436, TC417363, TC460615, TC403573, TC411941, CK201126, TC457991, TC387710, TC413700, TC403929, TC394118, TC449043, TC380882, TC423880, TC440321, TC379635, TC387007, TC373259, TC433589, TC377410, TC389816, TC372530, TC392323, TC413066, TC384194, TC439472, TC372167, TC371073, TC461622, TC393820, TC400056, CK211469, CB307332, TC404842, TC435808, TC381817, TC403588, TC375798, TC397729, TC394728, DR740372, TC403803, TC391122, TC396895, TC382342, TC418850, TC393106, TC401758, TC432504, TC396460, TC397676, TC428944, TC389661, TC417067 |
| [Transporter activity](http://amigo.geneontology.org/cgi-bin/amigo/go.cgi?action=query&view=query&query=GO:0005215&search_constraint=terms) | TC410352, DR739471, TC404158, TC434820, TC443387, TC379711, TC373958, CK201269, TC378790, TC378225, TC390630, TC392272, TC429341, TC383047, TC397994, TC402440, TC371242, TC423252, TC394307, TC397660, TC375146, TC412317, TC404978, TC389363, TC375813, TC388914, TC379171, TC373583, TC412212, TC387621, TC389589, TC408309, TC446235, TC392778, TC432154, TC386396, CK211589, TC420735, TC383909, TC449463, TC373678, TC395453, TC386422, TC388688, TC387319, TC406594, TC442623, TC397562, TC384071, TC390569, TC416685, TC369182, TC403872, TC418365, TC389090, TC390150, TC389375, TC388822, TC406708, TC377496, TC373002, TC382655, TC429771, CK200433, TC423576, TC406038, TC416658, TC384678, TC395351, TC386646, TC392709, TC401915, TC393561, TC456619, TC435799, TC393948, TC386322, TC391671, TC412569, TC393554, TC407614, TC459656, TC438243, DR739994, TC384454, TC413571, TC375918, CA729339, TC409343, TC411784, TC385515, CK217367, TC378153, TC429374, TC425690, TC379853, TC398343, TC370315, TC389816, TC405440, TC446092, TC384344, TC435808, TC391621, TC391742, TC385659, TC376933, DR737360, TC382910, TC387135, TC444402, TC434831, TC396895, TC426326, TC393106, TC377749, TC418928, TC398379, TC455736, BQ607161, TC406807, TC431306, TC396460, TC428944, TC418340 |
| [Histone kinase activity](http://amigo.geneontology.org/cgi-bin/amigo/go.cgi?action=query&view=query&query=GO:0035173&search_constraint=terms) | TC374230, TC394796, TC407340, TC382045 |
| [S-methyltransferase activity](http://amigo.geneontology.org/cgi-bin/amigo/go.cgi?action=query&view=query&query=GO:0008172&search_constraint=terms) | TC380590, TC413854, TC417106, TC402072 |
| [Sugar:hydrogen symporter activity](http://amigo.geneontology.org/cgi-bin/amigo/go.cgi?action=query&view=query&query=GO:0005351&search_constraint=terms) | TC429374, TC392709, TC369182, TC373678, TC373958, TC378153 |
| [Cation:sugar symporter activity](http://amigo.geneontology.org/cgi-bin/amigo/go.cgi?action=query&view=query&query=GO:0005402&search_constraint=terms) | TC429374, TC392709, TC369182, TC373678, TC373958, TC378153 |
| [dUTP diphosphatase activity](http://amigo.geneontology.org/cgi-bin/amigo/go.cgi?action=query&view=query&query=GO:0004170&search_constraint=terms) | GH732878, TC454407, TC457112 |
| [Nucleoside-triphosphate diphosphatase activity](http://amigo.geneontology.org/cgi-bin/amigo/go.cgi?action=query&view=query&query=GO:0047429&search_constraint=terms) | GH732878, TC454407, TC457112 |
| [Kinase activity](http://amigo.geneontology.org/cgi-bin/amigo/go.cgi?action=query&view=query&query=GO:0016301&search_constraint=terms) | CA646741, TC374230, TC372563, TC394796, TC375726, TC416438, TC459656, TC391946, DR739350, TC449724, TC397909, TC407340, TC411908, TC394965, TC374240, TC413339, TC388751, TC411941, TC369655, TC457991, TC409187, TC389993, TC370912, BJ279521, TC398536, TC370315, TC398592, CJ854725, TC395723, TC371172, TC376420, TC372167, TC393820, TC394661, TC437163, TC384553, TC430544, TC394087, TC435595, TC370633, TC379422, TC405041, TC429747, TC392033, TC384688, TC387683, TC407076, TC370114, TC406807, CJ550278, CD892838, TC405896, TC395090, TC379965, TC381957, TC382045 |
| [Two-component response regulator activity](http://amigo.geneontology.org/cgi-bin/amigo/go.cgi?action=query&view=query&query=GO:0000156&search_constraint=terms) | TC392329, TC403968, TC420579, TC405041, TC380329 |
| [Amidase activity](http://amigo.geneontology.org/cgi-bin/amigo/go.cgi?action=query&view=query&query=GO:0004040&search_constraint=terms) | TC406106, TC383139 |
| [High affinity copper ion transmembrane transporter activity](http://amigo.geneontology.org/cgi-bin/amigo/go.cgi?action=query&view=query&query=GO:0015089&search_constraint=terms) | TC429341, TC444402 |
| [Flavonol synthase activity](http://amigo.geneontology.org/cgi-bin/amigo/go.cgi?action=query&view=query&query=GO:0045431&search_constraint=terms) | TC430821, TC413199 |
| [Chitin binding](http://amigo.geneontology.org/cgi-bin/amigo/go.cgi?action=query&view=query&query=GO:0008061&search_constraint=terms) | TC405079, TC384010, TC369807, DR740372, CK201126, CK201148, TC369199, TC377308 |
| [Solute:hydrogen symporter activity](http://amigo.geneontology.org/cgi-bin/amigo/go.cgi?action=query&view=query&query=GO:0015295&search_constraint=terms) | TC429374, TC392709, TC369182, TC373678, TC373958, TC378153 |
| [Sugar transmembrane Transporter activity](http://amigo.geneontology.org/cgi-bin/amigo/go.cgi?action=query&view=query&query=GO:0051119&search_constraint=terms) | TC429374, TC392709, TC369182, TC373678, TC373958, TC378153 |
| [Oxidoreductase activity, acting on paired donors, with incorporation or reduction of molecular oxygen, 2-oxoglutarate as one donor, and incorporation of one atom each of oxygen into both donors](http://amigo.geneontology.org/cgi-bin/amigo/go.cgi?action=query&view=query&query=GO:0016706&search_constraint=terms) | TC430821, TC413199, TC381923, TC384122, TC385710 |
| [Homocysteine S-methyltransferase activity](http://amigo.geneontology.org/cgi-bin/amigo/go.cgi?action=query&view=query&query=GO:0008898&search_constraint=terms) | TC380590, TC413854, TC402072 |
| [Transcription corepressor activity](http://amigo.geneontology.org/cgi-bin/amigo/go.cgi?action=query&view=query&query=GO:0003714&search_constraint=terms) | TC460615, TC400362, TC459656, TC370114, TC406807, TC393960, TC404413 |
| [Sulfate adenylyltransferase activity](http://amigo.geneontology.org/cgi-bin/amigo/go.cgi?action=query&view=query&query=GO:0004779&search_constraint=terms) | TC399245, TC369899 |
| [Sulfate adenylyltransferase (ATP) activity](http://amigo.geneontology.org/cgi-bin/amigo/go.cgi?action=query&view=query&query=GO:0004781&search_constraint=terms) | TC399245, TC369899 |
| [Carbohydrate transmembrane transporter activity](http://amigo.geneontology.org/cgi-bin/amigo/go.cgi?action=query&view=query&query=GO:0015144&search_constraint=terms) | TC429374, TC392709, TC369182, TC373678, TC373958, TC378153 |
| [Phosphofructokinase activity](http://amigo.geneontology.org/cgi-bin/amigo/go.cgi?action=query&view=query&query=GO:0008443&search_constraint=terms) | TC388751, TC416438, TC379422 |
| [Phosphatidyltransferase activity](http://amigo.geneontology.org/cgi-bin/amigo/go.cgi?action=query&view=query&query=GO:0030572&search_constraint=terms) | TC430501, TC395966 |
| [Pattern binding](http://amigo.geneontology.org/cgi-bin/amigo/go.cgi?action=query&view=query&query=GO:0001871&search_constraint=terms) | TC405079, TC384010, TC369807, DR740372, CK201126, CK201148, TC369199, TC377308 |
| [Polysaccharide binding](http://amigo.geneontology.org/cgi-bin/amigo/go.cgi?action=query&view=query&query=GO:0030247&search_constraint=terms) | TC405079, TC384010, TC369807, DR740372, CK201126, CK201148, TC369199, TC377308 |
| [Catalytic activity](http://amigo.geneontology.org/cgi-bin/amigo/go.cgi?action=query&view=query&query=GO:0003824&search_constraint=terms) | TC410352, TC373613, TC426838, TC453487, TC391946, TC375864, TC377766, TC420420, TC383139, TC394965, TC384373, TC417106, TC375313, CA710880, TC452945, TC457112, TC376351, TC371242, TC439423, TC423252, DR739303, TC397660, TC389363, TC434442, TC388136, TC413263, TC380590, TC387410, TC378072, TC433844, TC397176, TC388950, CJ944525, TC373702, TC410954, TC456784, TC395453, TC448840, TC409843, TC371145, TC384735, TC390569, TC407076, TC445939, TC403872, TC373791, CJ550278, TC394028, TC401915, GH732878, TC405652, TC385710, TC393523, DR739994, TC455515, TC397793, TC394820, TC382080, CK217367, TC391962, TC391128, TC387817, TC395069, TC379853, TC413199, CK199846, TC370315, TC452050, TC395723, TC405440, TC414899, TC370885, TC387981, TC436347, TC394087, TC386707, TC398514, TC376248, TC392033, TC429713, TC374409, CK201148, TC398379, TC422142, TC418928, TC376758, BQ607161, TC406807, CD892838, TC395090, TC372845, TC382045, CA646741, TC434820, TC441343, TC379711, TC393198, TC416438, TC378790, TC377308, TC406749, TC392272, TC378432, DR739350, TC417077, TC413339, TC375539, TC369844, TC370912, TC432369, TC375124, TC425291, TC392778, TC370350, CK211589, CK161232, TC405041, TC397019, TC382734, TC369199, TC406708, TC374461, TC409459, TC405896, TC386646, TC416471, TC383270, CN010359, TC369687, TC423354, TC369348, TC449724, TC413571, TC372867, TC397909, CA729339, BE585841, TC409187, TC384010, TC389993, TC425690, TC399352, TC409190, TC383677, TC372654, TC400362, TC392297, TC369092, TC379422, TC429747, TC430501, TC387683, TC406106, TC434831, TC432185, TC426326, TC391995, TC377749, TC387064, TC422841, TC431198, TC373145, TC427986, TC369899, TC378846, TC394796, TC372330, TC440499, TC425957, TC445166, TC381068, TC421162, TC397033, TC374240, TC376306, TC395966, TC398121, TC397994, TC439324, TC403234, TC413854, TC388914, BJ279521, TC377438, TC368548, TC446235, TC380125, TC427006, TC369064, TC376202, TC380433, TC420735, TC427405, TC397562, TC416685, TC384688, TC372664, TC389090, TC370347, TC389375, TC404413, TC375554, TC382655, CK200433, TC370114, TC406038, TC390319, TC393970, TC408192, TC424154, TC395351, TC372563, TC369928, CK162413, TC442100, TC376490, TC384454, TC411908, TC377373, EB512907, TC388751, TC382674, TC447801, TC380416, TC427981, TC392074, CV780698, TC434396, TC398536, TC421880, CJ854725, TC372701, TC405511, TC376420, TC377190, TC443814, TC404843, TC376606, TC384553, TC430544, TC370633, TC369736, DR737360, TC424376, TC376774, TC388049, CK197833, TC375760, TC381923, TC382786, TC409650, TC419727, TC388158, TC393960, TC385326, TC405773, TC389162, TC409077, TC382830, DR739471, TC373251, TC443387, CK212850, TC371600, TC378225, TC399245, TC390630, TC390285, TC398026, TC407340, TC387766, TC405295, TC369655, TC402072, TC402440, TC370158, TC417341, TC394307, TC375146, TC375813, TC454407, TC398592, TC389589, TC392363, TC381988, TC370106, TC388819, TC384122, TC394661, TC430821, TC435595, TC371037, TC386422, TC374164, TC385701, TC388688, TC439939, TC384071, TC418845, TC442623, TC375268, TC400181, CK207939, TC388822, TC377496, TC368549, TC401158, TC429771, TC410078, TC385526, TC397500, TC416658, TC379965, TC396650, TC402668, TC381957, TC374230, TC393561, TC375834, TC393948, TC392109, TC386322, TC375726, TC391671, TC393554, TC407614, TC400477, TC373787, TC388691, TC459656, DR734904, TC384357, TC403573, TC396487, TC411941, TC440632, TC457991, TC378601, TC380882, TC423880, TC392247, TC387116, TC372580, TC387007, TC369664, TC396083, TC389816, TC372530, TC384194, TC371172, TC446092, TC433574, TC372167, TC400056, TC393820, TC451519, TC437163, CJ930688, TC379470, TC381817, TC403588, TC399408, TC394728, DR740372, TC396895, TC440526, TC393106, TC396460, TC428944, TC381279, TC396657 |
| [Endochitinase activity](http://amigo.geneontology.org/cgi-bin/amigo/go.cgi?action=query&view=query&query=GO:0008843&search_constraint=terms) | TC384010, DR740372, CK201148, TC369199, TC377308 |
| [Amino acid transmembrane transporter activity](http://amigo.geneontology.org/cgi-bin/amigo/go.cgi?action=query&view=query&query=GO:0015171&search_constraint=terms) | TC382910, TC404158, TC391621, TC404978, TC379171, TC384678 |
| [Phosphotransferase activity, alcohol group as acceptor](http://amigo.geneontology.org/cgi-bin/amigo/go.cgi?action=query&view=query&query=GO:0016773&search_constraint=terms) | CA646741, TC374230, TC394796, TC416438, TC459656, DR739350, TC407340, TC394965, TC413339, TC374240, TC388751, TC411941, TC369655, TC457991, TC370912, BJ279521, TC370315, TC398592, TC395723, TC371172, TC376420, TC372167, TC437163, TC394087, TC435595, TC429747, TC405041, TC379422, TC387683, TC384688, TC407076, TC370114, TC406807, CJ550278, CD892838, TC395090, TC381957, TC382045 |
| [Citrate (Si)-synthase activity](http://amigo.geneontology.org/cgi-bin/amigo/go.cgi?action=query&view=query&query=GO:0004108&search_constraint=terms) | TC374230, TC407340, TC382045 |
| [Enoyl-[acyl-carrier-protein] reductase (NADH) activity](http://amigo.geneontology.org/cgi-bin/amigo/go.cgi?action=query&view=query&query=GO:0004318&search_constraint=terms) | TC371037, TC398121 |
| [Enoyl-[acyl-carrier-protein] reductase activity](http://amigo.geneontology.org/cgi-bin/amigo/go.cgi?action=query&view=query&query=GO:0016631&search_constraint=terms) | TC371037, TC398121 |
| [Symporter activity](http://amigo.geneontology.org/cgi-bin/amigo/go.cgi?action=query&view=query&query=GO:0015293&search_constraint=terms) | TC429374, TC392709, TC369182, TC373678, TC384344, TC373958, TC378153 |
| [Solute:cation symporter activity](http://amigo.geneontology.org/cgi-bin/amigo/go.cgi?action=query&view=query&query=GO:0015294&search_constraint=terms) | TC429374, TC392709, TC369182, TC373678, TC384344, TC373958, TC378153 |
| [Transferase activity, transferring acyl groups, acyl groups converted into alkyl on transfer](http://amigo.geneontology.org/cgi-bin/amigo/go.cgi?action=query&view=query&query=GO:0046912&search_constraint=terms) | TC374230, TC407340, TC369928, TC382045 |
| [DNA clamp loader activity](http://amigo.geneontology.org/cgi-bin/amigo/go.cgi?action=query&view=query&query=GO:0003689&search_constraint=terms) | TC389162, TC374164 |
| [Xylose isomerase activity](http://amigo.geneontology.org/cgi-bin/amigo/go.cgi?action=query&view=query&query=GO:0009045&search_constraint=terms) | TC376248, TC372654 |
| [Protein-DNA loading ATPase activity](http://amigo.geneontology.org/cgi-bin/amigo/go.cgi?action=query&view=query&query=GO:0033170&search_constraint=terms) | TC389162, TC374164 |
| [6-phosphofructokinase activity](http://amigo.geneontology.org/cgi-bin/amigo/go.cgi?action=query&view=query&query=GO:0003872&search_constraint=terms) | TC388751, TC379422 |
| [Transmembrane receptor protein tyrosine phosphatase activity](http://amigo.geneontology.org/cgi-bin/amigo/go.cgi?action=query&view=query&query=GO:0005001&search_constraint=terms) | TC459656, TC406807 |
| [Copper ion transmembrane transporter activity](http://amigo.geneontology.org/cgi-bin/amigo/go.cgi?action=query&view=query&query=GO:0005375&search_constraint=terms) | TC429341, TC444402 |
| [Transmembrane receptor protein phosphatase activity](http://amigo.geneontology.org/cgi-bin/amigo/go.cgi?action=query&view=query&query=GO:0019198&search_constraint=terms) | TC459656, TC406807 |
| [DNA polymerase processivity factor activity](http://amigo.geneontology.org/cgi-bin/amigo/go.cgi?action=query&view=query&query=GO:0030337&search_constraint=terms) | CK208222, TC377061 |
| [Amine transmembrane transporter activity](http://amigo.geneontology.org/cgi-bin/amigo/go.cgi?action=query&view=query&query=GO:0005275&search_constraint=terms) | TC382910, TC404158, TC391621, TC404978, TC379171, TC384678 |
| [Oxidoreductase activity, acting on CH or CH2 groups](http://amigo.geneontology.org/cgi-bin/amigo/go.cgi?action=query&view=query&query=GO:0016725&search_constraint=terms) | TC409459, TC370114, TC392247 |
| [Protein kinase activity](http://amigo.geneontology.org/cgi-bin/amigo/go.cgi?action=query&view=query&query=GO:0004672&search_constraint=terms) | CA646741, TC374230, TC395723, TC376420, TC372167, TC394796, TC437163, TC459656, TC394087, TC435595, TC407340, TC394965, TC405041, TC429747, TC413339, TC374240, TC411941, TC369655, TC384688, TC387683, TC407076, TC457991, TC370912, TC370114, TC406807, CD892838, CJ550278, TC395090, TC381957, TC370315, TC382045, TC398592 |
| [Phosphotransferase activity, for other substituted phosphate groups](http://amigo.geneontology.org/cgi-bin/amigo/go.cgi?action=query&view=query&query=GO:0016780&search_constraint=terms) | TC430501, TC395966 |
| [Cyclin-dependent protein kinase activity](http://amigo.geneontology.org/cgi-bin/amigo/go.cgi?action=query&view=query&query=GO:0004693&search_constraint=terms) | TC374230, TC407340, TC382045 |
| [Xanthine dehydrogenase activity](http://amigo.geneontology.org/cgi-bin/amigo/go.cgi?action=query&view=query&query=GO:0004854&search_constraint=terms) | TC409459, TC370114 |
| [Xanthine oxidase activity](http://amigo.geneontology.org/cgi-bin/amigo/go.cgi?action=query&view=query&query=GO:0004855&search_constraint=terms) | TC409459, TC370114 |
| [Oxidoreductase activity, acting on CH or CH2 groups, oxygen as acceptor](http://amigo.geneontology.org/cgi-bin/amigo/go.cgi?action=query&view=query&query=GO:0016727&search_constraint=terms) | TC409459, TC370114 |
| [(S)-2-hydroxy-acid oxidase activity](http://amigo.geneontology.org/cgi-bin/amigo/go.cgi?action=query&view=query&query=GO:0003973&search_constraint=terms) | TC392363, TC384194, TC387766 |
| [Glycolate oxidase activity](http://amigo.geneontology.org/cgi-bin/amigo/go.cgi?action=query&view=query&query=GO:0008891&search_constraint=terms) | TC392363, TC384194, TC387766 |
| [Oxidoreductase activity, acting on the CH-OH group of donors, oxygen as acceptor](http://amigo.geneontology.org/cgi-bin/amigo/go.cgi?action=query&view=query&query=GO:0016899&search_constraint=terms) | TC392363, TC384194, TC387766 |
| [Fatty acid synthase activity](http://amigo.geneontology.org/cgi-bin/amigo/go.cgi?action=query&view=query&query=GO:0004312&search_constraint=terms) | TC378072, TC371037, TC398121 |
| [Inorganic anion transmembrane transporter activity](http://amigo.geneontology.org/cgi-bin/amigo/go.cgi?action=query&view=query&query=GO:0015103&search_constraint=terms) | TC432154, TC387319, TC373958 |
| [Transcription cofactor activity](http://amigo.geneontology.org/cgi-bin/amigo/go.cgi?action=query&view=query&query=GO:0003712&search_constraint=terms) | TC460615, TC373129, TC394118, TC404413, TC400362, TC370114, TC459656, TC406807, TC393960 |
| [Nucleotidyltransferase activity](http://amigo.geneontology.org/cgi-bin/amigo/go.cgi?action=query&view=query&query=GO:0016779&search_constraint=terms) | EB512907, TC375313, TC383677, TC369899, TC443814, TC397176, TC368549, TC399245, TC397500, TC374164 |
| [UDP-glycosyltransferase activity](http://amigo.geneontology.org/cgi-bin/amigo/go.cgi?action=query&view=query&query=GO:0008194&search_constraint=terms) | TC439939, TC368548, TC393948, TC370347, TC403234, TC379470, TC390630, TC376758, TC369736 |
| [Two-component sensor activity](http://amigo.geneontology.org/cgi-bin/amigo/go.cgi?action=query&view=query&query=GO:0000155&search_constraint=terms) | TC413339, TC405041 |
| [Phosphomannomutase activity](http://amigo.geneontology.org/cgi-bin/amigo/go.cgi?action=query&view=query&query=GO:0004615&search_constraint=terms) | TC370885, TC372330 |
| [Oxidoreductase activity, acting on CH or CH2 groups, NAD or NADP as acceptor](http://amigo.geneontology.org/cgi-bin/amigo/go.cgi?action=query&view=query&query=GO:0016726&search_constraint=terms) | TC409459, TC370114 |
| [Acetyl-CoA C-acetyltransferase activity](http://amigo.geneontology.org/cgi-bin/amigo/go.cgi?action=query&view=query&query=GO:0003985&search_constraint=terms) | TC456784, TC398514 |
| [C-acetyltransferase activity](http://amigo.geneontology.org/cgi-bin/amigo/go.cgi?action=query&view=query&query=GO:0016453&search_constraint=terms) | TC456784, TC398514 |
| [Neutral amino acid transmembrane transporter activity](http://amigo.geneontology.org/cgi-bin/amigo/go.cgi?action=query&view=query&query=GO:0015175&search_constraint=terms) | TC404158, TC404978, TC379171, TC384678 |
| [Trehalose-phosphatase activity](http://amigo.geneontology.org/cgi-bin/amigo/go.cgi?action=query&view=query&query=GO:0004805&search_constraint=terms) | TC392297, TC399408 |
| [2-alkenal reductase activity](http://amigo.geneontology.org/cgi-bin/amigo/go.cgi?action=query&view=query&query=GO:0032440&search_constraint=terms) | TC427405, TC440526 |
| [Carbohydrate kinase activity](http://amigo.geneontology.org/cgi-bin/amigo/go.cgi?action=query&view=query&query=GO:0019200&search_constraint=terms) | TC388751, TC416438, TC379422 |
| [Aspartate-tRNA ligase activity](http://amigo.geneontology.org/cgi-bin/amigo/go.cgi?action=query&view=query&query=GO:0004815&search_constraint=terms) | TC381988, TC410078, TC445166, TC404843 |
| [Calmodulin-dependent protein kinase activity](http://amigo.geneontology.org/cgi-bin/amigo/go.cgi?action=query&view=query&query=GO:0004683&search_constraint=terms) | TC370912, TC411941, TC370114 |
| [Oxidoreductase activity, acting on paired donors, with incorporation or reduction of molecular oxygen](http://amigo.geneontology.org/cgi-bin/amigo/go.cgi?action=query&view=query&query=GO:0016705&search_constraint=terms) | TC375268, TC430821, TC440632, TC413199, TC381923, TC384122, TC385710 |
| [Calmodulin-dependent cyclic-nucleotide phosphodiesterase activity](http://amigo.geneontology.org/cgi-bin/amigo/go.cgi?action=query&view=query&query=GO:0004117&search_constraint=terms) | TC409459, TC370114 |
| [Protein histidine Kinase activity](http://amigo.geneontology.org/cgi-bin/amigo/go.cgi?action=query&view=query&query=GO:0004673&search_constraint=terms) | TC413339, TC405041 |
| [Phosphate transmembrane transporter activity](http://amigo.geneontology.org/cgi-bin/amigo/go.cgi?action=query&view=query&query=GO:0015114&search_constraint=terms) | TC432154, TC373958 |
| [cAMP binding](http://amigo.geneontology.org/cgi-bin/amigo/go.cgi?action=query&view=query&query=GO:0030552&search_constraint=terms) | TC409459, TC370114 |
| [L-3-cyanoalanine synthase activity](http://amigo.geneontology.org/cgi-bin/amigo/go.cgi?action=query&view=query&query=GO:0050017&search_constraint=terms) | TC373702, TC376351 |
| [Cysteine-type endopeptidase activity](http://amigo.geneontology.org/cgi-bin/amigo/go.cgi?action=query&view=query&query=GO:0004197&search_constraint=terms) | TC442623, TC414899, TC445939, TC452050, TC391995 |
| [Asparagine-tRNA ligase activity](http://amigo.geneontology.org/cgi-bin/amigo/go.cgi?action=query&view=query&query=GO:0004816&search_constraint=terms) | TC381988, TC410078, TC445166, TC404843 |
| [Transferase activity, transferring hexosyl groups](http://amigo.geneontology.org/cgi-bin/amigo/go.cgi?action=query&view=query&query=GO:0016758&search_constraint=terms) | TC439939, TC368548, TC382830, TC372563, TC388819, TC393948, CK212850, TC370347, TC403234, TC436347, TC390630, TC379470, TC376758, TC369736, TC384373 |
| [Aminoacyl-tRNA ligase activity](http://amigo.geneontology.org/cgi-bin/amigo/go.cgi?action=query&view=query&query=GO:0004812&search_constraint=terms) | TC381988, TC410078, TC387064, TC425957, TC445166, TC404843 |
| [Ligase activity, forming carbon-oxygen bonds](http://amigo.geneontology.org/cgi-bin/amigo/go.cgi?action=query&view=query&query=GO:0016875&search_constraint=terms) | TC381988, TC410078, TC387064, TC425957, TC445166, TC404843 |
| [Ligase activity, forming aminoacyl-tRNA and related compounds](http://amigo.geneontology.org/cgi-bin/amigo/go.cgi?action=query&view=query&query=GO:0016876&search_constraint=terms) | TC381988, TC410078, TC387064, TC425957, TC445166, TC404843 |
| [C-acyltransferase activity](http://amigo.geneontology.org/cgi-bin/amigo/go.cgi?action=query&view=query&query=GO:0016408&search_constraint=terms) | TC456784, TC398514 |
| [Growth hormone-releasing hormone activity](http://amigo.geneontology.org/cgi-bin/amigo/go.cgi?action=query&view=query&query=GO:0016608&search_constraint=terms) | TC409459, TC370114 |
| [Phosphotransferase activity, nitrogenous group as acceptor](http://amigo.geneontology.org/cgi-bin/amigo/go.cgi?action=query&view=query&query=GO:0016775&search_constraint=terms) | TC413339, TC405041 |
| [Ghrelin receptor binding](http://amigo.geneontology.org/cgi-bin/amigo/go.cgi?action=query&view=query&query=GO:0031768&search_constraint=terms) | TC409459, TC370114 |
| [AMP binding](http://amigo.geneontology.org/cgi-bin/amigo/go.cgi?action=query&view=query&query=GO:0016208&search_constraint=terms) | TC409459, TC370114 |
| [Adenylyltransferase activity](http://amigo.geneontology.org/cgi-bin/amigo/go.cgi?action=query&view=query&query=GO:0070566&search_constraint=terms) | TC399245, TC369899 |
| [Axon guidance receptor activity](http://amigo.geneontology.org/cgi-bin/amigo/go.cgi?action=query&view=query&query=GO:0008046&search_constraint=terms) | TC403588, TC461622, TC380882 |
| [Phospholipid-translocating ATPase activity](http://amigo.geneontology.org/cgi-bin/amigo/go.cgi?action=query&view=query&query=GO:0004012&search_constraint=terms) | TC446092, TC386322 |
| [Calcium activated cation channel activity](http://amigo.geneontology.org/cgi-bin/amigo/go.cgi?action=query&view=query&query=GO:0005227&search_constraint=terms) | TC456619, TC370315 |
| [DNA-dependent ATPase activity](http://amigo.geneontology.org/cgi-bin/amigo/go.cgi?action=query&view=query&query=GO:0008094&search_constraint=terms) | TC389162, TC374164 |
| [Calcium-activated potassium channel activity](http://amigo.geneontology.org/cgi-bin/amigo/go.cgi?action=query&view=query&query=GO:0015269&search_constraint=terms) | TC456619, TC370315 |
| [Small conductance calcium-activated potassium channel activity](http://amigo.geneontology.org/cgi-bin/amigo/go.cgi?action=query&view=query&query=GO:0016286&search_constraint=terms) | TC456619, TC370315 |
| [Ion gated channel activity](http://amigo.geneontology.org/cgi-bin/amigo/go.cgi?action=query&view=query&query=GO:0022839&search_constraint=terms) | TC456619, TC370315 |
| [Cyclic nucleotide binding](http://amigo.geneontology.org/cgi-bin/amigo/go.cgi?action=query&view=query&query=GO:0030551&search_constraint=terms) | TC409459, TC370114 |
| [Chitinase activity](http://amigo.geneontology.org/cgi-bin/amigo/go.cgi?action=query&view=query&query=GO:0004568&search_constraint=terms) | TC373787, TC384010, TC393523, DR740372, CK201148, TC369199, TC377308 |
| [Adenyl ribonucleotide binding](http://amigo.geneontology.org/cgi-bin/amigo/go.cgi?action=query&view=query&query=GO:0032559&search_constraint=terms) | CA646741, TC389162, TC375834, TC394796, TC423354, TC425957, TC459656, TC449724, TC455515, TC394965, TC374240, TC413339, TC369655, TC397994, TC457991, TC380882, TC423880, TC371242, TC425690, TC370315, TC399352, TC405511, TC395723, TC376420, TC372167, TC393820, TC394661, CJ944525, TC384553, TC369092, TC403588, TC435595, TC374164, TC429747, TC405041, TC394728, TC371641, TC387683, TC384688, TC407076, TC391947, TC398379, TC370114, TC409459, TC387064, TC406807, CJ550278, CD892838, TC395090, TC368591, TC381957 |
| [Secondary active transmembrane transporter activity](http://amigo.geneontology.org/cgi-bin/amigo/go.cgi?action=query&view=query&query=GO:0015291&search_constraint=terms) | TC406594, TC392709, TC369182, TC384344, TC373958, TC378153, TC429374, TC373678 |
| [Protein serine/threonine/tyrosine kinase activity](http://amigo.geneontology.org/cgi-bin/amigo/go.cgi?action=query&view=query&query=GO:0004712&search_constraint=terms) | TC384688, TC370315, TC437163 |
| [Intramolecular oxidoreductase activity, interconverting aldoses and ketoses](http://amigo.geneontology.org/cgi-bin/amigo/go.cgi?action=query&view=query&query=GO:0016861&search_constraint=terms) | TC376248, TC402668, TC372654 |
| [Oxidoreductase activity, acting on CH-OH group of donors](http://amigo.geneontology.org/cgi-bin/amigo/go.cgi?action=query&view=query&query=GO:0016614&search_constraint=terms) | TC392363, TC384194, TC383270, TC433574, TC387981, TC388950, TC390285, TC381817, TC377766, TC387766, TC386707, TC424376, TC403573, TC371145, TC376306, TC371242, TC375554, TC380416, TC422142, TC372580, TC434396, TC388158 |
| [Adenyl nucleotide binding](http://amigo.geneontology.org/cgi-bin/amigo/go.cgi?action=query&view=query&query=GO:0030554&search_constraint=terms) | CA646741, TC389162, TC375834, TC394796, TC423354, TC425957, TC459656, TC449724, TC455515, TC394965, TC374240, TC413339, TC369655, TC397994, TC457991, TC380882, TC423880, TC371242, TC425690, TC370315, TC399352, TC405511, TC395723, TC376420, TC372167, TC393820, TC394661, CJ944525, TC384553, TC369092, TC403588, TC435595, TC374164, TC429747, TC405041, TC394728, TC371641, TC387683, TC384688, TC407076, TC391947, TC398379, TC370114, TC409459, TC387064, TC406807, CJ550278, CD892838, TC395090, TC368591, TC381957 |
| [Phosphoenolpyruvate carboxykinase activity](http://amigo.geneontology.org/cgi-bin/amigo/go.cgi?action=query&view=query&query=GO:0004611&search_constraint=terms) | TC455515, TC369064 |
| [Aryl-alcohol dehydrogenase activity](http://amigo.geneontology.org/cgi-bin/amigo/go.cgi?action=query&view=query&query=GO:0018456&search_constraint=terms) | TC434396, TC386707 |
| [Nucleoside binding](http://amigo.geneontology.org/cgi-bin/amigo/go.cgi?action=query&view=query&query=GO:0001882&search_constraint=terms) | CA646741, TC389162, TC375834, TC394796, TC423354, TC425957, TC459656, TC449724, TC455515, TC394965, TC374240, TC413339, TC369655, TC397994, TC457991, TC380882, TC423880, TC371242, TC425690, TC370315, TC399352, TC405511, TC395723, TC376420, TC372167, TC393820, TC394661, CJ944525, TC384553, TC369092, TC403588, TC435595, TC374164, TC429747, TC405041, TC394728, TC371641, TC387683, TC384688, TC407076, TC391947, TC398379, TC370114, TC409459, TC387064, TC406807, CJ550278, CD892838, TC395090, TC368591, TC381957 |
| [Purine nucleoside binding](http://amigo.geneontology.org/cgi-bin/amigo/go.cgi?action=query&view=query&query=GO:0001883&search_constraint=terms) | CA646741, TC389162, TC375834, TC394796, TC423354, TC425957, TC459656, TC449724, TC455515, TC394965, TC374240, TC413339, TC369655, TC397994, TC457991, TC380882, TC423880, TC371242, TC425690, TC370315, TC399352, TC405511, TC395723, TC376420, TC372167, TC393820, TC394661, CJ944525, TC384553, TC369092, TC403588, TC435595, TC374164, TC429747, TC405041, TC394728, TC371641, TC387683, TC384688, TC407076, TC391947, TC398379, TC370114, TC409459, TC387064, TC406807, CJ550278, CD892838, TC395090, TC368591, TC381957 |
| [Carboxylic acid transmembrane transporter activity](http://amigo.geneontology.org/cgi-bin/amigo/go.cgi?action=query&view=query&query=GO:0046943&search_constraint=terms) | TC382910, TC404158, TC391621, TC404978, TC379171, TC435799, TC384678 |
| [Microfilament motor activity](http://amigo.geneontology.org/cgi-bin/amigo/go.cgi?action=query&view=query&query=GO:0000146&search_constraint=terms) | TC459656, TC406807, CJ944525 |
| [Organic acid transmembrane transporter activity](http://amigo.geneontology.org/cgi-bin/amigo/go.cgi?action=query&view=query&query=GO:0005342&search_constraint=terms) | TC382910, TC404158, TC391621, TC404978, TC379171, TC435799, TC384678 |
| [Phospholipid transporter activity](http://amigo.geneontology.org/cgi-bin/amigo/go.cgi?action=query&view=query&query=GO:0005548&search_constraint=terms) | TC446092, TC386322 |
| [Peroxidase activity](http://amigo.geneontology.org/cgi-bin/amigo/go.cgi?action=query&view=query&query=GO:0004601&search_constraint=terms) | TC429713, TC375760, TC382734, TC387817, DR739303, TC395069, TC422841, TC381068, TC384357, TC398026, TC372867, TC448840, TC396657, TC405773, TC385326 |
| [Oxidoreductase activity, acting on peroxide as acceptor](http://amigo.geneontology.org/cgi-bin/amigo/go.cgi?action=query&view=query&query=GO:0016684&search_constraint=terms) | TC429713, TC375760, TC382734, TC387817, DR739303, TC395069, TC422841, TC381068, TC384357, TC398026, TC372867, TC448840, TC396657, TC405773, TC385326 |
| [Protein heterodimerization activity](http://amigo.geneontology.org/cgi-bin/amigo/go.cgi?action=query&view=query&query=GO:0046982&search_constraint=terms) | TC407978, TC461622, TC380882, CJ944525, TC459656, TC403588, TC438243, TC406807 |
| [Lipid transporter activity](http://amigo.geneontology.org/cgi-bin/amigo/go.cgi?action=query&view=query&query=GO:0005319&search_constraint=terms) | TC446092, TC386322 |
| [Transferase activity, transferring glycosyl groups](http://amigo.geneontology.org/cgi-bin/amigo/go.cgi?action=query&view=query&query=GO:0016757&search_constraint=terms) | TC439939, TC368548, TC382830, TC372563, TC388819, TC393948, CK212850, TC370347, TC403234, TC436347, TC401158, TC390630, TC379470, TC376758, TC369736, TC384373 |
| [Transcription repressor activity](http://amigo.geneontology.org/cgi-bin/amigo/go.cgi?action=query&view=query&query=GO:0016564&search_constraint=terms) | TC460615, TC404413, TC418850, TC400362, TC370114, TC459656, TC409459, TC406807, TC393960 |
| [ATP binding](http://amigo.geneontology.org/cgi-bin/amigo/go.cgi?action=query&view=query&query=GO:0005524&search_constraint=terms) | CA646741, TC389162, TC375834, TC394796, TC423354, TC425957, TC459656, TC449724, TC455515, TC394965, TC374240, TC413339, TC369655, TC397994, TC457991, TC380882, TC423880, TC371242, TC425690, TC370315, TC399352, TC395723, TC405511, TC376420, TC372167, TC393820, TC394661, CJ944525, TC384553, TC369092, TC403588, TC435595, TC374164, TC429747, TC405041, TC394728, TC371641, TC387683, TC384688, TC407076, TC391947, TC398379, TC387064, TC406807, CJ550278, CD892838, TC395090, TC368591, TC381957 |
| [Poly(C) RNA binding](http://amigo.geneontology.org/cgi-bin/amigo/go.cgi?action=query&view=query&query=GO:0017130&search_constraint=terms) | TC449724, TC393820 |
| [Cinnamyl-alcohol dehydrogenase activity](http://amigo.geneontology.org/cgi-bin/amigo/go.cgi?action=query&view=query&query=GO:0045551&search_constraint=terms) | TC434396, TC386707 |
| [Oxidoreductase activity](http://amigo.geneontology.org/cgi-bin/amigo/go.cgi?action=query&view=query&query=GO:0016491&search_constraint=terms) | TC390285, TC391946, TC377766, TC381068, TC398026, TC387766, TC421162, TC376306, TC398121, TC371242, DR739303, TC388136, TC392363, TC427006, TC384122, TC388950, TC430821, TC371037, TC427405, TC448840, TC375268, TC371145, TC407076, TC382734, TC375554, TC370114, TC409459, TC379965, TC416471, GH732878, TC383270, TC392109, TC375726, TC385710, TC369348, TC384357, TC397909, TC372867, TC411908, TC403573, TC440632, TC447801, TC392247, TC409187, TC380416, TC387817, TC389993, TC372580, TC427981, TC395069, TC434396, TC413199, TC398536, TC421880, TC396083, TC384194, TC372701, TC433574, TC377190, TC387981, TC430544, TC381817, TC370633, TC386707, TC424376, TC392033, TC429713, TC388049, TC375760, TC381923, TC382786, TC440526, TC422142, TC422841, TC431198, TC388158, TC396657, TC405773, TC385326 |
| [Intramolecular transferase activity, phosphotransferases](http://amigo.geneontology.org/cgi-bin/amigo/go.cgi?action=query&view=query&query=GO:0016868&search_constraint=terms) | TC370885, TC372330 |
| [Protein serine/threonine kinase activity](http://amigo.geneontology.org/cgi-bin/amigo/go.cgi?action=query&view=query&query=GO:0004674&search_constraint=terms) | CA646741, TC374230, TC395723, TC376420, TC372167, TC394796, TC407340, TC394965, TC429747, TC374240, TC369655, TC411941, TC387683, TC407076, TC457991, TC370912, TC370114, CJ550278, TC395090, TC381957, TC370315, TC398592, TC382045 |
| [Structural constituent of muscle](http://amigo.geneontology.org/cgi-bin/amigo/go.cgi?action=query&view=query&query=GO:0008307&search_constraint=terms) | TC459656, TC406807, TC410126, CJ944525 |
| [Protein tyrosine kinase activity](http://amigo.geneontology.org/cgi-bin/amigo/go.cgi?action=query&view=query&query=GO:0004713&search_constraint=terms) | CA646741, TC395723, TC376420, TC372167, TC459656, TC435595, TC394965, TC429747, TC374240, TC369655, TC387683, TC407076, TC457991, CJ550278, TC406807, TC395090, TC370315 |
| [Acetyltransferase activity](http://amigo.geneontology.org/cgi-bin/amigo/go.cgi?action=query&view=query&query=GO:0016407&search_constraint=terms) | TC409843, TC456784, TC434442, TC398514 |
| [Intramolecular oxidoreductase activity](http://amigo.geneontology.org/cgi-bin/amigo/go.cgi?action=query&view=query&query=GO:0016860&search_constraint=terms) | TC380416, TC376248, TC402668, TC372654 |
| [Carboxypeptidase activity](http://amigo.geneontology.org/cgi-bin/amigo/go.cgi?action=query&view=query&query=GO:0004180&search_constraint=terms) | TC374409, TC382080 |
| [Serine-type carboxypeptidase activity](http://amigo.geneontology.org/cgi-bin/amigo/go.cgi?action=query&view=query&query=GO:0004185&search_constraint=terms) | TC374409, TC382080 |
| [Endonuclease activity](http://amigo.geneontology.org/cgi-bin/amigo/go.cgi?action=query&view=query&query=GO:0004519&search_constraint=terms) | TC373791, TC375864 |
| [Transition metal ion transmembrane transporter activity](http://amigo.geneontology.org/cgi-bin/amigo/go.cgi?action=query&view=query&query=GO:0046915&search_constraint=terms) | TC429341, TC444402 |
| [Serine-type exopeptidase activity](http://amigo.geneontology.org/cgi-bin/amigo/go.cgi?action=query&view=query&query=GO:0070008&search_constraint=terms) | TC374409, TC382080 |
| [UDP-glucosyltransferase activity](http://amigo.geneontology.org/cgi-bin/amigo/go.cgi?action=query&view=query&query=GO:0035251&search_constraint=terms) | TC368548, TC370347, TC369736 |
| [Transaminase activity](http://amigo.geneontology.org/cgi-bin/amigo/go.cgi?action=query&view=query&query=GO:0008483&search_constraint=terms) | TC370350, TC385526, TC369664, TC413263 |
| [Transferase activity, transferring nitrogenous groups](http://amigo.geneontology.org/cgi-bin/amigo/go.cgi?action=query&view=query&query=GO:0016769&search_constraint=terms) | TC370350, TC385526, TC369664, TC413263 |
| [Oxidoreductase activity, acting on the CH-OH group of donors, NAD or NADP as acceptor](http://amigo.geneontology.org/cgi-bin/amigo/go.cgi?action=query&view=query&query=GO:0016616&search_constraint=terms) | TC383270, TC433574, TC387981, TC388950, TC390285, TC381817, TC377766, TC386707, TC424376, TC403573, TC371145, TC376306, TC371242, TC375554, TC380416, TC422142, TC434396, TC388158 |
| [Di-, tri-valent inorganic cation transmembrane transporter activity](http://amigo.geneontology.org/cgi-bin/amigo/go.cgi?action=query&view=query&query=GO:0015082&search_constraint=terms) | TC429341, TC444402 |
| [Potassium channel activity](http://amigo.geneontology.org/cgi-bin/amigo/go.cgi?action=query&view=query&query=GO:0005267&search_constraint=terms) | CK211589, TC456619, TC413571, TC370315 |
| [Oxygen binding](http://amigo.geneontology.org/cgi-bin/amigo/go.cgi?action=query&view=query&query=GO:0019825&search_constraint=terms) | CV771134, TC391742, TC372953, TC371073 |
| [Ionotropic glutamate receptor activity](http://amigo.geneontology.org/cgi-bin/amigo/go.cgi?action=query&view=query&query=GO:0004970&search_constraint=terms) | CK211589, TC413571 |
| [Alpha-amino-3-hydroxy-5-methyl-4-isoxazole propionate selective glutamate receptor activity](http://amigo.geneontology.org/cgi-bin/amigo/go.cgi?action=query&view=query&query=GO:0004971&search_constraint=terms) | CK211589, TC413571 |
| [Kainate selective glutamate receptor activity](http://amigo.geneontology.org/cgi-bin/amigo/go.cgi?action=query&view=query&query=GO:0015277&search_constraint=terms) | CK211589, TC413571 |
| [Hydrolase activity, hydrolyzing O-glycosyl compounds](http://amigo.geneontology.org/cgi-bin/amigo/go.cgi?action=query&view=query&query=GO:0004553&search_constraint=terms) | TC388819, DR740372, TC452945, TC369844, CK201148, TC393198, TC369199, TC377308, TC373787, TC384010, TC393523, TC387064, TC375124 |
| [Ribonucleoprotein binding](http://amigo.geneontology.org/cgi-bin/amigo/go.cgi?action=query&view=query&query=GO:0043021&search_constraint=terms) | TC409843, TC423804, TC390402 |
| [Hydrolase activity, acting on glycosyl bonds](http://amigo.geneontology.org/cgi-bin/amigo/go.cgi?action=query&view=query&query=GO:0016798&search_constraint=terms) | TC382830, TC388819, DR740372, TC452945, CK212850, TC369844, CK201148, TC393198, TC369199, TC377308, TC373787, TC384010, TC393523, TC387064, TC375124 |
| [Glucuronosyltransferase activity](http://amigo.geneontology.org/cgi-bin/amigo/go.cgi?action=query&view=query&query=GO:0015020&search_constraint=terms) | TC390630, TC376758, TC393948 |
| [Carbohydrate phosphatase activity](http://amigo.geneontology.org/cgi-bin/amigo/go.cgi?action=query&view=query&query=GO:0019203&search_constraint=terms) | TC392297, TC399408 |
| [Carbon-sulfur lyase activity](http://amigo.geneontology.org/cgi-bin/amigo/go.cgi?action=query&view=query&query=GO:0016846&search_constraint=terms) | TC373702, CK161232, TC400056, TC376351 |
| [Cysteine synthase activity](http://amigo.geneontology.org/cgi-bin/amigo/go.cgi?action=query&view=query&query=GO:0004124&search_constraint=terms) | TC373702, TC376351 |
| [Glucosyltransferase activity](http://amigo.geneontology.org/cgi-bin/amigo/go.cgi?action=query&view=query&query=GO:0046527&search_constraint=terms) | TC368548, TC370347, TC369736 |
| [Poly(A) RNA binding](http://amigo.geneontology.org/cgi-bin/amigo/go.cgi?action=query&view=query&query=GO:0008143&search_constraint=terms) | TC449724, TC393820 |
| [Poly-purine tract binding](http://amigo.geneontology.org/cgi-bin/amigo/go.cgi?action=query&view=query&query=GO:0070717&search_constraint=terms) | TC449724, TC393820 |
| [Active transmembrane transporter activity](http://amigo.geneontology.org/cgi-bin/amigo/go.cgi?action=query&view=query&query=GO:0022804&search_constraint=terms) | TC392709, TC404158, TC384344, TC373958, TC378225, TC391621, TC373678, TC382910, TC406594, TC384071, TC369182, TC397994, TC371242, TC378153, TC429374, TC377749, TC398379, TC404978, TC379171, TC375813, TC384678, TC387621 |
| [S-adenosylmethionine-dependent methyltransferase activity](http://amigo.geneontology.org/cgi-bin/amigo/go.cgi?action=query&view=query&query=GO:0008757&search_constraint=terms) | TC380590, TC413854, TC402072 |
| [mRNA binding](http://amigo.geneontology.org/cgi-bin/amigo/go.cgi?action=query&view=query&query=GO:0003729&search_constraint=terms) | TC388049, TC449724, TC387710, TC404606, TC393820 |
| [Anion transmembrane transporter activity](http://amigo.geneontology.org/cgi-bin/amigo/go.cgi?action=query&view=query&query=GO:0008509&search_constraint=terms) | TC432154, TC387319, TC373958, TC373002 |
| [DNA-directed RNA polymerase activity](http://amigo.geneontology.org/cgi-bin/amigo/go.cgi?action=query&view=query&query=GO:0003899&search_constraint=terms) | EB512907, TC397500, TC443814, TC397176, TC368549 |
| [RNA polymerase activity](http://amigo.geneontology.org/cgi-bin/amigo/go.cgi?action=query&view=query&query=GO:0034062&search_constraint=terms) | EB512907, TC397500, TC443814, TC397176, TC368549 |
| [Protein prenyltransferase activity](http://amigo.geneontology.org/cgi-bin/amigo/go.cgi?action=query&view=query&query=GO:0008318&search_constraint=terms) | CK211589, TC413571 |
| [Cysteine-type peptidase activity](http://amigo.geneontology.org/cgi-bin/amigo/go.cgi?action=query&view=query&query=GO:0008234&search_constraint=terms) | TC370106, TC442623, TC414899, TC372664, TC445939, TC370158, TC391995, CV780698, TC452050 |
| [Hydrolase activity, acting on carbon-nitrogen (but not peptide) bonds, in linear amides](http://amigo.geneontology.org/cgi-bin/amigo/go.cgi?action=query&view=query&query=GO:0016811&search_constraint=terms) | TC384735, TC441343, TC406106, CJ930688, CK211589, TC381817, TC383139, TC413571 |
| [Protein kinase regulator activity](http://amigo.geneontology.org/cgi-bin/amigo/go.cgi?action=query&view=query&query=GO:0019887&search_constraint=terms) | TC379069, TC438243, TC407978 |
| [Identical protein binding](http://amigo.geneontology.org/cgi-bin/amigo/go.cgi?action=query&view=query&query=GO:0042802&search_constraint=terms) | TC374230, TC373613, TC441343, TC461622, CJ944525, CK211589, TC459656, TC381817, TC403588, TC413571, TC407340, TC409843, TC384735, TC432185, TC380882, TC406807, TC382045 |
| [Exopeptidase activity](http://amigo.geneontology.org/cgi-bin/amigo/go.cgi?action=query&view=query&query=GO:0008238&search_constraint=terms) | TC397033, CK207939, TC374409, TC382080 |
| [Selenide, water dikinase activity](http://amigo.geneontology.org/cgi-bin/amigo/go.cgi?action=query&view=query&query=GO:0004756&search_constraint=terms) | TC449724, TC393820 |
| [Nuclear export signal receptor activity](http://amigo.geneontology.org/cgi-bin/amigo/go.cgi?action=query&view=query&query=GO:0005049&search_constraint=terms) | TC409459, TC370114 |
| [Intramolecular transferase activity](http://amigo.geneontology.org/cgi-bin/amigo/go.cgi?action=query&view=query&query=GO:0016866&search_constraint=terms) | TC417341, TC370885, TC372330 |
| [Phosphatidylethanolamine binding](http://amigo.geneontology.org/cgi-bin/amigo/go.cgi?action=query&view=query&query=GO:0008429&search_constraint=terms) | TC459656, TC406807 |
| [Transcription factor binding](http://amigo.geneontology.org/cgi-bin/amigo/go.cgi?action=query&view=query&query=GO:0008134&search_constraint=terms) | TC460615, TC384735, TC373129, TC441343, TC394118, TC404413, TC400362, CK211589, TC370114, TC459656, TC406807, TC381817, TC413571, TC393960 |
| [Kinase regulator activity](http://amigo.geneontology.org/cgi-bin/amigo/go.cgi?action=query&view=query&query=GO:0019207&search_constraint=terms) | TC379069, TC438243, TC407978 |
| [Phosphotransferase activity, paired acceptors](http://amigo.geneontology.org/cgi-bin/amigo/go.cgi?action=query&view=query&query=GO:0016781&search_constraint=terms) | TC449724, TC393820 |
| [Protein phosphatase type 2A regulator activity](http://amigo.geneontology.org/cgi-bin/amigo/go.cgi?action=query&view=query&query=GO:0008601&search_constraint=terms) | TC398606, TC375530 |
| [Ligase activity, forming carbon-sulfur bonds](http://amigo.geneontology.org/cgi-bin/amigo/go.cgi?action=query&view=query&query=GO:0016877&search_constraint=terms) | TC387116, TC371242 |
| [ATPase activity](http://amigo.geneontology.org/cgi-bin/amigo/go.cgi?action=query&view=query&query=GO:0016887&search_constraint=terms) | TC389162, TC405511, TC409190, TC446092, TC386322, CJ944525, TC378225, TC423354, TC459656, TC403588, TC369092, TC374164, TC384071, TC397994, TC380882, TC423880, TC371242, TC377749, TC398379, TC425690, TC406807, TC375813, TC399352 |
| [Xyloglucan:xyloglucosyl transferase activity](http://amigo.geneontology.org/cgi-bin/amigo/go.cgi?action=query&view=query&query=GO:0016762&search_constraint=terms) | TC382830, CK212850 |
| [Transcription activator activity](http://amigo.geneontology.org/cgi-bin/amigo/go.cgi?action=query&view=query&query=GO:0016563&search_constraint=terms) | TC460615, TC400362, TC409459, TC370114, TC373129, TC393960, TC394118, TC404413 |
| [Acetylcholine receptor regulator activity](http://amigo.geneontology.org/cgi-bin/amigo/go.cgi?action=query&view=query&query=GO:0030548&search_constraint=terms) | TC407076, TC382045 |
| [Antioxidant activity](http://amigo.geneontology.org/cgi-bin/amigo/go.cgi?action=query&view=query&query=GO:0016209&search_constraint=terms) | TC429713, TC375760, TC382734, TC387817, DR739303, TC395069, TC422841, TC381068, TC384357, TC398026, TC372867, TC448840, TC396657, TC405773, TC385326 |
| [Protein kinase inhibitor activity](http://amigo.geneontology.org/cgi-bin/amigo/go.cgi?action=query&view=query&query=GO:0004860&search_constraint=terms) | TC438243, TC407978 |
| [Kinase inhibitor activity](http://amigo.geneontology.org/cgi-bin/amigo/go.cgi?action=query&view=query&query=GO:0019210&search_constraint=terms) | TC438243, TC407978 |
| [Carbon-oxygen lyase activity](http://amigo.geneontology.org/cgi-bin/amigo/go.cgi?action=query&view=query&query=GO:0016835&search_constraint=terms) | TC380416, TC373702, TC453487, TC388049, TC394028, TC451519, TC376351 |
| [Ankyrin binding](http://amigo.geneontology.org/cgi-bin/amigo/go.cgi?action=query&view=query&query=GO:0030506&search_constraint=terms) | TC421914, TC374240, TC395723, CJ550278, TC378271 |
| [Protein tyrosine phosphatase activity](http://amigo.geneontology.org/cgi-bin/amigo/go.cgi?action=query&view=query&query=GO:0004725&search_constraint=terms) | TC459656, TC406807, TC409650 |
| [Ran GTPase binding](http://amigo.geneontology.org/cgi-bin/amigo/go.cgi?action=query&view=query&query=GO:0008536&search_constraint=terms) | TC435808, TC409459, TC370114 |
| [Ras GTPase binding](http://amigo.geneontology.org/cgi-bin/amigo/go.cgi?action=query&view=query&query=GO:0017016&search_constraint=terms) | TC435808, TC409459, TC370114 |
| [Small GTPase binding](http://amigo.geneontology.org/cgi-bin/amigo/go.cgi?action=query&view=query&query=GO:0031267&search_constraint=terms) | TC435808, TC409459, TC370114 |
| [GTPase binding](http://amigo.geneontology.org/cgi-bin/amigo/go.cgi?action=query&view=query&query=GO:0051020&search_constraint=terms) | TC435808, TC409459, TC370114 |
| [Actin binding](http://amigo.geneontology.org/cgi-bin/amigo/go.cgi?action=query&view=query&query=GO:0003779&search_constraint=terms) | TC381988, TC410078, TC409459, TC459656, TC370114, TC406807, TC445166, TC404843, CJ944525 |
| [Specific transcriptional repressor activity](http://amigo.geneontology.org/cgi-bin/amigo/go.cgi?action=query&view=query&query=GO:0016566&search_constraint=terms) | TC409459, TC370114 |
| [Cytokine binding](http://amigo.geneontology.org/cgi-bin/amigo/go.cgi?action=query&view=query&query=GO:0019955&search_constraint=terms) | TC376420, TC394118 |
| [Hormone activity](http://amigo.geneontology.org/cgi-bin/amigo/go.cgi?action=query&view=query&query=GO:0005179&search_constraint=terms) | TC409459, TC370114 |
| [Protein homodimerization activity](http://amigo.geneontology.org/cgi-bin/amigo/go.cgi?action=query&view=query&query=GO:0042803&search_constraint=terms) | TC409843, TC459656, TC406807, CJ944525 |
| [Prenyltransferase activity](http://amigo.geneontology.org/cgi-bin/amigo/go.cgi?action=query&view=query&query=GO:0004659&search_constraint=terms) | CK211589, TC413571 |
| [Phosphatase regulator activity](http://amigo.geneontology.org/cgi-bin/amigo/go.cgi?action=query&view=query&query=GO:0019208&search_constraint=terms) | TC398606, TC375530 |
| [Protein phosphatase regulator activity](http://amigo.geneontology.org/cgi-bin/amigo/go.cgi?action=query&view=query&query=GO:0019888&search_constraint=terms) | TC398606, TC375530 |
| [Protein dimerization activity](http://amigo.geneontology.org/cgi-bin/amigo/go.cgi?action=query&view=query&query=GO:0046983&search_constraint=terms) | TC409843, TC407978, TC461622, TC380882, CJ944525, TC370114, TC459656, TC409459, TC406807, TC403588, TC438243, TC376490 |
| [ATPase activity, coupled](http://amigo.geneontology.org/cgi-bin/amigo/go.cgi?action=query&view=query&query=GO:0042623&search_constraint=terms) | TC389162, TC405511, TC446092, TC386322, TC378225, TC423354, TC403588, TC374164, TC384071, TC397994, TC380882, TC371242, TC423880, TC377749, TC398379, TC425690, TC375813, TC399352 |
| [Methyltransferase activity](http://amigo.geneontology.org/cgi-bin/amigo/go.cgi?action=query&view=query&query=GO:0008168&search_constraint=terms) | TC380590, TC413854, TC417106, TC402072 |
| [Phosphogluconate dehydrogenase (decarboxylating) activity](http://amigo.geneontology.org/cgi-bin/amigo/go.cgi?action=query&view=query&query=GO:0004616&search_constraint=terms) | TC424376, TC390285, TC381817, TC377766, TC388950, TC371242 |
| [Hydrolase activity, acting on carbon-nitrogen (but not peptide) bonds](http://amigo.geneontology.org/cgi-bin/amigo/go.cgi?action=query&view=query&query=GO:0016810&search_constraint=terms) | TC384735, TC441343, TC406106, CJ930688, CK211589, TC381817, TC383139, TC413571 |
| [Aminopeptidase activity](http://amigo.geneontology.org/cgi-bin/amigo/go.cgi?action=query&view=query&query=GO:0004177&search_constraint=terms) | TC397033, CK207939 |
| [Transferase activity, transferring one-carbon groups](http://amigo.geneontology.org/cgi-bin/amigo/go.cgi?action=query&view=query&query=GO:0016741&search_constraint=terms) | TC380590, TC413854, TC417106, TC402072, TC378601 |
| [G-protein coupled amine receptor activity](http://amigo.geneontology.org/cgi-bin/amigo/go.cgi?action=query&view=query&query=GO:0008227&search_constraint=terms) | TC425690, TC449724, TC387710, TC404606, TC393820 |
| [Receptor regulator activity](http://amigo.geneontology.org/cgi-bin/amigo/go.cgi?action=query&view=query&query=GO:0030545&search_constraint=terms) | TC407076, TC382045 |
| [Phosphoprotein phosphatase activity](http://amigo.geneontology.org/cgi-bin/amigo/go.cgi?action=query&view=query&query=GO:0004721&search_constraint=terms) | TC380125, TC409650, TC370114, TC410954, TC459656, TC378432, TC406807, TC424154, TC429747 |
| [Hydro-lyase activity](http://amigo.geneontology.org/cgi-bin/amigo/go.cgi?action=query&view=query&query=GO:0016836&search_constraint=terms) | TC380416, TC453487, TC388049, TC394028, TC451519 |
| [rRNA binding](http://amigo.geneontology.org/cgi-bin/amigo/go.cgi?action=query&view=query&query=GO:0019843&search_constraint=terms) | TC387861, TC416906, TC398862, TC418414, TC417388 |
| [Phosphatase activity](http://amigo.geneontology.org/cgi-bin/amigo/go.cgi?action=query&view=query&query=GO:0016791&search_constraint=terms) | TC399408, TC380125, TC409650, TC392297, TC370114, TC410954, TC459656, TC378432, TC406807, TC424154, TC429747 |
| [Fructose-bisphosphate aldolase activity](http://amigo.geneontology.org/cgi-bin/amigo/go.cgi?action=query&view=query&query=GO:0004332&search_constraint=terms) | TC373613, TC432185 |
| [Histone deacetylase activity](http://amigo.geneontology.org/cgi-bin/amigo/go.cgi?action=query&view=query&query=GO:0004407&search_constraint=terms) | CK211589, TC384735, TC381817, TC441343, TC413571 |
| [Deacetylase activity](http://amigo.geneontology.org/cgi-bin/amigo/go.cgi?action=query&view=query&query=GO:0019213&search_constraint=terms) | CK211589, TC384735, TC381817, TC441343, TC413571 |
| [Protein deacetylase activity](http://amigo.geneontology.org/cgi-bin/amigo/go.cgi?action=query&view=query&query=GO:0033558&search_constraint=terms) | CK211589, TC384735, TC381817, TC441343, TC413571 |
| [Acyl carrier activity](http://amigo.geneontology.org/cgi-bin/amigo/go.cgi?action=query&view=query&query=GO:0000036&search_constraint=terms) | TC455736, TC375918 |
| [G-protein-coupled receptor binding](http://amigo.geneontology.org/cgi-bin/amigo/go.cgi?action=query&view=query&query=GO:0001664&search_constraint=terms) | TC409459, TC370114, TC438243 |
| [Metal ion transmembrane transporter activity](http://amigo.geneontology.org/cgi-bin/amigo/go.cgi?action=query&view=query&query=GO:0046873&search_constraint=terms) | TC429341, TC444402 |
| [Transcription coactivator activity](http://amigo.geneontology.org/cgi-bin/amigo/go.cgi?action=query&view=query&query=GO:0003713&search_constraint=terms) | TC373129, TC394118 |
| [Heat shock protein binding](http://amigo.geneontology.org/cgi-bin/amigo/go.cgi?action=query&view=query&query=GO:0031072&search_constraint=terms) | TC409459, TC370114 |
| [Kinase binding](http://amigo.geneontology.org/cgi-bin/amigo/go.cgi?action=query&view=query&query=GO:0019900&search_constraint=terms) | CK211589, TC376420, TC413571 |
| [Protein kinase binding](http://amigo.geneontology.org/cgi-bin/amigo/go.cgi?action=query&view=query&query=GO:0019901&search_constraint=terms) | CK211589, TC376420, TC413571 |
| [Motor activity](http://amigo.geneontology.org/cgi-bin/amigo/go.cgi?action=query&view=query&query=GO:0003774&search_constraint=terms) | TC390630, TC418845, TC418928, TC459656, TC406807, TC407076, TC393948, CJ944525 |
| [Chromatin binding](http://amigo.geneontology.org/cgi-bin/amigo/go.cgi?action=query&view=query&query=GO:0003682&search_constraint=terms) | TC374240, TC395723, TC402545, TC418850, TC370114, TC409459, CJ550278, TC417077, TC374879 |
| [Protein phosphorylated amino acid binding](http://amigo.geneontology.org/cgi-bin/amigo/go.cgi?action=query&view=query&query=GO:0045309&search_constraint=terms) | TC438243, TC407978 |
| [Ribonucleotide binding](http://amigo.geneontology.org/cgi-bin/amigo/go.cgi?action=query&view=query&query=GO:0032553&search_constraint=terms) | CA646741, TC389162, TC375834, TC394796, TC378790, TC423354, TC425957, TC459656, TC449724, TC455515, TC413571, TC394965, TC374240, TC413339, TC369655, TC397994, TC457991, BE585841, TC380882, TC423880, TC371242, TC425690, TC379171, TC370315, TC399352, TC372530, TC405511, TC395723, TC387410, TC376420, TC372167, TC393820, CJ944525, TC394661, TC384553, CK211589, TC369092, TC403588, TC435595, TC374164, TC405041, TC429747, TC394728, TC371641, TC384688, TC387683, TC407076, TC391947, TC398379, TC387064, TC370114, TC409459, TC406807, CJ550278, CD892838, TC395090, TC368591, TC381957 |
| [Purine ribonucleotide binding](http://amigo.geneontology.org/cgi-bin/amigo/go.cgi?action=query&view=query&query=GO:0032555&search_constraint=terms) | CA646741, TC389162, TC375834, TC394796, TC378790, TC423354, TC425957, TC459656, TC449724, TC455515, TC413571, TC394965, TC374240, TC413339, TC369655, TC397994, TC457991, BE585841, TC380882, TC423880, TC371242, TC425690, TC379171, TC370315, TC399352, TC372530, TC405511, TC395723, TC387410, TC376420, TC372167, TC393820, CJ944525, TC394661, TC384553, CK211589, TC369092, TC403588, TC435595, TC374164, TC405041, TC429747, TC394728, TC371641, TC384688, TC387683, TC407076, TC391947, TC398379, TC387064, TC370114, TC409459, TC406807, CJ550278, CD892838, TC395090, TC368591, TC381957 |
| [Nuclease activity](http://amigo.geneontology.org/cgi-bin/amigo/go.cgi?action=query&view=query&query=GO:0004518&search_constraint=terms) | TC373145, TC373791, TC375864 |
| [Alcohol dehydrogenase (NAD) activity](http://amigo.geneontology.org/cgi-bin/amigo/go.cgi?action=query&view=query&query=GO:0004022&search_constraint=terms) | TC371145, TC383270 |
| [Purine nucleotide binding](http://amigo.geneontology.org/cgi-bin/amigo/go.cgi?action=query&view=query&query=GO:0017076&search_constraint=terms) | CA646741, TC389162, TC375834, TC394796, TC378790, TC423354, TC425957, TC459656, TC449724, TC455515, TC413571, TC394965, TC374240, TC413339, TC369655, TC397994, TC457991, BE585841, TC380882, TC423880, TC371242, TC425690, TC379171, TC370315, TC399352, TC372530, TC405511, TC395723, TC387410, TC376420, TC372167, TC393820, CJ944525, TC394661, TC384553, CK211589, TC369092, TC403588, TC435595, TC374164, TC405041, TC429747, TC394728, TC371641, TC384688, TC387683, TC407076, TC391947, TC398379, TC387064, TC370114, TC409459, TC406807, CJ550278, CD892838, TC395090, TC368591, TC381957 |
| [Serine-type peptidase activity](http://amigo.geneontology.org/cgi-bin/amigo/go.cgi?action=query&view=query&query=GO:0008236&search_constraint=terms) | TC374409, TC382080 |
| [Serine hydrolase activity](http://amigo.geneontology.org/cgi-bin/amigo/go.cgi?action=query&view=query&query=GO:0017171&search_constraint=terms) | TC374409, TC382080 |
| [ATPase activity, coupled to movement of substances](http://amigo.geneontology.org/cgi-bin/amigo/go.cgi?action=query&view=query&query=GO:0043492&search_constraint=terms) | TC384071, TC446092, TC397994, TC386322, TC371242, TC378225, TC377749, TC398379, TC375813 |
| [Aldehyde-lyase activity](http://amigo.geneontology.org/cgi-bin/amigo/go.cgi?action=query&view=query&query=GO:0016832&search_constraint=terms) | TC373613, TC432185 |
| [Protein transporter activity](http://amigo.geneontology.org/cgi-bin/amigo/go.cgi?action=query&view=query&query=GO:0008565&search_constraint=terms) | TC409343, TC383047, TC411784, TC378790, TC412569, TC418928, TC435808, TC425690, TC438243, TC398343, TC387621 |
| [Racemase and epimerase activity](http://amigo.geneontology.org/cgi-bin/amigo/go.cgi?action=query&view=query&query=GO:0016854&search_constraint=terms) | TC380416, TC376490 |
| [Hormone binding](http://amigo.geneontology.org/cgi-bin/amigo/go.cgi?action=query&view=query&query=GO:0042562&search_constraint=terms) | TC380433, TC376420 |
| [Nucleotide binding](http://amigo.geneontology.org/cgi-bin/amigo/go.cgi?action=query&view=query&query=GO:0000166&search_constraint=terms) | CA646741, TC389162, TC375834, TC394796, TC378790, TC423354, TC425957, TC459656, TC449724, TC455515, TC413571, TC394965, TC374240, TC413339, TC369655, TC397994, TC457991, BE585841, TC380882, TC423880, TC371242, TC425690, TC379171, TC370315, TC399352, TC372530, TC405511, TC395723, TC387410, TC376420, TC372167, TC393820, CJ944525, TC394661, TC379536, TC384553, CK211589, TC369092, TC403588, TC435595, TC374164, TC405041, TC429747, TC394728, TC371641, TC384688, TC387683, TC407076, TC391947, TC398379, TC387064, TC370114, TC409459, TC406807, CD892838, CJ550278, TC395090, TC368591, TC396650, TC381957 |
| [Coenzyme binding](http://amigo.geneontology.org/cgi-bin/amigo/go.cgi?action=query&view=query&query=GO:0050662&search_constraint=terms) | TC376490, TC396650 |
| [Phosphoprotein binding](http://amigo.geneontology.org/cgi-bin/amigo/go.cgi?action=query&view=query&query=GO:0051219&search_constraint=terms) | TC438243, TC407978 |
| [Cyclase activity](http://amigo.geneontology.org/cgi-bin/amigo/go.cgi?action=query&view=query&query=GO:0009975&search_constraint=terms) | TC380433, TC417077 |
| [Peptidase activity, acting on L-amino acid peptides](http://amigo.geneontology.org/cgi-bin/amigo/go.cgi?action=query&view=query&query=GO:0070011&search_constraint=terms) | TC370106, TC397033, TC442623, TC397562, TC414899, TC372664, TC445939, CK207939, TC374409, TC370158, TC382080, TC391995, CV780698, TC420420, TC452050 |
| [Endopeptidase activity](http://amigo.geneontology.org/cgi-bin/amigo/go.cgi?action=query&view=query&query=GO:0004175&search_constraint=terms) | TC442623, TC397562, TC414899, TC445939, TC420420, TC452050, TC391995 |
| [Monooxygenase activity](http://amigo.geneontology.org/cgi-bin/amigo/go.cgi?action=query&view=query&query=GO:0004497&search_constraint=terms) | TC427006, TC421162 |
| [Unfolded protein binding](http://amigo.geneontology.org/cgi-bin/amigo/go.cgi?action=query&view=query&query=GO:0051082&search_constraint=terms) | TC405511, TC423354, TC403588, TC379171, TC380882, TC423880 |
| [Protein serine/threonine phosphatase activity](http://amigo.geneontology.org/cgi-bin/amigo/go.cgi?action=query&view=query&query=GO:0004722&search_constraint=terms) | TC378432, TC380125, TC424154, TC429747 |
| [Olfactory receptor activity](http://amigo.geneontology.org/cgi-bin/amigo/go.cgi?action=query&view=query&query=GO:0004984&search_constraint=terms) | TC416906, TC407076, TC398862, TC418414, TC432185, TC438243, TC377061, TC383909, TC449463, TC417388 |
| [Phosphoric ester hydrolase activity](http://amigo.geneontology.org/cgi-bin/amigo/go.cgi?action=query&view=query&query=GO:0042578&search_constraint=terms) | TC399408, TC380125, TC409650, TC392297, TC370114, TC410954, TC459656, TC409459, TC378432, TC406807, TC393970, TC408192, TC424154, TC429747 |
| [DNA bending activity](http://amigo.geneontology.org/cgi-bin/amigo/go.cgi?action=query&view=query&query=GO:0008301&search_constraint=terms) | TC409459, TC370114, TC417077 |
| [Phosphoric diester hydrolase activity](http://amigo.geneontology.org/cgi-bin/amigo/go.cgi?action=query&view=query&query=GO:0008081&search_constraint=terms) | TC409459, TC370114, TC393970, TC408192 |
| [3',5'-cyclic-nucleotide phosphodiesterase activity](http://amigo.geneontology.org/cgi-bin/amigo/go.cgi?action=query&view=query&query=GO:0004114&search_constraint=terms) | TC409459, TC370114, TC393970 |
| [Sequence-specific DNA binding](http://amigo.geneontology.org/cgi-bin/amigo/go.cgi?action=query&view=query&query=GO:0043565&search_constraint=terms) | TC409459, TC370114 |
| [Cofactor binding](http://amigo.geneontology.org/cgi-bin/amigo/go.cgi?action=query&view=query&query=GO:0048037&search_constraint=terms) | TC376490, TC396650 |
| [Enzyme binding](http://amigo.geneontology.org/cgi-bin/amigo/go.cgi?action=query&view=query&query=GO:0019899&search_constraint=terms) | CK211589, TC435808, TC409459, TC370114, TC438243, TC376420, TC413571 |
| [Enzyme inhibitor activity](http://amigo.geneontology.org/cgi-bin/amigo/go.cgi?action=query&view=query&query=GO:0004857&search_constraint=terms) | TC438243, TC407978 |
| [Hydrolase activity, acting on acid anhydrides, catalyzing transmembrane movement of substances](http://amigo.geneontology.org/cgi-bin/amigo/go.cgi?action=query&view=query&query=GO:0016820&search_constraint=terms) | TC377749, TC398379, TC384071, TC375813, TC397994, TC371242, TC378225 |
| [ATPase activity, coupled to transmembrane movement of substances](http://amigo.geneontology.org/cgi-bin/amigo/go.cgi?action=query&view=query&query=GO:0042626&search_constraint=terms) | TC377749, TC398379, TC384071, TC375813, TC397994, TC371242, TC378225 |
| [Calcium ion binding](http://amigo.geneontology.org/cgi-bin/amigo/go.cgi?action=query&view=query&query=GO:0005509&search_constraint=terms) | TC381988, TC405511, TC384194, TC377441, TC404843, TC380433, TC425821, TC370044, TC423354, TC459656, TC403588, TC445166, TC415588, TC429747, TC380882, TC423880, TC410078, TC425690, TC406807, TC393970, TC376220, TC409043, TC416471 |
| [Protein complex binding](http://amigo.geneontology.org/cgi-bin/amigo/go.cgi?action=query&view=query&query=GO:0032403&search_constraint=terms) | TC374230, TC425690, TC377061, TC376420, TC407076, TC382045 |
| [Primary active transmembrane transporter activity](http://amigo.geneontology.org/cgi-bin/amigo/go.cgi?action=query&view=query&query=GO:0015399&search_constraint=terms) | TC384071, TC404158, TC397994, TC371242, TC378225, TC377749, TC398379, TC375813, TC387621 |
| [Zinc ion binding](http://amigo.geneontology.org/cgi-bin/amigo/go.cgi?action=query&view=query&query=GO:0008270&search_constraint=terms) | TC403580, TC383270, TC459245, TC394820, TC416906, TC407978, TC398862, TC418414, TC389092, TC432205, TC394028, TC417388 |
| [Sequence-specific DNA binding RNA polymerase II transcription factor activity](http://amigo.geneontology.org/cgi-bin/amigo/go.cgi?action=query&view=query&query=GO:0000981&search_constraint=terms) | TC409459, TC370114, TC417077 |
| [Sequence-specific enhancer binding RNA polymerase II transcription factor activity](http://amigo.geneontology.org/cgi-bin/amigo/go.cgi?action=query&view=query&query=GO:0003705&search_constraint=terms) | TC409459, TC370114, TC417077 |
| [Peptidase activity](http://amigo.geneontology.org/cgi-bin/amigo/go.cgi?action=query&view=query&query=GO:0008233&search_constraint=terms) | TC370106, TC414899, TC420420, TC377373, TC397033, TC397562, TC442623, TC445939, TC407076, TC372664, TC382080, TC370158, TC374409, CK207939, TC391995, CV780698, TC452050 |
| [Cyclic-nucleotide phosphodiesterase activity](http://amigo.geneontology.org/cgi-bin/amigo/go.cgi?action=query&view=query&query=GO:0004112&search_constraint=terms) | TC409459, TC370114, TC393970 |
| [Isomerase activity](http://amigo.geneontology.org/cgi-bin/amigo/go.cgi?action=query&view=query&query=GO:0016853&search_constraint=terms) | TC376248, TC370885, TC372330, TC391128, TC372654, TC380416, TC417341, TC387007, TC376490, TC402668, TC386646 |
| [Structural constituent of cytoskeleton](http://amigo.geneontology.org/cgi-bin/amigo/go.cgi?action=query&view=query&query=GO:0005200&search_constraint=terms) | TC390630, TC379171, TC393948, TC371242 |
| [NADH dehydrogenase activity](http://amigo.geneontology.org/cgi-bin/amigo/go.cgi?action=query&view=query&query=GO:0003954&search_constraint=terms) | TC388049, TC377190 |
| [Poly-pyrimidine tract binding](http://amigo.geneontology.org/cgi-bin/amigo/go.cgi?action=query&view=query&query=GO:0008187&search_constraint=terms) | TC449724, TC393820 |
| [Poly(U) RNA binding](http://amigo.geneontology.org/cgi-bin/amigo/go.cgi?action=query&view=query&query=GO:0008266&search_constraint=terms) | TC449724, TC393820 |
| [Iron ion binding](http://amigo.geneontology.org/cgi-bin/amigo/go.cgi?action=query&view=query&query=GO:0005506&search_constraint=terms) | TC387135, TC388049, TC388136 |
| [Protein domain specific binding](http://amigo.geneontology.org/cgi-bin/amigo/go.cgi?action=query&view=query&query=GO:0019904&search_constraint=terms) | TC438243, TC376420, TC407978 |
| [Oxidoreductase activity, acting on a sulfur group of donors](http://amigo.geneontology.org/cgi-bin/amigo/go.cgi?action=query&view=query&query=GO:0016667&search_constraint=terms) | TC396083, TC382786 |
| [P-P-bond-hydrolysis-driven transmembrane transporter activity](http://amigo.geneontology.org/cgi-bin/amigo/go.cgi?action=query&view=query&query=GO:0015405&search_constraint=terms) | TC384071, TC397994, TC371242, TC378225, TC377749, TC398379, TC375813, TC387621 |
| [Enzyme regulator activity](http://amigo.geneontology.org/cgi-bin/amigo/go.cgi?action=query&view=query&query=GO:0030234&search_constraint=terms) | TC398606, CK208222, TC407978, TC426743, TC379069, TC380433, TC375530, TC438243, TC377061, TC374164 |
| [Carboxylic acid binding](http://amigo.geneontology.org/cgi-bin/amigo/go.cgi?action=query&view=query&query=GO:0031406&search_constraint=terms) | TC438243, TC407076 |
| [drug binding](http://amigo.geneontology.org/cgi-bin/amigo/go.cgi?action=query&view=query&query=GO:0008144&search_constraint=terms) | TC387007, TC391128 |
| [Hydrolase activity, acting on ester bonds](http://amigo.geneontology.org/cgi-bin/amigo/go.cgi?action=query&view=query&query=GO:0016788&search_constraint=terms) | TC373145, TC380125, TC406749, TC392297, TC410954, TC459656, TC378432, TC375864, TC429747, TC399408, TC409650, TC370114, TC409459, TC373791, TC406807, TC408192, TC393970, TC424154 |
| [ATP-dependent helicase activity](http://amigo.geneontology.org/cgi-bin/amigo/go.cgi?action=query&view=query&query=GO:0008026&search_constraint=terms) | TC425690, TC399352 |
| [Purine NTP-dependent helicase activity](http://amigo.geneontology.org/cgi-bin/amigo/go.cgi?action=query&view=query&query=GO:0070035&search_constraint=terms) | TC425690, TC399352 |
| [Helicase activity](http://amigo.geneontology.org/cgi-bin/amigo/go.cgi?action=query&view=query&query=GO:0004386&search_constraint=terms) | TC425690, TC399352 |
| [Peptide binding](http://amigo.geneontology.org/cgi-bin/amigo/go.cgi?action=query&view=query&query=GO:0042277&search_constraint=terms) | TC376420, TC391128 |
| [Transition metal ion binding](http://amigo.geneontology.org/cgi-bin/amigo/go.cgi?action=query&view=query&query=GO:0046914&search_constraint=terms) | TC403580, TC383270, TC459245, TC416906, TC407978, TC418414, TC420057, TC417388, TC387135, TC394820, TC388049, TC398862, TC389092, TC432205, TC394028, TC388136 |
| [Double-stranded DNA binding](http://amigo.geneontology.org/cgi-bin/amigo/go.cgi?action=query&view=query&query=GO:0003690&search_constraint=terms) | TC409459, TC370114 |
| [Oxidoreductase activity, acting on the aldehyde or oxo group of donors](http://amigo.geneontology.org/cgi-bin/amigo/go.cgi?action=query&view=query&query=GO:0016903&search_constraint=terms) | TC427981, TC407076, TC447801 |
| [Oxidoreductase activity, acting on NADH or NADPH](http://amigo.geneontology.org/cgi-bin/amigo/go.cgi?action=query&view=query&query=GO:0016651&search_constraint=terms) | TC388049, TC369348, TC377190 |
| [Non-membrane spanning protein tyrosine kinase activity](http://amigo.geneontology.org/cgi-bin/amigo/go.cgi?action=query&view=query&query=GO:0004715&search_constraint=terms) | TC376420, TC407076 |
| [Receptor signaling protein tyrosine kinase activity](http://amigo.geneontology.org/cgi-bin/amigo/go.cgi?action=query&view=query&query=GO:0004716&search_constraint=terms) | TC376420, TC407076 |
| [Oxidoreductase activity, acting on the aldehyde or oxo group of donors, NAD or NADP as acceptor](http://amigo.geneontology.org/cgi-bin/amigo/go.cgi?action=query&view=query&query=GO:0016620&search_constraint=terms) | TC427981, TC407076 |
| [Integrin binding](http://amigo.geneontology.org/cgi-bin/amigo/go.cgi?action=query&view=query&query=GO:0005178&search_constraint=terms) | TC376420, TC407076 |
| [Receptor signaling protein activity](http://amigo.geneontology.org/cgi-bin/amigo/go.cgi?action=query&view=query&query=GO:0005057&search_constraint=terms) | TC425690, TC376420, TC407076, TC398592 |
| [Ligase activity](http://amigo.geneontology.org/cgi-bin/amigo/go.cgi?action=query&view=query&query=GO:0016874&search_constraint=terms) | TC381988, TC375834, TC433844, TC404843, TC400362, TC380433, TC425957, TC390285, TC445166, TC376774, TC400181, TC394820, TC371242, TC404413, TC387116, TC410078, TC409459, TC370114, TC387064, TC393960 |
| [Translation initiation factor activity](http://amigo.geneontology.org/cgi-bin/amigo/go.cgi?action=query&view=query&query=GO:0003743&search_constraint=terms) | TC423804, TC369726, TC374392, TC402121 |
| [Peptidyl-prolyl cis-trans isomerase activity](http://amigo.geneontology.org/cgi-bin/amigo/go.cgi?action=query&view=query&query=GO:0003755&search_constraint=terms) | TC387007, TC391128, TC386646 |
| [Cis-trans isomerase activity](http://amigo.geneontology.org/cgi-bin/amigo/go.cgi?action=query&view=query&query=GO:0016859&search_constraint=terms) | TC387007, TC391128, TC386646 |
| [Pyrophosphatase activity](http://amigo.geneontology.org/cgi-bin/amigo/go.cgi?action=query&view=query&query=GO:0016462&search_constraint=terms) | TC389162, GH732878, TC393948, TC386322, TC378790, TC378225, TC390630, TC423354, TC459656, TC413571, TC375539, TC457112, TC397994, BE585841, TC391962, TC380882, TC423880, TC371242, TC425690, TC375813, TC454407, TC399352, TC372530, TC409190, TC405511, TC387410, TC446092, TC376420, CJ944525, CK211589, TC369092, TC403588, TC374164, TC384071, TC418845, TC407076, TC377749, TC418928, TC398379, TC406807 |
| [Hydrolase activity, acting on acid anhydrides](http://amigo.geneontology.org/cgi-bin/amigo/go.cgi?action=query&view=query&query=GO:0016817&search_constraint=terms) | TC389162, GH732878, TC393948, TC386322, TC378790, TC378225, TC390630, TC423354, TC459656, TC413571, TC375539, TC457112, TC397994, BE585841, TC391962, TC380882, TC423880, TC371242, TC425690, TC375813, TC454407, TC399352, TC372530, TC409190, TC405511, TC387410, TC446092, TC376420, CJ944525, CK211589, TC369092, TC403588, TC374164, TC384071, TC418845, TC407076, TC377749, TC418928, TC398379, TC406807 |
| [Hydrolase activity, acting on acid anhydrides, in phosphorus-containing anhydrides](http://amigo.geneontology.org/cgi-bin/amigo/go.cgi?action=query&view=query&query=GO:0016818&search_constraint=terms) | TC389162, GH732878, TC393948, TC386322, TC378790, TC378225, TC390630, TC423354, TC459656, TC413571, TC375539, TC457112, TC397994, BE585841, TC391962, TC380882, TC423880, TC371242, TC425690, TC375813, TC454407, TC399352, TC372530, TC409190, TC405511, TC387410, TC446092, TC376420, CJ944525, CK211589, TC369092, TC403588, TC374164, TC384071, TC418845, TC407076, TC377749, TC418928, TC398379, TC406807 |
| [Hydrolase activity](http://amigo.geneontology.org/cgi-bin/amigo/go.cgi?action=query&view=query&query=GO:0016787&search_constraint=terms) | TC389162, TC373145, TC382830, TC373251, TC441343, CK212850, TC393198, TC378790, TC377308, TC378225, TC406749, TC390630, TC378432, TC375864, TC420420, TC383139, TC397033, TC405295, TC375539, TC452945, TC397994, TC457112, TC369844, TC370158, TC371242, TC375813, TC454407, TC375124, TC370106, TC387410, TC380125, TC388819, CJ944525, CK211589, TC410954, TC374164, TC442623, TC418845, TC397562, TC384071, TC384735, TC372664, TC407076, TC445939, CK207939, TC369199, TC373791, TC409459, TC370114, TC393970, TC408192, TC424154, GH732878, TC393948, TC386322, TC373787, TC393523, TC459656, TC423354, CK162413, TC413571, TC377373, TC382674, TC382080, TC391962, BE585841, TC380882, TC423880, TC384010, TC425690, CV780698, TC399352, TC452050, TC372530, TC405511, TC409190, TC446092, TC376420, TC414899, CJ930688, TC392297, TC381817, TC369092, TC403588, TC429747, TC399408, TC394728, DR740372, TC406106, TC374409, CK201148, TC409650, TC391995, TC377749, TC418928, TC398379, TC387064, TC406807 |
| [Receptor binding](http://amigo.geneontology.org/cgi-bin/amigo/go.cgi?action=query&view=query&query=GO:0005102&search_constraint=terms) | TC384735, CA710880, TC376420, TC407076, TC441343, TC420735, TC370114, TC409459, TC438243, TC382045 |
| [Cation binding](http://amigo.geneontology.org/cgi-bin/amigo/go.cgi?action=query&view=query&query=GO:0043169&search_constraint=terms) | TC403580, TC383270, TC459245, TC416906, TC407978, TC377441, TC425821, TC420057, TC423354, TC459656, TC445166, TC417388, TC415588, TC394820, TC398862, TC380882, TC423880, TC389092, TC432205, TC425690, TC382742, TC376220, TC409043, TC388136, TC381988, TC384194, TC405511, TC404843, TC418414, TC380433, TC370044, TC403588, TC429747, TC387135, TC388049, TC410078, TC406807, TC393970, TC394028, TC416471 |
| [Metal ion binding](http://amigo.geneontology.org/cgi-bin/amigo/go.cgi?action=query&view=query&query=GO:0046872&search_constraint=terms) | TC403580, TC383270, TC459245, TC416906, TC407978, TC377441, TC425821, TC420057, TC423354, TC459656, TC445166, TC417388, TC415588, TC394820, TC398862, TC380882, TC423880, TC389092, TC432205, TC425690, TC382742, TC376220, TC409043, TC388136, TC381988, TC384194, TC405511, TC404843, TC418414, TC380433, TC370044, TC403588, TC429747, TC387135, TC388049, TC410078, TC406807, TC393970, TC394028, TC416471 |
| [Nucleoside-triphosphatase activity](http://amigo.geneontology.org/cgi-bin/amigo/go.cgi?action=query&view=query&query=GO:0017111&search_constraint=terms) | TC389162, TC393948, TC386322, TC378790, TC378225, TC390630, TC423354, TC459656, TC413571, TC397994, BE585841, TC380882, TC423880, TC371242, TC425690, TC375813, TC399352, TC372530, TC409190, TC405511, TC387410, TC446092, TC376420, CJ944525, CK211589, TC369092, TC403588, TC374164, TC384071, TC418845, TC407076, TC377749, TC418928, TC398379, TC406807 |
| [Ion binding](http://amigo.geneontology.org/cgi-bin/amigo/go.cgi?action=query&view=query&query=GO:0043167&search_constraint=terms) | TC403580, TC383270, TC459245, TC416906, TC407978, TC377441, TC425821, TC420057, TC423354, TC459656, TC445166, TC417388, TC415588, TC394820, TC398862, TC380882, TC423880, TC389092, TC432205, TC425690, TC382742, TC376220, TC409043, TC388136, TC381988, TC384194, TC405511, TC404843, TC418414, TC380433, TC370044, TC403588, TC429747, TC387135, TC388049, TC410078, TC406807, TC393970, TC394028, TC416471 |
| [Hydrogen-exporting ATPase activity, phosphorylative mechanism](http://amigo.geneontology.org/cgi-bin/amigo/go.cgi?action=query&view=query&query=GO:0008553&search_constraint=terms) | TC397994, TC371242 |
| [Translation elongation factor activity](http://amigo.geneontology.org/cgi-bin/amigo/go.cgi?action=query&view=query&query=GO:0003746&search_constraint=terms) | TC460689, TC376420 |
| [ATPase activity, coupled to transmembrane movement of ions, phosphorylative mechanism](http://amigo.geneontology.org/cgi-bin/amigo/go.cgi?action=query&view=query&query=GO:0015662&search_constraint=terms) | TC397994, TC371242 |
| [Single-stranded RNA binding](http://amigo.geneontology.org/cgi-bin/amigo/go.cgi?action=query&view=query&query=GO:0003727&search_constraint=terms) | TC402545, TC449724, TC393820 |
| [Ubiquitin-protein ligase activity](http://amigo.geneontology.org/cgi-bin/amigo/go.cgi?action=query&view=query&query=GO:0004842&search_constraint=terms) | TC376774, TC400181, TC394820, TC404413, TC400362, TC370114, TC409459, TC393960 |
| [Small conjugating protein ligase activity](http://amigo.geneontology.org/cgi-bin/amigo/go.cgi?action=query&view=query&query=GO:0019787&search_constraint=terms) | TC376774, TC400181, TC394820, TC404413, TC400362, TC370114, TC409459, TC393960 |
| [Acid-amino acid ligase activity](http://amigo.geneontology.org/cgi-bin/amigo/go.cgi?action=query&view=query&query=GO:0016881&search_constraint=terms) | TC376774, TC400181, TC394820, TC404413, TC400362, TC370114, TC409459, TC393960 |
| [Ligase activity, forming carbon-nitrogen bonds](http://amigo.geneontology.org/cgi-bin/amigo/go.cgi?action=query&view=query&query=GO:0016879&search_constraint=terms) | TC376774, TC400181, TC394820, TC375834, TC433844, TC404413, TC400362, TC370114, TC390285, TC409459, TC393960 |
| [ATPase activity, coupled to transmembrane movement of ions](http://amigo.geneontology.org/cgi-bin/amigo/go.cgi?action=query&view=query&query=GO:0042625&search_constraint=terms) | TC397994, TC371242 |
| [Translation factor activity, nucleic acid binding](http://amigo.geneontology.org/cgi-bin/amigo/go.cgi?action=query&view=query&query=GO:0008135&search_constraint=terms) | TC460689, TC423804, TC369726, TC374392, TC376420, TC402121 |
| [Structure-specific DNA binding](http://amigo.geneontology.org/cgi-bin/amigo/go.cgi?action=query&view=query&query=GO:0043566&search_constraint=terms) | TC409459, TC370114 |
| [GTP binding](http://amigo.geneontology.org/cgi-bin/amigo/go.cgi?action=query&view=query&query=GO:0005525&search_constraint=terms) | TC372530, CK211589, TC387410, TC379171, TC413571, BE585841, TC378790 |
| [Guanyl ribonucleotide binding](http://amigo.geneontology.org/cgi-bin/amigo/go.cgi?action=query&view=query&query=GO:0032561&search_constraint=terms) | TC372530, CK211589, TC387410, TC379171, TC413571, BE585841, TC378790 |
| [Guanyl nucleotide binding](http://amigo.geneontology.org/cgi-bin/amigo/go.cgi?action=query&view=query&query=GO:0019001&search_constraint=terms) | TC372530, CK211589, TC387410, TC379171, TC413571, BE585841, TC378790 |
| [GTPase activity](http://amigo.geneontology.org/cgi-bin/amigo/go.cgi?action=query&view=query&query=GO:0003924&search_constraint=terms) | TC372530, CK211589, TC387410, TC425690, TC376420, TC413571, BE585841, TC378790 |
| [Lyase activity](http://amigo.geneontology.org/cgi-bin/amigo/go.cgi?action=query&view=query&query=GO:0016829&search_constraint=terms) | TC373613, TC369064, TC451519, TC400056, TC373702, TC453487, CK161232, TC455515, TC417077, TC385701, TC388049, TC376351, TC432185, TC380416, TC394028, TC396650 |
| [Electron carrier activity](http://amigo.geneontology.org/cgi-bin/amigo/go.cgi?action=query&view=query&query=GO:0009055&search_constraint=terms) | TC387135, TC387191 |
| [Inorganic cation transmembrane transporter activity](http://amigo.geneontology.org/cgi-bin/amigo/go.cgi?action=query&view=query&query=GO:0022890&search_constraint=terms) | TC429341, TC444402, TC373583, TC397994, TC371242 |
| [Structural constituent of ribosome](http://amigo.geneontology.org/cgi-bin/amigo/go.cgi?action=query&view=query&query=GO:0003735&search_constraint=terms) | TC416906, TC379942, TC407978, TC441343, TC444546, TC417077, TC451511, TC445166, TC417388, TC440066, TC387861, TC389168, TC398862, TC415685, TC409208, TC416442, TC381988, TC421914, TC402545, TC378271, TC431201, TC405475, TC418414, TC380433, CK211589, TC370044, TC435281, TC383909, TC449463, TC388718, TC403328, TC384735, TC372664, TC391948, TC410078, TC370114, TC373994, TC409459, TC381619, TC398970, TC457126, TC376874, TC403580, TC456619, TC435799, TC371970, TC403264, TC459656, TC417769, TC449724, TC413571, CA598430, TC413027, TC368603, TC409599, TC399471, TC425690, TC373259, TC423182, TC410066, TC418091, TC408312, TC391641, TC443814, TC393820, TC404843, TC391613, TC382737, TC400108, TC391785, TC418850, TC375431, TC390489, TC396451, TC406807, TC450285, TC395298 |
| [RNA binding](http://amigo.geneontology.org/cgi-bin/amigo/go.cgi?action=query&view=query&query=GO:0003723&search_constraint=terms) | TC369628, TC403580, TC416906, TC407978, TC441343, TC373429, TC369726, TC459656, TC449724, TC445166, TC417388, TC387861, TC398862, TC387710, TC394118, TC417341, TC423804, CK163367, TC381988, TC408312, TC460689, TC387410, TC376420, TC402545, TC418414, TC404843, TC393820, TC383909, TC449463, TC440819, TC388718, TC384735, TC374392, CA682223, TC388049, TC372664, TC402121, TC375431, TC410078, TC409459, TC370114, TC406807, TC457126, TC404606 |
| [Tetrapyrrole binding](http://amigo.geneontology.org/cgi-bin/amigo/go.cgi?action=query&view=query&query=GO:0046906&search_constraint=terms) | TC387135, TC439472 |
| [Carbon-carbon lyase activity](http://amigo.geneontology.org/cgi-bin/amigo/go.cgi?action=query&view=query&query=GO:0016830&search_constraint=terms) | TC373613, TC455515, TC396650, TC369064, TC385701, TC432185 |
| [Hydrogen ion transmembrane transporter activity](http://amigo.geneontology.org/cgi-bin/amigo/go.cgi?action=query&view=query&query=GO:0015078&search_constraint=terms) | TC397994, TC371242 |
| [Monovalent inorganic cation transmembrane transporter activity](http://amigo.geneontology.org/cgi-bin/amigo/go.cgi?action=query&view=query&query=GO:0015077&search_constraint=terms) | TC397994, TC371242 |
| [Carboxy-lyase activity](http://amigo.geneontology.org/cgi-bin/amigo/go.cgi?action=query&view=query&query=GO:0016831&search_constraint=terms) | TC455515, TC396650, TC369064, TC385701 |
| [Structural molecule activity](http://amigo.geneontology.org/cgi-bin/amigo/go.cgi?action=query&view=query&query=GO:0005198&search_constraint=terms) | TC416906, TC379942, TC407978, TC441343, TC444546, TC390630, TC417077, TC451511, TC445166, TC417388, TC440066, TC387861, TC389168, TC398862, TC371242, TC415685, TC409208, TC375813, TC379171, TC416442, TC381988, TC421914, TC410126, TC402545, TC378271, TC431201, TC405475, TC418414, CJ944525, TC380433, CK211589, TC370044, TC435281, TC383909, TC449463, TC374879, TC388718, TC403328, TC384735, TC372664, TC391948, TC410078, TC370114, TC373994, TC409459, TC381619, TC398970, TC457126, TC376874, TC403580, TC456619, TC393948, TC435799, TC371970, TC403264, TC459656, TC417769, TC449724, TC413571, CA598430, TC413027, TC368603, TC409599, TC399471, TC425690, TC373259, TC423182, TC410066, TC418091, TC408312, TC391641, TC443814, TC393820, TC404843, TC391613, TC382737, TC400108, TC391785, TC418850, TC375431, TC390489, TC396451, TC406807, TC450285, TC395298 |
