# Supplementary material for: Global Analysis of Differentially Expressed Genes and Proteins in the Wheat Callus Infected by Agrobacterium tumefaciens
Source: PLoS One. 2013 Nov 20;8(11):e79390. doi: 10.1371/journal.pone.0079390 (PMC3835833; doi:10.1371/journal.pone.0079390)
Supplement: File S8 — Gene Ontology analysis information for biological process. (DOC) [file pone.0079390.s008.doc]

| **Gene ontology term**  **File S8 Gene ontology analysis information for biological processes** | **Genes annotated to the term** |
| --- | --- |
| [Chromatin assembly or disassembly](http://amigo.geneontology.org/cgi-bin/amigo/go.cgi?action=query&view=query&query=GO:0006333&search_constraint=terms) | TC461921, TC410352, TC386040, TC390393, TC416529, TC397258, TC389190, CV775873, TC396230, TC460760, TC427210, TC416069, BQ838511, TC398052, CA710880, TC400388, TC433162, TC394459, TC416154, TC384738, TC423252, TC397660, TC389363, TC388410, CK203550, TC416493, TC458562, TC406264, TC380063, TC398538, TC410063, TC378054, TC448471, TC395453, TC410194, TC392875, TC435546, TC390569, TC405540, TC419057, TC403872, TC425847, CJ727624, TC398714, BQ294582, CA614761, CJ550278, TC413427, TC411128, TC401915, TC397444, TC398304, TC425878, TC393100, TC433957, TC417012, CA632212, DR739994, TC459193, TC394916, TC394206, TC394820, TC406236, TC418073, TC378274, TC391130, TC417308, CK217367, TC445767, TC446038, TC413460, TC379853, BQ609416, CK214702, TC449504, TC395723, TC405440, CK213497, CJ792862, TC389718, TC404052, TC377225, TC381943, TC412520, TC432001, TC413392, BQ607161, TC402308, TC434820, TC379711, TC400330, CA595837, TC379903, TC423265, TC392272, TC401244, CA730421, TC446465, TC386313, CV759879, TC408907, CA606693, TC391438, CK211707, TC386963, TC412732, TC405695, CA700201, TC392778, TC396751, TC397312, TC405030, TC388403, TC376527, CA615187, TC416695, TC419584, TC383701, TC385780, TC406469, TC405784, TC399919, TC407572, TC372175, TC410690, TC406708, TC419747, TC410074, TC402186, CV763657, TC405356, TC402603, TC441241, TC413043, CA729339, CK214224, TC408299, BQ239045, CA709177, TC376263, TC416169, TC381218, TC408229, TC422425, CA613620, TC398805, CV781430, TC388520, TC411191, TC399342, TC434831, TC426326, TC381462, CD882425, TC373914, TC392263, TC392303, CA611770, TC435533, CA720842, TC425841, CF554444, TC424204, TC387579, TC406870, TC388914, TC393814, TC387621, TC398633, TC394716, TC446235, TC380943, TC393830, TC403885, TC420735, TC404371, TC384219, TC386961, TC416685, TC387344, TC412483, TC389090, TC389375, TC382655, CK200433, TC406038, TC395351, TC418032, CK210754, TC410147, TC384454, TC411287, TC386237, TC393904, TC390994, TC391143, TC381463, TC403157, TC386414, TC395303, TC400755, TC440636, TC403986, TC405615, TC458987, TC404843, TC431879, CD876572, TC385365, TC401210, DR737360, TC379357, TC390135, TC397885, TC386279, TC411480, TC411684, TC380662, TC393960, DR739471, TC402534, TC443387, TC415083, TC386535, GH729256, TC406584, TC386519, CA605200, TC402440, TC417992, TC391411, TC394307, TC388665, TC375146, TC389589, TC452762, TC386344, TC381963, TC383176, CD878039, TC374879, TC386422, TC388688, TC388547, TC414606, TC388822, TC377496, TC388976, TC396365, TC429771, TC423576, TC386639, TC416658, TC393561, TC393692, TC391671, TC393554, TC407614, TC382928, TC379436, TC417363, TC403573, TC403929, TC413700, TC449043, TC379635, TC440321, TC433589, TC377410, TC389816, TC392323, TC413066, CB307332, CK211469, TC404842, TC397729, TC403803, TC396895, TC382342, TC393106, TC401758, TC397676, TC396460, TC432504, TC417067, TC389661, TC428944 |
| [DNA packaging](http://amigo.geneontology.org/cgi-bin/amigo/go.cgi?action=query&view=query&query=GO:0006323&search_constraint=terms) | TC461921, TC410352, TC386040, TC390393, TC416529, TC397258, TC389190, CV775873, TC396230, TC460760, TC427210, TC416069, BQ838511, TC398052, CA710880, TC400388, TC433162, TC394459, TC416154, TC384738, TC423252, TC397660, TC389363, TC388410, CK203550, TC416493, TC458562, TC406264, TC380063, TC398538, TC410063, TC378054, TC448471, TC395453, TC410194, TC392875, TC409843, TC435546, TC390569, TC405540, TC419057, TC403872, TC425847, CJ727624, TC398714, BQ294582, CA614761, CJ550278, TC413427, TC411128, TC401915, TC397444, TC398304, TC425878, TC393100, TC433957, TC417012, CA632212, DR739994, TC459193, TC394916, TC394206, TC394820, TC406236, TC418073, TC378274, TC391130, TC417308, CK217367, TC445767, TC446038, TC413460, TC379853, BQ609416, CK214702, TC449504, TC395723, TC405440, CK213497, CJ792862, TC389718, TC404052, TC377225, TC381943, TC412520, TC432001, TC413392, BQ607161, TC402308, TC434820, TC379711, TC400330, CA595837, TC379903, TC423265, TC392272, TC401244, CA730421, TC446465, TC386313, CV759879, CA606693, TC391438, CK211707, TC386963, TC412732, TC405695, CA700201, TC392778, TC396751, TC397312, TC405030, TC388403, TC376527, CA615187, TC416695, TC419584, TC383701, TC385780, TC406469, TC405784, TC399919, TC407572, TC372175, TC410690, TC406708, TC419747, TC410074, TC402186, CV763657, TC405356, TC402603, TC441241, TC413043, CA729339, TC408299, BQ239045, CA709177, TC376263, TC416169, TC381218, TC408229, TC422425, CA613620, TC398805, CV781430, TC388520, TC411191, TC399342, TC434831, TC426326, TC381462, CD882425, TC373914, TC392263, TC392303, TC394796, CA611770, TC435533, CA720842, TC425841, CF554444, TC424204, TC387579, TC406870, TC388914, TC393814, TC387621, TC398633, TC394716, TC446235, TC380943, TC393830, TC420735, TC403885, TC384219, TC386961, TC416685, TC387344, TC412483, TC389090, TC389375, TC382655, CK200433, TC406038, TC395351, TC418032, CK210754, TC410147, TC384454, TC411287, TC386237, TC393904, TC390994, TC391143, TC381463, TC403157, TC386414, TC395303, TC400755, TC440636, TC403986, TC405615, TC458987, TC404843, TC431879, CD876572, TC385365, TC401210, DR737360, TC390135, TC397885, TC411480, TC411684, TC380662, TC393960, DR739471, TC402534, TC443387, TC415083, TC386535, GH729256, TC406584, TC386519, CA605200, TC402440, TC417992, TC391411, TC394307, TC388665, TC375146, TC389589, TC452762, TC386344, TC381963, TC383176, CD878039, TC386422, TC388688, TC388547, TC414606, TC388822, TC377496, TC388976, TC396365, TC429771, TC423576, TC386639, TC416658, TC393561, TC393692, TC391671, TC393554, TC407614, TC382928, TC379436, TC417363, TC403573, TC403929, TC413700, TC449043, TC379635, TC440321, TC433589, TC377410, TC389816, TC392323, TC413066, CB307332, CK211469, TC404842, TC397729, TC403803, TC396895, TC382342, TC393106, TC401758, TC397676, TC396460, TC432504, TC417067, TC389661, TC428944 |
| [Chromatin assembly](http://amigo.geneontology.org/cgi-bin/amigo/go.cgi?action=query&view=query&query=GO:0031497&search_constraint=terms) | TC461921, TC410352, TC386040, TC390393, TC416529, TC397258, TC389190, CV775873, TC396230, TC460760, TC427210, TC416069, BQ838511, TC398052, CA710880, TC400388, TC433162, TC394459, TC416154, TC384738, TC423252, TC397660, TC389363, TC388410, CK203550, TC416493, TC458562, TC406264, TC380063, TC398538, TC410063, TC378054, TC448471, TC395453, TC410194, TC392875, TC435546, TC390569, TC405540, TC419057, TC403872, TC425847, CJ727624, TC398714, BQ294582, CA614761, CJ550278, TC413427, TC411128, TC401915, TC397444, TC398304, TC425878, TC393100, TC433957, TC417012, CA632212, DR739994, TC459193, TC394916, TC394206, TC394820, TC406236, TC418073, TC378274, TC391130, TC417308, CK217367, TC445767, TC446038, TC413460, TC379853, BQ609416, CK214702, TC449504, TC395723, TC405440, CK213497, CJ792862, TC389718, TC404052, TC377225, TC381943, TC412520, TC432001, TC413392, BQ607161, TC402308, TC434820, TC379711, TC400330, CA595837, TC379903, TC423265, TC392272, TC401244, CA730421, TC446465, TC386313, CV759879, CA606693, TC391438, CK211707, TC386963, TC412732, TC405695, CA700201, TC392778, TC396751, TC397312, TC405030, TC388403, TC376527, CA615187, TC416695, TC419584, TC383701, TC385780, TC406469, TC405784, TC399919, TC407572, TC372175, TC410690, TC406708, TC419747, TC410074, TC402186, CV763657, TC405356, TC402603, TC441241, TC413043, CA729339, TC408299, BQ239045, CA709177, TC376263, TC416169, TC381218, TC408229, TC422425, CA613620, TC398805, CV781430, TC388520, TC411191, TC399342, TC434831, TC426326, TC381462, CD882425, TC373914, TC392263, TC392303, CA611770, TC435533, CA720842, TC425841, CF554444, TC424204, TC387579, TC406870, TC388914, TC393814, TC387621, TC398633, TC394716, TC446235, TC380943, TC393830, TC420735, TC403885, TC384219, TC386961, TC416685, TC387344, TC412483, TC389090, TC389375, TC382655, CK200433, TC406038, TC395351, TC418032, CK210754, TC410147, TC384454, TC411287, TC386237, TC393904, TC390994, TC391143, TC381463, TC403157, TC386414, TC395303, TC400755, TC440636, TC403986, TC405615, TC458987, TC404843, TC431879, CD876572, TC385365, TC401210, DR737360, TC390135, TC397885, TC411480, TC411684, TC380662, TC393960, DR739471, TC402534, TC443387, TC415083, TC386535, GH729256, TC406584, TC386519, CA605200, TC402440, TC417992, TC391411, TC394307, TC388665, TC375146, TC389589, TC452762, TC386344, TC381963, TC383176, CD878039, TC386422, TC388688, TC388547, TC414606, TC388822, TC377496, TC388976, TC396365, TC429771, TC423576, TC386639, TC416658, TC393561, TC393692, TC391671, TC393554, TC407614, TC382928, TC379436, TC417363, TC403573, TC403929, TC413700, TC449043, TC379635, TC440321, TC433589, TC377410, TC389816, TC413066, TC392323, CB307332, CK211469, TC404842, TC397729, TC403803, TC396895, TC382342, TC393106, TC401758, TC397676, TC396460, TC432504, TC417067, TC389661, TC428944 |
| [Nucleosome assembly](http://amigo.geneontology.org/cgi-bin/amigo/go.cgi?action=query&view=query&query=GO:0006334&search_constraint=terms) | TC461921, TC410352, TC386040, TC390393, TC416529, TC397258, TC389190, CV775873, TC396230, TC460760, TC427210, TC416069, BQ838511, TC398052, CA710880, TC400388, TC433162, TC394459, TC416154, TC384738, TC423252, TC397660, TC389363, TC388410, CK203550, TC416493, TC458562, TC406264, TC380063, TC398538, TC410063, TC378054, TC448471, TC395453, TC410194, TC392875, TC435546, TC390569, TC405540, TC419057, TC403872, TC425847, CJ727624, TC398714, BQ294582, CA614761, CJ550278, TC413427, TC411128, TC401915, TC397444, TC398304, TC425878, TC393100, TC433957, TC417012, CA632212, DR739994, TC459193, TC394916, TC394206, TC394820, TC406236, TC418073, TC378274, TC391130, TC417308, CK217367, TC445767, TC446038, TC413460, TC379853, BQ609416, CK214702, TC449504, TC395723, TC405440, CK213497, CJ792862, TC389718, TC404052, TC377225, TC381943, TC412520, TC432001, TC413392, BQ607161, TC402308, TC434820, TC379711, TC400330, CA595837, TC379903, TC423265, TC392272, TC401244, CA730421, TC446465, TC386313, CV759879, CA606693, TC391438, CK211707, TC386963, TC412732, TC405695, CA700201, TC392778, TC396751, TC397312, TC405030, TC388403, TC376527, CA615187, TC416695, TC419584, TC385780, TC406469, TC405784, TC399919, TC407572, TC372175, TC410690, TC406708, TC419747, TC410074, TC402186, CV763657, TC405356, TC402603, TC441241, TC413043, CA729339, TC408299, BQ239045, CA709177, TC376263, TC416169, TC381218, TC408229, TC422425, CA613620, TC398805, CV781430, TC388520, TC411191, TC399342, TC434831, TC426326, TC381462, CD882425, TC373914, TC392263, TC392303, CA611770, TC435533, CA720842, TC425841, CF554444, TC424204, TC387579, TC406870, TC388914, TC393814, TC387621, TC398633, TC394716, TC446235, TC380943, TC393830, TC420735, TC403885, TC384219, TC386961, TC416685, TC387344, TC412483, TC389090, TC389375, TC382655, CK200433, TC406038, TC395351, TC418032, CK210754, TC410147, TC384454, TC411287, TC386237, TC393904, TC390994, TC391143, TC381463, TC403157, TC386414, TC395303, TC400755, TC440636, TC403986, TC405615, TC458987, TC404843, TC431879, CD876572, TC385365, TC401210, DR737360, TC390135, TC397885, TC411480, TC411684, TC380662, TC393960, DR739471, TC402534, TC443387, TC415083, TC386535, GH729256, TC406584, TC386519, CA605200, TC402440, TC417992, TC391411, TC394307, TC388665, TC375146, TC389589, TC452762, TC386344, TC381963, TC383176, CD878039, TC386422, TC388688, TC388547, TC414606, TC388822, TC377496, TC388976, TC396365, TC429771, TC423576, TC386639, TC416658, TC393561, TC393692, TC391671, TC393554, TC407614, TC382928, TC379436, TC417363, TC403573, TC403929, TC413700, TC449043, TC379635, TC440321, TC433589, TC377410, TC389816, TC413066, TC392323, CB307332, CK211469, TC404842, TC397729, TC403803, TC396895, TC382342, TC393106, TC401758, TC397676, TC396460, TC432504, TC417067, TC389661, TC428944 |
| [Nucleosome organization](http://amigo.geneontology.org/cgi-bin/amigo/go.cgi?action=query&view=query&query=GO:0034728&search_constraint=terms) | TC461921, TC410352, TC386040, TC390393, TC416529, TC397258, TC389190, CV775873, TC396230, TC460760, TC427210, TC416069, BQ838511, TC398052, CA710880, TC400388, TC433162, TC394459, TC416154, TC384738, TC423252, TC397660, TC389363, TC388410, CK203550, TC416493, TC458562, TC406264, TC380063, TC398538, TC410063, TC378054, TC448471, TC395453, TC410194, TC392875, TC435546, TC390569, TC405540, TC419057, TC403872, TC425847, CJ727624, TC398714, BQ294582, CA614761, CJ550278, TC413427, TC411128, TC401915, TC397444, TC398304, TC425878, TC393100, TC433957, TC417012, CA632212, DR739994, TC459193, TC394916, TC394206, TC394820, TC406236, TC418073, TC378274, TC391130, TC417308, CK217367, TC445767, TC446038, TC413460, TC379853, BQ609416, CK214702, TC449504, TC395723, TC405440, CK213497, CJ792862, TC389718, TC404052, TC377225, TC381943, TC412520, TC432001, TC413392, BQ607161, TC402308, TC434820, TC379711, TC400330, CA595837, TC379903, TC423265, TC392272, TC401244, CA730421, TC446465, TC386313, CV759879, CA606693, TC391438, CK211707, TC386963, TC412732, TC405695, CA700201, TC392778, TC396751, TC397312, TC405030, TC388403, TC376527, CA615187, TC416695, TC419584, TC385780, TC406469, TC405784, TC399919, TC407572, TC372175, TC410690, TC406708, TC419747, TC410074, TC402186, CV763657, TC405356, TC402603, TC441241, TC413043, CA729339, TC408299, BQ239045, CA709177, TC376263, TC416169, TC381218, TC408229, TC422425, CA613620, TC398805, CV781430, TC388520, TC411191, TC399342, TC434831, TC426326, TC381462, CD882425, TC373914, TC392263, TC392303, CA611770, TC435533, CA720842, TC425841, CF554444, TC424204, TC387579, TC406870, TC388914, TC393814, TC387621, TC398633, TC394716, TC446235, TC380943, TC393830, TC420735, TC403885, TC384219, TC386961, TC416685, TC387344, TC412483, TC389090, TC389375, TC382655, CK200433, TC406038, TC395351, TC418032, CK210754, TC410147, TC384454, TC411287, TC386237, TC393904, TC390994, TC391143, TC381463, TC403157, TC386414, TC395303, TC400755, TC440636, TC403986, TC405615, TC458987, TC404843, TC431879, CD876572, TC385365, TC401210, DR737360, TC390135, TC397885, TC411480, TC411684, TC380662, TC393960, DR739471, TC402534, TC443387, TC415083, TC386535, GH729256, TC406584, TC386519, CA605200, TC402440, TC417992, TC391411, TC394307, TC388665, TC375146, TC389589, TC452762, TC386344, TC381963, TC383176, CD878039, TC386422, TC388688, TC388547, TC414606, TC388822, TC377496, TC388976, TC396365, TC429771, TC423576, TC386639, TC416658, TC393561, TC393692, TC391671, TC393554, TC407614, TC382928, TC379436, TC417363, TC403573, TC403929, TC413700, TC449043, TC379635, TC440321, TC433589, TC377410, TC389816, TC413066, TC392323, CB307332, CK211469, TC404842, TC397729, TC403803, TC396895, TC382342, TC393106, TC401758, TC397676, TC396460, TC432504, TC417067, TC389661, TC428944 |
| [Protein-DNA complex assembly](http://amigo.geneontology.org/cgi-bin/amigo/go.cgi?action=query&view=query&query=GO:0065004&search_constraint=terms) | TC461921, TC410352, TC386040, TC390393, TC416529, TC397258, TC389190, CV775873, TC396230, TC460760, TC427210, TC416069, BQ838511, TC398052, CA710880, TC400388, TC433162, TC394459, TC416154, TC384738, TC423252, TC397660, TC389363, TC388410, CK203550, TC416493, TC458562, TC406264, TC380063, TC398538, TC410063, TC378054, TC448471, TC395453, TC410194, TC392875, TC435546, TC390569, TC405540, TC419057, TC403872, TC425847, CJ727624, TC398714, BQ294582, CA614761, CJ550278, TC413427, TC411128, TC401915, TC397444, TC398304, TC425878, TC393100, TC433957, TC417012, CA632212, DR739994, TC459193, TC394916, TC394206, TC394820, TC406236, TC418073, TC378274, TC391130, TC417308, CK217367, TC445767, TC446038, TC413460, TC379853, BQ609416, CK214702, TC449504, TC395723, TC405440, CK213497, CJ792862, TC389718, TC404052, TC377225, TC381943, TC412520, TC432001, TC413392, BQ607161, TC402308, TC434820, TC379711, TC400330, CA595837, TC379903, TC423265, TC392272, TC401244, CA730421, TC446465, TC386313, CV759879, CA606693, TC391438, CK211707, TC386963, TC412732, TC405695, CA700201, TC392778, TC396751, TC397312, TC405030, TC388403, TC376527, CA615187, TC416695, TC419584, TC385780, TC406469, TC405784, TC399919, TC407572, TC372175, TC410690, TC406708, TC419747, TC410074, TC402186, CV763657, TC405356, TC402603, TC441241, TC413043, CA729339, TC408299, BQ239045, CA709177, TC376263, TC416169, TC381218, TC408229, TC422425, CA613620, TC398805, CV781430, TC388520, TC411191, TC399342, TC434831, TC426326, TC381462, CD882425, TC373914, TC392263, TC392303, CA611770, TC435533, CA720842, TC425841, CF554444, TC424204, TC387579, TC406870, TC388914, TC393814, TC387621, TC398633, TC394716, TC446235, TC380943, TC393830, TC420735, TC403885, TC384219, TC386961, TC416685, TC387344, TC412483, TC389090, TC389375, TC382655, CK200433, TC406038, TC395351, TC418032, CK210754, TC410147, TC384454, TC411287, TC386237, TC393904, TC390994, TC391143, TC381463, TC403157, TC386414, TC395303, TC400755, TC440636, TC403986, TC405615, TC458987, TC404843, TC431879, CD876572, TC385365, TC401210, DR737360, TC390135, TC397885, TC411480, TC411684, TC380662, TC393960, DR739471, TC402534, TC443387, TC415083, TC386535, GH729256, TC406584, TC386519, CA605200, TC402440, TC417992, TC391411, TC394307, TC388665, TC375146, TC389589, TC452762, TC386344, TC381963, TC383176, CD878039, TC386422, TC388688, TC388547, TC414606, TC388822, TC377496, TC388976, TC396365, TC429771, TC423576, TC386639, TC416658, TC393561, TC393692, TC391671, TC393554, TC407614, TC382928, TC379436, TC417363, TC403573, TC403929, TC413700, TC449043, TC379635, TC440321, TC433589, TC377410, TC389816, TC413066, TC392323, CB307332, CK211469, TC404842, TC397729, TC403803, TC396895, TC382342, TC393106, TC401758, TC397676, TC396460, TC432504, TC417067, TC389661, TC428944 |
| [Protein-DNA complex subunit organization](http://amigo.geneontology.org/cgi-bin/amigo/go.cgi?action=query&view=query&query=GO:0071824&search_constraint=terms) | TC461921, TC410352, TC386040, TC390393, TC416529, TC397258, TC389190, CV775873, TC396230, TC460760, TC427210, TC416069, BQ838511, TC398052, CA710880, TC400388, TC433162, TC394459, TC416154, TC384738, TC423252, TC397660, TC389363, TC388410, CK203550, TC416493, TC458562, TC406264, TC380063, TC398538, TC410063, TC378054, TC448471, TC395453, TC410194, TC392875, TC435546, TC390569, TC405540, TC419057, TC403872, TC425847, CJ727624, TC398714, BQ294582, CA614761, CJ550278, TC413427, TC411128, TC401915, TC397444, TC398304, TC425878, TC393100, TC433957, TC417012, CA632212, DR739994, TC459193, TC394916, TC394206, TC394820, TC406236, TC418073, TC378274, TC391130, TC417308, CK217367, TC445767, TC446038, TC413460, TC379853, BQ609416, CK214702, TC449504, TC395723, TC405440, CK213497, CJ792862, TC389718, TC404052, TC377225, TC381943, TC412520, TC432001, TC413392, BQ607161, TC402308, TC434820, TC379711, TC400330, CA595837, TC379903, TC423265, TC392272, TC401244, CA730421, TC446465, TC386313, CV759879, CA606693, TC391438, CK211707, TC386963, TC412732, TC405695, CA700201, TC392778, TC396751, TC397312, TC405030, TC388403, TC376527, CA615187, TC416695, TC419584, TC385780, TC406469, TC405784, TC399919, TC407572, TC372175, TC410690, TC406708, TC419747, TC410074, TC402186, CV763657, TC405356, TC402603, TC441241, TC413043, CA729339, TC408299, BQ239045, CA709177, TC376263, TC416169, TC381218, TC408229, TC422425, CA613620, TC398805, CV781430, TC388520, TC411191, TC399342, TC434831, TC426326, TC381462, CD882425, TC373914, TC392263, TC392303, CA611770, TC435533, CA720842, TC425841, CF554444, TC424204, TC387579, TC406870, TC388914, TC393814, TC387621, TC398633, TC394716, TC446235, TC380943, TC393830, TC420735, TC403885, TC384219, TC386961, TC416685, TC387344, TC412483, TC389090, TC389375, TC382655, CK200433, TC406038, TC395351, TC418032, CK210754, TC410147, TC384454, TC411287, TC386237, TC393904, TC390994, TC391143, TC381463, TC403157, TC386414, TC395303, TC400755, TC440636, TC403986, TC405615, TC458987, TC404843, TC431879, CD876572, TC385365, TC401210, DR737360, TC390135, TC397885, TC411480, TC411684, TC380662, TC393960, DR739471, TC402534, TC443387, TC415083, TC386535, GH729256, TC406584, TC386519, CA605200, TC402440, TC417992, TC391411, TC394307, TC388665, TC375146, TC389589, TC452762, TC386344, TC381963, TC383176, CD878039, TC386422, TC388688, TC388547, TC414606, TC388822, TC377496, TC388976, TC396365, TC429771, TC423576, TC386639, TC416658, TC393561, TC393692, TC391671, TC393554, TC407614, TC382928, TC379436, TC417363, TC403573, TC403929, TC413700, TC449043, TC379635, TC440321, TC433589, TC377410, TC389816, TC413066, TC392323, CB307332, CK211469, TC404842, TC397729, TC403803, TC396895, TC382342, TC393106, TC401758, TC397676, TC396460, TC432504, TC417067, TC389661, TC428944 |
| [DNA conformation change](http://amigo.geneontology.org/cgi-bin/amigo/go.cgi?action=query&view=query&query=GO:0071103&search_constraint=terms) | TC461921, TC410352, TC386040, TC390393, TC416529, TC397258, TC389190, CV775873, TC396230, TC460760, TC427210, TC416069, BQ838511, TC398052, CA710880, TC400388, TC433162, TC394459, TC416154, TC384738, TC423252, TC397660, TC389363, TC388410, CK203550, TC416493, TC458562, TC406264, TC380063, TC398538, TC410063, TC378054, TC448471, TC395453, TC410194, TC392875, TC409843, TC435546, TC390569, TC405540, TC419057, TC403872, TC425847, CJ727624, TC398714, BQ294582, CA614761, CJ550278, TC413427, TC411128, TC401915, TC397444, TC398304, TC425878, TC393100, TC433957, TC417012, CA632212, DR739994, TC459193, TC394916, TC394206, TC394820, TC406236, TC418073, TC378274, TC391130, TC417308, CK217367, TC445767, TC446038, TC413460, TC379853, BQ609416, CK214702, TC449504, TC395723, TC405440, CK213497, CJ792862, TC389718, TC404052, TC377225, TC381943, TC412520, TC432001, TC413392, BQ607161, TC402308, TC434820, TC379711, TC400330, CA595837, TC379903, TC423265, TC392272, TC401244, CA730421, TC446465, TC386313, CV759879, CA606693, TC391438, CK211707, TC386963, TC412732, TC405695, CA700201, TC392778, TC396751, TC397312, TC405030, TC388403, TC376527, CA615187, TC416695, TC419584, TC383701, TC385780, TC406469, TC405784, TC399919, TC407572, TC372175, TC410690, TC406708, TC419747, TC410074, TC402186, CV763657, TC405356, TC402603, TC441241, TC413043, CA729339, TC408299, BQ239045, CA709177, TC376263, TC416169, TC381218, TC408229, TC422425, CA613620, TC398805, CV781430, TC388520, TC411191, TC399342, TC434831, TC426326, TC381462, CD882425, TC373914, TC392263, TC392303, TC394796, CA611770, TC435533, CA720842, TC425841, CF554444, TC424204, TC387579, TC406870, TC388914, TC393814, TC387621, TC398633, TC394716, TC446235, TC380943, TC393830, TC420735, TC403885, TC384219, TC386961, TC416685, TC387344, TC412483, TC389090, TC389375, TC382655, CK200433, TC406038, TC395351, TC418032, CK210754, TC410147, TC384454, TC411287, TC386237, TC393904, TC390994, TC391143, TC381463, TC403157, TC386414, TC395303, TC400755, TC440636, TC403986, TC405615, TC458987, TC404843, TC431879, CD876572, TC385365, TC401210, DR737360, TC390135, TC397885, TC411480, TC411684, TC380662, TC393960, DR739471, TC402534, TC443387, TC415083, TC386535, GH729256, TC406584, TC386519, CA605200, TC402440, TC417992, TC391411, TC394307, TC388665, TC375146, TC389589, TC452762, TC386344, TC381963, TC383176, CD878039, TC386422, TC388688, TC388547, TC414606, TC388822, TC377496, TC388976, TC396365, TC429771, TC423576, TC386639, TC416658, TC393561, TC393692, TC391671, TC393554, TC407614, TC382928, TC379436, TC417363, TC403573, TC403929, TC413700, TC449043, TC379635, TC440321, TC433589, TC377410, TC389816, TC392323, TC413066, CB307332, CK211469, TC404842, TC397729, TC403803, TC396895, TC382342, TC393106, TC401758, TC397676, TC396460, TC432504, TC417067, TC389661, TC428944 |
| [Chromatin organization](http://amigo.geneontology.org/cgi-bin/amigo/go.cgi?action=query&view=query&query=GO:0006325&search_constraint=terms) | TC461921, TC410352, TC386040, TC390393, TC416529, TC397258, TC389190, CV775873, TC396230, TC460760, TC427210, TC416069, BQ838511, TC398052, CA710880, TC400388, TC433162, TC394459, TC416154, TC384738, TC423252, TC397660, TC389363, TC388410, CK203550, TC416493, TC458562, TC406264, TC380063, TC398538, TC410063, TC378054, TC448471, TC395453, TC410194, TC392875, TC435546, TC384735, TC390569, TC405540, TC419057, TC403872, TC425847, CJ727624, TC398714, BQ294582, CA614761, CJ550278, TC413427, TC411128, TC401915, TC397444, TC398304, TC425878, TC393100, TC433957, TC417012, CA632212, DR739994, TC459193, TC394916, TC394206, TC394820, TC406236, TC418073, TC378274, TC391130, TC417308, CK217367, TC409599, TC445767, TC446038, TC413460, TC379853, BQ609416, CK214702, TC449504, TC395723, TC405440, CK213497, CJ792862, TC389718, TC404052, TC377225, TC381943, TC412520, TC432001, TC413392, BQ607161, TC402308, TC434820, TC441343, TC379711, TC400330, CA595837, TC379903, TC423265, TC392272, TC401244, CA730421, TC446465, TC386313, CV759879, TC408907, CA606693, TC391438, CK211707, TC386963, TC412732, TC405695, CA700201, TC392778, TC396751, TC397312, TC405030, CK211589, TC388403, TC376527, CA615187, TC416695, TC419584, TC383701, TC385780, TC406469, TC405784, TC399919, TC407572, TC372175, TC410690, TC406708, TC419747, TC410074, TC402186, TC405356, CV763657, TC402603, TC441241, TC413571, TC413043, CA729339, CK214224, TC408299, BQ239045, CA709177, TC376263, TC416169, TC381218, TC408229, TC422425, CA613620, TC398805, CV781430, TC388520, TC411191, TC399342, TC434831, TC426326, TC381462, CD882425, TC373914, TC392263, TC392303, TC394796, CA611770, TC435533, CA720842, TC425841, CF554444, TC424204, TC387579, TC406870, TC388914, TC393814, TC387621, TC398633, TC394716, TC446235, TC380943, TC393830, TC403885, TC420735, TC404371, TC384219, TC386961, TC416685, TC387344, TC412483, TC389090, TC389375, TC382655, CK200433, TC406038, TC395351, TC418032, CK210754, TC410147, TC384454, TC411287, TC386237, TC393904, TC390994, TC391143, TC381463, TC403157, TC386414, TC395303, TC400755, TC440636, TC403986, TC405615, TC458987, TC404843, TC431879, CD876572, TC385365, TC401210, DR737360, TC379357, TC390135, TC397885, TC386279, TC411480, TC411684, TC380662, TC393960, DR739471, TC402534, TC443387, TC415083, TC386535, GH729256, TC406584, TC386519, CA605200, TC402440, TC417992, TC391411, TC394307, TC388665, TC375146, TC389589, TC452762, TC386344, TC381963, TC383176, CD878039, TC374879, TC386422, TC388688, TC388547, TC414606, TC388822, TC377496, TC388976, TC396365, TC429771, TC423576, TC386639, TC416658, TC393561, TC393692, TC391671, TC393554, TC407614, TC382928, TC379436, TC417363, TC403573, TC403929, TC413700, TC449043, TC379635, TC440321, TC433589, TC377410, TC389816, TC392323, TC413066, CB307332, CK211469, TC404842, TC381817, TC397729, TC403803, TC396895, TC382342, TC393106, TC401758, TC397676, TC396460, TC432504, TC417067, TC389661, TC428944 |
| [Response to bacterium](http://amigo.geneontology.org/cgi-bin/amigo/go.cgi?action=query&view=query&query=GO:0009617&search_constraint=terms) | TC392303, TC386040, TC390393, TC416529, TC397258, CV775873, TC396230, TC435533, TC416069, BQ838511, TC374240, TC398052, TC425841, TC424204, TC433162, TC387579, TC394459, TC416154, TC384738, TC393814, TC388410, TC416493, TC458562, TC394716, TC398633, TC380063, TC398538, TC393830, TC380943, TC410063, TC378054, TC403885, TC404371, TC386961, TC384219, TC410194, TC387344, TC419057, TC412483, TC398714, TC370114, TC418032, TC397444, TC398304, TC393100, TC433957, CK210754, TC417012, CA632212, TC411287, TC386237, TC394916, TC393904, TC390994, TC406236, TC378274, TC391130, TC391143, TC446038, TC434396, TC395303, TC386414, CK214702, TC400755, TC403986, TC405511, TC440636, CK213497, TC458987, TC385365, TC389718, TC401210, TC386707, TC390135, TC379357, TC377225, TC386279, TC411684, TC381943, TC412520, TC380662, TC413392, TC402308, TC402534, TC415083, TC379903, TC423265, TC401244, TC406584, TC386519, TC386313, CV759879, TC408907, CA606693, TC391438, TC417992, CK211707, TC386963, TC398592, TC412732, TC452762, TC405695, TC386344, TC396751, TC405030, TC381963, TC388403, TC383176, CA615187, TC416695, TC385780, TC388547, TC406469, TC405784, TC399919, TC407572, TC372175, TC410690, TC388976, TC396365, TC419747, TC409459, TC386639, TC402186, TC405356, TC393692, TC397415, TC382928, TC402603, TC423354, TC441241, TC379436, TC413043, TC417363, CK214224, TC408299, TC403929, TC376263, TC380882, TC379635, TC423880, TC416169, TC425690, TC377410, TC381218, TC422425, TC392323, TC413066, TC383677, TC461622, CK211469, CB307332, TC404842, TC403588, TC388520, TC397729, TC411191, TC403803, TC399342, TC382342, TC381462, TC401758, TC373914, TC432504, TC392263, TC397676, TC389661 |
| [Cellular macromolecular complex assembly](http://amigo.geneontology.org/cgi-bin/amigo/go.cgi?action=query&view=query&query=GO:0034622&search_constraint=terms) | TC461921, TC410352, TC386040, TC390393, TC416529, TC397258, TC389190, CV775873, TC396230, TC460760, TC427210, TC416069, BQ838511, TC398052, CA710880, TC400388, TC421871, TC433162, TC394459, TC416154, TC384738, TC423252, TC397660, TC389363, TC388410, CK203550, TC416493, TC458562, TC406264, TC380063, TC398538, TC410063, TC378054, TC448471, TC395453, TC410194, TC392875, TC435546, TC390569, TC405540, TC419057, TC403872, TC425847, CJ727624, TC398714, BQ294582, CA614761, CJ550278, TC457126, TC413427, TC411128, TC401915, TC397444, TC398304, TC425878, TC393100, TC433957, TC417012, CA632212, DR739994, TC459193, TC394916, TC394206, TC394820, TC406236, TC418073, TC378274, TC391130, TC417308, CK217367, TC445767, TC446038, TC413460, TC379853, BQ609416, CK214702, TC449504, TC395723, TC405440, CK213497, CJ792862, TC389718, TC404052, TC377225, TC381943, TC412520, TC432001, TC413392, BQ607161, TC382045, TC402308, TC434820, TC379711, TC400330, TC400260, CA595837, TC379903, TC423265, TC392272, TC401244, CA730421, TC446465, TC386313, CV759879, CA606693, TC391438, CK211707, TC386963, TC412732, TC405695, CA700201, TC392778, TC396751, TC397312, TC405030, TC388403, TC376527, CA615187, TC416695, TC388718, TC419584, TC385780, TC406469, TC405784, TC399919, TC407572, TC372175, TC410690, TC406708, TC419747, TC410074, TC402186, CV763657, TC405356, TC402603, TC441241, TC413043, CA729339, TC408299, BQ239045, CA709177, TC376263, TC416169, TC381218, TC408229, TC422425, CA613620, TC398805, CV781430, TC388520, TC411191, TC399342, TC434831, TC426326, TC381462, CD882425, TC373914, TC392263, TC392303, CA611770, TC435533, CA720842, TC425841, CF554444, TC424204, TC387579, TC406870, TC388914, TC393814, TC387621, TC398633, TC394716, TC446235, TC380943, TC393830, TC403885, TC420735, TC384219, TC386961, TC416685, TC372664, TC387344, TC412483, TC389090, TC389375, TC382655, CK200433, TC406038, TC395351, TC418032, CK210754, TC410147, TC384454, TC411287, TC386237, TC393904, TC390994, TC391143, TC381463, TC403157, TC386414, TC395303, TC400755, TC440636, TC403986, TC405615, TC458987, TC404843, TC431879, CD876572, TC385365, TC401210, DR737360, TC390135, TC387135, TC397885, TC411480, TC411684, TC380662, TC393960, DR739471, TC402534, TC443387, TC415083, TC386535, GH729256, TC406584, TC386519, CA605200, TC402440, TC417992, TC391411, TC394307, TC388665, TC375146, TC389589, TC452762, TC386344, TC381963, TC383176, CD878039, TC386422, TC388688, TC388547, TC414606, TC388822, TC377496, TC388976, TC396365, TC429771, TC423576, TC386639, TC416658, TC393561, TC393692, TC391671, TC393554, TC407614, TC382928, TC379436, TC417363, TC403573, TC403929, TC413700, TC449043, TC379635, TC440321, TC433589, TC377410, TC389816, TC392323, TC413066, TC391641, CB307332, CK211469, TC404842, TC397729, TC403803, TC396895, TC382342, TC393106, TC401758, TC397676, TC396460, TC432504, TC417067, TC389661, TC428944 |
| [Cellular macromolecular complex subunit organization](http://amigo.geneontology.org/cgi-bin/amigo/go.cgi?action=query&view=query&query=GO:0034621&search_constraint=terms) | TC461921, TC410352, TC386040, TC390393, TC416529, TC397258, TC389190, CV775873, TC396230, TC460760, TC427210, TC416069, BQ838511, TC398052, CA710880, TC400388, TC421871, TC433162, TC394459, TC416154, TC384738, TC423252, TC397660, TC389363, TC388410, CK203550, TC416493, TC458562, TC406264, TC380063, TC398538, TC410063, TC378054, TC448471, TC395453, TC410194, TC392875, TC435546, TC390569, TC405540, TC419057, TC403872, TC425847, CJ727624, TC398714, BQ294582, CA614761, CJ550278, TC457126, TC413427, TC411128, TC401915, TC397444, TC398304, TC425878, TC393100, TC433957, TC417012, CA632212, DR739994, TC459193, TC394916, TC394206, TC394820, TC406236, TC418073, TC378274, TC391130, TC417308, CK217367, TC445767, TC446038, TC413460, TC379853, BQ609416, CK214702, TC449504, TC395723, TC405440, CK213497, CJ792862, TC389718, TC404052, TC377225, TC381943, TC412520, TC432001, TC413392, BQ607161, TC382045, TC402308, TC434820, TC379711, TC400330, TC400260, CA595837, TC379903, TC423265, TC392272, TC401244, CA730421, TC446465, TC386313, CV759879, CA606693, TC391438, CK211707, TC386963, TC412732, TC405695, CA700201, TC392778, TC396751, TC397312, TC405030, TC388403, TC376527, CA615187, TC416695, TC388718, TC419584, TC385780, TC406469, TC405784, TC399919, TC407572, TC372175, TC410690, TC406708, TC419747, TC410074, TC402186, CV763657, TC405356, TC402603, TC441241, TC413043, CA729339, TC408299, BQ239045, CA709177, TC376263, TC416169, TC381218, TC408229, TC422425, CA613620, TC398805, CV781430, TC388520, TC411191, TC399342, TC434831, TC426326, TC381462, CD882425, TC373914, TC392263, TC392303, CA611770, TC435533, CA720842, TC425841, CF554444, TC424204, TC387579, TC406870, TC388914, TC393814, TC387621, TC398633, TC394716, TC446235, TC380943, TC393830, TC403885, TC420735, TC384219, TC386961, TC416685, TC372664, TC387344, TC412483, TC389090, TC389375, TC382655, CK200433, TC406038, TC395351, TC418032, CK210754, TC410147, TC384454, TC411287, TC386237, TC393904, TC390994, TC391143, TC381463, TC403157, TC386414, TC395303, TC400755, TC440636, TC403986, TC405615, TC458987, TC404843, TC431879, CD876572, TC385365, TC401210, DR737360, TC390135, TC387135, TC397885, TC411480, TC411684, TC380662, TC393960, DR739471, TC402534, TC443387, TC415083, TC386535, GH729256, TC406584, TC386519, CA605200, TC402440, TC417992, TC391411, TC394307, TC388665, TC375146, TC389589, TC452762, TC386344, TC381963, TC383176, CD878039, TC386422, TC388688, TC388547, TC414606, TC388822, TC377496, TC388976, TC396365, TC429771, TC423576, TC386639, TC416658, TC393561, TC393692, TC391671, TC393554, TC407614, TC382928, TC379436, TC417363, TC403573, TC403929, TC413700, TC449043, TC379635, TC440321, TC433589, TC377410, TC389816, TC392323, TC413066, TC391641, CB307332, CK211469, TC404842, TC397729, TC403803, TC396895, TC382342, TC393106, TC401758, TC397676, TC396460, TC432504, TC417067, TC389661, TC428944 |
| [Chromosome organization](http://amigo.geneontology.org/cgi-bin/amigo/go.cgi?action=query&view=query&query=GO:0051276&search_constraint=terms) | TC461921, TC410352, TC386040, TC390393, TC416529, TC397258, TC389190, CV775873, TC396230, TC460760, TC427210, TC416069, BQ838511, TC398052, CA710880, TC400388, TC433162, TC394459, TC371242, TC416154, TC384738, TC423252, TC397660, TC389363, TC379171, TC388410, CK203550, TC416493, TC458562, TC406264, TC380063, TC398538, TC410063, TC378054, TC448471, TC395453, TC410194, TC392875, TC409843, TC435546, TC384735, TC390569, TC405540, TC419057, TC403872, TC425847, CJ727624, TC398714, BQ294582, CA614761, CJ550278, TC403580, TC413427, TC411128, TC401915, TC397444, TC398304, TC425878, TC393100, TC433957, TC417012, CA632212, DR739994, TC459193, TC394916, TC394206, TC394820, TC406236, TC418073, TC378274, TC391130, TC417308, CK217367, TC409599, TC445767, TC446038, TC413460, TC379853, BQ609416, CK214702, TC449504, TC395723, TC405440, CK213497, CJ792862, TC389718, TC404052, TC377225, TC381943, TC412520, TC432001, TC413392, BQ607161, TC402308, TC434820, TC441343, TC379711, TC400330, CA595837, TC379903, TC423265, TC392272, TC401244, CA730421, TC446465, TC386313, CV759879, TC408907, CA606693, TC391438, CK211707, TC386963, TC412732, TC405695, CA700201, TC392778, TC396751, TC397312, TC405030, CK211589, TC388403, TC376527, CA615187, TC416695, TC419584, TC383701, TC385780, TC406469, TC405784, TC399919, TC407572, TC372175, TC410690, TC406708, TC419747, TC410074, TC402186, TC405356, CV763657, TC423354, TC402603, TC441241, TC413571, TC413043, CA729339, CK214224, TC408299, BQ239045, CA709177, TC376263, TC416169, TC381218, TC408229, TC422425, CA613620, TC398805, CV781430, TC388520, TC411191, TC399342, TC434831, TC426326, TC381462, CD882425, TC373914, TC392263, TC392303, TC394796, CA611770, TC435533, CA720842, TC425841, CF554444, TC424204, TC387579, TC406870, TC388914, TC393814, TC387621, TC398633, TC394716, TC446235, TC380943, TC393830, TC403885, TC420735, TC404371, TC384219, TC386961, TC416685, TC387344, TC412483, TC389090, TC389375, TC382655, CK200433, TC406038, TC395351, TC418032, CK210754, TC410147, TC384454, TC411287, TC386237, TC393904, TC390994, TC391143, TC381463, TC403157, TC386414, TC395303, TC400755, TC440636, TC405511, TC403986, TC405615, TC458987, TC404843, TC431879, CD876572, TC385365, TC401210, DR737360, TC379357, TC390135, TC397885, TC386279, TC411480, TC411684, TC380662, TC393960, TC389162, DR739471, TC402534, TC443387, TC415083, TC386535, GH729256, TC406584, TC386519, CA605200, TC402440, TC417992, TC391411, TC394307, TC409208, TC388665, TC375146, TC389589, TC452762, TC386344, TC381963, TC383176, CD878039, TC374879, TC386422, TC388688, TC388547, TC414606, TC388822, TC377496, TC388976, TC396365, TC429771, TC423576, TC386639, TC416658, TC393561, TC393692, TC391671, TC393554, TC407614, TC382928, TC379436, TC417363, TC403573, TC403929, TC413700, TC449043, TC380882, TC379635, TC440321, TC423880, TC433589, TC377410, TC418091, TC389816, TC392323, TC413066, TC461622, CK211469, CB307332, TC404842, TC381817, TC403588, TC397729, TC403803, TC396895, TC382342, TC393106, TC401758, TC432504, TC397676, TC396460, TC417067, TC389661, TC428944 |
| [Defense response](http://amigo.geneontology.org/cgi-bin/amigo/go.cgi?action=query&view=query&query=GO:0006952&search_constraint=terms) | TC392303, TC386040, TC390393, TC416529, TC397258, CV775873, TC391946, TC396230, TC435533, TC416069, BQ838511, TC374240, TC398052, TC425841, TC424204, TC433162, TC387579, TC394459, TC371242, TC416154, TC384738, TC393814, TC388410, TC416493, TC458562, TC394716, TC398633, TC380063, TC398538, TC393830, TC380943, TC410063, TC378054, TC403885, TC404371, TC386961, TC384219, TC410194, TC387344, TC419057, TC412483, TC398714, TC370114, CJ550278, TC418032, TC397444, TC398304, TC393100, TC433957, CK210754, TC417012, TC423239, CA632212, TC411908, TC411287, TC386237, TC394916, TC393904, TC390994, TC406236, TC378274, TC391130, TC391143, TC446038, TC434396, TC398536, TC395303, TC386414, CK214702, TC400755, TC403986, TC395723, TC440636, CK213497, TC458987, TC385365, TC430544, TC370633, TC389718, TC401210, TC386707, TC390135, TC379357, TC377225, TC386279, TC411684, TC392033, CK201148, TC381943, TC439225, TC412520, TC380662, TC413392, TC418928, TC402308, TC402534, TC377308, TC415083, TC379903, TC423265, TC401244, TC406584, TC386519, TC386313, CV759879, TC374726, TC408907, CA606693, TC391438, TC417992, CK211707, TC386963, TC398592, TC412732, TC452762, TC405695, TC386344, TC396751, TC405030, TC381963, TC388403, TC383176, CA615187, TC416695, TC405041, TC452503, TC385780, TC388547, TC406469, TC405784, TC399919, TC407572, TC372175, TC369199, TC410690, TC396365, TC388976, TC419747, TC409459, TC386639, TC402186, TC379965, TC405356, TC393692, TC375726, TC382928, TC402603, TC441241, TC379436, TC397909, TC413043, TC417363, CK214224, TC408299, TC403929, TC376263, TC379635, TC409187, TC384010, TC416169, TC389993, TC425690, TC433589, TC377410, TC381218, TC422425, TC392323, TC413066, TC383677, CK211469, CB307332, TC404842, TC388520, TC397729, TC411191, DR740372, TC403803, TC399342, TC381462, TC382342, TC401758, TC373914, TC432504, TC397676, TC392263, TC389661 |
| [Macromolecular complex assembly](http://amigo.geneontology.org/cgi-bin/amigo/go.cgi?action=query&view=query&query=GO:0065003&search_constraint=terms) | TC461921, TC410352, TC386040, TC390393, TC416529, TC397258, TC389190, CV775873, TC396230, TC460760, TC427210, TC416069, BQ838511, TC398052, CA710880, TC400388, TC421871, TC433162, TC394459, TC416154, TC384738, TC423252, TC397660, TC389363, TC388410, CK203550, TC416493, TC458562, TC406264, TC380063, TC398538, TC410063, TC378054, TC448471, TC395453, TC410194, TC392875, TC435546, TC390569, TC405540, TC407076, TC419057, TC403872, TC425847, CJ727624, TC398714, BQ294582, CA614761, CJ550278, TC457126, TC413427, TC411128, TC401915, TC397444, TC398304, TC425878, TC393100, TC433957, TC417012, CA632212, DR739994, TC459193, TC394916, TC394206, TC394820, TC406236, TC418073, TC378274, TC391130, TC417308, CK217367, TC445767, TC446038, TC413460, TC379853, BQ609416, CK214702, TC449504, TC395723, TC405440, CK213497, CJ792862, TC389718, TC404052, TC377225, TC381943, TC412520, TC432001, TC413392, BQ607161, TC382045, TC402308, TC434820, TC379711, TC400330, TC400260, CA595837, TC379903, TC423265, TC392272, TC401244, CA730421, TC446465, TC386313, CV759879, CA606693, TC391438, CK211707, TC386963, TC412732, TC405695, CA700201, TC392778, TC396751, TC397312, TC405030, TC388403, TC376527, CA615187, TC416695, TC388718, TC419584, TC385780, TC406469, TC405784, TC399919, TC407572, TC372175, TC410690, TC406708, TC419747, TC410074, TC402186, CV763657, TC405356, TC402603, TC441241, TC413043, CA729339, TC408299, BQ239045, CA709177, TC376263, TC416169, TC381218, TC408229, TC422425, CA613620, TC398805, CV781430, TC388520, TC411191, TC399342, TC434831, TC426326, TC381462, CD882425, TC373914, TC392263, TC392303, CA611770, TC435533, CA720842, TC425841, CF554444, TC424204, TC387579, TC406870, TC388914, TC393814, TC387621, TC398633, TC394716, TC446235, TC380943, TC393830, TC403885, TC420735, TC384219, TC386961, TC416685, TC372664, TC387344, TC412483, TC389090, TC389375, TC382655, CK200433, TC406038, TC395351, TC418032, CK210754, TC410147, TC384454, TC411287, TC386237, TC393904, TC390994, TC391143, TC381463, TC403157, TC386414, TC395303, TC400755, TC440636, TC403986, TC405615, TC376420, TC458987, TC404843, TC431879, CD876572, TC385365, TC401210, DR737360, TC390135, TC387135, TC397885, TC411480, TC411684, TC380662, TC393960, DR739471, TC402534, TC443387, TC415083, TC386535, GH729256, TC406584, TC386519, CA605200, TC402440, TC417992, TC391411, TC394307, TC388665, TC375146, TC389589, TC452762, TC386344, TC381963, TC383176, CD878039, TC386422, TC388688, TC388547, TC414606, TC388822, TC377496, TC388976, TC396365, TC429771, TC423576, TC386639, TC416658, TC393561, TC393692, TC391671, TC393554, TC407614, TC382928, TC379436, TC417363, TC403573, TC403929, TC413700, TC449043, TC379635, TC440321, TC433589, TC377410, TC389816, TC392323, TC413066, TC391641, CB307332, CK211469, TC404842, TC397729, TC403803, TC396895, TC382342, TC393106, TC401758, TC397676, TC396460, TC432504, TC417067, TC389661, TC428944 |
| [Cellular component assembly at cellular level](http://amigo.geneontology.org/cgi-bin/amigo/go.cgi?action=query&view=query&query=GO:0071844&search_constraint=terms) | TC461921, TC410352, TC386040, TC390393, TC416529, TC397258, TC389190, CV775873, TC396230, TC460760, TC427210, TC416069, BQ838511, TC398052, CA710880, TC400388, TC421871, TC433162, TC394459, TC416154, TC384738, TC423252, TC397660, TC389363, TC388410, CK203550, TC416493, TC458562, TC421914, TC406264, TC387410, TC380063, TC398538, TC410063, TC378054, TC448471, TC395453, TC410194, TC392875, TC435546, TC390569, TC405540, TC407076, TC419057, TC403872, TC425847, CJ727624, TC398714, BQ294582, CA614761, CJ550278, TC457126, TC413427, TC411128, TC401915, TC397444, TC398304, TC425878, TC393100, TC433957, TC417012, CA632212, DR739994, TC459193, TC394916, TC394206, TC394820, TC406236, TC418073, TC378274, TC391130, TC417308, CK217367, TC445767, TC446038, TC413460, TC379853, BQ609416, CK214702, TC449504, TC395723, TC405440, CK213497, CJ792862, TC389718, TC404052, TC377225, TC381943, TC412520, TC432001, TC413392, BQ607161, TC382045, TC402308, TC434820, TC379711, TC400330, TC400260, CA595837, TC379903, TC423265, TC392272, TC377061, TC401244, CA730421, TC446465, TC386313, CV759879, CA606693, TC391438, CK211707, TC386963, TC412732, TC405695, CA700201, TC392778, TC396751, TC397312, TC405030, TC388403, TC376527, CA615187, TC416695, TC388718, TC419584, TC383701, TC385780, TC406469, TC405784, TC399919, TC407572, TC372175, TC410690, TC406708, TC419747, TC410074, TC402186, TC405356, CV763657, TC402603, TC441241, TC413043, CA729339, TC408299, BQ239045, CA709177, TC376263, TC416169, TC381218, TC408229, TC422425, CA613620, TC398805, CV781430, TC388520, TC411191, TC399342, TC434831, TC426326, TC381462, CD882425, TC373914, TC392263, TC392303, CA611770, TC435533, TC374240, CA720842, TC425841, CF554444, TC424204, TC387579, TC406870, TC388914, TC393814, TC387621, TC398633, TC394716, TC446235, TC378271, TC380943, TC393830, TC403885, TC420735, TC384219, TC386961, TC416685, TC372664, TC387344, TC412483, TC389090, TC389375, TC382655, CK200433, TC406038, TC393970, TC395351, TC418032, CK210754, TC410147, TC384454, TC411287, TC386237, TC393904, TC390994, TC391143, TC381463, TC403157, TC386414, TC395303, TC400755, TC440636, TC403986, TC405615, TC458987, TC404843, TC431879, CD876572, TC385365, TC401210, DR737360, TC390135, TC387135, TC397885, TC411480, TC411684, TC380662, TC393960, DR739471, TC402534, TC443387, TC415083, TC386535, GH729256, TC406584, TC386519, CA605200, TC402440, TC417992, TC391411, TC394307, TC388665, TC375146, TC389589, TC452762, TC386344, TC381963, TC383176, CD878039, TC386422, TC388688, TC418845, TC388547, TC414606, TC388822, TC377496, TC388976, TC396365, TC429771, TC423576, TC386639, TC416658, TC393561, TC393692, TC391671, TC393554, TC407614, TC382928, TC379436, TC417363, TC403573, TC403929, TC413700, TC449043, TC379635, TC440321, TC433589, TC377410, TC389816, TC372530, TC392323, TC413066, TC391641, CK211469, CB307332, TC404842, TC397729, TC403803, TC396895, TC382342, TC393106, TC401758, TC432504, TC397676, TC396460, TC417067, TC389661, TC428944 |
| [DNA repair](http://amigo.geneontology.org/cgi-bin/amigo/go.cgi?action=query&view=query&query=GO:0006281&search_constraint=terms) | TC392303, TC410352, TC386040, TC390393, TC397258, TC375864, TC435533, TC374240, CA710880, TC433162, TC387579, TC371242, TC423252, TC397660, TC393814, TC389363, TC388914, TC458562, TC394716, TC398633, TC446235, TC380063, TC393830, TC380943, TC378054, TC420735, TC404371, TC395453, TC386961, TC384219, TC390569, TC416685, TC419057, TC403872, TC389090, TC389375, TC382655, CK200433, TC398714, BQ294582, TC406038, TC395351, TC401915, CK210754, CA632212, DR739994, TC384454, TC411287, TC394916, TC390994, TC378274, TC391143, CK217367, TC379853, TC370315, TC386414, TC400755, TC405511, TC440636, TC395723, TC405440, TC389718, TC401210, DR737360, TC379357, TC377225, TC386279, TC381943, BQ607161, TC393960, TC389162, DR739471, TC402308, TC434820, TC443387, TC379711, TC379903, TC392272, TC377061, TC386519, CV759879, TC386313, TC408907, TC402440, TC391438, TC394307, TC375146, TC386963, TC389589, TC452762, TC386344, TC392778, TC405030, TC397312, TC381963, TC388403, TC383176, TC386422, TC374164, TC388688, TC385780, TC388822, TC377496, TC388976, TC406708, TC429771, TC423576, TC386639, TC416658, TC402186, TC393561, TC393692, TC391671, TC393554, TC407614, TC402603, TC423354, TC441241, TC413571, TC379436, CA729339, CK214224, TC380882, TC423880, TC433589, TC377410, TC381218, TC389816, CA613620, CK211469, TC403588, TC397729, TC396895, TC434831, TC426326, TC382342, TC381462, TC393106, TC401758, TC373914, TC432504, TC392263, TC397676, TC396460, TC389661, TC428944 |
| [Macromolecular complex subunit organization](http://amigo.geneontology.org/cgi-bin/amigo/go.cgi?action=query&view=query&query=GO:0043933&search_constraint=terms) | TC461921, TC410352, TC386040, TC390393, TC416529, TC397258, TC389190, CV775873, TC396230, TC460760, TC427210, TC416069, BQ838511, TC398052, CA710880, TC400388, TC421871, TC433162, TC394459, TC416154, TC384738, TC423252, TC397660, TC389363, TC388410, CK203550, TC416493, TC458562, TC406264, TC380063, TC398538, TC410063, TC378054, TC448471, TC395453, TC410194, TC392875, TC435546, TC390569, TC405540, TC407076, TC419057, TC403872, TC425847, CJ727624, TC398714, BQ294582, CA614761, CJ550278, TC457126, TC413427, TC411128, TC401915, TC397444, TC398304, TC425878, TC393100, TC433957, TC417012, CA632212, DR739994, TC459193, TC394916, TC394206, TC394820, TC406236, TC418073, TC378274, TC391130, TC417308, CK217367, TC445767, TC446038, TC413460, TC379853, BQ609416, CK214702, TC449504, TC395723, TC405440, CK213497, CJ792862, TC389718, TC404052, TC377225, TC381943, TC412520, TC432001, TC413392, BQ607161, TC382045, TC402308, TC434820, TC379711, TC400330, TC400260, CA595837, TC379903, TC423265, TC392272, TC401244, CA730421, TC446465, TC386313, CV759879, CA606693, TC391438, CK211707, TC386963, TC412732, TC405695, CA700201, TC392778, TC396751, TC397312, TC405030, TC388403, TC376527, CA615187, TC416695, TC388718, TC419584, TC385780, TC406469, TC405784, TC399919, TC407572, TC372175, TC410690, TC406708, TC419747, TC410074, TC402186, CV763657, TC405356, TC402603, TC441241, TC413043, CA729339, TC408299, BQ239045, CA709177, TC376263, TC416169, TC381218, TC408229, TC422425, CA613620, TC398805, CV781430, TC388520, TC411191, TC399342, TC434831, TC426326, TC381462, CD882425, TC373914, TC392263, TC392303, CA611770, TC435533, CA720842, TC425841, CF554444, TC424204, TC387579, TC406870, TC388914, TC393814, TC387621, TC398633, TC394716, TC446235, TC380943, TC393830, TC403885, TC420735, TC384219, TC386961, TC416685, TC372664, TC387344, TC412483, TC389090, TC389375, TC382655, CK200433, TC406038, TC395351, TC418032, CK210754, TC410147, TC384454, TC411287, TC386237, TC393904, TC390994, TC391143, TC381463, TC403157, TC386414, TC395303, TC400755, TC440636, TC403986, TC405615, TC376420, TC458987, TC404843, TC431879, CD876572, TC385365, TC401210, DR737360, TC390135, TC387135, TC397885, TC411480, TC411684, TC380662, TC393960, DR739471, TC402534, TC443387, TC415083, TC386535, GH729256, TC406584, TC386519, CA605200, TC402440, TC417992, TC391411, TC394307, TC388665, TC375146, TC389589, TC452762, TC386344, TC381963, TC383176, CD878039, TC386422, TC388688, TC388547, TC414606, TC388822, TC377496, TC388976, TC396365, TC429771, TC423576, TC386639, TC416658, TC393561, TC393692, TC391671, TC393554, TC407614, TC382928, TC379436, TC417363, TC403573, TC403929, TC413700, TC449043, TC379635, TC440321, TC433589, TC377410, TC389816, TC392323, TC413066, TC391641, CB307332, CK211469, TC404842, TC397729, TC403803, TC396895, TC382342, TC393106, TC401758, TC397676, TC396460, TC432504, TC417067, TC389661, TC428944 |
| [Response to other organism](http://amigo.geneontology.org/cgi-bin/amigo/go.cgi?action=query&view=query&query=GO:0051707&search_constraint=terms) | TC392303, TC404158, TC386040, TC390393, TC416529, TC397258, CV775873, TC396230, TC435533, TC416069, BQ838511, TC374240, TC398052, TC425841, TC424204, TC433162, TC387579, TC394459, TC416154, TC384738, TC404978, TC393814, TC388410, TC416493, TC458562, TC394716, TC398633, TC380063, TC398538, TC393830, TC380943, TC410063, TC378054, TC403885, TC404371, TC386961, TC384219, TC410194, TC387344, TC407076, TC419057, TC412483, TC398714, TC370114, TC418032, TC397444, TC398304, TC393100, TC433957, CK210754, TC417012, TC393523, CA632212, TC411287, TC386237, TC394916, TC393904, TC390994, TC406236, TC378274, TC391130, TC391143, TC446038, TC434396, TC395303, TC386414, CK214702, TC400755, TC403986, TC405511, TC440636, CK213497, TC458987, TC385365, TC389718, TC401210, TC386707, TC390135, TC379357, TC377225, TC386279, TC411684, CK201148, TC381943, TC412520, TC380662, TC413392, TC402308, TC402534, TC377308, TC415083, TC379903, TC423265, TC377061, TC401244, TC406584, TC386519, TC386313, CV759879, TC408907, CA606693, TC391438, TC417992, CK211707, TC386963, TC398592, TC412732, TC452762, TC405695, TC386344, TC396751, TC405030, TC381963, TC388403, TC383176, CA615187, TC416695, TC405041, TC384071, TC452503, TC385780, TC388547, TC406469, TC405784, TC399919, TC407572, TC372175, TC369199, TC410690, TC396365, TC388976, TC419747, TC409459, TC386639, TC402186, TC405356, TC393692, TC373787, TC397415, TC382928, TC402603, TC423354, TC441241, TC449724, TC379436, TC413043, TC417363, CK214224, TC408299, TC403929, TC376263, TC380882, TC423880, TC379635, TC429374, TC384010, TC416169, TC425690, TC377410, TC381218, TC422425, TC392323, TC413066, TC383677, TC393820, TC461622, CK211469, CB307332, TC404842, TC403588, TC388520, TC376933, TC397729, TC411191, DR740372, TC403803, TC399342, TC381462, TC382342, TC401758, TC373914, TC432504, TC392263, TC397676, TC389661 |
| [Response to DNA damage stimulus](http://amigo.geneontology.org/cgi-bin/amigo/go.cgi?action=query&view=query&query=GO:0006974&search_constraint=terms) | TC392303, TC410352, TC386040, TC407978, TC390393, TC397258, TC375864, TC435533, TC374240, CA710880, TC433162, TC387579, TC371242, TC423252, TC397660, TC393814, TC389363, TC388914, TC458562, TC394716, TC398633, TC446235, TC380063, TC393830, TC380943, TC378054, TC420735, TC404371, TC395453, TC386961, TC384219, TC390569, TC416685, TC419057, TC403872, TC389090, TC389375, TC382655, CK200433, TC398714, BQ294582, TC406038, TC395351, TC401915, TC456619, CK210754, CA632212, DR739994, TC384454, TC411287, TC394916, TC390994, TC378274, TC391143, CK217367, TC379853, TC370315, TC386414, TC400755, TC405511, TC440636, TC395723, TC405440, TC389718, TC401210, DR737360, TC379357, TC377225, TC386279, TC381943, BQ607161, TC393960, TC382045, TC389162, DR739471, TC402308, TC434820, TC443387, TC379711, TC379903, TC392272, TC377061, TC386519, CV759879, TC386313, TC408907, TC402440, TC391438, TC394307, TC375146, TC386963, TC389589, TC452762, TC386344, TC392778, TC405030, TC397312, TC381963, TC388403, TC383176, TC386422, TC374164, TC388688, TC385780, TC388822, TC377496, TC388976, TC406708, TC429771, TC423576, TC386639, TC402186, TC416658, TC393561, TC393692, TC391671, TC393554, TC407614, TC402603, TC423354, TC441241, TC438243, TC413571, TC379436, CA729339, CK214224, TC380882, TC423880, TC433589, TC377410, TC381218, TC389816, CA613620, CK211469, TC403588, TC397729, TC396895, TC434831, TC426326, TC382342, TC381462, TC393106, TC401758, TC373914, TC432504, TC392263, TC397676, TC396460, TC389661, TC428944 |
| [Negative regulation of transcription, DNA-dependent](http://amigo.geneontology.org/cgi-bin/amigo/go.cgi?action=query&view=query&query=GO:0045892&search_constraint=terms) | TC392303, TC410352, TC386040, TC390393, TC397258, TC435533, TC374240, CA710880, TC398862, TC433162, TC387579, TC423252, TC397660, TC393814, TC389363, TC388914, TC458562, TC394716, TC398633, TC446235, TC380063, TC393830, TC380943, TC418414, TC378054, TC420735, TC404371, TC395453, TC386961, TC384219, TC390569, TC416685, TC419057, TC403872, TC389090, TC389375, TC404413, TC382655, CK200433, TC398714, TC370114, BQ294582, TC406038, CJ550278, TC395351, TC401915, CK210754, CA632212, DR739994, TC384454, TC411287, TC394916, TC390994, TC378274, TC391143, CK217367, TC379853, TC386414, TC400755, TC440636, TC395723, TC405440, TC389718, TC401210, DR737360, TC379357, TC377225, TC386279, TC381943, BQ607161, TC406807, TC393960, DR739471, TC416906, TC402308, TC434820, TC443387, TC379711, TC379903, TC392272, TC417388, TC386519, TC386313, CV759879, TC408907, TC402440, TC391438, TC394307, TC375146, TC386963, TC389589, TC452762, TC386344, TC392778, TC405030, TC397312, TC381963, TC388403, TC383176, TC386422, TC388688, TC385780, TC388822, TC377496, TC388976, TC406708, TC429771, TC409459, TC423576, TC386639, TC416658, TC402186, TC393561, TC393692, TC391671, TC393554, TC407614, TC402603, TC459656, TC441241, TC379436, CA729339, TC460615, CK214224, TC433589, TC377410, TC381218, TC389816, CA613620, CK211469, TC400362, TC397729, TC396895, TC434831, TC426326, TC418850, TC382342, TC381462, TC393106, TC401758, TC373914, TC432504, TC392263, TC397676, TC396460, TC389661, TC428944 |
| [Cellular component assembly](http://amigo.geneontology.org/cgi-bin/amigo/go.cgi?action=query&view=query&query=GO:0022607&search_constraint=terms) | TC461921, TC410352, TC386040, TC390393, TC416529, TC397258, TC389190, CV775873, TC396230, TC460760, TC427210, TC416069, BQ838511, TC398052, CA710880, TC400388, TC421871, TC433162, TC394459, TC416154, TC384738, TC423252, TC397660, TC389363, TC388410, CK203550, TC416493, TC458562, TC421914, TC406264, TC387410, TC380063, TC398538, TC410063, TC378054, TC448471, TC395453, TC410194, TC392875, TC435546, TC390569, TC405540, TC407076, TC419057, TC403872, TC425847, CJ727624, TC398714, BQ294582, CA614761, CJ550278, TC457126, TC413427, TC411128, TC401915, TC397444, TC398304, TC425878, TC393100, TC433957, TC417012, CA632212, DR739994, TC459193, TC394916, TC394206, TC394820, TC406236, TC418073, TC378274, TC391130, TC417308, CK217367, TC445767, TC446038, TC413460, TC379853, BQ609416, CK214702, TC449504, TC395723, TC405440, CK213497, CJ792862, TC389718, TC404052, TC377225, TC381943, TC412520, TC432001, TC413392, BQ607161, TC382045, TC402308, TC434820, TC379711, TC400330, TC400260, CA595837, TC379903, TC423265, TC392272, TC377061, TC401244, CA730421, TC446465, TC386313, CV759879, CA606693, TC391438, CK211707, TC386963, TC412732, TC405695, CA700201, TC392778, TC396751, TC397312, TC405030, TC388403, TC376527, CA615187, TC416695, TC388718, TC419584, TC383701, TC385780, TC406469, TC405784, TC399919, TC407572, TC372175, TC410690, TC406708, TC419747, TC410074, TC402186, TC405356, CV763657, TC402603, TC441241, TC413043, CA729339, TC408299, BQ239045, CA709177, TC376263, TC416169, TC381218, TC408229, TC422425, CA613620, TC398805, CV781430, TC388520, TC411191, TC399342, TC434831, TC426326, TC381462, CD882425, TC373914, TC392263, TC392303, CA611770, TC435533, TC374240, CA720842, TC425841, CF554444, TC424204, TC387579, TC406870, TC388914, TC393814, TC387621, TC398633, TC394716, TC446235, TC378271, TC380943, TC393830, TC403885, TC420735, TC384219, TC386961, TC416685, TC372664, TC387344, TC412483, TC389090, TC389375, TC382655, CK200433, TC406038, TC393970, TC395351, TC418032, CK210754, TC410147, TC384454, TC411287, TC386237, TC393904, TC390994, TC391143, TC381463, TC403157, TC386414, TC395303, TC400755, TC440636, TC403986, TC405615, TC376420, TC458987, TC404843, TC431879, CD876572, TC385365, TC401210, DR737360, TC390135, TC387135, TC397885, TC411480, TC411684, TC380662, TC393960, DR739471, TC402534, TC443387, TC415083, TC386535, GH729256, TC406584, TC386519, CA605200, TC402440, TC417992, TC391411, TC394307, TC388665, TC375146, TC389589, TC452762, TC386344, TC381963, TC383176, CD878039, TC386422, TC388688, TC418845, TC388547, TC414606, TC388822, TC377496, TC388976, TC396365, TC429771, TC423576, TC386639, TC416658, TC393561, TC393692, TC391671, TC393554, TC407614, TC382928, TC379436, TC417363, TC403573, TC403929, TC413700, TC449043, TC379635, TC440321, TC433589, TC377410, TC389816, TC372530, TC392323, TC413066, TC391641, CK211469, CB307332, TC404842, TC397729, TC403803, TC396895, TC382342, TC393106, TC401758, TC432504, TC397676, TC396460, TC417067, TC389661, TC428944 |
| [Negative regulation of RNA metabolic process](http://amigo.geneontology.org/cgi-bin/amigo/go.cgi?action=query&view=query&query=GO:0051253&search_constraint=terms) | TC392303, TC410352, TC386040, TC390393, TC397258, TC435533, TC374240, CA710880, TC398862, TC433162, TC387579, TC423252, TC397660, TC393814, TC389363, TC388914, TC458562, TC394716, TC398633, TC446235, TC380063, TC393830, TC380943, TC418414, TC380433, TC378054, TC420735, TC404371, TC395453, TC386961, TC384219, TC390569, TC416685, TC419057, TC403872, TC389090, TC389375, TC404413, TC382655, CK200433, TC398714, TC370114, BQ294582, TC406038, CJ550278, TC395351, TC401915, CK210754, CA632212, DR739994, TC384454, TC411287, TC394916, TC390994, TC378274, TC391143, CK217367, TC379853, TC386414, TC400755, TC440636, TC395723, TC405440, TC389718, TC401210, DR737360, TC379357, TC377225, TC386279, TC381943, BQ607161, TC406807, TC393960, DR739471, TC416906, TC402308, TC434820, TC443387, TC379711, TC379903, TC392272, TC417388, TC386519, TC386313, CV759879, TC408907, TC402440, TC391438, TC394307, TC375146, TC386963, TC389589, TC452762, TC386344, TC392778, TC405030, TC397312, TC381963, TC388403, TC383176, TC386422, TC388688, TC385780, TC388822, TC377496, TC388976, TC406708, TC429771, TC409459, TC423576, TC386639, TC402186, TC416658, TC393561, TC393692, TC391671, TC393554, TC407614, TC402603, TC459656, TC441241, TC379436, CA729339, TC460615, CK214224, TC433589, TC377410, TC381218, TC389816, CA613620, CK211469, TC400362, TC397729, TC396895, TC434831, TC426326, TC418850, TC382342, TC381462, TC393106, TC401758, TC373914, TC432504, TC392263, TC397676, TC396460, TC389661, TC428944 |
| [Negative regulation of gene expression](http://amigo.geneontology.org/cgi-bin/amigo/go.cgi?action=query&view=query&query=GO:0010629&search_constraint=terms) | TC392303, TC410352, TC386040, TC390393, TC397258, TC435533, TC374240, CA710880, TC398862, TC433162, TC387579, TC423252, TC397660, TC393814, TC389363, TC388914, TC458562, TC394716, TC398633, TC446235, TC380063, TC393830, TC380943, TC418414, TC378054, TC420735, TC404371, TC395453, TC386961, TC384219, TC390569, TC416685, TC419057, TC403872, TC389090, TC389375, TC404413, TC382655, CK200433, TC398714, TC370114, BQ294582, TC406038, CJ550278, TC395351, TC401915, CK210754, CA632212, DR739994, TC384454, TC411287, TC394916, TC390994, TC378274, TC391143, CK217367, TC379853, TC386414, TC400755, TC440636, TC395723, TC405440, TC377190, TC389718, TC401210, DR737360, TC379357, TC377225, TC386279, TC381943, BQ607161, TC406807, TC393960, DR739471, TC416906, TC402308, TC434820, TC443387, TC379711, TC379903, TC392272, TC417388, TC386519, TC386313, CV759879, TC408907, TC402440, TC391438, TC394307, TC375146, TC386963, TC389589, TC452762, TC386344, TC392778, TC405030, TC397312, TC381963, TC388403, TC383176, TC386422, TC388688, TC385780, TC388822, TC377496, TC388976, TC406708, TC429771, TC409459, TC423576, TC386639, TC402186, TC416658, TC393561, TC393692, TC391671, TC393554, TC407614, TC402603, TC459656, TC441241, TC379436, CA729339, TC460615, CK214224, TC425690, TC433589, TC377410, TC381218, TC389816, CA613620, CK211469, TC400362, TC397729, TC396895, TC434831, TC426326, TC418850, TC382342, TC381462, TC393106, TC401758, TC373914, TC432504, TC392263, TC397676, TC396460, TC389661, TC428944 |
| [Negative regulation of transcription](http://amigo.geneontology.org/cgi-bin/amigo/go.cgi?action=query&view=query&query=GO:0016481&search_constraint=terms) | TC392303, TC410352, TC386040, TC390393, TC397258, TC435533, TC374240, CA710880, TC398862, TC433162, TC387579, TC423252, TC397660, TC393814, TC389363, TC388914, TC458562, TC394716, TC398633, TC446235, TC380063, TC393830, TC380943, TC418414, TC378054, TC420735, TC404371, TC395453, TC386961, TC384219, TC390569, TC416685, TC419057, TC403872, TC389090, TC389375, TC404413, TC382655, CK200433, TC398714, TC370114, BQ294582, TC406038, CJ550278, TC395351, TC401915, CK210754, CA632212, DR739994, TC384454, TC411287, TC394916, TC390994, TC378274, TC391143, CK217367, TC379853, TC386414, TC400755, TC440636, TC395723, TC405440, TC389718, TC401210, DR737360, TC379357, TC377225, TC386279, TC381943, BQ607161, TC406807, TC393960, DR739471, TC416906, TC402308, TC434820, TC443387, TC379711, TC379903, TC392272, TC417388, TC386519, TC386313, CV759879, TC408907, TC402440, TC391438, TC394307, TC375146, TC386963, TC389589, TC452762, TC386344, TC392778, TC405030, TC397312, TC381963, TC388403, TC383176, TC386422, TC388688, TC385780, TC388822, TC377496, TC388976, TC406708, TC429771, TC409459, TC423576, TC386639, TC402186, TC416658, TC393561, TC393692, TC391671, TC393554, TC407614, TC402603, TC459656, TC441241, TC379436, CA729339, TC460615, CK214224, TC425690, TC433589, TC377410, TC381218, TC389816, CA613620, CK211469, TC400362, TC397729, TC396895, TC434831, TC426326, TC418850, TC382342, TC381462, TC393106, TC401758, TC373914, TC432504, TC392263, TC397676, TC396460, TC389661, TC428944 |
| [Negative regulation of cellular process](http://amigo.geneontology.org/cgi-bin/amigo/go.cgi?action=query&view=query&query=GO:0048523&search_constraint=terms) | TC392303, TC410352, TC386040, TC390393, TC394796, TC397258, CV775873, TC435533, TC374240, TC398052, CA710880, TC398862, TC433162, TC387579, TC394459, TC371242, TC384738, TC423252, TC397660, TC393814, TC389363, TC388914, TC388410, TC458562, TC394716, TC398633, TC446235, TC380063, TC393830, TC380943, TC418414, TC380433, TC378054, TC420735, TC404371, TC395453, TC386961, TC384219, TC392329, TC384735, TC390569, TC416685, TC387344, TC419057, TC403872, TC389090, TC389375, TC404413, TC382655, CK200433, TC398714, TC370114, BQ294582, TC406038, CJ550278, TC395351, TC401915, TC398304, TC393100, TC433957, CK210754, CA632212, DR739994, TC384454, TC411287, TC386237, TC394916, TC393904, TC390994, TC378274, TC391143, CK217367, TC379853, TC395303, TC386414, TC400755, TC405511, TC395723, TC440636, TC405440, TC377190, TC389718, TC401210, TC390135, DR737360, TC379357, TC377225, TC386279, TC381943, TC380662, BQ607161, TC406807, TC393960, TC382045, DR739471, TC416906, TC402308, TC434820, TC443387, TC441343, TC379711, TC379903, TC392272, TC417388, TC386519, TC386313, CV759879, TC408907, TC402440, TC420579, TC391438, TC394307, CK211707, TC375146, TC386963, TC389589, TC452762, TC386344, TC392778, TC396751, TC397312, TC405030, TC381963, CK211589, TC388403, TC383176, CA615187, TC386422, TC388688, TC405041, TC385780, TC388547, TC405784, TC399919, TC407572, TC372175, TC388822, TC396365, TC377496, TC388976, TC406708, TC419747, TC429771, TC409459, TC423576, TC386639, TC402186, TC416658, TC393561, TC393692, TC391671, TC393554, TC407614, TC459656, TC382928, TC402603, TC423354, TC441241, TC438243, TC413571, TC379436, CA729339, TC460615, CK214224, TC403929, TC380882, TC423880, TC425690, TC433589, TC377410, TC381218, TC389816, TC422425, CA613620, TC400056, CK211469, TC400362, TC381817, TC403588, TC388520, TC397729, TC411191, TC434831, TC396895, TC426326, TC381462, TC418850, TC382342, TC393106, TC401758, TC373914, TC432504, TC397676, TC396460, TC392263, TC428944, TC389661 |
| [Negative regulation of nucleobase, nucleoside, nucleotide and nucleic acid metabolic process](http://amigo.geneontology.org/cgi-bin/amigo/go.cgi?action=query&view=query&query=GO:0045934&search_constraint=terms) | TC392303, TC410352, TC386040, TC390393, TC397258, TC435533, TC374240, CA710880, TC398862, TC433162, TC387579, TC423252, TC397660, TC393814, TC389363, TC388914, TC458562, TC394716, TC398633, TC446235, TC380063, TC393830, TC380943, TC418414, TC380433, TC378054, TC420735, TC404371, TC395453, TC386961, TC384219, TC390569, TC416685, TC419057, TC403872, TC389090, TC389375, TC404413, TC382655, CK200433, TC398714, TC370114, BQ294582, TC406038, CJ550278, TC395351, TC401915, CK210754, CA632212, DR739994, TC384454, TC411287, TC394916, TC390994, TC378274, TC391143, CK217367, TC379853, TC386414, TC400755, TC440636, TC395723, TC405440, TC389718, TC401210, DR737360, TC379357, TC377225, TC386279, TC381943, BQ607161, TC406807, TC393960, TC382045, DR739471, TC416906, TC402308, TC434820, TC443387, TC379711, TC379903, TC392272, TC417388, TC386519, TC386313, CV759879, TC408907, TC402440, TC391438, TC394307, TC375146, TC386963, TC389589, TC452762, TC386344, TC392778, TC405030, TC397312, TC381963, TC388403, TC383176, TC386422, TC388688, TC385780, TC388822, TC377496, TC388976, TC406708, TC429771, TC409459, TC423576, TC386639, TC402186, TC416658, TC393561, TC393692, TC391671, TC393554, TC407614, TC402603, TC459656, TC441241, TC379436, CA729339, TC460615, CK214224, TC425690, TC433589, TC377410, TC381218, TC389816, CA613620, CK211469, TC400362, TC397729, TC396895, TC434831, TC426326, TC418850, TC382342, TC381462, TC393106, TC401758, TC373914, TC432504, TC392263, TC397676, TC396460, TC389661, TC428944 |
| [Negative regulation of nitrogen compound metabolic process](http://amigo.geneontology.org/cgi-bin/amigo/go.cgi?action=query&view=query&query=GO:0051172&search_constraint=terms) | TC392303, TC410352, TC386040, TC390393, TC397258, TC435533, TC374240, CA710880, TC398862, TC433162, TC387579, TC423252, TC397660, TC393814, TC389363, TC388914, TC458562, TC394716, TC398633, TC446235, TC380063, TC393830, TC380943, TC418414, TC380433, TC378054, TC420735, TC404371, TC395453, TC386961, TC384219, TC390569, TC416685, TC419057, TC403872, TC389090, TC389375, TC404413, TC382655, CK200433, TC398714, TC370114, BQ294582, TC406038, CJ550278, TC395351, TC401915, CK210754, CA632212, DR739994, TC384454, TC411287, TC394916, TC390994, TC378274, TC391143, CK217367, TC379853, TC386414, TC400755, TC440636, TC395723, TC405440, TC389718, TC401210, DR737360, TC379357, TC377225, TC386279, TC381943, BQ607161, TC406807, TC393960, TC382045, DR739471, TC416906, TC402308, TC434820, TC443387, TC379711, TC379903, TC392272, TC417388, TC386519, TC386313, CV759879, TC408907, TC402440, TC391438, TC394307, TC375146, TC386963, TC389589, TC452762, TC386344, TC392778, TC405030, TC397312, TC381963, TC388403, TC383176, TC386422, TC388688, TC385780, TC388822, TC377496, TC388976, TC406708, TC429771, TC409459, TC423576, TC386639, TC402186, TC416658, TC393561, TC393692, TC391671, TC393554, TC407614, TC402603, TC459656, TC441241, TC379436, CA729339, TC460615, CK214224, TC425690, TC433589, TC377410, TC381218, TC389816, CA613620, CK211469, TC400362, TC397729, TC396895, TC434831, TC426326, TC418850, TC382342, TC381462, TC393106, TC401758, TC373914, TC432504, TC392263, TC397676, TC396460, TC389661, TC428944 |
| [Negative regulation of macromolecule biosynthetic process](http://amigo.geneontology.org/cgi-bin/amigo/go.cgi?action=query&view=query&query=GO:0010558&search_constraint=terms) | TC392303, TC410352, TC386040, TC390393, TC397258, TC435533, TC374240, CA710880, TC398862, TC433162, TC387579, TC423252, TC397660, TC393814, TC389363, TC388914, TC458562, TC394716, TC398633, TC446235, TC380063, TC393830, TC380943, TC418414, TC380433, TC378054, TC420735, TC404371, TC395453, TC386961, TC384219, TC390569, TC416685, TC419057, TC403872, TC389090, TC389375, TC404413, TC382655, CK200433, TC398714, TC370114, BQ294582, TC406038, CJ550278, TC395351, TC401915, CK210754, CA632212, DR739994, TC384454, TC411287, TC394916, TC390994, TC378274, TC391143, CK217367, TC379853, TC386414, TC400755, TC440636, TC395723, TC405440, TC389718, TC401210, DR737360, TC379357, TC377225, TC386279, TC381943, BQ607161, TC406807, TC393960, TC382045, DR739471, TC416906, TC402308, TC434820, TC443387, TC379711, TC379903, TC392272, TC417388, TC386519, TC386313, CV759879, TC408907, TC402440, TC391438, TC394307, TC375146, TC386963, TC389589, TC452762, TC386344, TC392778, TC405030, TC397312, TC381963, TC388403, TC383176, TC386422, TC388688, TC385780, TC388822, TC377496, TC388976, TC406708, TC429771, TC409459, TC423576, TC386639, TC402186, TC416658, TC393561, TC393692, TC391671, TC393554, TC407614, TC402603, TC459656, TC441241, TC379436, CA729339, TC460615, CK214224, TC425690, TC433589, TC377410, TC381218, TC389816, CA613620, CK211469, TC400362, TC397729, TC396895, TC434831, TC426326, TC418850, TC382342, TC381462, TC393106, TC401758, TC373914, TC432504, TC392263, TC397676, TC396460, TC389661, TC428944 |
| [Negative regulation of cellular macromolecule biosynthetic process](http://amigo.geneontology.org/cgi-bin/amigo/go.cgi?action=query&view=query&query=GO:2000113&search_constraint=terms) | TC392303, TC410352, TC386040, TC390393, TC397258, TC435533, TC374240, CA710880, TC398862, TC433162, TC387579, TC423252, TC397660, TC393814, TC389363, TC388914, TC458562, TC394716, TC398633, TC446235, TC380063, TC393830, TC380943, TC418414, TC380433, TC378054, TC420735, TC404371, TC395453, TC386961, TC384219, TC390569, TC416685, TC419057, TC403872, TC389090, TC389375, TC404413, TC382655, CK200433, TC398714, TC370114, BQ294582, TC406038, CJ550278, TC395351, TC401915, CK210754, CA632212, DR739994, TC384454, TC411287, TC394916, TC390994, TC378274, TC391143, CK217367, TC379853, TC386414, TC400755, TC440636, TC395723, TC405440, TC389718, TC401210, DR737360, TC379357, TC377225, TC386279, TC381943, BQ607161, TC406807, TC393960, TC382045, DR739471, TC416906, TC402308, TC434820, TC443387, TC379711, TC379903, TC392272, TC417388, TC386519, TC386313, CV759879, TC408907, TC402440, TC391438, TC394307, TC375146, TC386963, TC389589, TC452762, TC386344, TC392778, TC405030, TC397312, TC381963, TC388403, TC383176, TC386422, TC388688, TC385780, TC388822, TC377496, TC388976, TC406708, TC429771, TC409459, TC423576, TC386639, TC402186, TC416658, TC393561, TC393692, TC391671, TC393554, TC407614, TC402603, TC459656, TC441241, TC379436, CA729339, TC460615, CK214224, TC425690, TC433589, TC377410, TC381218, TC389816, CA613620, CK211469, TC400362, TC397729, TC396895, TC434831, TC426326, TC418850, TC382342, TC381462, TC393106, TC401758, TC373914, TC432504, TC392263, TC397676, TC396460, TC389661, TC428944 |
| [Negative regulation of biological process](http://amigo.geneontology.org/cgi-bin/amigo/go.cgi?action=query&view=query&query=GO:0048519&search_constraint=terms) | TC392303, TC410352, TC386040, TC390393, TC394796, TC397258, CV775873, TC435533, TC374240, TC398052, CA710880, TC398862, TC433162, TC387579, TC394459, TC371242, TC384738, TC423252, TC397660, TC393814, TC389363, TC388914, TC388410, TC458562, TC394716, TC398633, TC446235, TC380063, TC393830, TC380943, TC418414, TC380433, TC378054, TC420735, TC404371, TC395453, TC386961, TC384219, TC392329, TC384735, TC390569, TC416685, TC387344, TC419057, TC403872, TC389090, TC389375, TC404413, TC382655, CK200433, TC398714, TC370114, BQ294582, TC406038, CJ550278, TC395351, TC401915, TC398304, TC393100, TC433957, CK210754, CA632212, DR739994, TC384454, TC411287, TC386237, TC394916, TC393904, TC390994, TC378274, TC391143, CK217367, TC379853, TC395303, TC386414, TC400755, TC405511, TC395723, TC440636, TC405440, TC377190, TC389718, TC401210, TC390135, DR737360, TC379357, TC377225, TC386279, TC381943, TC380662, BQ607161, TC406807, TC393960, TC382045, DR739471, TC416906, TC402308, TC434820, TC443387, TC441343, TC379711, TC379903, TC392272, TC417388, TC386519, TC386313, CV759879, TC408907, TC402440, TC420579, TC391438, TC394307, CK211707, TC375146, TC386963, TC389589, TC452762, TC386344, TC392778, TC396751, TC397312, TC405030, TC381963, CK211589, TC388403, TC383176, CA615187, TC386422, TC388688, TC405041, TC385780, TC388547, TC405784, TC399919, TC407572, TC372175, TC388822, TC396365, TC377496, TC388976, TC406708, TC419747, TC429771, TC409459, TC423576, TC386639, TC402186, TC416658, TC393561, TC393692, TC391671, TC393554, TC407614, TC459656, TC382928, TC402603, TC423354, TC441241, TC438243, TC413571, TC379436, CA729339, TC460615, CK214224, TC403929, TC380882, TC423880, TC425690, TC433589, TC377410, TC381218, TC389816, TC422425, CA613620, TC400056, CK211469, TC400362, TC381817, TC403588, TC388520, TC397729, TC411191, TC434831, TC396895, TC426326, TC381462, TC418850, TC382342, TC393106, TC401758, TC373914, TC432504, TC397676, TC396460, TC392263, TC428944, TC389661 |
| [Negative regulation of cellular biosynthetic process](http://amigo.geneontology.org/cgi-bin/amigo/go.cgi?action=query&view=query&query=GO:0031327&search_constraint=terms) | TC392303, TC410352, TC386040, TC390393, TC397258, TC435533, TC374240, CA710880, TC398862, TC433162, TC387579, TC423252, TC397660, TC393814, TC389363, TC388914, TC458562, TC394716, TC398633, TC446235, TC380063, TC393830, TC380943, TC418414, TC380433, TC378054, TC420735, TC404371, TC395453, TC386961, TC384219, TC390569, TC416685, TC419057, TC403872, TC389090, TC389375, TC404413, TC382655, CK200433, TC398714, TC370114, BQ294582, TC406038, CJ550278, TC395351, TC401915, CK210754, CA632212, DR739994, TC384454, TC411287, TC394916, TC390994, TC378274, TC391143, CK217367, TC379853, TC386414, TC400755, TC440636, TC395723, TC405440, TC389718, TC401210, DR737360, TC379357, TC377225, TC386279, TC381943, BQ607161, TC406807, TC393960, TC382045, DR739471, TC416906, TC402308, TC434820, TC443387, TC379711, TC379903, TC392272, TC417388, TC386519, TC386313, CV759879, TC408907, TC402440, TC391438, TC394307, TC375146, TC386963, TC389589, TC452762, TC386344, TC392778, TC405030, TC397312, TC381963, TC388403, TC383176, TC386422, TC388688, TC385780, TC388822, TC377496, TC388976, TC406708, TC429771, TC409459, TC423576, TC386639, TC402186, TC416658, TC393561, TC393692, TC391671, TC393554, TC407614, TC402603, TC459656, TC441241, TC379436, CA729339, TC460615, CK214224, TC425690, TC433589, TC377410, TC381218, TC389816, CA613620, CK211469, TC400362, TC397729, TC396895, TC434831, TC426326, TC418850, TC382342, TC381462, TC393106, TC401758, TC373914, TC432504, TC392263, TC397676, TC396460, TC389661, TC428944 |
| [Negative regulation of biosynthetic process](http://amigo.geneontology.org/cgi-bin/amigo/go.cgi?action=query&view=query&query=GO:0009890&search_constraint=terms) | TC392303, TC410352, TC386040, TC390393, TC397258, TC435533, TC374240, CA710880, TC398862, TC433162, TC387579, TC423252, TC397660, TC393814, TC389363, TC388914, TC458562, TC394716, TC398633, TC446235, TC380063, TC393830, TC380943, TC418414, TC380433, TC378054, TC420735, TC404371, TC395453, TC386961, TC384219, TC390569, TC416685, TC419057, TC403872, TC389090, TC389375, TC404413, TC382655, CK200433, TC398714, TC370114, BQ294582, TC406038, CJ550278, TC395351, TC401915, CK210754, CA632212, DR739994, TC384454, TC411287, TC394916, TC390994, TC378274, TC391143, CK217367, TC379853, TC386414, TC400755, TC440636, TC395723, TC405440, TC389718, TC401210, DR737360, TC379357, TC377225, TC386279, TC381943, BQ607161, TC406807, TC393960, TC382045, DR739471, TC416906, TC402308, TC434820, TC443387, TC379711, TC379903, TC392272, TC417388, TC386519, TC386313, CV759879, TC408907, TC402440, TC391438, TC394307, TC375146, TC386963, TC389589, TC452762, TC386344, TC392778, TC405030, TC397312, TC381963, TC388403, TC383176, TC386422, TC388688, TC385780, TC388822, TC377496, TC388976, TC406708, TC429771, TC409459, TC423576, TC386639, TC402186, TC416658, TC393561, TC393692, TC391671, TC393554, TC407614, TC402603, TC459656, TC441241, TC379436, CA729339, TC460615, CK214224, TC425690, TC433589, TC377410, TC381218, TC389816, CA613620, CK211469, TC400362, TC397729, TC396895, TC434831, TC426326, TC418850, TC382342, TC381462, TC393106, TC401758, TC373914, TC432504, TC392263, TC397676, TC396460, TC389661, TC428944 |
| [Response to biotic stimulus](http://amigo.geneontology.org/cgi-bin/amigo/go.cgi?action=query&view=query&query=GO:0009607&search_constraint=terms) | TC392303, TC404158, TC386040, TC390393, TC416529, TC397258, CV775873, TC396230, TC435533, TC416069, BQ838511, TC374240, TC398052, TC425841, TC424204, TC433162, TC387579, TC394459, TC416154, TC384738, TC404978, TC393814, TC388410, TC416493, TC458562, TC394716, TC398633, TC380063, TC398538, TC393830, TC380943, TC410063, TC378054, TC403885, TC404371, TC386961, TC384219, TC410194, TC387344, TC407076, TC419057, TC412483, TC398714, TC370114, TC418032, TC397444, TC398304, TC393100, TC433957, CK210754, TC417012, TC393523, CA632212, TC411287, TC386237, TC394916, TC393904, TC390994, TC406236, TC378274, TC391130, TC391143, TC446038, TC434396, TC395303, TC386414, CK214702, TC400755, TC403986, TC405511, TC440636, CK213497, TC458987, TC385365, TC389718, TC401210, TC386707, TC390135, TC379357, TC377225, TC386279, TC411684, CK201148, TC381943, TC412520, TC380662, TC413392, TC402308, TC402534, TC377308, TC415083, TC379903, TC423265, TC377061, TC401244, TC406584, TC386519, TC386313, CV759879, TC408907, CA606693, TC391438, TC417992, CK211707, TC386963, TC398592, TC412732, TC452762, TC405695, TC386344, TC396751, TC405030, TC381963, TC388403, TC383176, CA615187, TC416695, TC405041, TC384071, TC452503, TC385780, TC388547, TC406469, TC405784, TC399919, TC407572, TC372175, TC369199, TC410690, TC396365, TC388976, TC419747, TC409459, TC386639, TC402186, TC405356, TC393692, TC373787, TC397415, TC382928, TC402603, TC423354, TC441241, TC449724, TC379436, TC413043, TC417363, CK214224, TC408299, TC403929, TC376263, TC380882, TC423880, TC379635, TC429374, TC384010, TC416169, TC425690, TC377410, TC381218, TC422425, TC392323, TC413066, TC383677, TC393820, TC461622, CK211469, CB307332, TC404842, TC403588, TC388520, TC376933, TC397729, TC411191, DR740372, TC403803, TC399342, TC381462, TC382342, TC401758, TC373914, TC432504, TC392263, TC397676, TC389661 |
| [Sensory perception of pain](http://amigo.geneontology.org/cgi-bin/amigo/go.cgi?action=query&view=query&query=GO:0019233&search_constraint=terms) | TC410352, DR739471, TC434820, TC443387, TC379711, TC392272, CV775873, TC386313, TC398052, TC425841, TC402440, TC387579, TC394459, TC384738, TC423252, TC394307, TC397660, CK211707, TC375146, TC386963, TC389363, TC388914, TC388410, TC389589, TC446235, TC392778, TC397312, TC420735, TC395453, TC386422, TC386961, TC388688, TC390569, TC416685, TC403872, TC399919, TC407572, TC389090, TC372175, TC389375, TC388822, TC396365, TC406708, TC377496, TC419747, TC382655, TC429771, CK200433, TC423576, TC406038, TC416658, TC395351, TC386646, TC401915, TC393561, TC393100, TC391671, TC393554, TC407614, TC382928, TC459656, DR739994, TC384454, CA729339, TC386237, TC417363, TC393904, CK217367, TC379853, TC377410, TC381218, TC389816, TC395303, TC400755, TC405440, TC458987, TC401210, DR737360, TC390135, TC397729, TC377225, TC434831, TC396895, TC426326, TC381462, TC393106, TC401758, BQ607161, TC406807, TC392263, TC396460, TC428944, TC389661, TC382045 |
| [Negative regulation of macromolecule metabolic process](http://amigo.geneontology.org/cgi-bin/amigo/go.cgi?action=query&view=query&query=GO:0010605&search_constraint=terms) | TC392303, TC410352, TC386040, TC390393, TC397258, TC435533, TC374240, CA710880, TC398862, TC433162, TC387579, TC371242, TC423252, TC397660, TC393814, TC389363, TC388914, TC458562, TC394716, TC398633, TC446235, TC380063, TC393830, TC380943, TC418414, TC380433, TC378054, TC420735, TC404371, TC395453, TC386961, TC384219, TC390569, TC416685, TC419057, TC403872, TC389090, TC389375, TC404413, TC382655, CK200433, TC398714, TC370114, BQ294582, TC406038, CJ550278, TC395351, TC401915, CK210754, CA632212, DR739994, TC384454, TC411287, TC394916, TC390994, TC378274, TC391143, CK217367, TC379853, TC386414, TC400755, TC440636, TC395723, TC405440, TC377190, TC389718, TC401210, DR737360, TC379357, TC377225, TC386279, TC381943, BQ607161, TC406807, TC393960, TC382045, DR739471, TC416906, TC402308, TC434820, TC443387, TC379711, TC379903, TC392272, TC417388, TC386519, TC386313, CV759879, TC408907, TC402440, TC391438, TC394307, TC375146, TC386963, TC389589, TC452762, TC386344, TC392778, TC405030, TC397312, TC381963, TC388403, TC383176, TC386422, TC388688, TC385780, TC388822, TC377496, TC388976, TC406708, TC429771, TC409459, TC423576, TC386639, TC402186, TC416658, TC393561, TC393692, TC391671, TC393554, TC407614, TC459656, TC402603, TC441241, TC379436, CA729339, TC460615, CK214224, TC425690, TC433589, TC377410, TC381218, TC389816, CA613620, CK211469, TC400362, TC397729, TC396895, TC434831, TC426326, TC418850, TC382342, TC381462, TC393106, TC401758, TC373914, TC432504, TC392263, TC397676, TC396460, TC389661, TC428944 |
| [Ngative regulation of cellular metabolic process](http://amigo.geneontology.org/cgi-bin/amigo/go.cgi?action=query&view=query&query=GO:0031324&search_constraint=terms) | TC392303, TC410352, TC386040, TC390393, TC397258, TC435533, TC374240, CA710880, TC398862, TC433162, TC387579, TC371242, TC423252, TC397660, TC393814, TC389363, TC388914, TC458562, TC394716, TC398633, TC446235, TC380063, TC393830, TC380943, TC418414, TC380433, TC378054, TC420735, TC404371, TC395453, TC386961, TC384219, TC390569, TC416685, TC419057, TC403872, TC389090, TC389375, TC404413, TC382655, CK200433, TC398714, TC370114, BQ294582, TC406038, CJ550278, TC395351, TC401915, CK210754, CA632212, DR739994, TC384454, TC411287, TC394916, TC390994, TC378274, TC391143, CK217367, TC379853, TC386414, TC400755, TC440636, TC395723, TC405440, TC389718, TC401210, DR737360, TC379357, TC377225, TC386279, TC381943, BQ607161, TC406807, TC393960, TC382045, DR739471, TC416906, TC402308, TC434820, TC443387, TC379711, TC379903, TC392272, TC417388, TC386519, TC386313, CV759879, TC408907, TC402440, TC391438, TC394307, TC375146, TC386963, TC389589, TC452762, TC386344, TC392778, TC405030, TC397312, TC381963, TC388403, TC383176, TC386422, TC388688, TC385780, TC388822, TC377496, TC388976, TC406708, TC429771, TC409459, TC423576, TC386639, TC402186, TC416658, TC393561, TC393692, TC391671, TC393554, TC407614, TC459656, TC402603, TC441241, TC379436, CA729339, TC460615, CK214224, TC425690, TC433589, TC377410, TC381218, TC389816, CA613620, CK211469, TC400362, TC397729, TC396895, TC434831, TC426326, TC418850, TC382342, TC381462, TC393106, TC401758, TC373914, TC432504, TC392263, TC397676, TC396460, TC389661, TC428944 |
| [Negative regulation of metabolic process](http://amigo.geneontology.org/cgi-bin/amigo/go.cgi?action=query&view=query&query=GO:0009892&search_constraint=terms) | TC392303, TC410352, TC386040, TC390393, TC397258, TC435533, TC374240, CA710880, TC398862, TC433162, TC387579, TC371242, TC423252, TC397660, TC393814, TC389363, TC388914, TC458562, TC394716, TC398633, TC446235, TC380063, TC393830, TC380943, TC418414, TC380433, TC378054, TC420735, TC404371, TC395453, TC386961, TC384219, TC390569, TC416685, TC419057, TC403872, TC389090, TC389375, TC404413, TC382655, CK200433, TC398714, TC370114, BQ294582, TC406038, CJ550278, TC395351, TC401915, CK210754, CA632212, DR739994, TC384454, TC411287, TC394916, TC390994, TC378274, TC391143, CK217367, TC379853, TC386414, TC400755, TC440636, TC395723, TC405440, TC377190, TC389718, TC401210, DR737360, TC379357, TC377225, TC386279, TC381943, BQ607161, TC406807, TC393960, TC382045, DR739471, TC416906, TC402308, TC434820, TC443387, TC379711, TC379903, TC392272, TC417388, TC386519, TC386313, CV759879, TC408907, TC402440, TC391438, TC394307, TC375146, TC386963, TC389589, TC452762, TC386344, TC392778, TC405030, TC397312, TC381963, TC388403, TC383176, TC386422, TC388688, TC385780, TC388822, TC377496, TC388976, TC406708, TC429771, TC409459, TC423576, TC386639, TC402186, TC416658, TC393561, TC393692, TC391671, TC393554, TC407614, TC459656, TC402603, TC441241, TC379436, CA729339, TC460615, CK214224, TC425690, TC433589, TC377410, TC381218, TC389816, CA613620, CK211469, TC400362, TC397729, TC396895, TC434831, TC426326, TC418850, TC382342, TC381462, TC393106, TC401758, TC373914, TC432504, TC392263, TC397676, TC396460, TC389661, TC428944 |
| [Multi-organism process](http://amigo.geneontology.org/cgi-bin/amigo/go.cgi?action=query&view=query&query=GO:0051704&search_constraint=terms) | TC392303, TC404158, TC386040, TC390393, TC416529, TC397258, CV775873, TC396230, TC435533, TC416069, BQ838511, TC374240, TC398052, TC425841, TC424204, TC433162, TC387579, TC394459, TC371242, TC416154, TC384738, TC404978, TC393814, TC379171, TC388410, TC416493, TC458562, TC394716, TC398633, TC380063, TC398538, TC393830, TC380943, TC410063, TC378054, TC403885, TC404371, TC386961, TC384219, TC410194, TC387344, TC407076, TC419057, TC412483, TC398714, TC370114, TC393970, TC418032, TC397444, TC398304, TC393100, TC433957, CK210754, TC417012, TC393523, CA632212, TC411287, TC386237, TC394916, TC393904, TC390994, TC406236, TC378274, TC391130, TC391143, TC446038, TC434396, TC395303, TC386414, CK214702, TC400755, TC403986, TC405511, TC440636, CK213497, TC458987, TC385365, TC389718, TC401210, TC386707, TC390135, TC379357, TC377225, TC386279, TC411684, CK201148, TC381943, TC412520, TC380662, TC413392, TC402308, TC402534, TC377308, TC415083, TC379903, TC423265, TC377061, TC401244, TC406584, TC386519, TC386313, CV759879, TC408907, CA606693, TC391438, TC417992, CK211707, TC386963, TC398592, TC412732, TC452762, TC405695, TC386344, TC396751, TC370350, TC405030, TC381963, TC388403, TC383176, CA615187, TC416695, TC405041, TC384071, TC452503, TC385780, TC388547, TC406469, TC405784, TC399919, TC407572, TC372175, TC369199, TC410690, TC396365, TC388976, TC419747, TC409459, TC386639, TC402186, TC405356, TC393692, TC373787, TC397415, TC382928, TC402603, TC423354, TC441241, TC449724, TC379436, TC413043, TC417363, CK214224, TC408299, TC403929, TC376263, TC380882, TC423880, TC379635, TC429374, TC384010, TC416169, TC425690, TC377410, TC381218, TC422425, TC392323, TC413066, TC383677, TC393820, TC461622, CK211469, CB307332, TC404842, TC403588, TC388520, TC376933, TC397729, TC411191, DR740372, TC403803, TC399342, TC381462, TC382342, TC401758, TC373914, TC432504, TC392263, TC397676, TC389661 |
| [RNA catabolic process](http://amigo.geneontology.org/cgi-bin/amigo/go.cgi?action=query&view=query&query=GO:0006401&search_constraint=terms) | TC410352, DR739471, TC386040, TC434820, TC402308, TC416906, TC443387, TC379711, TC390393, TC379903, TC397258, TC392272, TC417388, TC435533, TC386519, TC374240, CV759879, CA710880, TC408907, TC402440, TC398862, TC433162, TC391438, TC423252, TC394307, TC397660, TC375146, TC393814, TC389363, TC388914, TC389589, TC394716, TC398633, TC446235, TC452762, TC380063, TC393830, TC380943, TC418414, TC386344, TC392778, TC378054, TC405030, TC381963, TC388403, TC420735, TC383176, TC404371, TC395453, TC386422, TC384219, TC388688, TC390569, TC385780, TC416685, TC403872, TC389090, TC389375, TC388822, TC406708, TC388976, TC377496, TC382655, TC429771, CK200433, TC423576, TC373791, TC406038, TC386639, TC402186, TC416658, TC395351, TC401915, TC393561, TC393692, TC391671, TC393554, TC407614, TC402603, DR739994, TC449724, TC384454, TC379436, TC411287, CA729339, CK214224, TC391143, TC387710, CK217367, TC433589, TC379853, TC386414, TC389816, TC440636, TC395723, TC405440, CA613620, TC393820, CK211469, TC389718, TC379357, DR737360, TC386279, TC434831, TC396895, TC381943, TC426326, TC382342, TC393106, BQ607161, TC373914, TC432504, TC396460, TC397676, TC428944, TC404606 |
| [mRNA catabolic process](http://amigo.geneontology.org/cgi-bin/amigo/go.cgi?action=query&view=query&query=GO:0006402&search_constraint=terms) | TC410352, DR739471, TC386040, TC434820, TC402308, TC416906, TC443387, TC379711, TC390393, TC379903, TC397258, TC392272, TC417388, TC435533, TC386519, TC374240, CV759879, CA710880, TC408907, TC402440, TC398862, TC433162, TC391438, TC423252, TC394307, TC397660, TC375146, TC393814, TC389363, TC388914, TC389589, TC394716, TC398633, TC446235, TC452762, TC380063, TC393830, TC380943, TC418414, TC386344, TC392778, TC378054, TC405030, TC381963, TC388403, TC420735, TC383176, TC404371, TC395453, TC386422, TC384219, TC388688, TC390569, TC385780, TC416685, TC403872, TC389090, TC389375, TC388822, TC406708, TC388976, TC377496, TC382655, TC429771, CK200433, TC423576, TC373791, TC406038, TC386639, TC402186, TC416658, TC395351, TC401915, TC393561, TC393692, TC391671, TC393554, TC407614, TC402603, DR739994, TC384454, TC379436, TC411287, CA729339, CK214224, TC391143, TC387710, CK217367, TC433589, TC379853, TC386414, TC389816, TC440636, TC395723, TC405440, CA613620, CK211469, TC389718, TC379357, DR737360, TC386279, TC434831, TC396895, TC381943, TC426326, TC382342, TC393106, BQ607161, TC373914, TC432504, TC396460, TC397676, TC428944, TC404606 |
| [Cellular response to stress](http://amigo.geneontology.org/cgi-bin/amigo/go.cgi?action=query&view=query&query=GO:0033554&search_constraint=terms) | TC392303, TC410352, TC386040, TC407978, TC390393, TC397258, TC375864, TC435533, TC374240, CA710880, TC433162, TC387579, TC371242, TC423252, TC397660, TC393814, TC389363, TC388914, TC458562, TC394716, TC398633, TC446235, TC380063, TC433844, TC393830, TC380943, TC378054, TC420735, TC404371, TC395453, TC386961, TC384219, TC390569, TC416685, TC407076, TC419057, TC403872, TC389090, TC389375, TC382655, CK200433, TC398714, BQ294582, TC406038, TC395351, TC401915, TC456619, CK210754, CA632212, DR739994, TC384454, TC411287, TC394916, TC390994, TC378274, TC391143, CK217367, TC434396, TC379853, TC370315, TC386414, TC400755, TC405511, TC440636, TC395723, TC405440, TC376420, TC389718, TC401210, TC386707, DR737360, TC379357, TC377225, TC386279, TC381943, BQ607161, TC393960, TC382045, TC389162, DR739471, TC402308, TC434820, TC443387, TC379711, TC379903, TC392272, TC377061, TC386519, TC386313, CV759879, TC408907, TC402440, TC391438, TC394307, TC375146, TC386963, TC389589, TC452762, TC386344, TC392778, TC405030, TC397312, TC381963, TC388403, TC383176, TC386422, TC374164, TC388688, TC385780, TC388822, TC377496, TC388976, TC406708, TC429771, TC423576, TC386639, TC402186, TC416658, TC393561, TC393692, TC391671, TC393554, TC407614, TC402603, TC423354, TC441241, TC438243, TC413571, TC379436, CA729339, CK214224, TC380882, TC423880, TC433589, TC377410, TC381218, TC389816, CA613620, TC461622, CK211469, TC403588, TC397729, TC396895, TC434831, TC426326, TC382342, TC381462, TC393106, TC401758, TC373914, TC432504, TC392263, TC397676, TC396460, TC389661, TC428944 |
| [Defense response to bacterium](http://amigo.geneontology.org/cgi-bin/amigo/go.cgi?action=query&view=query&query=GO:0042742&search_constraint=terms) | TC392303, TC402534, TC415083, TC416529, TC423265, CV775873, TC396230, TC401244, TC406584, TC416069, BQ838511, TC386313, TC398052, TC425841, TC424204, TC387579, CA606693, TC417992, TC394459, TC416154, TC384738, CK211707, TC386963, TC388410, TC416493, TC398592, TC458562, TC412732, TC398538, TC405695, TC396751, TC410063, TC403885, CA615187, TC416695, TC386961, TC410194, TC406469, TC388547, TC387344, TC405784, TC399919, TC407572, TC372175, TC412483, TC410690, TC396365, TC419747, TC398714, TC405356, TC397444, TC418032, TC398304, TC433957, TC393100, CK210754, TC417012, TC382928, TC441241, TC413043, TC417363, TC386237, TC394916, TC393904, TC390994, TC408299, TC406236, TC391130, TC378274, TC403929, TC376263, TC379635, TC446038, TC416169, TC425690, TC377410, TC381218, TC395303, CK214702, TC422425, TC400755, TC392323, TC403986, TC413066, TC383677, CK213497, TC458987, CB307332, TC404842, TC385365, TC388520, TC401210, TC390135, TC397729, TC377225, TC411684, TC411191, TC403803, TC399342, TC381462, TC412520, TC380662, TC413392, TC401758, TC392263, TC389661 |
| [Cellular component biogenesis](http://amigo.geneontology.org/cgi-bin/amigo/go.cgi?action=query&view=query&query=GO:0044085&search_constraint=terms) | TC461921, TC410352, TC386040, TC379942, TC407978, TC390393, TC416529, TC397258, TC389190, CV775873, TC396230, TC460760, TC427210, TC416069, BQ838511, TC398052, CA710880, TC400388, TC421871, TC398862, TC433162, TC394459, TC416154, TC384738, TC423252, TC397660, TC389363, TC388410, CK203550, TC416493, TC458562, TC421914, TC406264, TC387410, TC380063, TC398538, TC418414, TC410063, TC378054, TC448471, TC395453, TC410194, TC392875, TC435546, TC390569, TC405540, TC407076, TC419057, TC403872, TC425847, TC391948, CJ727624, TC398714, BQ294582, CA614761, CJ550278, TC381619, TC457126, TC403580, TC413427, TC411128, TC401915, TC397444, TC398304, TC425878, TC393100, TC433957, TC417012, CA632212, DR739994, TC459193, TC394916, TC394206, TC394820, TC406236, TC418073, TC378274, TC391130, TC417308, CK217367, TC409599, TC445767, TC446038, TC423804, TC413460, TC379853, BQ609416, CK214702, TC449504, TC395723, TC405440, CK213497, CJ792862, TC389718, TC404052, TC377225, TC381943, TC412520, TC432001, TC413392, BQ607161, TC382045, TC404606, TC416906, TC402308, TC434820, TC379711, TC400330, TC400260, CA595837, TC379903, TC423265, TC392272, TC377061, TC401244, CA730421, TC417388, TC446465, TC386313, CV759879, CA606693, TC391438, CK211707, TC386963, TC412732, TC405695, CA700201, TC392778, TC396751, TC397312, TC405030, TC388403, TC376527, CA615187, TC416695, TC388718, TC419584, TC383701, TC385780, TC406469, TC405784, TC399919, TC407572, TC372175, TC410690, TC406708, TC419747, TC410074, TC402186, TC405356, CV763657, TC402603, TC441241, TC413043, CA729339, TC408299, BQ239045, CA709177, TC376263, TC416169, TC381218, TC399352, TC408229, TC422425, TC408312, CA613620, TC398805, CV781430, TC388520, TC411191, TC400108, TC399342, TC434831, TC426326, TC381462, CD882425, TC373914, TC392263, TC392303, TC445166, CA611770, TC435533, TC374240, CA720842, TC425841, CF554444, TC424204, TC387579, TC406870, TC388914, TC393814, TC387621, TC398633, TC394716, TC446235, TC402545, TC378271, TC380943, TC393830, TC380433, TC403885, TC420735, TC435281, TC384219, TC386961, TC416685, TC372664, TC387344, TC412483, TC389090, TC389375, TC382655, CK200433, TC406038, TC393970, TC395351, TC418032, CK210754, TC410147, TC376490, TC384454, TC411287, TC386237, TC393904, TC390994, TC391143, TC381463, TC403157, TC410066, TC386414, TC395303, TC400755, TC440636, TC403986, TC405615, TC376420, TC458987, TC404843, TC431879, TC391613, CD876572, TC385365, TC382737, TC401210, TC369736, DR737360, TC390135, TC387135, TC397885, TC411684, TC411480, TC380662, TC393960, TC395298, DR739471, TC402534, TC443387, TC415083, TC386535, GH729256, TC406584, TC386519, CA605200, TC402440, TC417992, TC391411, TC417341, TC394307, TC388665, TC375146, TC389589, TC381988, TC452762, TC405475, TC386344, TC381963, TC383176, TC370044, CD878039, TC386422, TC388688, TC403328, TC418845, TC388547, TC414606, TC388822, TC377496, TC388976, TC396365, TC429771, TC410078, TC423576, TC386639, TC416658, TC393561, TC393692, TC391671, TC393554, TC407614, TC382928, TC379436, TC417363, TC403573, TC403929, TC413700, TC394118, TC449043, TC379635, TC440321, TC373259, TC433589, TC377410, TC389816, TC372530, TC392323, TC413066, TC391641, CK211469, CB307332, TC404842, TC397729, TC403803, TC396895, TC382342, TC393106, TC401758, TC432504, TC397676, TC396460, TC417067, TC389661, TC428944 |
| [DNA metabolic process](http://amigo.geneontology.org/cgi-bin/amigo/go.cgi?action=query&view=query&query=GO:0006259&search_constraint=terms) | TC392303, TC410352, TC386040, TC390393, TC397258, TC375864, TC435533, TC374240, CA710880, TC433162, TC387579, TC371242, TC423252, TC397660, TC393814, TC389363, TC388914, TC458562, TC394716, TC398633, TC446235, TC380063, TC393830, TC380943, TC378054, TC420735, TC404371, TC395453, TC386961, TC384219, TC384735, TC390569, TC416685, TC419057, TC403872, TC389090, TC389375, TC382655, CK200433, TC398714, TC373994, BQ294582, TC406038, TC395351, TC403580, TC401915, GH732878, CK210754, CA632212, DR739994, TC384454, TC411287, TC394916, TC390994, TC378274, TC391143, CK217367, TC379853, TC370315, TC386414, TC400755, TC405511, TC440636, TC395723, TC405440, TC377190, TC389718, TC401210, DR737360, TC379357, TC377225, TC386279, TC381943, BQ607161, TC393960, TC389162, DR739471, TC402308, TC434820, TC441343, TC443387, TC379711, TC379903, TC392272, TC377061, TC386519, TC386313, CV759879, TC408907, TC402440, TC391438, TC394307, TC409208, TC375146, TC386963, TC454407, TC389589, TC452762, TC386344, TC392778, TC405030, TC397312, TC381963, TC388403, TC383176, TC371455, TC386422, TC374164, TC388688, TC385780, TC388822, TC377496, TC388976, TC406708, TC429771, TC423576, TC386639, TC402186, TC416658, TC393561, TC393692, TC391671, TC393554, TC407614, TC458205, TC402603, TC423354, TC441241, TC413571, TC379436, CA729339, CK214224, TC380882, TC423880, TC433589, TC377410, TC381218, TC418091, TC389816, CA613620, CK211469, TC369092, TC403588, TC397729, TC396895, TC434831, TC426326, TC382342, TC381462, TC393106, TC401758, TC373914, TC432504, TC392263, TC397676, TC396460, TC389661, TC428944 |
| [Organelle organization](http://amigo.geneontology.org/cgi-bin/amigo/go.cgi?action=query&view=query&query=GO:0006996&search_constraint=terms) | TC461921, TC410352, TC386040, TC390393, TC416529, TC397258, TC389190, CV775873, TC396230, TC460760, TC427210, TC416069, BQ838511, TC398052, CA710880, TC400388, TC433162, TC394459, TC371242, TC416154, TC384738, TC423252, TC397660, TC389363, TC379171, TC388410, CK203550, TC416493, TC458562, TC406264, TC387410, TC380063, TC398538, TC410063, TC378054, TC448471, TC395453, TC410194, TC392875, TC409843, TC435546, TC384735, TC390569, TC405540, TC419057, TC403872, TC425847, CJ727624, TC398714, BQ294582, CA614761, CJ550278, TC457126, TC403580, TC413427, TC411128, TC401915, TC397444, TC398304, TC425878, TC393100, TC433957, TC417012, CA632212, DR739994, TC459193, TC394916, TC394206, TC394820, TC406236, TC418073, TC378274, TC391130, TC417308, CK217367, TC409599, TC445767, TC446038, TC413460, TC379853, TC370315, BQ609416, CK214702, TC449504, TC395723, TC405440, CK213497, CJ792862, TC385233, TC389718, TC404052, TC377225, TC381943, TC412520, TC432001, TC413392, BQ607161, TC406807, TC382045, TC402308, TC434820, TC441343, TC379711, TC400330, TC378790, CA595837, TC379903, TC423265, TC392272, TC377061, TC401244, CA730421, TC446465, TC386313, CV759879, TC408907, CA606693, TC391438, CK211707, TC386963, TC412732, TC405695, CA700201, TC392778, TC396751, TC397312, TC405030, CK211589, TC388403, TC376527, CA615187, TC416695, TC388718, TC419584, TC383701, TC385780, TC406469, TC405784, TC399919, TC407572, TC372175, TC410690, TC406708, TC419747, TC410074, TC402186, TC405356, CV763657, TC423354, TC402603, TC441241, TC413571, TC413043, CA729339, CK214224, TC408299, BQ239045, CA709177, TC376263, TC416169, TC425690, TC381218, TC408229, TC422425, TC408312, CA613620, TC398805, TC400362, CV781430, TC388520, TC411191, TC399342, TC434831, TC426326, TC381462, CD882425, TC373914, TC392263, TC392303, TC394796, TC445166, CA611770, TC435533, TC374240, CA720842, TC425841, CF554444, TC424204, TC387579, TC406870, TC388914, TC393814, TC376220, TC387621, TC398633, TC394716, TC446235, TC380943, TC393830, TC380433, TC403885, TC420735, TC404371, TC384219, TC386961, TC416685, TC372664, TC387344, TC412483, TC389090, TC389375, TC404413, TC382655, CK200433, TC406038, TC393970, TC395351, TC418032, CK210754, TC410147, TC384454, TC411287, TC386237, TC393904, TC390994, TC391143, TC381463, TC403157, TC386414, TC395303, TC400755, TC440636, TC405511, TC403986, TC405615, TC458987, TC377190, TC404843, TC431879, CD876572, TC385365, TC401210, DR737360, TC379357, TC390135, TC397885, TC386279, TC411684, TC411480, TC409650, TC380662, TC393960, TC389162, DR739471, TC402534, TC443387, TC415083, TC386535, TC390630, GH729256, TC406584, TC386519, CA605200, TC402440, TC417992, TC391411, TC394307, TC409208, TC388665, TC375146, TC389589, TC381988, TC452762, TC386344, TC373637, TC381963, TC383176, CD878039, TC374879, TC386422, TC388688, TC418845, TC388547, TC414606, TC388822, TC377496, TC388976, TC396365, TC429771, TC410078, TC423576, TC386639, TC416658, TC393561, TC393948, TC393692, TC391671, TC393554, TC407614, TC459656, TC382928, TC379436, TC417363, TC403573, TC413700, TC403929, TC449043, TC380882, TC379635, TC440321, TC423880, TC433589, TC377410, TC418091, TC389816, TC372530, TC392323, TC413066, TC391641, TC461622, CK211469, CB307332, TC404842, TC381817, TC403588, TC397729, TC403803, TC396895, TC382342, TC393106, TC401758, TC432504, TC397676, TC396460, TC417067, TC389661, TC428944 |
| [Cellular response to stimulus](http://amigo.geneontology.org/cgi-bin/amigo/go.cgi?action=query&view=query&query=GO:0051716&search_constraint=terms) | TC392303, TC410352, TC386040, TC407978, TC390393, TC397258, TC375864, TC435533, TC374240, CA710880, TC433162, TC387579, TC371242, TC423252, TC397660, TC393814, TC389363, TC388914, TC458562, TC394716, TC398633, TC446235, TC380063, TC433844, TC393830, TC380943, TC378054, TC420735, TC404371, TC395453, TC386961, TC384219, TC392329, TC390569, TC416685, TC407076, TC419057, TC403872, TC389090, TC389375, TC382655, CK200433, TC398714, BQ294582, TC406038, TC395351, TC401915, TC459245, TC456619, CK210754, CA632212, DR739994, TC384454, TC411287, TC380329, TC394916, TC390994, TC378274, TC391143, CK217367, TC434396, TC379853, TC370315, TC386414, TC400755, TC405511, TC440636, TC395723, TC405440, TC403968, TC376420, TC389718, TC401210, TC386707, DR737360, TC379357, TC377225, TC386279, TC381943, BQ607161, TC393960, TC382045, TC389162, TC382830, DR739471, TC402308, TC434820, TC443387, CK212850, TC379711, TC379903, TC378790, TC392272, TC377061, TC386519, TC386313, CV759879, TC408907, TC402440, TC420579, TC391438, TC394307, TC375146, TC386963, TC398592, TC389589, TC452762, TC386344, TC392778, TC405030, TC397312, TC381963, TC388403, TC383176, TC386422, TC374164, TC388688, TC405041, TC385780, TC412150, TC388822, TC377496, TC388976, TC406708, TC429771, TC423576, TC386639, TC402186, TC416658, TC393561, TC393692, TC391671, TC393554, TC407614, TC402603, TC423354, TC441241, TC438243, TC413571, TC379436, CA729339, CK214224, TC411941, TC380882, TC423880, TC425690, TC433589, TC377410, TC381218, TC389816, CA613620, TC461622, CK211469, TC403588, TC397729, TC434831, TC396895, TC426326, TC382342, TC381462, TC393106, TC377749, TC401758, TC373914, TC432504, TC392263, TC397676, TC396460, TC428944, TC389661 |
| [mRNA metabolic process](http://amigo.geneontology.org/cgi-bin/amigo/go.cgi?action=query&view=query&query=GO:0016071&search_constraint=terms) | TC369628, TC410352, DR739471, TC386040, TC434820, TC402308, TC416906, TC443387, TC379711, TC390393, TC388566, TC379903, TC397258, TC392272, TC417388, TC435533, TC386519, TC374240, CV759879, CA710880, TC408907, TC402440, TC398862, TC433162, TC391438, TC423252, TC394307, TC397660, TC375146, TC393814, TC389363, TC388914, TC389589, TC376220, TC394716, TC398633, TC446235, TC387410, TC452762, TC380063, TC393830, TC380943, TC418414, TC386344, TC392778, TC378054, TC405030, TC381963, TC388403, TC420735, TC383176, TC404371, TC395453, TC386422, TC384219, TC388688, TC390569, TC405540, TC385780, TC416685, TC403872, TC389090, TC389375, TC388822, TC406708, TC388976, TC377496, TC382655, TC429771, CK200433, TC423576, TC373791, TC406038, TC386639, TC402186, TC416658, TC395351, TC401915, TC393561, TC393692, TC391671, TC393554, TC407614, TC402603, DR739994, TC384454, TC379436, TC411287, CA729339, CK214224, TC391143, TC387710, CK217367, TC433589, TC379853, TC399352, TC386414, TC389816, TC440636, TC395723, TC405440, CA613620, CK211469, TC385233, TC389718, TC379357, DR737360, TC387135, TC386279, TC434831, TC396895, TC381943, TC426326, TC382342, TC393106, BQ607161, TC373914, TC432504, TC396460, TC397676, TC428944, TC404606 |
| [Response to stress](http://amigo.geneontology.org/cgi-bin/amigo/go.cgi?action=query&view=query&query=GO:0006950&search_constraint=terms) | TC410352, TC386040, TC373613, TC407978, TC390393, TC416529, TC397258, CV775873, TC391946, TC396230, TC375864, TC416069, BQ838511, TC398052, CA710880, TC433162, TC376351, TC394459, TC371242, TC416154, TC384738, TC423252, TC397660, TC389363, TC379171, TC388410, TC416493, TC458562, TC387410, TC380063, TC398538, TC433844, TC388950, CJ944525, TC410063, TC378054, TC373702, TC395453, TC410194, TC392329, TC390569, TC407076, TC419057, TC445939, TC403872, TC398714, BQ294582, CJ550278, TC401915, TC397444, TC398304, TC393100, TC433957, CV771134, TC417012, TC423239, CA632212, DR739994, TC394916, TC406236, TC378274, TC391130, CK217367, TC391128, TC409599, TC446038, TC379853, TC370315, CK214702, TC395723, TC405440, TC403968, CK213497, TC391742, TC389718, TC386707, TC377225, TC392033, CK201148, TC381943, TC412520, TC413392, TC418928, BQ607161, TC406807, TC372845, TC382045, TC369628, TC402308, TC434820, TC379711, TC377308, TC379903, TC423265, TC392272, TC377061, TC401244, DR739350, TC386313, CV759879, TC408907, CA606693, TC391438, CK211707, TC412317, TC386963, TC412732, TC405695, TC392778, TC396751, TC397312, TC370350, TC405030, TC388403, TC373678, CA615187, TC416695, TC405041, TC385780, TC406469, TC405784, TC399919, TC407572, TC372175, TC369199, TC410690, TC406708, TC419747, TC409459, TC402186, TC386646, TC405356, TC423354, TC402603, TC441241, TC369348, TC449724, TC413571, TC397909, TC413043, CA729339, TC374268, CK214224, TC408299, TC376263, TC409187, TC384010, TC389993, TC416169, TC425690, TC381218, TC422425, TC383677, CA613620, TC388520, TC411191, TC399342, TC434831, TC432185, TC426326, TC381462, TC373914, TC392263, TC392303, TC435533, TC374240, TC425841, TC424204, TC387579, TC388914, TC393814, TC398633, TC394716, TC460689, TC446235, TC380943, TC393830, TC432154, TC403885, TC420735, TC404371, TC397964, TC427405, TC384219, TC386961, TC416685, TC387344, TC412483, TC389090, TC389375, TC382655, TC370114, CK200433, TC406038, TC395351, TC418032, TC456619, CK210754, TC384454, TC411908, TC380329, TC411287, TC386237, TC393904, TC390994, TC391143, TC434396, TC382742, TC398536, TC386414, TC395303, TC400755, TC440636, TC405511, TC403986, TC376420, TC458987, TC385365, TC430544, TC370633, TC401210, DR737360, TC379357, TC390135, TC386279, TC411684, TC382786, TC439225, TC380662, TC393960, TC430561, TC389162, DR739471, TC402534, TC443387, TC415083, TC406584, TC386519, TC374726, TC402440, TC417992, TC394307, TC375146, TC398592, TC389589, TC452762, TC386344, TC381963, TC383176, TC370044, TC386422, TC374164, TC385701, TC388688, TC452503, TC388547, TC388822, TC377496, TC388976, TC396365, TC429771, TC423576, TC386639, TC416658, TC379965, TC393561, TC393692, TC375726, TC391671, TC393554, TC407614, TC400477, TC397415, TC459656, TC382928, TC438243, TC371008, TC379436, TC417363, TC411941, TC403929, TC394118, TC380882, TC379635, TC423880, TC433589, TC377410, TC389816, TC372530, TC392323, TC413066, TC393820, TC461622, CK211469, CB307332, TC404842, TC403588, TC376933, TC397729, DR740372, TC403803, TC396895, TC440526, TC382342, TC393106, TC401758, TC432504, TC397676, TC396460, TC389661, TC428944 |
| [G-protein signaling, coupled to cAMP nucleotide second messenger](http://amigo.geneontology.org/cgi-bin/amigo/go.cgi?action=query&view=query&query=GO:0007188&search_constraint=terms) | TC410352, DR739471, TC434820, TC443387, TC379711, TC392272, CV775873, TC386313, TC398052, CA710880, TC425841, TC402440, TC387579, TC394459, TC371242, TC384738, TC423252, TC394307, TC397660, CK211707, TC375146, TC386963, TC389363, TC388914, TC388410, TC389589, TC446235, TC392778, TC397312, TC420735, TC395453, TC386422, TC386961, TC388688, TC390569, TC416685, TC403872, TC399919, TC407572, TC389090, TC372175, TC389375, TC388822, TC396365, TC406708, TC377496, TC419747, TC382655, TC429771, CK200433, TC423576, BQ294582, TC406038, TC416658, TC395351, TC401915, TC393561, TC393100, TC391671, TC393554, TC407614, TC382928, DR739994, TC384454, CA729339, TC386237, TC417363, TC393904, CK217367, TC379853, TC377410, TC381218, TC389816, TC395303, TC400755, TC405440, TC458987, CA613620, TC401210, DR737360, TC390135, TC397729, TC377225, TC434831, TC396895, TC426326, TC381462, TC393106, TC401758, BQ607161, TC392263, TC396460, TC428944, TC389661 |
| [G-protein signaling, coupled to cyclic nucleotide second messenger](http://amigo.geneontology.org/cgi-bin/amigo/go.cgi?action=query&view=query&query=GO:0007187&search_constraint=terms) | TC410352, DR739471, TC434820, TC443387, TC379711, TC392272, CV775873, TC386313, TC398052, CA710880, TC425841, TC402440, TC387579, TC394459, TC371242, TC384738, TC423252, TC394307, TC397660, CK211707, TC375146, TC386963, TC389363, TC388914, TC388410, TC389589, TC446235, TC392778, TC397312, TC420735, TC395453, TC386422, TC386961, TC388688, TC390569, TC416685, TC403872, TC399919, TC407572, TC389090, TC372175, TC389375, TC388822, TC396365, TC406708, TC377496, TC419747, TC382655, TC429771, CK200433, TC423576, BQ294582, TC406038, TC416658, TC395351, TC401915, TC393561, TC393100, TC391671, TC393554, TC407614, TC382928, DR739994, TC384454, CA729339, TC386237, TC417363, TC393904, CK217367, TC379853, TC377410, TC381218, TC389816, TC395303, TC400755, TC405440, TC458987, CA613620, TC401210, DR737360, TC390135, TC397729, TC377225, TC434831, TC396895, TC426326, TC381462, TC393106, TC401758, BQ607161, TC392263, TC396460, TC428944, TC389661 |
| [Cellular component organization at cellular level](http://amigo.geneontology.org/cgi-bin/amigo/go.cgi?action=query&view=query&query=GO:0071842&search_constraint=terms) | TC461921, TC410352, TC386040, TC390393, TC416529, TC397258, TC389190, CV775873, TC396230, TC375864, TC460760, TC427210, TC416069, BQ838511, TC398052, CA710880, TC400388, TC421871, TC433162, TC394459, TC371242, TC416154, TC384738, TC423252, TC397660, TC389363, TC379171, TC388410, CK203550, TC416493, TC458562, TC421914, TC406264, TC387410, TC380063, TC398538, TC410063, TC378054, TC448471, TC395453, TC410194, TC392875, TC409843, TC435546, TC384735, TC390569, TC405540, TC407076, TC419057, TC403872, TC425847, CJ727624, TC398714, BQ294582, CA614761, CJ550278, TC457126, TC403580, TC413427, TC411128, TC401915, TC397444, TC398304, TC425878, TC393100, TC433957, TC417012, CA632212, DR739994, TC459193, TC394916, TC394206, TC394820, TC406236, TC418073, TC378274, TC391130, TC417308, CK217367, TC409599, TC445767, TC446038, TC413460, TC379853, TC370315, BQ609416, CK214702, TC449504, TC395723, TC405440, CK213497, CJ792862, TC385233, TC389718, TC404052, TC377225, TC381943, TC412520, TC432001, TC413392, BQ607161, TC406807, TC382045, TC402308, TC434820, TC441343, TC379711, TC400330, TC400260, TC378790, CA595837, TC379903, TC423265, TC392272, TC377061, TC401244, CA730421, TC446465, TC386313, CV759879, TC408907, CA606693, TC391438, CK211707, TC386963, TC412732, TC405695, CA700201, TC392778, TC396751, TC397312, TC405030, CK211589, TC388403, TC376527, CA615187, TC416695, TC388718, TC419584, TC383701, TC385780, TC406469, TC405784, TC399919, TC407572, TC372175, TC410690, TC406708, TC419747, TC410074, TC402186, TC405356, CV763657, TC423354, TC402603, TC441241, TC413571, TC413043, CA729339, CK214224, TC408299, BQ239045, CA709177, TC376263, TC416169, TC425690, TC381218, TC408229, TC422425, TC408312, CA613620, TC398805, TC400362, CV781430, TC388520, TC411191, TC399342, TC434831, TC426326, TC381462, CD882425, TC373914, TC392263, TC392303, TC394796, TC445166, CA611770, TC435533, TC374240, CA720842, TC425841, CF554444, TC424204, TC387579, TC406870, TC388914, TC393814, TC376220, TC387621, TC398633, TC394716, TC446235, TC378271, TC380943, TC393830, TC380433, TC403885, TC420735, TC404371, TC384219, TC386961, TC416685, TC372664, TC387344, TC412483, TC389090, TC389375, TC404413, TC382655, CK200433, TC406038, TC393970, TC395351, TC418032, CK210754, TC410147, TC384454, TC411287, TC386237, TC393904, TC390994, TC391143, TC381463, TC403157, TC386414, TC395303, TC400755, TC440636, TC405511, TC403986, TC405615, TC458987, TC377190, TC404843, TC431879, CD876572, TC385365, TC401210, DR737360, TC379357, TC390135, TC387135, TC397885, TC386279, TC411684, TC411480, TC409650, TC380662, TC393960, TC389162, DR739471, TC402534, TC443387, TC415083, TC386535, TC390630, GH729256, TC406584, TC386519, CA605200, TC402440, TC417992, TC391411, TC394307, TC409208, TC388665, TC375146, TC389589, TC381988, TC452762, TC386344, TC373637, TC381963, TC383176, CD878039, TC374879, TC386422, TC388688, TC418845, TC388547, TC414606, TC388822, TC377496, TC388976, TC396365, TC429771, TC410078, TC423576, TC386639, TC416658, TC393561, TC393948, TC393692, TC391671, TC393554, TC407614, TC459656, TC382928, TC379436, TC417363, TC403573, TC413700, TC403929, TC449043, TC380882, TC379635, TC440321, TC423880, TC433589, TC377410, TC418091, TC389816, TC372530, TC392323, TC413066, TC391641, TC461622, CK211469, CB307332, TC404842, TC381817, TC403588, TC397729, TC403803, TC396895, TC382342, TC393106, TC401758, TC432504, TC397676, TC396460, TC428944, TC417067, TC389661 |
| [Regulation of cyclic nucleotide metabolic process](http://amigo.geneontology.org/cgi-bin/amigo/go.cgi?action=query&view=query&query=GO:0030799&search_constraint=terms) | TC410352, DR739471, TC434820, TC443387, TC379711, TC392272, CV775873, TC417077, TC386313, TC398052, CA710880, TC425841, TC402440, TC387579, TC394459, TC371242, TC384738, TC423252, TC394307, TC397660, CK211707, TC375146, TC386963, TC389363, TC388914, TC388410, TC389589, TC446235, TC392778, TC397312, TC420735, TC395453, TC386422, TC386961, TC388688, TC390569, TC416685, TC403872, TC399919, TC407572, TC389090, TC372175, TC389375, TC388822, TC396365, TC406708, TC377496, TC419747, TC382655, TC429771, CK200433, TC423576, BQ294582, TC406038, TC416658, TC395351, TC401915, TC393561, TC393100, TC391671, TC393554, TC407614, TC382928, DR739994, TC384454, CA729339, TC417363, TC386237, TC393904, CK217367, TC379853, TC377410, TC381218, TC389816, TC395303, TC400755, TC405440, TC458987, CA613620, TC401210, DR737360, TC390135, TC397729, TC377225, TC434831, TC396895, TC426326, TC381462, TC393106, TC401758, BQ607161, TC392263, TC396460, TC428944, TC389661 |
| [Regulation of cyclic nucleotide biosynthetic process](http://amigo.geneontology.org/cgi-bin/amigo/go.cgi?action=query&view=query&query=GO:0030802&search_constraint=terms) | TC410352, DR739471, TC434820, TC443387, TC379711, TC392272, CV775873, TC417077, TC386313, TC398052, CA710880, TC425841, TC402440, TC387579, TC394459, TC371242, TC384738, TC423252, TC394307, TC397660, CK211707, TC375146, TC386963, TC389363, TC388914, TC388410, TC389589, TC446235, TC392778, TC397312, TC420735, TC395453, TC386422, TC386961, TC388688, TC390569, TC416685, TC403872, TC399919, TC407572, TC389090, TC372175, TC389375, TC388822, TC396365, TC406708, TC377496, TC419747, TC382655, TC429771, CK200433, TC423576, BQ294582, TC406038, TC416658, TC395351, TC401915, TC393561, TC393100, TC391671, TC393554, TC407614, TC382928, DR739994, TC384454, CA729339, TC417363, TC386237, TC393904, CK217367, TC379853, TC377410, TC381218, TC389816, TC395303, TC400755, TC405440, TC458987, CA613620, TC401210, DR737360, TC390135, TC397729, TC377225, TC434831, TC396895, TC426326, TC381462, TC393106, TC401758, BQ607161, TC392263, TC396460, TC428944, TC389661 |
| [Regulation of nucleotide biosynthetic process](http://amigo.geneontology.org/cgi-bin/amigo/go.cgi?action=query&view=query&query=GO:0030808&search_constraint=terms) | TC410352, DR739471, TC434820, TC443387, TC379711, TC392272, CV775873, TC417077, TC386313, TC398052, CA710880, TC425841, TC402440, TC387579, TC394459, TC371242, TC384738, TC423252, TC394307, TC397660, CK211707, TC375146, TC386963, TC389363, TC388914, TC388410, TC389589, TC446235, TC392778, TC397312, TC420735, TC395453, TC386422, TC386961, TC388688, TC390569, TC416685, TC403872, TC399919, TC407572, TC389090, TC372175, TC389375, TC388822, TC396365, TC406708, TC377496, TC419747, TC382655, TC429771, CK200433, TC423576, BQ294582, TC406038, TC416658, TC395351, TC401915, TC393561, TC393100, TC391671, TC393554, TC407614, TC382928, DR739994, TC384454, CA729339, TC417363, TC386237, TC393904, CK217367, TC379853, TC377410, TC381218, TC389816, TC395303, TC400755, TC405440, TC458987, CA613620, TC401210, DR737360, TC390135, TC397729, TC377225, TC434831, TC396895, TC426326, TC381462, TC393106, TC401758, BQ607161, TC392263, TC396460, TC428944, TC389661 |
| [Regulation of cAMP metabolic process](http://amigo.geneontology.org/cgi-bin/amigo/go.cgi?action=query&view=query&query=GO:0030814&search_constraint=terms) | TC410352, DR739471, TC434820, TC443387, TC379711, TC392272, CV775873, TC417077, TC386313, TC398052, CA710880, TC425841, TC402440, TC387579, TC394459, TC371242, TC384738, TC423252, TC394307, TC397660, CK211707, TC375146, TC386963, TC389363, TC388914, TC388410, TC389589, TC446235, TC392778, TC397312, TC420735, TC395453, TC386422, TC386961, TC388688, TC390569, TC416685, TC403872, TC399919, TC407572, TC389090, TC372175, TC389375, TC388822, TC396365, TC406708, TC377496, TC419747, TC382655, TC429771, CK200433, TC423576, BQ294582, TC406038, TC416658, TC395351, TC401915, TC393561, TC393100, TC391671, TC393554, TC407614, TC382928, DR739994, TC384454, CA729339, TC417363, TC386237, TC393904, CK217367, TC379853, TC377410, TC381218, TC389816, TC395303, TC400755, TC405440, TC458987, CA613620, TC401210, DR737360, TC390135, TC397729, TC377225, TC434831, TC396895, TC426326, TC381462, TC393106, TC401758, BQ607161, TC392263, TC396460, TC428944, TC389661 |
| [Regulation of cAMP biosynthetic process](http://amigo.geneontology.org/cgi-bin/amigo/go.cgi?action=query&view=query&query=GO:0030817&search_constraint=terms) | TC410352, DR739471, TC434820, TC443387, TC379711, TC392272, CV775873, TC417077, TC386313, TC398052, CA710880, TC425841, TC402440, TC387579, TC394459, TC371242, TC384738, TC423252, TC394307, TC397660, CK211707, TC375146, TC386963, TC389363, TC388914, TC388410, TC389589, TC446235, TC392778, TC397312, TC420735, TC395453, TC386422, TC386961, TC388688, TC390569, TC416685, TC403872, TC399919, TC407572, TC389090, TC372175, TC389375, TC388822, TC396365, TC406708, TC377496, TC419747, TC382655, TC429771, CK200433, TC423576, BQ294582, TC406038, TC416658, TC395351, TC401915, TC393561, TC393100, TC391671, TC393554, TC407614, TC382928, DR739994, TC384454, CA729339, TC417363, TC386237, TC393904, CK217367, TC379853, TC377410, TC381218, TC389816, TC395303, TC400755, TC405440, TC458987, CA613620, TC401210, DR737360, TC390135, TC397729, TC377225, TC434831, TC396895, TC426326, TC381462, TC393106, TC401758, BQ607161, TC392263, TC396460, TC428944, TC389661 |
| [Regulation of cyclase activity](http://amigo.geneontology.org/cgi-bin/amigo/go.cgi?action=query&view=query&query=GO:0031279&search_constraint=terms) | TC410352, DR739471, TC434820, TC443387, TC379711, TC392272, CV775873, TC417077, TC386313, TC398052, CA710880, TC425841, TC402440, TC387579, TC394459, TC371242, TC384738, TC423252, TC394307, TC397660, CK211707, TC375146, TC386963, TC389363, TC388914, TC388410, TC389589, TC446235, TC392778, TC397312, TC420735, TC395453, TC386422, TC386961, TC388688, TC390569, TC416685, TC403872, TC399919, TC407572, TC389090, TC372175, TC389375, TC388822, TC396365, TC406708, TC377496, TC419747, TC382655, TC429771, CK200433, TC423576, BQ294582, TC406038, TC416658, TC395351, TC401915, TC393561, TC393100, TC391671, TC393554, TC407614, TC382928, DR739994, TC384454, CA729339, TC417363, TC386237, TC393904, CK217367, TC379853, TC377410, TC381218, TC389816, TC395303, TC400755, TC405440, TC458987, CA613620, TC401210, DR737360, TC390135, TC397729, TC377225, TC434831, TC396895, TC426326, TC381462, TC393106, TC401758, BQ607161, TC392263, TC396460, TC428944, TC389661 |
| [Regulation of adenylate cyclase activity](http://amigo.geneontology.org/cgi-bin/amigo/go.cgi?action=query&view=query&query=GO:0045761&search_constraint=terms) | TC410352, DR739471, TC434820, TC443387, TC379711, TC392272, CV775873, TC417077, TC386313, TC398052, CA710880, TC425841, TC402440, TC387579, TC394459, TC371242, TC384738, TC423252, TC394307, TC397660, CK211707, TC375146, TC386963, TC389363, TC388914, TC388410, TC389589, TC446235, TC392778, TC397312, TC420735, TC395453, TC386422, TC386961, TC388688, TC390569, TC416685, TC403872, TC399919, TC407572, TC389090, TC372175, TC389375, TC388822, TC396365, TC406708, TC377496, TC419747, TC382655, TC429771, CK200433, TC423576, BQ294582, TC406038, TC416658, TC395351, TC401915, TC393561, TC393100, TC391671, TC393554, TC407614, TC382928, DR739994, TC384454, CA729339, TC417363, TC386237, TC393904, CK217367, TC379853, TC377410, TC381218, TC389816, TC395303, TC400755, TC405440, TC458987, CA613620, TC401210, DR737360, TC390135, TC397729, TC377225, TC434831, TC396895, TC426326, TC381462, TC393106, TC401758, BQ607161, TC392263, TC396460, TC428944, TC389661 |
| [Regulation of lyase activity](http://amigo.geneontology.org/cgi-bin/amigo/go.cgi?action=query&view=query&query=GO:0051339&search_constraint=terms) | TC410352, DR739471, TC434820, TC443387, TC379711, TC392272, CV775873, TC417077, TC386313, TC398052, CA710880, TC425841, TC402440, TC387579, TC394459, TC371242, TC384738, TC423252, TC394307, TC397660, CK211707, TC375146, TC386963, TC389363, TC388914, TC388410, TC389589, TC446235, TC392778, TC397312, TC420735, TC395453, TC386422, TC386961, TC388688, TC390569, TC416685, TC403872, TC399919, TC407572, TC389090, TC372175, TC389375, TC388822, TC396365, TC406708, TC377496, TC419747, TC382655, TC429771, CK200433, TC423576, BQ294582, TC406038, TC416658, TC395351, TC401915, TC393561, TC393100, TC391671, TC393554, TC407614, TC382928, DR739994, TC384454, CA729339, TC417363, TC386237, TC393904, CK217367, TC379853, TC377410, TC381218, TC389816, TC395303, TC400755, TC405440, TC458987, CA613620, TC401210, DR737360, TC390135, TC397729, TC377225, TC434831, TC396895, TC426326, TC381462, TC393106, TC401758, BQ607161, TC392263, TC396460, TC428944, TC389661 |
| [Regulation of nucleotide metabolic process](http://amigo.geneontology.org/cgi-bin/amigo/go.cgi?action=query&view=query&query=GO:0006140&search_constraint=terms) | TC410352, DR739471, TC434820, TC443387, TC379711, TC392272, CV775873, TC417077, TC386313, TC398052, CA710880, TC425841, TC402440, TC387579, TC394459, TC371242, TC384738, TC423252, TC394307, TC397660, CK211707, TC375146, TC386963, TC389363, TC388914, TC388410, TC389589, TC446235, TC392778, TC397312, TC420735, TC395453, TC386422, TC386961, TC388688, TC390569, TC416685, TC403872, TC399919, TC407572, TC389090, TC372175, TC389375, TC388822, TC396365, TC406708, TC377496, TC419747, TC382655, TC429771, CK200433, TC423576, BQ294582, TC406038, TC416658, TC395351, TC401915, TC393561, TC393100, TC391671, TC393554, TC407614, TC382928, DR739994, TC384454, CA729339, TC417363, TC386237, TC393904, CK217367, TC379853, TC377410, TC381218, TC389816, TC395303, TC400755, TC405440, TC458987, CA613620, TC401210, DR737360, TC390135, TC397729, TC377225, TC434831, TC396895, TC426326, TC381462, TC393106, TC401758, BQ607161, TC392263, TC396460, TC428944, TC389661 |
| [Sensory perception](http://amigo.geneontology.org/cgi-bin/amigo/go.cgi?action=query&view=query&query=GO:0007600&search_constraint=terms) | TC410352, DR739471, TC434820, TC416906, TC443387, TC379711, TC392272, CV775873, TC377061, TC417388, TC386313, TC398052, TC425841, TC402440, TC398862, TC387579, TC394459, TC384738, TC423252, TC394307, TC397660, CK211707, TC375146, TC386963, TC389363, TC388914, TC379171, TC388410, CK199175, TC389589, TC446235, TC418414, TC392778, TC397312, TC420735, TC383909, TC449463, TC395453, TC386422, TC386961, TC388688, TC390569, TC416685, TC407076, TC403872, TC399919, TC407572, TC389090, TC372175, TC389375, TC388822, TC396365, TC406708, TC377496, TC419747, TC382655, TC429771, CK200433, TC423576, TC409459, TC370114, TC406038, TC416658, TC395351, TC386646, TC401915, TC393561, TC393100, TC391671, TC393554, TC407614, TC382928, TC459656, DR739994, TC438243, TC384454, CA729339, TC417363, TC386237, TC393904, CK217367, TC379853, TC377410, TC381218, TC389816, TC395303, TC400755, TC405440, TC458987, TC401210, DR737360, TC390135, TC397729, TC377225, TC434831, TC432185, TC396895, TC426326, TC381462, TC393106, TC401758, BQ607161, TC406807, TC396460, TC392263, TC428944, TC389661, TC382045 |
| [Signaling process](http://amigo.geneontology.org/cgi-bin/amigo/go.cgi?action=query&view=query&query=GO:0023046&search_constraint=terms) | TC410352, TC386040, TC407978, TC394796, TC397258, CV775873, TC435533, TC374240, TC398052, CA710880, TC425841, TC433162, TC387579, TC394459, TC371242, TC384738, TC423252, TC397660, TC389363, TC388914, TC388410, TC458562, TC394716, TC398633, TC421914, TC446235, TC387410, TC380063, TC378271, TC393830, TC380943, TC380433, TC378054, TC420735, TC404371, TC395453, TC386961, TC384219, TC392329, TC398606, TC384735, TC390569, TC416685, TC387344, TC407076, TC419057, TC403872, TC389090, TC389375, TC404413, TC382655, CK200433, TC398714, BQ294582, TC406038, CJ550278, TC393970, TC395351, TC403580, TC401915, TC398304, TC393100, TC433957, CK210754, CA632212, DR739994, TC384454, TC411287, TC380329, TC386237, TC394916, TC393904, TC390994, TC378274, TC391143, CK217367, TC391128, TC409599, TC379853, TC370315, TC395303, TC386414, TC400755, TC395723, TC440636, TC405440, TC403968, TC376420, TC458987, TC423110, TC379536, TC389718, TC401210, TC390135, TC398514, DR737360, TC379357, TC377225, TC386279, TC409650, TC381943, TC380662, BQ607161, TC393960, TC382045, CA646741, DR739471, TC402308, TC434820, TC443387, TC441343, TC379711, TC378790, TC392272, TC377061, TC417077, TC386519, TC386313, CV759879, TC413339, TC408907, TC402440, CA606693, TC420579, TC391438, TC394307, CK211707, TC375146, TC386963, TC398592, TC389589, TC421345, TC386344, TC392778, TC396751, TC397312, TC405030, TC381963, CK211589, TC388403, TC383176, CA615187, TC386422, TC388688, TC405041, TC385780, TC388547, TC405784, TC399919, TC407572, TC372175, TC388822, TC396365, TC377496, TC388976, TC406708, TC419747, TC429771, TC423576, TC386639, TC402186, TC416658, TC381957, TC386646, TC393561, TC393692, TC391671, TC393554, TC407614, TC382928, TC402603, TC438243, TC441241, TC413571, TC379436, CA729339, TC417363, CK214224, TC403929, BE585841, TC425690, TC373259, TC433589, TC377410, TC381218, TC389816, TC422425, TC372530, CA613620, CK211469, TC400362, TC397729, TC411191, TC434831, TC396895, TC432185, TC426326, TC381462, TC382342, TC393106, TC401758, TC373914, TC432504, TC397676, TC396460, TC392263, TC428944, TC389661 |
| [Signal transmission](http://amigo.geneontology.org/cgi-bin/amigo/go.cgi?action=query&view=query&query=GO:0023060&search_constraint=terms) | TC410352, TC386040, TC407978, TC394796, TC397258, CV775873, TC435533, TC374240, TC398052, CA710880, TC425841, TC433162, TC387579, TC394459, TC371242, TC384738, TC423252, TC397660, TC389363, TC388914, TC388410, TC458562, TC394716, TC398633, TC421914, TC446235, TC387410, TC380063, TC378271, TC393830, TC380943, TC380433, TC378054, TC420735, TC404371, TC395453, TC386961, TC384219, TC392329, TC398606, TC384735, TC390569, TC416685, TC387344, TC407076, TC419057, TC403872, TC389090, TC389375, TC404413, TC382655, CK200433, TC398714, BQ294582, TC406038, CJ550278, TC393970, TC395351, TC403580, TC401915, TC398304, TC393100, TC433957, CK210754, CA632212, DR739994, TC384454, TC411287, TC380329, TC386237, TC394916, TC393904, TC390994, TC378274, TC391143, CK217367, TC391128, TC409599, TC379853, TC370315, TC395303, TC386414, TC400755, TC395723, TC440636, TC405440, TC403968, TC376420, TC458987, TC423110, TC379536, TC389718, TC401210, TC390135, TC398514, DR737360, TC379357, TC377225, TC386279, TC409650, TC381943, TC380662, BQ607161, TC393960, TC382045, CA646741, DR739471, TC402308, TC434820, TC443387, TC441343, TC379711, TC378790, TC392272, TC377061, TC417077, TC386519, TC386313, CV759879, TC413339, TC408907, TC402440, CA606693, TC420579, TC391438, TC394307, CK211707, TC375146, TC386963, TC398592, TC389589, TC421345, TC386344, TC392778, TC396751, TC397312, TC405030, TC381963, CK211589, TC388403, TC383176, CA615187, TC386422, TC388688, TC405041, TC385780, TC388547, TC405784, TC399919, TC407572, TC372175, TC388822, TC396365, TC377496, TC388976, TC406708, TC419747, TC429771, TC423576, TC386639, TC402186, TC416658, TC381957, TC386646, TC393561, TC393692, TC391671, TC393554, TC407614, TC382928, TC402603, TC438243, TC441241, TC413571, TC379436, CA729339, TC417363, CK214224, TC403929, BE585841, TC425690, TC373259, TC433589, TC377410, TC381218, TC389816, TC422425, TC372530, CA613620, CK211469, TC400362, TC397729, TC411191, TC434831, TC396895, TC432185, TC426326, TC381462, TC382342, TC393106, TC401758, TC373914, TC432504, TC397676, TC396460, TC392263, TC428944, TC389661 |
| [Neurological system process](http://amigo.geneontology.org/cgi-bin/amigo/go.cgi?action=query&view=query&query=GO:0050877&search_constraint=terms) | TC410352, CV775873, TC374240, TC398052, TC425841, TC398862, TC387579, TC394459, TC384738, TC423252, TC397660, TC389363, TC388914, TC379171, TC388410, TC458562, TC421914, TC446235, TC378271, TC418414, TC420735, TC395453, TC386961, TC390569, TC416685, TC387344, TC407076, TC403872, TC389090, TC389375, TC382655, CK200433, TC398714, TC370114, TC406038, CJ550278, TC393970, TC395351, TC401915, TC398304, TC433957, TC393100, CK210754, DR739994, TC384454, TC386237, TC394916, TC393904, TC390994, TC378274, CK217367, TC379853, TC395303, TC400755, TC395723, TC405440, TC458987, TC401210, DR737360, TC398514, TC390135, TC377225, TC409650, TC380662, BQ607161, TC406807, TC382045, DR739471, TC416906, TC434820, TC443387, TC379711, TC392272, TC377061, TC417388, TC386313, TC402440, CA606693, TC394307, TC375146, CK211707, TC386963, CK199175, TC389589, TC392778, TC397312, TC396751, CK211589, TC383909, CA615187, TC449463, TC386422, TC388688, TC388547, TC405784, TC372175, TC407572, TC399919, TC388822, TC377496, TC406708, TC396365, TC419747, TC429771, TC409459, TC423576, TC416658, TC386646, TC393561, TC391671, TC393554, TC407614, TC459656, TC382928, TC441241, TC438243, TC413571, CA729339, TC417363, TC403929, TC377410, TC381218, TC389816, TC422425, TC397729, TC411191, TC432185, TC396895, TC434831, TC426326, TC381462, TC393106, TC401758, TC392263, TC396460, TC389661, TC428944 |
| [Nucleic acid metabolic process](http://amigo.geneontology.org/cgi-bin/amigo/go.cgi?action=query&view=query&query=GO:0090304&search_constraint=terms) | TC392303, TC410352, TC386040, TC390393, TC397258, TC425957, TC375864, TC445166, TC435533, TC374240, TC375313, CA710880, TC398862, TC433162, TC387579, TC371242, TC423252, TC397660, TC393814, TC389363, TC388914, TC376220, TC458562, TC394716, TC398633, TC446235, TC387410, TC378878, TC380063, TC393830, TC397176, TC380943, TC418414, TC380433, TC378054, TC420735, TC404371, TC395453, TC386961, TC384219, TC384735, TC390569, TC405540, TC416685, TC372664, TC419057, TC403872, TC389090, TC389375, TC382655, CK200433, TC373791, TC398714, TC373994, TC370114, BQ294582, TC406038, TC395351, TC403580, TC401915, GH732878, CK210754, CA632212, DR739994, TC384454, TC411287, TC394916, EB512907, TC390994, TC378274, TC391143, CK217367, TC423804, TC379853, TC370315, TC386414, TC400755, TC405511, TC395723, TC440636, TC405440, TC377190, TC443814, TC404843, TC385233, TC389718, TC401210, DR737360, TC379357, TC387135, TC377225, TC386279, TC381943, BQ607161, TC393960, TC404606, TC369628, TC389162, DR739471, TC416906, TC402308, TC434820, TC443387, TC441343, TC379711, TC388566, TC379903, TC392272, TC377061, TC417388, TC386519, TC386313, CV759879, TC408907, TC402440, TC391438, TC417341, TC394307, TC409208, TC375146, TC386963, TC454407, TC389589, TC381988, TC452762, TC386344, TC392778, TC397312, TC405030, TC381963, TC388403, TC383176, TC371455, TC386422, TC374164, TC388688, TC385780, TC388822, TC377496, TC368549, TC388976, TC406708, TC429771, TC410078, TC409459, TC423576, TC386639, TC397500, TC402186, TC416658, TC393561, TC393692, TC391671, TC393554, TC407614, TC458205, TC402603, TC423354, TC441241, TC449724, TC413571, TC379436, CA729339, CK214224, TC387710, TC394118, TC380882, TC423880, TC433589, TC377410, TC381218, TC418091, TC389816, TC399352, CA613620, TC393820, CK211469, TC369092, TC403588, TC397729, TC434831, TC396895, TC426326, TC381462, TC382342, TC393106, TC401758, TC373914, TC432504, TC392263, TC397676, TC396460, TC428944, TC389661 |
| [Defense response to Gram-negative bacterium](http://amigo.geneontology.org/cgi-bin/amigo/go.cgi?action=query&view=query&query=GO:0050829&search_constraint=terms) | TC392303, TC398304, TC393100, TC433957, CK210754, TC382928, CV775873, TC441241, TC417363, TC386237, TC394916, TC386313, TC393904, TC390994, TC398052, TC425841, TC378274, TC403929, TC387579, CA606693, TC394459, TC384738, CK211707, TC386963, TC388410, TC377410, TC381218, TC395303, TC422425, TC458562, TC400755, TC458987, TC396751, CA615187, TC388520, TC401210, TC386961, TC390135, TC397729, TC377225, TC411191, TC388547, TC387344, TC405784, TC399919, TC407572, TC372175, TC381462, TC396365, TC419747, TC380662, TC401758, TC398714, TC392263, TC389661 |
| [Defense response to Gram-positive bacterium](http://amigo.geneontology.org/cgi-bin/amigo/go.cgi?action=query&view=query&query=GO:0050830&search_constraint=terms) | TC392303, TC398304, TC393100, TC433957, CK210754, TC382928, CV775873, TC441241, TC417363, TC386237, TC394916, TC386313, TC393904, TC390994, TC398052, TC425841, TC378274, TC403929, TC387579, CA606693, TC394459, TC384738, CK211707, TC386963, TC388410, TC377410, TC381218, TC395303, TC422425, TC458562, TC400755, TC458987, TC396751, CA615187, TC388520, TC401210, TC386961, TC390135, TC397729, TC377225, TC411191, TC388547, TC387344, TC405784, TC399919, TC407572, TC372175, TC381462, TC396365, TC419747, TC380662, TC401758, TC398714, TC392263, TC389661 |
| [Cellular component organization or biogenesis at cellular level](http://amigo.geneontology.org/cgi-bin/amigo/go.cgi?action=query&view=query&query=GO:0071841&search_constraint=terms) | TC461921, TC410352, TC386040, TC379942, TC407978, TC390393, TC416529, TC397258, TC389190, CV775873, TC396230, TC375864, TC460760, TC427210, TC416069, BQ838511, TC398052, CA710880, TC400388, TC421871, TC398862, TC433162, TC394459, TC371242, TC416154, TC384738, TC423252, TC397660, TC389363, TC379171, TC388410, CK203550, TC416493, TC458562, TC421914, TC406264, TC387410, TC380063, TC398538, TC418414, TC410063, TC378054, TC448471, TC395453, TC410194, TC392875, TC409843, TC435546, TC384735, TC390569, TC405540, TC407076, TC419057, TC403872, TC425847, TC391948, CJ727624, TC398714, BQ294582, CA614761, CJ550278, TC381619, TC457126, TC403580, TC413427, TC411128, TC401915, TC397444, TC398304, TC425878, TC393100, TC433957, TC417012, CA632212, DR739994, TC459193, TC394916, TC394206, TC394820, TC406236, TC418073, TC378274, TC391130, TC417308, CK217367, TC409599, TC445767, TC446038, TC423804, TC413460, TC379853, TC370315, BQ609416, CK214702, TC449504, TC395723, TC405440, CK213497, CJ792862, TC385233, TC389718, TC404052, TC377225, TC381943, TC412520, TC432001, TC413392, BQ607161, TC406807, TC382045, TC404606, TC416906, TC402308, TC434820, TC441343, TC379711, TC400330, TC400260, TC378790, CA595837, TC379903, TC423265, TC392272, TC377061, TC401244, CA730421, TC417388, TC446465, TC386313, CV759879, TC408907, CA606693, TC391438, CK211707, TC386963, TC412732, TC405695, CA700201, TC392778, TC396751, TC397312, TC405030, CK211589, TC388403, TC376527, CA615187, TC416695, TC388718, TC419584, TC383701, TC406469, TC385780, TC405784, TC399919, TC407572, TC372175, TC410690, TC406708, TC419747, TC410074, TC402186, TC405356, CV763657, TC423354, TC402603, TC441241, TC433557, TC413571, TC413043, CA729339, CK214224, TC408299, BQ239045, CA709177, TC376263, TC416169, TC425690, TC381218, TC399352, TC408229, TC422425, TC408312, CA613620, TC398805, TC400362, CV781430, TC388520, TC411191, TC400108, TC399342, TC434831, TC426326, TC381462, CD882425, TC373914, TC392263, TC392303, TC394796, TC445166, CA611770, TC435533, TC374240, CA720842, TC425841, CF554444, TC424204, TC387579, TC406870, TC388914, TC393814, TC376220, TC387621, TC398633, TC394716, TC446235, TC402545, TC378271, TC380943, TC393830, TC380433, TC403885, TC420735, TC435281, TC404371, TC384219, TC386961, TC416685, TC387344, TC372664, TC389090, TC412483, TC389375, TC404413, TC382655, CK200433, TC406038, TC393970, TC395351, TC418032, CK210754, TC410147, TC376490, TC384454, TC411287, TC386237, TC393904, TC390994, TC391143, TC381463, TC403157, TC410066, TC395303, TC386414, TC400755, TC403986, TC440636, TC405511, TC405615, TC458987, TC377190, TC404843, TC431879, TC391613, CD876572, TC385365, TC382737, TC401210, TC369736, DR737360, TC379357, TC390135, TC387135, TC386279, TC397885, TC411684, TC411480, TC409650, TC380662, TC393960, TC395298, TC389162, DR739471, TC402534, TC443387, TC415083, TC390630, TC386535, GH729256, TC406584, TC386519, CA605200, TC402440, TC417992, TC391411, TC417341, TC394307, TC409208, TC375146, TC388665, TC389589, TC381988, TC452762, TC405475, TC386344, TC373637, TC381963, TC383176, TC370044, CD878039, TC374879, TC386422, TC403328, TC388688, TC418845, TC388547, TC414606, TC388822, TC396365, TC377496, TC388976, TC429771, TC410078, TC423576, TC386639, TC416658, TC393561, TC393948, TC393692, TC391671, TC393554, TC407614, TC459656, TC382928, TC379436, TC417363, TC403573, TC413700, TC403929, TC394118, TC449043, TC380882, TC423880, TC379635, TC440321, TC373259, TC433589, TC377410, TC418091, TC389816, TC372530, TC392323, TC413066, TC391641, TC461622, CK211469, CB307332, TC404842, TC381817, TC403588, TC397729, TC403803, TC396895, TC382342, TC393106, TC401758, TC432504, TC397676, TC396460, TC428944, TC389661, TC417067 |
| [Cell-cell signaling](http://amigo.geneontology.org/cgi-bin/amigo/go.cgi?action=query&view=query&query=GO:0007267&search_constraint=terms) | TC410352, DR739471, TC434820, TC443387, TC441343, TC379711, TC392272, CV775873, TC386313, TC398052, CA710880, TC425841, TC402440, TC387579, CA606693, TC394459, TC384738, TC423252, TC394307, TC397660, CK211707, TC375146, TC386963, TC389363, TC388914, TC388410, TC389589, TC458562, TC446235, TC392778, TC396751, TC397312, CK211589, TC420735, CA615187, TC395453, TC386422, TC386961, TC388688, TC384735, TC390569, TC388547, TC416685, TC387344, TC407076, TC403872, TC405784, TC399919, TC407572, TC389090, TC372175, TC389375, TC388822, TC396365, TC406708, TC377496, TC419747, TC382655, TC429771, CK200433, TC423576, TC398714, BQ294582, TC406038, TC393970, TC416658, TC395351, TC401915, TC393561, TC398304, TC433957, TC393100, CK210754, TC391671, TC393554, TC407614, TC382928, TC441241, DR739994, TC384454, TC413571, CA729339, TC417363, TC386237, TC394916, TC393904, TC390994, TC378274, TC403929, CK217367, TC379853, TC377410, TC381218, TC395303, TC389816, TC422425, TC400755, TC405440, TC376420, TC458987, CA613620, TC401210, DR737360, TC398514, TC390135, TC397729, TC377225, TC411191, TC434831, TC396895, TC409650, TC426326, TC381462, TC393106, TC380662, TC401758, BQ607161, TC396460, TC392263, TC428944, TC389661, TC382045 |
| [Intracellular signal transduction](http://amigo.geneontology.org/cgi-bin/amigo/go.cgi?action=query&view=query&query=GO:0035556&search_constraint=terms) | TC410352, TC386040, TC407978, TC397258, CV775873, TC435533, TC374240, TC398052, CA710880, TC425841, TC433162, TC387579, TC394459, TC371242, TC384738, TC423252, TC397660, TC389363, TC388914, TC388410, TC394716, TC398633, TC446235, TC387410, TC380063, TC393830, TC380943, TC378054, TC420735, TC404371, TC395453, TC386961, TC384219, TC390569, TC416685, TC407076, TC419057, TC403872, TC389090, TC389375, TC404413, TC382655, CK200433, BQ294582, TC406038, TC393970, TC395351, TC401915, TC393100, CA632212, DR739994, TC384454, TC411287, TC386237, TC393904, TC391143, CK217367, TC409599, TC379853, TC395303, TC386414, TC400755, TC440636, TC395723, TC405440, TC376420, TC458987, TC389718, TC401210, TC390135, DR737360, TC379357, TC377225, TC386279, TC381943, BQ607161, TC393960, TC382045, DR739471, TC402308, TC434820, TC443387, TC379711, TC378790, TC392272, TC377061, TC386519, CV759879, TC386313, TC408907, TC402440, TC391438, TC394307, CK211707, TC375146, TC386963, TC389589, TC386344, TC392778, TC405030, TC397312, TC381963, CK211589, TC388403, TC383176, TC386422, TC388688, TC385780, TC399919, TC407572, TC372175, TC388822, TC377496, TC388976, TC406708, TC396365, TC419747, TC429771, TC423576, TC386639, TC402186, TC416658, TC393561, TC393692, TC391671, TC393554, TC407614, TC402603, TC382928, TC438243, TC413571, TC379436, CA729339, TC417363, CK214224, BE585841, TC425690, TC373259, TC433589, TC377410, TC381218, TC389816, TC372530, CA613620, CK211469, TC400362, TC397729, TC432185, TC396895, TC434831, TC426326, TC382342, TC381462, TC393106, TC401758, TC373914, TC432504, TC392263, TC397676, TC396460, TC389661, TC428944 |
| [Negative regulation of transcription from RNA polymerase II promoter, global](http://amigo.geneontology.org/cgi-bin/amigo/go.cgi?action=query&view=query&query=GO:0045816&search_constraint=terms) | TC392303, TC393692, TC390393, CK210754, TC379903, TC397258, CA632212, TC402603, TC441241, TC379436, TC411287, TC435533, TC394916, TC386313, CV759879, CK214224, TC390994, TC408907, TC378274, TC387579, TC391438, TC386963, TC393814, TC433589, TC377410, TC381218, TC458562, TC400755, TC452762, CK211469, TC397312, TC388403, TC404371, TC401210, TC386961, TC384219, TC379357, TC397729, TC377225, TC386279, TC419057, TC381943, TC381462, TC382342, TC401758, TC398714, TC392263, TC402186, TC389661 |
| [Intracellular signaling pathway](http://amigo.geneontology.org/cgi-bin/amigo/go.cgi?action=query&view=query&query=GO:0023034&search_constraint=terms) | TC410352, TC386040, TC407978, TC397258, CV775873, TC435533, TC374240, TC398052, CA710880, TC425841, TC433162, TC387579, TC394459, TC371242, TC384738, TC423252, TC397660, TC389363, TC388914, TC388410, TC394716, TC398633, TC446235, TC387410, TC380063, TC393830, TC380943, TC380433, TC378054, TC420735, TC404371, TC395453, TC386961, TC384219, TC390569, TC416685, TC407076, TC419057, TC403872, TC389090, TC389375, TC404413, TC382655, CK200433, BQ294582, TC406038, TC393970, TC395351, TC401915, TC393100, CA632212, DR739994, TC384454, TC411287, TC386237, TC393904, TC391143, CK217367, TC409599, TC379853, TC395303, TC386414, TC400755, TC405511, TC440636, TC395723, TC405440, TC376420, TC458987, TC389718, TC401210, TC390135, DR737360, TC379357, TC377225, TC386279, TC381943, BQ607161, TC393960, TC382045, DR739471, TC402308, TC434820, TC443387, TC379711, TC378790, TC392272, TC377061, TC386519, CV759879, TC386313, TC408907, TC402440, TC391438, TC394307, CK211707, TC375146, TC386963, TC389589, TC386344, TC392778, TC405030, TC397312, TC381963, CK211589, TC388403, TC383176, TC386422, TC388688, TC385780, TC399919, TC407572, TC372175, TC388822, TC377496, TC388976, TC406708, TC396365, TC419747, TC429771, TC423576, TC386639, TC402186, TC416658, TC393561, TC393692, TC391671, TC393554, TC407614, TC402603, TC382928, TC423354, TC438243, TC413571, TC379436, CA729339, TC417363, CK214224, BE585841, TC425690, TC373259, TC433589, TC377410, TC381218, TC389816, TC372530, CA613620, CK211469, TC400362, TC397729, TC432185, TC396895, TC434831, TC426326, TC382342, TC381462, TC393106, TC401758, TC373914, TC432504, TC392263, TC397676, TC396460, TC389661, TC428944 |
| [Regulation of transcription from RNA polymerase II promoter, global](http://amigo.geneontology.org/cgi-bin/amigo/go.cgi?action=query&view=query&query=GO:0006358&search_constraint=terms) | TC392303, TC393692, TC390393, CK210754, TC379903, TC397258, CA632212, TC402603, TC441241, TC379436, TC411287, TC435533, TC394916, TC386313, CV759879, CK214224, TC390994, TC408907, TC378274, TC387579, TC391438, TC386963, TC393814, TC433589, TC377410, TC381218, TC458562, TC400755, TC452762, CK211469, TC397312, TC388403, TC404371, TC401210, TC386961, TC384219, TC379357, TC397729, TC377225, TC386279, TC419057, TC381943, TC381462, TC382342, TC401758, TC398714, TC392263, TC402186, TC389661 |
| [cAMP-mediated signaling](http://amigo.geneontology.org/cgi-bin/amigo/go.cgi?action=query&view=query&query=GO:0019933&search_constraint=terms) | TC410352, DR739471, TC434820, TC443387, TC379711, TC392272, CV775873, TC386313, TC398052, CA710880, TC425841, TC402440, TC387579, TC394459, TC371242, TC384738, TC423252, TC394307, TC397660, CK211707, TC375146, TC386963, TC389363, TC388914, TC388410, TC389589, TC446235, TC392778, TC397312, TC420735, TC395453, TC386422, TC386961, TC388688, TC390569, TC416685, TC403872, TC399919, TC407572, TC389090, TC372175, TC389375, TC388822, TC396365, TC406708, TC377496, TC419747, TC382655, TC429771, CK200433, TC423576, BQ294582, TC406038, TC393970, TC416658, TC395351, TC401915, TC393561, TC393100, TC391671, TC393554, TC407614, TC382928, DR739994, TC384454, CA729339, TC386237, TC417363, TC393904, CK217367, TC379853, TC377410, TC381218, TC389816, TC395303, TC400755, TC405440, TC458987, CA613620, TC401210, DR737360, TC390135, TC397729, TC377225, TC434831, TC396895, TC426326, TC381462, TC393106, TC401758, BQ607161, TC392263, TC396460, TC428944, TC389661 |
| [Cyclic-nucleotide-mediated signaling](http://amigo.geneontology.org/cgi-bin/amigo/go.cgi?action=query&view=query&query=GO:0019935&search_constraint=terms) | TC410352, DR739471, TC434820, TC443387, TC379711, TC392272, CV775873, TC386313, TC398052, CA710880, TC425841, TC402440, TC387579, TC394459, TC371242, TC384738, TC423252, TC394307, TC397660, CK211707, TC375146, TC386963, TC389363, TC388914, TC388410, TC389589, TC446235, TC392778, TC397312, TC420735, TC395453, TC386422, TC386961, TC388688, TC390569, TC416685, TC403872, TC399919, TC407572, TC389090, TC372175, TC389375, TC388822, TC396365, TC406708, TC377496, TC419747, TC382655, TC429771, CK200433, TC423576, BQ294582, TC406038, TC393970, TC416658, TC395351, TC401915, TC393561, TC393100, TC391671, TC393554, TC407614, TC382928, DR739994, TC384454, CA729339, TC386237, TC417363, TC393904, CK217367, TC379853, TC377410, TC381218, TC389816, TC395303, TC400755, TC405440, TC458987, CA613620, TC401210, DR737360, TC390135, TC397729, TC377225, TC434831, TC396895, TC426326, TC381462, TC393106, TC401758, BQ607161, TC392263, TC396460, TC428944, TC389661 |
| [Detection of temperature stimulus](http://amigo.geneontology.org/cgi-bin/amigo/go.cgi?action=query&view=query&query=GO:0016048&search_constraint=terms) | TC410352, TC401915, DR739471, TC393561, TC434820, TC443387, TC379711, TC391671, TC393554, TC407614, TC392272, TC459656, DR739994, TC384454, CA729339, TC402440, CK217367, TC423252, TC394307, TC397660, TC375146, TC389363, TC379853, TC388914, TC389589, TC389816, TC446235, TC405440, TC392778, TC420735, TC386422, TC395453, DR737360, TC388688, TC416685, TC390569, TC403872, TC389090, TC434831, TC396895, TC389375, TC426326, TC388822, TC377496, TC406708, TC382655, TC393106, TC429771, CK200433, BQ607161, TC423576, TC406807, TC406038, TC396460, TC416658, TC428944, TC395351, TC386646 |
| [Sensory perception of temperature stimulus](http://amigo.geneontology.org/cgi-bin/amigo/go.cgi?action=query&view=query&query=GO:0050951&search_constraint=terms) | TC410352, TC401915, DR739471, TC393561, TC434820, TC443387, TC379711, TC391671, TC393554, TC407614, TC392272, TC459656, DR739994, TC384454, CA729339, TC402440, CK217367, TC423252, TC394307, TC397660, TC375146, TC389363, TC379853, TC388914, TC389589, TC389816, TC446235, TC405440, TC392778, TC420735, TC386422, TC395453, DR737360, TC388688, TC416685, TC390569, TC403872, TC389090, TC434831, TC396895, TC389375, TC426326, TC388822, TC377496, TC406708, TC382655, TC393106, TC429771, CK200433, BQ607161, TC423576, TC406807, TC406038, TC396460, TC416658, TC428944, TC395351, TC386646 |
| [Detection of temperature stimulus involved in sensory perception](http://amigo.geneontology.org/cgi-bin/amigo/go.cgi?action=query&view=query&query=GO:0050961&search_constraint=terms) | TC410352, TC401915, DR739471, TC393561, TC434820, TC443387, TC379711, TC391671, TC393554, TC407614, TC392272, TC459656, DR739994, TC384454, CA729339, TC402440, CK217367, TC423252, TC394307, TC397660, TC375146, TC389363, TC379853, TC388914, TC389589, TC389816, TC446235, TC405440, TC392778, TC420735, TC386422, TC395453, DR737360, TC388688, TC416685, TC390569, TC403872, TC389090, TC434831, TC396895, TC389375, TC426326, TC388822, TC377496, TC406708, TC382655, TC393106, TC429771, CK200433, BQ607161, TC423576, TC406807, TC406038, TC396460, TC416658, TC428944, TC395351, TC386646 |
| [Detection of temperature stimulus involved in sensory perception of pain](http://amigo.geneontology.org/cgi-bin/amigo/go.cgi?action=query&view=query&query=GO:0050965&search_constraint=terms) | TC410352, TC401915, DR739471, TC393561, TC434820, TC443387, TC379711, TC391671, TC393554, TC407614, TC392272, TC459656, DR739994, TC384454, CA729339, TC402440, CK217367, TC423252, TC394307, TC397660, TC375146, TC389363, TC379853, TC388914, TC389589, TC389816, TC446235, TC405440, TC392778, TC420735, TC386422, TC395453, DR737360, TC388688, TC416685, TC390569, TC403872, TC389090, TC434831, TC396895, TC389375, TC426326, TC388822, TC377496, TC406708, TC382655, TC393106, TC429771, CK200433, BQ607161, TC423576, TC406807, TC406038, TC396460, TC416658, TC428944, TC395351, TC386646 |
| [Detection of mechanical stimulus involved in sensory perception of pain](http://amigo.geneontology.org/cgi-bin/amigo/go.cgi?action=query&view=query&query=GO:0050966&search_constraint=terms) | TC410352, TC401915, DR739471, TC393561, TC434820, TC443387, TC379711, TC391671, TC393554, TC407614, TC392272, TC459656, DR739994, TC384454, CA729339, TC402440, CK217367, TC423252, TC394307, TC397660, TC375146, TC389363, TC379853, TC388914, TC389589, TC389816, TC446235, TC405440, TC392778, TC420735, TC386422, TC395453, DR737360, TC388688, TC416685, TC390569, TC403872, TC389090, TC434831, TC396895, TC389375, TC426326, TC388822, TC377496, TC406708, TC382655, TC393106, TC429771, CK200433, BQ607161, TC423576, TC406807, TC406038, TC396460, TC416658, TC428944, TC395351, TC386646 |
| [Detection of chemical stimulus involved in sensory perception of pain](http://amigo.geneontology.org/cgi-bin/amigo/go.cgi?action=query&view=query&query=GO:0050968&search_constraint=terms) | TC410352, TC401915, DR739471, TC393561, TC434820, TC443387, TC379711, TC391671, TC393554, TC407614, TC392272, TC459656, DR739994, TC384454, CA729339, TC402440, CK217367, TC423252, TC394307, TC397660, TC375146, TC389363, TC379853, TC388914, TC389589, TC389816, TC446235, TC405440, TC392778, TC420735, TC386422, TC395453, DR737360, TC388688, TC416685, TC390569, TC403872, TC389090, TC434831, TC396895, TC389375, TC426326, TC388822, TC377496, TC406708, TC382655, TC393106, TC429771, CK200433, BQ607161, TC423576, TC406807, TC406038, TC396460, TC416658, TC428944, TC395351, TC386646 |
| [Second-messenger-mediated signaling](http://amigo.geneontology.org/cgi-bin/amigo/go.cgi?action=query&view=query&query=GO:0019932&search_constraint=terms) | TC410352, DR739471, TC434820, TC443387, TC379711, TC392272, CV775873, TC377061, TC386313, TC398052, CA710880, TC425841, TC402440, TC387579, TC394459, TC371242, TC384738, TC423252, TC394307, TC397660, CK211707, TC375146, TC386963, TC389363, TC388914, TC388410, TC389589, TC446235, TC392778, TC397312, TC420735, TC395453, TC386422, TC386961, TC388688, TC390569, TC416685, TC403872, TC399919, TC407572, TC389090, TC372175, TC389375, TC388822, TC404413, TC396365, TC406708, TC377496, TC419747, TC382655, TC429771, CK200433, TC423576, BQ294582, TC406038, TC393970, TC416658, TC395351, TC401915, TC393561, TC393100, TC391671, TC393554, TC407614, TC382928, DR739994, TC384454, CA729339, TC417363, TC386237, TC393904, CK217367, TC379853, TC377410, TC381218, TC389816, TC395303, TC400755, TC405440, TC458987, CA613620, TC400362, TC401210, DR737360, TC390135, TC397729, TC377225, TC434831, TC396895, TC426326, TC381462, TC393106, TC401758, BQ607161, TC392263, TC396460, TC428944, TC389661, TC393960 |
| [Response to acid](http://amigo.geneontology.org/cgi-bin/amigo/go.cgi?action=query&view=query&query=GO:0001101&search_constraint=terms) | TC410352, TC401915, DR739471, TC393561, TC434820, TC443387, TC379711, TC391671, TC393554, TC407614, TC392272, TC459656, DR739994, TC384454, CA729339, TC402440, CK217367, TC423252, TC394307, TC397660, TC375146, TC389363, TC379853, TC388914, TC389589, TC389816, TC446235, TC405440, TC392778, TC420735, TC386422, TC395453, DR737360, TC388688, TC416685, TC390569, TC403872, TC389090, TC434831, TC396895, TC389375, TC426326, TC388822, TC377496, TC406708, TC382655, TC393106, TC429771, CK200433, BQ607161, TC423576, TC406807, TC406038, TC396460, TC416658, TC428944, TC395351, TC386646 |
| [Cellular component organization](http://amigo.geneontology.org/cgi-bin/amigo/go.cgi?action=query&view=query&query=GO:0016043&search_constraint=terms) | TC461921, TC410352, TC386040, TC390393, TC416529, TC397258, TC389190, CV775873, TC396230, TC375864, TC460760, TC427210, TC416069, BQ838511, TC398052, CA710880, TC400388, TC421871, TC433162, TC394459, TC371242, TC416154, TC384738, TC423252, TC397660, TC389363, TC379171, TC388410, CK203550, TC416493, TC458562, TC421914, TC406264, TC387410, TC380063, TC398538, TC410063, TC378054, TC448471, TC395453, TC410194, TC392875, TC409843, TC435546, TC384735, TC390569, TC405540, TC407076, TC419057, TC403872, TC425847, CJ727624, TC398714, BQ294582, CA614761, CJ550278, TC457126, TC403580, TC413427, TC411128, TC401915, TC397444, TC398304, TC425878, TC393100, TC433957, TC412569, TC417012, CA632212, DR739994, TC459193, TC394916, TC394206, TC394820, TC406236, TC418073, TC378274, TC391130, TC417308, CK217367, TC409599, TC445767, TC446038, TC413460, TC379853, TC370315, BQ609416, CK214702, TC449504, TC395723, TC405440, CK213497, CJ792862, TC385233, TC389718, TC404052, TC398514, TC377225, TC381943, TC412520, TC432001, TC413392, BQ607161, TC406807, TC382045, TC402308, TC434820, TC441343, TC379711, TC400330, TC400260, TC378790, CA595837, TC379903, TC423265, TC392272, TC377061, TC401244, CA730421, TC446465, TC386313, CV759879, TC408907, CA606693, TC391438, CK211707, TC386963, TC412732, TC405695, CA700201, TC392778, TC396751, TC397312, TC370350, TC405030, CK211589, TC388403, TC376527, CA615187, TC416695, TC388718, TC419584, TC383701, TC406469, TC385780, TC405784, TC399919, TC407572, TC372175, TC410690, TC406708, TC419747, TC410074, TC402186, TC405356, CV763657, TC423354, TC402603, TC441241, TC433557, TC413571, TC413043, CA729339, CK214224, TC408299, BQ239045, CA709177, TC376263, TC416169, TC425690, TC381218, TC408229, TC422425, TC408312, CA613620, TC398805, TC400362, CV781430, TC388520, TC411191, TC399342, TC434831, TC426326, TC381462, CD882425, TC377749, TC373914, TC392263, TC392303, TC394796, TC445166, CA611770, TC435533, TC374240, CA720842, TC425841, CF554444, TC424204, TC387579, TC406870, TC388914, TC393814, TC376220, TC387621, TC398633, TC394716, TC446235, TC378271, TC380943, TC393830, TC380433, TC403885, TC420735, TC404371, TC384219, TC386961, TC416685, TC387344, TC372664, TC389090, TC412483, TC389375, TC404413, TC382655, CK200433, TC406038, TC393970, TC395351, TC418032, CK210754, TC369928, TC410147, TC384454, TC411287, TC386237, TC409343, TC393904, TC390994, TC391143, TC381463, TC403157, TC386414, TC395303, TC400755, TC440636, TC405511, TC403986, TC405615, TC376420, TC458987, TC377190, TC404843, TC431879, CD876572, TC385365, TC401210, DR737360, TC379357, TC390135, TC387135, TC386279, TC397885, TC411684, TC411480, TC409650, TC380662, TC393960, TC389162, DR739471, TC402534, TC443387, TC415083, TC386535, TC390630, GH729256, TC406584, TC386519, CA605200, TC402440, TC417992, TC391411, TC394307, TC409208, TC388665, TC375146, TC389589, TC381988, TC452762, TC386344, TC373637, TC381963, TC383176, CD878039, TC374879, TC386422, TC388688, TC418845, TC388547, TC414606, TC388822, TC377496, TC388976, TC396365, TC429771, TC410078, TC423576, TC386639, TC416658, TC393561, TC393948, TC393692, TC391671, TC393554, TC407614, TC459656, TC382928, TC438243, TC379436, TC417363, TC403573, TC413700, TC403929, TC449043, TC380882, TC423880, TC379635, TC440321, TC433589, TC377410, TC418091, TC389816, TC372530, TC392323, TC413066, TC391641, TC461622, CK211469, CB307332, TC404842, TC381817, TC403588, TC385659, TC397729, TC403803, TC396895, TC382342, TC393106, TC401758, TC432504, TC397676, TC396460, TC428944, TC417067, TC389661 |
| [Signaling](http://amigo.geneontology.org/cgi-bin/amigo/go.cgi?action=query&view=query&query=GO:0023052&search_constraint=terms) | TC410352, TC386040, TC407978, TC394796, TC397258, CV775873, TC435533, TC374240, TC398052, CA710880, TC425841, TC398862, TC433162, TC387579, TC394459, TC371242, TC384738, TC423252, TC397660, TC389363, TC388914, TC388410, TC458562, TC394716, TC398633, TC421914, TC446235, TC387410, TC380063, TC378271, TC393830, TC380943, TC418414, TC380433, TC378054, TC420735, TC404371, TC395453, TC386961, TC384219, TC392329, TC398606, TC384735, TC390569, TC416685, TC387344, TC407076, TC419057, TC403872, TC389090, TC389375, TC404413, TC382655, CK200433, TC398714, BQ294582, TC370114, TC406038, CJ550278, TC393970, TC395351, TC390904, TC403580, TC401915, TC459245, TC398304, TC393100, TC433957, CK210754, CA632212, DR739994, TC384454, TC411287, TC380329, TC386237, TC394916, TC393904, TC390994, TC378274, TC391143, CK217367, TC391128, TC409599, TC379853, TC370315, TC395303, TC386414, TC400755, TC405511, TC395723, TC440636, TC405440, TC403968, TC376420, TC458987, TC423110, TC379536, TC389718, TC401210, TC390135, TC398514, DR737360, TC379357, TC377225, TC386279, TC409650, TC381943, TC380662, BQ607161, TC393960, TC382045, CA646741, TC382830, DR739471, TC416906, TC402308, TC434820, TC443387, TC441343, CK212850, TC379711, TC378790, TC392272, TC377061, TC417077, TC417388, TC386519, TC386313, CV759879, TC413339, TC408907, TC402440, CA606693, TC420579, TC391438, TC394307, CK211707, TC375146, TC386963, TC398592, TC389589, TC421345, TC386344, TC392778, TC396751, TC397312, TC405030, TC381963, CK211589, TC388403, TC383176, TC383909, TC449463, TC435595, CA615187, TC386422, TC388688, TC405041, TC388547, TC385780, TC405784, TC412150, TC399919, TC407572, TC372175, TC388822, TC396365, TC377496, TC388976, TC406708, TC419747, TC429771, TC423576, TC386639, TC402186, TC416658, TC381957, TC386646, TC393561, TC393692, TC391671, TC393554, TC407614, TC382928, TC402603, TC423354, TC438243, TC441241, TC413571, TC379436, CA729339, TC417363, CK214224, TC411941, TC403929, BE585841, TC425690, TC373259, TC433589, TC377410, TC381218, TC389816, TC422425, TC372530, CA613620, CK211469, TC400362, TC397729, TC411191, TC434831, TC396895, TC432185, TC426326, TC381462, TC382342, TC393106, TC377749, TC401758, TC373914, TC432504, TC396460, TC397676, TC392263, TC428944, TC389661 |
| [Detection of mechanical stimulus involved in sensory perception](http://amigo.geneontology.org/cgi-bin/amigo/go.cgi?action=query&view=query&query=GO:0050974&search_constraint=terms) | TC410352, TC401915, DR739471, TC393561, TC434820, TC443387, TC379711, TC391671, TC393554, TC407614, TC392272, TC459656, DR739994, TC384454, CA729339, TC402440, CK217367, TC423252, TC394307, TC397660, TC375146, TC389363, TC379853, TC379171, TC388914, TC389589, TC389816, TC446235, TC405440, TC392778, TC420735, TC386422, TC395453, DR737360, TC388688, TC416685, TC390569, TC403872, TC389090, TC434831, TC396895, TC389375, TC426326, TC388822, TC377496, TC406708, TC382655, TC393106, TC429771, CK200433, BQ607161, TC423576, TC406807, TC406038, TC396460, TC416658, TC428944, TC395351, TC386646 |
| [Detection of mechanical stimulus](http://amigo.geneontology.org/cgi-bin/amigo/go.cgi?action=query&view=query&query=GO:0050982&search_constraint=terms) | TC410352, TC401915, DR739471, TC393561, TC434820, TC443387, TC379711, TC391671, TC393554, TC407614, TC392272, TC459656, DR739994, TC384454, CA729339, TC402440, CK217367, TC423252, TC394307, TC397660, TC375146, TC389363, TC379853, TC379171, TC388914, TC389589, TC389816, TC446235, TC405440, TC392778, TC420735, TC386422, TC395453, DR737360, TC388688, TC416685, TC390569, TC403872, TC389090, TC434831, TC396895, TC389375, TC426326, TC388822, TC377496, TC406708, TC382655, TC393106, TC429771, CK200433, BQ607161, TC423576, TC406807, TC406038, TC396460, TC416658, TC428944, TC395351, TC386646 |
| [Regulation of nucleobase, nucleoside, nucleotide and nucleic acid metabolic process](http://amigo.geneontology.org/cgi-bin/amigo/go.cgi?action=query&view=query&query=GO:0019219&search_constraint=terms) | TC392303, TC410352, TC386040, TC390393, TC397258, TC389190, CV775873, TC372677, TC435533, TC374240, TC398052, CA710880, TC425841, TC398862, TC433162, TC387579, TC394459, TC371242, TC384738, TC423252, TC397660, TC393814, TC389363, TC388914, TC388410, TC458562, TC394716, TC398633, TC446235, TC404926, TC380063, TC402545, TC393830, TC380943, TC418414, TC380433, TC378054, TC420735, TC404371, TC395453, TC386961, TC384219, TC373615, TC392329, TC390569, CA682223, TC416685, TC407076, TC419057, TC403872, TC389090, TC389375, TC404413, TC382655, CK200433, TC398714, TC373994, TC370114, BQ294582, TC406038, CJ550278, TC408192, TC395351, TC401915, TC393100, CK210754, CA632212, DR739994, TC384454, TC411287, TC380329, TC386237, TC394916, TC393904, TC390994, TC398731, TC378274, TC391143, CK217367, TC409599, TC379853, TC395303, TC386414, TC400755, TC395723, TC440636, TC406193, TC405440, TC403968, TC405615, TC458987, TC377190, TC422348, TC385233, TC389718, TC401210, TC390135, DR737360, TC379357, TC377225, TC386279, TC381943, BQ607161, TC403977, TC406807, TC393960, TC426358, TC382045, DR739471, TC416906, TC402308, TC434820, TC443387, TC379711, TC379903, TC392272, TC377061, TC417077, TC407340, TC417388, TC386519, TC386313, CV759879, CK208222, TC408907, TC402440, TC420579, TC391438, TC394307, CK211707, TC375146, TC386963, TC389589, TC452762, TC386344, TC392778, TC397312, TC405030, TC381963, CK211589, TC388403, TC383176, TC386422, TC388688, TC419584, TC385780, TC399919, TC407572, TC372175, TC388822, TC396365, TC377496, TC388976, TC406708, TC419747, TC429771, TC409459, TC423576, TC386639, TC402186, TC416658, TC374230, TC393561, TC393692, TC391671, TC393554, TC407614, TC459656, TC382928, TC402603, TC441241, TC413571, TC379436, CA729339, TC417363, TC460615, CK214224, TC425690, TC433589, TC377410, TC381218, TC389816, CA613620, CK211469, TC400362, TC397729, TC395872, TC434831, TC396895, TC426326, TC381462, TC418850, TC382342, TC393106, TC401758, TC373914, TC432504, TC392263, TC397676, TC396460, TC428944, TC389661 |
| [System process](http://amigo.geneontology.org/cgi-bin/amigo/go.cgi?action=query&view=query&query=GO:0003008&search_constraint=terms) | TC410352, CV775873, TC374240, TC398052, TC425841, TC398862, TC387579, TC394459, TC384738, TC423252, TC397660, TC389363, TC388914, TC379171, TC388410, TC458562, TC421914, TC446235, TC378271, TC418414, CJ944525, TC420735, TC395453, TC386961, TC390569, TC416685, TC387344, TC407076, TC403872, TC389090, TC389375, TC382655, CK200433, TC398714, TC370114, TC406038, CJ550278, TC393970, TC395351, TC401915, TC398304, TC433957, TC393100, CK210754, DR739994, TC384454, TC386237, TC394916, TC393904, TC390994, TC378274, CK217367, TC379853, TC395303, TC400755, TC395723, TC405440, TC458987, TC401210, DR737360, TC398514, TC390135, TC377225, TC409650, TC380662, BQ607161, TC406807, TC382045, TC404606, DR739471, TC416906, TC434820, TC443387, TC379711, TC392272, TC377061, TC417388, TC386313, TC402440, CA606693, TC394307, CK211707, TC375146, TC386963, CK199175, TC389589, TC392778, TC397312, TC396751, CK211589, TC383909, CA615187, TC449463, TC386422, TC388688, TC388547, TC405784, TC372175, TC407572, TC399919, TC388822, TC377496, TC406708, TC396365, TC419747, TC429771, TC409459, TC423576, TC416658, TC386646, TC393561, TC391671, TC393554, TC407614, TC459656, TC382928, TC441241, TC438243, TC449724, TC413571, CA729339, TC417363, TC403929, TC387710, TC425690, TC377410, TC381218, TC389816, TC422425, TC393820, TC397729, TC411191, TC432185, TC396895, TC434831, TC426326, TC381462, TC393106, TC401758, TC392263, TC396460, TC389661, TC428944 |
| [Cell communication](http://amigo.geneontology.org/cgi-bin/amigo/go.cgi?action=query&view=query&query=GO:0007154&search_constraint=terms) | TC410352, CV775873, TC374240, TC398052, CA710880, TC425841, TC387579, TC394459, TC384738, TC423252, TC397660, TC389363, TC388914, TC388410, TC458562, TC421914, TC446235, TC378271, TC433844, TC420735, TC395453, TC386961, TC384735, TC390569, TC416685, TC387344, TC407076, TC403872, TC389090, TC389375, TC382655, CK200433, TC398714, BQ294582, TC406038, CJ550278, TC393970, TC395351, TC401915, TC398304, TC433957, TC393100, CK210754, DR739994, TC384454, TC386237, TC394916, TC393904, TC390994, TC378274, CK217367, TC379853, TC395303, TC400755, TC395723, TC405440, TC376420, TC458987, TC398730, TC401210, DR737360, TC398514, TC390135, TC387135, TC377225, TC409650, TC380662, BQ607161, TC406807, TC382045, DR739471, TC434820, TC441343, TC443387, TC379711, TC392272, TC386313, TC402440, CA606693, TC394307, TC375146, CK211707, TC386963, TC389589, TC392778, TC370350, TC397312, TC396751, CK211589, CA615187, TC386422, TC388688, TC388547, TC405784, TC372175, TC407572, TC399919, TC377496, TC406708, TC396365, TC388822, TC419747, TC429771, TC423576, TC416658, TC393561, TC391671, TC393554, TC407614, TC459656, TC382928, TC441241, TC413571, CA729339, TC417363, TC403929, TC377410, TC381218, TC389816, TC422425, CA613620, TC397729, TC411191, TC396895, TC434831, TC426326, TC381462, TC393106, TC401758, TC392263, TC396460, TC389661, TC428944 |
| [Regulation of cell proliferation](http://amigo.geneontology.org/cgi-bin/amigo/go.cgi?action=query&view=query&query=GO:0042127&search_constraint=terms) | TC392303, TC410352, DR739471, TC434820, TC443387, TC379711, TC392272, CV775873, TC407340, TC386313, TC398052, CA710880, TC402440, TC387579, TC394459, TC371242, TC384738, TC423252, TC394307, TC397660, CK211707, TC375146, TC386963, TC389363, TC388914, TC388410, TC389589, TC458562, TC446235, TC392778, TC396751, TC420735, CA615187, TC395453, TC386422, TC386961, TC388688, TC390569, TC388547, TC416685, TC387344, TC407076, TC403872, TC405784, TC399919, TC407572, TC389090, TC372175, TC389375, TC388822, TC396365, TC406708, TC377496, TC419747, TC382655, TC429771, CK200433, TC423576, TC398714, BQ294582, TC406038, TC416658, TC395351, TC374230, TC401915, TC393561, TC398304, TC433957, TC393100, CK210754, TC391671, TC393554, TC407614, TC382928, TC438243, TC441241, DR739994, TC384454, CA729339, TC386237, TC394916, TC393904, TC403929, CK217367, TC379853, TC377410, TC381218, TC395303, TC389816, TC422425, TC400755, TC405440, TC376420, TC377190, CA613620, TC388520, TC401210, DR737360, TC390135, TC397729, TC411191, TC434831, TC396895, TC426326, TC381462, TC393106, TC380662, TC401758, BQ607161, TC396460, TC392263, TC428944, TC389661, TC382045 |
| [Regulation of transcription, DNA-dependent](http://amigo.geneontology.org/cgi-bin/amigo/go.cgi?action=query&view=query&query=GO:0006355&search_constraint=terms) | TC392303, TC410352, TC386040, TC390393, TC397258, TC389190, TC435533, TC374240, CA710880, TC398862, TC433162, TC387579, TC423252, TC397660, TC393814, TC389363, TC388914, TC458562, TC394716, TC398633, TC446235, TC380063, TC402545, TC393830, TC380943, TC418414, TC380433, TC378054, TC420735, TC404371, TC395453, TC386961, TC384219, TC373615, TC392329, TC390569, CA682223, TC416685, TC407076, TC419057, TC403872, TC389090, TC389375, TC404413, TC382655, CK200433, TC398714, TC373994, TC370114, BQ294582, TC406038, CJ550278, TC395351, TC401915, CK210754, CA632212, DR739994, TC384454, TC411287, TC380329, TC394916, TC390994, TC398731, TC378274, TC391143, CK217367, TC409599, TC379853, TC386414, TC400755, TC440636, TC395723, TC406193, TC405440, TC403968, TC405615, TC422348, TC385233, TC389718, TC401210, DR737360, TC379357, TC377225, TC386279, TC381943, BQ607161, TC406807, TC393960, TC426358, DR739471, TC416906, TC402308, TC434820, TC443387, TC379711, TC379903, TC392272, TC417077, TC417388, TC386519, TC386313, CV759879, TC408907, TC402440, TC420579, TC391438, TC394307, TC375146, TC386963, TC389589, TC452762, TC386344, TC392778, TC405030, TC397312, TC381963, CK211589, TC388403, TC383176, TC386422, TC388688, TC419584, TC385780, TC388822, TC377496, TC388976, TC406708, TC429771, TC409459, TC423576, TC386639, TC402186, TC416658, TC393561, TC393692, TC391671, TC393554, TC407614, TC459656, TC402603, TC441241, TC413571, TC379436, CA729339, TC460615, CK214224, TC433589, TC377410, TC381218, TC389816, CA613620, CK211469, TC400362, TC397729, TC395872, TC434831, TC396895, TC426326, TC418850, TC382342, TC381462, TC393106, TC401758, TC373914, TC432504, TC392263, TC397676, TC396460, TC428944, TC389661 |
| [Regulation of nitrogen compound metabolic process](http://amigo.geneontology.org/cgi-bin/amigo/go.cgi?action=query&view=query&query=GO:0051171&search_constraint=terms) | TC392303, TC410352, TC386040, TC390393, TC397258, TC389190, CV775873, TC372677, TC435533, TC374240, TC398052, CA710880, TC425841, TC398862, TC433162, TC387579, TC394459, TC371242, TC384738, TC423252, TC397660, TC393814, TC389363, TC388914, TC388410, TC458562, TC394716, TC398633, TC446235, TC404926, TC380063, TC402545, TC393830, TC380943, TC418414, TC380433, TC378054, TC420735, TC404371, TC395453, TC386961, TC384219, TC373615, TC392329, TC390569, CA682223, TC416685, TC407076, TC419057, TC403872, TC389090, TC389375, TC404413, TC382655, CK200433, TC398714, TC373994, TC370114, BQ294582, TC406038, CJ550278, TC408192, TC395351, TC401915, TC393100, CK210754, CA632212, DR739994, TC384454, TC411287, TC380329, TC386237, TC394916, TC393904, TC390994, TC398731, TC378274, TC391143, CK217367, TC409599, TC379853, TC395303, TC386414, TC400755, TC395723, TC440636, TC406193, TC405440, TC403968, TC405615, TC458987, TC377190, TC422348, TC385233, TC389718, TC401210, TC390135, DR737360, TC379357, TC377225, TC386279, TC381943, BQ607161, TC403977, TC406807, TC393960, TC426358, TC382045, DR739471, TC416906, TC402308, TC434820, TC443387, TC379711, TC379903, TC392272, TC377061, TC417077, TC407340, TC417388, TC386519, TC386313, CV759879, CK208222, TC408907, TC402440, TC420579, TC391438, TC394307, CK211707, TC375146, TC386963, TC389589, TC452762, TC386344, TC392778, TC397312, TC405030, TC381963, CK211589, TC388403, TC383176, TC386422, TC388688, TC419584, TC385780, TC399919, TC407572, TC372175, TC388822, TC396365, TC377496, TC388976, TC406708, TC419747, TC429771, TC409459, TC423576, TC386639, TC402186, TC416658, TC374230, TC393561, TC393692, TC391671, TC393554, TC407614, TC459656, TC382928, TC402603, TC441241, TC413571, TC379436, CA729339, TC417363, TC460615, CK214224, TC425690, TC433589, TC377410, TC381218, TC389816, CA613620, CK211469, TC400362, TC397729, TC395872, TC434831, TC396895, TC426326, TC381462, TC418850, TC382342, TC393106, TC401758, TC373914, TC432504, TC392263, TC397676, TC396460, TC428944, TC389661 |
| [Nucleobase, nucleoside, nucleotide and nucleic acid metabolic process](http://amigo.geneontology.org/cgi-bin/amigo/go.cgi?action=query&view=query&query=GO:0006139&search_constraint=terms) | TC392303, TC410352, TC386040, TC373613, TC390393, TC372330, TC397258, TC425957, TC375864, TC377766, TC445166, TC435533, TC374240, TC375313, CA710880, TC457112, TC398862, TC433162, TC387579, TC371242, TC423252, TC397660, TC393814, TC389363, TC388914, TC376220, TC377438, TC458562, TC411471, TC394716, TC398633, TC446235, TC387410, TC378878, TC380063, TC393830, TC397176, TC380943, TC418414, CJ944525, TC380433, TC378054, TC420735, TC404371, TC395453, TC386961, TC384219, TC384735, TC390569, TC405540, TC416685, TC372664, TC419057, TC403872, TC389090, TC389375, TC382655, CK200433, TC373791, TC398714, TC373994, BQ294582, TC370114, TC406038, TC395351, TC403580, TC401915, GH732878, CK210754, CA632212, DR739994, TC384454, TC376490, TC411287, TC394916, EB512907, TC390994, TC378274, TC391143, CK217367, TC391962, TC423804, TC379853, TC370315, TC386414, TC400755, TC372701, TC405511, TC395723, TC440636, TC405440, TC377190, TC370885, TC443814, TC404843, TC385233, TC389718, TC401210, DR737360, TC379357, TC387135, TC377225, TC424376, TC386279, TC381943, BQ607161, TC406807, TC393960, TC404606, TC369628, TC389162, DR739471, TC416906, TC402308, TC434820, TC443387, TC441343, TC379711, TC388566, TC379903, TC392272, TC390285, TC377061, TC417077, TC417388, TC386519, TC386313, CV759879, TC408907, TC402440, TC391438, TC417341, TC394307, TC409208, TC375146, TC386963, TC454407, TC389589, TC381988, TC452762, TC386344, TC394661, TC392778, TC397312, TC405030, TC381963, TC388403, TC383176, TC371455, TC386422, TC374164, TC388688, TC385780, TC388822, TC377496, TC368549, TC388976, TC406708, TC429771, TC410078, TC423576, TC409459, TC386639, TC397500, TC402186, TC416658, TC396650, TC393561, TC393692, TC391671, TC393554, TC407614, TC458205, TC459656, TC402603, TC423354, TC441241, TC449724, TC413571, TC379436, CA729339, CK214224, TC387710, TC394118, TC380882, TC423880, TC433589, TC377410, TC381218, TC418091, TC399352, TC389816, CA613620, TC393820, CK211469, TC381817, TC369092, TC403588, TC397729, TC434831, TC396895, TC432185, TC426326, TC381462, TC382342, TC393106, TC401758, TC373914, TC432504, TC396460, TC397676, TC392263, TC428944, TC389661 |
| [Response to mechanical stimulus](http://amigo.geneontology.org/cgi-bin/amigo/go.cgi?action=query&view=query&query=GO:0009612&search_constraint=terms) | TC410352, TC401915, DR739471, TC393561, TC434820, TC443387, TC379711, TC391671, TC393554, TC407614, TC392272, TC459656, DR739994, TC384454, CA729339, TC402440, CK217367, TC423252, TC394307, TC397660, TC375146, TC389363, TC379853, TC379171, TC388914, TC389589, TC389816, TC446235, TC405440, TC392778, TC420735, TC386422, TC395453, DR737360, TC388688, TC416685, TC390569, TC403872, TC389090, TC434831, TC396895, TC389375, TC426326, TC388822, TC377496, TC406708, TC382655, TC393106, TC429771, CK200433, BQ607161, TC423576, TC406807, TC406038, TC396460, TC416658, TC428944, TC395351, TC386646 |
| [Regulation of RNA metabolic process](http://amigo.geneontology.org/cgi-bin/amigo/go.cgi?action=query&view=query&query=GO:0051252&search_constraint=terms) | TC392303, TC410352, TC386040, TC390393, TC397258, TC389190, TC435533, TC374240, CA710880, TC398862, TC433162, TC387579, TC423252, TC397660, TC393814, TC389363, TC388914, TC458562, TC394716, TC398633, TC446235, TC380063, TC402545, TC393830, TC380943, TC418414, TC380433, TC378054, TC420735, TC404371, TC395453, TC386961, TC384219, TC373615, TC392329, TC390569, CA682223, TC416685, TC407076, TC419057, TC403872, TC389090, TC389375, TC404413, TC382655, CK200433, TC398714, TC373994, TC370114, BQ294582, TC406038, CJ550278, TC395351, TC401915, CK210754, CA632212, DR739994, TC384454, TC411287, TC380329, TC394916, TC390994, TC398731, TC378274, TC391143, CK217367, TC409599, TC379853, TC386414, TC400755, TC440636, TC395723, TC406193, TC405440, TC403968, TC405615, TC422348, TC385233, TC389718, TC401210, DR737360, TC379357, TC377225, TC386279, TC381943, BQ607161, TC406807, TC393960, TC426358, DR739471, TC416906, TC402308, TC434820, TC443387, TC379711, TC379903, TC392272, TC417077, TC417388, TC386519, TC386313, CV759879, TC408907, TC402440, TC420579, TC391438, TC394307, TC375146, TC386963, TC389589, TC452762, TC386344, TC392778, TC405030, TC397312, TC381963, CK211589, TC388403, TC383176, TC386422, TC388688, TC419584, TC385780, TC388822, TC377496, TC388976, TC406708, TC429771, TC409459, TC423576, TC386639, TC402186, TC416658, TC393561, TC393692, TC391671, TC393554, TC407614, TC459656, TC402603, TC441241, TC413571, TC379436, CA729339, TC460615, CK214224, TC433589, TC377410, TC381218, TC389816, CA613620, CK211469, TC400362, TC397729, TC395872, TC434831, TC396895, TC426326, TC418850, TC382342, TC381462, TC393106, TC401758, TC373914, TC432504, TC392263, TC397676, TC396460, TC428944, TC389661 |
| [Cellular component organization or biogenesis](http://amigo.geneontology.org/cgi-bin/amigo/go.cgi?action=query&view=query&query=GO:0071840&search_constraint=terms) | TC461921, TC410352, TC386040, TC379942, TC407978, TC390393, TC416529, TC397258, TC389190, CV775873, TC396230, TC375864, TC460760, TC427210, TC416069, BQ838511, TC398052, CA710880, TC400388, TC421871, TC398862, TC433162, TC394459, TC371242, TC416154, TC384738, TC423252, TC397660, TC389363, TC379171, TC388410, CK203550, TC416493, TC458562, TC421914, TC406264, TC387410, TC380063, TC398538, TC418414, TC410063, TC378054, TC448471, TC395453, TC410194, TC392875, TC409843, TC435546, TC384735, TC390569, TC405540, TC407076, TC419057, TC403872, TC425847, TC391948, CJ727624, TC398714, BQ294582, CA614761, CJ550278, TC381619, TC457126, TC403580, TC413427, TC411128, TC401915, TC397444, TC398304, TC425878, TC393100, TC433957, TC412569, TC417012, CA632212, TC393523, DR739994, TC459193, TC394916, TC394206, TC394820, TC406236, TC418073, TC378274, TC391130, TC417308, CK217367, TC409599, TC445767, TC446038, TC423804, TC413460, TC379853, TC370315, BQ609416, CK214702, TC449504, TC395723, TC405440, CK213497, CJ792862, TC385233, TC389718, TC404052, TC398514, TC377225, CK201148, TC381943, TC412520, TC432001, TC413392, BQ607161, TC406807, TC382045, TC404606, TC416906, TC402308, TC434820, TC441343, TC379711, TC400330, TC400260, TC378790, CA595837, TC379903, TC377308, TC423265, TC392272, TC377061, TC401244, CA730421, TC417388, TC446465, TC386313, CV759879, TC408907, CA606693, TC391438, CK211707, TC386963, TC412732, TC405695, CA700201, TC392778, TC396751, TC397312, TC405030, TC370350, CK211589, TC388403, TC376527, CA615187, TC416695, TC388718, TC419584, TC383701, TC406469, TC385780, TC405784, TC399919, TC407572, TC372175, TC369199, TC410690, TC406708, TC419747, TC410074, TC402186, TC405356, CV763657, TC423354, TC402603, TC441241, TC433557, TC413571, TC413043, CA729339, CK214224, TC408299, BQ239045, CA709177, TC376263, TC384010, TC416169, TC425690, TC381218, TC399352, TC408229, TC422425, TC408312, CA613620, TC398805, TC400362, CV781430, TC388520, TC411191, TC400108, TC399342, TC434831, TC426326, TC381462, CD882425, TC377749, TC373914, TC392263, TC392303, TC394796, TC445166, CA611770, TC435533, TC374240, CA720842, TC425841, CF554444, TC424204, TC387579, TC406870, TC388914, TC393814, TC376220, TC387621, TC398633, TC394716, TC446235, TC402545, TC378271, TC380943, TC393830, TC380433, TC403885, TC420735, TC435281, TC404371, TC386961, TC384219, TC416685, TC387344, TC372664, TC389090, TC412483, TC389375, TC404413, TC382655, CK200433, TC406038, TC393970, TC395351, TC418032, CK210754, TC369928, TC410147, TC376490, TC384454, TC411287, TC386237, TC409343, TC393904, TC390994, TC391143, TC381463, TC403157, TC410066, TC395303, TC386414, TC400755, TC403986, TC440636, TC405511, TC405615, TC376420, TC458987, TC377190, TC404843, TC431879, TC391613, CD876572, TC385365, TC382737, TC401210, TC369736, DR737360, TC379357, TC390135, TC387135, TC386279, TC397885, TC411684, TC411480, TC409650, TC380662, TC393960, TC395298, TC389162, DR739471, TC402534, TC443387, TC415083, TC390630, TC386535, GH729256, TC406584, TC386519, CA605200, TC402440, TC417992, TC391411, TC417341, TC394307, TC409208, TC375146, TC388665, TC389589, TC381988, TC452762, TC405475, TC386344, TC373637, TC381963, TC383176, TC370044, CD878039, TC374879, TC386422, TC403328, TC388688, TC418845, TC388547, TC414606, TC388822, TC396365, TC377496, TC388976, TC429771, TC410078, TC423576, TC386639, TC416658, TC393561, TC393948, TC393692, TC391671, TC393554, TC407614, TC373787, TC459656, TC382928, TC438243, TC379436, TC417363, TC403573, TC413700, TC403929, TC394118, TC449043, TC380882, TC423880, TC379635, TC440321, TC373259, TC433589, TC377410, TC418091, TC389816, TC372530, TC392323, TC413066, TC391641, TC461622, CK211469, CB307332, TC404842, TC381817, TC403588, TC385659, TC397729, DR740372, TC403803, TC396895, TC382342, TC393106, TC401758, TC432504, TC397676, TC396460, TC428944, TC389661, TC417067 |
| [Nitrogen compound metabolic process](http://amigo.geneontology.org/cgi-bin/amigo/go.cgi?action=query&view=query&query=GO:0006807&search_constraint=terms) | TC392303, TC410352, TC386040, TC373613, TC390393, TC372330, TC397258, TC425957, TC375864, TC377766, TC445166, TC435533, TC374240, TC417106, TC375313, CA710880, TC457112, TC398862, TC433162, TC376351, TC387579, TC371242, TC413854, TC423252, TC397660, TC393814, TC389363, TC388914, TC434442, BJ279521, TC376220, TC377438, TC458562, TC380590, TC411471, TC394716, TC398633, TC446235, TC387410, TC378878, TC380063, TC433844, TC393830, TC397176, TC380943, TC418414, CJ944525, TC380433, TC378054, TC420735, TC373702, TC404371, TC395453, TC386961, TC384219, TC384735, TC390569, TC405540, TC416685, TC372664, TC419057, TC403872, TC389090, TC389375, TC382655, CK200433, TC373791, TC398714, TC373994, BQ294582, TC370114, TC406038, TC395351, TC403580, TC401915, GH732878, CK210754, TC369928, TC385710, TC393523, CA632212, DR739994, TC384454, TC376490, TC411287, TC394916, EB512907, TC390994, TC378274, TC391143, CK217367, TC391962, TC423804, TC379853, TC421880, TC370315, TC386414, TC400755, TC372701, TC405511, TC395723, TC440636, TC405440, TC377190, TC370885, TC443814, TC404843, TC385233, TC389718, TC401210, TC379357, DR737360, TC387135, TC377225, TC424376, TC386279, CK201148, TC381943, BQ607161, TC406807, TC393960, TC372845, TC404606, TC369628, TC389162, TC409077, DR739471, TC416906, TC402308, TC434820, TC443387, TC441343, TC379711, TC388566, TC377308, TC379903, TC390630, TC392272, TC390285, TC377061, TC417077, TC417388, TC386519, TC386313, CV759879, TC402072, TC408907, TC402440, TC391438, TC417341, TC394307, TC409208, TC375146, TC386963, TC432369, TC454407, TC389589, TC381988, TC452762, TC384122, TC386344, TC392778, TC394661, TC397312, TC370350, TC405030, TC381963, TC388403, TC383176, TC371455, TC386422, TC374164, TC405041, TC388688, TC385780, TC369199, TC388822, TC406708, TC388976, TC368549, TC377496, TC429771, TC410078, TC423576, TC409459, TC386639, TC397500, TC402186, TC416658, TC402668, TC396650, TC393561, TC393948, TC393692, TC391671, TC393554, TC407614, TC373787, TC458205, TC423354, TC459656, TC402603, TC441241, TC449724, TC413571, TC379436, CA729339, CK214224, TC387710, TC394118, TC380882, TC423880, TC384010, TC433589, TC377410, TC381218, TC418091, TC399352, TC389816, TC451519, TC393820, CA613620, CK211469, CJ930688, TC403588, TC381817, TC369092, TC397729, DR740372, TC434831, TC396895, TC432185, TC426326, TC381462, TC382342, TC393106, TC401758, TC373914, TC432504, TC396460, TC397676, TC392263, TC428944, TC389661 |
| [Negative regulation of transcription from RNA polymerase II promoter](http://amigo.geneontology.org/cgi-bin/amigo/go.cgi?action=query&view=query&query=GO:0000122&search_constraint=terms) | TC392303, TC393692, TC390393, CK210754, TC379903, TC397258, CA632212, TC402603, TC441241, TC379436, TC411287, TC460615, TC435533, TC394916, TC386313, CV759879, CK214224, TC390994, TC408907, TC378274, TC387579, TC391438, TC386963, TC393814, TC433589, TC377410, TC381218, TC458562, TC400755, TC452762, CK211469, TC397312, TC400362, TC388403, TC404371, TC401210, TC386961, TC384219, TC379357, TC397729, TC377225, TC386279, TC419057, TC381943, TC404413, TC381462, TC382342, TC418850, TC401758, TC370114, TC398714, TC392263, TC402186, TC389661, TC393960 |
| [Detection of external stimulus](http://amigo.geneontology.org/cgi-bin/amigo/go.cgi?action=query&view=query&query=GO:0009581&search_constraint=terms) | TC410352, TC401915, DR739471, TC393561, TC434820, TC443387, TC379711, TC391671, TC393554, TC407614, TC392272, TC459656, DR739994, TC384454, CA729339, TC402440, CK217367, TC423252, TC394307, TC397660, TC375146, TC389363, TC379853, TC379171, TC388914, TC389589, TC389816, TC446235, TC405440, TC392778, TC420735, TC386422, TC395453, DR737360, TC388688, TC416685, TC390569, TC403872, TC389090, TC434831, TC396895, TC389375, TC426326, TC388822, TC377496, TC406708, TC382655, TC393106, TC429771, CK200433, BQ607161, TC423576, TC406807, TC406038, TC396460, TC416658, TC428944, TC395351, TC386646 |
| [Detection of abiotic stimulus](http://amigo.geneontology.org/cgi-bin/amigo/go.cgi?action=query&view=query&query=GO:0009582&search_constraint=terms) | TC410352, TC401915, DR739471, TC393561, TC434820, TC443387, TC379711, TC391671, TC393554, TC407614, TC392272, TC459656, DR739994, TC384454, CA729339, TC402440, CK217367, TC423252, TC394307, TC397660, TC375146, TC389363, TC379853, TC379171, TC388914, TC389589, TC389816, TC446235, TC405440, TC392778, TC420735, TC386422, TC395453, DR737360, TC388688, TC416685, TC390569, TC403872, TC389090, TC434831, TC396895, TC389375, TC426326, TC388822, TC377496, TC406708, TC382655, TC393106, TC429771, CK200433, BQ607161, TC423576, TC406807, TC406038, TC396460, TC416658, TC428944, TC395351, TC386646 |
| [Genetic transfer](http://amigo.geneontology.org/cgi-bin/amigo/go.cgi?action=query&view=query&query=GO:0009292&search_constraint=terms) | TC386040, TC402308, TC393692, TC390393, TC379903, TC397258, CA632212, TC402603, TC379436, TC411287, TC435533, TC386519, TC374240, CV759879, CK214224, TC408907, TC391143, TC433162, TC391438, TC393814, TC386414, TC398633, TC394716, TC440636, TC452762, TC380063, TC380943, TC393830, TC386344, CK211469, TC405030, TC378054, TC381963, TC388403, TC383176, TC404371, TC389718, TC384219, TC379357, TC386279, TC385780, TC419057, TC381943, TC382342, TC388976, TC373914, TC432504, TC386639, TC397676, TC402186 |
| [DNA mediated transformation](http://amigo.geneontology.org/cgi-bin/amigo/go.cgi?action=query&view=query&query=GO:0009294&search_constraint=terms) | TC386040, TC402308, TC393692, TC390393, TC379903, TC397258, CA632212, TC402603, TC379436, TC411287, TC435533, TC386519, TC374240, CV759879, CK214224, TC408907, TC391143, TC433162, TC391438, TC393814, TC386414, TC398633, TC394716, TC440636, TC452762, TC380063, TC380943, TC393830, TC386344, CK211469, TC405030, TC378054, TC381963, TC388403, TC383176, TC404371, TC389718, TC384219, TC379357, TC386279, TC385780, TC419057, TC381943, TC382342, TC388976, TC373914, TC432504, TC386639, TC397676, TC402186 |
| [Regulation of apoptosis](http://amigo.geneontology.org/cgi-bin/amigo/go.cgi?action=query&view=query&query=GO:0042981&search_constraint=terms) | TC410352, DR739471, TC434820, TC443387, TC441343, TC379711, TC392272, CV775873, TC386313, TC398052, CA710880, TC425841, TC402440, TC387579, TC394459, TC384738, TC423252, TC394307, TC397660, CK211707, TC375146, TC386963, TC389363, TC388914, TC388410, TC389589, TC446235, TC392778, TC397312, TC380433, CK211589, TC420735, TC395453, TC386422, TC386961, TC388688, TC384735, TC390569, TC416685, TC403872, TC399919, TC407572, TC389090, TC372175, TC389375, TC388822, TC396365, TC406708, TC377496, TC419747, TC382655, TC429771, CK200433, TC423576, TC409459, TC370114, BQ294582, TC406038, TC393970, TC416658, TC395351, TC401915, TC393561, TC393100, TC391671, TC393554, TC407614, TC382928, TC423354, DR739994, TC384454, TC413571, CA729339, TC417363, TC386237, TC393904, CK217367, TC394118, TC380882, TC423880, TC379853, TC377410, TC381218, TC395303, TC389816, TC400755, TC405511, TC405440, TC376420, TC458987, TC377190, CA613620, TC400056, TC381817, TC403588, TC401210, DR737360, TC390135, TC397729, TC377225, TC434831, TC391122, TC396895, TC426326, TC381462, TC393106, TC401758, BQ607161, TC396460, TC392263, TC428944, TC389661, TC382045 |
| [Regulation of cell death](http://amigo.geneontology.org/cgi-bin/amigo/go.cgi?action=query&view=query&query=GO:0010941&search_constraint=terms) | TC410352, DR739471, TC434820, TC443387, TC441343, TC379711, TC392272, CV775873, TC386313, TC398052, CA710880, TC425841, TC402440, TC387579, TC394459, TC384738, TC423252, TC394307, TC397660, CK211707, TC375146, TC386963, TC389363, TC388914, TC388410, TC389589, TC446235, TC392778, TC397312, TC380433, CK211589, TC420735, TC395453, TC386422, TC386961, TC388688, TC384735, TC390569, TC416685, TC403872, TC399919, TC407572, TC389090, TC372175, TC389375, TC388822, TC396365, TC406708, TC377496, TC419747, TC382655, TC429771, CK200433, TC423576, TC409459, TC370114, BQ294582, TC406038, TC393970, TC416658, TC395351, TC401915, TC393561, TC393100, TC391671, TC393554, TC407614, TC382928, TC423354, DR739994, TC384454, TC413571, CA729339, TC417363, TC386237, TC393904, CK217367, TC394118, TC380882, TC423880, TC379853, TC377410, TC381218, TC395303, TC389816, TC400755, TC405511, TC405440, TC376420, TC458987, TC377190, CA613620, TC400056, TC381817, TC403588, TC401210, DR737360, TC390135, TC397729, TC377225, TC434831, TC391122, TC396895, TC426326, TC381462, TC393106, TC401758, BQ607161, TC396460, TC392263, TC428944, TC389661, TC382045 |
| [Regulation of programmed cell death](http://amigo.geneontology.org/cgi-bin/amigo/go.cgi?action=query&view=query&query=GO:0043067&search_constraint=terms) | TC410352, DR739471, TC434820, TC443387, TC441343, TC379711, TC392272, CV775873, TC386313, TC398052, CA710880, TC425841, TC402440, TC387579, TC394459, TC384738, TC423252, TC394307, TC397660, CK211707, TC375146, TC386963, TC389363, TC388914, TC388410, TC389589, TC446235, TC392778, TC397312, TC380433, CK211589, TC420735, TC395453, TC386422, TC386961, TC388688, TC384735, TC390569, TC416685, TC403872, TC399919, TC407572, TC389090, TC372175, TC389375, TC388822, TC396365, TC406708, TC377496, TC419747, TC382655, TC429771, CK200433, TC423576, TC409459, TC370114, BQ294582, TC406038, TC393970, TC416658, TC395351, TC401915, TC393561, TC393100, TC391671, TC393554, TC407614, TC382928, TC423354, DR739994, TC384454, TC413571, CA729339, TC417363, TC386237, TC393904, CK217367, TC394118, TC380882, TC423880, TC379853, TC377410, TC381218, TC395303, TC389816, TC400755, TC405511, TC405440, TC376420, TC458987, TC377190, CA613620, TC400056, TC381817, TC403588, TC401210, DR737360, TC390135, TC397729, TC377225, TC434831, TC391122, TC396895, TC426326, TC381462, TC393106, TC401758, BQ607161, TC396460, TC392263, TC428944, TC389661, TC382045 |
| [Signal transduction](http://amigo.geneontology.org/cgi-bin/amigo/go.cgi?action=query&view=query&query=GO:0007165&search_constraint=terms) | TC410352, TC386040, TC407978, TC394796, TC397258, CV775873, TC435533, TC374240, TC398052, CA710880, TC425841, TC433162, TC387579, TC394459, TC371242, TC384738, TC423252, TC397660, TC389363, TC388914, TC388410, TC394716, TC398633, TC446235, TC387410, TC380063, TC393830, TC380943, TC380433, TC378054, TC420735, TC404371, TC395453, TC386961, TC384219, TC398606, TC384735, TC390569, TC416685, TC407076, TC419057, TC403872, TC389090, TC389375, TC404413, TC382655, CK200433, BQ294582, TC406038, CJ550278, TC393970, TC395351, TC403580, TC401915, TC393100, CA632212, DR739994, TC384454, TC411287, TC386237, TC393904, TC391143, CK217367, TC391128, TC409599, TC379853, TC370315, TC395303, TC386414, TC400755, TC440636, TC395723, TC405440, TC376420, TC458987, TC423110, TC379536, TC389718, TC401210, TC390135, DR737360, TC379357, TC377225, TC386279, TC381943, BQ607161, TC393960, TC382045, CA646741, DR739471, TC402308, TC434820, TC443387, TC441343, TC379711, TC378790, TC392272, TC377061, TC417077, TC386519, TC386313, CV759879, TC413339, TC408907, TC402440, TC391438, TC394307, CK211707, TC375146, TC386963, TC398592, TC389589, TC421345, TC386344, TC392778, TC397312, TC405030, TC381963, CK211589, TC388403, TC383176, TC386422, TC388688, TC405041, TC385780, TC399919, TC407572, TC372175, TC388822, TC377496, TC388976, TC406708, TC396365, TC419747, TC429771, TC423576, TC386639, TC402186, TC416658, TC381957, TC386646, TC393561, TC393692, TC391671, TC393554, TC407614, TC382928, TC402603, TC438243, TC413571, TC379436, CA729339, TC417363, CK214224, BE585841, TC425690, TC373259, TC433589, TC377410, TC381218, TC389816, TC372530, CA613620, CK211469, TC400362, TC397729, TC434831, TC432185, TC396895, TC426326, TC381462, TC382342, TC393106, TC401758, TC373914, TC432504, TC392263, TC397676, TC396460, TC428944, TC389661 |
| [Cellular nitrogen compound metabolic process](http://amigo.geneontology.org/cgi-bin/amigo/go.cgi?action=query&view=query&query=GO:0034641&search_constraint=terms) | TC392303, TC410352, TC386040, TC373613, TC390393, TC372330, TC397258, TC425957, TC375864, TC377766, TC445166, TC435533, TC374240, TC417106, TC375313, CA710880, TC457112, TC398862, TC433162, TC376351, TC387579, TC371242, TC413854, TC423252, TC397660, TC393814, TC389363, TC388914, TC434442, BJ279521, TC376220, TC377438, TC458562, TC380590, TC411471, TC394716, TC398633, TC446235, TC387410, TC378878, TC380063, TC433844, TC393830, TC397176, TC380943, TC418414, CJ944525, TC380433, TC378054, TC420735, TC373702, TC404371, TC395453, TC386961, TC384219, TC384735, TC390569, TC405540, TC416685, TC372664, TC419057, TC403872, TC389090, TC389375, TC382655, CK200433, TC373791, TC398714, TC373994, BQ294582, TC370114, TC406038, TC395351, TC403580, TC401915, GH732878, CK210754, TC369928, TC385710, CA632212, DR739994, TC384454, TC376490, TC411287, TC394916, EB512907, TC390994, TC378274, TC391143, CK217367, TC391962, TC423804, TC379853, TC421880, TC370315, TC386414, TC400755, TC372701, TC405511, TC395723, TC440636, TC405440, TC377190, TC370885, TC443814, TC404843, TC385233, TC389718, TC401210, DR737360, TC379357, TC387135, TC377225, TC424376, TC386279, TC381943, BQ607161, TC406807, TC393960, TC372845, TC404606, TC369628, TC389162, DR739471, TC416906, TC402308, TC434820, TC443387, TC441343, TC379711, TC388566, TC379903, TC392272, TC390285, TC377061, TC417077, TC417388, TC386519, TC386313, CV759879, TC402072, TC408907, TC402440, TC391438, TC417341, TC394307, TC409208, TC375146, TC386963, TC432369, TC454407, TC389589, TC381988, TC452762, TC384122, TC386344, TC392778, TC394661, TC397312, TC370350, TC405030, TC381963, TC388403, TC383176, TC371455, TC386422, TC374164, TC388688, TC385780, TC388822, TC406708, TC388976, TC368549, TC377496, TC429771, TC410078, TC423576, TC409459, TC386639, TC397500, TC402186, TC416658, TC402668, TC396650, TC393561, TC393692, TC391671, TC393554, TC407614, TC458205, TC459656, TC402603, TC423354, TC441241, TC449724, TC413571, TC379436, CA729339, CK214224, TC387710, TC394118, TC380882, TC423880, TC433589, TC377410, TC381218, TC418091, TC399352, TC389816, TC451519, CA613620, TC393820, CK211469, CJ930688, TC381817, TC369092, TC403588, TC397729, TC434831, TC396895, TC432185, TC426326, TC381462, TC382342, TC393106, TC401758, TC373914, TC432504, TC396460, TC397676, TC392263, TC428944, TC389661 |
| [Sensory perception of mechanical stimulus](http://amigo.geneontology.org/cgi-bin/amigo/go.cgi?action=query&view=query&query=GO:0050954&search_constraint=terms) | TC410352, TC401915, DR739471, TC393561, TC434820, TC443387, TC379711, TC391671, TC393554, TC407614, TC392272, TC459656, DR739994, TC384454, CA729339, TC402440, CK217367, TC423252, TC394307, TC397660, TC375146, TC389363, TC379853, TC379171, TC388914, CK199175, TC389589, TC389816, TC446235, TC405440, TC392778, TC420735, TC386422, TC395453, DR737360, TC388688, TC416685, TC390569, TC403872, TC389090, TC434831, TC396895, TC389375, TC426326, TC388822, TC377496, TC406708, TC382655, TC393106, TC429771, CK200433, BQ607161, TC423576, TC406807, TC406038, TC396460, TC416658, TC428944, TC395351, TC386646 |
| [Chromatin silencing at centromere](http://amigo.geneontology.org/cgi-bin/amigo/go.cgi?action=query&view=query&query=GO:0030702&search_constraint=terms) | TC410352, TC401915, DR739471, TC393561, TC434820, TC443387, TC379711, TC391671, TC393554, TC407614, TC392272, DR739994, TC384454, CA729339, CA710880, TC402440, CK217367, TC423252, TC394307, TC397660, TC375146, TC389363, TC379853, TC388914, TC389589, TC389816, TC446235, TC405440, CA613620, TC392778, TC420735, TC386422, TC395453, DR737360, TC388688, TC416685, TC390569, TC403872, TC389090, TC434831, TC396895, TC389375, TC426326, TC388822, TC377496, TC406708, TC382655, TC393106, TC429771, CK200433, TC423576, BQ294582, BQ607161, TC406038, TC396460, TC416658, TC428944, TC395351 |
| [RNA metabolic process](http://amigo.geneontology.org/cgi-bin/amigo/go.cgi?action=query&view=query&query=GO:0016070&search_constraint=terms) | TC410352, TC386040, TC390393, TC397258, TC425957, TC445166, TC435533, TC374240, TC375313, CA710880, TC398862, TC433162, TC423252, TC397660, TC393814, TC389363, TC388914, TC376220, TC394716, TC398633, TC446235, TC387410, TC378878, TC380063, TC393830, TC397176, TC380943, TC418414, TC380433, TC378054, TC420735, TC404371, TC395453, TC384219, TC390569, TC405540, TC416685, TC372664, TC403872, TC389090, TC389375, TC382655, CK200433, TC373791, TC370114, TC406038, TC395351, TC401915, DR739994, TC384454, TC411287, EB512907, TC391143, CK217367, TC423804, TC379853, TC386414, TC440636, TC395723, TC405440, TC404843, TC385233, TC389718, DR737360, TC379357, TC387135, TC386279, TC381943, BQ607161, TC393960, TC404606, TC369628, DR739471, TC416906, TC402308, TC434820, TC443387, TC379711, TC388566, TC379903, TC392272, TC417388, TC386519, CV759879, TC408907, TC402440, TC391438, TC417341, TC394307, TC375146, TC389589, TC381988, TC452762, TC386344, TC392778, TC405030, TC381963, TC388403, TC383176, TC386422, TC388688, TC385780, TC388822, TC377496, TC388976, TC406708, TC429771, TC410078, TC409459, TC423576, TC386639, TC416658, TC402186, TC393561, TC393692, TC391671, TC393554, TC407614, TC402603, TC449724, TC379436, CA729339, CK214224, TC387710, TC394118, TC433589, TC389816, TC399352, CA613620, TC393820, CK211469, TC396895, TC434831, TC426326, TC382342, TC393106, TC373914, TC397676, TC396460, TC432504, TC428944 |
| [Signaling pathway](http://amigo.geneontology.org/cgi-bin/amigo/go.cgi?action=query&view=query&query=GO:0023033&search_constraint=terms) | TC410352, TC386040, TC407978, TC397258, CV775873, TC435533, TC374240, TC398052, CA710880, TC425841, TC398862, TC433162, TC387579, TC394459, TC371242, TC384738, TC423252, TC397660, TC389363, TC388914, TC388410, TC394716, TC398633, TC446235, TC387410, TC380063, TC393830, TC380943, TC418414, TC380433, TC378054, TC420735, TC404371, TC395453, TC386961, TC384219, TC392329, TC390569, TC416685, TC407076, TC419057, TC403872, TC389090, TC389375, TC404413, TC382655, CK200433, BQ294582, TC370114, TC406038, TC393970, TC395351, TC390904, TC401915, TC459245, TC393100, CA632212, DR739994, TC384454, TC411287, TC380329, TC386237, TC393904, TC391143, CK217367, TC409599, TC379853, TC395303, TC386414, TC400755, TC405511, TC395723, TC440636, TC405440, TC403968, TC376420, TC458987, TC389718, TC401210, TC390135, DR737360, TC379357, TC377225, TC386279, TC381943, BQ607161, TC393960, TC382045, TC382830, DR739471, TC416906, TC402308, TC434820, TC443387, CK212850, TC379711, TC378790, TC392272, TC377061, TC417388, TC386519, TC386313, CV759879, TC408907, TC402440, TC420579, TC391438, TC394307, CK211707, TC375146, TC386963, TC398592, TC389589, TC386344, TC392778, TC397312, TC405030, TC381963, CK211589, TC388403, TC383176, TC383909, TC449463, TC435595, TC386422, TC388688, TC405041, TC385780, TC412150, TC399919, TC407572, TC372175, TC388822, TC396365, TC377496, TC388976, TC406708, TC419747, TC429771, TC423576, TC386639, TC402186, TC416658, TC393561, TC393692, TC391671, TC393554, TC407614, TC382928, TC402603, TC423354, TC438243, TC413571, TC379436, CA729339, TC417363, CK214224, TC411941, BE585841, TC425690, TC373259, TC433589, TC377410, TC381218, TC389816, TC372530, CA613620, CK211469, TC400362, TC397729, TC434831, TC432185, TC396895, TC426326, TC381462, TC382342, TC393106, TC377749, TC401758, TC373914, TC432504, TC392263, TC397676, TC396460, TC428944, TC389661 |
| [Sodium ion transport](http://amigo.geneontology.org/cgi-bin/amigo/go.cgi?action=query&view=query&query=GO:0006814&search_constraint=terms) | TC410352, TC401915, DR739471, TC393561, TC434820, TC443387, TC379711, TC391671, TC393554, TC407614, TC392272, TC459656, DR739994, TC384454, CA729339, TC402440, CK217367, TC423252, TC394307, TC397660, TC375146, TC389363, TC379853, TC388914, TC389589, TC389816, TC446235, TC405440, TC392778, TC420735, TC386422, TC395453, DR737360, TC388688, TC388049, TC416685, TC390569, TC403872, TC389090, TC434831, TC396895, TC389375, TC426326, TC388822, TC377496, TC406708, TC382655, TC393106, TC429771, CK200433, BQ607161, TC423576, TC406807, TC406038, TC396460, TC416658, TC428944, TC395351, TC386646 |
| [Regulation of catalytic activity](http://amigo.geneontology.org/cgi-bin/amigo/go.cgi?action=query&view=query&query=GO:0050790&search_constraint=terms) | TC410352, DR739471, TC434820, TC443387, TC379711, TC392272, CV775873, TC417077, TC386313, TC398052, CA710880, TC425841, TC402440, TC387579, TC394459, TC371242, TC384738, TC423252, TC394307, TC397660, CK211707, TC375146, TC386963, TC389363, TC388914, TC388410, TC389589, TC446235, TC387410, TC392778, TC397312, TC420735, TC395453, TC386422, TC386961, TC388688, TC390569, TC416685, TC407076, TC403872, TC399919, TC407572, TC389090, TC372175, TC389375, TC388822, TC396365, TC406708, TC377496, TC419747, TC382655, TC429771, CK200433, TC423576, BQ294582, TC406038, TC416658, TC395351, TC401915, TC393561, TC393100, TC391671, TC393554, TC407614, TC382928, TC423354, DR739994, TC438243, TC384454, CA729339, TC417363, TC386237, TC393904, CK217367, TC409599, TC380882, TC423880, TC373259, TC379853, TC377410, TC381218, TC395303, TC389816, TC400755, TC405511, TC405440, TC376420, TC458987, TC377190, CA613620, TC423110, TC403588, TC401210, DR737360, TC390135, TC397729, TC377225, TC434831, TC396895, TC426326, TC381462, TC393106, TC401758, BQ607161, TC392263, TC396460, TC428944, TC389661, TC382045 |
| [Signal transduction in response to DNA damage](http://amigo.geneontology.org/cgi-bin/amigo/go.cgi?action=query&view=query&query=GO:0042770&search_constraint=terms) | TC386040, TC402308, TC407978, TC393692, TC397258, CA632212, TC402603, TC377061, TC438243, TC379436, TC411287, TC435533, TC386519, TC374240, CV759879, CK214224, TC408907, TC391143, TC433162, TC391438, TC433589, TC386414, TC394716, TC398633, TC440636, TC395723, TC380063, TC380943, TC393830, TC386344, CK211469, TC405030, TC378054, TC381963, TC388403, TC383176, TC404371, TC389718, TC384219, TC379357, TC386279, TC385780, TC419057, TC381943, TC382342, TC388976, TC373914, TC432504, TC386639, TC397676, TC402186, TC382045 |
| [Double fertilization forming a zygote and endosperm](http://amigo.geneontology.org/cgi-bin/amigo/go.cgi?action=query&view=query&query=GO:0009567&search_constraint=terms) | TC410352, TC401915, DR739471, TC393561, TC434820, TC443387, TC379711, TC391671, TC393554, TC407614, TC392272, DR739994, TC384454, CA729339, TC402440, CK217367, TC423252, TC394307, TC397660, TC375146, TC389363, TC379853, TC388914, TC389589, TC389816, TC446235, TC405440, CA613620, TC392778, TC420735, TC386422, TC395453, TC388688, TC416685, TC390569, TC403872, TC389090, TC434831, TC396895, TC389375, TC426326, TC388822, TC377496, TC406708, TC382655, TC393106, TC429771, CK200433, BQ294582, BQ607161, TC423576, TC406038, TC396460, TC416658, TC428944, TC395351 |
| [Adult behavior](http://amigo.geneontology.org/cgi-bin/amigo/go.cgi?action=query&view=query&query=GO:0030534&search_constraint=terms) | TC410352, TC401915, DR739471, TC393561, TC434820, TC443387, TC379711, TC391671, TC393554, TC407614, TC392272, DR739994, TC384454, CA729339, CA710880, TC402440, CK217367, TC423252, TC394307, TC397660, TC375146, TC389363, TC379853, TC388914, TC389589, TC389816, TC446235, TC405440, CA613620, TC392778, TC420735, TC386422, TC395453, DR737360, TC388688, TC390569, TC416685, TC403872, TC389090, TC434831, TC396895, TC389375, TC426326, TC388822, TC377496, TC406708, TC382655, TC393106, TC429771, CK200433, TC423576, BQ294582, BQ607161, TC406038, TC396460, TC416658, TC428944, TC395351, TC382045 |
| [Regulation of cellular biosynthetic process](http://amigo.geneontology.org/cgi-bin/amigo/go.cgi?action=query&view=query&query=GO:0031326&search_constraint=terms) | TC392303, TC410352, TC386040, TC390393, TC397258, TC389190, CV775873, TC372677, TC435533, TC374240, TC398052, CA710880, TC425841, TC398862, TC433162, TC387579, TC394459, TC371242, TC384738, TC423252, TC397660, TC393814, TC389363, TC388914, TC388410, TC458562, TC394716, TC398633, TC446235, TC404926, TC380063, TC402545, TC393830, TC380943, TC418414, TC380433, TC378054, TC420735, TC404371, TC395453, TC386961, TC384219, TC373615, TC392329, TC384735, TC390569, CA682223, TC416685, TC407076, TC419057, TC403872, TC389090, TC389375, TC404413, TC382655, CK200433, TC398714, TC373994, TC370114, BQ294582, TC406038, CJ550278, TC408192, TC395351, TC401915, TC393100, CK210754, CA632212, DR739994, TC384454, TC411287, TC380329, TC386237, TC394916, TC393904, TC390994, TC398731, TC378274, TC391143, CK217367, TC409599, TC379853, TC395303, TC386414, TC400755, TC395723, TC440636, TC406193, TC405440, TC403968, TC405615, TC376420, TC458987, TC377190, TC422348, TC385233, TC389718, TC401210, TC390135, DR737360, TC379357, TC377225, TC386279, TC388049, TC381943, BQ607161, TC403977, TC406807, TC393960, TC426358, TC382045, DR739471, TC416906, TC402308, TC434820, TC443387, TC441343, TC379711, TC379903, TC392272, TC377061, TC417077, TC407340, TC417388, TC386519, TC386313, CV759879, CK208222, TC408907, TC402440, TC420579, TC391438, TC394307, CK211707, TC375146, TC386963, TC389589, TC452762, TC386344, TC392778, TC397312, TC405030, TC381963, CK211589, TC388403, TC383176, TC386422, TC388688, TC419584, TC385780, TC399919, TC407572, TC372175, TC388822, TC396365, TC377496, TC388976, TC406708, TC419747, TC429771, TC409459, TC423576, TC386639, TC402186, TC416658, TC374230, TC393561, TC393692, TC391671, TC393554, TC407614, TC459656, TC382928, TC402603, TC441241, TC413571, TC379436, CA729339, TC417363, TC460615, CK214224, TC425690, TC433589, TC377410, TC381218, TC389816, CA613620, CK211469, TC400362, TC397729, TC395872, TC434831, TC396895, TC426326, TC381462, TC418850, TC382342, TC393106, TC401758, TC373914, TC432504, TC392263, TC397676, TC396460, TC428944, TC389661 |
| [Calcium ion-dependent exocytosis](http://amigo.geneontology.org/cgi-bin/amigo/go.cgi?action=query&view=query&query=GO:0017156&search_constraint=terms) | TC398304, TC393100, TC433957, CK210754, TC382928, CV775873, TC441241, TC417363, TC386237, TC394916, TC386313, TC390994, TC398052, TC425841, TC378274, TC403929, TC387579, CA606693, TC394459, TC384738, CK211707, TC386963, TC388410, TC395303, TC422425, TC458562, TC400755, TC458987, TC396751, CA615187, TC386961, TC398514, TC390135, TC377225, TC411191, TC388547, TC387344, TC405784, TC399919, TC407572, TC372175, TC396365, TC419747, TC380662, TC401758, TC398714, TC389661 |
| [Synaptic vesicle exocytosis](http://amigo.geneontology.org/cgi-bin/amigo/go.cgi?action=query&view=query&query=GO:0016079&search_constraint=terms) | TC398304, TC393100, TC433957, CK210754, TC382928, CV775873, TC441241, TC417363, TC386237, TC394916, TC386313, TC390994, TC398052, TC425841, TC378274, TC403929, TC387579, CA606693, TC394459, TC384738, CK211707, TC386963, TC388410, TC395303, TC422425, TC458562, TC400755, TC458987, TC396751, CA615187, TC386961, TC398514, TC390135, TC377225, TC411191, TC388547, TC387344, TC405784, TC399919, TC407572, TC372175, TC396365, TC419747, TC380662, TC401758, TC398714, TC389661 |
| [Regulation of cell cycle process](http://amigo.geneontology.org/cgi-bin/amigo/go.cgi?action=query&view=query&query=GO:0010564&search_constraint=terms) | TC386040, TC402308, TC407978, TC393692, TC394796, TC397258, TC402603, TC377061, TC438243, TC379436, TC411287, TC435533, TC386519, TC374240, CV759879, CK214224, TC408907, TC391143, TC433162, TC391438, TC433589, TC386414, TC394716, TC398633, TC440636, TC395723, TC380063, TC380943, TC393830, TC423110, TC386344, CK211469, TC400362, TC405030, TC378054, TC381963, TC388403, TC383176, TC404371, TC389718, TC384219, TC379357, TC386279, TC385780, TC381943, TC404413, TC382342, TC388976, TC373914, TC432504, TC386639, TC397676, TC402186, TC393960, TC382045 |
| [DNA damage checkpoint](http://amigo.geneontology.org/cgi-bin/amigo/go.cgi?action=query&view=query&query=GO:0000077&search_constraint=terms) | TC386040, TC402308, TC407978, TC393692, TC397258, TC402603, TC377061, TC438243, TC379436, TC411287, TC435533, TC386519, TC374240, CV759879, CK214224, TC408907, TC391143, TC433162, TC391438, TC433589, TC386414, TC398633, TC394716, TC440636, TC395723, TC380063, TC380943, TC393830, TC386344, CK211469, TC405030, TC378054, TC381963, TC388403, TC383176, TC404371, TC389718, TC384219, TC379357, TC386279, TC385780, TC381943, TC388976, TC382342, TC373914, TC432504, TC386639, TC397676, TC402186, TC382045 |
| [DNA integrity checkpoint](http://amigo.geneontology.org/cgi-bin/amigo/go.cgi?action=query&view=query&query=GO:0031570&search_constraint=terms) | TC386040, TC402308, TC407978, TC393692, TC397258, TC402603, TC377061, TC438243, TC379436, TC411287, TC435533, TC386519, TC374240, CV759879, CK214224, TC408907, TC391143, TC433162, TC391438, TC433589, TC386414, TC398633, TC394716, TC440636, TC395723, TC380063, TC380943, TC393830, TC386344, CK211469, TC405030, TC378054, TC381963, TC388403, TC383176, TC404371, TC389718, TC384219, TC379357, TC386279, TC385780, TC381943, TC388976, TC382342, TC373914, TC432504, TC386639, TC397676, TC402186, TC382045 |
| [Synaptic vesicle transport](http://amigo.geneontology.org/cgi-bin/amigo/go.cgi?action=query&view=query&query=GO:0048489&search_constraint=terms) | TC398304, TC393100, TC433957, CK210754, TC382928, CV775873, TC441241, TC417363, TC386237, TC394916, TC386313, TC390994, TC398052, TC425841, TC378274, TC403929, TC387579, CA606693, TC394459, TC384738, CK211707, TC386963, TC388410, TC395303, TC422425, TC458562, TC400755, TC458987, TC396751, CA615187, TC386961, TC398514, TC390135, TC377225, TC411191, TC388547, TC387344, TC405784, TC399919, TC407572, TC372175, TC409650, TC396365, TC419747, TC380662, TC401758, TC398714, TC389661, TC382045 |
| [Adult locomotory behavior](http://amigo.geneontology.org/cgi-bin/amigo/go.cgi?action=query&view=query&query=GO:0008344&search_constraint=terms) | TC410352, TC401915, DR739471, TC393561, TC434820, TC443387, TC379711, TC391671, TC393554, TC407614, TC392272, DR739994, TC384454, CA729339, CA710880, TC402440, CK217367, TC423252, TC394307, TC397660, TC375146, TC389363, TC379853, TC388914, TC389589, TC389816, TC446235, TC405440, CA613620, TC392778, TC420735, TC386422, TC395453, DR737360, TC388688, TC416685, TC390569, TC403872, TC389090, TC434831, TC396895, TC389375, TC426326, TC388822, TC377496, TC406708, TC382655, TC393106, TC429771, CK200433, TC423576, BQ294582, BQ607161, TC406038, TC396460, TC416658, TC428944, TC395351 |
| [Neurotransmitter secretion](http://amigo.geneontology.org/cgi-bin/amigo/go.cgi?action=query&view=query&query=GO:0007269&search_constraint=terms) | TC398304, TC393100, TC433957, CK210754, TC382928, CV775873, TC441241, TC417363, TC386237, TC394916, TC386313, TC390994, TC398052, TC425841, TC378274, TC403929, TC387579, CA606693, TC394459, TC384738, CK211707, TC386963, TC388410, TC395303, TC422425, TC458562, TC400755, TC458987, TC396751, CA615187, TC386961, TC398514, TC390135, TC377225, TC411191, TC388547, TC387344, TC405784, TC399919, TC407572, TC372175, TC409650, TC396365, TC419747, TC380662, TC401758, TC398714, TC389661 |
| [Activation of adenylate cyclase activity by G-protein signaling pathway](http://amigo.geneontology.org/cgi-bin/amigo/go.cgi?action=query&view=query&query=GO:0007189&search_constraint=terms) | TC410352, TC401915, DR739471, TC393561, TC434820, TC443387, TC379711, TC391671, TC393554, TC407614, TC392272, DR739994, TC384454, CA729339, CA710880, TC402440, CK217367, TC423252, TC394307, TC397660, TC375146, TC389363, TC379853, TC388914, TC389589, TC389816, TC446235, TC405440, CA613620, TC392778, TC420735, TC386422, TC395453, DR737360, TC388688, TC416685, TC390569, TC403872, TC389090, TC434831, TC396895, TC389375, TC426326, TC388822, TC377496, TC406708, TC382655, TC393106, TC429771, CK200433, TC423576, BQ294582, BQ607161, TC406038, TC396460, TC416658, TC428944, TC395351 |
| [Regulation of adenylate cyclase activity involved in G-protein signaling pathway](http://amigo.geneontology.org/cgi-bin/amigo/go.cgi?action=query&view=query&query=GO:0010578&search_constraint=terms) | TC410352, TC401915, DR739471, TC393561, TC434820, TC443387, TC379711, TC391671, TC393554, TC407614, TC392272, DR739994, TC384454, CA729339, CA710880, TC402440, CK217367, TC423252, TC394307, TC397660, TC375146, TC389363, TC379853, TC388914, TC389589, TC389816, TC446235, TC405440, CA613620, TC392778, TC420735, TC386422, TC395453, DR737360, TC388688, TC416685, TC390569, TC403872, TC389090, TC434831, TC396895, TC389375, TC426326, TC388822, TC377496, TC406708, TC382655, TC393106, TC429771, CK200433, TC423576, BQ294582, BQ607161, TC406038, TC396460, TC416658, TC428944, TC395351 |
| [Positive regulation of adenylate cyclase activity by G-protein signaling pathway](http://amigo.geneontology.org/cgi-bin/amigo/go.cgi?action=query&view=query&query=GO:0010579&search_constraint=terms) | TC410352, TC401915, DR739471, TC393561, TC434820, TC443387, TC379711, TC391671, TC393554, TC407614, TC392272, DR739994, TC384454, CA729339, CA710880, TC402440, CK217367, TC423252, TC394307, TC397660, TC375146, TC389363, TC379853, TC388914, TC389589, TC389816, TC446235, TC405440, CA613620, TC392778, TC420735, TC386422, TC395453, DR737360, TC388688, TC416685, TC390569, TC403872, TC389090, TC434831, TC396895, TC389375, TC426326, TC388822, TC377496, TC406708, TC382655, TC393106, TC429771, CK200433, TC423576, BQ294582, BQ607161, TC406038, TC396460, TC416658, TC428944, TC395351 |
| [Regulation of biosynthetic process](http://amigo.geneontology.org/cgi-bin/amigo/go.cgi?action=query&view=query&query=GO:0009889&search_constraint=terms) | TC392303, TC410352, TC386040, TC390393, TC397258, TC389190, CV775873, TC372677, TC435533, TC374240, TC398052, CA710880, TC425841, TC398862, TC433162, TC387579, TC394459, TC371242, TC384738, TC423252, TC397660, TC393814, TC389363, TC388914, TC388410, TC458562, TC394716, TC398633, TC446235, TC404926, TC380063, TC402545, TC393830, TC380943, TC418414, TC380433, TC378054, TC420735, TC404371, TC395453, TC386961, TC384219, TC373615, TC392329, TC384735, TC390569, CA682223, TC416685, TC407076, TC419057, TC403872, TC389090, TC389375, TC404413, TC382655, CK200433, TC398714, TC373994, TC370114, BQ294582, TC406038, CJ550278, TC408192, TC395351, TC401915, TC393100, CK210754, CA632212, DR739994, TC384454, TC411287, TC380329, TC386237, TC394916, TC393904, TC390994, TC398731, TC378274, TC391143, CK217367, TC409599, TC379853, TC395303, TC386414, TC400755, TC395723, TC440636, TC406193, TC405440, TC403968, TC405615, TC376420, TC458987, TC377190, TC422348, TC385233, TC389718, TC401210, TC390135, DR737360, TC379357, TC377225, TC386279, TC388049, TC381943, BQ607161, TC403977, TC406807, TC393960, TC426358, TC382045, DR739471, TC416906, TC402308, TC434820, TC443387, TC441343, TC379711, TC379903, TC392272, TC377061, TC417077, TC407340, TC417388, TC386519, TC386313, CV759879, CK208222, TC408907, TC402440, TC420579, TC391438, TC394307, CK211707, TC375146, TC386963, TC389589, TC452762, TC386344, TC392778, TC397312, TC405030, TC381963, CK211589, TC388403, TC383176, TC386422, TC388688, TC419584, TC385780, TC399919, TC407572, TC372175, TC388822, TC396365, TC377496, TC388976, TC406708, TC419747, TC429771, TC409459, TC423576, TC386639, TC402186, TC416658, TC374230, TC393561, TC393692, TC391671, TC393554, TC407614, TC459656, TC382928, TC402603, TC441241, TC413571, TC379436, CA729339, TC417363, TC460615, CK214224, TC425690, TC433589, TC377410, TC381218, TC389816, CA613620, CK211469, TC400362, TC397729, TC395872, TC434831, TC396895, TC426326, TC381462, TC418850, TC382342, TC393106, TC401758, TC373914, TC432504, TC392263, TC397676, TC396460, TC428944, TC389661 |
| [Metal ion transport](http://amigo.geneontology.org/cgi-bin/amigo/go.cgi?action=query&view=query&query=GO:0030001&search_constraint=terms) | CA646741, TC410352, DR739471, TC434820, TC443387, TC379711, TC392272, TC429341, TC374240, TC402440, TC423252, TC394307, TC397660, TC375146, TC389363, TC388914, TC389589, TC446235, TC392778, CK211589, TC420735, TC386422, TC395453, TC388688, TC390569, TC416685, TC403872, TC389090, TC389375, TC388822, TC377496, TC406708, TC382655, TC429771, CK200433, TC423576, TC406038, CJ550278, TC416658, TC395351, TC386646, TC374230, TC393561, TC401915, TC456619, TC391671, TC393554, TC407614, TC459656, DR739994, TC384454, TC413571, CA729339, CK217367, TC379853, TC389816, TC370315, TC395723, TC405440, DR737360, TC444402, TC388049, TC396895, TC434831, TC426326, TC393106, BQ607161, TC396460, TC406807, TC428944, TC382045 |
| [Regulation of neurotransmitter levels](http://amigo.geneontology.org/cgi-bin/amigo/go.cgi?action=query&view=query&query=GO:0001505&search_constraint=terms) | TC398304, TC393100, TC433957, CK210754, TC382928, CV775873, TC441241, TC417363, TC386237, TC394916, TC386313, TC390994, TC398052, TC425841, TC378274, TC403929, TC387579, CA606693, TC394459, TC384738, CK211707, TC386963, TC388410, TC395303, TC422425, TC458562, TC400755, TC458987, TC396751, CA615187, TC386961, TC398514, TC390135, TC377225, TC411191, TC388547, TC387344, TC405784, TC399919, TC407572, TC372175, TC409650, TC396365, TC419747, TC380662, TC401758, TC398714, TC389661 |
| [Chromatin silencing](http://amigo.geneontology.org/cgi-bin/amigo/go.cgi?action=query&view=query&query=GO:0006342&search_constraint=terms) | TC410352, TC401915, DR739471, TC393561, TC434820, TC443387, TC379711, TC391671, TC393554, TC407614, TC392272, DR739994, TC384454, CA729339, TC374240, CA710880, TC402440, CK217367, TC423252, TC394307, TC397660, TC375146, TC389363, TC379853, TC388914, TC389589, TC389816, TC446235, TC395723, TC405440, CA613620, TC392778, TC420735, TC386422, TC395453, DR737360, TC388688, TC390569, TC416685, TC403872, TC389090, TC434831, TC396895, TC389375, TC426326, TC388822, TC377496, TC406708, TC382655, TC393106, TC429771, CK200433, TC423576, BQ294582, BQ607161, TC406038, CJ550278, TC396460, TC416658, TC428944, TC393960, TC395351 |
| [Negative regulation of gene expression, epigenetic](http://amigo.geneontology.org/cgi-bin/amigo/go.cgi?action=query&view=query&query=GO:0045814&search_constraint=terms) | TC410352, TC401915, DR739471, TC393561, TC434820, TC443387, TC379711, TC391671, TC393554, TC407614, TC392272, DR739994, TC384454, CA729339, TC374240, CA710880, TC402440, CK217367, TC423252, TC394307, TC397660, TC375146, TC389363, TC379853, TC388914, TC389589, TC389816, TC446235, TC395723, TC405440, CA613620, TC392778, TC420735, TC386422, TC395453, DR737360, TC388688, TC390569, TC416685, TC403872, TC389090, TC434831, TC396895, TC389375, TC426326, TC388822, TC377496, TC406708, TC382655, TC393106, TC429771, CK200433, TC423576, BQ294582, BQ607161, TC406038, CJ550278, TC396460, TC416658, TC428944, TC393960, TC395351 |
| [DNA recombination](http://amigo.geneontology.org/cgi-bin/amigo/go.cgi?action=query&view=query&query=GO:0006310&search_constraint=terms) | TC386040, TC402308, TC393692, TC397258, TC458205, TC402603, TC379436, TC411287, TC435533, TC386519, TC374240, CV759879, CK214224, TC408907, TC391143, TC433162, TC391438, TC433589, TC386414, TC398633, TC394716, TC440636, TC395723, TC380063, TC380943, TC393830, TC386344, CK211469, TC405030, TC378054, TC381963, TC388403, TC383176, TC404371, TC389718, TC384219, TC379357, TC386279, TC385780, TC381943, TC388976, TC382342, TC373914, TC432504, TC386639, TC397676, TC402186 |
| [Gene silencing](http://amigo.geneontology.org/cgi-bin/amigo/go.cgi?action=query&view=query&query=GO:0016458&search_constraint=terms) | TC410352, TC401915, DR739471, TC393561, TC434820, TC443387, TC379711, TC391671, TC393554, TC407614, TC392272, DR739994, TC384454, CA729339, TC374240, CA710880, TC402440, CK217367, TC423252, TC394307, TC397660, TC375146, TC389363, TC379853, TC388914, TC389589, TC389816, TC446235, TC395723, TC405440, CA613620, TC392778, TC420735, TC386422, TC395453, DR737360, TC388688, TC390569, TC416685, TC403872, TC389090, TC434831, TC396895, TC389375, TC426326, TC388822, TC377496, TC406708, TC382655, TC393106, TC429771, CK200433, TC423576, BQ294582, BQ607161, TC406038, CJ550278, TC396460, TC416658, TC428944, TC393960, TC395351 |
| [Regulation of transcription](http://amigo.geneontology.org/cgi-bin/amigo/go.cgi?action=query&view=query&query=GO:0045449&search_constraint=terms) | TC392303, TC410352, TC386040, TC390393, TC397258, TC389190, TC372677, TC435533, TC374240, CA710880, TC398862, TC433162, TC387579, TC423252, TC397660, TC393814, TC389363, TC388914, TC458562, TC394716, TC398633, TC446235, TC404926, TC380063, TC402545, TC393830, TC380943, TC418414, TC380433, TC378054, TC420735, TC404371, TC395453, TC386961, TC384219, TC373615, TC392329, TC390569, CA682223, TC416685, TC407076, TC419057, TC403872, TC389090, TC389375, TC404413, TC382655, CK200433, TC398714, TC373994, TC370114, BQ294582, TC406038, CJ550278, TC408192, TC395351, TC401915, CK210754, CA632212, DR739994, TC384454, TC411287, TC380329, TC394916, TC390994, TC398731, TC378274, TC391143, CK217367, TC409599, TC379853, TC386414, TC400755, TC440636, TC395723, TC406193, TC405440, TC403968, TC405615, TC377190, TC422348, TC385233, TC389718, TC401210, DR737360, TC379357, TC377225, TC386279, TC381943, BQ607161, TC403977, TC406807, TC393960, TC426358, TC382045, DR739471, TC416906, TC402308, TC434820, TC443387, TC379711, TC379903, TC392272, TC417077, TC417388, TC386519, TC386313, CV759879, TC408907, TC402440, TC420579, TC391438, TC394307, TC375146, TC386963, TC389589, TC452762, TC386344, TC392778, TC405030, TC397312, TC381963, CK211589, TC388403, TC383176, TC386422, TC388688, TC419584, TC385780, TC388822, TC377496, TC388976, TC406708, TC429771, TC409459, TC423576, TC386639, TC402186, TC416658, TC374230, TC393561, TC393692, TC391671, TC393554, TC407614, TC459656, TC402603, TC441241, TC413571, TC379436, CA729339, TC460615, CK214224, TC425690, TC433589, TC377410, TC381218, TC389816, CA613620, CK211469, TC400362, TC397729, TC395872, TC434831, TC396895, TC426326, TC381462, TC418850, TC382342, TC393106, TC401758, TC373914, TC432504, TC392263, TC397676, TC396460, TC428944, TC389661 |
| [Activation of adenylate cyclase activity](http://amigo.geneontology.org/cgi-bin/amigo/go.cgi?action=query&view=query&query=GO:0007190&search_constraint=terms) | TC410352, TC401915, DR739471, TC393561, TC434820, TC443387, TC379711, TC391671, TC393554, TC407614, TC392272, DR739994, TC384454, TC417077, CA729339, CA710880, TC402440, CK217367, TC423252, TC394307, TC397660, TC375146, TC389363, TC379853, TC388914, TC389589, TC389816, TC446235, TC405440, CA613620, TC392778, TC420735, TC386422, TC395453, DR737360, TC388688, TC390569, TC416685, TC403872, TC389090, TC434831, TC396895, TC389375, TC426326, TC388822, TC377496, TC406708, TC382655, TC393106, TC429771, CK200433, TC423576, BQ294582, BQ607161, TC406038, TC396460, TC416658, TC428944, TC395351 |
| [Positive regulation of cyclase activity](http://amigo.geneontology.org/cgi-bin/amigo/go.cgi?action=query&view=query&query=GO:0031281&search_constraint=terms) | TC410352, TC401915, DR739471, TC393561, TC434820, TC443387, TC379711, TC391671, TC393554, TC407614, TC392272, DR739994, TC384454, TC417077, CA729339, CA710880, TC402440, CK217367, TC423252, TC394307, TC397660, TC375146, TC389363, TC379853, TC388914, TC389589, TC389816, TC446235, TC405440, CA613620, TC392778, TC420735, TC386422, TC395453, DR737360, TC388688, TC390569, TC416685, TC403872, TC389090, TC434831, TC396895, TC389375, TC426326, TC388822, TC377496, TC406708, TC382655, TC393106, TC429771, CK200433, TC423576, BQ294582, BQ607161, TC406038, TC396460, TC416658, TC428944, TC395351 |
| [Positive regulation of adenylate cyclase activity](http://amigo.geneontology.org/cgi-bin/amigo/go.cgi?action=query&view=query&query=GO:0045762&search_constraint=terms) | TC410352, TC401915, DR739471, TC393561, TC434820, TC443387, TC379711, TC391671, TC393554, TC407614, TC392272, DR739994, TC384454, TC417077, CA729339, CA710880, TC402440, CK217367, TC423252, TC394307, TC397660, TC375146, TC389363, TC379853, TC388914, TC389589, TC389816, TC446235, TC405440, CA613620, TC392778, TC420735, TC386422, TC395453, DR737360, TC388688, TC390569, TC416685, TC403872, TC389090, TC434831, TC396895, TC389375, TC426326, TC388822, TC377496, TC406708, TC382655, TC393106, TC429771, CK200433, TC423576, BQ294582, BQ607161, TC406038, TC396460, TC416658, TC428944, TC395351 |
| [Positive regulation of lyase activity](http://amigo.geneontology.org/cgi-bin/amigo/go.cgi?action=query&view=query&query=GO:0051349&search_constraint=terms) | TC410352, TC401915, DR739471, TC393561, TC434820, TC443387, TC379711, TC391671, TC393554, TC407614, TC392272, DR739994, TC384454, TC417077, CA729339, CA710880, TC402440, CK217367, TC423252, TC394307, TC397660, TC375146, TC389363, TC379853, TC388914, TC389589, TC389816, TC446235, TC405440, CA613620, TC392778, TC420735, TC386422, TC395453, DR737360, TC388688, TC390569, TC416685, TC403872, TC389090, TC434831, TC396895, TC389375, TC426326, TC388822, TC377496, TC406708, TC382655, TC393106, TC429771, CK200433, TC423576, BQ294582, BQ607161, TC406038, TC396460, TC416658, TC428944, TC395351 |
| [Double-strand break repair via homologous recombination](http://amigo.geneontology.org/cgi-bin/amigo/go.cgi?action=query&view=query&query=GO:0000724&search_constraint=terms) | TC386040, TC402308, TC393692, TC397258, TC402603, TC379436, TC411287, TC435533, TC386519, TC374240, CV759879, CK214224, TC408907, TC391143, TC433162, TC391438, TC433589, TC386414, TC398633, TC394716, TC440636, TC395723, TC380063, TC380943, TC393830, TC386344, CK211469, TC405030, TC378054, TC381963, TC388403, TC383176, TC404371, TC389718, TC384219, TC379357, TC386279, TC385780, TC381943, TC388976, TC382342, TC373914, TC432504, TC386639, TC397676, TC402186 |
| [Recombinational repair](http://amigo.geneontology.org/cgi-bin/amigo/go.cgi?action=query&view=query&query=GO:0000725&search_constraint=terms) | TC386040, TC402308, TC393692, TC397258, TC402603, TC379436, TC411287, TC435533, TC386519, TC374240, CV759879, CK214224, TC408907, TC391143, TC433162, TC391438, TC433589, TC386414, TC398633, TC394716, TC440636, TC395723, TC380063, TC380943, TC393830, TC386344, CK211469, TC405030, TC378054, TC381963, TC388403, TC383176, TC404371, TC389718, TC384219, TC379357, TC386279, TC385780, TC381943, TC388976, TC382342, TC373914, TC432504, TC386639, TC397676, TC402186 |
| [Regulation of molecular function](http://amigo.geneontology.org/cgi-bin/amigo/go.cgi?action=query&view=query&query=GO:0065009&search_constraint=terms) | TC410352, DR739471, TC434820, TC443387, TC379711, TC392272, CV775873, TC417077, TC386313, TC398052, CA710880, TC425841, TC402440, TC387579, TC394459, TC371242, TC384738, TC423252, TC394307, TC397660, CK211707, TC375146, TC386963, TC389363, TC388914, TC388410, TC389589, TC446235, TC387410, TC392778, TC397312, TC420735, TC395453, TC386422, TC386961, TC388688, TC390569, TC416685, TC407076, TC403872, TC399919, TC407572, TC389090, TC372175, TC389375, TC388822, TC396365, TC406708, TC377496, TC419747, TC382655, TC429771, CK200433, TC423576, BQ294582, TC406038, TC416658, TC395351, TC401915, TC393561, TC393100, TC391671, TC393554, TC407614, TC382928, TC423354, DR739994, TC438243, TC384454, CA729339, TC417363, TC386237, TC393904, CK217367, TC409599, TC380882, TC423880, TC373259, TC379853, TC377410, TC381218, TC395303, TC389816, TC400755, TC405511, TC405440, TC376420, TC458987, TC377190, CA613620, TC423110, TC403588, TC401210, DR737360, TC390135, TC397729, TC377225, TC434831, TC396895, TC426326, TC381462, TC393106, TC401758, BQ607161, TC392263, TC396460, TC428944, TC389661, TC382045 |
| [Neurotransmitter transport](http://amigo.geneontology.org/cgi-bin/amigo/go.cgi?action=query&view=query&query=GO:0006836&search_constraint=terms) | TC398304, TC393100, TC433957, CK210754, TC382928, CV775873, TC441241, TC417363, TC386237, TC394916, TC386313, TC390994, TC398052, TC425841, TC378274, TC403929, TC387579, CA606693, TC394459, TC384738, CK211707, TC386963, TC388410, TC395303, TC422425, TC458562, TC400755, TC458987, TC396751, CA615187, TC386961, TC398514, TC390135, TC377225, TC411191, TC388547, TC387344, TC405784, TC399919, TC407572, TC372175, TC409650, TC396365, TC419747, TC380662, TC401758, TC398714, TC389661 |
| [Regulation of cellular macromolecule biosynthetic process](http://amigo.geneontology.org/cgi-bin/amigo/go.cgi?action=query&view=query&query=GO:2000112&search_constraint=terms) | TC392303, TC410352, TC386040, TC390393, TC397258, TC389190, TC372677, TC435533, TC374240, CA710880, TC398862, TC433162, TC387579, TC423252, TC397660, TC393814, TC389363, TC388914, TC458562, TC394716, TC398633, TC446235, TC404926, TC380063, TC402545, TC393830, TC380943, TC418414, TC380433, TC378054, TC420735, TC404371, TC395453, TC386961, TC384219, TC373615, TC392329, TC384735, TC390569, CA682223, TC416685, TC407076, TC419057, TC403872, TC389090, TC389375, TC404413, TC382655, CK200433, TC398714, TC373994, TC370114, BQ294582, TC406038, CJ550278, TC408192, TC395351, TC401915, CK210754, CA632212, DR739994, TC384454, TC411287, TC380329, TC394916, TC390994, TC398731, TC378274, TC391143, CK217367, TC409599, TC379853, TC386414, TC400755, TC395723, TC440636, TC406193, TC405440, TC403968, TC405615, TC377190, TC422348, TC385233, TC389718, TC401210, DR737360, TC379357, TC377225, TC386279, TC388049, TC381943, BQ607161, TC403977, TC406807, TC393960, TC426358, TC382045, DR739471, TC416906, TC402308, TC434820, TC443387, TC441343, TC379711, TC379903, TC392272, TC377061, TC417077, TC407340, TC417388, TC386519, TC386313, CV759879, CK208222, TC408907, TC402440, TC420579, TC391438, TC394307, TC375146, TC386963, TC389589, TC452762, TC386344, TC392778, TC397312, TC405030, TC381963, CK211589, TC388403, TC383176, TC386422, TC388688, TC419584, TC385780, TC388822, TC377496, TC388976, TC406708, TC429771, TC409459, TC423576, TC386639, TC402186, TC416658, TC374230, TC393561, TC393692, TC391671, TC393554, TC407614, TC459656, TC402603, TC441241, TC413571, TC379436, CA729339, TC460615, CK214224, TC425690, TC433589, TC377410, TC381218, TC389816, CA613620, CK211469, TC400362, TC397729, TC395872, TC434831, TC396895, TC426326, TC381462, TC418850, TC382342, TC393106, TC401758, TC373914, TC432504, TC392263, TC397676, TC396460, TC428944, TC389661 |
| [Oviposition](http://amigo.geneontology.org/cgi-bin/amigo/go.cgi?action=query&view=query&query=GO:0018991&search_constraint=terms) | TC386040, TC373613, TC402308, TC393692, TC390393, TC379903, TC397258, CA632212, TC402603, TC379436, TC411287, TC435533, TC386519, TC374240, CV759879, CK214224, TC408907, TC391143, TC433162, TC391438, TC393814, TC433589, TC386414, TC394716, TC398633, TC440636, TC395723, TC452762, TC380063, TC393830, TC380943, TC386344, CK211469, TC405030, TC378054, TC381963, TC388403, TC383176, TC404371, TC389718, TC384219, TC379357, TC386279, TC385780, TC419057, TC432185, TC381943, TC382342, TC388976, TC373914, TC432504, TC386639, CJ550278, TC397676, TC402186 |
| [Cell cycle checkpoint](http://amigo.geneontology.org/cgi-bin/amigo/go.cgi?action=query&view=query&query=GO:0000075&search_constraint=terms) | TC386040, TC402308, TC407978, TC393692, TC394796, TC397258, TC402603, TC377061, TC438243, TC379436, TC411287, TC435533, TC386519, TC374240, CV759879, CK214224, TC408907, TC391143, TC433162, TC391438, TC433589, TC386414, TC398633, TC394716, TC440636, TC395723, TC380063, TC380943, TC393830, TC386344, CK211469, TC405030, TC378054, TC381963, TC388403, TC383176, TC404371, TC389718, TC384219, TC379357, TC386279, TC385780, TC381943, TC382342, TC388976, TC373914, TC432504, TC386639, TC397676, TC402186, TC382045 |
| [Regulation of cell cycle arrest](http://amigo.geneontology.org/cgi-bin/amigo/go.cgi?action=query&view=query&query=GO:0071156&search_constraint=terms) | TC386040, TC402308, TC407978, TC393692, TC394796, TC397258, TC402603, TC377061, TC438243, TC379436, TC411287, TC435533, TC386519, TC374240, CV759879, CK214224, TC408907, TC391143, TC433162, TC391438, TC433589, TC386414, TC398633, TC394716, TC440636, TC395723, TC380063, TC380943, TC393830, TC386344, CK211469, TC405030, TC378054, TC381963, TC388403, TC383176, TC404371, TC389718, TC384219, TC379357, TC386279, TC385780, TC381943, TC382342, TC388976, TC373914, TC432504, TC386639, TC397676, TC402186, TC382045 |
| [Multicellular organismal reproductive behavior](http://amigo.geneontology.org/cgi-bin/amigo/go.cgi?action=query&view=query&query=GO:0033057&search_constraint=terms) | TC386040, TC373613, TC402308, TC393692, TC390393, TC379903, TC397258, CA632212, TC402603, TC379436, TC411287, TC435533, TC386519, TC374240, CV759879, CK214224, TC408907, TC391143, TC433162, TC391438, TC393814, TC433589, TC386414, TC394716, TC398633, TC440636, TC395723, TC452762, TC380063, TC393830, TC380943, TC386344, CK211469, TC405030, TC378054, TC381963, TC388403, TC383176, TC404371, TC389718, TC384219, TC379357, TC386279, TC385780, TC419057, TC432185, TC381943, TC382342, TC388976, TC373914, TC432504, TC386639, CJ550278, TC397676, TC402186 |
| [Reproductive behavior](http://amigo.geneontology.org/cgi-bin/amigo/go.cgi?action=query&view=query&query=GO:0019098&search_constraint=terms) | TC386040, TC373613, TC402308, TC393692, TC390393, TC379903, TC397258, CA632212, TC402603, TC379436, TC411287, TC435533, TC386519, TC374240, CV759879, CK214224, TC408907, TC391143, TC433162, TC391438, TC393814, TC433589, TC386414, TC394716, TC398633, TC440636, TC395723, TC452762, TC380063, TC393830, TC380943, TC386344, CK211469, TC405030, TC378054, TC381963, TC388403, TC383176, TC404371, TC389718, TC384219, TC379357, TC386279, TC385780, TC419057, TC432185, TC381943, TC382342, TC388976, TC373914, TC432504, TC386639, CJ550278, TC397676, TC402186 |
| [Regulation of macromolecule biosynthetic process](http://amigo.geneontology.org/cgi-bin/amigo/go.cgi?action=query&view=query&query=GO:0010556&search_constraint=terms) | TC392303, TC410352, TC386040, TC390393, TC397258, TC389190, TC372677, TC435533, TC374240, CA710880, TC398862, TC433162, TC387579, TC423252, TC397660, TC393814, TC389363, TC388914, TC458562, TC394716, TC398633, TC446235, TC404926, TC380063, TC402545, TC393830, TC380943, TC418414, TC380433, TC378054, TC420735, TC404371, TC395453, TC386961, TC384219, TC373615, TC392329, TC384735, TC390569, CA682223, TC416685, TC407076, TC419057, TC403872, TC389090, TC389375, TC404413, TC382655, CK200433, TC398714, TC373994, TC370114, BQ294582, TC406038, CJ550278, TC408192, TC395351, TC401915, CK210754, CA632212, DR739994, TC384454, TC411287, TC380329, TC394916, TC390994, TC398731, TC378274, TC391143, CK217367, TC409599, TC379853, TC386414, TC400755, TC395723, TC440636, TC406193, TC405440, TC403968, TC405615, TC376420, TC377190, TC422348, TC385233, TC389718, TC401210, DR737360, TC379357, TC377225, TC386279, TC388049, TC381943, BQ607161, TC403977, TC406807, TC393960, TC426358, TC382045, DR739471, TC416906, TC402308, TC434820, TC443387, TC441343, TC379711, TC379903, TC392272, TC377061, TC417077, TC407340, TC417388, TC386519, TC386313, CV759879, CK208222, TC408907, TC402440, TC420579, TC391438, TC394307, TC375146, TC386963, TC389589, TC452762, TC386344, TC392778, TC397312, TC405030, TC381963, CK211589, TC388403, TC383176, TC386422, TC388688, TC419584, TC385780, TC388822, TC377496, TC388976, TC406708, TC429771, TC409459, TC423576, TC386639, TC402186, TC416658, TC374230, TC393561, TC393692, TC391671, TC393554, TC407614, TC459656, TC402603, TC441241, TC413571, TC379436, CA729339, TC460615, CK214224, TC425690, TC433589, TC377410, TC381218, TC389816, CA613620, CK211469, TC400362, TC397729, TC395872, TC434831, TC396895, TC426326, TC381462, TC418850, TC382342, TC393106, TC401758, TC373914, TC432504, TC392263, TC397676, TC396460, TC428944, TC389661 |
| [Regulation of gene expression, epigenetic](http://amigo.geneontology.org/cgi-bin/amigo/go.cgi?action=query&view=query&query=GO:0040029&search_constraint=terms) | TC410352, TC401915, DR739471, TC393561, TC434820, TC443387, TC379711, TC391671, TC393554, TC407614, TC392272, DR739994, TC384454, CA729339, TC374240, CA710880, TC402440, CK217367, TC423252, TC394307, TC397660, TC375146, TC389363, TC379853, TC388914, TC389589, TC389816, TC446235, TC395723, TC405440, CA613620, TC392778, TC420735, TC386422, TC395453, DR737360, TC388688, TC390569, TC416685, TC403872, TC389090, TC434831, TC396895, TC389375, TC426326, TC388822, TC377496, TC406708, TC382655, TC393106, TC429771, CK200433, TC423576, BQ294582, BQ607161, TC406038, CJ550278, TC396460, TC416658, TC428944, TC393960, TC395351 |
| [G-protein coupled receptor protein signaling pathway](http://amigo.geneontology.org/cgi-bin/amigo/go.cgi?action=query&view=query&query=GO:0007186&search_constraint=terms) | TC410352, DR739471, TC434820, TC416906, TC443387, TC379711, TC392272, CV775873, TC377061, TC417388, TC386313, TC398052, CA710880, TC425841, TC402440, TC398862, TC387579, TC394459, TC371242, TC384738, TC423252, TC394307, TC397660, CK211707, TC375146, TC386963, TC389363, TC388914, TC388410, TC389589, TC446235, TC418414, TC392778, TC397312, TC420735, TC383909, TC449463, TC395453, TC386422, TC386961, TC388688, TC390569, TC416685, TC407076, TC403872, TC399919, TC407572, TC389090, TC372175, TC389375, TC388822, TC396365, TC406708, TC377496, TC419747, TC382655, TC429771, CK200433, TC423576, BQ294582, TC406038, TC416658, TC395351, TC390904, TC401915, TC393561, TC393100, TC391671, TC393554, TC407614, TC382928, DR739994, TC438243, TC384454, CA729339, TC417363, TC386237, TC393904, CK217367, TC379853, TC377410, TC381218, TC389816, TC395303, TC400755, TC405440, TC376420, TC458987, CA613620, TC401210, DR737360, TC390135, TC397729, TC377225, TC434831, TC432185, TC396895, TC426326, TC381462, TC393106, TC401758, BQ607161, TC396460, TC392263, TC428944, TC389661 |
| [Regulation of locomotion](http://amigo.geneontology.org/cgi-bin/amigo/go.cgi?action=query&view=query&query=GO:0040012&search_constraint=terms) | TC410352, TC401915, DR739471, TC393561, TC434820, TC443387, TC379711, TC391671, TC393554, TC407614, TC392272, DR739994, TC384454, CA729339, CA710880, TC402440, CK217367, TC423252, TC394307, TC397660, TC375146, TC389363, TC379853, TC388914, TC389589, TC389816, TC446235, TC405440, CA613620, TC392778, TC420735, TC386422, TC395453, DR737360, TC388688, TC390569, TC416685, TC403872, TC389090, TC434831, TC396895, TC389375, TC426326, TC388822, TC377496, TC406708, TC382655, TC393106, TC429771, CK200433, TC423576, BQ294582, BQ607161, TC406038, TC396460, TC416658, TC428944, TC395351, TC382045 |
| [Regulation of primary metabolic process](http://amigo.geneontology.org/cgi-bin/amigo/go.cgi?action=query&view=query&query=GO:0080090&search_constraint=terms) | TC392303, TC410352, TC386040, TC407978, TC390393, TC397258, TC389190, CV775873, TC372677, TC435533, TC374240, TC398052, CA710880, TC425841, TC398862, TC433162, TC387579, TC394459, TC371242, TC384738, TC423252, TC397660, TC393814, TC389363, TC388914, TC388410, TC458562, TC394716, TC398633, TC446235, TC404926, TC387410, TC380063, TC402545, TC393830, TC380943, TC418414, TC380433, TC378054, TC420735, TC404371, TC395453, TC386961, TC384219, TC373615, TC392329, TC384735, TC390569, CA682223, TC416685, TC407076, TC419057, TC403872, TC389090, TC389375, TC404413, TC382655, CK200433, TC398714, TC373994, TC370114, BQ294582, TC406038, CJ550278, TC408192, TC395351, TC401915, TC393100, CK210754, CA632212, DR739994, TC384454, TC411287, TC380329, TC386237, TC394916, TC393904, TC390994, TC398731, TC378274, TC391143, CK217367, TC409599, TC379853, TC395303, TC386414, TC400755, TC395723, TC440636, TC406193, TC405440, TC403968, TC405615, TC376420, TC458987, TC377190, TC423110, TC422348, TC385233, TC389718, TC401210, TC390135, DR737360, TC379357, TC377225, TC386279, TC388049, TC381943, BQ607161, TC403977, TC406807, TC393960, TC426358, TC382045, DR739471, TC416906, TC402308, TC434820, TC443387, TC441343, TC379711, TC379903, TC392272, TC377061, TC417077, TC407340, TC417388, TC386519, TC386313, CV759879, CK208222, TC408907, TC402440, TC420579, TC391438, TC394307, CK211707, TC375146, TC386963, TC389589, TC452762, TC386344, TC392778, TC397312, TC405030, TC381963, CK211589, TC388403, TC383176, TC386422, TC388688, TC419584, TC385780, TC399919, TC407572, TC372175, TC388822, TC396365, TC377496, TC388976, TC406708, TC419747, TC429771, TC409459, TC423576, TC386639, TC402186, TC416658, TC374230, TC393561, TC393692, TC391671, TC393554, TC407614, TC459656, TC382928, TC402603, TC441241, TC438243, TC413571, TC379436, CA729339, TC417363, TC460615, CK214224, TC372580, TC425690, TC433589, TC377410, TC381218, TC389816, CA613620, CK211469, TC400362, TC397729, TC395872, TC434831, TC396895, TC426326, TC381462, TC418850, TC382342, TC393106, TC401758, TC373914, TC432504, TC392263, TC397676, TC396460, TC428944, TC389661 |
| [Nerve-nerve synaptic transmission](http://amigo.geneontology.org/cgi-bin/amigo/go.cgi?action=query&view=query&query=GO:0007270&search_constraint=terms) | TC393100, TC382928, CV775873, TC417363, TC386237, TC386313, TC393904, TC398052, TC425841, TC387579, TC394459, TC384738, CK211707, TC386963, TC388410, TC377410, TC381218, TC395303, TC400755, TC458987, TC397312, TC401210, TC386961, TC390135, TC397729, TC377225, TC372175, TC407572, TC399919, TC396365, TC381462, TC419747, TC401758, TC392263, TC389661, TC382045 |
| [Synaptic transmission, glutamatergic](http://amigo.geneontology.org/cgi-bin/amigo/go.cgi?action=query&view=query&query=GO:0035249&search_constraint=terms) | TC393100, TC382928, CV775873, TC417363, TC386237, TC386313, TC393904, TC398052, TC425841, TC387579, TC394459, TC384738, CK211707, TC386963, TC388410, TC377410, TC381218, TC395303, TC400755, TC458987, TC397312, TC401210, TC386961, TC390135, TC397729, TC377225, TC372175, TC407572, TC399919, TC396365, TC381462, TC419747, TC401758, TC392263, TC389661, TC382045 |
| [DNA damage induced protein phosphorylation](http://amigo.geneontology.org/cgi-bin/amigo/go.cgi?action=query&view=query&query=GO:0006975&search_constraint=terms) | TC386040, TC402308, TC393692, CA632212, TC402603, TC411287, TC386519, TC391143, TC433162, TC391438, TC433589, TC386414, TC398633, TC394716, TC440636, TC380063, TC380943, TC393830, TC386344, CK211469, TC405030, TC378054, TC381963, TC388403, TC383176, TC404371, TC389718, TC384219, TC385780, TC419057, TC388976, TC382342, TC373914, TC397676, TC386639, TC432504, TC402186 |
| [Locomotory behavior](http://amigo.geneontology.org/cgi-bin/amigo/go.cgi?action=query&view=query&query=GO:0007626&search_constraint=terms) | TC389162, TC410352, DR739471, TC386040, TC434820, TC373613, TC402308, TC443387, TC379711, TC390393, TC379903, TC397258, TC392272, TC375864, TC435533, TC386519, TC374240, CV759879, CA710880, TC425841, TC408907, TC402440, TC433162, TC391438, TC371242, TC423252, TC394307, TC397660, TC375146, TC393814, TC389363, TC388914, TC379171, TC389589, TC394716, TC398633, TC446235, TC452762, TC380063, TC393830, TC380943, TC386344, TC392778, TC378054, TC405030, TC381963, CK211589, TC388403, TC420735, TC383176, TC404371, TC395453, TC386422, TC384219, TC388688, TC390569, TC385780, TC416685, TC419057, TC403872, TC389090, TC389375, TC388822, TC406708, TC388976, TC377496, TC382655, TC429771, CK200433, TC423576, BQ294582, TC406038, TC386639, CJ550278, TC402186, TC416658, TC395351, TC401915, TC393561, TC393692, TC391671, TC393554, TC407614, CA632212, TC402603, DR739994, TC384454, TC413571, TC379436, TC411287, CA729339, TC417363, CK214224, TC387191, TC391143, CK217367, TC433589, TC379853, TC399352, TC386414, TC389816, TC372530, TC440636, TC395723, TC405440, TC376420, CA613620, CK211469, TC385233, TC389718, DR737360, TC379357, TC387135, TC386279, TC434831, TC396895, TC432185, TC381943, TC426326, TC382342, TC393106, BQ607161, TC373914, TC432504, TC396460, TC397676, TC428944 |
| [Negative regulation of cell proliferation](http://amigo.geneontology.org/cgi-bin/amigo/go.cgi?action=query&view=query&query=GO:0008285&search_constraint=terms) | TC392303, TC398304, TC393100, TC433957, CK210754, TC382928, CV775873, TC438243, TC441241, TC386237, TC394916, TC386313, TC393904, TC398052, TC403929, TC387579, TC394459, TC384738, CK211707, TC386963, TC388410, TC377410, TC381218, TC395303, TC422425, TC458562, TC400755, TC396751, CA615187, TC388520, TC401210, TC386961, TC390135, TC397729, TC411191, TC388547, TC387344, TC405784, TC399919, TC407572, TC372175, TC381462, TC396365, TC419747, TC380662, TC401758, TC398714, TC392263, TC389661 |
| [Exocytosis](http://amigo.geneontology.org/cgi-bin/amigo/go.cgi?action=query&view=query&query=GO:0006887&search_constraint=terms) | TC398304, TC393100, TC433957, CK210754, TC378790, TC382928, CV775873, TC441241, TC417363, TC386237, TC394916, TC386313, TC390994, TC398052, TC425841, TC378274, TC403929, TC387579, CA606693, TC394459, TC384738, CK211707, TC386963, TC388410, TC395303, TC422425, TC458562, TC400755, TC458987, TC396751, CA615187, TC386961, TC398514, TC390135, TC377225, TC411191, TC388547, TC387344, TC405784, TC399919, TC407572, TC372175, TC396365, TC419747, TC380662, TC401758, TC398714, TC389661, TC382045 |
| [Regulation of metabolic process](http://amigo.geneontology.org/cgi-bin/amigo/go.cgi?action=query&view=query&query=GO:0019222&search_constraint=terms) | TC392303, TC410352, TC386040, TC407978, TC390393, TC397258, TC389190, CV775873, TC372677, TC435533, TC374240, TC398052, CA710880, TC425841, TC398862, TC433162, TC387579, TC394459, TC371242, TC384738, TC423252, TC397660, TC393814, TC389363, TC388914, TC388410, TC458562, TC394716, TC398633, TC446235, TC404926, TC387410, TC380063, TC402545, TC393830, TC380943, TC418414, TC380433, TC378054, TC420735, TC404371, TC395453, TC386961, TC384219, TC373615, TC392329, TC384735, TC390569, CA682223, TC416685, TC407076, TC419057, TC403872, TC389090, TC389375, TC404413, TC382655, CK200433, TC398714, TC373994, TC370114, BQ294582, TC406038, CJ550278, TC408192, TC395351, TC401915, TC393100, CK210754, CA632212, DR739994, TC384454, TC411287, TC380329, TC386237, TC394916, TC393904, TC390994, TC398731, TC378274, TC391143, CK217367, TC409599, TC379853, TC395303, TC386414, TC400755, TC405511, TC395723, TC440636, TC406193, TC405440, TC403968, TC405615, TC376420, TC458987, TC377190, TC423110, TC422348, TC385233, TC389718, TC401210, TC390135, DR737360, TC379357, TC377225, TC386279, TC388049, TC381943, BQ607161, TC403977, TC406807, TC393960, TC426358, TC382045, DR739471, TC416906, TC402308, TC434820, TC443387, TC441343, TC379711, TC379903, TC392272, TC377061, TC417077, TC407340, TC417388, TC386519, TC386313, CV759879, CK208222, TC408907, TC402440, TC420579, TC391438, TC394307, CK211707, TC375146, TC386963, TC389589, TC452762, TC386344, TC392778, TC397312, TC405030, TC381963, CK211589, TC388403, TC383176, TC386422, TC388688, TC419584, TC385780, TC399919, TC407572, TC372175, TC388822, TC396365, TC377496, TC388976, TC406708, TC419747, TC429771, TC423576, TC409459, TC386639, TC402186, TC416658, TC374230, TC393561, TC393692, TC391671, TC393554, TC407614, TC459656, TC382928, TC402603, TC423354, TC438243, TC441241, TC413571, TC379436, CA729339, TC417363, TC460615, CK214224, TC380882, TC423880, TC372580, TC425690, TC373259, TC433589, TC377410, TC381218, TC389816, CA613620, CK211469, TC400362, TC403588, TC397729, TC395872, TC434831, TC396895, TC426326, TC381462, TC418850, TC382342, TC393106, TC401758, TC373914, TC432504, TC392263, TC397676, TC396460, TC428944, TC389661 |
| [Regulation of gene expression](http://amigo.geneontology.org/cgi-bin/amigo/go.cgi?action=query&view=query&query=GO:0010468&search_constraint=terms) | TC392303, TC410352, TC386040, TC390393, TC397258, TC389190, TC372677, TC435533, TC374240, CA710880, TC398862, TC433162, TC387579, TC371242, TC423252, TC397660, TC393814, TC389363, TC388914, TC458562, TC394716, TC398633, TC446235, TC404926, TC380063, TC402545, TC393830, TC380943, TC418414, TC380433, TC378054, TC420735, TC404371, TC395453, TC386961, TC384219, TC373615, TC392329, TC384735, TC390569, CA682223, TC416685, TC407076, TC419057, TC403872, TC389090, TC389375, TC404413, TC382655, CK200433, TC398714, TC373994, TC370114, BQ294582, TC406038, CJ550278, TC408192, TC395351, TC401915, CK210754, CA632212, DR739994, TC384454, TC411287, TC380329, TC394916, TC390994, TC398731, TC378274, TC391143, CK217367, TC409599, TC379853, TC386414, TC400755, TC395723, TC440636, TC406193, TC405440, TC403968, TC405615, TC377190, TC422348, TC385233, TC389718, TC401210, DR737360, TC379357, TC377225, TC386279, TC388049, TC381943, BQ607161, TC403977, TC406807, TC393960, TC426358, TC382045, DR739471, TC416906, TC402308, TC434820, TC443387, TC441343, TC379711, TC379903, TC392272, TC417077, TC417388, TC386519, TC386313, CV759879, TC408907, TC402440, TC420579, TC391438, TC394307, TC375146, TC386963, TC389589, TC452762, TC386344, TC392778, TC397312, TC405030, TC381963, CK211589, TC388403, TC383176, TC386422, TC388688, TC419584, TC385780, TC388822, TC377496, TC388976, TC406708, TC429771, TC409459, TC423576, TC386639, TC402186, TC416658, TC374230, TC393561, TC393692, TC391671, TC393554, TC407614, TC459656, TC402603, TC441241, TC413571, TC379436, CA729339, TC460615, CK214224, TC425690, TC433589, TC377410, TC381218, TC389816, CA613620, CK211469, TC400362, TC397729, TC395872, TC434831, TC396895, TC426326, TC381462, TC418850, TC382342, TC393106, TC401758, TC373914, TC432504, TC392263, TC397676, TC396460, TC428944, TC389661 |
| [In utero embryonic development](http://amigo.geneontology.org/cgi-bin/amigo/go.cgi?action=query&view=query&query=GO:0001701&search_constraint=terms) | TC410352, TC401915, DR739471, TC393561, TC434820, TC443387, TC379711, TC391671, TC393554, TC407614, TC392272, DR739994, TC384454, CA729339, CA710880, TC402440, CK217367, TC423252, TC394307, TC397660, TC375146, TC389363, TC379853, TC388914, TC389589, TC389816, TC446235, TC405440, CA613620, TC392778, TC420735, TC386422, TC395453, DR737360, TC388688, TC416685, TC390569, TC403872, TC389090, TC434831, TC396895, TC389375, TC426326, TC388822, TC377496, TC406708, TC382655, TC393106, TC429771, CK200433, TC423576, BQ294582, BQ607161, TC406038, TC396460, TC416658, TC428944, TC395351 |
| [Double-strand break repair](http://amigo.geneontology.org/cgi-bin/amigo/go.cgi?action=query&view=query&query=GO:0006302&search_constraint=terms) | TC386040, TC402308, TC393692, TC397258, TC402603, TC379436, TC411287, TC435533, TC386519, TC374240, CV759879, CK214224, TC408907, TC391143, TC433162, TC391438, TC433589, TC386414, TC398633, TC394716, TC440636, TC395723, TC380063, TC380943, TC393830, TC386344, CK211469, TC405030, TC378054, TC381963, TC388403, TC383176, TC404371, TC389718, TC384219, TC379357, TC386279, TC385780, TC381943, TC388976, TC382342, TC373914, TC432504, TC386639, TC397676, TC402186 |
| [Respiratory tube development](http://amigo.geneontology.org/cgi-bin/amigo/go.cgi?action=query&view=query&query=GO:0030323&search_constraint=terms) | TC386040, TC402308, TC393692, TC390393, TC379903, TC397258, TC402603, TC435533, TC386519, CV759879, TC391143, TC433162, TC391438, TC425690, TC393814, TC433589, TC386414, TC398633, TC394716, TC440636, TC395723, TC452762, TC380063, TC380943, TC393830, TC386344, CK211469, TC405030, TC378054, TC381963, TC388403, TC383176, TC389718, TC384219, TC385780, TC419057, TC381943, TC388976, TC382342, TC373914, TC432504, TC386639, TC397676, CJ550278 |
| [Lung development](http://amigo.geneontology.org/cgi-bin/amigo/go.cgi?action=query&view=query&query=GO:0030324&search_constraint=terms) | TC386040, TC402308, TC393692, TC390393, TC379903, TC397258, TC402603, TC435533, TC386519, CV759879, TC391143, TC433162, TC391438, TC425690, TC393814, TC433589, TC386414, TC398633, TC394716, TC440636, TC395723, TC452762, TC380063, TC380943, TC393830, TC386344, CK211469, TC405030, TC378054, TC381963, TC388403, TC383176, TC389718, TC384219, TC385780, TC419057, TC381943, TC388976, TC382342, TC373914, TC432504, TC386639, TC397676, CJ550278 |
| [Regulation of cellular metabolic process](http://amigo.geneontology.org/cgi-bin/amigo/go.cgi?action=query&view=query&query=GO:0031323&search_constraint=terms) | TC392303, TC410352, TC386040, TC390393, TC397258, TC389190, CV775873, TC372677, TC435533, TC374240, TC398052, CA710880, TC425841, TC398862, TC433162, TC387579, TC394459, TC371242, TC384738, TC423252, TC397660, TC393814, TC389363, TC388914, TC388410, TC458562, TC394716, TC398633, TC446235, TC404926, TC387410, TC380063, TC402545, TC393830, TC380943, TC418414, TC380433, TC378054, TC420735, TC404371, TC395453, TC386961, TC384219, TC373615, TC392329, TC384735, TC390569, CA682223, TC416685, TC407076, TC419057, TC403872, TC389090, TC389375, TC404413, TC382655, CK200433, TC398714, TC373994, TC370114, BQ294582, TC406038, CJ550278, TC408192, TC395351, TC401915, TC393100, CK210754, CA632212, DR739994, TC384454, TC411287, TC380329, TC386237, TC394916, TC393904, TC390994, TC398731, TC378274, TC391143, CK217367, TC409599, TC379853, TC395303, TC386414, TC400755, TC395723, TC440636, TC406193, TC405440, TC403968, TC405615, TC376420, TC458987, TC377190, TC423110, TC422348, TC385233, TC389718, TC401210, TC390135, DR737360, TC379357, TC377225, TC386279, TC388049, TC381943, BQ607161, TC403977, TC406807, TC393960, TC426358, TC382045, DR739471, TC416906, TC402308, TC434820, TC443387, TC441343, TC379711, TC379903, TC392272, TC377061, TC417077, TC407340, TC417388, TC386519, TC386313, CV759879, CK208222, TC408907, TC402440, TC420579, TC391438, TC394307, CK211707, TC375146, TC386963, TC389589, TC452762, TC386344, TC392778, TC397312, TC405030, TC381963, CK211589, TC388403, TC383176, TC386422, TC388688, TC419584, TC385780, TC399919, TC407572, TC372175, TC388822, TC396365, TC377496, TC388976, TC406708, TC419747, TC429771, TC409459, TC423576, TC386639, TC402186, TC416658, TC374230, TC393561, TC393692, TC391671, TC393554, TC407614, TC459656, TC382928, TC402603, TC441241, TC438243, TC413571, TC379436, CA729339, TC417363, TC460615, CK214224, TC372580, TC425690, TC373259, TC433589, TC377410, TC381218, TC389816, CA613620, CK211469, TC400362, TC397729, TC395872, TC434831, TC396895, TC426326, TC381462, TC418850, TC382342, TC393106, TC401758, TC373914, TC432504, TC392263, TC397676, TC396460, TC428944, TC389661 |
| [Regulation of neurotransmitter secretion](http://amigo.geneontology.org/cgi-bin/amigo/go.cgi?action=query&view=query&query=GO:0046928&search_constraint=terms) | TC393100, TC382928, CV775873, TC417363, TC386237, TC386313, TC393904, TC398052, TC425841, TC387579, TC394459, TC384738, CK211707, TC386963, TC388410, TC377410, TC381218, TC395303, TC400755, TC458987, TC397312, TC401210, TC386961, TC390135, TC397729, TC377225, TC372175, TC407572, TC399919, TC396365, TC381462, TC419747, TC401758, TC392263, TC389661 |
| [Regulation of neurotransmitter transport](http://amigo.geneontology.org/cgi-bin/amigo/go.cgi?action=query&view=query&query=GO:0051588&search_constraint=terms) | TC393100, TC382928, CV775873, TC417363, TC386237, TC386313, TC393904, TC398052, TC425841, TC387579, TC394459, TC384738, CK211707, TC386963, TC388410, TC377410, TC381218, TC395303, TC400755, TC458987, TC397312, TC401210, TC386961, TC390135, TC397729, TC377225, TC372175, TC407572, TC399919, TC396365, TC381462, TC419747, TC401758, TC392263, TC389661 |
| [Inhibition of adenylate cyclase activity by metabotropic glutamate receptor signaling pathway](http://amigo.geneontology.org/cgi-bin/amigo/go.cgi?action=query&view=query&query=GO:0007196&search_constraint=terms) | TC393100, TC382928, CV775873, TC417363, TC386237, TC386313, TC393904, TC398052, TC425841, TC387579, TC394459, TC384738, CK211707, TC386963, TC388410, TC377410, TC381218, TC395303, TC400755, TC458987, TC397312, TC401210, TC386961, TC390135, TC397729, TC377225, TC372175, TC407572, TC399919, TC396365, TC381462, TC419747, TC401758, TC392263, TC389661 |
| [Glutamate signaling pathway](http://amigo.geneontology.org/cgi-bin/amigo/go.cgi?action=query&view=query&query=GO:0007215&search_constraint=terms) | TC393100, TC382928, CV775873, TC417363, TC386237, TC386313, TC393904, TC398052, TC425841, TC387579, TC394459, TC384738, CK211707, TC386963, TC388410, TC377410, TC381218, TC395303, TC400755, TC458987, TC397312, TC401210, TC386961, TC390135, TC397729, TC377225, TC372175, TC407572, TC399919, TC396365, TC381462, TC419747, TC401758, TC392263, TC389661 |
| [Metabotropic glutamate receptor signaling pathway](http://amigo.geneontology.org/cgi-bin/amigo/go.cgi?action=query&view=query&query=GO:0007216&search_constraint=terms) | TC393100, TC382928, CV775873, TC417363, TC386237, TC386313, TC393904, TC398052, TC425841, TC387579, TC394459, TC384738, CK211707, TC386963, TC388410, TC377410, TC381218, TC395303, TC400755, TC458987, TC397312, TC401210, TC386961, TC390135, TC397729, TC377225, TC372175, TC407572, TC399919, TC396365, TC381462, TC419747, TC401758, TC392263, TC389661 |
| [Regulation of secretion](http://amigo.geneontology.org/cgi-bin/amigo/go.cgi?action=query&view=query&query=GO:0051046&search_constraint=terms) | TC398304, TC393100, TC433957, CK210754, TC378790, TC382928, CV775873, TC441241, TC417363, TC386237, TC394916, TC386313, TC393904, TC390994, TC398052, TC425841, TC378274, TC403929, TC387579, CA606693, TC394459, TC384738, CK211707, TC386963, TC388410, TC377410, TC381218, TC395303, TC422425, TC458562, TC400755, TC376420, TC458987, TC396751, TC397312, CA615187, TC401210, TC386961, TC398514, TC390135, TC397729, TC377225, TC411191, TC388547, TC387344, TC407076, TC405784, TC399919, TC407572, TC372175, TC381462, TC396365, TC419747, TC380662, TC401758, TC398714, TC392263, TC389661, TC382045 |
| [Anti-apoptosis](http://amigo.geneontology.org/cgi-bin/amigo/go.cgi?action=query&view=query&query=GO:0006916&search_constraint=terms) | TC410352, DR739471, TC434820, TC443387, TC441343, TC379711, TC392272, CA710880, TC402440, TC423252, TC394307, TC397660, TC375146, TC389363, TC388914, TC389589, TC446235, TC392778, TC380433, CK211589, TC420735, TC386422, TC395453, TC388688, TC384735, TC390569, TC416685, TC403872, TC389090, TC389375, TC388822, TC406708, TC377496, TC382655, TC429771, CK200433, TC423576, BQ294582, TC370114, TC409459, TC406038, TC416658, TC395351, TC393561, TC401915, TC391671, TC393554, TC407614, TC423354, DR739994, TC384454, TC413571, CA729339, CK217367, TC380882, TC423880, TC379853, TC389816, TC405511, TC405440, TC377190, CA613620, TC400056, TC403588, TC381817, DR737360, TC396895, TC434831, TC426326, TC393106, BQ607161, TC396460, TC428944 |
| [Nucleus organization](http://amigo.geneontology.org/cgi-bin/amigo/go.cgi?action=query&view=query&query=GO:0006997&search_constraint=terms) | TC386040, TC402308, TC393692, TC390393, TC379903, TC397258, CA632212, TC402603, TC379436, TC411287, TC435533, TC386519, TC374240, CV759879, CK214224, TC408907, TC391143, TC433162, TC391438, TC393814, TC433589, TC386414, TC394716, TC398633, TC440636, TC395723, TC452762, TC380063, TC380943, TC393830, TC386344, CK211469, TC405030, TC378054, TC381963, TC388403, TC383176, TC404371, TC389718, TC384219, TC379357, TC386279, TC385780, TC419057, TC381943, TC382342, TC388976, TC373914, TC432504, TC386639, TC397676, CJ550278, TC402186, TC393970 |
| [Detection of chemical stimulus involved in sensory perception](http://amigo.geneontology.org/cgi-bin/amigo/go.cgi?action=query&view=query&query=GO:0050907&search_constraint=terms) | TC410352, DR739471, TC434820, TC416906, TC443387, TC379711, TC392272, TC417388, TC402440, TC398862, TC423252, TC394307, TC397660, TC375146, TC389363, TC388914, TC389589, TC446235, TC418414, TC392778, TC420735, TC383909, TC449463, TC386422, TC395453, TC388688, TC390569, TC416685, TC407076, TC403872, TC389090, TC389375, TC388822, TC377496, TC406708, TC382655, TC429771, CK200433, TC423576, TC406038, TC416658, TC395351, TC386646, TC393561, TC401915, TC391671, TC393554, TC407614, TC459656, DR739994, TC438243, TC384454, CA729339, CK217367, TC379853, TC389816, TC405440, DR737360, TC432185, TC396895, TC434831, TC426326, TC393106, BQ607161, TC396460, TC406807, TC428944 |
| [Detection of stimulus involved in sensory perception](http://amigo.geneontology.org/cgi-bin/amigo/go.cgi?action=query&view=query&query=GO:0050906&search_constraint=terms) | TC410352, DR739471, TC434820, TC416906, TC443387, TC379711, TC392272, TC417388, TC402440, TC398862, TC423252, TC394307, TC397660, TC375146, TC389363, TC388914, TC379171, TC389589, TC446235, TC418414, TC392778, TC420735, TC383909, TC449463, TC386422, TC395453, TC388688, TC390569, TC416685, TC407076, TC403872, TC389090, TC389375, TC388822, TC377496, TC406708, TC382655, TC429771, CK200433, TC423576, TC406038, TC416658, TC395351, TC386646, TC393561, TC401915, TC391671, TC393554, TC407614, TC459656, DR739994, TC438243, TC384454, CA729339, CK217367, TC379853, TC389816, TC405440, DR737360, TC432185, TC396895, TC434831, TC426326, TC393106, BQ607161, TC396460, TC406807, TC428944 |
| [Behavior](http://amigo.geneontology.org/cgi-bin/amigo/go.cgi?action=query&view=query&query=GO:0007610&search_constraint=terms) | TC410352, TC386040, TC373613, TC390393, TC397258, TC375864, TC435533, TC374240, CA710880, TC425841, TC433162, TC371242, TC423252, TC397660, TC393814, TC389363, TC388914, TC379171, TC394716, TC398633, TC446235, TC380063, TC393830, TC380943, TC378054, TC420735, TC404371, TC395453, TC384219, TC390569, TC416685, TC419057, TC403872, TC389090, TC389375, TC382655, CK200433, TC370114, BQ294582, TC406038, CJ550278, TC395351, TC401915, CA632212, DR739994, TC384454, TC411287, TC387191, TC391143, CK217367, TC379853, TC386414, TC440636, TC395723, TC405440, TC376420, TC385233, TC389718, DR737360, TC379357, TC387135, TC386279, TC381943, BQ607161, TC382045, TC389162, DR739471, TC402308, TC434820, TC443387, TC379711, TC379903, TC392272, TC386519, CV759879, TC408907, TC402440, TC391438, TC394307, TC375146, TC389589, TC452762, TC386344, TC392778, TC405030, TC388403, CK211589, TC381963, TC383176, TC386422, TC388688, TC385780, TC377496, TC388976, TC406708, TC388822, TC429771, TC409459, TC423576, TC386639, TC416658, TC402186, TC393561, TC393692, TC391671, TC407614, TC393554, TC402603, TC379436, TC413571, CA729339, TC417363, CK214224, TC433589, TC389816, TC399352, TC372530, CA613620, CK211469, TC432185, TC396895, TC434831, TC426326, TC382342, TC393106, TC397676, TC396460, TC432504, TC373914, TC428944 |
| [Sensory perception of chemical stimulus](http://amigo.geneontology.org/cgi-bin/amigo/go.cgi?action=query&view=query&query=GO:0007606&search_constraint=terms) | TC410352, DR739471, TC434820, TC416906, TC443387, TC379711, TC392272, TC377061, TC417388, TC402440, TC398862, TC423252, TC394307, TC397660, TC375146, TC389363, TC388914, TC389589, TC446235, TC418414, TC392778, TC420735, TC383909, TC449463, TC386422, TC395453, TC388688, TC390569, TC416685, TC407076, TC403872, TC389090, TC389375, TC388822, TC377496, TC406708, TC382655, TC429771, CK200433, TC423576, TC370114, TC409459, TC406038, TC416658, TC395351, TC386646, TC393561, TC401915, TC391671, TC393554, TC407614, TC459656, DR739994, TC438243, TC384454, CA729339, CK217367, TC379853, TC389816, TC405440, DR737360, TC432185, TC396895, TC434831, TC426326, TC393106, BQ607161, TC406807, TC396460, TC428944 |
| [Ion transport](http://amigo.geneontology.org/cgi-bin/amigo/go.cgi?action=query&view=query&query=GO:0006811&search_constraint=terms) | CA646741, TC410352, DR739471, TC404158, TC434820, TC443387, TC379711, TC373958, TC392272, TC429341, TC374240, TC397994, TC402440, TC423252, TC394307, TC397660, TC375146, TC389363, TC388914, TC373583, TC389589, TC411471, TC446235, TC392778, TC432154, CK211589, TC420735, TC386422, TC395453, TC388688, TC390569, TC416685, TC403872, TC389090, TC389375, TC388822, TC406708, TC373002, TC377496, TC382655, TC429771, CK200433, TC423576, TC406038, CJ550278, TC416658, TC395351, TC386646, TC374230, TC393561, TC401915, TC456619, TC391671, TC393554, TC407614, TC459656, DR739994, TC384454, TC413571, CA729339, CK217367, TC379853, TC370315, TC389816, TC395723, TC405440, DR737360, TC444402, TC394728, TC388049, TC396895, TC434831, TC426326, TC393106, BQ607161, TC406807, TC396460, TC428944, TC382045 |
| [Cell surface receptor linked signaling pathway](http://amigo.geneontology.org/cgi-bin/amigo/go.cgi?action=query&view=query&query=GO:0007166&search_constraint=terms) | TC410352, DR739471, TC434820, TC416906, TC443387, TC379711, TC392272, CV775873, TC377061, TC417388, TC386313, TC398052, CA710880, TC425841, TC402440, TC398862, TC387579, TC394459, TC371242, TC384738, TC423252, TC394307, TC397660, CK211707, TC375146, TC386963, TC389363, TC388914, TC388410, TC389589, TC446235, TC418414, TC392778, TC397312, TC420735, TC383909, TC449463, TC435595, TC395453, TC386422, TC386961, TC388688, TC390569, TC416685, TC407076, TC403872, TC399919, TC407572, TC389090, TC372175, TC389375, TC388822, TC396365, TC406708, TC377496, TC419747, TC382655, TC429771, CK200433, TC423576, TC370114, BQ294582, TC406038, TC416658, TC395351, TC390904, TC401915, TC393561, TC393100, TC391671, TC393554, TC407614, TC382928, DR739994, TC438243, TC384454, CA729339, TC417363, TC386237, TC393904, CK217367, TC409599, TC425690, TC379853, TC377410, TC381218, TC395303, TC389816, TC400755, TC405440, TC376420, TC458987, CA613620, TC401210, DR737360, TC390135, TC397729, TC377225, TC434831, TC432185, TC396895, TC426326, TC381462, TC393106, TC401758, BQ607161, TC396460, TC392263, TC428944, TC389661, TC382045 |
| [Regulation of macromolecule metabolic process](http://amigo.geneontology.org/cgi-bin/amigo/go.cgi?action=query&view=query&query=GO:0060255&search_constraint=terms) | TC392303, TC410352, TC386040, TC390393, TC397258, TC389190, TC372677, TC435533, TC374240, CA710880, TC398862, TC433162, TC387579, TC371242, TC423252, TC397660, TC393814, TC389363, TC388914, TC458562, TC394716, TC398633, TC446235, TC404926, TC380063, TC402545, TC393830, TC380943, TC418414, TC380433, TC378054, TC420735, TC404371, TC395453, TC386961, TC384219, TC373615, TC392329, TC384735, TC390569, CA682223, TC416685, TC407076, TC419057, TC403872, TC389090, TC389375, TC404413, TC382655, CK200433, TC398714, TC373994, TC370114, BQ294582, TC406038, CJ550278, TC408192, TC395351, TC401915, CK210754, CA632212, DR739994, TC384454, TC411287, TC380329, TC394916, TC390994, TC398731, TC378274, TC391143, CK217367, TC409599, TC379853, TC386414, TC400755, TC395723, TC440636, TC406193, TC405440, TC403968, TC405615, TC376420, TC377190, TC423110, TC422348, TC385233, TC389718, TC401210, DR737360, TC379357, TC377225, TC386279, TC388049, TC381943, BQ607161, TC403977, TC406807, TC393960, TC426358, TC382045, DR739471, TC416906, TC402308, TC434820, TC443387, TC441343, TC379711, TC379903, TC392272, TC377061, TC417077, TC407340, TC417388, TC386519, TC386313, CV759879, CK208222, TC408907, TC402440, TC420579, TC391438, TC394307, TC375146, TC386963, TC389589, TC452762, TC386344, TC392778, TC397312, TC405030, TC381963, CK211589, TC388403, TC383176, TC386422, TC388688, TC419584, TC385780, TC388822, TC377496, TC388976, TC406708, TC429771, TC409459, TC423576, TC386639, TC402186, TC416658, TC374230, TC393561, TC393692, TC391671, TC393554, TC407614, TC459656, TC402603, TC441241, TC413571, TC379436, CA729339, TC460615, CK214224, TC425690, TC433589, TC377410, TC381218, TC389816, CA613620, CK211469, TC400362, TC397729, TC395872, TC434831, TC396895, TC426326, TC381462, TC418850, TC382342, TC393106, TC401758, TC373914, TC432504, TC392263, TC397676, TC396460, TC428944, TC389661 |
| [Fertilization](http://amigo.geneontology.org/cgi-bin/amigo/go.cgi?action=query&view=query&query=GO:0009566&search_constraint=terms) | TC410352, TC401915, DR739471, TC393561, TC434820, TC443387, TC379711, TC391671, TC393554, TC407614, TC392272, DR739994, TC384454, CA729339, TC402440, CK217367, TC371242, TC423252, TC394307, TC397660, TC375146, TC389363, TC379853, TC379171, TC388914, TC389589, TC389816, TC446235, TC405440, CA613620, TC392778, TC420735, TC386422, TC395453, TC388688, TC416685, TC390569, TC403872, TC389090, TC434831, TC396895, TC389375, TC426326, TC388822, TC377496, TC406708, TC382655, TC393106, TC429771, CK200433, TC423576, BQ294582, BQ607161, TC406038, TC396460, TC416658, TC428944, TC395351 |
| [Respiratory system development](http://amigo.geneontology.org/cgi-bin/amigo/go.cgi?action=query&view=query&query=GO:0060541&search_constraint=terms) | TC386040, TC402308, TC393692, TC390393, TC379903, TC397258, TC402603, TC435533, TC386519, CV759879, TC391143, TC433162, TC391438, TC425690, TC393814, TC433589, TC386414, TC398633, TC394716, TC440636, TC395723, TC452762, TC380063, TC380943, TC393830, TC386344, CK211469, TC405030, TC378054, TC381963, TC388403, TC383176, TC389718, TC384219, TC385780, TC419057, TC381943, TC388976, TC382342, TC373914, TC432504, TC386639, TC397676, CJ550278 |
| [Post-embryonic morphogenesis](http://amigo.geneontology.org/cgi-bin/amigo/go.cgi?action=query&view=query&query=GO:0009886&search_constraint=terms) | TC410352, DR739471, TC434820, TC443387, TC379711, TC392272, TC418716, TC374240, CA710880, TC402440, TC423252, TC394307, TC397660, TC375146, TC389363, TC388914, TC389589, TC446235, TC387410, TC392778, TC420735, TC386422, TC395453, TC388688, TC390569, TC416685, TC403872, TC389090, TC389375, TC388822, TC406708, TC377496, TC382655, TC429771, CK200433, TC423576, BQ294582, TC406038, CJ550278, TC416658, TC395351, TC393561, TC401915, TC391671, TC393554, TC407614, DR739994, TC384454, CA729339, CK217367, TC379853, TC389816, TC372530, TC418685, TC395723, TC405440, CA613620, TC423110, DR737360, TC396895, TC434831, TC426326, TC393106, BQ607161, TC396460, TC428944 |
| [Growth](http://amigo.geneontology.org/cgi-bin/amigo/go.cgi?action=query&view=query&query=GO:0040007&search_constraint=terms) | TC410352, TC386040, TC407978, TC390393, TC397258, CV775873, TC451511, TC435533, TC374240, TC398052, TC387861, CA710880, TC425841, TC433162, TC387579, TC394459, TC371242, TC423252, TC397660, TC393814, TC389363, TC388914, TC379171, TC388410, TC458562, TC416442, TC394716, TC398633, TC421914, TC446235, TC387410, TC380063, TC402545, TC393830, TC380943, TC380433, TC378054, TC420735, TC404371, TC395453, TC384219, TC409843, TC384735, TC390569, TC405540, TC416685, TC387344, TC372664, TC407076, TC419057, TC403872, TC389090, TC389375, TC382655, CK200433, TC398714, TC373994, BQ294582, TC406038, CJ550278, TC393970, TC398970, TC457126, TC395351, TC403580, TC401915, TC398304, TC456619, TC393100, TC433957, CK210754, TC412569, CA632212, DR739994, TC384454, TC411287, CA598430, TC386237, TC409343, TC390994, TC387191, TC391143, CK217367, TC379853, TC370315, TC395303, TC386414, TC395723, TC440636, TC405440, TC376420, TC385233, TC389718, TC390135, DR737360, TC379357, TC387135, TC386279, TC409650, TC381943, TC380662, BQ607161, TC406807, TC372845, TC382045, DR739471, TC416906, TC402308, TC434820, TC443387, TC441343, TC379711, TC388566, TC378790, TC379903, TC390630, TC392272, TC440066, TC386519, CV759879, TC408907, TC402440, CA606693, TC391438, TC394307, CK211707, TC375146, TC389589, TC452762, TC386344, TC392778, TC396751, TC397312, TC370350, TC405030, TC381963, CK211589, TC388403, TC383176, TC370044, TC383909, TC449463, CA615187, TC386422, TC388718, TC388688, TC385780, TC388547, TC405784, TC399919, TC407572, TC372175, TC388822, TC396365, TC377496, TC388976, TC406708, TC419747, TC429771, TC423576, TC386639, TC402186, TC416658, TC393561, TC393692, TC391671, TC393554, TC407614, TC459656, TC403264, TC402603, TC441241, TC438243, TC449724, TC433557, TC413571, TC379436, CA729339, TC417363, CK214224, TC403929, TC394118, TC425690, TC433589, TC389816, TC399352, TC422425, TC372530, CA613620, TC393820, CK211469, TC411191, TC434831, TC396895, TC426326, TC375431, TC382342, TC393106, TC377749, TC401758, TC373914, TC432504, TC396460, TC397676, TC450285, TC428944, TC389661 |
| [Cation transport](http://amigo.geneontology.org/cgi-bin/amigo/go.cgi?action=query&view=query&query=GO:0006812&search_constraint=terms) | CA646741, TC410352, DR739471, TC434820, TC443387, TC379711, TC392272, TC429341, TC374240, TC397994, TC402440, TC423252, TC394307, TC397660, TC375146, TC389363, TC388914, TC373583, TC389589, TC411471, TC446235, TC392778, CK211589, TC420735, TC386422, TC395453, TC388688, TC390569, TC416685, TC403872, TC389090, TC389375, TC388822, TC377496, TC406708, TC382655, TC429771, CK200433, TC423576, TC406038, CJ550278, TC416658, TC395351, TC386646, TC374230, TC393561, TC401915, TC456619, TC391671, TC393554, TC407614, TC459656, DR739994, TC384454, TC413571, CA729339, CK217367, TC379853, TC389816, TC370315, TC395723, TC405440, DR737360, TC444402, TC394728, TC388049, TC396895, TC434831, TC426326, TC393106, BQ607161, TC406807, TC396460, TC428944, TC382045 |
| [Body morphogenesis](http://amigo.geneontology.org/cgi-bin/amigo/go.cgi?action=query&view=query&query=GO:0010171&search_constraint=terms) | TC410352, TC401915, DR739471, TC393561, TC434820, TC443387, TC379711, TC391671, TC393554, TC407614, TC392272, DR739994, TC384454, CA729339, TC374240, CA710880, TC402440, CK217367, TC423252, TC394307, TC397660, TC375146, TC389363, TC388914, TC379853, TC389589, TC389816, TC372530, TC446235, TC395723, TC405440, TC387410, CA613620, TC392778, TC420735, TC386422, TC395453, DR737360, TC388688, TC390569, TC416685, TC403872, TC389090, TC434831, TC396895, TC389375, TC426326, TC388822, TC377496, TC406708, TC382655, TC393106, TC429771, CK200433, TC423576, BQ294582, BQ607161, TC406038, CJ550278, TC396460, TC416658, TC428944, TC395351 |
| [Post-embryonic body morphogenesis](http://amigo.geneontology.org/cgi-bin/amigo/go.cgi?action=query&view=query&query=GO:0040032&search_constraint=terms) | TC410352, TC401915, DR739471, TC393561, TC434820, TC443387, TC379711, TC391671, TC393554, TC407614, TC392272, DR739994, TC384454, CA729339, TC374240, CA710880, TC402440, CK217367, TC423252, TC394307, TC397660, TC375146, TC389363, TC388914, TC379853, TC389589, TC389816, TC372530, TC446235, TC395723, TC405440, TC387410, CA613620, TC392778, TC420735, TC386422, TC395453, DR737360, TC388688, TC390569, TC416685, TC403872, TC389090, TC434831, TC396895, TC389375, TC426326, TC388822, TC377496, TC406708, TC382655, TC393106, TC429771, CK200433, TC423576, BQ294582, BQ607161, TC406038, CJ550278, TC396460, TC416658, TC428944, TC395351 |
| [Negative regulation of apoptosis](http://amigo.geneontology.org/cgi-bin/amigo/go.cgi?action=query&view=query&query=GO:0043066&search_constraint=terms) | TC410352, DR739471, TC434820, TC443387, TC441343, TC379711, TC392272, CA710880, TC402440, TC423252, TC394307, TC397660, TC375146, TC389363, TC388914, TC389589, TC446235, TC392778, TC380433, CK211589, TC420735, TC386422, TC395453, TC388688, TC384735, TC390569, TC416685, TC403872, TC389090, TC389375, TC388822, TC406708, TC377496, TC382655, TC429771, CK200433, TC423576, BQ294582, TC370114, TC409459, TC406038, TC416658, TC395351, TC393561, TC401915, TC391671, TC393554, TC407614, TC423354, DR739994, TC384454, TC413571, CA729339, CK217367, TC380882, TC423880, TC379853, TC389816, TC405511, TC405440, TC377190, CA613620, TC400056, TC403588, TC381817, DR737360, TC396895, TC434831, TC426326, TC393106, BQ607161, TC396460, TC428944, TC382045 |
| [Negative regulation of programmed cell death](http://amigo.geneontology.org/cgi-bin/amigo/go.cgi?action=query&view=query&query=GO:0043069&search_constraint=terms) | TC410352, DR739471, TC434820, TC443387, TC441343, TC379711, TC392272, CA710880, TC402440, TC423252, TC394307, TC397660, TC375146, TC389363, TC388914, TC389589, TC446235, TC392778, TC380433, CK211589, TC420735, TC386422, TC395453, TC388688, TC384735, TC390569, TC416685, TC403872, TC389090, TC389375, TC388822, TC406708, TC377496, TC382655, TC429771, CK200433, TC423576, BQ294582, TC370114, TC409459, TC406038, TC416658, TC395351, TC393561, TC401915, TC391671, TC393554, TC407614, TC423354, DR739994, TC384454, TC413571, CA729339, CK217367, TC380882, TC423880, TC379853, TC389816, TC405511, TC405440, TC377190, CA613620, TC400056, TC403588, TC381817, DR737360, TC396895, TC434831, TC426326, TC393106, BQ607161, TC396460, TC428944, TC382045 |
| [Negative regulation of cell death](http://amigo.geneontology.org/cgi-bin/amigo/go.cgi?action=query&view=query&query=GO:0060548&search_constraint=terms) | TC410352, DR739471, TC434820, TC443387, TC441343, TC379711, TC392272, CA710880, TC402440, TC423252, TC394307, TC397660, TC375146, TC389363, TC388914, TC389589, TC446235, TC392778, TC380433, CK211589, TC420735, TC386422, TC395453, TC388688, TC384735, TC390569, TC416685, TC403872, TC389090, TC389375, TC388822, TC406708, TC377496, TC382655, TC429771, CK200433, TC423576, BQ294582, TC370114, TC409459, TC406038, TC416658, TC395351, TC393561, TC401915, TC391671, TC393554, TC407614, TC423354, DR739994, TC384454, TC413571, CA729339, CK217367, TC380882, TC423880, TC379853, TC389816, TC405511, TC405440, TC377190, CA613620, TC400056, TC403588, TC381817, DR737360, TC396895, TC434831, TC426326, TC393106, BQ607161, TC396460, TC428944, TC382045 |
| [Immune response](http://amigo.geneontology.org/cgi-bin/amigo/go.cgi?action=query&view=query&query=GO:0006955&search_constraint=terms) | TC392303, TC398304, TC393100, TC433957, CK210754, TC382928, CV775873, TC377061, TC441241, TC386237, TC394916, TC386313, TC393904, TC398052, TC403929, TC387579, TC394459, TC384738, CK211707, TC425690, TC386963, TC434396, TC388410, TC377410, TC381218, TC395303, TC398592, TC422425, TC458562, TC400755, TC396751, CA615187, TC388520, TC401210, TC386961, TC386707, TC390135, TC405041, TC397729, TC411191, TC388547, TC387344, TC405784, TC399919, TC407572, TC372175, TC381462, TC396365, TC419747, TC380662, TC401758, TC398714, TC392263, TC389661 |
| [Nematode larval development](http://amigo.geneontology.org/cgi-bin/amigo/go.cgi?action=query&view=query&query=GO:0002119&search_constraint=terms) | TC410352, TC386040, TC390393, TC397258, CV775873, TC451511, TC435533, TC374240, TC398052, TC387861, CA710880, TC425841, TC433162, TC387579, TC394459, TC371242, TC423252, TC397660, TC393814, TC389363, TC388914, TC379171, TC388410, TC458562, TC416442, TC394716, TC398633, TC421914, TC446235, TC387410, TC380063, TC402545, TC393830, TC380943, TC380433, TC378054, TC420735, TC404371, TC395453, TC384219, TC409843, TC384735, TC390569, TC405540, TC416685, TC387344, TC372664, TC407076, TC419057, TC403872, TC389090, TC389375, TC382655, CK200433, TC398714, TC373994, BQ294582, TC406038, CJ550278, TC398970, TC395351, TC403580, TC401915, TC398304, TC456619, TC393100, TC433957, CK210754, TC412569, CA632212, DR739994, TC384454, TC411287, CA598430, TC386237, TC409343, TC390994, TC387191, TC391143, CK217367, TC379853, TC395303, TC386414, TC395723, TC440636, TC405440, TC376420, TC385233, TC389718, TC390135, DR737360, TC379357, TC387135, TC386279, TC409650, TC381943, TC380662, BQ607161, TC406807, TC372845, DR739471, TC416906, TC402308, TC434820, TC443387, TC441343, TC379711, TC388566, TC379903, TC378790, TC390630, TC392272, TC440066, TC386519, CV759879, TC408907, TC402440, CA606693, TC391438, TC394307, CK211707, TC375146, TC389589, TC452762, TC386344, TC392778, TC396751, TC397312, TC405030, TC381963, CK211589, TC388403, TC383176, TC370044, TC383909, TC449463, CA615187, TC386422, TC388688, TC385780, TC388547, TC405784, TC399919, TC407572, TC372175, TC388822, TC396365, TC377496, TC388976, TC406708, TC419747, TC429771, TC423576, TC386639, TC402186, TC416658, TC393561, TC393948, TC393692, TC391671, TC393554, TC407614, TC459656, TC403264, TC402603, TC441241, TC449724, TC413571, TC379436, CA729339, TC417363, CK214224, TC403929, TC394118, TC425690, TC433589, TC389816, TC399352, TC422425, CA613620, TC393820, CK211469, TC411191, TC434831, TC396895, TC426326, TC375431, TC382342, TC393106, TC401758, TC373914, TC432504, TC397676, TC396460, TC450285, TC428944, TC389661 |
| [Larval development](http://amigo.geneontology.org/cgi-bin/amigo/go.cgi?action=query&view=query&query=GO:0002164&search_constraint=terms) | TC410352, TC386040, TC390393, TC397258, CV775873, TC451511, TC435533, TC374240, TC398052, TC387861, CA710880, TC425841, TC433162, TC387579, TC394459, TC371242, TC423252, TC397660, TC393814, TC389363, TC388914, TC379171, TC388410, TC458562, TC416442, TC394716, TC398633, TC421914, TC446235, TC387410, TC380063, TC402545, TC393830, TC380943, TC380433, TC378054, TC420735, TC404371, TC395453, TC384219, TC409843, TC384735, TC390569, TC405540, TC416685, TC387344, TC372664, TC407076, TC419057, TC403872, TC389090, TC389375, TC382655, CK200433, TC398714, TC373994, BQ294582, TC406038, CJ550278, TC398970, TC395351, TC403580, TC401915, TC398304, TC456619, TC393100, TC433957, CK210754, TC412569, CA632212, DR739994, TC384454, TC411287, CA598430, TC386237, TC409343, TC390994, TC387191, TC391143, CK217367, TC379853, TC395303, TC386414, TC395723, TC440636, TC405440, TC376420, TC385233, TC389718, TC390135, DR737360, TC379357, TC387135, TC386279, TC409650, TC381943, TC380662, BQ607161, TC406807, TC372845, DR739471, TC416906, TC402308, TC434820, TC443387, TC441343, TC379711, TC388566, TC379903, TC378790, TC390630, TC392272, TC440066, TC386519, CV759879, TC408907, TC402440, CA606693, TC391438, TC394307, CK211707, TC375146, TC389589, TC452762, TC386344, TC392778, TC396751, TC397312, TC405030, TC381963, CK211589, TC388403, TC383176, TC370044, TC383909, TC449463, CA615187, TC386422, TC388688, TC385780, TC388547, TC405784, TC399919, TC407572, TC372175, TC388822, TC396365, TC377496, TC388976, TC406708, TC419747, TC429771, TC423576, TC386639, TC402186, TC416658, TC393561, TC393948, TC393692, TC391671, TC393554, TC407614, TC459656, TC403264, TC402603, TC441241, TC449724, TC413571, TC379436, CA729339, TC417363, CK214224, TC403929, TC394118, TC425690, TC433589, TC389816, TC399352, TC422425, CA613620, TC393820, CK211469, TC411191, TC434831, TC396895, TC426326, TC375431, TC382342, TC393106, TC401758, TC373914, TC432504, TC397676, TC396460, TC450285, TC428944, TC389661 |
| [Regulation of cellular localization](http://amigo.geneontology.org/cgi-bin/amigo/go.cgi?action=query&view=query&query=GO:0060341&search_constraint=terms) | TC398304, TC393100, TC433957, CK210754, TC378790, TC382928, CV775873, TC441241, TC417363, TC386237, TC394916, TC386313, TC393904, TC390994, TC398052, TC425841, TC378274, TC403929, TC387579, CA606693, TC394459, TC384738, CK211707, TC386963, TC388410, TC377410, TC381218, TC395303, TC422425, TC458562, TC400755, TC376420, TC458987, TC396751, TC397312, CA615187, TC401210, TC386961, TC398514, TC390135, TC397729, TC377225, TC411191, TC388547, TC387344, TC407076, TC405784, TC399919, TC407572, TC372175, TC381462, TC396365, TC419747, TC380662, TC401758, TC398714, TC392263, TC389661, TC382045 |
| [Regulation of cellular process](http://amigo.geneontology.org/cgi-bin/amigo/go.cgi?action=query&view=query&query=GO:0050794&search_constraint=terms) | TC410352, TC386040, TC407978, TC390393, TC397258, TC389190, CV775873, TC398052, CA710880, TC398862, TC433162, TC394459, TC371242, TC384738, TC423252, TC397660, TC389363, TC388410, TC458562, TC421914, TC387410, TC380063, TC418414, TC378054, TC395453, TC392329, TC384735, TC390569, CA682223, TC407076, TC419057, TC403872, TC398714, BQ294582, CJ550278, TC403580, TC401915, TC398304, TC393100, TC433957, CA632212, DR739994, TC394916, TC398731, TC378274, CK217367, TC391128, TC409599, TC379853, TC370315, TC395723, TC405440, TC403968, TC379536, TC385233, TC389718, TC398514, TC377225, TC381943, BQ607161, TC403977, TC406807, TC382045, TC404606, CA646741, TC416906, TC402308, TC434820, TC441343, TC379711, TC378790, TC379903, TC392272, TC377061, TC417077, TC417388, TC386313, CV759879, TC413339, CK208222, TC408907, CA606693, TC420579, TC391438, CK211707, TC386963, TC421345, TC392778, TC396751, TC397312, TC405030, CK211589, TC388403, CA615187, TC405041, TC419584, TC385780, TC405784, TC399919, TC407572, TC372175, TC406708, TC419747, TC409459, TC402186, TC386646, TC423354, TC402603, TC441241, TC449724, TC413571, CA729339, CK214224, BE585841, TC425690, TC381218, TC422425, CA613620, TC400362, TC388520, TC395872, TC411191, TC434831, TC432185, TC426326, TC381462, TC373914, TC392263, TC392303, TC394796, TC372677, TC435533, TC374240, TC425841, TC387579, TC388914, TC393814, TC398633, TC394716, TC404926, TC446235, TC378271, TC402545, TC380943, TC393830, TC380433, TC420735, TC404371, TC373615, TC384219, TC386961, TC398606, TC397562, TC416685, TC387344, TC389090, TC389375, TC404413, TC382655, TC370114, TC373994, CK200433, TC406038, TC408192, TC393970, TC395351, CK210754, TC384454, TC380329, TC411287, TC386237, TC390994, TC393904, TC391143, TC386414, TC395303, TC400755, TC440636, TC405511, TC406193, TC405615, TC458987, TC376420, TC377190, TC423110, TC391613, TC422348, TC401210, DR737360, TC379357, TC390135, TC386279, TC388049, TC380662, TC393960, TC426358, DR739471, TC443387, TC407340, TC386519, TC402440, TC394307, TC375146, TC389589, TC398592, TC452762, TC405475, TC386344, TC381963, TC383176, TC386422, TC388688, TC388547, TC377496, TC388976, TC396365, TC388822, TC379069, TC429771, TC423576, TC386639, TC416658, TC381957, TC374230, TC393561, TC393692, TC391671, TC407614, TC393554, TC459656, TC382928, TC438243, TC379436, TC460615, TC417363, TC403929, TC387710, TC394118, TC423880, TC380882, TC373259, TC372580, TC433589, TC377410, TC389816, TC372530, TC400056, TC393820, TC461622, CK211469, TC381817, TC403588, TC397729, TC396895, TC391122, TC418850, TC382342, TC393106, TC401758, TC397676, TC396460, TC432504, TC389661, TC428944 |
| [Regulation of exocytosis](http://amigo.geneontology.org/cgi-bin/amigo/go.cgi?action=query&view=query&query=GO:0017157&search_constraint=terms) | TC398304, TC393100, TC433957, CK210754, TC382928, CV775873, TC441241, TC417363, TC386237, TC394916, TC386313, TC390994, TC398052, TC425841, TC378274, TC403929, TC387579, CA606693, TC394459, TC384738, CK211707, TC386963, TC388410, TC395303, TC422425, TC458562, TC400755, TC376420, TC458987, TC396751, CA615187, TC386961, TC398514, TC390135, TC377225, TC411191, TC388547, TC387344, TC405784, TC407076, TC399919, TC407572, TC372175, TC396365, TC419747, TC380662, TC401758, TC398714, TC389661, TC382045 |
| [Embryonic cleavage](http://amigo.geneontology.org/cgi-bin/amigo/go.cgi?action=query&view=query&query=GO:0040016&search_constraint=terms) | TC386040, TC402308, TC393692, TC390393, TC379903, TC397258, TC390630, CA632212, TC402603, TC377061, TC379436, TC411287, TC435533, TC386519, TC374240, CV759879, CK214224, TC408907, TC391143, TC433162, TC391438, TC371242, TC393814, TC433589, TC379171, TC386414, TC394716, TC398633, TC440636, TC395723, TC452762, TC380063, TC393830, TC380943, TC386344, CK211469, TC405030, TC378054, TC381963, TC388403, TC383176, TC404371, TC389718, TC384219, TC379357, TC386279, TC385780, TC419057, TC381943, TC382342, TC388976, TC373914, TC432504, TC386639, CJ550278, TC397676, TC402186 |
| [Monovalent inorganic cation transport](http://amigo.geneontology.org/cgi-bin/amigo/go.cgi?action=query&view=query&query=GO:0015672&search_constraint=terms) | TC410352, DR739471, TC434820, TC443387, TC379711, TC392272, TC402440, TC423252, TC394307, TC397660, TC375146, TC389363, TC388914, TC389589, TC411471, TC446235, TC392778, CK211589, TC420735, TC386422, TC395453, TC388688, TC390569, TC416685, TC403872, TC389090, TC389375, TC388822, TC377496, TC406708, TC382655, TC429771, CK200433, TC423576, TC406038, TC416658, TC395351, TC386646, TC374230, TC393561, TC401915, TC456619, TC391671, TC393554, TC407614, TC459656, DR739994, TC384454, TC413571, CA729339, CK217367, TC379853, TC389816, TC370315, TC405440, DR737360, TC388049, TC396895, TC434831, TC426326, TC393106, BQ607161, TC396460, TC406807, TC428944, TC382045 |
| [Response to heat](http://amigo.geneontology.org/cgi-bin/amigo/go.cgi?action=query&view=query&query=GO:0009408&search_constraint=terms) | TC410352, DR739471, TC434820, TC443387, TC379711, TC392272, TC402440, TC423252, TC394307, TC397660, TC375146, TC389363, TC388914, TC389589, TC446235, TC392778, TC420735, TC397964, TC386422, TC395453, TC388688, TC405041, TC390569, TC416685, TC407076, TC403872, TC389090, TC389375, TC388822, TC377496, TC406708, TC382655, TC429771, CK200433, TC423576, TC406038, TC416658, TC395351, TC386646, TC393561, TC401915, TC391671, TC393554, TC407614, TC459656, TC423354, DR739994, TC384454, CA729339, TC409599, CK217367, TC380882, TC423880, TC379853, TC382742, TC389816, TC405511, TC405440, TC383677, TC461622, TC403588, DR737360, TC396895, TC434831, TC426326, TC393106, BQ607161, TC406807, TC396460, TC428944 |
| [Neuroprotection](http://amigo.geneontology.org/cgi-bin/amigo/go.cgi?action=query&view=query&query=GO:0043526&search_constraint=terms) | TC393100, TC382928, CV775873, TC417363, TC386237, TC386313, TC393904, TC398052, TC425841, TC387579, TC394459, TC384738, CK211707, TC386963, TC388410, TC377410, TC381218, TC395303, TC400755, TC376420, TC458987, TC380433, TC397312, TC401210, TC386961, TC390135, TC397729, TC377225, TC372175, TC407572, TC399919, TC396365, TC381462, TC419747, TC401758, TC392263, TC389661 |
| [Chordate embryonic development](http://amigo.geneontology.org/cgi-bin/amigo/go.cgi?action=query&view=query&query=GO:0043009&search_constraint=terms) | TC410352, TC401915, DR739471, TC393561, TC434820, TC443387, TC379711, TC391671, TC393554, TC407614, TC392272, DR739994, TC384454, TC417077, CA729339, CA710880, TC402440, CK217367, TC423252, TC394307, TC397660, TC375146, TC389363, TC388914, TC379853, TC389589, TC389816, TC446235, TC405440, CA613620, TC392778, TC420735, TC386422, TC395453, DR737360, TC388688, TC390569, TC416685, TC403872, TC389090, TC434831, TC396895, TC389375, TC426326, TC388822, TC377496, TC406708, TC382655, TC393106, TC429771, CK200433, TC423576, BQ294582, TC370114, BQ607161, TC409459, TC406038, TC396460, TC416658, TC428944, TC395351 |
| [Interphase](http://amigo.geneontology.org/cgi-bin/amigo/go.cgi?action=query&view=query&query=GO:0051325&search_constraint=terms) | CA646741, TC394716, TC374230, TC395723, TC440636, TC402308, TC393692, CK211469, TC400362, TC405030, TC388403, TC389718, TC407340, TC384219, TC411287, TC379357, TC460615, TC386279, TC374240, CK214224, TC394820, TC419057, TC408907, TC391438, TC404413, TC370114, TC386639, CJ550278, TC433589, TC402186, TC393960, TC382045 |
| [Regulation of multicellular organismal process](http://amigo.geneontology.org/cgi-bin/amigo/go.cgi?action=query&view=query&query=GO:0051239&search_constraint=terms) | TC410352, DR739471, TC434820, TC443387, TC441343, TC379711, TC378790, TC392272, CV775873, TC374240, TC386313, TC398052, CA710880, TC425841, TC402440, TC387579, TC394459, TC384738, TC423252, TC394307, TC397660, CK211707, TC375146, TC386963, TC389363, TC388914, TC388410, TC389589, TC421914, TC446235, TC387410, TC378271, TC392778, TC397312, CK211589, TC420735, TC383909, TC449463, TC395453, TC386422, TC386961, TC388688, TC383701, TC384735, TC390569, TC416685, TC407076, TC403872, TC399919, TC407572, TC389090, TC372175, TC389375, TC388822, TC396365, TC406708, TC377496, TC419747, TC382655, TC429771, CK200433, TC423576, TC409459, TC370114, BQ294582, TC406038, CJ550278, TC416658, TC395351, TC401915, TC393561, TC393100, TC369928, TC391671, TC393554, TC407614, TC382928, DR739994, TC449724, TC384454, TC413571, CA729339, TC417363, TC386237, TC393904, TC387710, CK217367, TC394118, TC380882, TC425690, TC379853, TC377410, TC381218, TC395303, TC389816, TC400755, TC372530, TC395723, TC405440, TC376420, TC458987, TC461622, CA613620, TC393820, TC403588, TC401210, TC375798, DR737360, TC390135, TC397729, TC377225, TC434831, TC396895, TC426326, TC381462, TC418850, TC393106, TC401758, BQ607161, TC396460, TC392263, TC428944, TC389661, TC404606, TC382045 |
| [Generation of a signal involved in cell-cell signaling](http://amigo.geneontology.org/cgi-bin/amigo/go.cgi?action=query&view=query&query=GO:0003001&search_constraint=terms) | TC398304, TC393100, TC433957, CK210754, TC382928, CV775873, TC441241, TC417363, TC386237, TC394916, TC386313, TC390994, TC398052, TC425841, TC378274, TC403929, TC387579, CA606693, TC394459, TC384738, CK211707, TC386963, TC388410, TC395303, TC422425, TC458562, TC400755, TC376420, TC458987, TC396751, CA615187, TC386961, TC398514, TC390135, TC377225, TC411191, TC388547, TC387344, TC405784, TC407076, TC399919, TC407572, TC372175, TC409650, TC396365, TC419747, TC380662, TC401758, TC398714, TC389661 |
| [Signal release](http://amigo.geneontology.org/cgi-bin/amigo/go.cgi?action=query&view=query&query=GO:0023061&search_constraint=terms) | TC398304, TC393100, TC433957, CK210754, TC382928, CV775873, TC441241, TC417363, TC386237, TC394916, TC386313, TC390994, TC398052, TC425841, TC378274, TC403929, TC387579, CA606693, TC394459, TC384738, CK211707, TC386963, TC388410, TC395303, TC422425, TC458562, TC400755, TC376420, TC458987, TC396751, CA615187, TC386961, TC398514, TC390135, TC377225, TC411191, TC388547, TC387344, TC405784, TC407076, TC399919, TC407572, TC372175, TC409650, TC396365, TC419747, TC380662, TC401758, TC398714, TC389661 |
| [Response to stimulus](http://amigo.geneontology.org/cgi-bin/amigo/go.cgi?action=query&view=query&query=GO:0050896&search_constraint=terms) | TC410352, TC386040, TC373613, TC407978, TC390393, TC416529, TC397258, CV775873, TC391946, TC396230, TC375864, TC416069, BQ838511, TC398052, CA710880, TC398862, TC433162, TC376351, TC394459, TC371242, TC416154, TC384738, TC423252, TC397660, TC404978, TC389363, TC379171, TC388410, TC416493, TC458562, TC416442, TC387410, TC380063, TC398538, TC433844, TC418414, TC388950, CJ944525, TC410063, TC378054, TC373702, TC395453, TC410194, TC392329, TC390569, TC407076, TC419057, TC445939, TC403872, TC398714, BQ294582, CJ550278, TC398970, TC401915, GH732878, TC397444, TC398304, TC393100, TC433957, CV771134, TC417012, TC423239, CA632212, TC393523, DR739994, TC394916, TC394820, TC406236, TC378274, TC391130, CK217367, TC391128, TC409599, TC446038, TC379853, TC370315, CK214702, TC418685, TC395723, TC405440, TC403968, CK213497, TC385233, TC391742, TC389718, TC386707, TC377225, TC392033, CK201148, TC381943, TC412520, TC413392, TC418928, BQ607161, TC406807, TC372845, TC382045, TC369628, TC434820, TC416906, TC402308, TC379711, TC378790, TC379903, TC377308, TC423265, TC392272, TC377061, TC401244, DR739350, TC417388, TC386313, CV759879, TC408907, CA606693, TC420579, TC391438, CK211707, TC412317, TC386963, TC412732, TC405695, TC392778, TC396751, TC397312, TC405030, TC370350, CK211589, TC388403, TC383909, TC373678, CA615187, TC416695, TC405041, TC406469, TC385780, TC405784, TC399919, TC407572, TC372175, TC369199, TC410690, TC406708, TC419747, TC409459, TC402186, TC386646, TC405356, TC423354, TC402603, TC403264, TC441241, TC369348, TC449724, TC413571, TC397909, TC413043, CA729339, TC374268, CK214224, TC408299, TC376263, TC429374, TC409187, TC384010, TC389993, TC416169, TC425690, TC381218, TC399352, TC422425, TC383677, CA613620, TC388520, TC411191, TC399342, TC434831, TC432185, TC426326, TC381462, TC377749, TC455736, TC373914, TC392263, TC450285, TC392303, TC404158, TC394796, TC397286, TC435533, TC374240, TC425841, TC424204, TC387579, TC388914, TC393814, TC398633, TC394716, TC460689, TC446235, TC380943, TC393830, TC380433, TC432154, TC403885, TC420735, TC404371, TC397964, TC427405, TC384219, TC386961, TC416685, TC387344, TC389090, TC412483, TC389375, TC382655, CK200433, TC370114, TC406038, TC393970, TC395351, TC459245, TC418032, TC456619, CK210754, TC369928, TC384454, TC411908, TC411287, TC380329, TC386237, TC393904, TC390994, TC387191, TC391143, TC434396, TC382742, TC398536, TC386414, TC395303, TC400755, TC403986, TC440636, TC405511, TC376420, TC458987, TC377190, TC385365, TC430544, TC370633, TC401210, DR737360, TC379357, TC390135, TC387135, TC386279, TC411684, TC382786, TC439225, TC380662, TC393960, TC430561, TC389162, TC382830, DR739471, TC402534, TC443387, CK212850, TC415083, TC390630, TC399636, TC418716, TC406584, TC386519, TC374726, TC402440, TC417992, TC394307, TC375146, TC398592, TC389589, TC452762, TC386344, TC381963, TC383176, TC370044, TC449463, TC386422, TC374164, TC385701, TC388688, TC384071, TC452503, TC388547, TC412150, TC388822, TC396365, TC377496, TC388976, TC429771, TC423576, TC386639, TC416658, TC379965, TC393561, TC391913, TC375834, TC393948, TC393692, TC375726, TC391671, TC393554, TC407614, TC400477, TC373787, TC397415, TC459656, TC382928, TC438243, TC379436, TC371008, TC417363, TC411941, TC403929, TC394118, TC380882, TC423880, TC379635, TC433589, TC377410, TC389816, TC372530, TC392323, TC413066, TC393820, TC461622, CK211469, CB307332, TC404842, TC403588, TC376933, TC397729, DR740372, TC403803, TC396895, TC440526, TC382342, TC393106, TC401758, TC432504, TC397676, TC396460, TC428944, TC381279, TC389661 |
| [S phase](http://amigo.geneontology.org/cgi-bin/amigo/go.cgi?action=query&view=query&query=GO:0051320&search_constraint=terms) | TC394716, TC395723, TC440636, TC402308, TC393692, CK211469, TC405030, TC388403, TC389718, TC384219, TC411287, TC379357, TC386279, CK214224, TC419057, TC408907, TC391438, TC386639, CJ550278, TC433589, TC402186, TC382045 |
| [Synaptic transmission](http://amigo.geneontology.org/cgi-bin/amigo/go.cgi?action=query&view=query&query=GO:0007268&search_constraint=terms) | TC398304, TC393100, TC433957, CK210754, TC382928, CV775873, TC441241, TC413571, TC417363, TC386237, TC394916, TC386313, TC393904, TC390994, TC398052, TC425841, TC378274, TC403929, TC387579, CA606693, TC394459, TC384738, CK211707, TC386963, TC388410, TC377410, TC381218, TC395303, TC422425, TC458562, TC400755, TC458987, TC396751, TC397312, CK211589, CA615187, TC401210, TC386961, TC398514, TC390135, TC397729, TC377225, TC411191, TC388547, TC387344, TC407076, TC405784, TC399919, TC407572, TC372175, TC409650, TC381462, TC396365, TC419747, TC380662, TC401758, TC398714, TC392263, TC393970, TC389661, TC382045 |
| [Regulation of growth rate](http://amigo.geneontology.org/cgi-bin/amigo/go.cgi?action=query&view=query&query=GO:0040009&search_constraint=terms) | TC410352, TC386040, TC373613, TC390393, TC397258, TC375864, TC445166, TC435533, TC374240, TC387861, CA710880, TC425841, TC433162, TC371242, TC423252, TC397660, TC393814, TC389363, TC388914, TC394716, TC398633, TC421914, TC446235, TC380063, TC402545, TC393830, TC380943, TC378054, TC420735, TC404371, TC395453, TC384219, TC390569, TC416685, TC372664, TC419057, TC403872, TC389090, TC389375, TC382655, CK200433, TC373994, BQ294582, TC406038, CJ550278, TC393970, TC395351, TC403580, TC401915, CA632212, DR739994, TC384454, TC411287, TC387191, TC391143, CK217367, TC379853, TC386414, TC440636, TC395723, TC405440, TC376420, TC389718, DR737360, TC379357, TC386279, TC381943, BQ607161, TC372845, DR739471, TC416906, TC402308, TC434820, TC443387, TC379711, TC379903, TC392272, TC417077, TC440066, TC386519, CV759879, TC408907, TC402440, TC391438, TC417341, TC394307, TC375146, TC389589, TC452762, TC386344, TC392778, TC405030, TC388403, CK211589, TC381963, TC383176, TC386422, TC388688, TC385780, TC388822, TC377496, TC388976, TC406708, TC429771, TC423576, TC386639, TC416658, TC402186, TC393561, TC393692, TC391671, TC393554, TC407614, TC402603, TC413571, TC379436, CA729339, TC417363, CK214224, TC394118, TC433589, TC389816, TC399352, CA613620, CK211469, TC432185, TC396895, TC434831, TC426326, TC382342, TC393106, TC397676, TC396460, TC432504, TC373914, TC428944 |
| [Positive regulation of growth rate](http://amigo.geneontology.org/cgi-bin/amigo/go.cgi?action=query&view=query&query=GO:0040010&search_constraint=terms) | TC410352, TC386040, TC373613, TC390393, TC397258, TC375864, TC445166, TC435533, TC374240, TC387861, CA710880, TC425841, TC433162, TC371242, TC423252, TC397660, TC393814, TC389363, TC388914, TC394716, TC398633, TC421914, TC446235, TC380063, TC402545, TC393830, TC380943, TC378054, TC420735, TC404371, TC395453, TC384219, TC390569, TC416685, TC372664, TC419057, TC403872, TC389090, TC389375, TC382655, CK200433, TC373994, BQ294582, TC406038, CJ550278, TC393970, TC395351, TC403580, TC401915, CA632212, DR739994, TC384454, TC411287, TC387191, TC391143, CK217367, TC379853, TC386414, TC440636, TC395723, TC405440, TC376420, TC389718, DR737360, TC379357, TC386279, TC381943, BQ607161, TC372845, DR739471, TC416906, TC402308, TC434820, TC443387, TC379711, TC379903, TC392272, TC417077, TC440066, TC386519, CV759879, TC408907, TC402440, TC391438, TC417341, TC394307, TC375146, TC389589, TC452762, TC386344, TC392778, TC405030, TC388403, CK211589, TC381963, TC383176, TC386422, TC388688, TC385780, TC388822, TC377496, TC388976, TC406708, TC429771, TC423576, TC386639, TC416658, TC402186, TC393561, TC393692, TC391671, TC393554, TC407614, TC402603, TC413571, TC379436, CA729339, TC417363, CK214224, TC394118, TC433589, TC389816, TC399352, CA613620, CK211469, TC432185, TC396895, TC434831, TC426326, TC382342, TC393106, TC397676, TC396460, TC432504, TC373914, TC428944 |
| [Tube development](http://amigo.geneontology.org/cgi-bin/amigo/go.cgi?action=query&view=query&query=GO:0035295&search_constraint=terms) | TC386040, TC402308, TC393948, TC393692, TC390393, TC379903, TC397258, TC390630, TC402603, TC435533, TC386519, CV759879, TC391143, TC433162, TC391438, TC380882, TC425690, TC393814, TC433589, TC386414, TC398633, TC394716, TC440636, TC395723, TC452762, TC380063, TC380943, TC393830, TC461622, TC386344, CK211469, TC370350, TC405030, TC378054, TC381963, TC388403, TC383176, TC403588, TC389718, TC384219, TC385780, TC419057, TC381943, TC382342, TC388976, TC373914, TC432504, TC386639, TC397676, CJ550278 |
| [Regulation of transport](http://amigo.geneontology.org/cgi-bin/amigo/go.cgi?action=query&view=query&query=GO:0051049&search_constraint=terms) | TC398304, TC393100, TC433957, CK210754, TC378790, TC382928, CV775873, TC441241, TC417363, TC386237, TC394916, TC386313, TC393904, TC390994, TC398052, TC425841, TC378274, TC403929, TC387579, CA606693, TC394459, TC384738, CK211707, TC425690, TC386963, TC388410, TC377410, TC381218, TC395303, TC422425, TC458562, TC400755, TC372530, TC376420, TC458987, TC396751, TC397312, CA615187, TC401210, TC386961, TC398514, TC390135, TC397729, TC377225, TC411191, TC388547, TC387344, TC407076, TC405784, TC399919, TC407572, TC372175, TC381462, TC396365, TC419747, TC380662, TC401758, TC398714, TC392263, TC389661, TC382045 |
| [Detection of chemical stimulus](http://amigo.geneontology.org/cgi-bin/amigo/go.cgi?action=query&view=query&query=GO:0009593&search_constraint=terms) | TC410352, DR739471, TC434820, TC416906, TC443387, TC379711, TC392272, TC417388, TC402440, TC398862, TC423252, TC394307, TC397660, TC375146, TC389363, TC388914, TC389589, TC446235, TC418414, TC392778, TC420735, TC383909, TC449463, TC386422, TC395453, TC388688, TC405041, TC390569, TC416685, TC407076, TC403872, TC389090, TC389375, TC388822, TC377496, TC406708, TC382655, TC429771, CK200433, TC423576, TC406038, TC393970, TC416658, TC395351, TC386646, TC393561, TC401915, TC391671, TC393554, TC407614, TC459656, DR739994, TC438243, TC384454, CA729339, CK217367, TC379853, TC389816, TC405440, DR737360, TC432185, TC396895, TC434831, TC426326, TC393106, BQ607161, TC396460, TC406807, TC428944 |
| [Positive regulation of multicellular organism growth](http://amigo.geneontology.org/cgi-bin/amigo/go.cgi?action=query&view=query&query=GO:0040018&search_constraint=terms) | TC410352, DR739471, TC434820, TC443387, TC379711, TC378790, TC392272, CA710880, TC402440, TC423252, TC394307, TC397660, TC375146, TC389363, TC388914, TC389589, TC421914, TC446235, TC392778, TC420735, TC383909, TC449463, TC386422, TC395453, TC388688, TC390569, TC416685, TC407076, TC403872, TC389090, TC389375, TC388822, TC377496, TC406708, TC382655, TC429771, CK200433, TC423576, BQ294582, TC406038, TC416658, TC395351, TC393561, TC401915, TC391671, TC407614, TC393554, DR739994, TC449724, TC384454, CA729339, CK217367, TC379853, TC389816, TC372530, TC405440, CA613620, TC393820, DR737360, TC396895, TC434831, TC426326, TC393106, BQ607161, TC396460, TC428944 |
| [Post-embryonic development](http://amigo.geneontology.org/cgi-bin/amigo/go.cgi?action=query&view=query&query=GO:0009791&search_constraint=terms) | TC410352, TC386040, TC390393, TC397258, CV775873, TC397286, TC451511, TC435533, TC374240, TC398052, TC387861, CA710880, TC425841, TC433162, TC387579, TC394459, TC371242, TC423252, TC397660, TC393814, TC389363, TC388914, TC379171, TC388410, TC458562, TC416442, TC394716, TC398633, TC421914, TC446235, TC387410, TC380063, TC402545, TC393830, TC380943, TC380433, TC378054, TC420735, TC404371, TC395453, TC384219, TC409843, TC384735, TC390569, TC405540, TC416685, TC387344, TC372664, TC407076, TC419057, TC403872, TC389090, TC389375, TC382655, CK200433, TC398714, TC373994, BQ294582, TC406038, CJ550278, TC398970, TC395351, TC403580, TC401915, TC398304, TC456619, TC393100, TC433957, CK210754, TC412569, CA632212, DR739994, TC384454, TC411287, CA598430, TC386237, TC409343, TC390994, TC387191, TC391143, CK217367, TC380416, TC423804, TC379853, TC395303, TC386414, TC418685, TC395723, TC440636, TC405440, TC376420, TC423110, TC385233, TC389718, TC390135, DR737360, TC379357, TC387135, TC386279, TC409650, TC381943, TC380662, BQ607161, TC406807, TC372845, DR739471, TC416906, TC402308, TC434820, TC443387, TC441343, TC379711, TC388566, TC378790, TC379903, TC390630, TC392272, TC390285, TC418716, TC440066, TC386519, CV759879, TC408907, TC402440, CA606693, TC391438, TC394307, CK211707, TC375146, TC389589, TC452762, TC386344, TC392778, TC396751, TC397312, TC405030, TC381963, CK211589, TC388403, TC383176, TC370044, TC383909, TC449463, CA615187, TC386422, TC403328, TC388688, TC383701, TC400181, TC385780, TC388547, TC405784, TC399919, TC407572, TC372175, TC388822, TC396365, TC377496, TC388976, TC406708, TC419747, TC429771, TC423576, TC386639, TC402186, TC416658, TC393561, TC375834, TC393948, TC393692, TC391671, TC393554, TC407614, TC459656, TC403264, TC402603, TC441241, TC449724, TC413571, TC379436, CA729339, TC417363, CK214224, TC403929, TC394118, TC425690, TC433589, TC389816, TC399352, TC422425, TC372530, CA613620, TC393820, CK211469, TC369092, TC416981, TC411191, TC434831, TC396895, TC426326, TC375431, TC382342, TC393106, TC401758, TC373914, TC432504, TC396460, TC397676, TC450285, TC428944, TC389661, TC396529 |
| [S phase-specific histone modification](http://amigo.geneontology.org/cgi-bin/amigo/go.cgi?action=query&view=query&query=GO:0006324&search_constraint=terms) | TC394716, TC395723, TC440636, TC402308, TC393692, CK211469, TC405030, TC388403, TC389718, TC384219, TC411287, TC379357, TC386279, CK214224, TC419057, TC408907, TC391438, TC386639, CJ550278, TC433589, TC402186 |
| [Cellular macromolecule catabolic process](http://amigo.geneontology.org/cgi-bin/amigo/go.cgi?action=query&view=query&query=GO:0044265&search_constraint=terms) | TC410352, TC386040, TC390393, TC397258, TC375864, TC420420, TC435533, TC374240, CA710880, TC398862, TC433162, TC423252, TC397660, TC393814, TC389363, TC388914, TC394716, TC398633, TC446235, TC380063, TC393830, TC380943, TC418414, TC378054, TC420735, TC404371, TC395453, TC384219, TC397562, TC390569, TC416685, TC403872, TC389090, TC389375, TC404413, TC382655, CK200433, TC373791, TC370114, TC406038, TC395351, TC401915, DR739994, TC384454, TC411287, TC394820, TC391143, CK217367, TC409599, TC379853, TC386414, TC440636, TC395723, TC405440, TC377190, TC423110, TC389718, DR737360, TC379357, TC386279, TC376774, TC420394, TC381943, BQ607161, TC393960, TC404606, DR739471, TC416906, TC402308, TC434820, TC443387, TC379711, TC379903, TC392272, TC417388, TC386519, CV759879, TC408907, TC402440, TC391438, TC394307, TC375146, TC389589, TC452762, TC386344, TC392778, TC405030, TC388403, TC381963, TC383176, TC386422, TC388688, TC400181, BJ317882, TC385780, TC377496, TC388976, TC406708, TC388822, TC429771, TC409459, TC423576, TC386639, TC416658, TC402186, TC393561, TC393692, TC391671, TC393554, TC407614, TC402603, TC449724, TC379436, TC391007, CA729339, CK214224, TC387710, TC433589, TC389816, CA613620, TC393820, CK211469, TC400362, TC396895, TC434831, TC426326, TC382342, TC393106, TC397676, TC396460, TC432504, TC373914, TC428944 |
| [Positive regulation of growth](http://amigo.geneontology.org/cgi-bin/amigo/go.cgi?action=query&view=query&query=GO:0045927&search_constraint=terms) | TC410352, TC386040, TC373613, TC390393, TC397258, TC375864, TC445166, TC435533, TC374240, TC387861, CA710880, TC425841, TC433162, TC371242, TC423252, TC397660, TC393814, TC389363, TC388914, TC394716, TC398633, TC421914, TC446235, TC380063, TC402545, TC393830, TC380943, TC378054, TC420735, TC404371, TC395453, TC384219, TC390569, TC416685, TC372664, TC407076, TC419057, TC403872, TC389090, TC389375, TC382655, CK200433, TC373994, BQ294582, TC406038, CJ550278, TC393970, TC395351, TC403580, TC401915, CA632212, DR739994, TC384454, TC411287, TC387191, TC391143, CK217367, TC379853, TC386414, TC440636, TC395723, TC405440, TC376420, TC389718, DR737360, TC379357, TC386279, TC381943, BQ607161, TC372845, DR739471, TC416906, TC402308, TC434820, TC443387, TC379711, TC379903, TC378790, TC392272, TC417077, TC440066, TC386519, CV759879, TC408907, TC402440, TC391438, TC417341, TC394307, TC375146, TC389589, TC452762, TC386344, TC392778, TC405030, TC381963, CK211589, TC388403, TC383176, TC383909, TC449463, TC386422, TC388688, TC385780, TC388822, TC377496, TC388976, TC406708, TC429771, TC423576, TC386639, TC416658, TC402186, TC393561, TC393692, TC391671, TC393554, TC407614, TC402603, TC438243, TC449724, TC413571, TC379436, CA729339, TC417363, CK214224, TC394118, TC433589, TC389816, TC399352, TC372530, CA613620, TC393820, CK211469, TC432185, TC396895, TC434831, TC426326, TC382342, TC393106, TC373914, TC397676, TC396460, TC432504, TC428944 |
| [Regulation of cell cycle](http://amigo.geneontology.org/cgi-bin/amigo/go.cgi?action=query&view=query&query=GO:0051726&search_constraint=terms) | TC374230, TC386040, TC402308, TC407978, TC393692, TC394796, TC397258, TC402603, TC377061, TC438243, TC379436, TC407340, TC411287, TC435533, TC386519, TC374240, CV759879, CK214224, TC408907, TC391143, TC433162, TC409599, TC391438, TC373259, TC433589, TC386414, TC394716, TC398633, TC440636, TC395723, TC380063, TC405475, TC380943, TC393830, TC423110, TC386344, CK211469, TC391613, TC400362, TC405030, TC378054, TC381963, TC388403, TC383176, TC404371, TC389718, TC384219, TC379357, TC397562, TC386279, TC385780, TC381943, TC404413, TC382342, TC388976, TC379069, TC373914, TC432504, TC386639, TC397676, TC402186, TC393970, TC393960, TC382045 |
| [Detection of stimulus](http://amigo.geneontology.org/cgi-bin/amigo/go.cgi?action=query&view=query&query=GO:0051606&search_constraint=terms) | TC410352, DR739471, TC434820, TC416906, TC443387, TC379711, TC392272, TC417388, TC402440, TC398862, TC423252, TC394307, TC397660, TC375146, TC389363, TC388914, TC379171, TC389589, TC446235, TC418414, TC392778, TC420735, TC383909, TC449463, TC386422, TC395453, TC388688, TC405041, TC390569, TC416685, TC407076, TC403872, TC389090, TC389375, TC388822, TC377496, TC406708, TC382655, TC429771, CK200433, TC423576, TC406038, TC393970, TC416658, TC395351, TC386646, TC393561, TC401915, TC391671, TC393554, TC407614, TC459656, DR739994, TC438243, TC384454, CA729339, CK217367, TC379853, TC389816, TC405440, DR737360, TC432185, TC396895, TC434831, TC426326, TC393106, BQ607161, TC406807, TC396460, TC428944 |
| [Transmission of nerve impulse](http://amigo.geneontology.org/cgi-bin/amigo/go.cgi?action=query&view=query&query=GO:0019226&search_constraint=terms) | CV775873, TC374240, TC386313, TC398052, TC425841, TC387579, CA606693, TC394459, TC384738, CK211707, TC386963, TC388410, TC458562, TC421914, TC378271, TC396751, TC397312, CK211589, CA615187, TC386961, TC388547, TC387344, TC407076, TC405784, TC399919, TC407572, TC372175, TC396365, TC419747, TC398714, CJ550278, TC393970, TC398304, TC433957, TC393100, CK210754, TC382928, TC441241, TC413571, TC386237, TC417363, TC394916, TC390994, TC393904, TC378274, TC403929, TC377410, TC381218, TC395303, TC422425, TC400755, TC395723, TC458987, TC401210, TC390135, TC398514, TC397729, TC377225, TC411191, TC409650, TC381462, TC380662, TC401758, TC392263, TC389661, TC382045 |
| [Negative regulation of catalytic activity](http://amigo.geneontology.org/cgi-bin/amigo/go.cgi?action=query&view=query&query=GO:0043086&search_constraint=terms) | TC393100, TC423354, TC382928, CV775873, TC417363, TC386237, TC386313, TC393904, TC398052, TC425841, TC387579, TC380882, TC423880, TC371242, TC394459, TC384738, CK211707, TC386963, TC388410, TC377410, TC381218, TC395303, TC400755, TC405511, TC458987, TC397312, TC403588, TC401210, TC386961, TC390135, TC397729, TC377225, TC399919, TC407572, TC372175, TC396365, TC381462, TC419747, TC401758, TC392263, TC389661 |
| [Negative regulation of molecular function](http://amigo.geneontology.org/cgi-bin/amigo/go.cgi?action=query&view=query&query=GO:0044092&search_constraint=terms) | TC393100, TC423354, TC382928, CV775873, TC417363, TC386237, TC386313, TC393904, TC398052, TC425841, TC387579, TC380882, TC423880, TC371242, TC394459, TC384738, CK211707, TC386963, TC388410, TC377410, TC381218, TC395303, TC400755, TC405511, TC458987, TC397312, TC403588, TC401210, TC386961, TC390135, TC397729, TC377225, TC399919, TC407572, TC372175, TC396365, TC381462, TC419747, TC401758, TC392263, TC389661 |
| [Positive regulation of catalytic activity](http://amigo.geneontology.org/cgi-bin/amigo/go.cgi?action=query&view=query&query=GO:0043085&search_constraint=terms) | TC410352, DR739471, TC434820, TC443387, TC379711, TC392272, TC417077, CA710880, TC402440, TC423252, TC394307, TC397660, TC375146, TC389363, TC388914, TC389589, TC446235, TC387410, TC392778, TC420735, TC386422, TC395453, TC388688, TC390569, TC416685, TC407076, TC403872, TC389090, TC389375, TC388822, TC377496, TC406708, TC382655, TC429771, CK200433, TC423576, BQ294582, TC406038, TC416658, TC395351, TC393561, TC401915, TC391671, TC393554, TC407614, DR739994, TC384454, CA729339, TC409599, CK217367, TC373259, TC379853, TC389816, TC405440, TC376420, TC377190, CA613620, TC423110, DR737360, TC396895, TC434831, TC426326, TC393106, BQ607161, TC396460, TC428944, TC382045 |
| [Macromolecule catabolic process](http://amigo.geneontology.org/cgi-bin/amigo/go.cgi?action=query&view=query&query=GO:0009057&search_constraint=terms) | TC410352, TC386040, TC390393, TC397258, TC375864, TC420420, TC435533, TC374240, CA710880, TC398862, TC433162, TC423252, TC397660, TC393814, TC389363, TC388914, TC394716, TC398633, TC446235, TC380063, TC393830, TC380943, TC418414, TC378054, TC420735, TC404371, TC395453, TC384219, TC397562, TC390569, TC416685, TC403872, TC389090, TC389375, TC404413, TC382655, CK200433, TC373791, TC370114, TC406038, TC393970, TC395351, TC401915, TC393523, DR739994, TC384454, TC411287, TC394820, TC391143, CK217367, TC409599, TC379853, TC386414, TC440636, TC395723, TC405440, TC377190, TC423110, TC389718, DR737360, TC379357, TC386279, TC376774, TC420394, CK201148, TC381943, BQ607161, TC393960, TC382045, TC404606, DR739471, TC416906, TC402308, TC434820, TC443387, TC379711, TC377308, TC379903, TC392272, TC417388, TC386519, CV759879, TC408907, TC402440, TC391438, TC394307, TC375146, TC389589, TC452762, TC386344, TC392778, TC405030, TC381963, TC388403, TC383176, TC386422, TC388688, BJ317882, TC400181, TC385780, TC369199, TC388822, TC377496, TC388976, TC406708, TC429771, TC409459, TC423576, TC386639, TC416658, TC402186, TC393561, TC393692, TC391671, TC393554, TC373787, TC407614, TC402603, TC449724, TC379436, TC391007, CA729339, CK214224, TC387710, TC384010, TC433589, TC389816, CA613620, TC393820, CK211469, TC400362, DR740372, TC396895, TC434831, TC426326, TC382342, TC393106, TC373914, TC397676, TC396460, TC432504, TC428944 |
| [Regulation of vesicle-mediated transport](http://amigo.geneontology.org/cgi-bin/amigo/go.cgi?action=query&view=query&query=GO:0060627&search_constraint=terms) | TC398304, TC393100, TC433957, CK210754, TC382928, CV775873, TC441241, TC417363, TC386237, TC394916, TC386313, TC390994, TC398052, TC425841, TC378274, TC403929, TC387579, CA606693, TC394459, TC384738, CK211707, TC425690, TC386963, TC388410, TC395303, TC422425, TC458562, TC400755, TC372530, TC376420, TC458987, TC396751, CA615187, TC386961, TC398514, TC390135, TC377225, TC411191, TC388547, TC387344, TC407076, TC405784, TC399919, TC407572, TC372175, TC396365, TC419747, TC380662, TC401758, TC398714, TC389661, TC382045 |
| [Positive regulation of multicellular organismal process](http://amigo.geneontology.org/cgi-bin/amigo/go.cgi?action=query&view=query&query=GO:0051240&search_constraint=terms) | TC410352, DR739471, TC434820, TC443387, TC379711, TC378790, TC392272, CA710880, TC402440, TC423252, TC394307, TC397660, TC375146, TC389363, TC388914, TC389589, TC421914, TC446235, TC392778, TC420735, TC383909, TC449463, TC386422, TC395453, TC388688, TC390569, TC416685, TC407076, TC403872, TC389090, TC389375, TC388822, TC377496, TC406708, TC382655, TC429771, CK200433, TC423576, BQ294582, TC406038, TC416658, TC395351, TC393561, TC401915, TC391671, TC407614, TC393554, DR739994, TC449724, TC384454, CA729339, CK217367, TC379853, TC389816, TC372530, TC405440, CA613620, TC393820, DR737360, TC396895, TC434831, TC426326, TC393106, BQ607161, TC396460, TC428944 |
| [Regulation of multicellular organism growth](http://amigo.geneontology.org/cgi-bin/amigo/go.cgi?action=query&view=query&query=GO:0040014&search_constraint=terms) | TC410352, DR739471, TC434820, TC443387, TC379711, TC378790, TC392272, CA710880, TC402440, TC423252, TC394307, TC397660, TC375146, TC389363, TC388914, TC389589, TC421914, TC446235, TC392778, TC420735, TC383909, TC449463, TC386422, TC395453, TC388688, TC390569, TC416685, TC407076, TC403872, TC389090, TC389375, TC388822, TC377496, TC406708, TC382655, TC429771, CK200433, TC423576, BQ294582, TC406038, TC416658, TC395351, TC393561, TC401915, TC391671, TC407614, TC393554, DR739994, TC449724, TC384454, CA729339, CK217367, TC379853, TC389816, TC372530, TC405440, CA613620, TC393820, DR737360, TC396895, TC434831, TC426326, TC393106, BQ607161, TC396460, TC428944 |
| [Regulation of growth](http://amigo.geneontology.org/cgi-bin/amigo/go.cgi?action=query&view=query&query=GO:0040008&search_constraint=terms) | TC410352, TC386040, TC373613, TC390393, TC397258, TC375864, TC445166, TC435533, TC374240, TC387861, CA710880, TC425841, TC433162, TC371242, TC423252, TC397660, TC393814, TC389363, TC388914, TC394716, TC398633, TC421914, TC446235, TC380063, TC402545, TC378271, TC393830, TC380943, TC378054, TC420735, TC404371, TC395453, TC384219, TC390569, TC416685, TC372664, TC407076, TC419057, TC403872, TC389090, TC389375, TC382655, CK200433, TC373994, BQ294582, TC406038, CJ550278, TC393970, TC395351, TC403580, TC401915, CA632212, DR739994, TC384454, TC411287, TC387191, TC391143, CK217367, TC379853, TC386414, TC440636, TC395723, TC405440, TC376420, TC389718, DR737360, TC379357, TC386279, TC381943, BQ607161, TC372845, DR739471, TC416906, TC402308, TC434820, TC443387, TC379711, TC379903, TC378790, TC392272, TC417077, TC440066, TC386519, CV759879, TC408907, TC402440, TC391438, TC417341, TC394307, TC375146, TC389589, TC452762, TC386344, TC392778, TC405030, TC381963, CK211589, TC388403, TC383176, TC383909, TC449463, TC386422, TC388688, TC385780, TC388822, TC377496, TC388976, TC406708, TC429771, TC423576, TC386639, TC416658, TC402186, TC393561, TC393692, TC391671, TC393554, TC407614, TC402603, TC438243, TC449724, TC413571, TC379436, CA729339, TC417363, CK214224, TC394118, TC433589, TC389816, TC399352, TC372530, CA613620, TC393820, CK211469, TC375798, TC432185, TC396895, TC434831, TC426326, TC382342, TC393106, TC373914, TC397676, TC396460, TC432504, TC428944 |
| [Cellular catabolic process](http://amigo.geneontology.org/cgi-bin/amigo/go.cgi?action=query&view=query&query=GO:0044248&search_constraint=terms) | TC410352, TC386040, TC390393, TC397258, TC375864, TC420420, TC435533, TC374240, CA710880, TC457112, TC398862, TC433162, TC423252, TC397660, TC393814, TC389363, TC388914, TC394716, TC398633, TC446235, TC380063, TC433844, TC393830, TC380943, TC369064, TC418414, CJ944525, TC378054, TC420735, TC404371, TC395453, TC384219, TC397562, TC390569, TC416685, TC403872, TC389090, TC389375, TC404413, TC382655, CK200433, TC373791, TC370114, TC406038, TC393970, TC395351, TC401915, GH732878, DR739994, TC384454, TC411287, TC394820, TC391143, CK217367, TC409599, TC380416, TC379853, TC386414, TC440636, TC395723, TC405440, TC377190, TC423110, TC389718, DR737360, TC379357, TC386279, TC376774, TC420394, CK197833, TC381943, BQ607161, TC406807, TC393960, TC372845, TC382045, TC404606, DR739471, TC416906, TC402308, TC434820, TC443387, TC379711, TC379903, TC392272, TC417388, TC386519, CV759879, TC408907, TC402440, TC391438, TC394307, TC375146, TC454407, TC389589, TC452762, TC386344, TC392778, TC370350, TC405030, TC381963, TC388403, TC383176, TC386422, TC388688, BJ317882, TC400181, TC385780, TC388822, TC377496, TC388976, TC406708, TC429771, TC409459, TC423576, TC386639, TC402186, TC416658, TC393561, TC393692, TC391671, TC393554, TC400477, TC407614, TC402603, TC459656, TC449724, TC379436, TC391007, CA729339, CK214224, TC387710, TC372580, TC433589, TC389816, CA613620, TC393820, CK211469, TC400362, TC396895, TC434831, TC426326, TC382342, TC393106, TC373914, TC432504, TC397676, TC396460, TC431198, TC428944 |
| [Regulation of neuron apoptosis](http://amigo.geneontology.org/cgi-bin/amigo/go.cgi?action=query&view=query&query=GO:0043523&search_constraint=terms) | TC393100, TC382928, CV775873, TC417363, TC386237, TC386313, TC393904, TC398052, TC425841, TC387579, TC394459, TC384738, CK211707, TC386963, TC388410, TC377410, TC381218, TC395303, TC400755, TC376420, TC458987, TC380433, TC397312, TC401210, TC386961, TC390135, TC397729, TC377225, TC399919, TC372175, TC407572, TC396365, TC381462, TC419747, TC401758, TC392263, TC389661, TC382045 |
| [Regulation of localization](http://amigo.geneontology.org/cgi-bin/amigo/go.cgi?action=query&view=query&query=GO:0032879&search_constraint=terms) | TC398304, TC393100, TC433957, CK210754, TC378790, TC382928, CV775873, TC441241, TC417363, TC386237, TC394916, TC386313, TC393904, TC390994, TC398052, TC425841, TC378274, TC403929, TC387579, CA606693, TC394459, TC384738, CK211707, TC425690, TC386963, TC388410, TC377410, TC381218, TC395303, TC422425, TC458562, TC400755, TC372530, TC376420, TC458987, TC396751, TC397312, CA615187, TC401210, TC386961, TC398514, TC390135, TC397729, TC377225, TC411191, TC388547, TC387344, TC407076, TC405784, TC399919, TC407572, TC372175, TC381462, TC396365, TC419747, TC380662, TC401758, TC398714, TC392263, TC389661, TC382045 |
| [Secretion by cell](http://amigo.geneontology.org/cgi-bin/amigo/go.cgi?action=query&view=query&query=GO:0032940&search_constraint=terms) | TC398304, TC393100, TC433957, CK210754, TC378790, TC382928, CV775873, TC441241, TC449724, TC417363, TC386237, TC394916, TC386313, TC390994, TC398052, TC425841, TC378274, TC403929, TC387710, TC387579, CA606693, TC394459, TC384738, CK211707, TC425690, TC386963, TC388410, TC395303, TC422425, TC458562, TC400755, TC376420, TC458987, TC393820, TC396751, CA615187, TC386961, TC398514, TC390135, TC377225, TC411191, TC388547, TC387344, TC407076, TC405784, TC399919, TC407572, TC372175, TC409650, TC396365, TC419747, TC380662, TC401758, TC398714, TC389661, TC382045, TC404606 |
| [Cell division](http://amigo.geneontology.org/cgi-bin/amigo/go.cgi?action=query&view=query&query=GO:0051301&search_constraint=terms) | TC386040, TC402308, TC393948, TC393692, TC390393, TC394796, TC379903, TC397258, TC390630, CA632212, TC402603, TC377061, TC379436, TC411287, TC435533, TC386519, TC374240, CV759879, CK214224, TC408907, TC391143, TC433162, TC391438, TC371242, TC393814, TC433589, TC379171, TC386414, TC394716, TC398633, TC440636, TC395723, TC452762, TC380063, TC393830, TC380943, TC386344, CK211469, TC378054, TC405030, TC381963, TC388403, TC383176, TC404371, TC389718, TC384219, TC379357, TC386279, TC385780, TC419057, TC381943, TC382342, TC388976, TC415501, TC373914, TC432504, TC386639, CJ550278, TC397676, TC402186, TC393970, TC382045 |
| [Cellular membrane fusion](http://amigo.geneontology.org/cgi-bin/amigo/go.cgi?action=query&view=query&query=GO:0006944&search_constraint=terms) | TC398304, TC393100, TC433957, CK210754, TC378790, TC382928, CV775873, TC441241, TC417363, TC386237, TC394916, TC386313, TC390994, TC398052, TC425841, TC378274, TC403929, TC387579, CA606693, TC394459, TC384738, CK211707, TC386963, TC388410, TC395303, TC422425, TC458562, TC400755, TC408312, TC372530, TC458987, TC396751, CA615187, TC386961, TC398514, TC390135, TC377225, TC411191, TC388547, TC387344, TC405784, TC399919, TC407572, TC372175, TC409650, TC396365, TC419747, TC380662, TC401758, TC398714, TC393970, TC389661 |
| [Membrane fusion](http://amigo.geneontology.org/cgi-bin/amigo/go.cgi?action=query&view=query&query=GO:0061025&search_constraint=terms) | TC398304, TC393100, TC433957, CK210754, TC378790, TC382928, CV775873, TC441241, TC417363, TC386237, TC394916, TC386313, TC390994, TC398052, TC425841, TC378274, TC403929, TC387579, CA606693, TC394459, TC384738, CK211707, TC386963, TC388410, TC395303, TC422425, TC458562, TC400755, TC408312, TC372530, TC458987, TC396751, CA615187, TC386961, TC398514, TC390135, TC377225, TC411191, TC388547, TC387344, TC405784, TC399919, TC407572, TC372175, TC409650, TC396365, TC419747, TC380662, TC401758, TC398714, TC393970, TC389661 |
| [Embryo development ending in birth or egg hatching](http://amigo.geneontology.org/cgi-bin/amigo/go.cgi?action=query&view=query&query=GO:0009792&search_constraint=terms) | TC410352, TC386040, TC373613, TC390393, TC394796, TC397258, CV775873, TC375864, TC451511, TC435533, TC374240, TC398052, TC387861, CA710880, TC425841, TC433162, TC387579, TC394459, TC371242, TC423252, TC397660, TC393814, TC389363, TC388914, TC379171, TC388410, TC458562, TC416442, TC394716, TC398633, TC421914, TC446235, TC387410, TC380063, TC402545, TC393830, TC380943, TC380433, TC378054, TC420735, TC404371, TC395453, TC384219, TC409843, TC390569, TC405540, TC416685, TC387344, TC372664, TC407076, TC419057, TC403872, TC389090, TC389375, TC382655, CK200433, TC398714, TC373994, BQ294582, TC370114, TC406038, CJ550278, TC393970, TC398970, TC395351, TC403580, TC401915, TC398304, TC456619, TC393100, TC433957, CK210754, TC412569, CA632212, DR739994, TC384454, TC411287, CA598430, TC386237, TC409343, TC390994, TC387191, TC391143, CK217367, TC391128, TC379853, TC395303, TC386414, TC395723, TC440636, TC405440, TC376420, TC385233, TC389718, TC390135, DR737360, TC379357, TC387135, TC386279, TC409650, TC381943, TC380662, BQ607161, TC406807, TC372845, TC389162, DR739471, TC416906, TC402308, TC434820, TC443387, TC379711, TC388566, TC378790, TC379903, TC390630, TC392272, TC377061, TC417077, TC440066, TC386519, CV759879, TC408907, TC402440, CA606693, TC391438, TC394307, CK211707, TC375146, TC389589, TC452762, TC386344, TC392778, TC396751, TC397312, TC405030, TC381963, CK211589, TC388403, TC383176, TC370044, TC383909, TC449463, CA615187, TC386422, TC388688, TC385780, TC388547, TC405784, TC399919, TC407572, TC372175, TC388822, TC396365, TC377496, TC388976, TC406708, TC419747, TC429771, TC423576, TC409459, TC386639, TC402186, TC416658, TC386646, TC393561, TC393948, TC393692, TC391671, TC393554, TC407614, TC459656, TC403264, TC402603, TC441241, TC449724, TC413571, TC379436, CA729339, TC417363, CK214224, TC403929, TC394118, TC425690, TC433589, TC389816, TC399352, TC422425, TC372530, CA613620, TC393820, CK211469, TC411191, TC434831, TC396895, TC432185, TC426326, TC375431, TC382342, TC393106, TC401758, TC373914, TC432504, TC396460, TC397676, TC450285, TC428944, TC389661 |
| [Positive regulation of molecular function](http://amigo.geneontology.org/cgi-bin/amigo/go.cgi?action=query&view=query&query=GO:0044093&search_constraint=terms) | TC410352, DR739471, TC434820, TC443387, TC379711, TC392272, TC417077, CA710880, TC402440, TC423252, TC394307, TC397660, TC375146, TC389363, TC388914, TC389589, TC446235, TC387410, TC392778, TC420735, TC386422, TC395453, TC388688, TC390569, TC416685, TC407076, TC403872, TC389090, TC389375, TC388822, TC377496, TC406708, TC382655, TC429771, CK200433, TC423576, BQ294582, TC406038, TC416658, TC395351, TC393561, TC401915, TC391671, TC393554, TC407614, DR739994, TC384454, CA729339, TC409599, CK217367, TC373259, TC379853, TC389816, TC405440, TC376420, TC377190, CA613620, TC423110, DR737360, TC396895, TC434831, TC426326, TC393106, BQ607161, TC396460, TC428944, TC382045 |
| [Inhibition of adenylate cyclase activity by G-protein signaling pathway](http://amigo.geneontology.org/cgi-bin/amigo/go.cgi?action=query&view=query&query=GO:0007193&search_constraint=terms) | TC393100, TC382928, CV775873, TC417363, TC386237, TC386313, TC393904, TC398052, TC425841, TC387579, TC371242, TC394459, TC384738, CK211707, TC386963, TC388410, TC377410, TC381218, TC395303, TC400755, TC458987, TC397312, TC401210, TC386961, TC390135, TC397729, TC377225, TC372175, TC407572, TC399919, TC396365, TC381462, TC419747, TC401758, TC392263, TC389661 |
| [Negative regulation of adenylate cyclase activity](http://amigo.geneontology.org/cgi-bin/amigo/go.cgi?action=query&view=query&query=GO:0007194&search_constraint=terms) | TC393100, TC382928, CV775873, TC417363, TC386237, TC386313, TC393904, TC398052, TC425841, TC387579, TC371242, TC394459, TC384738, CK211707, TC386963, TC388410, TC377410, TC381218, TC395303, TC400755, TC458987, TC397312, TC401210, TC386961, TC390135, TC397729, TC377225, TC372175, TC407572, TC399919, TC396365, TC381462, TC419747, TC401758, TC392263, TC389661 |
| [Negative regulation of cyclase activity](http://amigo.geneontology.org/cgi-bin/amigo/go.cgi?action=query&view=query&query=GO:0031280&search_constraint=terms) | TC393100, TC382928, CV775873, TC417363, TC386237, TC386313, TC393904, TC398052, TC425841, TC387579, TC371242, TC394459, TC384738, CK211707, TC386963, TC388410, TC377410, TC381218, TC395303, TC400755, TC458987, TC397312, TC401210, TC386961, TC390135, TC397729, TC377225, TC372175, TC407572, TC399919, TC396365, TC381462, TC419747, TC401758, TC392263, TC389661 |
| [Negative regulation of lyase activity](http://amigo.geneontology.org/cgi-bin/amigo/go.cgi?action=query&view=query&query=GO:0051350&search_constraint=terms) | TC393100, TC382928, CV775873, TC417363, TC386237, TC386313, TC393904, TC398052, TC425841, TC387579, TC371242, TC394459, TC384738, CK211707, TC386963, TC388410, TC377410, TC381218, TC395303, TC400755, TC458987, TC397312, TC401210, TC386961, TC390135, TC397729, TC377225, TC372175, TC407572, TC399919, TC396365, TC381462, TC419747, TC401758, TC392263, TC389661 |
| [Multicellular organismal process](http://amigo.geneontology.org/cgi-bin/amigo/go.cgi?action=query&view=query&query=GO:0032501&search_constraint=terms) | TC461921, TC410352, TC386040, TC373613, TC390393, TC397258, CV775873, TC375864, TC460760, TC451511, TC427210, TC398052, TC387861, CA710880, TC400388, TC398862, TC433162, TC394459, TC371242, TC384738, TC423252, TC397660, TC389363, TC379171, TC388410, CK203550, TC458562, TC416442, TC421914, TC406264, TC387410, TC380063, TC418414, CJ944525, TC378054, TC448471, TC395453, TC392875, TC409843, TC435546, TC384735, TC390569, TC405540, TC407076, TC419057, TC403872, TC425847, CJ727624, TC398714, BQ294582, CJ550278, TC398970, TC403580, TC413427, TC411128, TC401915, GH732878, TC398304, TC425878, TC393100, TC433957, TC412569, CA632212, DR739994, TC459193, TC394916, TC394206, TC394820, TC406236, TC418073, TC378274, TC417308, CK217367, TC391128, TC445767, TC423804, TC413460, TC379853, TC370315, BQ609416, TC449504, TC418685, TC395723, TC405440, CJ792862, TC385233, TC389718, TC404052, TC398514, TC377225, TC381943, TC432001, BQ607161, TC406807, TC372845, TC382045, TC404606, TC416906, TC402308, TC434820, TC441343, TC379711, TC400330, TC378790, CA595837, TC379903, TC392272, TC377061, CA730421, TC417077, TC417388, TC446465, TC440066, TC386313, CV759879, TC408907, CA606693, TC391438, CK211707, TC386963, TC392778, TC396751, TC397312, TC370350, TC405030, CK211589, TC388403, TC383909, TC376527, CA615187, TC383701, TC385780, TC405784, TC399919, TC407572, TC372175, TC406708, TC419747, TC409459, TC410074, TC402186, TC386646, CV763657, TC423354, TC403264, TC402603, TC441241, TC449724, TC413571, CA729339, TC383019, CK214224, BQ239045, CA709177, TC425690, TC381218, TC399352, TC408229, TC422425, CA613620, TC398805, TC400362, TC369092, CV781430, TC411191, TC434831, TC432185, TC426326, TC381462, TC375431, CD882425, TC377749, TC373914, TC392263, TC450285, TC396529, TC394796, TC397286, TC435533, TC374240, TC425841, CF554444, TC387579, TC406870, TC388914, TC393814, TC387621, TC398633, TC394716, TC446235, TC402545, TC378271, TC380943, TC393830, TC380433, TC420735, TC404371, TC384219, TC386961, TC416685, TC372664, TC387344, TC389090, TC389375, TC404413, TC382655, CK200433, TC370114, TC373994, TC406038, TC393970, TC395351, TC459245, TC456619, CK210754, TC369928, TC410147, TC376490, TC384454, CA598430, TC411287, TC386237, TC409343, TC393904, TC390994, TC387191, TC391143, TC428066, TC380416, TC381463, TC403157, TC386414, TC395303, TC400755, TC440636, TC405511, TC376420, TC458987, TC404843, TC423110, TC431879, CD876572, TC401210, DR737360, TC379357, TC390135, TC387135, TC397885, TC386279, TC411480, TC409650, TC380662, TC393960, TC389162, DR739471, TC443387, TC388566, TC386535, TC390630, TC390285, GH729256, TC418716, TC386519, TC390402, TC402440, TC391411, TC394307, TC375146, CK199175, TC389589, TC452762, TC386344, TC381963, TC383176, TC370044, CD878039, TC449463, TC386422, TC388688, TC403328, TC418845, TC400181, TC388547, TC414606, TC388822, TC377496, TC388976, TC396365, TC429771, TC423576, TC386639, TC375530, TC416658, TC393561, TC375834, TC393948, TC435799, TC393692, TC391671, TC393554, TC407614, TC459656, TC382928, TC438243, TC391007, TC379436, TC460615, TC417363, TC403573, TC387710, TC413700, TC403929, TC394118, TC449043, TC380882, TC440321, TC423880, TC433589, TC377410, TC389816, TC372530, TC393820, TC461622, CK211469, TC403588, TC416981, TC375798, TC397729, TC396895, TC418850, TC382342, TC393106, TC401758, TC432504, TC397676, TC396460, TC428944, TC389661, TC417067 |
| [Secretion](http://amigo.geneontology.org/cgi-bin/amigo/go.cgi?action=query&view=query&query=GO:0046903&search_constraint=terms) | TC398304, TC393100, TC433957, CK210754, TC378790, TC382928, CV775873, TC441241, TC449724, TC417363, TC386237, TC394916, TC386313, TC390994, TC398052, TC425841, TC378274, TC403929, TC387710, TC387579, CA606693, TC394459, TC384738, CK211707, TC425690, TC386963, TC388410, TC395303, TC422425, TC458562, TC400755, TC376420, TC458987, TC393820, TC396751, CA615187, TC386961, TC398514, TC390135, TC377225, TC411191, TC388547, TC387344, TC407076, TC405784, TC399919, TC407572, TC372175, TC409650, TC396365, TC419747, TC380662, TC401758, TC370114, TC409459, TC398714, TC389661, TC382045, TC404606 |
| [Regulation of biological process](http://amigo.geneontology.org/cgi-bin/amigo/go.cgi?action=query&view=query&query=GO:0050789&search_constraint=terms) | TC410352, TC386040, TC373613, TC407978, TC390393, TC397258, TC389190, CV775873, TC375864, TC398052, TC387861, CA710880, TC398862, TC433162, TC394459, TC371242, TC384738, TC423252, TC397660, TC389363, TC388410, TC458562, TC421914, TC387410, TC380063, TC418414, TC378054, TC395453, TC392329, TC384735, TC390569, CA682223, TC407076, TC419057, TC403872, TC398714, BQ294582, CJ550278, TC403580, TC401915, TC398304, TC393100, TC433957, CA632212, DR739994, TC394916, TC398731, TC378274, CK217367, TC391128, TC409599, TC379853, TC370315, TC395723, TC405440, TC403968, TC379536, TC385233, TC389718, TC398514, TC377225, TC381943, BQ607161, TC403977, TC406807, TC372845, TC382045, TC404606, CA646741, TC416906, TC402308, TC434820, TC441343, TC379711, TC378790, TC379903, TC392272, TC377061, TC417077, TC417388, TC440066, TC386313, CV759879, TC413339, CK208222, TC408907, CA606693, TC420579, TC391438, CK211707, TC386963, TC421345, TC392778, TC396751, TC397312, TC405030, CK211589, TC388403, TC383909, CA615187, TC405041, TC419584, TC383701, TC385780, TC405784, TC399919, TC407572, TC372175, TC406708, TC419747, TC409459, TC402186, TC386646, TC423354, TC402603, TC441241, TC449724, TC413571, CA729339, CK214224, BE585841, TC425690, TC381218, TC399352, TC422425, CA613620, TC400362, TC388520, TC395872, TC411191, TC434831, TC432185, TC426326, TC381462, TC373914, TC392263, TC392303, TC394796, TC445166, TC372677, TC435533, TC374240, TC425841, TC387579, TC388914, TC393814, TC398633, TC394716, TC404926, TC446235, TC378271, TC402545, TC380943, TC393830, TC380433, TC420735, TC404371, TC373615, TC384219, TC386961, TC398606, TC397562, TC416685, TC372664, TC387344, TC389090, TC389375, TC404413, TC382655, TC370114, TC373994, CK200433, TC406038, TC408192, TC393970, TC395351, CK210754, TC369928, TC384454, TC380329, TC411287, TC386237, TC387191, TC390994, TC393904, TC391143, TC386414, TC395303, TC400755, TC440636, TC405511, TC406193, TC405615, TC376420, TC458987, TC377190, TC423110, TC391613, TC422348, TC401210, DR737360, TC379357, TC390135, TC386279, TC388049, TC380662, TC393960, TC426358, DR739471, TC443387, TC407340, TC386519, TC402440, TC417341, TC394307, TC375146, TC398592, TC389589, TC452762, TC405475, TC386344, TC381963, TC383176, TC449463, TC386422, TC388688, TC388547, TC388822, TC377496, TC388976, TC396365, TC379069, TC429771, TC423576, TC386639, TC416658, TC381957, TC374230, TC393561, TC393692, TC391671, TC393554, TC407614, TC459656, TC382928, TC438243, TC379436, TC460615, TC417363, TC403929, TC387710, TC394118, TC380882, TC423880, TC372580, TC373259, TC433589, TC377410, TC389816, TC372530, TC400056, TC393820, TC461622, CK211469, TC381817, TC403588, TC375798, TC397729, TC396895, TC391122, TC418850, TC382342, TC393106, TC401758, TC397676, TC396460, TC432504, TC389661, TC428944 |
| [Protein phosphorylation](http://amigo.geneontology.org/cgi-bin/amigo/go.cgi?action=query&view=query&query=GO:0006468&search_constraint=terms) | CA646741, TC374230, TC386040, TC402308, TC393692, TC394796, CA632212, TC402603, TC459656, TC394965, TC411287, TC386519, TC374240, TC411941, TC369655, TC391143, TC457991, TC433162, TC409599, TC391438, TC370912, TC373259, TC433589, TC370315, TC386414, TC394716, TC398633, TC440636, TC395723, TC380063, TC376420, TC372167, TC393830, TC380943, TC386344, CK211469, TC405030, TC378054, TC381963, TC388403, TC383176, TC404371, TC435595, TC389718, TC384219, TC429747, TC385780, TC384688, TC387683, TC407076, TC419057, TC382342, TC388976, TC373914, TC406807, TC432504, TC386639, CD892838, CJ550278, TC397676, TC402186, TC395090, TC381957, TC382045 |
| [Regulation of synaptic transmission](http://amigo.geneontology.org/cgi-bin/amigo/go.cgi?action=query&view=query&query=GO:0050804&search_constraint=terms) | TC393100, TC382928, CV775873, TC449724, TC413571, TC417363, TC386237, TC386313, TC393904, TC398052, TC425841, TC387710, TC387579, TC394459, TC384738, CK211707, TC425690, TC386963, TC388410, TC377410, TC381218, TC395303, TC400755, TC458987, TC393820, TC397312, CK211589, TC401210, TC386961, TC390135, TC397729, TC377225, TC399919, TC407572, TC372175, TC396365, TC381462, TC419747, TC401758, TC392263, TC389661, TC404606, TC382045 |
| [Regulation of transmission of nerve impulse](http://amigo.geneontology.org/cgi-bin/amigo/go.cgi?action=query&view=query&query=GO:0051969&search_constraint=terms) | TC393100, TC382928, CV775873, TC449724, TC413571, TC417363, TC386237, TC386313, TC393904, TC398052, TC425841, TC387710, TC387579, TC394459, TC384738, CK211707, TC425690, TC386963, TC388410, TC377410, TC381218, TC395303, TC400755, TC458987, TC393820, TC397312, CK211589, TC401210, TC386961, TC390135, TC397729, TC377225, TC399919, TC407572, TC372175, TC396365, TC381462, TC419747, TC401758, TC392263, TC389661, TC404606, TC382045 |
| [Regulation of neurological system process](http://amigo.geneontology.org/cgi-bin/amigo/go.cgi?action=query&view=query&query=GO:0031644&search_constraint=terms) | TC393100, TC382928, CV775873, TC449724, TC413571, TC417363, TC386237, TC386313, TC393904, TC398052, TC425841, TC387710, TC387579, TC394459, TC384738, CK211707, TC425690, TC386963, TC388410, TC377410, TC381218, TC395303, TC400755, TC458987, TC393820, TC397312, CK211589, TC401210, TC386961, TC390135, TC397729, TC377225, TC399919, TC407572, TC372175, TC396365, TC381462, TC419747, TC401758, TC392263, TC389661, TC404606, TC382045 |
| [Catabolic process](http://amigo.geneontology.org/cgi-bin/amigo/go.cgi?action=query&view=query&query=GO:0009056&search_constraint=terms) | TC410352, TC386040, TC373613, TC390393, TC397258, TC375864, TC377766, TC420420, TC435533, TC374240, CA710880, TC457112, TC398862, TC433162, TC371242, TC423252, TC397660, TC393814, TC389363, TC388914, TC394716, TC398633, TC446235, TC380063, TC433844, TC393830, TC380943, TC369064, TC418414, CJ944525, TC378054, TC420735, TC404371, TC395453, TC384219, TC397562, TC390569, TC416685, TC407076, TC403872, TC389090, TC389375, TC404413, TC382655, CK200433, TC373791, TC370114, TC406038, TC393970, TC395351, TC401915, GH732878, TC393523, DR739994, TC384454, TC411287, TC388751, TC394820, TC391143, CK217367, TC409599, TC380416, TC379853, TC386414, TC395723, TC440636, TC405440, TC377190, TC423110, TC389718, DR737360, TC379357, TC424376, TC386279, TC376774, TC420394, CK197833, CK201148, TC381943, BQ607161, TC406807, TC393960, TC372845, TC382045, TC404606, DR739471, TC416906, TC402308, TC434820, TC443387, TC379711, TC377308, TC379903, TC416438, TC390285, TC392272, TC417388, TC386519, CV759879, TC408907, TC402440, TC391438, TC394307, TC375146, TC454407, TC389589, TC452762, TC386344, TC392778, TC370350, TC405030, TC381963, TC388403, TC383176, TC386422, TC388688, BJ317882, TC400181, TC385780, TC369199, TC388822, TC377496, TC388976, TC406708, TC429771, TC409459, TC423576, TC386639, TC402186, TC416658, TC393561, TC393692, TC391671, TC393554, TC407614, TC400477, TC373787, TC459656, TC402603, TC449724, TC391007, TC379436, CA729339, CK214224, TC387710, TC384010, TC372580, TC433589, TC389816, TC371172, CA613620, TC393820, CK211469, TC400362, TC381817, TC379422, DR740372, TC434831, TC432185, TC396895, TC426326, TC382342, TC393106, TC373914, TC432504, TC397676, TC396460, TC431198, TC428944 |
| [Sexual reproduction](http://amigo.geneontology.org/cgi-bin/amigo/go.cgi?action=query&view=query&query=GO:0019953&search_constraint=terms) | TC461921, TC410352, TC386040, TC373613, TC397258, TC460760, TC427210, TC435533, TC374240, TC387861, TC400388, CF554444, TC433162, TC371242, TC406870, TC423252, TC397660, TC389363, TC388914, TC379171, CK203550, TC387621, TC394716, TC398633, TC446235, TC406264, TC380063, TC393830, TC380943, TC378054, TC420735, TC404371, TC448471, TC395453, TC384219, TC392875, TC435546, TC390569, TC405540, TC416685, TC403872, TC425847, TC389090, TC389375, CJ727624, TC382655, CK200433, BQ294582, TC406038, TC393970, TC395351, TC403580, TC413427, TC411128, TC401915, TC425878, TC412569, TC410147, DR739994, TC384454, TC459193, TC411287, TC409343, TC394206, TC394820, TC406236, TC418073, TC417308, TC391143, CK217367, TC391128, TC445767, TC413460, TC381463, TC379853, TC403157, BQ609416, TC386414, TC449504, TC440636, TC395723, TC405440, CJ792862, TC376420, TC431879, TC404843, CD876572, TC389718, TC404052, TC379357, TC397885, TC386279, TC411480, TC381943, TC432001, BQ607161, TC389162, DR739471, TC402308, TC434820, TC443387, TC379711, TC400330, CA595837, TC386535, GH729256, TC392272, TC377061, CA730421, TC417077, TC446465, TC440066, TC386519, CV759879, TC408907, TC402440, TC391438, TC391411, TC394307, TC375146, TC389589, TC386344, TC392778, TC405030, TC381963, TC388403, TC383176, CD878039, TC376527, TC386422, TC388688, TC418845, TC385780, TC414606, TC388822, TC377496, TC388976, TC406708, TC429771, TC423576, TC386639, TC410074, TC402186, TC416658, CV763657, TC393561, TC393692, TC391671, TC393554, TC407614, TC402603, TC379436, CA729339, TC403573, CK214224, BQ239045, TC413700, TC394118, CA709177, TC449043, TC440321, TC433589, TC389816, TC408229, TC372530, CA613620, CK211469, TC398805, CV781430, TC434831, TC432185, TC396895, TC426326, CD882425, TC382342, TC393106, TC373914, TC432504, TC397676, TC396460, TC428944, TC417067 |
| [Response to wounding](http://amigo.geneontology.org/cgi-bin/amigo/go.cgi?action=query&view=query&query=GO:0009611&search_constraint=terms) | TC386040, TC402308, TC393692, TC390393, TC379903, TC397258, CA632212, TC402603, DR739350, TC449724, TC379436, TC411287, TC435533, TC386519, TC374240, CV759879, CK214224, TC408907, TC391143, TC433162, TC391438, TC371242, TC393814, TC386414, TC394716, TC398633, TC440636, TC452762, TC380063, TC393830, TC380943, TC393820, TC386344, CK211469, TC405030, TC378054, TC381963, TC388403, TC383176, TC370044, TC404371, TC373678, TC389718, TC384219, TC405041, TC379357, TC386279, TC385780, TC419057, TC381943, TC382342, TC388976, TC370114, TC409459, TC373914, TC432504, TC386639, TC397676, TC402186 |
| [Embryo development](http://amigo.geneontology.org/cgi-bin/amigo/go.cgi?action=query&view=query&query=GO:0009790&search_constraint=terms) | TC410352, TC386040, TC373613, TC390393, TC394796, TC397258, CV775873, TC375864, TC397286, TC451511, TC435533, TC374240, TC398052, TC387861, CA710880, TC425841, TC433162, TC387579, TC394459, TC371242, TC423252, TC397660, TC393814, TC389363, TC388914, TC379171, TC388410, TC458562, TC416442, TC394716, TC398633, TC421914, TC446235, TC387410, TC380063, TC402545, TC393830, TC380943, TC380433, TC378054, TC420735, TC404371, TC395453, TC384219, TC409843, TC384735, TC390569, TC405540, TC416685, TC387344, TC372664, TC407076, TC419057, TC403872, TC389090, TC389375, TC382655, CK200433, TC398714, TC373994, BQ294582, TC370114, TC406038, CJ550278, TC393970, TC398970, TC395351, TC403580, TC401915, TC398304, TC456619, TC393100, TC433957, CK210754, TC412569, CA632212, DR739994, TC384454, TC411287, CA598430, TC386237, TC409343, TC390994, TC387191, TC391143, CK217367, TC391128, TC423804, TC379853, TC395303, TC386414, TC395723, TC440636, TC405440, TC376420, TC385233, TC389718, TC390135, DR737360, TC379357, TC387135, TC386279, TC409650, TC381943, TC380662, BQ607161, TC406807, TC372845, TC382045, TC389162, DR739471, TC416906, TC402308, TC434820, TC443387, TC441343, TC379711, TC388566, TC378790, TC379903, TC390630, TC392272, TC390285, TC377061, TC417077, TC440066, TC386519, CV759879, TC408907, TC402440, CA606693, TC391438, TC394307, CK211707, TC375146, TC389589, TC452762, TC386344, TC392778, TC396751, TC397312, TC405030, TC381963, CK211589, TC388403, TC383176, TC370044, TC383909, TC449463, CA615187, TC386422, TC403328, TC388688, TC383701, TC385780, TC388547, TC405784, TC399919, TC407572, TC372175, TC388822, TC396365, TC377496, TC388976, TC406708, TC419747, TC429771, TC423576, TC409459, TC386639, TC402186, TC416658, TC386646, TC393561, TC375834, TC393948, TC393692, TC391671, TC393554, TC407614, TC403264, TC459656, TC402603, TC441241, TC449724, TC413571, TC379436, CA729339, TC417363, CK214224, TC403929, TC394118, TC425690, TC433589, TC389816, TC399352, TC422425, TC372530, CA613620, TC393820, CK211469, TC369092, TC416981, TC411191, TC434831, TC396895, TC432185, TC426326, TC375431, TC382342, TC393106, TC401758, TC373914, TC432504, TC396460, TC397676, TC450285, TC428944, TC389661, TC396529 |
| [Regulation of transcription from RNA polymerase II promoter](http://amigo.geneontology.org/cgi-bin/amigo/go.cgi?action=query&view=query&query=GO:0006357&search_constraint=terms) | TC392303, TC393692, TC390393, CK210754, TC379903, TC397258, CA632212, TC402603, TC441241, TC417077, TC379436, TC411287, TC460615, TC435533, TC394916, TC374240, TC386313, CV759879, CK214224, TC390994, TC408907, TC378274, TC387579, TC391438, TC386963, TC393814, TC433589, TC377410, TC381218, TC458562, TC400755, TC395723, TC452762, TC402545, CK211469, TC397312, TC400362, TC380433, TC388403, TC404371, TC401210, TC386961, TC384219, TC379357, TC397729, TC377225, TC386279, TC419057, TC381943, TC404413, TC381462, TC382342, TC418850, TC401758, TC370114, TC409459, TC398714, CJ550278, TC392263, TC402186, TC389661, TC393960 |
| [Positive regulation of cell proliferation](http://amigo.geneontology.org/cgi-bin/amigo/go.cgi?action=query&view=query&query=GO:0008284&search_constraint=terms) | TC410352, DR739471, TC434820, TC443387, TC379711, TC392272, TC407340, CA710880, TC402440, TC371242, TC423252, TC394307, TC397660, TC375146, TC389363, TC388914, TC389589, TC446235, TC392778, TC420735, TC386422, TC395453, TC388688, TC390569, TC416685, TC407076, TC403872, TC389090, TC389375, TC388822, TC377496, TC406708, TC382655, TC429771, CK200433, TC423576, BQ294582, TC406038, TC416658, TC395351, TC374230, TC393561, TC401915, TC391671, TC407614, TC393554, DR739994, TC384454, CA729339, CK217367, TC379853, TC389816, TC405440, TC376420, TC377190, CA613620, DR737360, TC396895, TC434831, TC426326, TC393106, BQ607161, TC396460, TC428944, TC382045 |
| [Postreplication repair](http://amigo.geneontology.org/cgi-bin/amigo/go.cgi?action=query&view=query&query=GO:0006301&search_constraint=terms) | TC400755, TC392303, CK210754, TC397312, TC377061, TC441241, TC401210, TC386961, TC397729, TC394916, TC386313, TC377225, TC390994, TC378274, TC387579, TC381462, TC401758, TC398714, TC386963, TC392263, TC377410, TC381218, TC389661, TC393960, TC458562 |
| [Response to external stimulus](http://amigo.geneontology.org/cgi-bin/amigo/go.cgi?action=query&view=query&query=GO:0009605&search_constraint=terms) | TC410352, DR739471, TC434820, TC373613, TC443387, TC379711, TC390630, TC392272, TC402440, TC423252, TC394307, TC397660, TC375146, TC389363, TC388914, TC379171, TC389589, TC446235, TC387410, TC433844, TC392778, TC420735, TC386422, TC395453, TC388688, TC390569, TC416685, TC407076, TC403872, TC389090, TC389375, TC388822, TC406708, TC377496, TC382655, TC429771, CK200433, TC423576, TC406038, TC416658, TC395351, TC386646, TC393561, TC401915, TC393948, TC369928, TC391671, TC393554, TC407614, TC459656, DR739994, TC384454, CA729339, TC391128, CK217367, TC380882, TC425690, TC379853, TC389816, TC405440, TC376420, TC461622, TC403588, DR737360, TC432185, TC396895, TC434831, TC426326, TC377749, TC393106, BQ607161, TC406807, TC396460, TC428944, TC382045 |
| [Anatomical structure morphogenesis](http://amigo.geneontology.org/cgi-bin/amigo/go.cgi?action=query&view=query&query=GO:0009653&search_constraint=terms) | TC410352, TC386040, TC407978, TC390393, TC397258, TC375864, TC435533, TC374240, CA710880, TC433162, TC371242, TC423252, TC397660, TC393814, TC389363, TC388914, TC394716, TC398633, TC421914, TC446235, TC387410, TC380063, TC378271, TC393830, TC380943, TC378054, TC420735, TC395453, TC384219, TC384735, TC390569, TC416685, TC407076, TC419057, TC403872, TC389090, TC389375, TC382655, CK200433, BQ294582, TC370114, TC406038, CJ550278, TC393970, TC395351, TC401915, DR739994, TC384454, TC376490, TC391143, CK217367, TC391128, TC379853, TC370315, TC386414, TC405511, TC440636, TC395723, TC418685, TC405440, TC376420, TC423110, TC389718, DR737360, TC381943, BQ607161, TC382045, DR739471, TC402308, TC434820, TC441343, TC443387, TC379711, TC379903, TC378790, TC390630, TC392272, TC377061, TC417077, TC418716, TC386519, CV759879, TC402440, TC391438, TC394307, TC375146, TC389589, TC452762, TC386344, TC392778, TC370350, TC405030, TC381963, TC388403, TC383176, TC386422, TC388688, TC418845, TC385780, TC388822, TC377496, TC388976, TC406708, TC429771, TC409459, TC423576, TC386639, TC416658, TC393561, TC375834, TC393948, TC393692, TC391671, TC393554, TC407614, TC402603, TC423354, TC438243, TC433557, CA729339, TC380882, TC423880, TC433589, TC389816, TC372530, CA613620, TC461622, CK211469, TC403588, TC375798, TC396895, TC434831, TC426326, TC382342, TC377749, TC393106, TC373914, TC397676, TC396460, TC432504, TC428944, TC396529 |
| [Spermatogenesis](http://amigo.geneontology.org/cgi-bin/amigo/go.cgi?action=query&view=query&query=GO:0007283&search_constraint=terms) | TC461921, TC386040, TC402308, TC400330, CA595837, TC397258, TC386535, GH729256, TC460760, CA730421, TC446465, TC427210, TC435533, TC386519, TC374240, CV759879, TC408907, TC400388, CF554444, TC433162, TC391438, TC391411, TC406870, CK203550, TC387621, TC394716, TC398633, TC406264, TC380063, TC393830, TC380943, TC386344, TC378054, TC405030, TC381963, TC388403, TC383176, TC404371, CD878039, TC448471, TC376527, TC384219, TC392875, TC418845, TC435546, TC414606, TC405540, TC385780, TC425847, CJ727624, TC388976, TC386639, TC410074, TC402186, TC413427, CV763657, TC411128, TC425878, TC393692, TC402603, TC410147, TC459193, TC379436, TC411287, TC403573, CK214224, TC394206, TC394820, BQ239045, TC406236, TC418073, TC417308, TC413700, TC391143, CA709177, TC449043, TC440321, TC445767, TC413460, TC433589, TC381463, TC403157, BQ609416, TC386414, TC408229, TC449504, TC395723, TC440636, CJ792862, TC431879, TC404843, CK211469, TC398805, CD876572, CV781430, TC404052, TC389718, TC379357, TC397885, TC386279, TC411480, TC381943, CD882425, TC382342, TC432001, TC373914, TC432504, TC397676, TC417067 |
| [Male gamete generation](http://amigo.geneontology.org/cgi-bin/amigo/go.cgi?action=query&view=query&query=GO:0048232&search_constraint=terms) | TC461921, TC386040, TC402308, TC400330, CA595837, TC397258, TC386535, GH729256, TC460760, CA730421, TC446465, TC427210, TC435533, TC386519, TC374240, CV759879, TC408907, TC400388, CF554444, TC433162, TC391438, TC391411, TC406870, CK203550, TC387621, TC394716, TC398633, TC406264, TC380063, TC393830, TC380943, TC386344, TC378054, TC405030, TC381963, TC388403, TC383176, TC404371, CD878039, TC448471, TC376527, TC384219, TC392875, TC418845, TC435546, TC414606, TC405540, TC385780, TC425847, CJ727624, TC388976, TC386639, TC410074, TC402186, TC413427, CV763657, TC411128, TC425878, TC393692, TC402603, TC410147, TC459193, TC379436, TC411287, TC403573, CK214224, TC394206, TC394820, BQ239045, TC406236, TC418073, TC417308, TC413700, TC391143, CA709177, TC449043, TC440321, TC445767, TC413460, TC433589, TC381463, TC403157, BQ609416, TC386414, TC408229, TC449504, TC395723, TC440636, CJ792862, TC431879, TC404843, CK211469, TC398805, CD876572, CV781430, TC404052, TC389718, TC379357, TC397885, TC386279, TC411480, TC381943, CD882425, TC382342, TC432001, TC373914, TC432504, TC397676, TC417067 |
| [Biological regulation](http://amigo.geneontology.org/cgi-bin/amigo/go.cgi?action=query&view=query&query=GO:0065007&search_constraint=terms) | TC410352, TC386040, TC373613, TC407978, TC390393, TC397258, TC389190, CV775873, TC375864, TC398052, TC387861, CA710880, TC398862, TC433162, TC394459, TC371242, TC384738, TC423252, TC397660, TC389363, TC388410, TC458562, TC421914, TC387410, TC380063, TC418414, TC378054, TC395453, TC392329, TC384735, TC390569, CA682223, TC407076, TC419057, TC403872, TC398714, BQ294582, CJ550278, TC403580, TC401915, GH732878, TC398304, TC393100, TC433957, TC412569, CA632212, DR739994, TC394916, TC398731, TC378274, CK217367, TC391128, TC409599, TC379853, TC370315, TC395723, TC405440, TC403968, TC379536, TC385233, TC389718, TC398514, TC377225, TC381943, BQ607161, TC403977, TC406807, TC372845, TC382045, TC404606, CA646741, TC416906, TC402308, TC434820, TC441343, TC379711, TC378790, TC379903, TC392272, TC377061, TC417077, TC417388, TC440066, TC386313, CV759879, TC413339, CK208222, TC408907, CA606693, TC420579, TC391438, CK211707, TC386963, TC421345, TC392778, TC396751, TC397312, TC370350, TC405030, CK211589, TC388403, TC383909, CA615187, TC405041, TC419584, TC383701, TC385780, TC405784, TC399919, TC407572, TC372175, TC406708, TC419747, TC409459, TC402186, TC386646, TC423354, TC402603, TC441241, TC433557, TC449724, TC413571, CA729339, CK214224, BE585841, TC425690, TC381218, TC399352, TC422425, TC384344, CA613620, TC400362, TC388520, TC395872, TC411191, TC434831, TC432185, TC426326, TC381462, TC377749, TC373914, TC392263, TC392303, TC394796, TC445166, TC372677, TC435533, TC374240, TC425841, TC387579, TC388914, TC393814, TC398633, TC394716, TC404926, TC446235, TC378271, TC402545, TC380943, TC393830, TC380433, TC420735, TC404371, TC373615, TC384219, TC386961, TC398606, TC397562, TC416685, TC372664, TC387344, TC389090, TC389375, TC404413, TC382655, TC370114, TC373994, CK200433, TC406038, TC408192, TC393970, TC395351, CK210754, TC369928, TC384454, TC380329, TC411287, TC386237, TC409343, TC393904, TC387191, TC390994, TC391143, TC386414, TC395303, TC400755, TC440636, TC405511, TC406193, TC405615, TC376420, TC458987, TC377190, TC423110, TC391613, TC422348, TC401210, DR737360, TC379357, TC390135, TC386279, TC388049, TC409650, TC380662, TC393960, TC426358, DR739471, TC443387, TC407340, TC386519, TC402440, TC417341, TC394307, TC409208, TC375146, TC375813, TC398592, TC389589, TC452762, TC405475, TC386344, TC381963, TC383176, TC370044, TC449463, TC386422, TC388688, TC388547, TC388822, TC377496, TC388976, TC396365, TC379069, TC429771, TC423576, TC386639, TC416658, TC381957, TC374230, TC393561, TC393692, TC386322, TC391671, TC393554, TC407614, TC459656, TC382928, TC438243, TC379436, TC460615, TC417363, TC403929, TC387710, TC394118, TC380882, TC423880, TC372580, TC373259, TC433589, TC377410, TC418091, TC389816, TC372530, TC446092, TC400056, TC393820, TC461622, CK211469, TC381817, TC403588, TC375798, TC397729, TC396895, TC391122, TC418850, TC382342, TC393106, TC401758, TC397676, TC396460, TC432504, TC389661, TC428944 |
| [Response to water deprivation](http://amigo.geneontology.org/cgi-bin/amigo/go.cgi?action=query&view=query&query=GO:0009414&search_constraint=terms) | TC405356, TC418032, TC397444, TC402534, TC415083, TC416529, TC423265, TC417012, TC396230, TC401244, TC406584, TC413043, TC416069, BQ838511, TC408299, TC406236, TC391130, TC424204, TC376263, TC379635, TC417992, TC416154, TC416169, TC446038, TC416493, CK214702, TC412732, TC392323, TC403986, TC413066, CK213497, TC398538, TC405695, CB307332, TC410063, TC404842, TC403885, TC385365, TC376933, TC416695, TC410194, TC411684, TC406469, TC445939, TC403803, TC412483, TC399342, TC410690, TC412520, TC413392 |
| [Reproductive process](http://amigo.geneontology.org/cgi-bin/amigo/go.cgi?action=query&view=query&query=GO:0022414&search_constraint=terms) | TC461921, TC410352, TC386040, TC373613, TC390393, TC394796, TC397258, TC375864, TC397286, TC460760, TC427210, TC435533, TC374240, TC387861, TC425841, TC400388, CF554444, TC433162, TC371242, TC406870, TC423252, TC397660, TC393814, TC389363, TC388914, TC379171, CK203550, TC387621, TC416442, TC394716, TC398633, TC446235, TC406264, TC380063, TC393830, TC380943, TC378054, TC420735, TC404371, TC448471, TC395453, TC384219, TC392875, TC435546, TC384735, TC390569, TC405540, TC416685, TC407076, TC419057, TC403872, TC425847, TC389090, TC389375, CJ727624, TC382655, CK200433, BQ294582, TC370114, TC406038, CJ550278, TC393970, TC398970, TC395351, TC403580, TC413427, TC411128, TC401915, TC425878, TC412569, CA632212, TC410147, DR739994, TC384454, TC459193, TC411287, TC409343, TC394206, TC394820, TC406236, TC418073, TC417308, TC391143, CK217367, TC391128, TC445767, TC380416, TC423804, TC413460, TC381463, TC379853, TC403157, BQ609416, TC386414, TC449504, TC395723, TC440636, TC405440, CJ792862, TC376420, TC431879, TC404843, CD876572, TC389718, TC404052, TC379357, TC386279, TC397885, TC411480, TC381943, TC432001, BQ607161, TC389162, DR739471, TC402308, TC434820, TC443387, TC441343, TC379711, TC400330, TC378790, TC379903, CA595837, TC390630, TC386535, GH729256, TC392272, TC390285, TC377061, CA730421, TC417077, TC446465, TC440066, TC386519, CV759879, TC408907, TC402440, TC391438, TC391411, TC394307, TC375146, TC389589, TC452762, TC386344, TC392778, TC370350, TC405030, TC381963, TC388403, TC383176, TC370044, CD878039, TC376527, TC386422, TC403328, TC388688, TC383701, TC418845, TC414606, TC385780, TC388822, TC377496, TC388976, TC406708, TC429771, TC423576, TC409459, TC386639, TC410074, TC402186, TC416658, TC386646, CV763657, TC393561, TC375834, TC393948, TC393692, TC391671, TC393554, TC407614, TC403264, TC402603, TC379436, CA729339, TC417363, TC403573, CK214224, BQ239045, TC413700, CA709177, TC394118, TC449043, TC440321, TC425690, TC433589, TC389816, TC408229, TC372530, CA613620, CK211469, TC398805, TC369092, CV781430, TC416981, TC434831, TC396895, TC432185, TC426326, CD882425, TC382342, TC393106, TC373914, TC432504, TC396460, TC397676, TC450285, TC428944, TC417067, TC396529 |
| [Response to water](http://amigo.geneontology.org/cgi-bin/amigo/go.cgi?action=query&view=query&query=GO:0009415&search_constraint=terms) | TC405356, TC418032, TC397444, TC402534, TC415083, TC416529, TC423265, TC417012, TC396230, TC401244, TC371008, TC406584, TC413043, TC416069, BQ838511, TC374268, TC408299, TC406236, TC391130, TC424204, TC376263, TC379635, TC417992, TC416154, TC416169, TC446038, TC416493, CK214702, TC412732, TC392323, TC403986, TC413066, CK213497, TC398538, TC405695, CB307332, TC410063, TC404842, TC403885, TC385365, TC376933, TC416695, TC410194, TC411684, TC406469, TC445939, TC403803, TC412483, TC399342, TC410690, TC412520, TC413392 |
| [Jasmonic acid metabolic process](http://amigo.geneontology.org/cgi-bin/amigo/go.cgi?action=query&view=query&query=GO:0009694&search_constraint=terms) | TC392033, TC375726, TC409187, TC389993, TC430544, TC391946, TC370633, TC398536, TC397909, TC379965, TC411908 |
| [Jasmonic acid biosynthetic process](http://amigo.geneontology.org/cgi-bin/amigo/go.cgi?action=query&view=query&query=GO:0009695&search_constraint=terms) | TC392033, TC375726, TC409187, TC389993, TC430544, TC391946, TC370633, TC398536, TC397909, TC379965, TC411908 |
| [Oxylipin metabolic process](http://amigo.geneontology.org/cgi-bin/amigo/go.cgi?action=query&view=query&query=GO:0031407&search_constraint=terms) | TC392033, TC375726, TC409187, TC389993, TC430544, TC391946, TC370633, TC398536, TC397909, TC379965, TC411908 |
| [Oxylipin biosynthetic process](http://amigo.geneontology.org/cgi-bin/amigo/go.cgi?action=query&view=query&query=GO:0031408&search_constraint=terms) | TC392033, TC375726, TC409187, TC389993, TC430544, TC391946, TC370633, TC398536, TC397909, TC379965, TC411908 |
| [Immune system process](http://amigo.geneontology.org/cgi-bin/amigo/go.cgi?action=query&view=query&query=GO:0002376&search_constraint=terms) | TC392303, TC398304, TC393100, TC433957, CK210754, TC382928, CV775873, TC377061, TC441241, TC386237, TC394916, TC386313, TC393904, TC398052, TC403929, TC387579, TC394118, TC394459, TC384738, CK211707, TC425690, TC386963, TC434396, TC388410, TC377410, TC381218, TC395303, TC398592, TC422425, TC458562, TC400755, TC376420, TC396751, TC385233, CA615187, TC388520, TC401210, TC386961, TC386707, TC405041, TC390135, TC397729, TC411191, TC388547, TC387344, TC407076, TC405784, TC399919, TC407572, TC372175, TC381462, TC396365, TC419747, TC380662, TC401758, TC370114, TC409459, TC398714, TC392263, TC389661 |
| [Regulation of system process](http://amigo.geneontology.org/cgi-bin/amigo/go.cgi?action=query&view=query&query=GO:0044057&search_constraint=terms) | TC393100, TC382928, CV775873, TC449724, TC413571, TC417363, TC386237, TC386313, TC393904, TC398052, TC425841, TC387710, TC387579, TC394459, TC384738, CK211707, TC425690, TC386963, TC388410, TC377410, TC381218, TC395303, TC400755, TC458987, TC393820, TC397312, CK211589, TC401210, TC386961, TC390135, TC397729, TC377225, TC399919, TC407572, TC372175, TC396365, TC381462, TC419747, TC401758, TC392263, TC389661, TC404606, TC382045 |
| [Cellular process](http://amigo.geneontology.org/cgi-bin/amigo/go.cgi?action=query&view=query&query=GO:0009987&search_constraint=terms) | TC461921, TC410352, TC386040, TC373613, TC407978, TC390393, TC444546, TC416529, TC389190, TC453487, TC391946, TC375864, TC420420, TC460760, BQ838511, TC417106, TC398052, TC387861, CA710880, TC400388, TC457112, TC421871, TC376351, TC394459, TC371242, TC416154, TC384738, TC423252, TC397660, TC379171, TC388410, BJ282766, CK203550, TC416493, TC458562, TC421914, TC393436, TC380063, TC398538, TC433844, TC418414, CJ944525, TC410063, TC378054, TC373702, TC448471, TC456784, TC395453, TC392329, TC409843, TC369255, TC407076, TC403872, TC425847, CJ727624, CA614761, CJ550278, TC411128, GH732878, TC397444, TC398304, TC425878, TC393100, TC412569, TC417012, CA632212, TC455515, TC413027, TC394916, TC394820, TC418073, TC378274, TC411784, TC391130, TC417308, CK217367, TC413460, BQ609416, CK214702, TC395723, TC385233, TC398730, TC389718, TC404052, TC386707, TC381943, TC432001, TC413392, TC418928, BQ607161, TC372845, TC382045, TC369628, TC402308, TC441343, TC400330, TC400260, TC378790, TC377061, TC417077, TC446465, CV759879, CA606693, TC391438, TC370912, TC432369, TC405695, TC392778, TC397312, TC405030, TC388403, CA615187, TC416695, TC405041, BJ317882, TC406469, TC399919, TC407572, TC402121, TC410690, TC406708, TC409459, TC410074, TC386646, CV763657, TC458205, TC402603, TC441241, TC433557, TC449724, TC413571, TC397909, CA729339, TC408299, BQ239045, TC389993, TC399352, TC422425, TC408312, CA613620, TC398805, TC400362, TC428992, TC369092, TC429747, TC430501, TC400108, TC399342, TC432185, TC381462, CD882425, TC377749, TC455736, TC396451, TC392263, TC431198, TC425957, TC445166, TC372677, TC374240, CA720842, TC425841, TC389168, TC398121, TC424204, TC387579, TC406870, TC413854, TC393814, BJ279521, TC376220, TC446235, TC402545, TC393830, TC380943, TC403885, TC386961, TC384688, TC387344, TC389090, TC370347, TC412483, TC382655, CK200433, TC373994, TC393970, TC395351, TC418032, TC456619, TC397291, TC411908, CA598430, TC380329, EB512907, TC409343, TC390994, TC391143, TC428066, TC381463, TC403157, TC410066, TC395303, TC405511, TC377190, TC443814, TC431879, TC423110, TC391613, TC385365, TC430544, TC401210, DR737360, TC379357, TC387135, TC397885, TC411684, TC438587, TC374485, TC393960, TC409077, TC443387, TC388566, TC415083, TC399245, TC390630, TC386535, GH729256, CA605200, TC386519, TC369655, TC390402, TC402072, TC417992, TC417341, TC394307, TC388665, TC389589, TC398592, TC435034, TC381988, TC452762, TC405475, TC386344, TC373637, TC370044, CD878039, TC435595, TC374164, TC386422, TC418845, TC414606, TC412150, TC388822, TC429771, TC386639, TC416658, TC381957, TC396650, TC379965, TC374230, TC393948, TC371970, TC375726, TC393554, TC407614, TC400477, TC459656, TC391007, TC379436, TC460615, TC403573, TC411941, TC457991, TC403929, TC394118, TC449043, TC392247, TC379635, TC387116, TC373259, TC433589, TC398343, TC418091, TC389816, TC372530, TC392323, TC413066, TC384194, TC371172, TC439472, TC372167, TC391641, TC461622, TC400056, TC403588, TC381817, TC385659, TC403803, TC391122, TC418850, TC401758, TC428944, TC389661, TC417067, TC379942, TC397258, TC396230, CV775873, TC377766, TC451511, TC394965, TC416069, TC427210, TC375313, TC398862, TC433162, TC372450, TC389363, TC434442, TC380590, TC411471, TC416442, TC378878, TC387410, TC406264, TC397176, TC410194, TC369371, TC392875, TC435546, TC384735, TC405540, TC390569, TC419057, TC391948, BQ294582, TC398714, TC373791, TC381619, TC394028, TC398970, TC457126, TC403580, TC413427, TC401915, TC433957, TC385710, DR739994, TC459193, TC394206, TC406236, TC409599, TC391128, TC391962, TC445767, TC404636, TC446038, TC423804, TC379853, TC413199, TC370315, TC449504, TC405440, TC403968, CJ792862, CK213497, TC414899, TC370885, TC398514, TC391900, TC377225, TC392033, TC412520, TC406807, CD892838, TC395090, TC404606, CA646741, TC416906, TC434820, TC379711, TC379903, CA595837, TC416438, TC423265, TC392272, TC401244, CA730421, TC417388, TC440066, TC386313, TC408907, TC420579, CK211707, TC386963, TC412732, TC431201, CA700201, TC370350, TC396751, CK211589, TC383909, TC376527, TC388718, TC419584, TC383701, TC385780, TC374392, TC405784, TC372175, TC419747, TC402186, TC405356, TC403264, TC423354, TC369348, TC413043, CK214224, TC368603, TC376263, BE585841, CA709177, TC409187, TC416169, TC425690, TC423182, TC381218, TC408229, TC383677, TC392297, CV781430, TC388520, TC379422, TC411191, TC387683, TC434831, TC426326, TC375431, TC391995, TC415501, TC373914, TC450285, TC392303, TC369899, TC394796, TC372330, CA611770, TC435533, TC395966, CF554444, TC415685, TC388914, TC387621, TC377438, TC398633, TC394716, TC378271, TC369064, TC380433, TC420735, TC435281, TC404371, TC397964, TC384219, TC397562, TC416685, TC372664, TC389375, TC404413, TC370114, TC406038, TC376874, TC459245, CK210754, TC369928, TC417769, TC410147, TC376490, TC384454, TC411287, TC386237, TC393904, TC388751, TC399471, TC380416, TC434396, TC398536, TC421880, TC386414, TC400755, TC440636, TC372701, TC403986, TC405615, TC376420, TC458987, TC422851, TC404843, TC384553, CD876572, TC382737, TC370633, TC369736, TC390135, TC424376, TC386279, TC411480, TC376774, CK197833, TC420394, TC391785, TC382786, TC409650, TC380662, TC388649, TC395298, TC382830, TC389162, DR739471, TC402534, CK212850, TC390285, TC369726, TC407340, TC406584, TC402440, TC391411, TC409208, TC375146, TC375813, TC454407, TC384122, TC394661, TC381963, TC383176, TC430821, TC371455, TC371037, TC449463, TC374879, TC388688, TC403328, TC400181, TC388547, TC368549, TC377496, TC388976, TC396365, TC410078, TC423576, TC397500, TC368591, TC402668, TC393561, TC435799, TC393692, TC391671, TC382928, TC438243, TC375918, TC417363, TC387710, TC413700, TC385515, TC380882, TC440321, TC423880, TC372580, TC377410, TC396083, TC393820, TC451519, CK211469, CB307332, CJ930688, TC404842, TC435808, TC397729, TC399408, TC396895, TC382342, TC390489, TC393106, TC432504, TC397676, TC396460 |
| [Multicellular organismal development](http://amigo.geneontology.org/cgi-bin/amigo/go.cgi?action=query&view=query&query=GO:0007275&search_constraint=terms) | TC410352, TC386040, TC373613, TC390393, TC394796, TC397258, CV775873, TC375864, TC397286, TC451511, TC435533, TC374240, TC398052, TC387861, CA710880, TC425841, TC433162, TC387579, TC394459, TC371242, TC423252, TC397660, TC393814, TC389363, TC388914, TC379171, TC388410, TC458562, TC416442, TC394716, TC398633, TC421914, TC446235, TC387410, TC380063, TC402545, TC378271, TC393830, TC380943, CJ944525, TC380433, TC378054, TC420735, TC404371, TC395453, TC384219, TC409843, TC384735, TC390569, TC405540, TC416685, TC387344, TC372664, TC407076, TC419057, TC403872, TC389090, TC389375, TC404413, TC382655, CK200433, TC398714, TC373994, BQ294582, TC370114, TC406038, CJ550278, TC393970, TC398970, TC395351, TC403580, TC401915, TC459245, TC398304, TC456619, TC393100, TC433957, CK210754, TC369928, TC412569, CA632212, DR739994, TC384454, TC376490, TC411287, CA598430, TC386237, TC409343, TC390994, TC387191, TC391143, CK217367, TC391128, TC428066, TC380416, TC423804, TC379853, TC370315, TC395303, TC386414, TC405511, TC418685, TC395723, TC440636, TC405440, TC376420, TC423110, TC385233, TC389718, TC390135, TC379357, DR737360, TC387135, TC386279, TC409650, TC381943, TC380662, BQ607161, TC406807, TC393960, TC372845, TC382045, TC389162, DR739471, TC416906, TC402308, TC434820, TC443387, TC441343, TC379711, TC388566, TC378790, TC379903, TC390630, TC392272, TC390285, TC377061, TC417077, TC418716, TC440066, TC386519, CV759879, TC390402, TC408907, TC402440, CA606693, TC391438, TC394307, CK211707, TC375146, TC389589, TC452762, TC386344, TC392778, TC396751, TC397312, TC370350, TC405030, TC381963, CK211589, TC388403, TC383176, TC370044, TC383909, TC449463, CA615187, TC386422, TC403328, TC388688, TC383701, TC400181, TC385780, TC388547, TC405784, TC399919, TC407572, TC372175, TC388822, TC396365, TC406708, TC377496, TC388976, TC419747, TC429771, TC423576, TC409459, TC386639, TC375530, TC402186, TC416658, TC386646, TC393561, TC375834, TC435799, TC393948, TC393692, TC391671, TC393554, TC407614, TC403264, TC459656, TC402603, TC423354, TC438243, TC441241, TC449724, TC413571, TC391007, TC379436, CA729339, TC383019, TC417363, TC460615, CK214224, TC403929, TC394118, TC380882, TC423880, TC425690, TC433589, TC399352, TC389816, TC422425, TC372530, TC461622, CA613620, TC393820, CK211469, TC400362, TC369092, TC403588, TC416981, TC375798, TC411191, TC434831, TC396895, TC432185, TC426326, TC382342, TC375431, TC418850, TC393106, TC377749, TC401758, TC373914, TC432504, TC396460, TC397676, TC450285, TC428944, TC389661, TC396529 |
| [Response to chemical stimulus](http://amigo.geneontology.org/cgi-bin/amigo/go.cgi?action=query&view=query&query=GO:0042221&search_constraint=terms) | TC410352, TC373613, TC394796, TC416529, TC396230, TC397286, TC416069, BQ838511, TC424204, TC398862, TC376351, TC416154, TC423252, TC397660, TC389363, TC388914, TC379171, TC416493, TC416442, TC446235, TC387410, TC398538, TC418414, TC388950, CJ944525, TC410063, TC380433, TC403885, TC420735, TC373702, TC427405, TC395453, TC410194, TC392329, TC390569, TC416685, TC407076, TC445939, TC403872, TC389090, TC412483, TC389375, TC382655, CK200433, TC370114, TC406038, TC393970, TC398970, TC395351, TC401915, TC418032, TC397444, TC459245, CV771134, TC417012, DR739994, TC384454, TC380329, TC394820, TC406236, TC391130, CK217367, TC391128, TC446038, TC379853, TC370315, CK214702, TC403986, TC405511, TC418685, TC405440, TC403968, CK213497, TC376420, TC385365, TC391742, DR737360, TC411684, TC412520, TC413392, BQ607161, TC406807, TC372845, TC382045, TC382830, DR739471, TC416906, TC402534, TC434820, TC443387, CK212850, TC379711, TC415083, TC378790, TC423265, TC390630, TC392272, TC401244, TC399636, TC418716, TC417388, TC406584, TC402440, TC420579, TC417992, TC394307, TC375146, TC398592, TC389589, TC412732, TC405695, TC392778, TC370350, TC383909, TC449463, TC386422, TC416695, TC385701, TC388688, TC405041, TC406469, TC412150, TC388822, TC410690, TC377496, TC406708, TC429771, TC409459, TC423576, TC416658, TC386646, TC405356, TC393561, TC391913, TC375834, TC393948, TC391671, TC393554, TC407614, TC400477, TC397415, TC459656, TC403264, TC423354, TC438243, TC369348, TC371008, TC413043, CA729339, TC374268, TC411941, TC408299, TC394118, TC376263, TC380882, TC423880, TC379635, TC416169, TC425690, TC389816, TC372530, TC392323, TC413066, TC383677, TC461622, CB307332, TC404842, TC403588, TC376933, TC403803, TC399342, TC434831, TC432185, TC396895, TC426326, TC440526, TC393106, TC377749, TC396460, TC450285, TC428944, TC381279 |
| [Polytene chromosome puffing](http://amigo.geneontology.org/cgi-bin/amigo/go.cgi?action=query&view=query&query=GO:0035079&search_constraint=terms) | TC423354, TC403588, TC461622, TC380882, TC423880 |
| [Heat shock-mediated polytene chromosome puffing](http://amigo.geneontology.org/cgi-bin/amigo/go.cgi?action=query&view=query&query=GO:0035080&search_constraint=terms) | TC423354, TC403588, TC461622, TC380882, TC423880 |
| [Biological_process](http://amigo.geneontology.org/cgi-bin/amigo/go.cgi?action=query&view=query&query=GO:0008150&search_constraint=terms) | TC461921, TC410352, TC386040, TC373613, TC390944, TC407978, TC373958, TC390393, TC444546, TC416529, TC389190, TC453487, TC391946, TC375864, TC420420, TC460760, TC408524, TC389842, BQ838511, TC417106, TC398052, TC387861, TC374098, CA710880, TC452945, TC400388, TC457112, TC421871, CK215979, CK206714, TC376351, TC394459, TC371242, TC416154, TC384738, TC423252, TC397660, TC379171, TC388410, BJ282766, CK203550, TC416493, TC374996, TC458562, TC421914, TC393436, TC417260, TC380063, TC398538, TC433844, TC418414, CJ944525, TC410063, TC378054, TC386396, TC373702, TC410954, TC448471, TC456784, TC395453, TC392329, TC409843, TC406594, TC369255, CA682223, TC407076, TC445939, TC403872, TC425847, CJ727624, CA614761, CJ550278, TC411128, GH732878, TC397444, TC398304, TC379558, TC425878, TC393100, TC412569, CV771134, TC417012, TC391447, CA632212, TC393523, TC455515, TC413027, TC394916, TC394820, TC398731, TC418073, TC378274, TC411784, TC391130, TC417308, CK217367, TC378153, TC413460, BQ609416, TC452050, CK214702, TC395723, TC387981, TC379536, TC385233, TC398730, TC391621, TC389718, TC404052, TC386707, TC376248, TC374409, CK201148, TC381943, TC432001, TC398379, TC413392, TC418928, BQ607161, TC372845, TC382045, TC369628, TC382950, TC402308, TC441343, TC400330, TC400260, TC378790, TC378432, TC377061, TC417077, TC446465, TC416801, BF474051, CV759879, TC391438, CA606693, TC370912, TC412317, TC432369, TC412212, TC425291, TC421345, TC405695, TC392778, TC397312, TC405030, TC388403, TC373678, CA615187, TC416695, TC405041, BJ317882, TC406469, TC399919, TC407572, TC402121, TC410690, TC406708, TC409459, TC410074, TC386646, CV763657, TC458205, TC402603, TC441241, TC433557, TC449724, TC413571, TC377559, TC397909, CA729339, TC383019, TC408299, BQ239045, TC389993, TC398830, TC399352, TC422425, TC408312, TC401941, CA613620, TC372654, TC398805, TC400362, TC428992, TC369092, TC429747, TC375612, TC430501, TC395872, TC400108, TC399342, TC432185, TC381462, CD882425, TC377749, TC455736, TC396451, TC392263, TC431198, TC424344, TC432130, TC396529, TC373145, TC404158, CK201269, TC425957, TC445166, TC372677, TC429341, TC374240, CA720842, TC425841, TC389168, TC398121, TC424204, TC387579, TC406870, TC413854, TC393814, BJ279521, TC376220, TC408309, TC397779, TC446235, TC404926, TC402545, TC393830, TC380943, TC403885, TC386961, TC398606, TC370603, TC384688, TC369182, TC387344, TC373204, TC418365, TC389090, TC370347, TC412483, TC373002, TC382655, CK200433, TC373994, TC393970, TC374731, TC395351, TC392709, TC418032, TC456619, TC397291, TC411908, CA598430, TC377373, TC380329, TC415365, EB512907, TC409343, TC390994, TC391143, TC428066, TC381463, TC419733, TC392074, TC403157, TC410066, TC382742, TC395303, TC405511, TC377190, TC443814, TC431879, TC423110, TC376606, TC422348, TC391613, TC385365, TC430544, TC401210, DR737360, TC379357, TC387135, TC397885, TC411684, TC388049, TC438587, TC381923, BJ309186, TC374485, TC393960, TC426358, TC409077, TC443387, TC388566, TC415083, TC399245, TC373429, TC390630, TC386535, GH729256, TC418716, CA605200, TC386519, TC369655, TC383047, TC402072, TC390402, TC374726, TC370158, TC401260, TC417992, TC417341, TC394307, TC388665, TC373583, TC406371, TC389589, TC398592, TC435034, TC370106, TC381988, TC452762, TC405475, TC386344, TC373637, TC370044, CD878039, TC435595, TC374164, TC386422, TC442623, TC418845, TC384071, TC414606, TC412150, TC388822, TC384592, TC429771, TC386639, TC416658, TC381957, TC384678, TC396650, TC379965, TC374230, TC391913, TC375834, TC393948, TC392109, TC371970, TC375726, TC400638, TC393554, TC407614, TC400477, TC459656, TC391007, TC379436, TC460615, TC403573, TC411941, TC440632, TC403929, TC457991, TC395841, TC394118, TC449043, TC392247, TC379635, TC387116, TC373259, TC433589, TC398343, TC418091, TC369664, TC389816, TC371387, TC372530, TC392323, TC384194, TC413066, TC371172, TC446092, TC439472, TC372167, TC391641, TC461622, TC400056, TC379470, TC403588, TC381817, CN013096, TC385659, TC376933, TC375798, TC382910, TC389451, TC394728, DR740372, TC403803, TC391122, TC440526, TC418850, TC401758, TC428944, TC417067, TC389661, TC381279, TC383820, TC379942, TC397258, CV775873, TC374404, TC396230, TC377766, TC451511, TC394965, TC384373, TC427210, TC416069, TC375313, TC398862, TC433162, TC372450, TC404978, TC389363, TC434442, TC379338, TC380590, TC416442, TC411471, TC378878, TC387410, TC406264, TC397176, TC390792, TC406516, TC388950, TC419222, TC369371, TC410194, TC392875, TC435546, TC371145, TC384735, TC439904, TC405540, TC390569, TC419057, TC391948, TC386895, BQ294582, TC398714, TC373791, TC381619, TC440693, TC394028, TC398970, TC457126, TC390904, TC403679, TC403580, TC413427, TC401915, TC424044, TC433957, TC423374, TC421954, TC385710, TC423239, TC425821, DR739994, TC459193, TC377021, TC394206, TC406236, TC382080, TC409599, TC391128, TC391962, TC445767, TC404636, TC446038, TC423804, TC379853, TC413199, TC370315, TC449504, TC418685, TC405440, TC403968, CK213497, CJ792862, TC414899, TC370885, TC422789, TC436347, TC391742, TC440819, TC422691, TC398514, TC416492, TC391900, TC377225, TC392033, TC412520, TC403977, TC406807, CD892838, TC395090, TC404606, CA646741, TC416906, TC434820, TC379711, TC416438, TC377308, TC379903, CA595837, TC423265, TC392272, TC372744, TC401244, CA730421, DR739350, TC417388, TC440066, TC386313, TC413339, CK208222, TC375539, TC408907, TC369844, TC420579, CK211707, TC386963, TC392126, CK163367, TC412732, TC431201, CA700201, TC396751, TC370350, CK211589, TC383909, TC376527, TC388718, TC419584, TC383701, TC390373, TC385780, TC378568, TC374392, TC405784, TC372175, TC369199, TC419747, TC411350, TC402186, TC405356, TC403264, TC423354, TC369348, TC413043, TC374268, CK214224, TC368603, TC384939, TC398040, TC376263, BE585841, CA709177, TC429374, TC409187, TC384010, TC416169, TC425690, TC423182, TC381218, TC408229, TC409190, TC383677, TC435224, TC384344, TC403537, TC392297, CV781430, TC388520, TC379422, TC411191, TC387683, TC434831, TC426326, TC375431, TC391995, TC415501, TC387064, TC373914, TC450285, TC392303, TC369899, TC394796, TC372330, TC448051, TC420057, TC397286, CA611770, TC435533, TC395966, TC397994, CF554444, TC414243, TC451276, TC403234, TC415685, CJ692089, TC371738, TC388914, TC387621, TC377438, TC398633, TC394716, TC369633, TC460689, TC378271, TC369064, TC380433, TC432154, TC420735, TC435281, TC422922, TC404371, TC397964, TC427405, TC373615, TC449256, TC384219, TC397562, TC416685, TC372664, TC389375, TC404413, TC370114, TC406038, TC408192, TC376874, TC424154, TC459245, CK210754, TC369928, TC370727, TC417769, TC449235, TC410147, TC376490, TC384454, TC411287, TC386237, TC393904, TC388751, TC387191, TC399471, TC380416, CV780698, TC434396, TC398536, TC421880, TC386414, TC400755, TC403986, TC372701, TC440636, TC406193, TC405615, TC376420, TC458987, TC383763, TC422851, TC404843, TC384553, CD876572, TC434140, TC382737, TC370633, TC369736, TC390135, TC386279, TC424376, TC376774, TC411480, TC420394, CK197833, TC432320, TC391785, TC382786, TC409650, TC439225, TC380662, TC388649, TC430561, TC395298, TC389162, TC382830, DR739471, CD905784, TC402534, CK212850, TC390285, TC369726, TC399636, TC407340, TC406584, TC417480, TC402440, TC455676, TC391411, TC409208, TC375146, TC432205, TC375813, CK199175, TC454407, TC384122, TC394661, TC381963, TC383176, TC430821, TC371455, TC449463, TC371037, TC374879, TC385701, TC403328, TC388688, TC439939, TC379459, TC400181, TC452503, TC388547, CK207939, TC396365, TC377496, TC368549, TC388976, TC379069, TC387133, TC410078, TC423576, TC397500, TC375530, TC368591, TC402668, TC393561, TC435799, TC393692, TC386322, TC391671, TC373787, TC388691, TC397415, TC382928, TC438243, TC371008, TC375918, TC417363, TC396487, TC387710, TC413700, TC385515, TC380882, TC423880, TC440321, TC451285, TC372580, TC377410, TC396083, TC393820, TC451519, CK211469, CB307332, CJ930688, TC404842, TC435808, TC416981, BE586004, TC397729, TC399408, TC444402, TC411817, TC426743, TC436290, TC396895, TC382342, TC393106, TC390489, TC407183, TC432504, TC397676, TC396460, TC431306, TC399124 |
| [Chromatin modification](http://amigo.geneontology.org/cgi-bin/amigo/go.cgi?action=query&view=query&query=GO:0016568&search_constraint=terms) | TC394716, TC395723, TC440636, TC402308, TC441343, TC393692, TC394796, CK211469, TC405030, CK211589, TC388403, TC381817, TC413571, TC389718, TC384219, TC411287, TC379357, TC383701, TC386279, CK214224, TC384735, TC419057, TC408907, TC391438, TC386639, CJ550278, TC433589, TC402186, TC393960 |
| [Covalent chromatin modification](http://amigo.geneontology.org/cgi-bin/amigo/go.cgi?action=query&view=query&query=GO:0016569&search_constraint=terms) | TC394716, TC395723, TC440636, TC402308, TC441343, TC393692, TC394796, CK211469, TC405030, CK211589, TC388403, TC381817, TC413571, TC389718, TC384219, TC411287, TC379357, TC386279, CK214224, TC384735, TC419057, TC408907, TC391438, TC386639, CJ550278, TC433589, TC402186, TC393960 |
| [Histone modification](http://amigo.geneontology.org/cgi-bin/amigo/go.cgi?action=query&view=query&query=GO:0016570&search_constraint=terms) | TC394716, TC395723, TC440636, TC402308, TC441343, TC393692, TC394796, CK211469, TC405030, CK211589, TC388403, TC381817, TC413571, TC389718, TC384219, TC411287, TC379357, TC386279, CK214224, TC384735, TC419057, TC408907, TC391438, TC386639, CJ550278, TC433589, TC402186, TC393960 |
| [Positive regulation of exit from mitosis](http://amigo.geneontology.org/cgi-bin/amigo/go.cgi?action=query&view=query&query=GO:0031536&search_constraint=terms) | TC400362, TC393960, TC423110, TC404413 |
| [Cellular response to heat](http://amigo.geneontology.org/cgi-bin/amigo/go.cgi?action=query&view=query&query=GO:0034605&search_constraint=terms) | TC423354, TC403588, TC461622, TC380882, TC423880 |
| [Phosphorylation](http://amigo.geneontology.org/cgi-bin/amigo/go.cgi?action=query&view=query&query=GO:0016310&search_constraint=terms) | CA646741, TC374230, TC386040, TC402308, TC393692, TC394796, CA632212, TC402603, TC459656, TC394965, TC411287, TC386519, TC374240, TC411941, TC369655, TC391143, TC457991, TC433162, TC409599, TC391438, TC370912, TC373259, TC433589, TC370315, TC386414, TC394716, TC398633, TC440636, TC395723, TC380063, TC376420, TC372167, TC393830, TC380943, TC386344, CK211469, TC405030, TC378054, TC381963, TC388403, TC383176, TC404371, TC435595, TC389718, TC384219, TC429747, TC385780, TC384688, TC387683, TC407076, TC419057, TC382342, TC388976, TC373914, TC406807, TC432504, TC386639, CD892838, CJ550278, TC397676, TC402186, TC395090, TC381957, TC382045 |
| [Regulation of cell communication](http://amigo.geneontology.org/cgi-bin/amigo/go.cgi?action=query&view=query&query=GO:0010646&search_constraint=terms) | TC393100, TC378790, TC382928, CV775873, TC449724, TC413571, TC417363, TC386237, TC386313, TC393904, TC398052, TC425841, TC387710, TC387579, TC394459, TC384738, CK211707, TC425690, TC386963, TC388410, TC377410, TC381218, TC395303, TC400755, TC376420, TC458987, TC393820, TC397312, CK211589, TC401210, TC386961, TC390135, TC405041, TC397729, TC377225, TC407076, TC399919, TC407572, TC372175, TC396365, TC381462, TC419747, TC401758, TC392263, TC389661, TC404606, TC382045 |
| [Response to xenobiotic stimulus](http://amigo.geneontology.org/cgi-bin/amigo/go.cgi?action=query&view=query&query=GO:0009410&search_constraint=terms) | TC405511, TC423354, TC403588, TC380882, TC423880 |
| [Nucleoside triphosphate catabolic process](http://amigo.geneontology.org/cgi-bin/amigo/go.cgi?action=query&view=query&query=GO:0009143&search_constraint=terms) | TC459656, GH732878, TC406807, TC454407, TC457112, CJ944525 |
| [Positive regulation of biological process](http://amigo.geneontology.org/cgi-bin/amigo/go.cgi?action=query&view=query&query=GO:0048518&search_constraint=terms) | TC410352, TC386040, TC373613, TC390393, TC397258, TC375864, TC445166, TC435533, TC374240, TC387861, CA710880, TC425841, TC433162, TC371242, TC423252, TC397660, TC393814, TC389363, TC388914, TC394716, TC398633, TC421914, TC446235, TC387410, TC380063, TC402545, TC378271, TC393830, TC380943, TC380433, TC378054, TC420735, TC404371, TC395453, TC384219, TC390569, TC416685, TC372664, TC407076, TC419057, TC403872, TC389090, TC389375, TC404413, TC382655, CK200433, TC373994, TC370114, BQ294582, TC406038, CJ550278, TC393970, TC395351, TC403580, TC401915, TC369928, CA632212, DR739994, TC384454, TC411287, TC387191, TC391143, CK217367, TC379853, TC386414, TC440636, TC395723, TC405440, TC376420, TC377190, TC423110, TC389718, DR737360, TC379357, TC386279, TC381943, BQ607161, TC393960, TC372845, TC382045, DR739471, TC416906, TC402308, TC434820, TC443387, TC379711, TC379903, TC378790, TC392272, TC417077, TC407340, TC440066, TC386519, CV759879, TC408907, TC402440, TC391438, TC417341, TC394307, TC375146, TC398592, TC389589, TC452762, TC386344, TC392778, TC405030, TC381963, CK211589, TC388403, TC383176, TC383909, TC449463, TC386422, TC388688, TC385780, TC388822, TC377496, TC388976, TC406708, TC429771, TC409459, TC423576, TC386639, TC402186, TC416658, TC374230, TC393561, TC393692, TC391671, TC393554, TC407614, TC402603, TC438243, TC449724, TC413571, TC379436, CA729339, TC460615, TC417363, CK214224, TC394118, TC380882, TC425690, TC433589, TC389816, TC399352, TC372530, CA613620, TC393820, TC461622, CK211469, TC400362, TC403588, TC432185, TC396895, TC434831, TC426326, TC382342, TC393106, TC373914, TC432504, TC397676, TC396460, TC428944 |
| [Transport](http://amigo.geneontology.org/cgi-bin/amigo/go.cgi?action=query&view=query&query=GO:0006810&search_constraint=terms) | TC410352, TC404158, TC373958, CK201269, CV775873, TC372677, TC429341, TC374240, TC398052, TC425841, TC397994, TC387579, TC394459, TC371242, TC384738, TC423252, TC397660, TC404978, TC389363, TC388914, TC379171, TC388410, TC387621, TC458562, TC411471, TC408309, TC446235, TC387410, TC432154, TC386396, TC420735, TC395453, TC386961, TC406594, TC397562, TC390569, TC369182, TC416685, TC387344, TC407076, TC403872, TC418365, TC389090, TC389375, TC373002, TC382655, CK200433, TC398714, TC370114, TC406038, CJ550278, TC393970, TC395351, TC392709, TC401915, TC456619, TC398304, TC393100, TC433957, CK210754, TC412569, CV771134, DR739994, TC384454, TC386237, TC394916, TC409343, TC390994, TC378274, TC411784, CK217367, TC409599, TC378153, TC423804, TC379853, TC370315, TC395303, TC400755, TC405511, TC418685, TC395723, TC405440, TC376420, TC458987, TC391621, TC391742, TC390135, TC398514, DR737360, TC377225, TC388049, TC409650, TC380662, TC398379, TC418928, BQ607161, TC406807, TC382045, TC404606, CA646741, DR739471, TC434820, TC443387, TC379711, TC378790, TC390630, TC392272, TC377061, TC418716, TC386313, TC383047, TC402440, CA606693, TC394307, CK211707, TC375146, TC412317, TC386963, TC373583, TC412212, TC389589, TC392778, TC396751, CK211589, TC370044, CA615187, TC373678, TC386422, TC388688, TC442623, TC452503, TC388547, TC405784, TC412150, TC399919, TC407572, TC372175, TC388822, TC396365, TC377496, TC406708, TC419747, TC429771, TC409459, TC423576, TC416658, TC384678, TC386646, TC374230, TC393561, TC393948, TC435799, TC391671, TC393554, TC407614, TC459656, TC382928, TC423354, TC441241, TC449724, TC413571, CA729339, TC417363, TC387710, TC403929, TC385515, BE585841, TC380882, TC423880, TC425690, TC398343, TC389816, TC422425, TC372530, TC384344, TC393820, TC435808, TC403588, TC385659, TC376933, TC382910, TC444402, TC394728, TC411191, TC434831, TC396895, TC426326, TC393106, TC377749, TC401758, TC396460, TC431306, TC428944, TC389661 |
| [Camera-type eye development](http://amigo.geneontology.org/cgi-bin/amigo/go.cgi?action=query&view=query&query=GO:0043010&search_constraint=terms) | TC405511, TC423354, TC403588, TC380882, TC423880 |
| [Camera-type eye morphogenesis](http://amigo.geneontology.org/cgi-bin/amigo/go.cgi?action=query&view=query&query=GO:0048593&search_constraint=terms) | TC405511, TC423354, TC403588, TC380882, TC423880 |
| [Positive regulation of cell cycle process](http://amigo.geneontology.org/cgi-bin/amigo/go.cgi?action=query&view=query&query=GO:0090068&search_constraint=terms) | TC400362, TC393960, TC423110, TC404413 |
| [Nucleotide catabolic process](http://amigo.geneontology.org/cgi-bin/amigo/go.cgi?action=query&view=query&query=GO:0009166&search_constraint=terms) | TC459656, GH732878, TC406807, TC454407, TC457112, CJ944525 |
| [Organ morphogenesis](http://amigo.geneontology.org/cgi-bin/amigo/go.cgi?action=query&view=query&query=GO:0009887&search_constraint=terms) | TC386040, TC402308, TC441343, TC393692, TC390393, TC378790, TC379903, TC397258, TC423354, TC402603, TC417077, TC435533, TC386519, CV759879, CA710880, TC391143, TC433162, TC391438, TC380882, TC423880, TC371242, TC393814, TC433589, TC386414, TC394716, TC398633, TC405511, TC440636, TC395723, TC452762, TC380063, TC376420, TC393830, TC380943, TC423110, TC386344, CK211469, TC405030, TC378054, TC381963, TC388403, TC420735, TC383176, TC403588, TC389718, TC375798, TC384219, TC384735, TC385780, TC407076, TC419057, TC381943, TC382342, TC388976, TC370114, TC409459, TC373914, TC432504, TC386639, CJ550278, TC397676 |
| [Nucleobase, nucleoside, nucleotide and nucleic acid catabolic process](http://amigo.geneontology.org/cgi-bin/amigo/go.cgi?action=query&view=query&query=GO:0034655&search_constraint=terms) | TC459656, GH732878, TC406807, TC454407, TC457112, CJ944525 |
| [Nucleobase, nucleoside and nucleotide catabolic process](http://amigo.geneontology.org/cgi-bin/amigo/go.cgi?action=query&view=query&query=GO:0034656&search_constraint=terms) | TC459656, GH732878, TC406807, TC454407, TC457112, CJ944525 |
| [G1 phase of mitotic cell cycle](http://amigo.geneontology.org/cgi-bin/amigo/go.cgi?action=query&view=query&query=GO:0000080&search_constraint=terms) | TC460615, TC400362, TC374230, TC370114, TC393960, TC407340, TC382045, TC404413 |
| [G1 phase](http://amigo.geneontology.org/cgi-bin/amigo/go.cgi?action=query&view=query&query=GO:0051318&search_constraint=terms) | TC460615, TC400362, TC374230, TC370114, TC393960, TC407340, TC382045, TC404413 |
| R[reproduction](http://amigo.geneontology.org/cgi-bin/amigo/go.cgi?action=query&view=query&query=GO:0000003&search_constraint=terms) | TC461921, TC410352, TC386040, TC373613, TC407978, TC390393, TC394796, TC397258, TC375864, TC397286, TC460760, TC451511, TC445166, TC427210, TC435533, TC374240, TC387861, TC425841, TC400388, CF554444, TC433162, TC371242, TC406870, TC423252, TC397660, TC393814, TC389363, TC388914, TC379171, CK203550, TC387621, TC416442, TC394716, TC398633, TC421914, TC446235, TC406264, TC387410, TC380063, TC393830, TC380943, TC380433, TC378054, TC420735, TC404371, TC448471, TC395453, TC384219, TC392875, TC435546, TC384735, TC390569, TC405540, TC416685, TC372664, TC407076, TC419057, TC403872, TC425847, TC389090, TC389375, CJ727624, TC382655, CK200433, TC373994, BQ294582, TC370114, TC406038, CJ550278, TC393970, TC398970, TC395351, TC403580, TC413427, TC411128, TC401915, TC456619, TC425878, TC412569, CA632212, TC410147, DR739994, TC384454, TC459193, TC411287, CA598430, TC409343, TC394206, TC394820, TC406236, TC418073, TC417308, TC391143, CK217367, TC391128, TC380416, TC445767, TC423804, TC413460, TC381463, TC379853, TC403157, BQ609416, TC386414, TC449504, TC395723, TC440636, TC405440, CJ792862, TC376420, TC431879, TC404843, CD876572, TC385233, TC389718, TC404052, TC379357, TC387135, TC386279, TC397885, TC411480, TC409650, TC381943, TC432001, BQ607161, TC406807, TC372845, TC382045, TC389162, DR739471, TC416906, TC402308, TC434820, TC443387, TC441343, TC379711, TC400330, TC388566, TC378790, TC379903, CA595837, TC390630, TC386535, GH729256, TC392272, TC390285, TC377061, CA730421, TC417077, TC446465, TC440066, TC386519, CV759879, TC408907, TC402440, TC391438, TC391411, TC394307, TC375146, TC389589, TC452762, TC386344, TC392778, TC370350, TC405030, TC381963, CK211589, TC388403, TC383176, TC370044, CD878039, TC383909, TC449463, TC376527, TC386422, TC403328, TC388688, TC383701, TC418845, TC414606, TC385780, TC388822, TC377496, TC388976, TC406708, TC429771, TC423576, TC409459, TC386639, TC410074, TC402186, TC416658, TC386646, CV763657, TC393561, TC375834, TC393948, TC393692, TC391671, TC393554, TC407614, TC403264, TC459656, TC402603, TC438243, TC449724, TC413571, TC379436, CA729339, TC417363, TC403573, CK214224, BQ239045, TC413700, CA709177, TC394118, TC449043, TC440321, TC425690, TC433589, TC399352, TC389816, TC408229, TC372530, CA613620, TC393820, CK211469, TC398805, TC369092, CV781430, TC416981, TC434831, TC396895, TC432185, TC426326, TC382342, CD882425, TC375431, TC393106, TC373914, TC432504, TC396460, TC397676, TC450285, TC428944, TC417067, TC396529 |
| [Phosphorus metabolic process](http://amigo.geneontology.org/cgi-bin/amigo/go.cgi?action=query&view=query&query=GO:0006793&search_constraint=terms) | CA646741, TC386040, TC402308, TC394796, TC394965, TC386519, TC374240, TC369655, TC433162, TC391438, TC370912, TC394716, TC398633, TC380063, TC393830, TC380943, TC386344, TC378054, TC405030, TC381963, TC388403, TC383176, TC404371, TC435595, TC384219, TC385780, TC384688, TC407076, TC419057, TC388976, TC370114, TC386639, CJ550278, TC402186, TC381957, TC374230, TC393692, CA632212, TC459656, TC402603, TC411287, TC411941, TC457991, TC391143, TC409599, TC373259, TC433589, TC386414, TC370315, TC395723, TC440636, TC372167, TC376420, CK211469, TC389718, TC429747, TC387683, TC409650, TC382342, TC397676, CD892838, TC432504, TC406807, TC373914, TC395090, TC382045 |
| [Phosphate metabolic process](http://amigo.geneontology.org/cgi-bin/amigo/go.cgi?action=query&view=query&query=GO:0006796&search_constraint=terms) | CA646741, TC386040, TC402308, TC394796, TC394965, TC386519, TC374240, TC369655, TC433162, TC391438, TC370912, TC394716, TC398633, TC380063, TC393830, TC380943, TC386344, TC378054, TC405030, TC381963, TC388403, TC383176, TC404371, TC435595, TC384219, TC385780, TC384688, TC407076, TC419057, TC388976, TC370114, TC386639, CJ550278, TC402186, TC381957, TC374230, TC393692, CA632212, TC459656, TC402603, TC411287, TC411941, TC457991, TC391143, TC409599, TC373259, TC433589, TC386414, TC370315, TC395723, TC440636, TC372167, TC376420, CK211469, TC389718, TC429747, TC387683, TC409650, TC382342, TC397676, CD892838, TC432504, TC406807, TC373914, TC395090, TC382045 |
[truncated: 291,711 more chars]
