# Supplementary material for: Global Analysis of Differentially Expressed Genes and Proteins in the Wheat Callus Infected by Agrobacterium tumefaciens
Source: PLoS One. 2013 Nov 20;8(11):e79390. doi: 10.1371/journal.pone.0079390 (PMC3835833; doi:10.1371/journal.pone.0079390)
Supplement: File S9 — Distribution of all GEGs on the pathways. (DOC) [file pone.0079390.s009.doc]

**File S9 Distribution of all GEGs on the pathways**

| **#** | **Pathway** | **Differentially expressed genes** |
| --- | --- | --- |
| 1 | [DNA replication](../../../../E:%5C实验%5Cdata%5Cdata%5Crnaseq%5Cwheat.tar%5Cupload%5CGeneDiffExp%5CPathway%5CW-VS-I_map%5Cmap03030.html) | TC395357, TC451347, TC371341, TC369535, TC393846, BQ238080, CA639940, TC389074, TC419297, CK210851, TC399008, BJ286631, TC401637, CA635612, TC369092, TC380584, TC389162, TC371386, TC398458, TC448152, TC377061, TC371455, TC407662, TC460031, TC408462, TC370344, CJ815867, TC371125, TC390369, TC458892, TC369548, CK208222, TC384657, NP821542, TC381031, TC370486, TC406193, TC420274, TC374164, TC375864, TC424396 |
| 2 | [ABC transporters](../../../../E:%5C实验%5Cdata%5Cdata%5Crnaseq%5Cwheat.tar%5Cupload%5CGeneDiffExp%5CPathway%5CW-VS-I_map%5Cmap02010.html) | AL821103, CV781430, TC450855, TC447417, CV760609, DR734255, TC428930, TC381634, DR733545, TC381497, TC424169, CA499624, CA501082, CD929875, TC455532, TC417970, TC428026, TC457217, CJ689844, TC394078, TC453926, TC418691, TC378225, TC377749, TC378383, TC415808, TC414133, TC413302, TC384071, CD866864 |
| 3 | [Base excision repair](../../../../E:%5C实验%5Cdata%5Cdata%5Crnaseq%5Cwheat.tar%5Cupload%5CGeneDiffExp%5CPathway%5CW-VS-I_map%5Cmap03410.html) | BE500080, TC451347, TC387538, TC374879, TC370962, TC383570, TC376800, TC375593, CA639940, TC419297, BJ229502, BJ286631, CA635612, BJ208576, TC396928, TC423248, TC448152, TC377061, TC430197, TC407919, TC407662, TC408462, TC405411, TC371125, CK208222, TC384657, TC406193, TC375864, TC424396 |
| 4 | [Caffeine metabolism](../../../../E:%5C实验%5Cdata%5Cdata%5Crnaseq%5Cwheat.tar%5Cupload%5CGeneDiffExp%5CPathway%5CW-VS-I_map%5Cmap00232.html) | TC454121, BG909274, BJ281583, BE403954, TC440508, CA695289, TC372701, BJ258498, CJ694502, TC427880, TC400125, TC454526 |
| 5 | [Purine metabolism](../../../../E:%5C实验%5Cdata%5Cdata%5Crnaseq%5Cwheat.tar%5Cupload%5CGeneDiffExp%5CPathway%5CW-VS-I_map%5Cmap00230.html) | EB512907, CJ628257, TC454121, BJ256335, TC401715, TC371172, BG909274, BJ281583, BE403954, BQ167150, BQ609463, BE517280, TC440508, CA695289, TC369899, TC455080, TC376888, TC444588, CJ930688, CF133957, TC372701, TC443814, BJ258498, TC444921, TC402080, CD874875, TC425623, BE516446, CA499357, TC419621, CJ694502, TC427880, TC454714, TC399245, BE422762, TC378782, TC400125, TC409190, TC454526, TC368549, TC400401, TC384553, TC427537, TC419297, TC391962, TC399008, CA635612, TC420502, CD935065, TC397176, TC398458, TC397500, CJ813329, CK155765, TC418683, TC425830, TC408462, TC376820, CJ815867, TC371125, TC410041, TC458892, TC374447, TC369548, TC384657, CA602703, TC374709, TC406505, TC406193, TC380402, TC383739, TC373775, TC392232 |
| 6 | [Nucleotide excision repair](../../../../E:%5C实验%5Cdata%5Cdata%5Crnaseq%5Cwheat.tar%5Cupload%5CGeneDiffExp%5CPathway%5CW-VS-I_map%5Cmap03420.html) | BQ239662, TC417152, CA604422, TC415329, TC395357, TC396262, TC417939, TC412468, TC451347, TC369535, TC393846, TC381899, CA639940, TC419297, BJ286631, CA635612, TC369092, BJ217586, TC380584, TC389162, TC448152, TC377061, TC371455, TC460031, TC408462, TC371125, TC419920, CK208222, TC384657, TC394820, TC381031, TC412777, TC406193, TC401132, TC374164, TC424396 |
| 7 | [Mismatch repair](../../../../E:%5C实验%5Cdata%5Cdata%5Crnaseq%5Cwheat.tar%5Cupload%5CGeneDiffExp%5CPathway%5CW-VS-I_map%5Cmap03430.html) | TC395357, TC457961, TC451347, TC369535, TC393846, CA639940, TC415306, BJ286631, CA635612, TC369092, TC380584, TC389162, TC448152, TC377061, TC371455, TC460031, TC371125, CK208222, TC384657, TC381031, TC374164, TC424396 |
| 8 | [alpha-Linolenic acid metabolism](../../../../E:%5C实验%5Cdata%5Cdata%5Crnaseq%5Cwheat.tar%5Cupload%5CGeneDiffExp%5CPathway%5CW-VS-I_map%5Cmap00592.html) | CK161463, TC397738, TC408041, TC425657, BE586132, TC400224, TC410376, CA733413, TC406579, TC388136, TC416007, TC451272, TC388505, TC393437, TC379965, TC397909, TC407672, CJ854725, CK162453, CJ854095, TC430544, TC370633, TC409187, TC433422, TC391946, TC392033, TC456784, TC375726, TC413124, TC389993, TC407490, TC398536, TC431198, TC400562, BJ283132, TC421162, TC380416, TC411908, TC415336 |
| 9 | [RNA polymerase](../../../../E:%5C实验%5Cdata%5Cdata%5Crnaseq%5Cwheat.tar%5Cupload%5CGeneDiffExp%5CPathway%5CW-VS-I_map%5Cmap03020.html) | EB512907, CJ628257, BJ256335, BQ167150, BE517280, TC443814, TC425623, BE516446, CA499357, BE422762, TC368549, TC400401, TC427537, TC420502, TC397176, TC397500, TC418683, TC425830, TC383739, TC392232 |
| 10 | [Glutathione metabolism](../../../../E:%5C实验%5Cdata%5Cdata%5Crnaseq%5Cwheat.tar%5Cupload%5CGeneDiffExp%5CPathway%5CW-VS-I_map%5Cmap00480.html) | TC419727, BE586004, TC451188, TC371738, TC374098, TC383068, CK197833, NP637334, TC369687, BJ268303, TC411858, CA683961, TC429724, TC390319, TC427986, TC396487, TC371357, TC371600, TC399986, TC408869, CK199846, TC449256, CD875411, TC412532, TC394253, TC428351, CK217741, TC440499, TC378069, TC435803, TC384947, TC380281, TC382802, TC405652, TC396007, TC408608, TC378783, CK207939, TC398209, TC383235, TC432678, CJ868963, DR734904, TC397793, TC378846, TC387501, TC397019, TC378782, TC391648, TC376202, TC374461, TC439423, TC375234, TC400477, TC394670, TC439324, EB512741, TC377064, TC378409, TC378437, TC379161, EB512568, TC376820, TC389590, CJ625718 |
| 11 | [Zeatin biosynthesis](../../../../E:%5C实验%5Cdata%5Cdata%5Crnaseq%5Cwheat.tar%5Cupload%5CGeneDiffExp%5CPathway%5CW-VS-I_map%5Cmap00908.html) | TC458205, TC379055, CF554463, CV775491, CV770684, TC445333, CD929101, TC369415, TC439939, TC457168, TC452225, TC386475, TC404914, CJ854095, TC389137, TC436347, TC426293, TC404179, TC385927, TC400164, TC421162, TC392363, TC396723 |
| 12 | [Sulfur metabolism](../../../../E:%5C实验%5Cdata%5Cdata%5Crnaseq%5Cwheat.tar%5Cupload%5CGeneDiffExp%5CPathway%5CW-VS-I_map%5Cmap00920.html) | TC419796, TC435231, CK210510, TC401715, TC397374, TC369899, TC373702, TC455080, TC394589, TC376351, TC402080, TC434442, TC382786, TC454714, TC399245, TC456755, TC384553 |
| 13 | [RNA degradation](../../../../E:%5C实验%5Cdata%5Cdata%5Crnaseq%5Cwheat.tar%5Cupload%5CGeneDiffExp%5CPathway%5CW-VS-I_map%5Cmap03018.html) | CJ630817, CD491698, TC452969, TC426663, TC418924, TC379386, CJ783546, TC461705, CD876014, CJ664158, TC375313, BQ743232, CA594144, TC392062, TC405849, TC457485, TC395563, CA661298, TC404388, GH722941, CJ882281, TC369762, TC391423, TC384499, TC438189, TC387710, TC404606, TC443035, TC430325, TC418747, TC380608, TC402715, TC377559, TC382129, CN010553, TC380492, TC399136, TC398730 |
| 14 | [Pyrimidine metabolism](../../../../E:%5C实验%5Cdata%5Cdata%5Crnaseq%5Cwheat.tar%5Cupload%5CGeneDiffExp%5CPathway%5CW-VS-I_map%5Cmap00240.html) | EB512907, CJ628257, BJ256335, BQ167150, BE517280, TC407325, TC421363, CJ707239, TC443814, TC425623, TC410126, BE516446, CA499357, BE422762, TC378782, TC390285, TC394661, TC368549, TC445212, TC400401, TC427537, TC419297, TC462041, TC454407, TC399008, BE424562, CA635612, TC420502, TC419181, TC397176, TC398458, GH732878, TC397500, TC418683, TC425830, TC408462, TC457112, TC376820, CJ815867, TC371125, TC458892, TC369548, TC384657, CA602703, TC406193, TC383739, TC373775, TC392232 |
| 15 | [Endocytosis](../../../../E:%5C实验%5Cdata%5Cdata%5Crnaseq%5Cwheat.tar%5Cupload%5CGeneDiffExp%5CPathway%5CW-VS-I_map%5Cmap04144.html) | TC442697, CK209594, TC413061, CV775489, TC383174, CA730648, CN010468, BE418867, TC441472, TC393625, BE517058, CJ690976, TC460259, TC424266, CA733951, TC394851, TC372020, TC458283, TC461622, TC370931, BE427240, TC425803, TC380882, TC423537, TC397964, TC431259, TC423880, TC372389, TC413753, TC402426, EV253937, TC380058, TC421376, TC400136, TC423354, TC403588, TC401124, TC405511, TC437004, BE517532, TC371546, BJ267564, TC379334, TC426518, TC431306, TC461536, TC372530, TC427621, TC381561, TC384979 |
| 16 | [Phenylalanine metabolism](../../../../E:%5C实验%5Cdata%5Cdata%5Crnaseq%5Cwheat.tar%5Cupload%5CGeneDiffExp%5CPathway%5CW-VS-I_map%5Cmap00360.html) | BE414946, TC418073, TC369189, TC422841, TC442758, TC404452, TC385962, TC372867, TC375760, TC452793, TC381862, TC439472, TC376651, TC441915, TC396657, TC423533, TC448840, TC431175, TC387817, CA612260, TC372823, TC385013, TC429713, DR739303, DR739299, CA600984, TC384357, TC428778, TC378324, TC395069, CA702126, TC419550, TC398026, TC390594, TC459008, TC443038, CJ729932, TC386401, BE217043, TC404786, TC390762, TC422583, TC400388, TC405773, TC389044, TC391116, TC419090, TC426916, TC410159, TC420057, TC381068, TC372953, TC442727, TC385326, TC370392, TC382734, CV766937, TC371073, TC434510, TC403504, CJ521228, TC409987, TC434947, TC430788, TC399341, TC388627, TC395813, TC386944, TC444824, TC402423, TC407651, TC454508 |
| 17 | [Spliceosome](../../../../E:%5C实验%5Cdata%5Cdata%5Crnaseq%5Cwheat.tar%5Cupload%5CGeneDiffExp%5CPathway%5CW-VS-I_map%5Cmap03040.html) | TC442697, TC451799, CV775489, TC434709, TC383174, CA702167, BE516477, BE418867, TC441472, TC450855, TC422645, TC457423, BE431108, TC410563, CD905784, DR733545, CA733951, CK208107, CJ695406, TC395764, TC458283, TC461622, BE427240, TC434469, TC424169, TC380882, TC426432, CA715649, TC400906, TC386097, TC455532, TC397964, TC417970, CV778006, TC431259, TC423880, BE498836, TC372389, TC392930, TC402529, TC452401, BJ261010, TC442577, EV253937, TC419081, CA500614, TC399352, TC441239, TC444765, TC444915, TC423354, TC403588, TC408524, TC450732, TC405511, TC447650, TC402112, TC415808, TC371546, TC415171, TC460115, TC387710, TC369628, TC397779, GH728721, CV771935, TC373429, TC404606, BQ161558, TC372288, BJ249812, TC382950, TC460842, TC456230, TC418101, TC413129, TC405540, TC410927, TC386381, TC447563, TC405219, TC402121, TC390323, TC388566, TC376220, TC387410, CJ826348, TC387135, TC398730, TC384085, TC387050, TC384374, TC385233, TC388317, TC402839 |
| 18 | Other glycan degradation (no map in kegg database) | TC431603, TC369373, TC436483, TC384877, TC369844, TC411008, TC398984, TC414916, TC386139 |
| 19 | [Galactose metabolism](../../../../E:%5C实验%5Cdata%5Cdata%5Crnaseq%5Cwheat.tar%5Cupload%5CGeneDiffExp%5CPathway%5CW-VS-I_map%5Cmap00052.html) | CV780016, TC434630, CV774255, TC393877, CK163142, TC406096, TC435431, BQ241509, TC430163, TC390016, CJ854427, TC388751, TC369510, TC421796, TC431603, TC393198, TC376490, TC379422, TC418269, TC423241, TC369373, TC436483, TC384877, GH726691, CK193510, TC388819, TC438376, TC411008, TC398984, TC406505, TC400690 |
| 20 | [Amino sugar and nucleotide sugar metabolism](../../../../E:%5C实验%5Cdata%5Cdata%5Crnaseq%5Cwheat.tar%5Cupload%5CGeneDiffExp%5CPathway%5CW-VS-I_map%5Cmap00520.html) | TC431382, TC383028, TC460907, DR740372, TC393523, TC460760, TC456197, TC385738, BQ238873, TC400721, BJ259409, TC377308, BJ270999, TC384010, TC419957, TC394567, TC401434, CK201148, TC446440, TC439821, TC373787, TC376490, TC403234, TC369199, TC405079, TC369807, CK201126, TC417670, TC457313, AL819012, TC459867, TC396981, TC446038, TC370384, TC456959, TC437555, GH726691, TC396650, TC388280, TC452779, TC369844, TC438376, CV777843, TC370885, TC372330, TC406505, TC375921, CA638808, TC405546 |
| 21 | [Non-homologous end-joining](../../../../E:%5C实验%5Cdata%5Cdata%5Crnaseq%5Cwheat.tar%5Cupload%5CGeneDiffExp%5CPathway%5CW-VS-I_map%5Cmap03450.html) | BJ300791, GH727050, TC407662, TC375864 |
| 22 | [Sphingolipid metabolism](../../../../E:%5C实验%5Cdata%5Cdata%5Crnaseq%5Cwheat.tar%5Cupload%5CGeneDiffExp%5CPathway%5CW-VS-I_map%5Cmap00600.html) | TC447848, TC449510, TC431603, TC369373, TC436483, TC384877, TC410093, TC424750, TC411008, TC398984, TC400690 |
| 23 | [Anthocyanin biosynthesis](../../../../E:%5C实验%5Cdata%5Cdata%5Crnaseq%5Cwheat.tar%5Cupload%5CGeneDiffExp%5CPathway%5CW-VS-I_map%5Cmap00942.html) | TC446065, DR734764 |
| 24 | [Homologous recombination](../../../../E:%5C实验%5Cdata%5Cdata%5Crnaseq%5Cwheat.tar%5Cupload%5CGeneDiffExp%5CPathway%5CW-VS-I_map%5Cmap03440.html) | BJ300791, TC391840, CJ728551, TC397422, TC395357, TC369535, TC393846, CA635612, TC380584, TC371455, TC460031, TC371125, TC384657 |
| 25 | [Phenylpropanoid biosynthesis](../../../../E:%5C实验%5Cdata%5Cdata%5Crnaseq%5Cwheat.tar%5Cupload%5CGeneDiffExp%5CPathway%5CW-VS-I_map%5Cmap00940.html) | BE414946, TC418073, TC369189, TC422841, TC442758, TC404452, TC382807, TC447579, TC385962, TC372549, TC386707, TC433013, TC372867, CV769814, TC375760, TC452793, CK164619, TC377135, CD929101, TC381862, TC386323, TC392875, TC439472, CA682500, TC376651, TC441915, TC396657, TC382080, TC423533, TC448840, TC431175, TC387817, CV780986, CA612260, TC372823, TC385013, TC429713, DR739303, DR739299, TC434396, TC384357, TC428778, EB514888, TC393857, TC378324, TC395069, TC392203, CA702126, TC435364, TC419550, TC398026, TC373133, TC390594, TC459008, TC404420, TC387064, CJ729932, TC386401, BE217043, TC404786, TC390762, TC422583, TC400388, TC405773, TC375124, TC389044, TC391116, TC419090, TC426916, TC410159, TC372658, TC381068, TC396781, TC372953, TC442727, TC391900, TC385326, TC429600, TC370392, TC382734, TC371073, TC434510, TC403504, CJ521228, TC409987, TC458697, TC434947, TC431814, TC445817, TC430788, TC377760, TC404410, TC399341, TC388627, TC400114, TC374409, TC444220, TC395813, TC386944, TC403549, TC402423, TC407651 |
| 26 | [Starch and sucrose metabolism](../../../../E:%5C实验%5Cdata%5Cdata%5Crnaseq%5Cwheat.tar%5Cupload%5CGeneDiffExp%5CPathway%5CW-VS-I_map%5Cmap00500.html) | TC458807, TC372549, DR733256, TC380772, NP234380, TC385909, CV764944, CV769814, TC434630, TC419305, CK201841, TC460760, TC399408, CV774255, TC393877, TC387751, TC446901, TC383362, CK163142, TC454758, TC385738, TC448275, BQ609093, TC406096, TC383964, BQ241509, TC383227, TC430163, TC392297, TC460397, TC373133, TC404420, BJ270999, TC387064, TC390016, TC419957, CJ854427, TC399841, CA647610, TC401749, TC401434, TC375124, TC375214, TC439821, TC461977, TC420201, TC391516, TC370347, TC453587, TC435984, TC403234, GH731123, TC418269, TC368548, AL827131, TC423241, BQ169564, TC431814, TC370384, TC452945, CA701904, TC404410, TC437555, GH726691, CK193510, TC461565, TC388280, TC431932, TC452779, TC388819, TC438376, TC444220, TC419483, TC421908, TC406505, TC375921, TC380825, TC405546, TC439229 |
| 27 | [Indole alkaloid biosynthesis](../../../../E:%5C实验%5Cdata%5Cdata%5Crnaseq%5Cwheat.tar%5Cupload%5CGeneDiffExp%5CPathway%5CW-VS-I_map%5Cmap00901.html) | TC420816, TC425291, AJ890242, TC426838, TC413990, TC453352 |
| 28 | [Cyanoamino acid metabolism](../../../../E:%5C实验%5Cdata%5Cdata%5Crnaseq%5Cwheat.tar%5Cupload%5CGeneDiffExp%5CPathway%5CW-VS-I_map%5Cmap00460.html) | TC415483, CF554463, TC372549, TC419796, CJ780546, CV769814, TC377135, CD929101, TC452225, TC456247, TC392283, CD882790, TC373702, CK196090, TC436832, TC373133, TC404420, TC387064, TC389137, TC376351, TC376758, TC404179, TC385927, TC375124, TC378601, TC431814, TC404410, TC444220, TC370791 |
| 29 | [Phosphatidylinositol signaling system](../../../../E:%5C实验%5Cdata%5Cdata%5Crnaseq%5Cwheat.tar%5Cupload%5CGeneDiffExp%5CPathway%5CW-VS-I_map%5Cmap04070.html) | CA635103, CN010468, TC436405, DR733517, BE517058, CJ655278, TC448907, TC399452, GH731884, CJ690976, TC460259, CA501474, TC423537, TC398396, TC402426, TC416750, TC418693, BJ267564, TC434894, TC409004, TC393970, TC403258 |
| 30 | [Ubiquitin mediated proteolysis](../../../../E:%5C实验%5Cdata%5Cdata%5Crnaseq%5Cwheat.tar%5Cupload%5CGeneDiffExp%5CPathway%5CW-VS-I_map%5Cmap04120.html) | TC452029, CA714086, CJ705412, BF483505, TC415626, TC404091, BE516558, TC432353, DR733422, CJ631281, BQ239662, TC420414, TC417152, TC429033, CA599823, TC415329, TC424532, TC395901, TC431875, TC396262, TC416566, TC385142, TC449093, TC418928, TC419081, TC445443, TC391916, TC417939, TC388649, BJ302404, TC405910, TC436889, TC423237, TC412194, TC400362, GH728721, TC457772, TC410343, TC393960, TC404413, TC400656, TC423110, TC383346, TC448929, TC391007, TC376774, TC371905, TC387170, TC379536, TC394820, TC412777 |
| 31 | [Glycosphingolipid biosynthesis - ganglio series](../../../../E:%5C实验%5Cdata%5Cdata%5Crnaseq%5Cwheat.tar%5Cupload%5CGeneDiffExp%5CPathway%5CW-VS-I_map%5Cmap00604.html) | TC431603, TC369844, TC411008 |
| 32 | [Nitrogen metabolism](../../../../E:%5C实验%5Cdata%5Cdata%5Crnaseq%5Cwheat.tar%5Cupload%5CGeneDiffExp%5CPathway%5CW-VS-I_map%5Cmap00910.html) | TC418073, TC369189, TC404452, TC394028, TC402340, TC452793, CA744836, TC381862, TC439472, TC390184, TC376651, TC441915, BG907133, TC423533, TC385013, DR739299, CA702126, TC419550, TC459008, TC386401, BE217043, TC390762, TC419090, TC442727, TC433844, TC376414, CK197746, TC406749, TC387191, TC388904, TC369346, TC407651 |
| 33 | [Glycosaminoglycan degradation](../../../../E:%5C实验%5Cdata%5Cdata%5Crnaseq%5Cwheat.tar%5Cupload%5CGeneDiffExp%5CPathway%5CW-VS-I_map%5Cmap00531.html) | TC431603, TC369844, TC411008 |
| 34 | [Selenoamino acid metabolism](../../../../E:%5C实验%5Cdata%5Cdata%5Crnaseq%5Cwheat.tar%5Cupload%5CGeneDiffExp%5CPathway%5CW-VS-I_map%5Cmap00450.html) | TC403036, TC419796, TC435231, CK210510, TC401715, TC397374, TC369899, TC373702, TC455080, TC394589, TC376351, TC402080, TC382365, TC382786, TC454714, TC399245, TC404719, TC456755, TC403477, TC384553 |
| 35 | [Inositol phosphate metabolism](../../../../E:%5C实验%5Cdata%5Cdata%5Crnaseq%5Cwheat.tar%5Cupload%5CGeneDiffExp%5CPathway%5CW-VS-I_map%5Cmap00562.html) | CA635103, CN010468, TC436405, DR733517, BE517058, CJ655278, TC399452, GH731884, CJ690976, TC460259, CA501474, TC423537, TC402426, TC416750, TC418693, BJ267564, CV767688 |
| 36 | [N-Glycan biosynthesis](../../../../E:%5C实验%5Cdata%5Cdata%5Crnaseq%5Cwheat.tar%5Cupload%5CGeneDiffExp%5CPathway%5CW-VS-I_map%5Cmap00510.html) | TC427632, TC445354, TC424671, TC406096, TC420020, BQ842471, TC391411, TC388232, TC381628, TC381434 |
| 37 | [Pantothenate and CoA biosynthesis](../../../../E:%5C实验%5Cdata%5Cdata%5Crnaseq%5Cwheat.tar%5Cupload%5CGeneDiffExp%5CPathway%5CW-VS-I_map%5Cmap00770.html) | TC395279, TC407325, TC413263, BG905284, TC374741, TC432369, TC384637, TC374948 |
| 38 | [Ether lipid metabolism](../../../../E:%5C实验%5Cdata%5Cdata%5Crnaseq%5Cwheat.tar%5Cupload%5CGeneDiffExp%5CPathway%5CW-VS-I_map%5Cmap00565.html) | TC424266, TC400136, TC401124, TC426518, TC395966, TC408586, TC430501 |
| 39 | [Aminoacyl-tRNA biosynthesis](../../../../E:%5C实验%5Cdata%5Cdata%5Crnaseq%5Cwheat.tar%5Cupload%5CGeneDiffExp%5CPathway%5CW-VS-I_map%5Cmap00970.html) | CA497112, TC382365, TC430995, TC385881, TC424621, TC383139, TC439904, TC406106, TC411658, TC428112, TC381548, TC447193, TC394439, TC385556, CK199725, TC425957, CA664929 |
| 40 | [Valine, leucine and isoleucine biosynthesis](../../../../E:%5C实验%5Cdata%5Cdata%5Crnaseq%5Cwheat.tar%5Cupload%5CGeneDiffExp%5CPathway%5CW-VS-I_map%5Cmap00290.html) | TC395279, TC430995, TC385881, TC413263, TC374741, TC432369, TC384637, CD911155, TC381548, TC447193, TC374948, TC394439 |
| 41 | [Vitamin B6 metabolism](../../../../E:%5C实验%5Cdata%5Cdata%5Crnaseq%5Cwheat.tar%5Cupload%5CGeneDiffExp%5CPathway%5CW-VS-I_map%5Cmap00750.html) | TC417347, TC434986 |
| 42 | [Linoleic acid metabolism](../../../../E:%5C实验%5Cdata%5Cdata%5Crnaseq%5Cwheat.tar%5Cupload%5CGeneDiffExp%5CPathway%5CW-VS-I_map%5Cmap00591.html) | CK161463, CA733413, TC388136, TC416007, TC451272, TC404914, TC407672, CJ854095, TC429062, TC389678, TC400379, BJ283132, TC421162, TC415336 |
| 43 | [Tropane, piperidine and pyridine alkaloid biosynthesis](../../../../E:%5C实验%5Cdata%5Cdata%5Crnaseq%5Cwheat.tar%5Cupload%5CGeneDiffExp%5CPathway%5CW-VS-I_map%5Cmap00960.html) | CA600984, TC443038, TC420057, CV766937, TC444824, TC371145, TC389586 |
| 44 | [Tryptophan metabolism](../../../../E:%5C实验%5Cdata%5Cdata%5Crnaseq%5Cwheat.tar%5Cupload%5CGeneDiffExp%5CPathway%5CW-VS-I_map%5Cmap00380.html) | TC397738, TC454121, CJ827429, TC414330, BG909274, BJ281583, BE403954, TC440508, CK196090, TC398514, TC408095, TC406264, BJ258498, TC421733, CJ694502, TC427880, TC400125, TC454526, TC447801, TC379241, TC385701, TC419215, TC431814, TC454251, TC457566, TC387386 |
| 45 | [C5-Branched dibasic acid metabolism](../../../../E:%5C实验%5Cdata%5Cdata%5Crnaseq%5Cwheat.tar%5Cupload%5CGeneDiffExp%5CPathway%5CW-VS-I_map%5Cmap00660.html) | TC395279, TC432369 |
| 46 | [Diterpenoid biosynthesis](../../../../E:%5C实验%5Cdata%5Cdata%5Crnaseq%5Cwheat.tar%5Cupload%5CGeneDiffExp%5CPathway%5CW-VS-I_map%5Cmap00904.html) | TC413199, CN013015, CJ625788, BJ247780, TC410108, TC438587, TC430821 |
| 47 | [Flavonoid biosynthesis](../../../../E:%5C实验%5Cdata%5Cdata%5Crnaseq%5Cwheat.tar%5Cupload%5CGeneDiffExp%5CPathway%5CW-VS-I_map%5Cmap00941.html) | CA663181, TC381923, TC442758, TC382807, TC447579, TC433013, TC392875, CA682500, TC413199, CN013015, TC424987, CD930656, TC431175, CA612260, EB514888, BJ247780, TC435364, TC382858, BQ247004, TC421733, TC410108, CJ826490, TC438587, TC400388, TC391116, TC392045, TC391564, TC372953, TC399442, TC370392, TC371073, TC377760, TC403549, TC419552 |
| 48 | [SNARE interactions in vesicular transport](../../../../E:%5C实验%5Cdata%5Cdata%5Crnaseq%5Cwheat.tar%5Cupload%5CGeneDiffExp%5CPathway%5CW-VS-I_map%5Cmap04130.html) | TC373848, TC370178, TC419314, TC396131, TC382829, TC385865, TC420460, TC380474, TC413118 |
| 49 | [Glycosphingolipid biosynthesis - globo series](../../../../E:%5C实验%5Cdata%5Cdata%5Crnaseq%5Cwheat.tar%5Cupload%5CGeneDiffExp%5CPathway%5CW-VS-I_map%5Cmap00603.html) | TC369844, TC400690 |
| 50 | [Lysine biosynthesis](../../../../E:%5C实验%5Cdata%5Cdata%5Crnaseq%5Cwheat.tar%5Cupload%5CGeneDiffExp%5CPathway%5CW-VS-I_map%5Cmap00300.html) | TC370507, TC404151, TC421880 |
| 51 | [Biotin metabolism](../../../../E:%5C实验%5Cdata%5Cdata%5Crnaseq%5Cwheat.tar%5Cupload%5CGeneDiffExp%5CPathway%5CW-VS-I_map%5Cmap00780.html) | TC432261 |
| 52 | [Folate biosynthesis](../../../../E:%5C实验%5Cdata%5Cdata%5Crnaseq%5Cwheat.tar%5Cupload%5CGeneDiffExp%5CPathway%5CW-VS-I_map%5Cmap00790.html) | TC416981, BE424562 |
| 53 | [Synthesis and degradation of ketone bodies](../../../../E:%5C实验%5Cdata%5Cdata%5Crnaseq%5Cwheat.tar%5Cupload%5CGeneDiffExp%5CPathway%5CW-VS-I_map%5Cmap00072.html) | TC398514, TC427138 |
| 54 | [Circadian rhythm - plant](../../../../E:%5C实验%5Cdata%5Cdata%5Crnaseq%5Cwheat.tar%5Cupload%5CGeneDiffExp%5CPathway%5CW-VS-I_map%5Cmap04712.html) | TC420579, TC392329, TC424987, TC402513, CA691990, TC401824, TC372402, TC403968, TC389823, CJ826490, TC380329, TC399232, CV780115, CD892838, TC403977, TC404926 |
| 55 | [Stilbenoid, diarylheptanoid and gingerol biosynthesis](../../../../E:%5C实验%5Cdata%5Cdata%5Crnaseq%5Cwheat.tar%5Cupload%5CGeneDiffExp%5CPathway%5CW-VS-I_map%5Cmap00945.html) | TC442758, TC382807, TC447579, TC433013, TC418873, TC392875, TC414330, CV782061, CD930656, TC405112, TC431175, CA612260, EB514888, TC383147, TC408095, TC372040, TC395566, TC421733, AL822449, TC400388, TC382795, TC391116, TC422550, TC372953, TC429600, TC370392, TC371073, TC427006, TC403549 |
| 56 | [Regulation of autophagy](../../../../E:%5C实验%5Cdata%5Cdata%5Crnaseq%5Cwheat.tar%5Cupload%5CGeneDiffExp%5CPathway%5CW-VS-I_map%5Cmap04140.html) | CD874590, CA501474, TC427710, TC391360, TC417924, TC384688, TC381957 |
| 57 | [Thiamine metabolism](../../../../E:%5C实验%5Cdata%5Cdata%5Crnaseq%5Cwheat.tar%5Cupload%5CGeneDiffExp%5CPathway%5CW-VS-I_map%5Cmap00730.html) | CA602703, TC409077 |
| 58 | [Isoquinoline alkaloid biosynthesis](../../../../E:%5C实验%5Cdata%5Cdata%5Crnaseq%5Cwheat.tar%5Cupload%5CGeneDiffExp%5CPathway%5CW-VS-I_map%5Cmap00950.html) | CA600984, TC443038, TC420057, CV766937, TC444824 |
| 59 | [Fatty acid biosynthesis](../../../../E:%5C实验%5Cdata%5Cdata%5Crnaseq%5Cwheat.tar%5Cupload%5CGeneDiffExp%5CPathway%5CW-VS-I_map%5Cmap00061.html) | TC456607, TC375834, TC404209, TC371037, TC429816, TC398121, TC453487, TC389586, TC378072 |
| 60 | [Peroxisome](../../../../E:%5C实验%5Cdata%5Cdata%5Crnaseq%5Cwheat.tar%5Cupload%5CGeneDiffExp%5CPathway%5CW-VS-I_map%5Cmap04146.html) | TC397738, TC456607, TC454121, BG909274, BJ281583, BE403954, TC387766, TC416757, TC426558, TC440508, BJ271070, CA695289, BM134506, BF484091, TC387116, CA499624, CK162453, CD929875, TC383586, CJ854095, TC456252, BJ258498, TC456784, BJ238421, CJ694502, TC427880, TC400125, TC410058, TC431198, TC454526, TC388522, TC421162, TC384194, CA630163, TC378615 |
| 61 | [Fatty acid metabolism](../../../../E:%5C实验%5Cdata%5Cdata%5Crnaseq%5Cwheat.tar%5Cupload%5CGeneDiffExp%5CPathway%5CW-VS-I_map%5Cmap00071.html) | TC397738, TC416757, BF484091, TC398514, TC387116, CK162453, CJ854095, TC414848, TC383270, TC456252, TC434791, TC456784, BJ238421, TC410058, TC431198, TC421162, CA630163, TC457566, TC378615 |
| 62 | [Natural killer cell mediated cytotoxicity](../../../../E:%5C实验%5Cdata%5Cdata%5Crnaseq%5Cwheat.tar%5Cupload%5CGeneDiffExp%5CPathway%5CW-VS-I_map%5Cmap04650.html) | CJ801437, TC393646, TC398592, TC398731, TC373615, TC426358, TC422348 |
| 63 | [Terpenoid backbone biosynthesis](../../../../E:%5C实验%5Cdata%5Cdata%5Crnaseq%5Cwheat.tar%5Cupload%5CGeneDiffExp%5CPathway%5CW-VS-I_map%5Cmap00900.html) | TC458205, CV770684, TC445333, CA658909, TC398514, TC392247, TC452710, TC427138, TC451949 |
| 64 | [Nicotinate and nicotinamide metabolism](../../../../E:%5C实验%5Cdata%5Cdata%5Crnaseq%5Cwheat.tar%5Cupload%5CGeneDiffExp%5CPathway%5CW-VS-I_map%5Cmap00760.html) | TC390379, TC377438 |
| 65 | Glucosinolate biosynthesis (no map in kegg database) | CJ827429, CK196090, TC406264, TC413263, TC454251 |
| 66 | Biosynthesis of unsaturated fatty acids (no map in kegg database) | TC397738, TC440971, TC456607, TC375268, CK162453, CJ854095, TC456784, TC431198, TC421162, TC405033, TC441995, TC389586 |
| 67 | [Lysine degradation](../../../../E:%5C实验%5Cdata%5Cdata%5Crnaseq%5Cwheat.tar%5Cupload%5CGeneDiffExp%5CPathway%5CW-VS-I_map%5Cmap00310.html) | TC397738, TC398514, TC438132, TC447801, TC457566, TC402069, TC398614, TC372845, TC406725 |
| 68 | [Glycerolipid metabolism](../../../../E:%5C实验%5Cdata%5Cdata%5Crnaseq%5Cwheat.tar%5Cupload%5CGeneDiffExp%5CPathway%5CW-VS-I_map%5Cmap00561.html) | TC382674, TC429062, TC389678, TC400379, TC385164, TC403573, TC443756, TC388158, CV766349, CJ671621, CJ796281, TC457566, TC400690 |
| 69 | [Basal transcription factors](../../../../E:%5C实验%5Cdata%5Cdata%5Crnaseq%5Cwheat.tar%5Cupload%5CGeneDiffExp%5CPathway%5CW-VS-I_map%5Cmap03022.html) | CJ696409, TC423032, TC403157, TC404755, TC386322, BJ217586 |
| 70 | [Limonene and pinene degradation](../../../../E:%5C实验%5Cdata%5Cdata%5Crnaseq%5Cwheat.tar%5Cupload%5CGeneDiffExp%5CPathway%5CW-VS-I_map%5Cmap00903.html) | TC418873, TC414330, CV782061, CD930656, TC405112, TC431175, BQ241127, TC383147, TC408095, TC372040, TC395566, TC421733, AL822449, TC382795, TC422550, TC444399, TC429600, TC427006, TC388950, TC457566, BE213365 |
| 71 | [One carbon pool by folate](../../../../E:%5C实验%5Cdata%5Cdata%5Crnaseq%5Cwheat.tar%5Cupload%5CGeneDiffExp%5CPathway%5CW-VS-I_map%5Cmap00670.html) | TC378601, BE424562, TC380402 |
| 72 | [Fructose and mannose metabolism](../../../../E:%5C实验%5Cdata%5Cdata%5Crnaseq%5Cwheat.tar%5Cupload%5CGeneDiffExp%5CPathway%5CW-VS-I_map%5Cmap00051.html) | TC416438, CV780016, TC390517, CD867411, TC411512, TC410344, TC373613, TC461705, TC438826, TC429062, TC389678, TC400379, TC388751, TC401434, TC376248, TC372654, TC444637, TC379422, TC432185, GH726691, CV767688, TC370885, TC372330, TC395885, CA638808 |
| 73 | [Benzoxazinoid biosynthesis](../../../../E:%5C实验%5Cdata%5Cdata%5Crnaseq%5Cwheat.tar%5Cupload%5CGeneDiffExp%5CPathway%5CW-VS-I_map%5Cmap00402.html) | TC415483, TC381923, CF554463, CJ780546, TC377135, CD929101, TC414330, TC452225, TC436832, TC376758, TC372658, TC429600 |
| 74 | [Tyrosine metabolism](../../../../E:%5C实验%5Cdata%5Cdata%5Crnaseq%5Cwheat.tar%5Cupload%5CGeneDiffExp%5CPathway%5CW-VS-I_map%5Cmap00350.html) | TC428066, CV766916, CA600984, TC414848, TC383270, TC443038, TC420057, TC404719, CV766937, TC427981, TC444824, TC454508 |
| 75 | [Plant-pathogen interaction](../../../../E:%5C实验%5Cdata%5Cdata%5Crnaseq%5Cwheat.tar%5Cupload%5CGeneDiffExp%5CPathway%5CW-VS-I_map%5Cmap04626.html) | TC374940, CA499029, TC421345, DR739731, TC457938, CJ801437, TC460677, TC458720, CJ656770, TC458795, CJ875468, TC368591, BQ241076, CD874590, BE419182, TC393646, TC453678, TC378017, CA614435, TC404620, CK193202, DR733463, TC404384, TC424629, TC406986, TC395090, CJ729769, TC392667, TC401986, CJ628751, TC417755, CJ782333, TC413495, TC376527, TC423010, DR734417, TC435595, TC430930, TC434742, CJ660381, CA696062, TC390057, TC383945, TC457991, TC385164, TC398396, CA730167, TC379343, TC443756, TC387683, TC417263, TC425932, CD871213, TC426629, TC380868, TC398592, TC432623, TC394965, TC369655, TC399013, TC374679, TC461417, TC386410, TC399714, TC416471, CJ629812, TC431768, TC390944, TC409167, TC398805, TC434781, CD869059, TC454280, TC409043, BQ743607, BJ297939, TC396134, BJ307291, TC370751, CK205822, TC399730, TC434894, TC409004, TC443333, TC426486, TC393970, TC445767, TC370912, CA639537, TC398731, TC442140, TC373615, TC426358, TC414243, TC409504, TC415588, TC456781, TC413415, TC377441, TC379313, TC403258, TC419165, TC416780, TC376756, TC449739, TC423893, TC425821, TC411941, TC439015, TC378862, TC422348 |
| 76 | [Alanine, aspartate and glutamate metabolism](../../../../E:%5C实验%5Cdata%5Cdata%5Crnaseq%5Cwheat.tar%5Cupload%5CGeneDiffExp%5CPathway%5CW-VS-I_map%5Cmap00250.html) | BG907133, CV766916, CA600984, TC421363, BQ238873, TC419278, TC451694, TC443038, CV766937, TC433844, TC376414, CK197746, TC385526, TC427981, TC388904, TC369346, TC395497 |
| 77 | [Polyketide sugar unit biosynthesis](../../../../E:%5C实验%5Cdata%5Cdata%5Crnaseq%5Cwheat.tar%5Cupload%5CGeneDiffExp%5CPathway%5CW-VS-I_map%5Cmap00523.html) | TC432176, CA638808 |
| 78 | [Carotenoid biosynthesis](../../../../E:%5C实验%5Cdata%5Cdata%5Crnaseq%5Cwheat.tar%5Cupload%5CGeneDiffExp%5CPathway%5CW-VS-I_map%5Cmap00906.html) | CJ854095, TC459163, TC377274 |
| 79 | [Ubiquinone and other terpenoid -quinone biosynthesis](../../../../E:%5C实验%5Cdata%5Cdata%5Crnaseq%5Cwheat.tar%5Cupload%5CGeneDiffExp%5CPathway%5CW-VS-I_map%5Cmap00130.html) | BE414946, TC390594, TC444824, TC454508 |
| 80 | [Cysteine and methionine metabolism](../../../../E:%5C实验%5Cdata%5Cdata%5Crnaseq%5Cwheat.tar%5Cupload%5CGeneDiffExp%5CPathway%5CW-VS-I_map%5Cmap00270.html) | TC381923, TC403036, TC402072, TC419796, TC452520, TC422649, TC386125, TC397374, CA614435, TC413854, CA600984, TC373702, TC380590, TC427807, TC376351, TC434442, TC443038, TC410108, TC389735, TC392045, TC417106, TC446096, CV766937, CA645113, TC461359, TC374430, TC410586, TC370927, TC383385, TC420248, TC435373, TC444824, TC376756 |
| 81 | [Butanoate metabolism](../../../../E:%5C实验%5Cdata%5Cdata%5Crnaseq%5Cwheat.tar%5Cupload%5CGeneDiffExp%5CPathway%5CW-VS-I_map%5Cmap00650.html) | TC395279, CV766916, TC398514, TC429062, TC389678, TC400379, TC427138, TC432369, TC427981 |
| 82 | [Pentose and glucuronate interconversions](../../../../E:%5C实验%5Cdata%5Cdata%5Crnaseq%5Cwheat.tar%5Cupload%5CGeneDiffExp%5CPathway%5CW-VS-I_map%5Cmap00040.html) | CV763657, TC376248, TC372654, TC452945, TC438376, TC402778 |
| 83 | [Glycine, serine and threonine metabolism](../../../../E:%5C实验%5Cdata%5Cdata%5Crnaseq%5Cwheat.tar%5Cupload%5CGeneDiffExp%5CPathway%5CW-VS-I_map%5Cmap00260.html) | TC395792, TC433068, TC451519, TC378601, TC420057, TC417347, TC385526, TC427981, TC434986 |
| 84 | [Phenylalanine, tyrosine and tryptophan biosynthesis](../../../../E:%5C实验%5Cdata%5Cdata%5Crnaseq%5Cwheat.tar%5Cupload%5CGeneDiffExp%5CPathway%5CW-VS-I_map%5Cmap00400.html) | TC433068, CA600984, TC451519, TC443038, CV766937, TC444824 |
| 85 | [Arachidonic acid metabolism](../../../../E:%5C实验%5Cdata%5Cdata%5Crnaseq%5Cwheat.tar%5Cupload%5CGeneDiffExp%5CPathway%5CW-VS-I_map%5Cmap00590.html) | TC435739 |
| 86 | [Flavone and flavonol biosynthesis](../../../../E:%5C实验%5Cdata%5Cdata%5Crnaseq%5Cwheat.tar%5Cupload%5CGeneDiffExp%5CPathway%5CW-VS-I_map%5Cmap00944.html) | TC442758, TC386323, CD930656, TC431175, TC421733, TC372658 |
| 87 | [Histidine metabolism](../../../../E:%5C实验%5Cdata%5Cdata%5Crnaseq%5Cwheat.tar%5Cupload%5CGeneDiffExp%5CPathway%5CW-VS-I_map%5Cmap00340.html) | TC410987, TC404719, TC457566, TC402668 |
| 88 | [Glycerophospholipid metabolism](../../../../E:%5C实验%5Cdata%5Cdata%5Crnaseq%5Cwheat.tar%5Cupload%5CGeneDiffExp%5CPathway%5CW-VS-I_map%5Cmap00564.html) | DR739350, TC439944, TC448907, TC424266, TC400136, TC401124, TC426518, TC395966, TC408586, TC430501 |
| 89 | [Steroid biosynthesis](../../../../E:%5C实验%5Cdata%5Cdata%5Crnaseq%5Cwheat.tar%5Cupload%5CGeneDiffExp%5CPathway%5CW-VS-I_map%5Cmap00100.html) | CN010359, TC440632, CA603417, TC424480 |
| 90 | [Citrate cycle (TCA cycle)](../../../../E:%5C实验%5Cdata%5Cdata%5Crnaseq%5Cwheat.tar%5Cupload%5CGeneDiffExp%5CPathway%5CW-VS-I_map%5Cmap00020.html) | BE420168, TC410970, TC410656, TC455515, TC425655, TC414039, BQ238531, TC388049, TC389108, TC447801, CA596067, TC419767, TC408079, TC369928, TC387098, TC372580, TC372845 |
| 91 | [Protein export](../../../../E:%5C实验%5Cdata%5Cdata%5Crnaseq%5Cwheat.tar%5Cupload%5CGeneDiffExp%5CPathway%5CW-VS-I_map%5Cmap03060.html) | EV253937, TC412569, TC385009, TC383047, TC390402, TC409343 |
| 92 | [Propanoate metabolism](../../../../E:%5C实验%5Cdata%5Cdata%5Crnaseq%5Cwheat.tar%5Cupload%5CGeneDiffExp%5CPathway%5CW-VS-I_map%5Cmap00640.html) | TC375834, TC398514, TC379082, TC382858, TC434791, TC457566, TC376523 |
| 93 | [Valine, leucine and isoleucine degradation](../../../../E:%5C实验%5Cdata%5Cdata%5Crnaseq%5Cwheat.tar%5Cupload%5CGeneDiffExp%5CPathway%5CW-VS-I_map%5Cmap00280.html) | TC398514, TC434791, TC413263, TC456784, TC444399, TC427138, TC388950, TC457566 |
| 94 | [Ascorbate and aldarate metabolism](../../../../E:%5C实验%5Cdata%5Cdata%5Crnaseq%5Cwheat.tar%5Cupload%5CGeneDiffExp%5CPathway%5CW-VS-I_map%5Cmap00053.html) | TC387981, TC433574, TC403573, TC394567, TC457566, TC406749, CV777843, TC389590, CA638808, TC381436 |
| 95 | [Arginine and proline metabolism](../../../../E:%5C实验%5Cdata%5Cdata%5Crnaseq%5Cwheat.tar%5Cupload%5CGeneDiffExp%5CPathway%5CW-VS-I_map%5Cmap00330.html) | BU099431, TC376772, CA600984, CD884609, CJ930688, TC443038, CK207939, TC383235, TC409179, CV766937, TC370927, TC370350, TC416571, CK197746, TC457566 |
| 96 | [Protein processing in endoplasmic reticulum](../../../../E:%5C实验%5Cdata%5Cdata%5Crnaseq%5Cwheat.tar%5Cupload%5CGeneDiffExp%5CPathway%5CW-VS-I_map%5Cmap04141.html) | TC442697, TC427632, TC391681, CV775489, TC383174, TC445354, TC458773, TC424671, TC449504, BE418867, TC441472, TC459325, TC398127, TC443575, CA733951, CK209362, CJ681314, TC406096, TC458283, TC461622, BE427240, TC380882, CA604422, TC448163, CV780698, TC397964, TC431259, TC423880, TC372389, EV253937, TC444619, TC398385, BQ483856, BQ842471, TC391916, TC419273, TC440412, TC412212, TC423354, TC419510, TC391411, TC403588, TC405511, TC400051, CA726837, TC388232, TC371546, CJ629812, TC415685, TC427784, TC397027, TC382053, TC457772, TC381899, TC400656, TC376603, TC412569, TC416976, TC442911, TC383047, TC423091, TC372553, TC396559, TC394820, TC409343, TC369983, CK205670 |
| 97 | [beta-Alanine metabolism](../../../../E:%5C实验%5Cdata%5Cdata%5Crnaseq%5Cwheat.tar%5Cupload%5CGeneDiffExp%5CPathway%5CW-VS-I_map%5Cmap00410.html) | TC407325, TC434791, TC420057, TC457566 |
| 98 | [Pyruvate metabolism](../../../../E:%5C实验%5Cdata%5Cdata%5Crnaseq%5Cwheat.tar%5Cupload%5CGeneDiffExp%5CPathway%5CW-VS-I_map%5Cmap00620.html) | BE420168, TC412551, TC371172, TC455515, TC375834, TC398514, TC376888, TC379082, TC444921, TC419621, TC369064, TC375733, TC400250, TC392363, TC383143, TC457566, TC433532, TC376523, TC372580, TC400056 |
| 99 | [Pentose phosphate pathway](../../../../E:%5C实验%5Cdata%5Cdata%5Crnaseq%5Cwheat.tar%5Cupload%5CGeneDiffExp%5CPathway%5CW-VS-I_map%5Cmap00030.html) | CV780016, TC373613, TC385738, TC449256, TC388751, TC439821, TC387501, TC379422, TC432185, TC370384, TC406505, CJ625718 |
| 100 | [Proteasome](../../../../E:%5C实验%5Cdata%5Cdata%5Crnaseq%5Cwheat.tar%5Cupload%5CGeneDiffExp%5CPathway%5CW-VS-I_map%5Cmap03050.html) | BQ239765, CJ707252, TC394411, TC452691, TC377021, CX536025, TC448305, TC371641, TC400165, TC379459, TC419703, TC420420, TC397562, CA695866 |
| 101 | [Phagosome](../../../../E:%5C实验%5Cdata%5Cdata%5Crnaseq%5Cwheat.tar%5Cupload%5CGeneDiffExp%5CPathway%5CW-VS-I_map%5Cmap04145.html) | CA635103, TC436405, TC458773, BE517058, CJ655278, TC399452, CV759297, TC371242, TC372020, CA501474, TC419261, TC379171, TC423537, CJ714840, TC402426, TC426186, BE585841, TC390630, TC407456, TC399327, TC418693, BJ267564, TC435055, TC412569, TC383047, TC372530, TC460726, TC397997, CK152172, TC409343, TC413118 |
| 102 | [Porphyrin and chlorophyll metabolism](../../../../E:%5C实验%5Cdata%5Cdata%5Crnaseq%5Cwheat.tar%5Cupload%5CGeneDiffExp%5CPathway%5CW-VS-I_map%5Cmap00860.html) | TC420816, TC425291, TC426838, TC424621, TC452710 |
| 103 | Biosynthesis of secondary metabolites (no map in kegg database) | BE414946, CA663181, TC418073, TC415483, TC381923, TC458205, TC369189, TC422841, TC442758, CF554463, TC404452, TC382807, CV780016, TC447579, TC385962, TC402072, TC372549, TC386707, TC433013, TC372867, TC387981, CV770684, TC454121, TC452520, TC410987, CJ780546, CV769814, TC375760, TC433068, TC418873, TC452793, TC445333, BE420168, TC377135, CD929101, TC381862, CJ827429, TC386323, TC395279, TC420816, TC392875, TC410970, TC439472, CA682500, TC376651, TC433574, TC441915, TC414330, TC396657, TC371172, TC413199, BG909274, CN013015, TC410656, BJ281583, TC422649, CA658909, BG907133, TC423533, CV782061, CJ625788, TC452225, TC424987, CD930656, TC448840, BE403954, TC405112, TC431175, TC455515, TC456247, TC386125, TC387817, TC397374, CV780986, TC373613, CA612260, BQ609463, BQ241127, TC392283, TC425655, TC372823, TC385738, TC440508, CD882790, TC376772, TC385013, TC429713, TC413854, DR739303, DR739299, CA695289, CA600984, TC434396, TC384357, TC428778, TC414039, EB514888, TC404914, TC378324, TC380590, BJ247780, TC425291, TC451519, CK196090, TC398514, TC436832, TC376888, TC395069, TC383147, TC449256, CA702126, TC435364, TC379082, TC419550, TC408095, TC392247, TC398026, TC382858, TC373133, CJ854095, TC414848, TC390594, TC459008, CF133957, TC429062, TC383270, AJ890242, TC426838, TC404420, TC406264, TC389678, TC387064, TC389137, TC434791, TC405849, BQ238531, TC407076, BJ258498, TC444921, TC372040, TC400379, TC395566, BQ247004, TC443038, TC376758, TC421733, CD874875, CJ729932, TC386401, TC388751, TC388049, TC413263, TC404179, TC403573, TC410108, TC394567, TC385927, BE217043, TC404786, CJ826490, TC438587, TC389735, TC456784, TC390762, TC422583, AL822449, TC419621, TC383235, TC400388, TC405773, TC375124, TC389044, TC424621, TC382795, TC391116, TC452710, CJ694502, TC413990, TC427880, TC439821, TC419090, TC387501, TC400125, TC426916, TC392045, TC422550, TC410159, TC372658, TC378601, TC372761, TC389108, TC379422, TC444399, TC391564, TC417106, TC420057, TC381068, TC432185, TC440632, TC454526, TC447801, TC372953, TC442727, TC391900, TC385326, TC379241, TC429600, TC399442, CA596067, TC388158, TC370392, TC419767, TC446096, TC382734, CV766937, TC427138, TC421162, TC374741, TC408079, TC430821, TC371073, TC432369, TC384637, TC427006, TC453352, TC434510, TC403504, CA603417, CJ521228, TC409987, TC388950, TC433844, TC451949, TC424480, TC370507, TC434947, TC431814, TC376414, TC404151, TC370350, TC370384, TC430788, TC454251, TC377760, TC404410, TC416571, TC399341, GH726691, TC369928, CD911155, TC376606, TC457566, TC388627, TC387098, CV767688, CJ813329, TC374948, TC438376, CV777843, TC421880, TC444220, TC395813, TC386944, TC444824, TC388904, TC403549, TC402423, TC369346, TC370885, TC410041, TC374447, TC372330, TC432176, TC376523, TC372580, TC371145, TC407651, BE213365, TC406505, TC419552, TC380402, TC389586, CA638808, TC372845, CA682223, CJ625718, TC402668 |
| 104 | [Glycolysis / Gluconeogenesis](../../../../E:%5C实验%5Cdata%5Cdata%5Crnaseq%5Cwheat.tar%5Cupload%5CGeneDiffExp%5CPathway%5CW-VS-I_map%5Cmap00010.html) | CV780016, BE420168, TC371172, TC455515, TC373613, TC385738, TC376888, TC379082, TC414848, TC429062, TC383270, TC389678, TC405849, TC407076, TC444921, TC400379, TC388751, TC403573, TC419621, TC439821, TC372761, TC379422, TC432185, TC379241, TC388158, TC370384, GH726691, TC376606, TC457566, CV767688, TC376523, TC406505, CA682223 |
| 105 | [Glyoxylate and dicarboxylate metabolism](../../../../E:%5C实验%5Cdata%5Cdata%5Crnaseq%5Cwheat.tar%5Cupload%5CGeneDiffExp%5CPathway%5CW-VS-I_map%5Cmap00630.html) | TC410970, TC410656, TC387766, TC425655, TC414039, BQ238531, TC388049, TC389108, CA596067, TC408079, TC384194, TC372580 |
| 106 | [Photosynthesis](../../../../E:%5C实验%5Cdata%5Cdata%5Crnaseq%5Cwheat.tar%5Cupload%5CGeneDiffExp%5CPathway%5CW-VS-I_map%5Cmap00195.html) | TC400260 |
| 107 | [Oxidative phosphorylation](../../../../E:%5C实验%5Cdata%5Cdata%5Crnaseq%5Cwheat.tar%5Cupload%5CGeneDiffExp%5CPathway%5CW-VS-I_map%5Cmap00190.html) | CV759297, TC383585, TC444146, TC419261, CJ714840, TC397994, TC422842, TC426186, DR735108, TC410182, TC399327, TC419767, TC395672, TC406827, TC387194, TC375539, TC368687, TC455736, TC414155, TC384674, TC377190, TC392564, TC411471, BJ282766, TC375918, TC411015 |
| 108 | [Photosynthesis - antenna proteins](../../../../E:%5C实验%5Cdata%5Cdata%5Crnaseq%5Cwheat.tar%5Cupload%5CGeneDiffExp%5CPathway%5CW-VS-I_map%5Cmap00196.html) | TC439472 |
| 109 | Metabolic pathways (no map in kegg database) | BE414946, TC418073, TC381923, TC458205, CK161463, TC369189, TC422841, TC395792, EB512907, TC442758, TC427632, TC458807, CN010359, TC404452, TC403036, TC397738, CV780016, DR739350, TC385962, TC402072, TC408041, TC386707, TC456607, TC380772, NP234380, TC372867, TC387981, CV770684, CJ628257, TC454121, TC419796, TC445354, TC385909, CN010468, TC410987, TC375760, TC433068, TC434630, TC424671, TC435231, CK210510, TC452793, TC445333, TC425657, BJ256335, BE420168, BE586132, TC400224, TC410376, TC381862, TC460760, TC395279, CV774255, TC410970, TC412551, TC439472, TC428066, DR733517, TC393877, CA682500, TC376651, TC387751, TC433574, CA733413, TC441915, TC401715, TC406579, BE517058, TC388136, TC416007, TC396657, TC371172, TC413199, BG909274, TC446901, CN013015, TC410656, BJ281583, CA658909, BG907133, TC423533, CK163142, TC382674, TC424987, CD930656, TC448840, TC448907, BE403954, GH731884, BU099431, TC431175, TC455515, CJ690976, TC387766, BQ167150, TC387817, TC460259, TC397374, CV780986, TC424266, TC373613, CA612260, BQ609463, TC416757, CV759297, BQ241127, CA614435, BE517280, TC425655, CV766916, TC372823, TC385738, TC451272, TC440508, TC376772, TC385013, TC429713, TC413854, TC448275, DR739303, DR739299, CA695289, TC388505, CA600984, TC407325, TC434396, BM134506, TC384357, TC375834, TC428778, TC414039, TC406096, TC404914, TC369899, TC421363, TC378324, TC373702, TC380590, TC455080, TC379965, BJ247780, TC451519, BF484091, TC398514, TC376888, TC395069, TC444588, CJ707239, TC397909, TC387116, TC449256, CD875411, BQ241509, TC383227, CA702126, TC435364, TC407672, CJ854725, TC379082, TC430163, TC419550, CD884609, BQ238873, TC392247, TC427807, CA501474, TC398026, CK162453, TC419261, BJ259409, TC460397, TC419278, TC423537, CJ854095, TC414848, TC390594, TC430544, CJ714840, TC459008, CF133957, TC429062, TC383270, TC372701, TC443814, TC394589, TC370633, TC456252, TC390379, TC409187, TC389678, TC434791, TC376351, TC405849, TC377438, BQ238531, TC407076, TC451694, BJ258498, TC444921, TC400379, TC402080, BQ247004, TC390016, TC434442, TC443038, TC419957, TC421733, CD874875, CJ854427, CJ729932, TC399841, CA647610, TC425623, TC386401, TC433422, TC391946, TC388751, TC385164, TC388049, TC408608, TC410126, TC413263, TC403573, TC410108, BE516446, TC394567, TC443756, BE217043, TC404786, TC392033, CA499357, TC420020, TC401434, CK207939, CJ826490, TC438587, TC456784, TC402426, TC390762, TC422583, TC419621, TC369064, BG905284, TC383235, TC400388, TC426186, TC447848, TC405773, BJ238421, TC375726, TC389044, TC424621, TC376248, TC383139, TC391116, TC426629, TC449510, TC382786, TC375214, TC452710, TC431603, CJ694502, TC427880, TC454714, TC400136, TC372654, TC439821, TC461977, TC399245, TC420201, TC413124, TC419090, BE422762, TC389993, BQ842471, TC387501, TC409179, TC378782, TC400125, TC426916, TC407490, TC410058, TC392045, TC410159, TC398536, TC378601, TC370347, TC431198, TC376490, TC389108, TC379422, TC406106, TC390285, TC444399, TC391564, TC417106, TC420057, TC381068, TC432185, TC440632, TC391411, TC454526, TC456755, TC375733, TC447801, TC372953, TC399327, TC442727, TC391900, TC400562, TC401124, BJ283132, TC385326, GH731123, TC379241, TC429600, TC400250, TC399442, CA596067, TC388232, TC388158, TC370392, TC419767, TC446096, TC382734, TC385701, TC388522, TC375234, TC428112, CV766937, TC427138, BJ267564, TC421162, TC374741, CV766349, TC408079, TC373251, TC380416, TC418269, TC368548, TC411908, TC430821, AL827131, TC371073, TC432369, TC419215, TC384194, TC384637, TC434510, TC403504, TC426518, CA603417, CJ521228, TC394661, TC409987, CJ671621, TC395672, TC423241, TC388950, TC433844, TC451949, CA645113, TC461359, TC406827, TC387194, TC404209, TC374430, TC424480, TC396981, TC410586, CJ796281, TC417347, TC434947, TC368549, CA630163, TC446038, TC376414, TC404151, TC370927, TC445212, TC369373, TC370350, TC400401, TC370384, TC395966, TC384553, TC427537, TC430788, TC377760, TC371037, TC419297, TC456959, TC452945, TC416571, TC436483, TC383143, TC399341, CK197746, TC384877, TC462041, GH726691, TC454407, TC369928, TC396650, CK193510, CD911155, TC376606, TC368687, TC399008, TC457566, BE424562, TC383385, CA635612, TC420248, TC388627, TC420502, TC455736, TC419181, TC414155, TC427981, TC406749, TC435739, TC387098, TC452779, CV767688, TC435373, TC384674, TC377190, CD935065, TC397176, TC398458, GH732878, TC408586, TC392564, TC397500, TC388819, CJ813329, TC369844, TC374948, EB512568, TC430501, CK155765, TC434986, TC438376, TC400260, TC429816, TC418683, TC425830, TC411471, TC411008, TC408462, CV777843, TC421880, TC457112, TC395813, TC386944, TC444824, TC388904, TC376820, TC402423, CJ815867, TC369346, TC395497, TC371125, TC432261, TC433532, TC370885, TC378615, TC410041, TC458892, TC374447, TC398984, TC372330, TC369548, TC398121, TC432176, TC384657, TC376523, CA602703, TC372580, TC376756, TC371145, TC407651, BE213365, TC409077, BJ282766, TC381628, TC406505, TC415336, TC375918, TC453487, TC406193, TC419552, TC380402, TC370791, TC381434, TC389586, CA638808, TC372845, TC378072, TC383739, TC454508, CA682223, TC381436, TC392232, CJ625718, TC402668 |
| 110 | [Ribosome](../../../../E:%5C实验%5Cdata%5Cdata%5Crnaseq%5Cwheat.tar%5Cupload%5CGeneDiffExp%5CPathway%5CW-VS-I_map%5Cmap03010.html) | TC441343, TC451511, TC444586, TC415942, TC379942, TC450285, TC398862, TC435909, TC410078, TC373994, CK211589, TC421914, TC409459, TC435799, TC444138, TC381988, CA640430, TC391785, TC435281, TC416906, TC416442, TC438863, TC391613, TC440066, TC408312, TC418091, BI750421, TC398970, TC403580, TC409208, TC456619, TC370044, TC417388, CV761628, TC390489, TC431722, TC378271, TC370114, TC434804, TC435825, TC383909, TC410066, TC404843, TC407051, TC449724, TC399471, TC389168, TC400108, TC391948, TC423182, TC381619, TC425690, TC413571, TC457126, TC371970, TC414090, TC459656, TC382737, CA598430, TC376874, TC405475, TC445166, TC393820, TC372667, TC402545, TC417077, TC384735, TC395298, TC375431, TC413027, TC387861, BJ313676, TC406807, TC373259, TC418414, TC409599, TC372664, TC419981, TC380433, TC418850, TC388718, TC449463, TC396451, TC403328, TC440819 |
| 111 | [Carbon fixation in photosynthetic organisms](../../../../E:%5C实验%5Cdata%5Cdata%5Crnaseq%5Cwheat.tar%5Cupload%5CGeneDiffExp%5CPathway%5CW-VS-I_map%5Cmap00710.html) | BE420168, TC412551, TC371172, TC455515, TC373613, CA600984, TC376888, TC449256, TC419278, TC451694, TC444921, TC443038, TC419621, TC369064, TC432185, TC375733, TC400250, CV766937, TC383143, CV767688, TC395497, TC433532, TC372580 |
