# Supplementary material for: Global Analysis of Differentially Expressed Genes and Proteins in the Wheat Callus Infected by Agrobacterium tumefaciens
Source: PLoS One. 2013 Nov 20;8(11):e79390. doi: 10.1371/journal.pone.0079390 (PMC3835833; doi:10.1371/journal.pone.0079390)
Supplement: File S10 — Comparison of expression level between the data of real-time PCR and RNA-seq. (DOC) [file pone.0079390.s010.doc]

**File S10 Comparison of expression level between the data of real-time PCR and RNA-seq**

| **DEGs** | **2^-ΔΔCT^**  **(Real-time PCR)** | **|Log2|ratio (infected/control) (RNA-seq)** |
| --- | --- | --- |
| CF133353 | 8.036733** | 13.55414 |
| CJ654139 | 10.50023** | 13.42353 |
| TC416495 | 87.0334** | 13.29479 |
| TC451603 | 17.21286** | 13.24985 |
| BJ236765 | 10.89963** | 13.13309 |
| TC458205 | 12.6376** | 11.92889 |
| TC379055 | 22.04192** | 11.2469 |
| TC419727 | 3.614534** | 3.101984 |
| TC388136 | 1.481546 | 2.196725 |
| TC434396 | 0.18726** | 1.831048 |
| TC419278 | 4.032211** | 1.587472 |
| CK201148 | 0.962055 | 1.325723 |
| TC389044 | 1.715797* | 1.315388 |
| TC370347 | 1.74282 | 1.17555 |
| TC379241 | 2.943157** | 1.108033 |
| TC389590 | 1.090209 | -1.05259 |
| TC406505 | 0.121779** | -1.09083 |
| TC411471 | 0.9505 | -1.32428 |
| TC420420 | 0.460811* | -1.334001 |
| CK155765 | 0.357996 | -1.36399 |
| CV767688 | 0.293805 | -1.49137 |
