# Supplementary figures and images for: Global Analysis of Differentially Expressed Genes and Proteins in the Wheat Callus Infected by Agrobacterium tumefaciens
Source: PLoS One. 2013 Nov 20;8(11):e79390. doi: 10.1371/journal.pone.0079390 (PMC3835833; doi:10.1371/journal.pone.0079390)

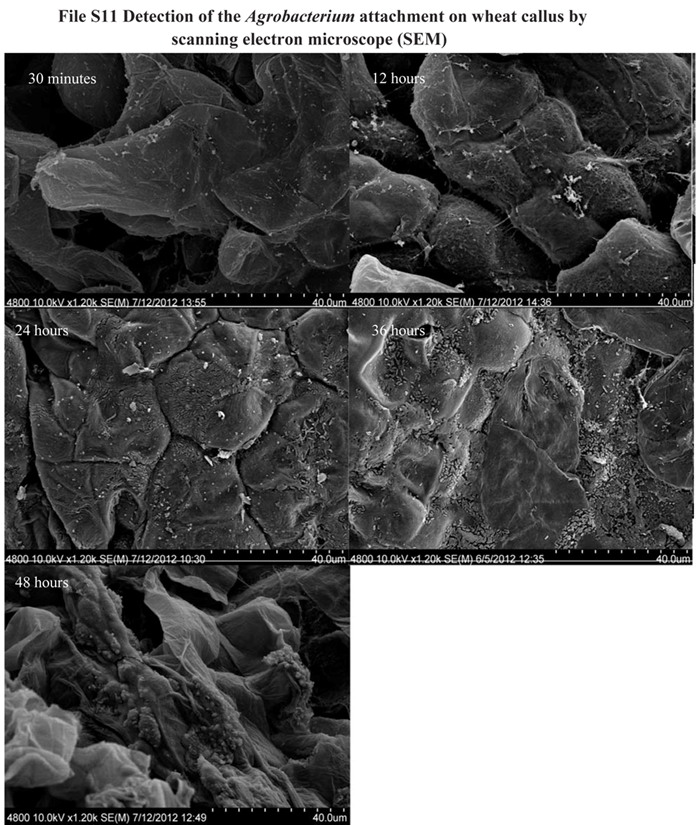

Supplement: File S11 — Detection of the Agrobacterium attachment on wheat callus by scanning electron microscope (SEM). The adsorption of Agrobacterium to wheat callus after co-culture for 30 minutes, 12 hours, 24 hours, 36 hours, and 48 hours. (TIF) [file pone.0079390.s011.tif]
